# Traceless cysteine-linchpin enables precision engineering of lysine in native proteins

**DOI:** 10.1038/s41467-022-33772-1

**Published:** 2022-10-13

**Authors:** Neelesh C. Reddy, Rajib Molla, Pralhad Namdev Joshi, Sajeev T. K., Ipsita Basu, Jyotsna Kawadkar, Neetu Kalra, Ram Kumar Mishra, Suman Chakrabarty, Sanjeev Shukla, Vishal Rai

**Affiliations:** 1 Department of Chemistry, Indian Institute of Science Education and Research Bhopal, Bhopal Bypass Road, Bhauri, Bhopal, 462 066 M.P. India; 2 Department of Biological Sciences, Indian Institute of Science Education and Research Bhopal, Bhopal Bypass Road, Bhauri, Bhopal, 462 066 M.P. India; 3 Department of Chemical and Biological Sciences, S. N. Bose National Centre for Basic Sciences, Block-JD, Sector-III, Salt Lake, Kolkata, 700 106 W.B. India; 4 School of Bioengineering, VIT Bhopal, India

**Keywords:** Chemical modification, Synthetic chemistry methodology, Chemical modification

## Abstract

The maintenance of machinery requires its operational understanding and a toolbox for repair. The methods for the precision engineering of native proteins meet a similar requirement in biosystems. Its success hinges on the principles regulating chemical reactions with a protein. Here, we report a technology that delivers high-level control over reactivity, chemoselectivity, site-selectivity, modularity, dual-probe installation, and protein-selectivity. It utilizes cysteine-based chemoselective Linchpin-Directed site-selective Modification of lysine residue in a protein (LDM_C-K_). The efficiency of the end-user-friendly protocol is evident in quantitative conversions within an hour. A chemically orthogonal C-S bond-formation and bond-dissociation are essential among multiple regulatory attributes. The method offers protein selectivity by targeting a single lysine residue of a single protein in a complex biomolecular mixture. The protocol renders analytically pure single-site probe-engineered protein bioconjugate. Also, it provides access to homogeneous antibody conjugates (AFC and ADC). The LDM_C-K_-ADC exhibits highly selective anti-proliferative activity towards breast cancer cells.

## Abstract (web summary)

Controlling the selectivity of the chemical modification of a genetic-manipulation-free protein is currently problematic. Here the authors report a method using cysteine-based chemoselective Linchpin Directed site-selective Modification of the lysine residue in a protein.

## Introduction

The functional well-being of a living organism thrives on the seamless coordination of diverse biomolecules. Hence, molecular-level intervention becomes vital for rescue whenever a component malfunctions. For instance, the ongoing development of inhibitors^1–4^ and degraders^5–7^ provides access to an extended landscape of undruggable proteome. Assisted by chemoproteomics, the leads often emerge from the top-down screening of a ligand library against the proteome. Such activity-based protein profiling also provides vital insight into the off-target modifications responsible for multiple failures. Hence, controlling the precision while targeting a site in a complex protein-derived ecosystem needs urgent attention. Initially, it requires a profound knowledge of operating principles in the bioconjugation of model peptides^8,9^ or isolated proteins. Subsequently, we can translate the concepts while understanding the impact from additional layers of complexity. Alongside, such platforms are desired to probe biological systems^10^ and meet the requirements of protein-based healthcare tools and therapeutics^11,12^. For example, the challenge of heterogeneity and ambiguous conjugation sites has been the most significant roadblock to developing antibody-drug conjugates (ADCs) for directed cancer chemotherapeutics. A chemical platform that empowers the regulation of homogeneity, drug-to-antibody ratio, and site-of-drug-conjugation is of immense value in this perspective.

The biochemical single-site engineering of chemically orthogonal handles such as carbonyls creates an excellent opportunity for their precise conjugation^13,14^. However, controlling the selectivity in the chemical modification of a genetic manipulation-free protein faces a spectrum of challenges. The magnitude depends on whether the protein is isolated or accompanied by other biomolecules. For example, we and others demonstrated that the labeling of reactivity hotspots provides efficient tools for chemoselective and site-selective modification of isolated proteins^15–27^. However, such methods are not equipped to deal with the drastic increase in competing functional groups presented by a complex mixture of proteins. Besides redefining the chemoselectivity and site-selectivity, it also presents the question of protein-selectivity for achieving absolute precision. In this perspective, the non-covalent ligands bound to an electrophilic warhead have been able to offer an interesting case^28,29^. Besides, residue-specific labeling emerged as a robust tool for targeting N-Cys^30–33^ and N-Gly^34^. The impact could be multifold if a chemical technology can exhibit a combination of chemoselectivity, site-selectivity, modularity, and protein-selectivity to constitute a platform that can address the precision engineering of proteins in a complex mixture.

**Fig. 1:**
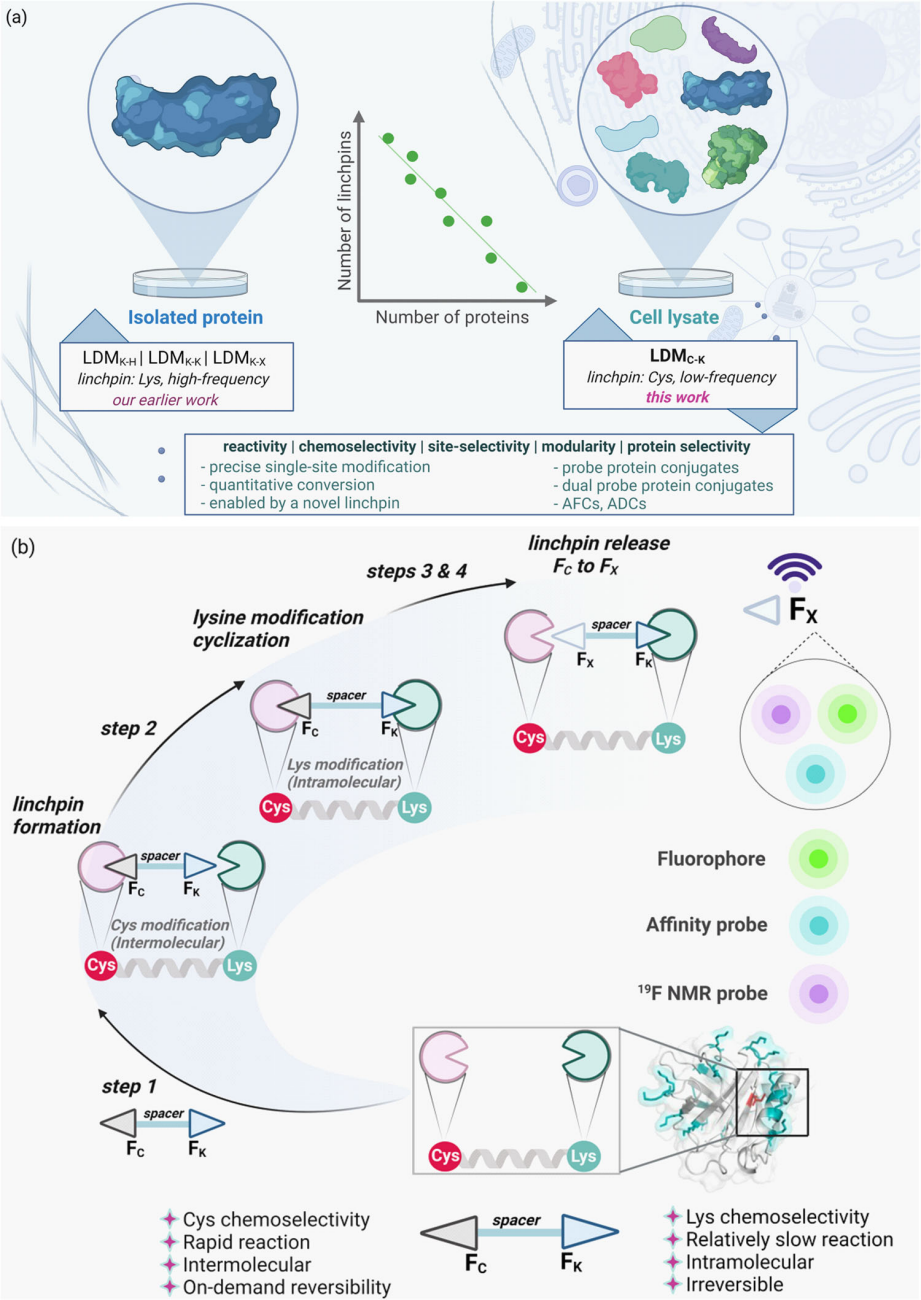
Chemical technology for precision engineering of native proteins. **a** Requirement: low-frequency residue-derived proximity regulator to create a unique targetable landscape and opportunity for simultaneous regulation of reactivity, chemoselectivity, site-selectivity, modularity, and protein-selectivity. **b** Hypothesis: Cys-linchpin-directed modification of Lys residue (LDM_C-K_). Step 1, *linchpin formation*: rapid, intermolecular, chemoselective reaction of F_C_ with Cys; step 2, *cyclization*: intramolecular, irreversible, site-selective reaction of F_K_ with Lys; step 3, *F*_*C*_ *to F*_*X*_: sequential C-S and C-C bond dissociation; step 4, *late-stage modification*.

We recently demonstrated that a chemical method could deliver single-site protein modification irrespective of the residue’s reactivity order. Here, a lysine-derived linchpin-directed modification (LDM®) results in the labeling of a His (LDM_K-H_, LDM_K-X_), Lys (LDM_K-K_), and Asp (LDM_K-X_)^35–37^. The high-frequency Lys residues regulate the number of linchpins and direct the modification of the other residue. It is noteworthy that Lys-derived linchpin would offer excellent opportunities with an isolated protein (Fig. 1a). As we increase the number of proteins in the mixture, it becomes exceedingly challenging to identify the LDM reagent suitable for precise modification of a single protein. In this perspective, we hypothesized that a method could be valuable if it derives linchpin from a low-frequency residue to modify a high-frequency site (steps 1–4, Fig. 1b). Such an expedition would require the development of a chemoselective electrophile that could enable a rapid reaction with the identified residue at low micromolar concentration. We argued that cysteine could be an ideal residue to anchor the linchpins with F_C_ if we can solve a combination of two non-trivial challenges. At first, the linchpin must be released under physiological conditions after the completion of the bioconjugation. Secondly, the linchpin-released functional group must render a chemically unique handle for late-stage modifications. We realized that the popular cysteine-selective electrophiles would not serve the purpose^38^. It is not surprising as they evolved to deliver enhanced stability of the thiol-adduct. On the contrary, there are only a few electrophilic reagents where reversibility was pursued in addition to chemoselectivity^39^. Hence, it became necessary to develop one that can meet the requirements of our hypothesis.

Here, we report a Cys-derived linchpin-directed single-site modification of lysine (LDM_C-K_) to meet the technological demands. The success of site-selective LDM_C-K_ required an appropriate multifunctional reagent (F_C_-spacer-F_K_, Fig. 1b). At first, we developed nitroolefin (F_C_) as an electrophile capable of rendering a rapid chemoselective reaction with cysteine (>99% conversion). Subsequently, we identified an acylating group (F_K_) for a relatively slow, irreversible, and chemoselective reaction with lysine. However, the success of LDM_C-K_ required us to develop another methodology for reverting the nitroolefin thio-Michael adduct. Also, such a C-S bond dissociation should render a functional group amenable to orthogonal late-stage modification. In this perspective, we developed a one-pot sequential protocol for retro-Michael/retro-Henry reaction that operates under mild conditions with remarkable efficiency over two steps. Importantly, it offers an aromatic aldehyde to enable the capture, enrichment, purification, and late-stage installation of probes. At first, we demonstrate that LDM_C-K_ offers chemoselectivity and site-selectivity to render single-site modification of a protein. The simulation results gave insight into how the design, length, and adaptable rigidity of the spacer regulate the bioconjugation site. Subsequently, we demonstrate that the method translates efficiently to the protein mixture or cell lysate and addresses the question of protein selectivity. Besides, we present a user-friendly protocol for isolating analytically pure protein tagged with the probe of interest at a single site. In this modular platform, the reagent design enables modification of a lysine beyond protein-defined reactivity order. We demonstrate that LDM_C-K_ could deliver the single-site installation of various biophysical probes. The chemical and functional space expansion for selective targeting has also opened gateways for dual-probe bioconjugates. We established it through LDM_C-K_-enabled installation of a fluorophore, affinity tag, or NMR probe coupled with chemoselective single-site installation of the second tag at the linchpin site to ensure proximal precision dual-labeling of the protein. Further, we demonstrate that the methodology enables the synthesis of homogeneous ADC capable of highly selective anti‐proliferative activity towards HER‐2 expressing SKBR‐3 breast cancer cells.

## Results

### Design and synthesis of LDM_C-K_ reagent

**Fig. 2:**
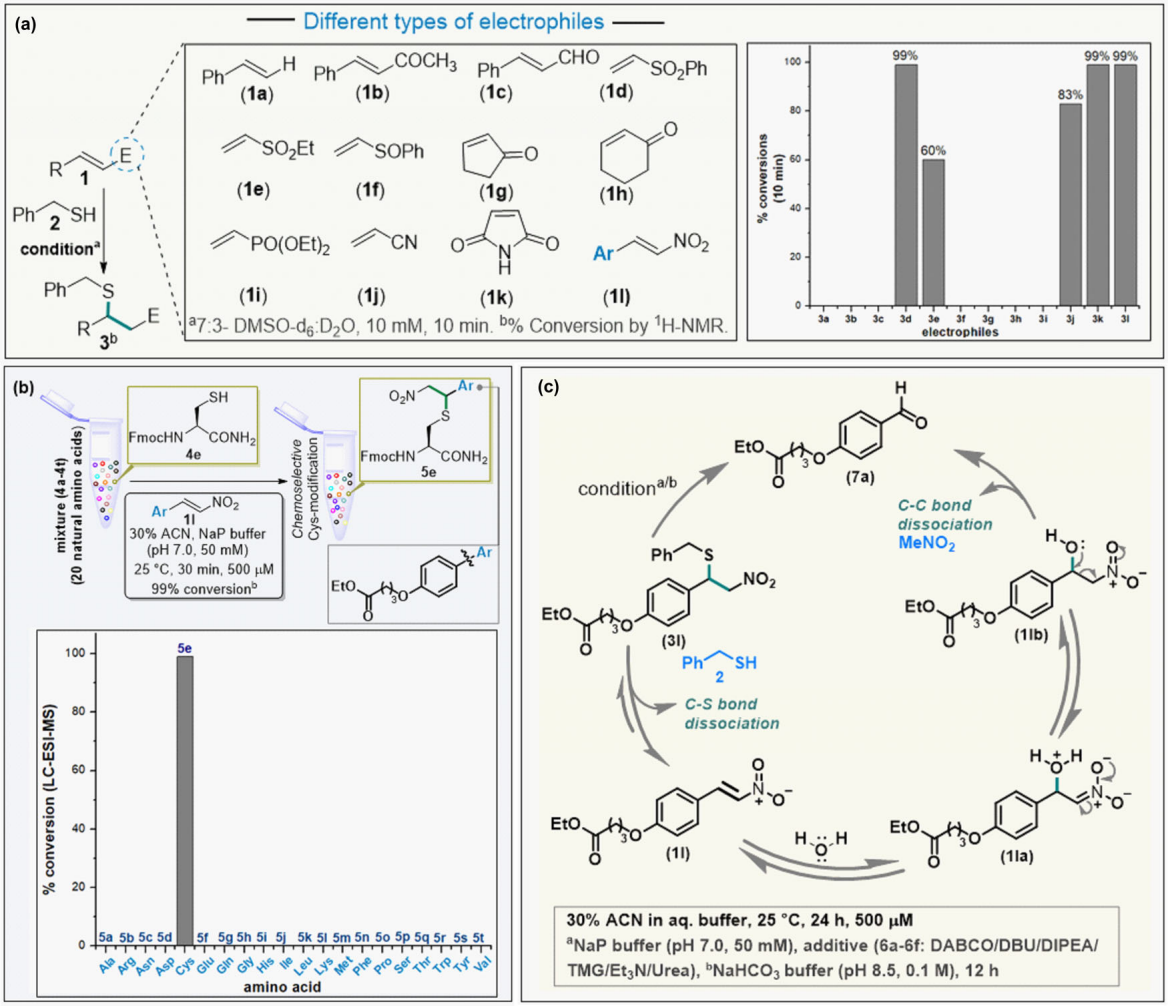
Development of Cys-based linchpin, F_C_. **a** Establishing the reactivity for C-S bond formation (Ar, -Ph-4-O(CH_2_)_3_CO_2_Et). **b** Validating the chemoselectivity for C-S bond formation with Cys. **c** Establishing C-S/C-C bond dissociation to generate a functional group, F_X_, amenable for late-stage transformations.

The design of the LDM_C-K_ reagent involved three critical building blocks: F_C_, F_K_, and the spacer. We initiated by searching for an electrophile that can result in a rapid chemoselective reaction with cysteine (F_C_). Besides, it must offer the attribute of on-demand C-S bond dissociation to render an orthogonally maneuverable functional group. In this perspective, we selected a set of soft electrophiles (**1a-1l**) bearing polarized double bonds and diffused electron densities for screening with thiol-based reagent **2** (Fig. 2a and Supplementary Figs. 15–26, also see Supplementary Table 1). A few electrophiles (**1a–c**, **1f**–**i**) were non-reactive under the reaction conditions. Interestingly, vinyl sulfone **1e** resulted in 60% conversion (**3e**). While acrylonitrile (**1j**) has shown better reactivity (**3j**, 83% conversion), another vinyl sulfone derivative, **1d** led to quantitative yields. Keeping the synthetic maneuverability constraints in mind, we furthered our screening. The maleimide (**1k**) also worked well to result in high conversions to the thio-Michael adduct. However, it is challenging to trigger retro-Michael addition or nucleophilic substitution in this case without interference from side reactions, particularly hydrolysis^38^. To our delight, nitroolefin (**1l**) resulted in quantitative conversions (Fig. 2a). Further, we mixed twenty amino acids with unprotected side-chain and found it to exhibit exclusive Cys-selectivity (Fig. 2b and Supplementary Figs. 27–28, also see Supplementary Table 2). Hence, we selected the thio-Michael adduct with nitroolefin (**3l**) to test it for the C-S bond dissociation reaction under mild aqueous conditions (Fig. 2c).

The C-S bond dissociation proceeded with moderate efficiency through retro-Michael pathway over time or in the presence of DABCO, TMG, and urea (Fig. 2c, also see Supplementary Table 3 and Supplementary Figs. 29–37). The regenerated nitroolefins can be used for late-stage installation of probes with nucleophilic thiol handles. However, such a system is more suited for applications involving reversibly installed tags but not for biologics such as ADCs. Hence, it was important for us to develop a method for the transformation of F_C_ to another chemically orthogonal handle (F_X_) that can result in stable probe conjugates. The subsequent transformation of F_C_, such as retro-Henry pathway involving C-C bond dissociation, remained elusive in the previous cases. However, DBU, DIPEA, and TEA enabled the retro-Henry reaction to form aldehyde in moderate conversions (14–22% in 24 h, Supplementary Table 3). After a thorough screening, we were delighted to establish a one-pot sequence of reactions involving both C-S and C-C bond dissociation under mild conditions (55% within 12 h, bicarbonate buffer, pH 8.5, Supplementary Table 3). The chemoselectivity with thiol bioconjugation and chemically orthogonal dissociation rendering F_X_ made nitroolefin the preferred group to be examined as the F_C_ component.

**Fig. 3:**
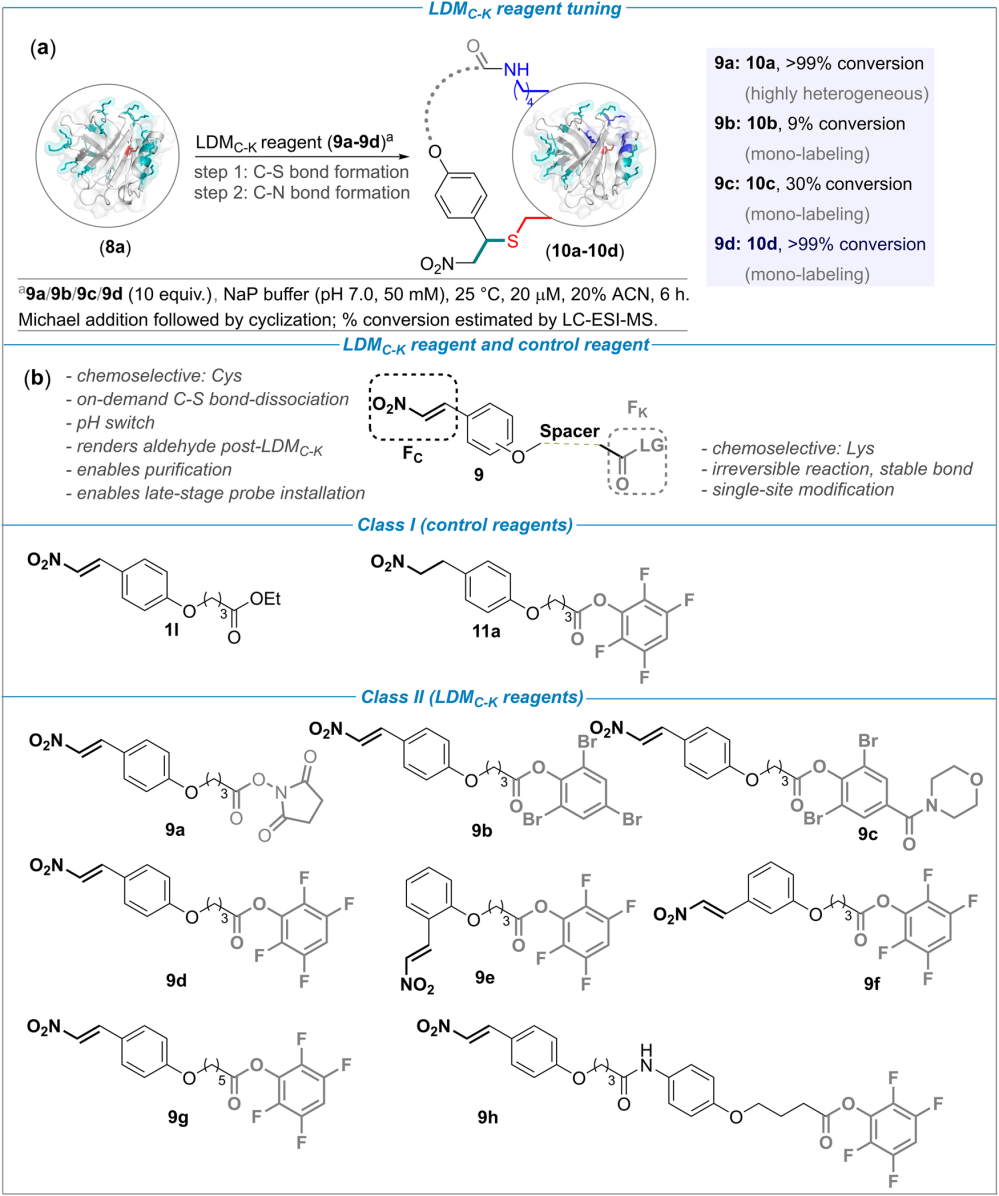
Development of LDM_C-K_ reagents. **a** Initial exploration with BLGA to establish the design of F_K_ and relative reactivity with F_C_. **b** The design and synthesis of potential LDM_C-K_ and control reagents (for synthesis, see Supplementary Figs. 1–10).

The aryl group in these test studies offers the synthetic maneuverability for subsequent installation of F_K_ and spacer. This feature is vital as the screening of the second electrophilic group, F_K_, needs to be performed in conjugation with the F_C_ and spacer. This leap of faith was necessary to validate the chemoselectivity and site-selectivity along with the relative reactivity order of F_C_ versus F_K_. In this perspective, we identified acylating groups to serve the latter’s purpose. Next, we synthesized LDM_C-K_ reagents (**9a**–**9d**) with a range of leaving groups to establish the right combination of functional groups (Fig. 3a; for multistep synthesis of reagents, see Supplementary Figs. 1–5). We selected β-lactoglobulin A (BLGA, **8a**, PDB ID: 3BLG, Supplementary Fig. 39) with one free Cys and fifteen Lys residues as the model protein to validate the hypothesis (Fig. 3a). The NHS-ester derivative (**9a**) was highly reactive and resulted in heterogeneous labeling of proteins (Supplementary Figs. 38 and 40). Upon replacing the leaving group to bring down the reactivity, the tribromophenoxide derivative (**9b**) resulted in mono-labeled BLGA (**10b**, 9% conv., Supplementary Fig. 41). Interestingly, substituting one bromine with a morpholine amide (**9c**) led to improved conversions (**10c**, 30%, Supplementary Fig. 42) while retaining the homogeneity. Finally, we were delighted to note quantitative yields (**10d**, >99%, Supplementary Fig. 43) with tetrafluorophenoxide (**9d**) as the leaving group. With desired F_C_ and F_K_ in hand, we synthesized a set of LDM_C-K_ reagents for further evaluation (**9e**–**9h**, Fig. 3b and Supplementary Figs. 6–9).

### Investigation with peptides

**Fig. 4:**
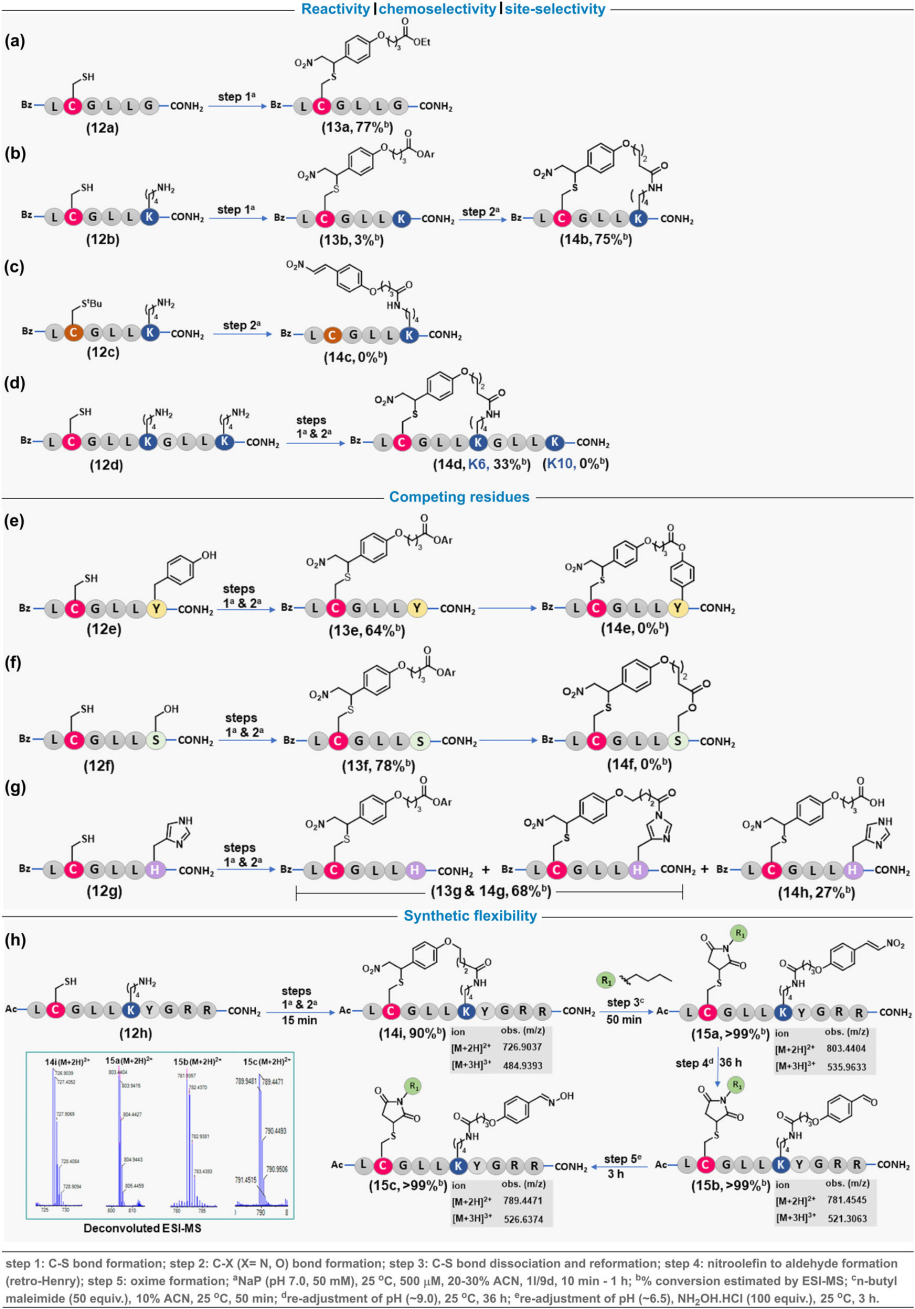
Peptide modification. **a** Control reagent **1l** with F_C_ and Cys-containing peptide **12a** results in efficient C-S bond formation. **b** LDM_C-K_ reagent **9d** mixed with peptide **12b** having Cys and Lys at *i* and *i* + *4* positions result in C-S bond followed by C-N bond formation rendering the cyclic peptide **14b** within 1 h. **c** LDM_C-K_ reagent **9d** with peptide **12c** having thiol protected Cys and Lys at *i* and *i* + *4* positions result in no conversion within 1 h. **d** LDM_C-K_ reagent **9d** with peptide **12d** having Cys at *i* and two Lys residues at *i* + *4*/*i* + *8* positions result in C-S bond formation followed by site-selective C-N bond formation at *i* + *4* position. Control experiments to examine competition with **e** Tyr, **f** Ser, and **g** His. **h** Establishing the complete LDM_C-K_ workflow. The selected MS for **14i**, **15a**, **15b**, and **15c** are given in the inset. Also see Supplementary Figs. 102–106 for detailed data, full XIC, and MS spectra.

At this point, we designed a set of experiments with peptides to validate the reactivity and selectivity attributes of LDM_C-K_ (Fig. 4 and Supplementary Table 4). The reaction time (1 h) was selected to keep conversions in a range where we can visualize the differences in efficiency. At first, we examined the reactivity of F_C_ containing control reagent (**1** **l**) with Cys in a peptide (BzLCGLLG, **12a**) devoid of Lys. Here, a rapid C-S bond formation led to the formation of the Michael adduct (**13a**, 77% conv., Fig. 4a and Supplementary Fig. 44). Next, we designed and synthesized a peptide BzLCGLLK (**12b**) with Cys (*i*) and Lys (*i* + *4*) in proximity. We treated this peptide with the LDM_C-K_ reagent (**9d**). Gratifyingly, a clear sequence of C-S bond formation followed by cyclization resulted in excellent conversions (**14b**, 75% conv., Fig. 4b and Supplementary Fig. 48) within an hour. The MS and MS-MS confirmed the selective modification of proximal Lys (*i* + *4*). To re-validate, we pre-installed a protecting group on Cys [BzLC(tBu)GLLK (**12c**)] and treated it with the same LDM_C-K_ reagent (**9d**). With all the other reaction parameters retained, no conversions were observed in this case (**14c**, 0% conv., Fig. 4c and Supplementary Fig. 49). Finally, we treated the peptide BzLCGLLKGLLK (**12d**) with **9d** to test for site-selectivity (**14d**, 33% conv., Fig. 4d and Supplementary Fig. 50). The MS and MS-MS confirmed the selective modification of proximal Lys (*i* + *4*) while the other Lys (*i* + *8*) remained unaffected. Next, we investigated the potential competition from selected residues from the amino acid pool examined earlier (Fig. 2b). For this purpose, we synthesized the single-site mutants for LCGLLK (**12b**), where K is replaced with Tyr (**12e**), Ser (**12f**), and His (**12g**). The linchpin formation could not render covalent bond formation with both Tyr and Ser (Figs. 4e and 4f). On the other hand, the His forms acyl imidazole that undergoes hydrolysis, rendering unmodified residue (Fig. 4g). These experiments confirmed that such competing residues would not leave traces after the C-S bond dissociation. Next, we established the efficiency of the LDM_C-K_ workflow with a similar peptide equipped with two additional arginine residues for enhanced solubility (Fig. 4h). The rapid LDM_C-K_ results in the cyclic peptide (**14i**) within 15 minutes. The subsequent C-S bond dissociation, thiol interception with maleimide, and retro-Henry reaction allow quantitative aldehyde generation (**15b**, >99%). Finally, the oxime formation proceeds in excellent conversions (>99%) to render the dual-labeled peptide **15c** (Fig. 4h).

### Precision engineering of native proteins–simultaneous regulation of reactivity, chemoselectivity, site-selectivity, and modularity

**Fig. 5:**
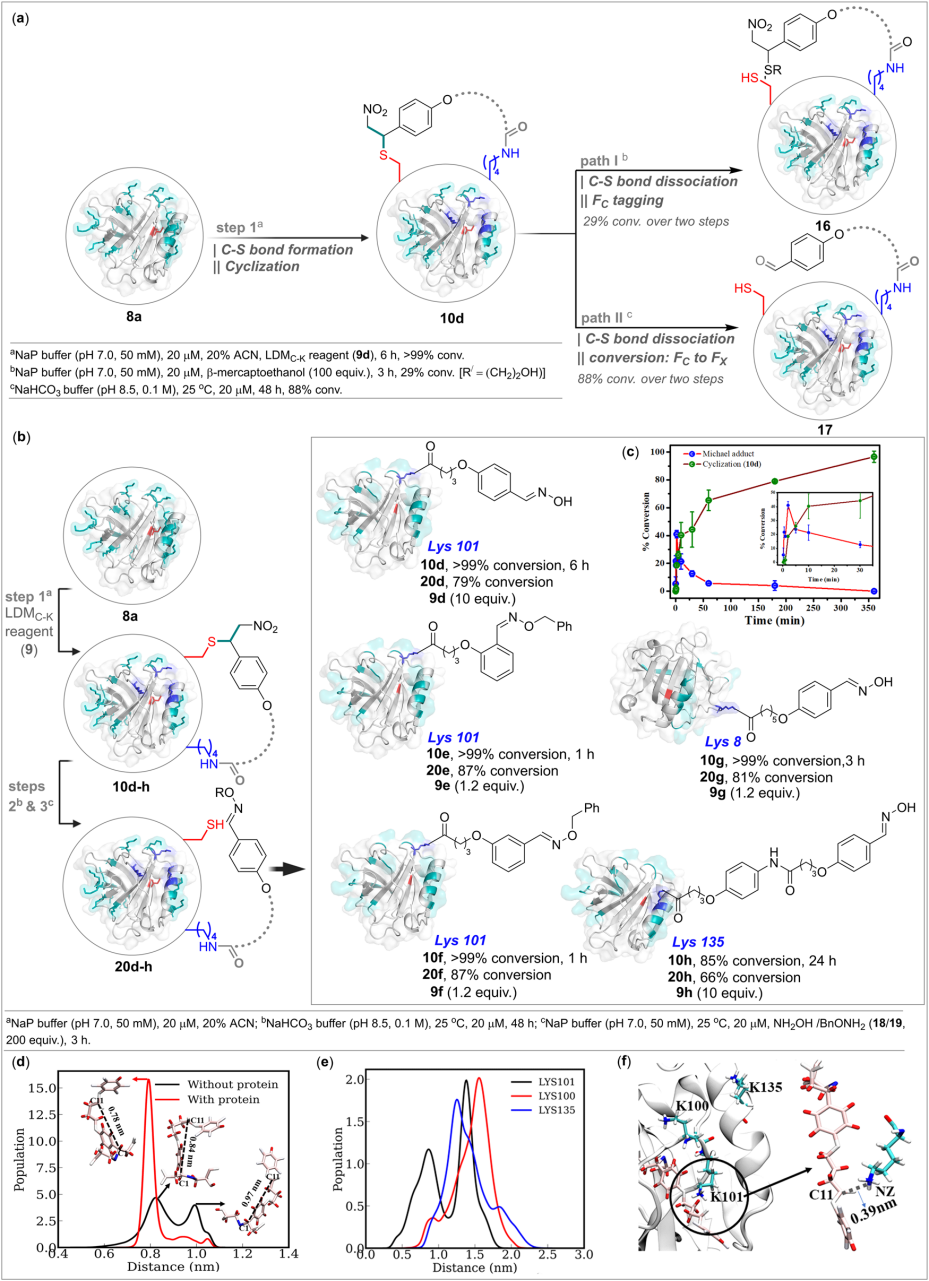
LDM_C-K_ technology. **a** Workflow for C-S bond formation, cyclization, C-S/C-C bond dissociation, and F_C_ to F_X_ transformation. **b** The chemoselective, site-selective, and modular single-site labeling of a native protein (β-lactoglobulin, BLGA, **8a**). **c** The plot of % conversion versus time highlights the progress of Michael addition and cyclization with BLGA in the presence of LDM_C-K_ reagent **9d**. Data are presented as mean values (±SD), *n* = 3 independent experiments. **d** Adaptable rigidity of LDM_C-K_ reagent: Probability distribution for the reagent (**9d**) and end-to-end distance between electrophilic C1 and C11 atoms in water with (red) or without (black) BLGA. The most probable conformations corresponding to peaks are shown. **e** Probability distribution of the distance between electrophilic C11 atom of the LDM_C-K_ reagent **9d** and nearby lysine residues (NZ atom) in BLGA. **f** Interaction of C11 in **9d** with the nearby lysine residues. Inset highlights the interaction of Lys101 with the C11 (**9d**).

After validating the hypothesis and a clear understanding of reactivity and selectivity parameters, we were all set to establish the capability of LDM_C-K_ with a native protein. At first, we re-validated the chemoselectivity of the nitroolefin-based control reagent (**1l**) with BLGA (**8a**). The proteolytic digestion of **1l**-labeled BLGA with α-chymotrypsin, peptide mapping (labeled VCQCL; residues 118–122; *m/z* 844.7 [M + H]^+^), and MS-MS confirmed the chemoselective labeling of C121 (Supplementary Fig. 52). In a control experiment, the proteins devoid of Cys did not result in any irreversible bioconjugation (Supplementary Table 5 and Supplementary Fig. 51). Now, we had to establish the efficiency of the C-S bond dissociation protocol (Fig. 5a). For this purpose, we selected the optimized example (Fig. 3) and synthesized mono-labeled BLGA (**10d**) with LDM_C-K_ reagent (**9d**) in >99% conversion (step 1, Fig. 5a). In a control experiment, the BLGA (**8a**) pre-treated with maleimide does not result in bioconjugation with **9d** (Supplementary Fig. 53). The non-reactivity of **9d** with proteins devoid of free Cys revalidates the case (Supplementary Table 6 and Supplementary Figs. 58–64). Also, the reduced nitroolefin derivative of LDM_C-K_ reagent **9d** (**11a**) leads to heterogeneous modification of BLGA, potentially through Cys-based thioester (Supplementary Fig. 54). Further, it was interesting to note that our findings with the small molecule model system (Fig. 2c) translated for the C-S bond dissociation in BLGA bioconjugate (**10d**). We trapped the retro-Michael reaction-led nitroolefin (path I, Fig. 5a and Supplementary Fig. 55) immediately by a thiol to avoid the reversible reaction. We were delighted to note the efficient translation of one-pot C-S and C-C bond dissociation to proteins for transforming F_C_ (nitroolefin) to F_X_ (aldehyde) under operationally simple conditions (path II, Fig. 5a and Supplementary Fig. 56). To enable unambiguous protein sequencing, we further treated the BLGA bioconjugate (**17**) with hydroxylamine (**18**). The subsequent mono-labeled product (**20d**) was digested with α-chymotrypsin enabling the identification of labeled KKY in peptide mapping (residues 100–102, *m/z* 643.3 [M + H]^+^). The MS-MS confirmed the site of modification as K101 (Supplementary Fig. 66). Besides multiple other Lys residues, the other proximal Lys (K100) does not undergo any modification highlighting the method’s capability in distinguishing closely placed targets.

After establishing the concept unambiguously, we were equipped with all the tools of LDM_C-K_ technology to test it further. We selected the LDM_C-K_ reagents (**9e** and **9f**) with ortho- and meta-disubstituted aromatic ring-based spacer in place of para-disubstituted aryl ring in **9d**. Interestingly, eight-fold less bioconjugation reagent **9e** was enough for six-fold improvement in reaction time to render mono-labeled BLGA within 1 h (Fig. 5b and Supplementary Fig. 67). Also, the LDM_C-K_ reagent (**9f**) turned out to be remarkable and delivered mono-labeled BLGA (**8a**) within an hour (Fig. 5b and Supplementary Fig. 68). We released the linchpin for these two bioconjugates by C-S/C-C bond dissociation and the subsequently formed aldehyde was captured with benzyloxyamine (**19**). These adducts were then subjected to protease degradation followed by peptide mapping. Both **20e** and **20f** resulted in labeled KKY (residues 100–102, *m/z* 733.3 [M + H]^+^) that upon subsequent MS-MS confirmed the site-of-modification as K101 (Supplementary Figs. 67 and 68). To gain further insights into the sequence of steps, we performed a time-dependent investigation of LDM_C-K_-enabled bioconjugation (Fig. 5c and Supplementary Fig. 65). Here, mixing BLGA and LDM_C-K_ reagent **9d**, quenching it with dilution after ten seconds, and subjecting it immediately to MS confirmed a rapid C-S bond formation with F_C_. At this point, no acylation is observed. Within the next twenty seconds, the increase of linchpin formation by thio-Michael addition (Cys-F_C_) is accompanied by cyclization (Lys-F_K_), while the latter becomes more prominent after 2 minutes. The Michael adduct disappears gradually and renders >99% cyclic adduct within 6 h.

With these detailed studies in the background, the next step was to establish modular capabilities. The initial noteworthy result in this perspective came with >99% conversion to bioconjugate **10g** within 3 h when we treated BLGA with the re-designed LDM_C-K_ reagent **9g** (Fig. 5b). Upon subsequent steps involving C-S/C-C bond dissociation followed by oxime formation, we subjected the mono-labeled product **20g** to protein sequencing. The proteolytic digestion and peptide mapping led to the identification of labeled KGL (residues 8–10, *m/z* 550.4 [M + H]^+^). The MS-MS confirmed the labeling of site K8 and the modularity of LDM_C-K_ technology (Supplementary Fig. 69). Interestingly, the closely placed K91 and K141 do not get labeled at all. We further challenged the method to label another unique Lys residue in the vicinity of Cys121. Gratifyingly, the LDM_C-K_ reagent **9h** rendered the chemoselective and site-selective modification of K135 (EKF, residues 134–136, *m/z* 806.4 [M + H]^+^). Again, the neighboring Lys residues (K138 and K141, Supplementary Fig. 70) do not compromise the bioconjugation selectivity. Overall, these results established that LDM_C-K_ can simultaneously regulate reactivity, chemoselectivity, site-selectivity, and modularity with excellent control.

#### Molecular dynamics simulation: impact of linchpin formation on the adaptable conformational rigidity of LDM_C-K_ reagent and downstream site-selection

We anticipated that the effectiveness and selectivity of the LDM_C-K_ reagents would be primarily dependent on their geometry and conformation. It is tempting to speculate that these parameters would regulate the effective distance between F_C_ and F_K_. Hence, the BLGA modification must happen if the distance between the linchpin (Cys121) and the target site (Lys) matches the spacer’s effective length. However, the dynamics of the protein and the reagent could make the linear correlation unreliable. Hence, we resorted to MD simulation to provide microscopic insights into the structure and dynamics of this complex molecular system and investigate our assumptions’ validity.

At first, we analyzed the conformational space of the LDM_C-K_ reagent (capped) in an aqueous medium in the absence and presence of the protein. Figure 5d shows the distance distribution between the atoms C1 and C11, which forms covalent bonds with the S atom of Cys121 and side-chain N atom of target Lys, respectively. Hence, it would be an appropriate parameter to capture the conformational space explored by the LDM_C-K_ reagent and check whether it is consistent with the linchpin-target distance in the protein. It shows the comparison of the distance distribution in both systems. In absence of protein (black line), there is a broad distribution with two predominant peaks around 0.85 nm and 1.0 nm. The representative structures of these conformations in water are highlighted (Fig. 5d). There is a minor peak above 1.0 nm, which is not analyzed in detail. This data clearly indicates that the LDM_C-K_ reagent can’t be treated as a rigid entity. Instead, it exists in multiple possible conformations in an aqueous medium that is characterized by a significant difference in its effective length. Interestingly, this distribution landscape changes drastically in the presence of the protein (BLGA), and there is a major peak at around 0.8 nm (red line). Although there are signatures of other conformations, their population is substantially low. The shift in the conformational ensemble state is induced by the local environment and interactions with the protein. Consequently, a subset of this population is stabilized preferentially. This attribute of adaptable rigidity of otherwise flexible LDM_C-K_ reagent enables it to adjust as per the surface landscape of the protein. These detailed investigations also confirm that the design principle of the bioconjugation reagent merely based on a static length of the extended configuration would not be successful in general.

With these insights, we turned our attention to the LDM_C-K_ reagent in the presence of protein. Being anchored to Cys121, this large and flexible molecule has considerable degrees of freedom to access and interact with different parts of the protein. But the key question is whether it can come into close contact with any of the target Lys residues, particularly the Lys101 residue, which gets selectively modified by LDM_C-K_ reagent **9d**. Further, we investigated the distance distribution between the S atom of the Cys121 and N atom of various Lys residues in the neighborhood (Supplementary Fig. 91). The peaks for both Lys101 and Lys135 appear in proximity around 1 nm, making them tough competitors to be modified selectively by the linchpin.

In order to investigate the propensity of the linchpin to approach various Lys residues in a selective manner, we looked into the probability distribution of the distance between the C11 atom and the N atoms of multiple nearby Lys residues (Lys100, Lys101, and Lys135, Fig. 5e). The comparison of these distance distributions clearly establishes that the C11 atom has the highest propensity to approach the Lys101 residue compared to others. A representative structure showing the proximal interaction is shown in Fig. 5f. Results obtained from all three independent trajectories are consistent with this trend (Supplementary Fig. 92). Thus, our simulation results validate the experimental findings and provide a mechanistic basis of action of the LDM_C-K_ reagent. It also highlights how the conformational flexibility of the reagent coupled with protein-induced rigidity could enable the site selection in protein bioconjugation.

### Late-stage modification and dual-probe installation

**Fig. 6:**
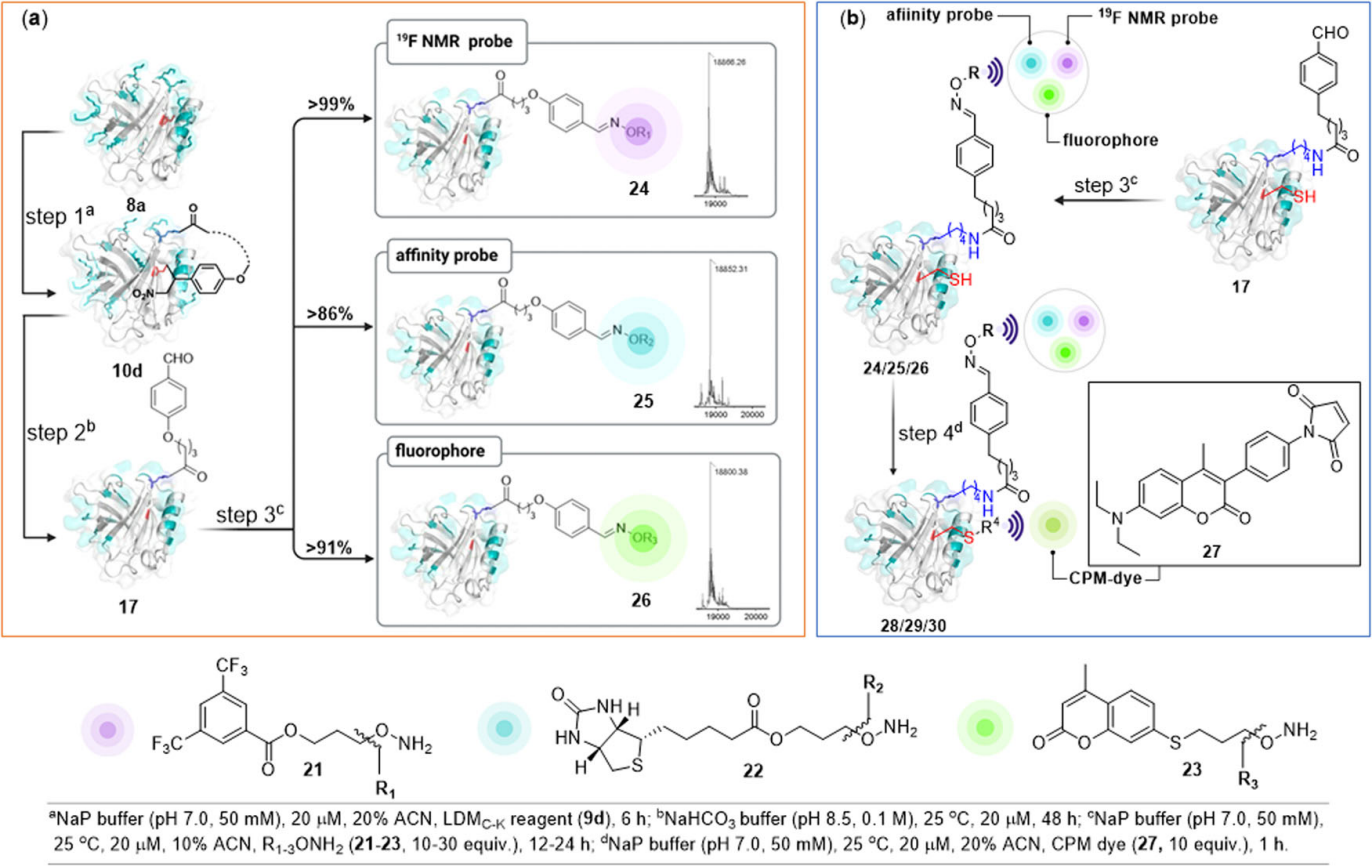
Late-stage installation of various tags on protein. **a** Single-site parallel installation of ^19^F NMR probe **21**, affinity probe **22**, and fluorophore **23**. **b** Precise second probe installation through the chemoselective installation of CPM dye (**27**) on protein bioconjugates (**24**–**26**) to render dual-probe conjugates (**28**–**30**).

At this point, we decided to validate the potential of re-engineered linchpin (F_X_) for the installation of probes. In this perspective, we treated BLGA with the LDM_C-K_ reagent (**9d**, Fig. 6a). After bioconjugation rendered cyclic protein (**10d**, >99% conv.), we subjected it to one-pot C-S/C-C bond dissociation rendering F_X_ (**17**). Finally, we distributed the precursor to three reaction vials and subjected them to the parallel installation of probes through oxime formation. Here, the treatment of hydroxylamine derivative (**21–23**) of the probes delivered installation of ^19^F NMR tag (**24**, >99% conv. over two steps), biotin-based affinity probe (**25**, 86% overall conv.), and fluorophore (**26**, 91% overall conv.). The remarkable overall efficiency of the protocol is noteworthy. The independent sequencing of the products by MS, proteolytic digestion, peptide mapping (labeled KKY, residues 100–102; *m/z* 941.9, *m/z* 927.9, and *m/z* 875.3 [M + H]^+^), and MS-MS confirms the site-of-conjugation (K101, Supplementary Figs. 71–73). Next, we selected single-site engineered BLGA bioconjugates (**24**–**26**) for dual-probe installation. In a user-friendly chemoselective protocol, the CPM-maleimide derivative rendered dual-probe conjugates (**28**–**30**, 71–78%, Supplementary Figs. 75–77) and established its utility for applications in this perspective.

### Challenges of serum albumins

At this stage, we decided to display the potential of LDM_C-K_ with serum albumins. These proteins present additional complexity levels due to the display of a large number of solvent-accessible lysine residues and heterogeneous post-translational modifications (Supplementary Figs. 78 and 81). Besides, the solvent accessibility of Cys is much lower in comparison to BLGA. Interestingly, LDM_C-K_ reagent **9d** resulted in single-site modification of bovine serum albumin (BSA **8j**, Supplementary Fig. 79). The proteolytic degradation, peptide mapping (labeled KVPQVSTPTLVEVSR, residues 413–427, *m/z* 645.69 [M + 3H]^3+^), and MS-MS confirmed the site-of-modification as K413 (Supplementary Fig. 79). Further, the single-site labeling of BSA at K431 with LDM_C-K_ reagent **9g** confirmed the modularity of the method (labeled SLGKVGTR, residues 428–435, *m/z* 570.82 [M + 2H]^2+^, Fig. S80). The level of precision becomes even more noteworthy in the backdrop of fifty-nine lysine residues in BSA, where more than twenty are in the proximity of cysteine. With these results in hand, we examined the potential of LDM_C-K_ with human serum albumin (HSA, **8k**). It contains fifty-seven lysine residues, with more than half of them in the proximity of cysteine. Gratifyingly, the LDM_C-K_ reagent **9d** delivered single-site modification of HSA (Supplementary Fig. 82). Further, sequencing confirmed the labeled peptide (LKCASLQK, residues 198–205, *m/z* 621.83 [M + 2H]^2+^) and the site-of-modification (K199, Supplementary Fig. 82). The observation of labeled LKCASLQK (residues 198–205) with LDM_C-K_ reagents **9e** (*m/z* 414.89 [M + 3H]^3+^) and **9f** (*m/z* 621.83 [M + 2H]^2+^) and subsequent MS-MS re-validated the results (Supplementary Figs. 83–84). The esterase-like activity of HSA remains unperturbed after bioconjugation (Supplementary Fig. 109).

### Protein-selectivity empowered by residue-pair selectivity coupled with a purification protocol

**Fig. 7:**
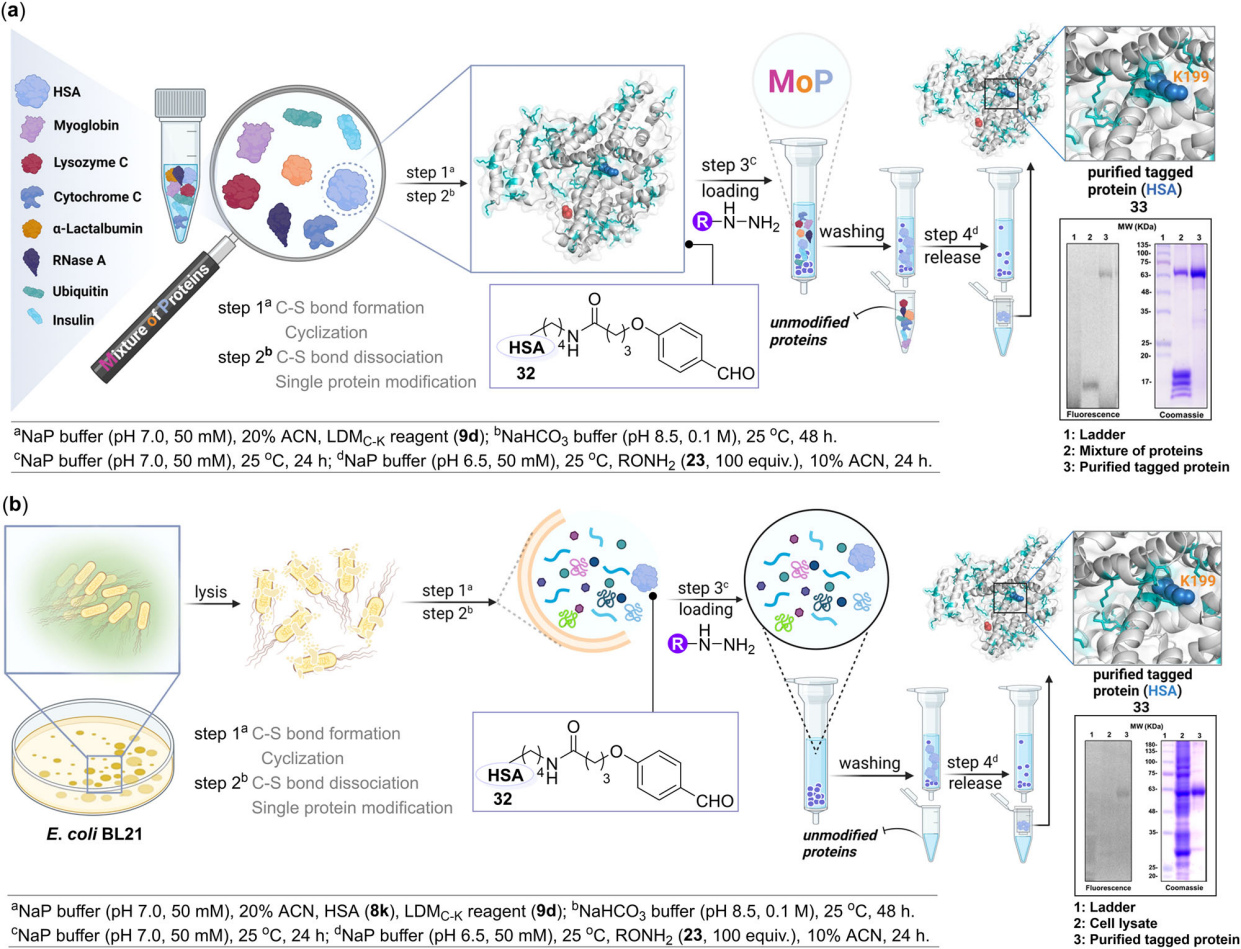
Protein-selectivity coupled with chemoselectivity and site-selectivity and the purification workflow. Single-site, single-protein labeling of human serum albumin in **a** mixture of proteins (MoP), and **b** cell lysate. Hydrazone formation captures and enriches the bioconjugate by ordered on-resin immobilization allowing recovery of unlabeled proteins. Next, the transoximization and centrifugal spin concentration render analytically pure single-site tagged protein bioconjugate.

We selected a *mixture of proteins* to establish whether the method can sustain the biomolecular crowding and deliver simultaneous regulation of protein selectivity along with chemoselectivity and site-selectivity (Fig. 7a). At first, a structurally and functionally diverse mixture of myoglobin, lysozyme C, cytochrome C, α-lactalbumin, RNase A, ubiquitin, insulin, and HSA was treated with LDM_C-K_ reagent (**9d**). The MS data confirmed the exclusive modification of HSA (**8k**, Supplementary Fig. 85). Next, we coupled the method with a purification protocol. In the process, we used acyl hydrazide functionalized beads to capture and enrich the HSA bioconjugate. After recovering the unreacted proteins, the immobilized HSA bioconjugate on-resin was released through transoximization using hydroxylamine derivative of coumarin (**33**). While the SDS-PAGE (see Supplementary Fig. 86) validated the purification protocol, the peptide mapping, and MS-MS (see Supplementary Fig. 86) of the isolated HSA bioconjugate confirmed the conjugation site (K199). It is noteworthy that the chemoselectivity and site-selectivity remained unaffected by the crowding of other proteins.

### Modification of a protein in cell lysate

Later, we challenged the performance of the LDM_C-K_ method in a cell lysate derived from *E. coli* BL21 cells (Fig. 7b). The cell lysate was spiked with HSA (**8k**) and treated with LDM_C-K_ reagent **9d**. We were delighted to note protein-specific modification of HSA. We utilized the purification protocol involving hydrazone-oxime chemistry to enrich the HSA bioconjugate from the cell lysate and installed a coumarin tag in the process. After confirming the purity by SDS-PAGE (Supplementary Fig. 87), the proteolytic digestion, peptide mapping, and MS-MS confirmed the conservation of chemoselectivity and site-selectivity (K199, Supplementary Fig. 87) in combination with protein selectivity. It further revalidates that LDM_C-K_ can negate the biomolecular crowding effect making it a suitable method for diverse applications.

### Antibody-drug conjugates

**Fig. 8:**
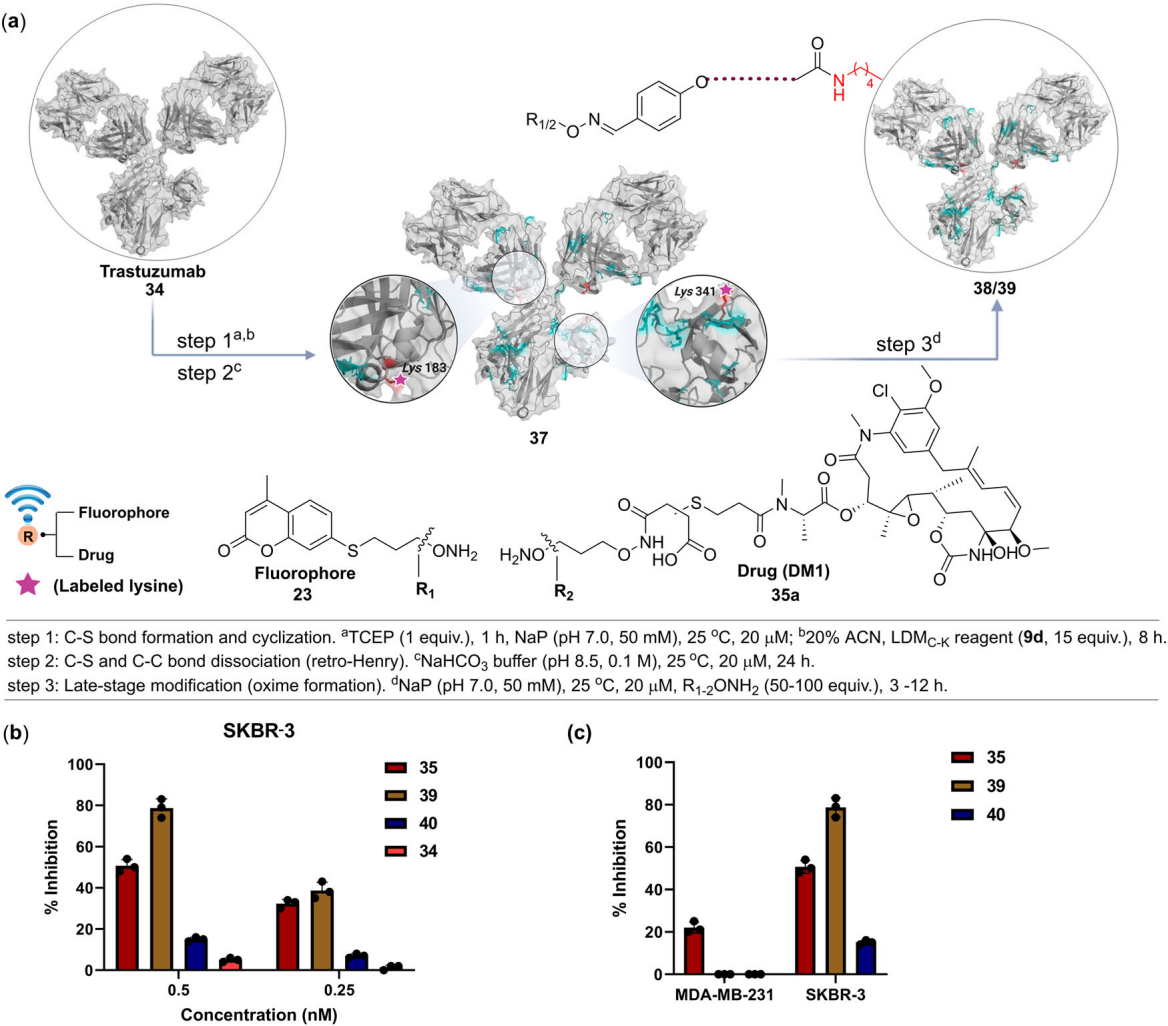
LDM_C-K_ for homogeneous antibody-fluorophore and drug conjugate (AFC and ADC). **a** LDM_C-K_ reagent **9d** renders homogeneous trastuzumab conjugates with specific modifications of K183 and K341. Subsequently, AFC (**38**) and ADC (**39**) were prepared by late-stage installation of hydroxylamine derivative of fluorophore (coumarin, **23**) and drug (emtansine, DM1, **35a**). **b** Inhibition of cell proliferation by LDM_C-K_-ADC (**39**) as compared to trastuzumab (**34**), DM1 (**35**), and Kadcyla (**40**) in SKBR-3 (HER-2 positive) cancer cell line. The percentage inhibition was calculated using untreated cells as control. **c** Inhibition of cell proliferation by DM1 (**35**), ADC (**39)**, and Kadcyla (**40**) at 0.5 nM concentration in HER-2 positive SKBR-3 as compared to HER-2 negative MDA-MB-231 cells. Data are presented as mean values (±SD), *n* = 3 biologically independent experiments.

The emergence of platforms for precision engineering of proteins has promised to meet protein-based therapeutics’ requirements. One of the biggest beneficiaries in this perspective has been the ADCs. These constructs provide a state-of-the-art platform for directed cancer chemotherapeutics. However, many efforts failed at the pre-clinical stages due to the lack of methods to access homogeneous ADCs. In this regard, the LDM platform offers promise. The ADC involves a monoclonal antibody conjugated to a drug molecule. While the antibody delivers the conjugate to tumor cells with overexpressed antigens, the drug is responsible for the low-dose cellular killing. The homogeneity of conjugation sites could ensure better control over the ADC attributes. Hence, we applied the LDM_C-K_ method to synthesize antibody conjugates (AFC and ADC, Fig. 8a). At the onset, we treated the mAb with TCEP to reduce the disulfide and introduce free cysteine as per the established protocols^40^. Subsequent treatment with the LDM_C-K_ reagent **9d** led to the formation of cyclic adduct **36** that upon subsequent C-S/C-C dissociation yields the aldehyde-antibody conjugate (AAC, **37**). Further, we sequenced the bioconjugate to establish the conjugation sites (K183, light chain, and K341, heavy chain, Supplementary Fig. 89) and aldehyde-antibody ratio (AAR 1.0, Supplementary Fig. 88). Next, the AAC (**37**) was treated with the hydroxylamine derivatives of coumarin (**23**) and emtansine (**35a**) to render AFC (**38**) and ADC (**39**), respectively. The SDS-PAGE revalidates the conjugation with both heavy and light chains (Supplementary Fig. 90). Next, the antiproliferative assay was performed to determine the efficacy of the ADC in the HER-2 overexpressing cell line SKBR-3. The results showed significant inhibition of cell growth at 0.25 nM (38%) and 0.5 nM doses (78%, Fig. 8b). Under the same conditions, Kadcyla (**40**) delivered 7 and 16% inhibition, respectively. Importantly, the LDM_C-K_ ADC (0.5 nM) did not affect the viability of the HER-2 negative MDA-MB-231 cell line, while emtansine (DM1, **35**) decreased its growth by 22% (Fig. 8c). Overall, the results establish the high efficacy and specificity of LDM_C-K_-ADC towards antiproliferation of HER-2 positive breast cancer cells.

## Discussion

The precision engineering of proteins creates a hinge to connect chemistry, biology, and medicine. Hence, it is not surprising that it has drawn attention from diverse segments of science, specifically the rapidly growing biologics for precision therapeutics. However, the inherent challenges kept the principles for regulating precision elusive. As a result, the technological demand for specifically engineered proteins remained unmet, thrusting the use of heterogeneous bioconjugates. The efforts of our group and a few others helped gain insight into principles for rendering chemoselectivity and site-selectivity. However, simultaneous regulation of multiple selectivity attributes beyond protein-defined reactivity order remains challenging.

This manuscript presents an LDM that establishes the principles for creating a cysteine-based linchpin and directs single-site modification of a proximal lysine residue (LDM_C-K_). The method renders simultaneous regulation of reactivity, chemoselectivity, site-selectivity, modularity, and protein selectivity within the spectrum of optimized reaction parameters. The selection of low-frequency cysteine was critical to directly modifying a single lysine of a single protein within a complex mixture of proteins. However, finding an appropriate handle to serve multiple attributes around C-S bond formation and chemically orthogonal bond dissociation under mild physiological conditions was non-trivial. Hence, the major credit for the success of LDM_C-K_ goes to the unique F_C_ or nitroolefin chemistry that enabled the cysteine-linchpin by rendering an array of synchronized bond formation and dissociation processes. Besides, its successful transformation to aldehyde under mild conditions enables the integration of orthogonal late-stage transformations. Importantly, it drives a highly efficient purification protocol to deliver ordered protein immobilization and analytically pure single-site probe-tagged bioconjugates. Notably, the seamless operation extends to a complex biomolecular mixture to deliver protein selectivity coupled with single-site precision. Another critical attribute of the LDM_C-K_ technology emerges from the adaptable rigidity of spacer that facilitates the placement of lysine selective group and irreversible bioconjugation. Notably, the overall protocol is user-friendly for non-experts. The platform proved highly efficient for synthesizing homogeneous dual-probe protein conjugates, antibody-fluorophore conjugate (AFC), and ADC. The LDM_C-K_-ADC exhibited excellent antiproliferative activity toward HER-2-positive breast cancer cells without negatively impacting the HER-2-negative cells. Further, the LDM technology offers principles that promise to bridge the gap between precision engineering of isolated proteins versus when they reside in a complex ecosystem.

## Methods

### General procedure of peptide labeling

The peptide stock solution was freshly prepared using acetonitrile with two drops of HPLC-grade methanol to enhance the solubility.

### Michael addition to the peptide

Peptide containing free cysteine **12a** (68 µg, 100 nmol) in acetonitrile (20 µl) from a freshly prepared stock solution was taken into a 1.5 ml HPLC vial containing phosphate buffer (140 µl, 50 mM, pH 7.0). To this solution, reagent **1** **l** (28 µg, 50 nmol) in acetonitrile (40 µl) was added and incubated at 25 °C. The overall concentration of the reaction was 500 µM. The progress of the Michael addition was monitored by LC-ESI-MS for up to 1 h.

### Acylation with the peptide

Peptide containing protected cysteine and free lysine **12c** (80 µg, 100 nmol) in acetonitrile (20 µl) from a freshly prepared stock solution was taken into a 1.5 ml HPLC vial containing phosphate buffer (140 µl, 50 mM, pH 7.0). To this solution, reagent **9d** (40 µg, 100 nmol) in acetonitrile (40 µl) was added and incubated at 25 °C. The overall concentration of the reaction was 500 µM. The progress of the reaction was monitored by LC-ESI-MS for up to 1 h.

### Peptide macrocyclization

Peptide containing free cysteine and lysine **12b** (75 µg, 100 nmol) in acetonitrile (20 µl) from a freshly prepared stock solution was taken into a 1.5 ml HPLC vial containing phosphate buffer (140 µl, 50 mM, pH 7.0). To this solution, reagent **9d** (40 µg, 100 nmol) in acetonitrile (40 µl) was added and incubated at 25 °C. The overall concentration of the reaction was 500 µM. The progress of the Michael addition followed by macrocyclization was monitored by LC-ESI-MS up to 1 h. This protocol was also used for peptides **12e**–**12g** for examining the competition from Tyr, Ser, and His. The peptide **12h** requires a few changes in the method (ESI) due to improved solubility in water.

### Site-selective peptide modification

Peptide containing one free cysteine and two lysine residues **12d** (116 µg, 100 nmol) in acetonitrile (20 µl) from a freshly prepared stock solution was taken into a 1.5 ml HPLC vial containing phosphate buffer (140 µl, 50 mM, pH 7.0). To this solution, reagent **9d** (40 µg, 100 nmol) in ACN (40 µl) was added and incubated at 25 °C. The overall concentration of the reaction was 500 µM. The progress of the reaction was monitored by LC-ESI-MS up to 1 h. The site-of-modification was confirmed by MS-MS.

### General procedure for site-selective modification of native proteins

Protein **8a** (2 nmol) in phosphate buffer (70–80 µl, 50 mM, pH 7.0) was taken in a 1.5 ml microcentrifuge tube. To this solution, LDM_C-K_ reagent (**9d**–**9h**, 2.4–20 nmol, each separately) in acetonitrile (20–30 µl) from a freshly prepared stock solution was added and incubated at 25 °C. The overall concentration of protein and LDM_C-K_ reagent was 20 µM and 24–200 µM, respectively. After 1–24 h, the reaction mixture was diluted with water (500 µl), followed by centrifugation for 1 min at 9600×*g* to precipitate the unreacted LDM_C-K_ reagent. The unreacted reagent and salts were removed using a centrifugal spin concentrator (0.5 mL, 10 kDa MWCO). The protein mixture was further washed with Grade I water (2 × 0.4 ml). The sample was analyzed by LC-ESI-MS or MALDI-ToF-MS. The sample was then buffer exchanged using NaHCO_3_ (100 µl, 0.1 M, pH 8.5) and incubated for 48 h. It resulted in the C-S bond dissociation, and a free aldehyde handle on the protein (**17**) was generated via the retro-Henry reaction. The sample was then buffer exchanged using phosphate buffer (100 µl, 50 mM, pH 7.0). To this solution, hydroxylamine hydrochloride **18** or *O*-benzylhydroxylamine hydrochloride **19** (2 µmol) in water (10 µl) from a freshly prepared stock solution was added for late-stage modification (oxime formation) and incubated for 3 h. The excess hydroxylamine and salts were removed by a centrifugal spin concentrator (0.5 ml, 10 kDa MWCO) and the sample was collected in an aqueous medium. The modification of protein was analyzed by LC-ESI-MS. The aqueous sample was concentrated by lyophilization before subjecting it to digestion, peptide mapping, and sequencing by MS-MS. [**Note**: The same protocol was followed for the single-site modification of BSA (**8j**) and HSA (**8k**)].

### General procedure for molecular dynamics simulations

The atomistic classical molecular dynamics (MD) simulation studies were performed to understand LDM chemistry. The structures were solvated in TIP3P water box, extending 12 Å from the solute in all three directions using the LEAP module in AMBER20. Appropriate numbers of Na^+^ and Cl^−^ counterions were added to neutralize the charges and to maintain 150 mM physiological salt concentration. AMBER ff14 force field was used to describe the interaction involving protein and Joung and Cheatham parameters for ions. The resulting solvated structures were subjected to minimization. Then MD was performed under constant pressure-constant temperature conditions (NPT) for 500 ps using a harmonic restraint on the solute with a force constant of 10 kcal/mol/Å^2^. This was followed by a production run of 1000 ns. Three independent trajectories were generated for the production runs. The LDM_C-K_ reagent and the protein were simulated separately in water using similar protocols as reference systems.

The MD simulations were carried out using the PMEMD module of AMBER20 package with imposed 3D periodic boundary conditions. TIP3P water model was used to solvate the systems. A time step of 2 fs was used to integrate the equation of motion. The temperatures were maintained for the simulations using Langevin dynamics, while pressure was kept constant at 1 atm using Berendsen weak coupling method with anisotropic pressure scaling. The particle mesh Ewald method was used to calculate long-range electrostatic calculations with a real space cut-off distance of 10 Å where the vdW and direct electrostatic interactions were truncated. All bond lengths involving hydrogen atoms were held fixed using the SHAKE algorithm. All analyses were done using CPPTRAJ module of AMBER20 tools. Snapshot generations were done using (Visual Molecular Dynamics).

### General procedure for single-site installation of tags

After the installation of free aldehyde handle on the protein (**17**) via retro-Henry process, various derivatives of hydroxylamine (**21**–**23**, 20–60 nmol) in H_2_O:ACN (1:1) from a freshly prepared stock solution were added for the late-stage modification (oxime formation) and incubated for 12–24 h. The excess hydroxylamine and salts were removed by a centrifugal spin concentrator (0.5 ml, 10 kDa MWCO), and the sample was collected in an aqueous medium. The modification of protein was analyzed by LC-ESI-MS. The aqueous sample was concentrated by lyophilization before subjecting it to digestion, peptide mapping, and sequencing by MS-MS.

### General procedure for dual-probe installation

Protein **8a** (2 nmol) in phosphate buffer (80 µl, 50 mM, pH 7.0) was taken in a 1.5 ml microcentrifuge tube. To this solution, LDM_C-K_ reagent (**9d**, 20 nmol) in acetonitrile (20 µl) from a freshly prepared stock solution was added and incubated at 25 °C. The overall concentration of protein and LDM_C-K_ reagent was 20 µM and 200 µM, respectively. After 6 h, the reaction mixture was diluted with water (500 µl), followed by centrifugation for 1 min at 9600×*g* to precipitate the unreacted LDM_C-K_ reagent. The unreacted reagent and salts were removed using a centrifugal spin concentrator (0.5 mL, 10 kDa MWCO). The protein mixture was further washed with Grade I water (2 × 0.4 ml). The sample was analyzed by LC-ESI-MS. The sample was then buffer exchanged using NaHCO_3_ (100 µl, 0.1 M, pH 8.5) and incubated for 48 h. The C-S bond dissociation and retro-Henry reaction render the free aldehyde handle on the protein (**17**). The sample was then buffer exchanged using phosphate buffer (100 µl, 50 mM, pH 7.0). To this solution, various derivatives of hydroxylamine (**21**–**23**, 20–60 nmol) in ACN (10 µl) from the freshly prepared stock solution were added. The incubation for 12–24 h rendered the late-stage modification through oxime formation. Later to this solution, the CPM dye (**27**, 20 nmol) in acetonitrile (20 µl) from freshly prepared stock solution was added and incubated for 1 h. The excess hydroxylamine derivative, CPM dye, and salts were removed by a centrifugal spin concentrator (0.5 ml, 10 kDa MWCO), and the sample was collected in an aqueous medium. The modification of protein was analyzed by LC-ESI-MS.

#### Protein-selectivity coupled chemoselective and site-selective protein modification, enrichment, and late-stage tagging in a representative mixture of eight proteins

Ubiquitin (17 µg, 2 nmol), cytochrome C (24 µg, 2 nmol), RNase A (27 µg, 2 nmol), insulin (23 µg, 4 nmol), α-lactalbumin (28 µg, 2 nmol), lysozyme C (28 µg, 2 nmol), myoglobin (34 µg, 2 nmol), and HSA (25 µg, 0.4 nmol) in phosphate buffer (80 µl, 50 mM, pH 7.0) were mixed in a 1.5 ml microcentrifuge tube. Here, the HSA concentration was kept five times lower than the other proteins to present a substantial challenge to the method. To this solution, LDM_C-K_ reagent **9d** (1.6 µg, 4 nmol) in ACN (20 µl) from a freshly prepared stock solution was added and incubated at 25 °C. After 3 h, the reaction mixture was diluted with grade I water (500 µl). The unreacted LDM_C-K_ reagent **9d** and salts were removed by centrifugal spin concentrator (0.5 ml, 3 kDa MWCO) and the protein mixture was collected in the NaHCO_3_ buffer (100 µl, 0.1 M, pH 8.5). Next, the incubation for 48 h generates a free aldehyde handle on protein (**32**) via the retro-Henry reaction. Subsequently, the reaction mixture was diluted with phosphate buffer (500 µl, 50 mM, pH 7.0). The buffer exchange was performed by centrifugal spin concentrator (0.5 ml, 3 kDa MWCO). Subsequently, this reaction mixture was utilized for the enrichment of HSA.

In a 5 ml fritted polypropylene chromatography column with end tip closures, hydrazide beads (400 µl, hydrazide resin loading: 16 µmol/ml) were taken. The beads were washed with phosphate buffer (0.1 M, pH 7.0, 5 × 1 ml) and re-suspended (phosphate buffer, 100 µl, 50 mM, pH 7.0). The reaction mixture containing modified HSA **32** (40 nmol) in phosphate buffer (500 µl, 50 mM, pH 7.0) was added to the beads. Next, the end-to-end rotation (29 × *g*, rotary mixer) was performed at 25 °C for 24 h. The supernatant was collected, and the beads were washed with KCl (0.5 M, 6 × 1 ml) and phosphate buffer (0.1 M, pH 7.0, 4 × 1 ml) to remove the unreacted and adsorbed proteins from the resin. The beads were further washed with Grade I water (6 × 1 ml) and re-suspended (phosphate buffer, 450 µl, 50 mM, pH 6.5). To release the labeled protein from its immobilized derivative, the *O*-hydroxylamine derivative of coumarin **23** (20 µM) in ACN:H_2_O (50 µl) was added. The subsequent end-to-end rotation at 25 °C for 24 h led to transoximization. The supernatant was collected while the salts and **23** were removed using the centrifugal spin concentrator (10 kDa MWCO). The purity of the labeled protein **33** was confirmed by in-gel fluorescence (please see the source data file), peptide mapping, and MS-MS. The protocol results in coumarin tagged labeled protein **33** with excellent purity.

#### Single-site, single-protein labeling in cell lysate and its enrichment

*E. coli* BL21 cell lysate (2 µg/1 µl) in phosphate buffer (75 µl, 50 mM, pH 7.0) spiked with HSA **8k** (25 µg, 0.4 nmol, in phosphate buffer, 5 µl, 50 mM, pH 7.0) were taken in a 1.5 ml microcentrifuge tube. To this solution, the LDM_C-K_ reagent **9d** (1.6 µg, 4 nmol) in ACN (20 µl) from a freshly prepared stock solution was added and incubated at 25 °C. After 3 h, the reaction mixture was diluted with Grade I water (500 µl). The unreacted LDM_C-K_ reagent **9d** and salts were removed by centrifugal spin concentrator (0.5 ml, 3 kDa MWCO). The reaction mixture was collected in NaHCO_3_ buffer (100 µl, 0.1 M, pH 8.5). The subsequent incubation for 48 h renders a free aldehyde handle on protein (**32**) via retro-Henry reaction. Next, the reaction mixture was diluted with phosphate buffer (500 µl, 50 mM, pH 7.0). The buffer exchange was performed by centrifugal spin concentrator (0.5 ml, 3 kDa MWCO). This reaction mixture was utilized for the enrichment of modified HSA in the subsequent step.

In a 5 ml fritted polypropylene chromatography column with end tip closures, hydrazide beads (400 µl, hydrazide resin loading: 16 µmol/ml) were taken. The beads were washed with phosphate buffer (0.1 M, pH 7.0, 5 × 1 ml) and re-suspended (phosphate buffer, 100 µl, 50 mM, pH 7.0). The cell lysate containing labeled and unlabeled HSA (combined batch: 2.6 mg, 40 nmol) in phosphate buffer (500 µl, 50 mM, pH 7.0) were added to the beads, followed by end-to-end rotation (29 × *g*, rotary mixer) at 25 °C for 24 h. The supernatant was collected, and the beads were washed with KCl (0.5 M, 6 × 1 ml) and phosphate buffer (0.1 M, pH 7.0, 4 × 1 ml) to remove the unreacted and adsorbed proteins from resin. The beads were further washed with Grade I water (6 × 1 ml) and re-suspended (phosphate buffer, 450 µl, 50 mM, pH 6.5). To release the labeled protein from its immobilized derivative, the *O*-hydroxylamine derivative of coumarin **23** (20 µM) in ACN:H_2_O (50 µl) was added. Next, the transoximization is facilitated by end-to-end rotation at 25 °C for 24 h. The supernatant was collected while the salts and **23** were removed using the centrifugal spin concentrator (10 kDa MWCO). The purity of the labeled protein **33** was confirmed by in-gel fluorescence (please see the source data file), peptide mapping, and MS-MS. The protocol results in coumarin tagged labeled protein **33** with excellent purity.

#### Site-selective modification of trastuzumab and synthesis of AFC

Trastuzumab **34** (300 µg, 2 nmol, Emcure, N7123B01) in phosphate buffer (80 µl, 50 mM, pH 7.0) was taken in a 1.5 ml microcentrifuge tube. TCEP (5 µg, 2 nmol) was added to this solution, and the reaction mixture was vortexed for 1 h. It was followed by the addition of reagent **9d** (12 µg, 30 nmol) in ACN (20 µl) from a freshly prepared stock solution and vortexed for 8 h at 25 °C. The overall concentration of the trastuzumab **34** and LDM_C-K_ reagent **9d** was 20 µM and 300 µM, respectively. After 8 h, the reaction mixture was diluted with water (500 µl), followed by centrifugation for 1 min at 9600 × *g* to precipitate the unreacted LDM_C-K_ reagent. The unreacted reagent and salts were removed using a centrifugal spin concentrator (0.5 mL, 10 kDa MWCO). The protein mixture was further washed with Grade I water (2 × 0.4 ml). The sample was analyzed by LC-ESI-MS. The sample was then buffer exchanged using NaHCO_3_ (100 µl, 0.1 M, pH 8.5) and incubated for 48 h. This step resulted in C-S bond dissociation to generate the free aldehyde handle on the protein (**37**) enabled by the retro-Henry reaction. This trastuzumab conjugate (**37**) can be stored after lyophilization for late-stage installation of desired probes through their hydroxylamine derivative. The labeled trastuzumab (**37**, 2 nmol) in phosphate buffer (80 µl, 50 mM, pH 7.0) was taken in a 1.5 ml microcentrifuge tube. To this solution, the hydroxylamine derivative of coumarin **23** (0.2 µmol) in Grade I water (10 µl) from a freshly prepared stock solution was added separately for the late-stage installation of probes. The reaction mixture was incubated for 3 h to yield AFC (**38**, please see the source data file). The excess hydroxylamine derivative and salts were removed by centrifugal spin concentrator (0.5 ml, 10 kDa MWCO).

#### Synthesis of ADC and antiproliferative assay

The hydroxylamine derivative of DM1 (**35a**, 2 µmol) in DMSO (20 µl) and 10% HCl (10 µl) from a freshly prepared stock solution was added to the antibody bioconjugate **37** in phosphate buffer (80 µl, 50 mM, pH 7.0). Subsequently, the reaction mixture was vortexed at 25 °C for 12 h to form the oxime derivative, i.e., ADC (**39**). The reaction mixture was frozen, lyophilized, followed by the addition of 100 µl of water. Under these conditions, the unreacted DM1 derivative **35a** is insoluble, enabling its precipitation and removal. The centrifugal spin concentration (10 kDa MWCO) and volume reduction to 250 µl ensured the complete removal of **35a**. The sample was lyophilized and stored for further studies.

SKBR-3 cells (10^4^, source: NCCS Pune) were seeded in a 96-well plate (tissue culture grade, flat bottom) in a final volume of 100 μl of MacCoy’s 5 A culture medium. After seeding for 24 h, the cells were treated with various concentrations (0.25–0.5 nM) of ADC (**39**), Kadcyla (T-DM1, **40**, Roche, N1037B18), DM1 (**35**), and trastuzumab (**34**) for the next 48 h and the total volume was kept 200 μl after addition of compounds. All the treatments were given in triplicate. The inhibition of cell proliferation was assessed using the MTT assay from Sigma Aldrich (Sigma Aldrich, Saint Louis, USA). Briefly, MTT reagent (100 μl, final concentration 0.5 mg/ml) was added after removing the medium, and the plates were incubated at 37 °C. After 1–1.5 h (depending upon the formation of crystals) of incubation, DMSO (100 μl) was added, and absorbance was taken on an ELISA plate reader (CYTATION 5, BioTeK) with a test wavelength of 570 nm and a reference wavelength of 630 nm. Relative growth inhibition rates for the untreated control were calculated and expressed as % inhibition of cell proliferation. In order to check the selectivity of ADC (**39**), we performed the MTT assay in HER-2 negative MDA-MB-231 cells (please see the source data file). MDA-MB-231 cells (source: ATCC, STR profiling authentication) were seeded in the DMEM medium, and the same protocol was followed as mentioned above.

#### Statistics and reproducibility

All the experiments were independently repeated at least three times. Data are presented as mean values ± SD (standard deviation) calculated using OriginPro 8.5 and GraphPad Prism 8.

### Reporting summary

Further information on research design is available in the Nature Research Reporting Summary linked to this article.

## Supplementary information

Supplementary Information

Reporting Summary

## Data Availability

All data supporting the findings of this study are available within the Article and its accompanying Supplementary Information file. The source data for Figs. 5c, 7a, b, 8b, c and Supplementary Figs. 65, 86a, 87b, 90, 98a, b, 98c, 108, 111, 112, is provided in the Source Data file. The raw data generated in this study have been deposited in the Figshare public repository 10.6084/m9.figshare.21219896. Source data are provided with this paper.

## Source data

Source Data
