# Supplementary material for: Traceless cysteine-linchpin enables precision engineering of lysine in native proteins
Source: Nat Commun. 2022 Oct 13;13:6038. doi: 10.1038/s41467-022-33772-1 (PMC9561114; doi:10.1038/s41467-022-33772-1)
Supplement: Supplementary file 1 — Supplementary Information [file 41467_2022_33772_MOESM1_ESM.pdf]

Traceless cysteine-linchpin enables precision engineering of lysine in native proteins

Neelesh C. Reddy,[a] Rajib Molla,[a] Pralhad Namdev Joshi,[a] Sajeev T. K.,[b] Ipsita Basu,[c] Jyotsna Kawadkar,[b] Neetu Kalra,[d] Ram Kumar Mishra,[b] Suman Chakrabarty,[c] Sanjeev Shukla,[b] Vishal Rai*[a]

[a]Department of Chemistry, and [b]Department of Biological Sciences, Indian Institute of Science Education and Research Bhopal, Bhopal Bypass Road, Bhauri, Bhopal, 462 066 M.P. India [c]Department of Chemical and Biological Sciences, S. N. Bose National Centre for Basic Sciences, Block-JD, Sector-III, Salt Lake, Kolkata-700 106 W.B. India. [d]School of Bioengineering, VIT, Bhopal.

vrai@iiserb.ac.in

Table of Contents

1. Supplementary Notes 1 ..... S4

2. Supplementary Methods S10

2.1 LDM_{C-K} reagent – selection of F_C component S10

2.1.1 Identification of F_C (Cys-selective electrophile) S10

2.1.2 Establishing chemoselectivity of F_C (nitroolefin) S10

2.1.3 Establishing C-S bond dissociation with F_C S10

2.2 Peptide labeling S11

2.2.1 Michael addition with the peptide S11

2.2.2 Acylation with the peptide S11

2.2.3 Macrocyclization with the peptide S11

2.2.4 Site-selectivity with the peptide S11

2.2.5 LDM_{C-K} workflow with peptide S11

2.3 Protein labeling S12

2.3.1 Reactivity and selectivity of nitroolefin (F_C) in protein bioconjugation S12

2.3.2 LDM_{C-K} reagent - selection of F_K component (acyl donor) S12

2.3.3 Kinetics of Michael addition and cyclization S12

2.3.4 Site-selective modification of native proteins S12

2.3.5 Molecular dynamics simulations S13

2.3.6 Single-site installation of tags S13

2.3.7 Dual-probe installation S14

2.4 Protein digestion S14

2.5 Single protein (HSA) labeling in the mixture of proteins and its enrichment S15

2.6 Modification of HSA in cell lysate and its enrichment S15

2.7 Antibody drug conjugate S16

2.7.1 Site-selective modification of trastuzumab S16

2.7.2 Tagging of trastuzumab S16
3. Synthesis and characterization S18
3.1 LDM_{C-K} reagent synthesis S18
3.2 Derivatives of O-hydroxyl amine S36
4. Reference table: characterization data for protein labeling S40
5. Supplementary Notes 2: small molecules and peptides S41
5.1 Small molecule characterization S41
Supplementary Table 1. Screening of Cys-selective electrophiles (F_C) for C-S bond formation S41
Supplementary Table 2. Chemoselectivity of nitroolefin (F_C) with cysteine. S48
Supplementary Table 3. C-S bond dissociation of benzylmercaptan-nitrolefin adduct (3l). S51
5.2 Selection of acyl-donor (F_K) S56
5.3 Peptide modification S58
Supplementary Table 4. Peptide screening S58
6. Supplementary Notes 3: protein labelling S68
6.1 Screening of proteins with nitroolefin S68
Supplementary Table 5. Screening of proteins with nitroolefin S68
6.1.1 C-S bond formation of nitroolefin with β-lactoglobulin S69
6.2 Acylation of lysine in BLG-A (Cys-Protected) S70
6.3 Acylation of Cysteine in BLG-A S71
6.4 Retro-Michael addition with protein S72
6.5 Retro-Henry reaction with protein S72
6.6 Screening of proteins with LDM_{C-K} reagent (9d) S73
Supplementary Table 6. Screening of proteins with LDM_{C-K} reagent 9d. S73
6.7 Kinetics of C-S bond formation and cyclization S75
Supplementary Table 7. Time-wise monitoring of C-S bond formation and cyclization with reagent 9d. S75
6.8 Single-site labeling of native proteins S76
6.9 Late-stage modification S86
6.10 Dual probe installation S91
6.11 Single-site modification of serum albumins (BSA and HSA) S92
6.12 Modification and enrichment of HSA from mixture of proteins S98
6.13 Modification and enrichment of HSA from cell lysate S101
7. Trastuzumab modification S102
8. Supplementary Notes 4 .... S105
8.1 Molecular dynamics simulations S105
8.2 Stability of the LDM_{C-K} reagent 9d S106

Supplementary Table 8: Stability of LDM_{C-K} reagent 9d. S106
8.3 pH screening S112
Supplementary Table 9. pH screening with β-lactoglobulin A and LDM_{C-K} reagent 9d. S112
8.4 Concentration screening S114
Supplementary Table 10. Concentration screening with β-lactoglobulin A and LDM_{C-K} reagent 9d. S114
8.5 ^{19}F NMR data of labeled β-lactoglobulin A S116
8.6 Fluorescence data of labeled β-lactoglobulin A S116
8.7 Circular dichroism spectra of proteins S117
8.8a Reaction of peptides (competing nucleophile) with LDM_{C-K} reagent 9d S118
8.8b C-S and C-C bond cleavage in peptides S122
8.8c Linchpin dissociation in cyclic protein (BLGA) S125
Supplementary Table 11: Stability of cyclic BLGA at different pH as a function of time. S125
8.9a Enrichment of protein from E. Coli BL21 cell-lysate (without HSA) using coumarin tag S128
8.9b Enzymatic assay of HSA (Human serum albumin) S129
8.10 Comparative analysis of light chain (LC) and heavy chain (HC) in native and modified trastuzumab S130
8.11 Experiments with CASLQK tagged Hs-Ubc9 S131
9. Protein sequence S135
10. Spectral data S137
Supplementary references S175

1. Supplementary Notes 1

The reagents, proteins, and enzymes were purchased from Sigma-Aldrich (Merck), Alfa Aesar, Novabiochem, Emcure, Roche, Labex, and Thermo Fisher Scientific. Fritted polypropylene chromatography columns were purchased from Bio-Rad. The reagent-grade organic solvents were used. Aqueous buffers were prepared freshly using Merck Millipore Grade I water (Resistivity > 5 MΩ cm, Conductivity < 0.2 μS/cm, TOC <30 ppb). Mettler Toledo (FE20) pH meter was used to adjust the final pH. The reaction mixture for the small molecules was stirred (Heidolph magnetic stirrer, 480-576 x g). Proteins were either vortexed (336 x g, 25-37 $^{\circ}$C) or incubated (25-37 $^{\circ}$C) in Thermo Fisher Scientific MaxQ 8000 incubator-shaker or Thermo Scientific Digital Heating Shaking Drybath. Amicon® Ultra-0.5 mL 3-kDa or 10-kDa 30-kDa MWCO centrifugal filters from Merck Millipore were used to remove small molecules from protein mixture, desalting, and buffer exchange. Organic solvents were removed by BUCHI rotavapor R-210/215, whereas aqueous samples were lyophilized by CHRiST ALPHA 2-4 LD plus lyophilizer. Circular Dichroism (CD) measurements were recorded on a JASCO J-815 CD spectropolarimeter equipped with a Peltier temperature controller. All the spectra were measured with a 50 nm/min scan speed, spectral bandwidth 1 nm, using a 10 mm path length cuvette at 25 $^{\circ}$C. UV-Vis spectra were recorded in Agilent Carry-100 UV-Vis Spectrophotometer connected with a Peltier temperature controller. Microplate reader measurements were performed on BioTek Cytation 5. Steady-state fluorescence spectra were recorded in HORIBA JOBIN YVON, FLUOROLOG 3-111. The quartz cuvette of 1 cm path length was used to record the fluorescence spectra.

Chromatography: Thin-layer chromatography (TLC) was performed on silica gel coated aluminium TLC plates (Merck, TLC Silica gel 60 F254). The compounds were visualized using a UV lamp (254 nm) and stains such as iodine, ninhydrin, 2,4-diphenylhydrazine. The flash column chromatography was carried out on CombiFlash Rf 200, CombiFlash NextGen 300+, and gravity columns using 230-400 or 100-200 mesh silica gel from Merck.

Nuclear magnetic resonance spectra: ^{1}H, ^{13}C, and ^{19}F NMR spectra were recorded on Bruker Advance III 400 and 500 MHz NMR spectrometer. ^{1}H NMR spectra were referenced to TMS (0 ppm), DMSO-d_6 (2.50 ppm), D_2O (4.79 ppm), and acetone-d_6 (2.05 ppm), whereas ^{13}C NMR spectra were referenced to $CDCl_3$ (77.16 ppm), DMSO-d_6 (39.52 ppm), and acetone-d_6 (29.84 ppm). ^{19}F NMR was referenced to trifluoroacetic acid (-75.70 ppm). The following abbreviations designate peak multiplicities: s, singlet; bs, broad singlet; d, doublet; t, triplet; q, quartet; m, multiplet; dd, doublet of doublets; tt, triplet of triplets. All the NMR spectra were recorded at 298 K.

Mass spectrometry: SCIEX X500B qToF platform paired with ExionLC AD UHPLC and Agilent 6130 mass spectrometer with Agilent 1200 series HPLC was used for the MS data of small molecules, peptides, and proteins. HRMS data for small molecules were recorded on Bruker Daltonics MicroTOF-Q-II with electrospray ionization (ESI). Matrix-assisted laser desorption/ionization time of flight mass spectrometry was performed with Bruker Daltonics UltrafleXtreme Software-Flex control version 3.4, using sinapic acid and α-cyano-4-hydroxycinnamic acid (HCCA) matrix. Data collection and analysis was performed using Open LAB CDS A.01, SCIEX OS 1.7, Bruker Daltonics ESI Compass 1.3, and BioPharmaView Flex 2.1. Peptide mass and fragment ion calculator (proteomicsToolkit, FragIonServlet) were used for peptide mapping and sequencing.

Acetonitrile and H_2O were buffered with 0.01% formic acid and used as the mobile phase. Method A was used to record the LC-ESI-MS data for proteins. The stability of LDM reagents in aqueous buffer is analyzed through LC-ESI-MS using Method B. All the Amino acid and peptide related data is analyzed through LC-ESI-MS using Method C.

Method A: Intact mass of proteins on Agilent 6130 Quadrupole paired with Agilent 1200 infinity series HPLC (Column: Agilent, Poroshell 300 SB-C18 5 µm 2.1 × 75 mm, flow rate 0.5 ml/min)

Time (min)	H_2O (%)	Acetonitrile (%)
0	90	10
1	90	10
8	40	60
9	90	10
10	90	10

Method B: Intact mass of small molecules on Agilent 6130 Quadrupole paired with agilent 1200 infinity series HPLC (Column: Agilent, Poroshell 120 EC-C18 2.7 µM 3.0 × 50 mm, flow rate 0.3 ml/min)

Time (min)	H_2O (%)	Acetonitrile (%)
0	75	25
4	65	35
5	65	35
5.5	10	90
8	10	90
12	10	90

Method C: Intact mass of peptides and amino acids on Agilent 6130 Quadrupole paired with Agilent 1200 infinity series HPLC (Column: Agilent, AdvanceBio Peptide 2.7 µm 4.6 × 150 mm, flow rate 0.5 ml/min)

Time (min)	H_2O (%)	Acetonitrile (%)
0	90	10
3	50	50
5	25	75
10	10	90
15	10	90
25	10	90

Determination of percentage conversion in the protein labeled experiments

ESI-MS: Conversion for protein labeled was calculated based on the relative peak intensity of native protein and labeled protein in the deconvoluted mass spectrum.

% Conversion = $I_{desired\ product}$ /$I_{all\ relevant\ species}$, where $I_{desired\ product}$ is the peak intensity of labeled protein, and $I_{all\ relevant\ species}$ is the sum of the peak intensities of native protein and labeled protein in the deconvoluted mass spectra.

MALDI-ToF-MS: Conversion for protein labeling was calculated based on the relative peak intensity of native protein and labeled protein in the mass spectrum.

For all the examples, we preferred ESI-MS data over MALDI-ToF-MS for the estimation of conversions due to deviations less than ±5%.

Method D: Peptide mapping and MS-MS of proteins on SCIEX X500B paired with ExionLC AD UHPLC [Column: Phenomenex, bioZen, Peptide XB-C18 (2.5 x 150 mm, 1.7 µm, 100 Å) flow rate 0.3 ml/minutes. Acetonitrile and H_2O were buffered with 0.1% formic acid and used as the mobile phase.]

Time (minutes)	H_2O (%)	Acetonitrile (%)
0	95	5
2	95	5
25	50	50
26	20	80
27	20	80
28	95	5
29	95	5
30	95	5

Method E: Intact antibody conjugate analysis on SCIEX X500B paired with ExionLC AD UHPLC [Column: Phenomenex, bioZen, Protein XB-C8 (4.6 x 150 mm, 1.8 µm, 300 Å) flow rate 0.5 ml/minutes. Acetonitrile and H_2O were buffered with 0.1% formic acid and used as the mobile phase.]

Time (minutes)	H_2O (%)	Acetonitrile (%)
0	75	25
5	75	25
7	55	45
9	10	90
11	10	90
13	75	25
15	75	25

Method F: Light and heavy chains analysis of antibody on SCIEX X500B paired with ExionLC AD UHPLC [Column: Phenomenex, bioZen, Protein XB-C8 (4.6 x 150 mm, 1.8 µm, 300 Å) flow rate 0.5 ml/minutes. Acetonitrile and H_2O were buffered with 0.1% formic acid and used as the mobile phase.]

Time (minutes)	H_2O (%)	Acetonitrile (%)
0	95	5
2	95	5
35	70	30

45	50	50
47	20	80
48	20	80
49	95	5

Method G: Peptide mapping and MS-MS of antibody on SCIEX X500B paired with ExionLC AD UHPLC [Column: Phenomenex, bioZen, Peptide XB-C18 (2.5 x 150 mm, 1.7 µm, 100 Å) flow rate 0.3 ml/minutes. Acetonitrile and H_2O were buffered with 0.1% formic acid and used as the mobile phase.]

Time (minutes)	H_2O (%)	Acetonitrile (%)
0	95	5
5	95	5
50	50	50
55	20	80
60	20	80
62	95	5
65	95	5

Method H: Intact mass of protein on SCIEX X500B paired with ExonLC AD UPLC [Column: Phenomenex, bioZen, Peptide XB-C8 (4.6 x 150 mm, 1.8 µm, 300 Å) flow rate 0.6 ml/minutes. Acetonitrile and H_2O were buffered with 0.1% formic acid and used as the mobile phase.]

Time (minutes)	H_2O (%)	Acetonitrile (%)
0	80	20
1	80	20
5	20	80
7	20	80
9	80	20
10	80	20

Method I: Intact mass of peptide on SCIEX X500B paired with ExionLC AD UHPLC [Column: Phenomenex, bioZen, Peptide PS-C18 (2.1 x 150 mm, 3 µm, 100 Å) flow rate 0.5 ml/minutes. Acetonitrile and H_2O were buffered with 0.1% formic acid and used as the mobile phase.]

Time (minutes)	H_2O (%)	Acetonitrile (%)
0	90	10

3	90	10
5	50	50
10	50	50
15	25	75
20	10	90
22	90	10
25	90	5

Method J: MS-MS of small peptide on SCIEX X500B paired with ExionLC AD UHPLC [Column: Phenomenex, bioZen, Peptide PS-C18 (2.1 x 150 mm, 3 µm, 100 Å) flow rate 0.5 ml/minutes. Acetonitrile and H_2O were buffered with 0.1% formic acid and used as the mobile phase.]

Time (minutes)	H_2O (%)	Acetonitrile (%)
0	90	10
3	90	10
5	50	50
10	50	50
12	10	90
15	90	10

MS parameters (variables) for intact antibody, light, and heavy chains, and peptides

Parameter set A: Intact antibody

Curtain gas (psi):	40
Ion source gas 1 (psi):	60
Ion source gas 2 (psi):	60
Temperature (^oC):	500
Declustering potential (V):	225
Time bins to sum:	80

Parameter set B: HC and LC

Curtain gas (psi):	35
Ion source gas 1 (psi):	45
Ion source gas 2 (psi):	40
Temperature (^oC):	600
Declustering potential (V):	120
Time bins to sum:	20

Parameter set C: Peptide mapping and MS-MS

Curtain gas (psi):	30
Ion source gas 1 (psi):	45
Ion source gas 2 (psi):	40
Temperature (^{o}C):	550
Declustering potential (V):	80
Time bins to sum:	4

Parameter set D: Intact mass of protein

Curtain gas (psi):	35
Ion source gas 1 (psi):	45
Ion source gas 2 (psi):	40
Temperature (^{o}C):	400
Declustering potential (V):	120
Time bins to sum:	20

Parameter set E: Intact Peptide MS

Curtain gas (psi):	30
Ion source gas 1 (psi):	45
Ion source gas 2 (psi):	40
Temperature (^{o}C):	550
Declustering potential (V):	80
Time bins to sum:	4

Parameter set F: small Peptide MS-MS

Curtain gas (psi):	30
Ion source gas 1 (psi):	45
Ion source gas 2 (psi):	40
Temperature (^{o}C):	550
Declustering potential (V):	80
Time bins to sum:	4

SPPS (Solid Phase Peptide Synthesis): All the Fmoc amino acids were derivatized to amide with rink amide resin using SPPS with 95% purity. Similarly, the synthesis of all the peptide were done using the same resin i.e. rink amide.

The peptides were designed to be conformationally flexible to avoid biases towards a specific secondary structure. It was important that they present an extreme case to the LDM_{C-K} reagents so that we can anticipate the potential challenges with protein-based substrates. Besides, the selection ensured that C-S and C-N bond formation steps were well deconvoluted. This attribute was critical to establish the sequential order of these reactions. Further, we anticipated that even if the selectivity with such peptides is moderate, the methods would be appropriate for proteins as they display additional conformational constraints.

Cleavage of peptide from resin: The peptide was cleaved from the resin by mixing it with TFA/H_2O/TIPS (95:2.5:2.5, 4 ml) for 2 h. Subsequently, TFA was removed by evaporation on rotatory evaporator. The crude mixture was triturated (diethyl ether) and purified by preparative HPLC. [**Hint**: To cleave all cysteine (after cleavage it is free) containing peptide from resin TFA/H_2O/TIPS/DTT/Phenol (90:2.5:2.5:2.5:2.5, 4 ml) for 2 h was used.]

Purification of peptides: The purification was done using reverse-phase HPLC using Agilent column ZORBAX 300SB-C18 (5 µm, 9.4*250 mm)

Purification method: Mobile phase A: H_2O; mobile phase B: ACN

Time	Mobile phase A (%)	Mobile phase B (%)	Flow rate (ml/min)
0	85.0	15.0	4.0
25	5.0	95.0	-
30	5.0	95.0	-

MD simulations: The structure for β-lactoglobulin was obtained from the Protein Data Bank (PDB ID: 3BLG). The Cys121 residue was modified by the $LDM_{C\text{-}K}$ reagent (**9d**) manually using Pymol.[1,2] The force field parameters for the modified Cys121 (linchpin) were generated using the Antechamber[3] utility of AmberTools 20 software suite. The generated parameters were consistent with the GAFF2[4] atom types and AM1-BCC partial charges.

2. Supplementary Methods

2.1 $LDM_{C\text{-}K}$ reagent – selection of F_C component

2.1.1 Identification of F_C (Cys-selective electrophile)

The electrophile for conjugate addition (**1a-1l**, 5 µmol each in DMSO-d^6 (300 µl) was taken separately in 5 ml glass vial having 150 µl of D_2O. To this solution, reagent **2** (0.6 mg, 5 µmol) in DMSO-d^6 (50 µl) from a freshly prepared stock solution was added and stirred at 25 °C. The overall concentration of the reaction was 10 mM. The progress of the reaction was monitored by ^1H NMR within 10 min. (Related: Fig. 2a, Supplementary Fig. 15-26, Supplementary Table1)

2.1.2 Establishing chemoselectivity of F_C (nitroolefin)

All the twenty Fmoc-amino acids (**4a-4t**, 100 nmol each) in DMF: acetonitrile (3:7, total vol. 40 µl) from a freshly prepared stock solution were taken into 1.5 ml HPLC vial containing phosphate buffer (140 µl, 50 mM, pH 7.0). To this solution, the reagent **1l** (28 µg, 100 nmol) in acetonitrile (20 µl) was added and incubated at 25 °C. The overall concentration of the reaction was 500 µM. The progress of the reaction was monitored by ESI-MS within 10 min. (Related: Fig. 2b, Supplementary Figs. 27-28, Supplementary Table 2)

2.1.3 Establishing C-S bond dissociation with F_C

The reagent **3l** (40 µg, 100 nmol) in acetonitrile (60 µl) from a freshly prepared stock solution was taken into a 1.5 ml microcentrifuge tube containing phosphate buffer (140 µl, 50 mM, pH 7.0). To this solution, a selected base (**6a**/**6b**/**6c**/**6d**/**6e/6f**, 100 nmol) was added followed by vortexing at 25 °C. In parallel, another reaction was performed using $NaHCO_3$ buffer (140 µl, 0.1 M, pH 8.5) in the absence of base while keeping other parameters constant. The overall

concentration of the reaction was 500 µM. The progress of all the reactions were monitored by ESI-MS for 12-24 h. (Related: Fig. 2c, Supplementary Figs. 29-37, Supplementary Table 3)

2.2 Peptide labeling

The peptide stock solution was freshly prepared using acetonitrile with two drops of HPLC grade methanol to enhance the solubility (2.2.1-2.2.4).

2.2.1 Michael addition with the peptide

Peptide containing free cysteine **12a** (68 µg, 100 nmol) in acetonitrile (20 µl) from a freshly prepared stock solution was taken into a 1.5 ml HPLC vial containing phosphate buffer (140 µl, 50 mM, pH 7.0). To this solution, reagent **1l** (28 µg, 50 nmol) in acetonitrile (40 µl) was added and incubated at 25 °C. The overall concentration of the reaction was 500 µM. The progress of the Michael addition was monitored by LC-ESI-MS up to 1 h.

2.2.2 Acylation with the peptide

Peptide containing protected cysteine and free lysine **12c** (80 µg, 100 nmol) in acetonitrile (20 µl) from a freshly prepared stock solution was taken into a 1.5 ml HPLC vial containing phosphate buffer (140 µl, 50 mM, pH 7.0). To this solution, reagent **9d** (40 µg, 100 nmol) in acetonitrile (40 µl) was added and incubated at 25 °C. The overall concentration of the reaction was 500 µM. The progress of the reaction was monitored by LC-ESI-MS up to 1 h.

2.2.3 Macrocyclization with the peptide

Peptide containing free cysteine and lysine **12S1-S3** and **12b** (75 µg, 100 nmol) in acetonitrile (20 µl) from a freshly prepared stock solution were taken separately into a 1.5 ml HPLC vial containing phosphate buffer (140 µl, 50 mM, pH 7.0). To this solution, reagent **9d** (40 µg, 100 nmol) in acetonitrile (40 µl) was added and incubated at 25 °C. The overall concentration of the reaction was 500 µM. The progress of the Michael addition followed by macrocyclization was monitored by LC-ESI-MS up to 1 h. This protocol was also used for peptides **12e-12g** for examining the competition from Tyr, Ser, and His.

2.2.4 Site-selectivity with the peptide

Peptide containing one free cysteine and two lysine residues **12d** (116 µg, 100 nmol) in acetonitrile (20 µl) from a freshly prepared stock solution was taken into a 1.5 ml HPLC vial containing phosphate buffer (140 µl, 50 mM, pH 7.0). To this solution, reagent **9d** (40 µg, 100 nmol) in ACN (40 µl) was added and incubated at 25 °C. The overall concentration of the reaction was 500 µM. The progress of the reaction was monitored by LC-ESI-MS up to 1 h. The site-of-modification was confirmed by MS-MS.

2.2.5 LDM$_{C\text{-}K}$ workflow with peptide

Peptide containing free cysteine and lysine **12h** (122 µg, 100 nmol) in phosphate buffer (160 µl, 50 mM, pH 7.0) from a freshly prepared stock solution was taken separately into a 1.5 ml HPLC vial. To this solution, reagent **9d** (40 µg, 100 nmol) in acetonitrile (40 µl) was added and incubated at 25 °C for 15 minutes. The overall concentration of the reaction was 500 µM. Next, n-butyl maleimide (**S34**, 382 µg, 50 equiv.) solution in acetonitrile (10 µl) was added to the reaction mixture (**14i**) and incubated at 25 °C for 50 min. to form **15a**. The pH of the reaction mixture was readjusted to ~9.0 and incubated further for retro-Henry reaction (**15b**) at 25 °C

for 36 h. The pH was readjusted back to ~7.0 and $NH_2OH.HCl$ (690 µg, 200 equiv., 10 µl) was added from a freshly prepared stock solution. The reaction mixture was incubated at 37 °C for 3 h to render oxime **15c**. The progress of all the reactions was monitored by ESI-MS (XIC).

2.3 Protein labeling

2.3.1 Reactivity and selectivity of nitroolefin (F_C) in protein bioconjugation

Protein **8a** (2 nmol) in phosphate buffer (80 µl, 50 mM, pH 7.0) was taken in a 1.5 ml microcentrifuge tube. To this solution, control reagent **1l** (20 nmol) in acetonitrile (20 µl) from a freshly prepared stock solution was added and incubated at 25 °C. The overall concentration of protein and control reagent was 20 µM and 200 µM, respectively. After 30 min, the reaction mixture was diluted with water (500 µl). The unreacted reagent and salts were removed using a centrifugal spin concentrator (0.5 mL, 10 kDa MWCO). The protein mixture was further washed with Grade I water (2 x 0.4 ml). The sample was analyzed by LC-ESI-MS or MALDI-ToF-MS. The aqueous sample was concentrated by lyophilization before subjecting it to digestion, peptide mapping, and sequencing by MS-MS.

2.3.2 LDM_{C-K} reagent - selection of F_K component (acyl donor)

Protein **8a** (2 nmol) in phosphate buffer (80 µl, 50 mM, pH 7.0) was taken in a 1.5 ml microcentrifuge tube. To this solution, LDM_{C-K} reagent (**9d-9h**, 20 nmol, each separately) in acetonitrile (20 µl) from a freshly prepared stock solution was added and incubated at 25 °C. The overall concentration of protein and LDM_{C-K} reagent was 20 µM and 200 µM, respectively. After 6 h, the reaction mixture was diluted with water (500 µl), followed by centrifugation for 1 min at 9600 x g to settle down the unreacted LDM_{C-K} reagent. The unreacted reagent and salts were removed using a centrifugal spin concentrator (0.5 mL, 10 kDa MWCO). The protein mixture was further washed with Grade I water (2 x 0.4 ml). The sample was analyzed by LC-ESI-MS.

2.3.3 Kinetics of Michael addition and cyclization

Protein **8a** (2 nmol) in phosphate buffer (80 µl, 50 mM, pH 7.0) was taken in a 1.5 ml microcentrifuge tube. To this solution, LDM_{C-K} reagent (**9d**, 20 nmol) in acetonitrile (20 µl) from a freshly prepared stock solution was added and incubated at 25 °C. The overall concentration of protein and LDM_{C-K} reagent was 20 µM and 200 µM, respectively. The progress of the transformation was ceased at the given time points by diluting the reaction mixture from 100 µl to 1000 µl with Grade I water. A control experiment confirmed that the reaction showed no noticeable progress for thirty minutes after dilution. The sample was immediately subjected to mass analysis at regular intervals (10 sec to 6 h).

2.3.4 Site-selective modification of native proteins

Protein **8a** (2 nmol) in phosphate buffer (70-80 µl, 50 mM, pH 7.0) was taken in a 1.5 ml microcentrifuge tube. To this solution, LDM_{C-K} reagent (**9d-9h**, 2.4-20 nmol, each separately) in acetonitrile (20-30 µl) from a freshly prepared stock solution was added and incubated at 25 °C. The overall concentration of protein and LDM_{C-K} reagent was 20 µM and 24-200 µM, respectively. After 1-24 h, the reaction mixture was diluted with water (500 µl), followed by centrifugation for 1 min at 9600 x g to precipitate the unreacted LDM_{C-K} reagent. The unreacted reagent and salts were removed using a centrifugal spin concentrator (0.5 mL, 10 kDa MWCO). The protein mixture was further washed with Grade I water (2×0.4 ml). The sample was

analyzed by LC-ESI-MS or MALDI-ToF-MS. The sample was then buffer exchanged using $NaHCO_3$ (100 µl, 0.1 M, pH 8.5) and incubated for 48 h. It resulted in the C-S bond dissociation, and a free *aldehyde handle* on the protein (**17**) was generated via the retro-Henry reaction. The sample was then buffer exchanged using phosphate buffer (100 µl, 50 mM, pH 7.0). To this solution, hydroxylamine hydrochloride **18** or *O*-benzylhydroxylamine hydrochloride **19** (2 µmol) in water (10 µl) from a freshly prepared stock solution was added for late-stage modification (oxime formation) and incubated for 3 h. The excess of hydroxylamine and salts were removed by centrifugal spin concentrator (0.5 ml, 10 kDa MWCO) and the sample was collected in an aqueous medium. The modification of protein was analyzed by LC-ESI-MS. The aqueous sample was concentrated by lyophilization before subjecting it to digestion, peptide mapping, and sequencing by MS-MS. [**Note**: The same protocol was followed for the single-site modification of Bovine Serum Albumin (BSA, **8j**) and Human Serum Albumin (HSA, **8k**)]

2.3.5 Molecular dynamics simulations

The atomistic classical molecular dynamics (MD) simulation studies were performed the LDM chemistry. The structures were solvated in TIP3P[5] water box, extending 12 Å from the solute in all three directions using the LEAP module in AMBER20.[6] Appropriate numbers of Na^+ and Cl^- counterions were added to neutralize the charges and to maintain 150 mM physiological salt concentration. AMBER ff14[7] force field was used to describe the interaction involving protein and Joung and Cheatham parameters[8] for ions. Resulting solvated structures were subjected to minimization. Then MD was performed under constant pressure-constant temperature conditions (NPT) for 500 ps using a harmonic restraint on the solute with a force constant of 10 kcal/mol/Å2. This was followed by production run of 1000 ns. Three independent trajectories were generated for the production runs. The linchpin molecule and the protein were simulated separately in water using the similar protocols as reference systems.

The MD simulations were carried out using the PMEMD module of AMBER20 package with imposed 3D periodic boundary conditions.[9] TIP3P water model was used to solvate the systems. A time step of 2 fs was used to integrate the equation of motion. The temperatures were maintained for the simulations using Langevin dynamics,[10] while pressure was kept constant at 1 atm using Berendsen weak coupling method with anisotropic pressure scaling.[11] The particle mesh Ewald method[12] was used to calculate long-range electrostatic calculations with a real space cut-off distance of 10 Å where the vdW and direct electrostatic interactions were truncated. All bond lengths involving hydrogen atoms were held fixed using the SHAKE algorithm.[13] All analyses were done using CPPTRAJ module of AMBER20 tools. Snapshot generations were done using (Visual Molecular Dynamics).[14]

2.3.6 Single-site installation of tags

After the installation of free *aldehyde handle* on the protein (**17**) via retro-Henry process, various derivatives of hydroxylamine **(21-23)** (20-60 nmol) in H_2O:ACN (1:1) from a freshly prepared stock solution was added for the late-stage modification (oxime formation) and incubated for 12-24 h. The excess hydroxylamine and salts were removed by centrifugal spin concentrator (0.5 ml, 10 kDa MWCO), and the sample was collected in an aqueous medium. The modification of protein was analyzed by LC-ESI-MS. The aqueous sample was concentrated by lyophilization before subjecting it to digestion, peptide mapping, and sequencing by MS-MS.

2.3.7 Dual-probe installation

Protein **8a** (2 nmol) in phosphate buffer (80 µl, 50 mM, pH 7.0) was taken in a 1.5 ml microcentrifuge tube. To this solution, $LDM_{C\text{-}K}$ reagent (**9d**, 20 nmol) in acetonitrile (20 µl) from a freshly prepared stock solution was added and incubated at 25 °C. The overall concentration of protein and $LDM_{C\text{-}K}$ reagent was 20 µM and 200 µM, respectively. After 6 h, the reaction mixture was diluted with water (500 µl), followed by centrifugation for 1 min at 9600 x g to precipitate the unreacted $LDM_{C\text{-}K}$ reagent. The unreacted reagent and salts were removed using a centrifugal spin concentrator (0.5 mL, 10 kDa MWCO). The protein mixture was further washed with Grade I water (2×0.4 ml). The sample was analyzed by LC-ESI-MS. The sample was then buffer exchanged using $NaHCO_3$ (100 µl, 0.1 M, pH 8.5) and incubated for 48 h. The C-S bond dissociation and retro-Henry reaction render the free *aldehyde handle* on the protein (**17**). The sample was then buffer exchanged using phosphate buffer (100 µl, 50 mM, pH 7.0). To this solution, various derivatives of hydroxylamine (**21-23**) (20-60 nmol) in ACN (10 µl) from the freshly prepared stock solution were added. The incubation for 12-24 h rendered the late-stage modification through oxime formation. Later to this solution, the CPM dye (**27**, 20 nmol) in acetonitrile (20 µl) from freshly prepared stock solution was added and incubated for 1 h. The excess hydroxylamine derivative, CPM dye, and salts were removed by centrifugal spin concentrator (0.5 ml, 10 kDa MWCO), and the sample was collected in an aqueous medium. The modification of protein was analyzed by LC-ESI-MS.

2.4 Protein digestion

All the solutions were prepared freshly before use in reactions.[15]

Step 1. **Denaturation:** Protein (0.13 mg, 7.3 nmol) in grade I water (10 µl) with urea (6 M) was taken in a 1.5 ml microcentrifuge tube and incubated for 30 minutes.

Step 2. **Disulfide reduction:** To cleave the disulfide bonds, a reducing agent (1 µl, 0.2 M DTT in Grade I water) was added to the solution, and the sample was incubated for 1 h at 37 °C.

Step 3. **Sulfhydryl alkylation:** To block the free sulfhydryl groups, alkylating agent (5 µl, 0.2 M iodoacetamide in Grade I water) was added to the solution and incubated (in the dark) for 1 h at ambient temperature.

Step 4. **Quenching excess alkylating reagent:** To quench the unreacted iodoacetamide, a reducing agent (DTT, 5 µl) was added again to the mixture and the sample was incubated at 25 °C for 1 h. The dilution of the reaction mixture with Grade I water reduced the urea concentration to 0.6 M.

Step 5. **Desalting:** The unreacted reagent and salts were removed using centrifugal spin concentrator (0.5 ml, 10 kDa MWCO). This step helps in avoiding the signal suppression during subsequent analysis by ESI-MS after step 6.

Step 6. **Enzymatic digestion:** The desalted sample was diluted to 100 µl with Grade I water. To this mixture, 5 µl of α-chymotrypsin or trypsin solution [5 µg, based on ratio of chymotrypsin or trypsin/protein (1:20); α-chymotrypsin or trypsin in Grade I water] was added. After incubation at 37 °C for 18 h, trifluoroacetic acid (0.5%) was used to adjust the pH of digested solution to <6 (verified by pH paper). Subsequently, the sample was subjected to peptide mapping by MS and sequencing by MS-MS.

2.5 Single protein (HSA) labeling in the mixture of proteins and its enrichment

Bioconjugation in a representative mixture of eight proteins – Ubiquitin (17 µg, 2 nmol), cytochrome C (24 µg, 2 nmol), RNase A (27 µg, 2 nmol), insulin (23 µg, 4 nmol), α-lactalbumin (28 µg, 2 nmol), lysozyme C (28 µg, 2 nmol), myoglobin (34 µg, 2 nmol), and HSA (25 µg, 0.4 nmol) in phosphate buffer (80 µl, 50 mM, pH 7.0) were mixed in a 1.5 ml microcentrifuge tube. Here, the HSA concentration was kept five times lower than the other proteins to present a substantial challenge to the method. To this solution, $LDM_{C\text{-}K}$ reagent **9d** (1.6 µg, 4 nmol) in ACN (20 µl) from a freshly prepared stock solution was added and incubated at 25 °C. After 3 h, the reaction mixture was diluted with grade I water (500 µl). The unreacted $LDM_{C\text{-}K}$ reagent **9d** and salts were removed by centrifugal spin concentrator (0.5 ml, 3 kDa MWCO) and the protein mixture was collected in the $NaHCO_3$ buffer (100 µl, 0.1 M, pH 8.5). Next, the incubation for 48 h generates free *aldehyde handle* on protein (**32**) via the retro-Henry reaction. After 48 h, the reaction mixture was diluted with phosphate buffer (500 µl, 50 mM, pH 7.0). The buffer exchange was performed by centrifugal spin concentrator (0.5 ml, 3 kDa MWCO). Subsequently, this reaction mixture was utilized for the enrichment of HSA.

Enrichment protocol: In a 5 ml fritted polypropylene chromatography column with end tip closures, hydrazide beads (400 µl, hydrazide resin loading: 16 µmol/ml) were taken. The beads were washed with phosphate buffer (0.1 M, pH 7.0, 5 x 1 ml) and re-suspended (phosphate buffer, 100 µl, 50 mM, pH 7.0). The reaction mixture containing modified HSA **32** (40 nmol) in phosphate buffer (500 µl, 50 mM, pH 7.0) was added to the beads. Next, the end-to-end rotation (29 x g, rotary mixer) was performed at 25 °C for 24 h. The supernatant was collected, and the beads were washed with KCl (0.5 M, 6 x 1 ml) and phosphate buffer (0.1 M, pH 7.0, 4 x 1 ml) to remove the unreacted and adsorbed proteins from the resin. The beads were further washed with Grade I water (6 x 1 ml) and re-suspended (phosphate buffer, 450 µl, 50 mM, pH 6.5). To release the labeled protein from its immobilized derivative, the *O*-hydroxylamine derivative of coumarin **23** (20 µM) in ACN:H_2O (50 µl) was added. The subsequent end-to-end rotation at 25 °C for 24 h led to transoximization. The supernatant was collected while the salts and **23** were removed using the centrifugal spin concentrator (10 kDa MWCO). The purity of the labeled protein **33** was confirmed by in-gel fluorescence, peptide mapping, and MS-MS. The protocol results in coumarin tagged labeled protein **33** with excellent purity.

HSA activity: We have recorded the esterase activity of the labeled and unlabeled protein using *p*-nitrophenyl acetate (*p*NPA) as the substrate.[16] It produces *p*-nitrophenolate as a product after the hydrolysis of *p*NPA in the presence of the protein, and *p*-nitrophenolate absorbs at 400 nm. We monitored the change in absorbance at 400 nm over 150 minutes, providing us with the progression of the hydrolysis of *p*NPA in the presence of the protein. The protein and *p*NPA concentrations were 5 mM and 200 mM, respectively, for all the experiments. Data were acquired in a Carry 100 UV-Vis spectrophotometer at 25 °C.

2.6 Modification of HSA in cell lysate and its enrichment

E. coli BL21 cell lysate (2 µg/1 µl) in phosphate buffer (75 µl, 50 mM, pH 7.0) spiked with HSA **8k** (25 µg, 0.4 nmol, in phosphate buffer, 5 µl, 50 mM, pH 7.0) were taken in a 1.5 ml microcentrifuge tube. To this solution, the $LDM_{C\text{-}K}$ reagent **9d** (1.6 µg, 4 nmol) in ACN (20 µl) from a freshly prepared stock solution was added and incubated at 25 °C. After 3 h, the reaction mixture was diluted with Grade I water (500 µl). The unreacted $LDM_{C\text{-}K}$ reagent **9d** and salts

were removed by centrifugal spin concentrator (0.5 ml, 3 kDa MWCO). The reaction mixture was collected in $NaHCO_3$ buffer (100 µl, 0.1 M, pH 8.5). The subsequent incubation for 48 h renders free *aldehyde handle* on protein (**32**) via retro-Henry reaction. Next, the reaction mixture was diluted with phosphate buffer (500 µl, 50 mM, pH 7.0). The buffer exchange was performed by centrifugal spin concentrator (0.5 ml, 3 kDa MWCO). This reaction mixture was utilized for the enrichment of modified HSA in the subsequent step.

Enrichment protocol: In a 5 ml fritted polypropylene chromatography column with end tip closures, hydrazide beads (400 µl, hydrazide resin loading: 16 µmol/ml) were taken. The beads were washed with phosphate buffer (0.1 M, pH 7.0, 5 x 1 ml) and re-suspended (phosphate buffer, 100 µl, 50 mM, pH 7.0). The cell lysate containing labeled and unlabeled HSA (combined batch: 2.6 mg, 40 nmol) in phosphate buffer (500 µl, 50 mM, pH 7.0) were added to the beads, followed by end-to-end rotation (39 x g, rotary mixer) at 25 °C for 24 h. The supernatant was collected, and the beads were washed with KCl (0.5 M, 6 x 1 ml) and phosphate buffer (0.1 M, pH 7.0, 4 x 1 ml) to remove the unreacted and adsorbed proteins from resin. The beads were further washed with Grade I water (6 x 1 ml) and re-suspended (phosphate buffer, 450 µl, 50 mM, pH 6.5). To release the labeled protein from its immobilized derivative, the *O*-hydroxylamine derivative of coumarin **23** (20 µM) in ACN:H_2O (50 µl) was added. Next, the transoximization is facilitated by end-to-end rotation at 25 °C for 24 h. The supernatant was collected while the salts and **23** were removed using the centrifugal spin concentrator (10 kDa MWCO). The purity of the labeled protein **33** was confirmed by in-gel fluorescence, peptide mapping, and MS-MS. The protocol results in coumarin tagged labeled protein **33** with excellent purity.

2.7 Antibody drug conjugate

2.7.1 Site-selective modification of trastuzumab

Trastuzumab **34** (300 µg, 2 nmol) in phosphate buffer (80 µl, 50 mM, pH 7.0) was taken in a 1.5 ml microcentrifuge tube. TCEP (5 µg, 2 nmol) was added to this solution, and the reaction mixture was vortexed for 1 h. It was followed by the addition of reagent **9d** (12 µg, 30 nmol) in ACN (20 µl) from a freshly prepared stock solution and vortexed for 8 h at 25 °C. The overall concentration of the trastuzumab **34** and $LDM_{C\text{-}K}$ reagent **9d** was 20 µM and 300 µM, respectively. After 8 h, the reaction mixture was diluted with water (500 µl), followed by centrifugation for 1 min at 9600 x g to precipitate the unreacted $LDM_{C\text{-}K}$ reagent. The unreacted reagent and salts were removed using a centrifugal spin concentrator (0.5 mL, 10 kDa MWCO). The protein mixture was further washed with Grade I water (2×0.4 ml). The sample was analyzed by ESI-MS. The sample was then buffer exchanged using $NaHCO_3$ (100 µl, 0.1 M, pH 8.5) and incubated for 24 h. This step resulted in C-S bond dissociation to generate the free *aldehyde handle* on the protein (**37)** enabled by the retro-Henry reaction. This trastuzumab conjugate (**37**) can be stored after lyophilization for late-stage installation of desired probes through their hydroxylamine derivative.

2.7.2 Tagging of trastuzumab

The labelled trastuzumab (**37**, 2 nmol) in phosphate buffer (80 µl, 50 mM, pH 7.0) was taken in a 1.5 ml microcentrifuge tube. To this solution, the hydroxylamine derivative of coumarin **23** (2 µmol) in Grade I water (10 µl) from a freshly prepared stock solution were added

separately for the late-stage installation of probes. The reaction mixture was incubated for 3 h to yield antibody-fluorophore conjugate (AFC, **38**). The excess hydroxylamine derivative and salts were removed by centrifugal spin concentrator (0.5 ml, 10 kDa MWCO).

Antibody-drug conjugates (ADC): The hydroxylamine derivative of DM1 **35a** (2 µmol) in DMSO (20 µl) and 10% HCl (10 µl) from a freshly prepared stock solution was added to the antibody bioconjugate **37** in phosphate buffer (80 µl, 50 mM, pH 7.0). Subsequently, the reaction mixture was vortexed at 25 °C for 12 h to form the oxime derivative i.e. antibody-drug conjugate (ADC, **39**). The reaction mixture was frozen, lyophilized, followed by the addition of 100 µl of water. Under these conditions, the unreacted DM1 derivative **35a** is insoluble, enabling its precipitation and removal. The centrifugal spin concentration (10 kDa MWCO) and volume reduction to 250 µl ensured the complete removal of **35a**. The sample was lyophilized and stored for further studies.

Antiproliferative assay: SKBR-3 cells (10^4) were seeded in a 96-well plate (tissue culture grade, flat bottom) in a final volume of 100 µl of MacCoy's 5A culture medium. After seeding for 24 h, the cells were treated with various concentrations (0.25-0.5 nM) of ADC (**39**), Kadcyla (T-DM1, **40**), DM1 (**35**), and Trastuzumab (**34**) for the next 48 h and the total volume was kept 200 µl after addition of compounds. All the treatments were given in triplicate. The inhibition of cell proliferation was assessed using the MTT assay from Sigma Aldrich (Sigma Aldrich, Saint Louis, USA). Briefly, MTT reagent (100 µl, final concentration 0.5 mg/ml) was added after removing the medium, and the plates were incubated at 37 ºC. After 1-1.5 h (depending upon the formation of crystals) of incubation, DMSO (100 µl) was added, and absorbance was taken on an ELISA plate reader (CYTATION 5, BioTeK) with a test wavelength of 570 nm and a reference wavelength of 630 nm. Relative growth inhibition rates for the untreated control were calculated and expressed as % inhibition of cell proliferation. In order to check the selectivity of ADC (**39**), we performed the MTT assay in HER-2 negative MDA-MB-231 cells. MDA-MB-231 cells were seeded in the DMEM medium, and the same protocol was followed as mentioned above.

3. Synthesis and characterization

3.1 LDM$_{C\text{-}K}$ reagent synthesis

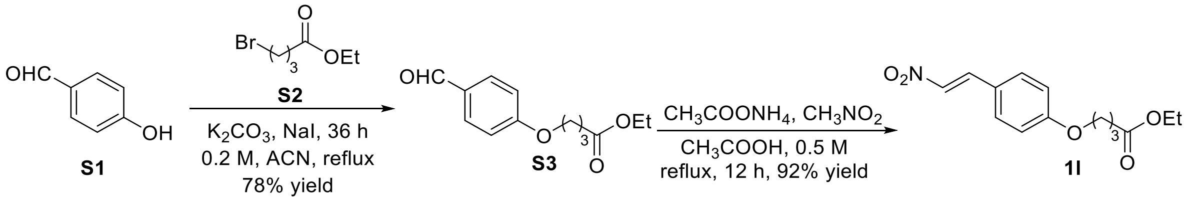

Supplementary Fig. 1. Synthesis of ethyl (E)-4-(4-(2-nitrovinyl)phenoxy)butanoate **1l**.

Synthesis of ethyl 4-(4-formylphenoxy)butanoate S3

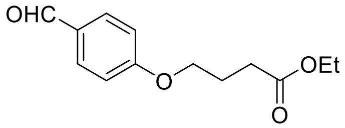

In a 50 ml round bottom flask, *p*-hydroxybenzaldehyde **S1** (611 mg, 5 mmol), ethyl 4-bromobutyrate **S2** (859 µl, 6 mmol), K_2CO_3 (1.037 g, 7.5 mmol) and NaI (374 mg, 2.5 mmol) were dissolved in ACN (25 ml). The reaction mixture was stirred at reflux condition for 36 h followed by filtration to remove potassium carbonate. The filtrate was concentrated to remove ACN and the residue was purified using silica gel flash column chromatography ethyl acetate:n-hexane (3:97) to give **S3** (969 mg, 82% yield, colourless liquid). TLC (ethyl acetate:*n*-hexane, 10:90 v/v): R_f = 0.59; ^{1}H NMR (500 MHz, $CDCl_3$): δ 9.88 (s, 1H), 7.81 (d, J = 8.8 Hz, 2H), 6.98 (d, J = 8.8 Hz, 2H), 4.15 (q, J = 7.2 Hz, 2H), 4.10 (t, J = 6.2 Hz, 2H), 2.51 (t, J = 7.2 Hz, 2H), 2.14 (m, 2H), 1.25 (t, J = 7.1 Hz, 3H); ^{13}C NMR (101 MHz, $CDCl_3$): δ 190.9, 173.1, 164.0, 132.1, 130.1, 114.8, 67.2, 60.6, 30.7, 24.5, 14.3; HRMS (ESI): $[M+H]^+$ calcd. for $C_{13}H_{17}O_4$, 237.1127; found, 237.1121.

Synthesis of ethyl (E)-4-(4-(2-nitrovinyl)phenoxy)butanoate (1l)

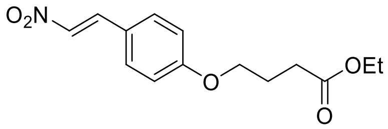

In a 100 ml round bottom flask, ethyl 4-(4-formylphenoxy)butanoate **S3** (2.36 g, 10 mmol), ammonium acetate (2.31 g, 30 mmol), nitromethane (2.7 ml, 50 mmol) and acetic acid (25 ml) were refluxed for 12 h. The reaction mixture turns into a red-brown solution while heating. The progress of the reaction was followed by TLC and 2,4-DNP was utilized to check the consumption of starting material i.e., aldehyde. After the completion of reaction, reaction mixture was allowed to cool and poured onto the ice to get a yellow colour solid, which was filtered using Büchner funnel. The crude reaction mixture was purified by silica gel flash column chromatography using ethyl acetate:n-hexane (2:98) to afford ethyl (E)-4-(4-(2-nitrovinyl)phenoxy)butanoate **1l** (2.51 g, 90% yield, yellow solid). TLC (ethyl acetate:*n*-hexane, 10:90 v/v): R_f = 0.89; ^{1}H NMR (500 MHz, $CDCl_3$): δ 7.96 (d, J = 13.6 Hz, 1H), 7.53 – 7.46 (m, 3H), 6.93 (d, J = 8.7 Hz, 2H), 4.15 (q, J = 7.1 Hz, 2H), 4.07 (t, J = 6.2 Hz, 2H), 2.52 (t, J = 7.2 Hz, 2H), 2.14 (m, 2H), 1.26 (t, J = 7.1 Hz, 3H); ^{13}C NMR (126 MHz, $CDCl_3$): δ

173.4, 162.6, 139.4, 135.5, 131.6, 123.0, 115.8, 67.5, 60.9, 31.0, 24.8, 14.6; HRMS (ESI): $[M+Na]^+$ calcd. for $C_{14}H_{17}NNaO_5$, 302.1107; found, 302.0999.

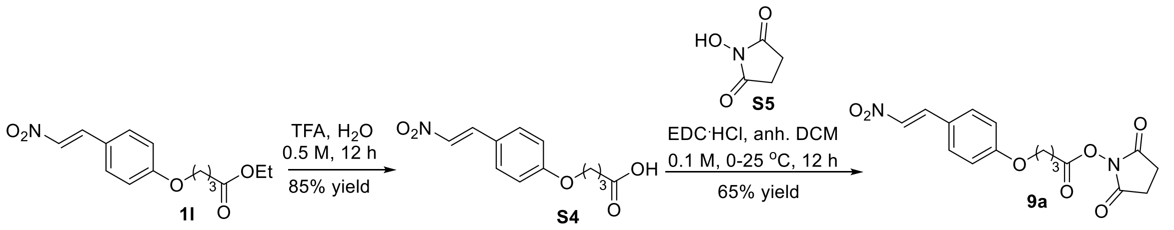

Supplementary Fig. 2. 2,5-dioxopyrrolidin-1-yl (E)-4-(4-(2-nitrovinyl)phenoxy)butanoate **9a**.

Synthesis of (E)-4-(4-(2-nitrovinyl)phenoxy)butanoic acid (S4)

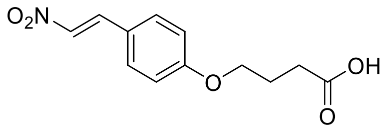

In a 10 ml round bottom flask, ethyl (E)-4-(4-(2-nitrovinyl)phenoxy)butanoate **1l** (279 mg, 1 mmol) and trifluoroacetic acid (307 µl, 4 mmol) were dissolved in water (5 ml). The reaction mixture was stirred at 100 °C for 12 h. The reaction mixture is allowed to cool and resulting into green yellow solid, which was filter using Büchner funnel. The progress of the reaction was followed by TLC. The purification of crude reaction mixture was performed by silica gel flash column chromatography using ethyl acetate:n-hexane (30:70) to isolate gave (E)-4-(4-(2-nitrovinyl)phenoxy)butanoic acid **S4** (240 mg, 95% yield, yellow solid). TLC (ethyl acetate:*n*-hexane, 30:70 v/v): R_f = 0.30; ^1H NMR (500 MHz, $CDCl_3$): δ 7.97 (d, J = 13.6 Hz, 1H), 7.54 – 7.46 (m, 3H), 6.94 (d, J = 8.6 Hz, 2H), 4.09 (t, J = 6.1 Hz, 2H), 2.60 (t, J = 7.1 Hz, 2H), 2.16 (m, 2H); ^{13}C NMR (126 MHz, $CDCl_3$): δ 178.3, 162.2, 139.1, 135.3, 131.3, 122.9, 115.5, 67.0, 30.3, 24.3; HRMS (ESI): $[M+H]^+$ calcd. for $C_{12}H_{14}NO_5$, 252.0872; found, 252.0900.

Synthesis of 2,5-dioxopyrrolidin-1-yl (E)-4-(4-(2-nitrovinyl)phenoxy)butanoate (9a)

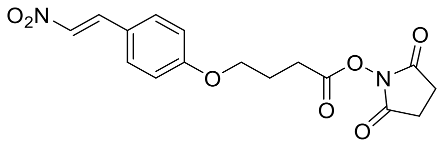

In a 50 ml round bottom flask, (E)-4-(4-(2-nitrovinyl)phenoxy)butanoic acid (**S4**) (502 mg, 2 mmol) and 1-(3-dimethylaminopropyl)-3-ethylcarbodiimide hydrochloride (764 mg, 4 mmol) were dried on high vacuum for 30 min. Next, the reagents were dissolved in anh. DCM (20 ml) under the presence of an inert atmosphere and ice-cold condition. Subsequently, the reaction mixture is allowed to achieve the ambient temperature. To this solution, N-hydroxysuccinimide (920 mg, 8 mmol) was added and stirred at 25 °C, under an inert atmosphere. The progress of the reaction was followed by thin-layer chromatography. After 12 h, the product was extracted using DCM (50 ml × 2). The collected organic fractions were dried over anh. sodium sulfate and concentrated in vacuo. The purification of crude reaction mixture was performed by silica gel flash column chromatography (ethyl acetate:n-hexane, 30:70) to isolate 2,5-dioxopyrrolidin-1-yl (E)-4-(4-(2-nitrovinyl) phenoxy)butanoate **9a** (487.2 mg, 70% yield, yellow solid). TLC (ethyl acetate:*n*-hexane, 50:50 v/v): R_f = 0.62; ^1H NMR (500 MHz, DMSO-

d6): δ 8.11 (d, J = 13.6 Hz, 1H), 8.07 (d, J = 13.6 Hz, 1H), 7.82 (d, J = 8.9 Hz, 2H), 7.06 (d, J = 8.8 Hz, 2H), 4.14 (t, J = 6.2 Hz, 2H), 2.86 (t, J = 7.3 Hz, 2H), 2.82 (s, 4H), 2.10 (m, 2H); ^{13}C NMR (126 MHz, DMSO-d6): δ 171.0, 168.6, 161.5, 139.2, 135.7, 131.9, 122.7, 115.1, 66.1, 26.9, 25.3, 23.7; HRMS (ESI): $[M+Na]^+$ calcd. for $C_{16}H_{16}N_2NaO_7$, 371.0855; found, 371.0846.

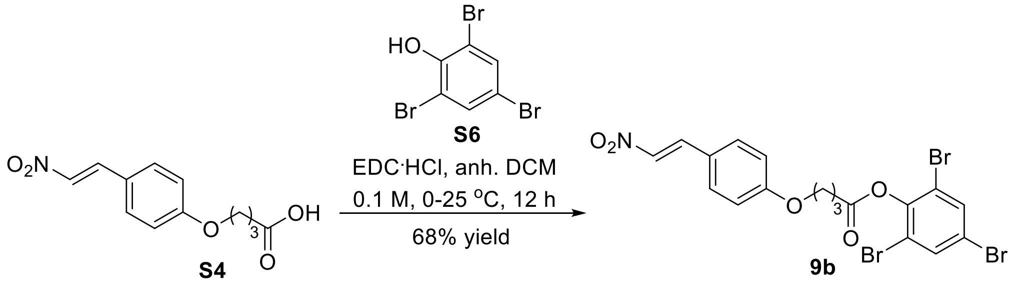

Supplementary Fig. 3. Synthesis of 2,4,6-tribromophenyl (E)-4-(4-(2-nitrovinyl)phenoxy)butanoate **9b**.

Synthesis of 2,4,6-tribromophenyl (E)-4-(4-(2-nitrovinyl)phenoxy)butanoate (9b)

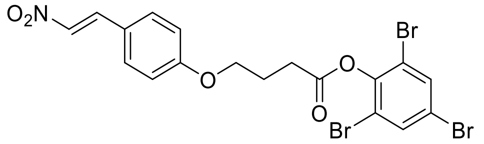

In a 50 ml round bottom flask, (E)-4-(4-(2-nitrovinyl)phenoxy)butanoic acid **S4** (502 mg, 2 mmol) and 1-(3-dimethylaminopropyl)-3-ethylcarbodiimide hydrochloride (764 mg, 4 mmol) were kept on high vacuum for 30 min. Next, the reagents were dissolved in anh. DCM (20 ml) under inert atmosphere and ice-cold conditions. Subsequently, the reaction mixture is allowed to achieve the ambient temperature. To this solution, 2,4,6-tribromophenol (1320 mg, 4 mmol) was added and stirred at 25 °C, under an inert atmosphere. The progress of the reaction was followed by TLC. After 12 h, the product was extracted using DCM (50 ml × 2). The collected organic fractions were dried over anh. sodium sulfate and concentrated in vacuo. The purification of crude reaction mixture was performed by silica gel flash column chromatography (ethyl acetate:n-hexane, 30:70) to isolate 2,4,6-tribromophenyl (E)-4-(4-(2-nitrovinyl)phenoxy)butanoate **9b** (487.2 mg, 70% yield, yellow solid). TLC (ethyl acetate:*n*-hexane, 50:50 v/v): R_f = 0.65; ^1H NMR (500 MHz, $CDCl_3$): δ 7.98 (d, J = 13.6 Hz, 1H), 7.71 (s, 2H), 7.54 – 7.49 (m, 3H), 6.98 (d, J = 8.7 Hz, 2H), 4.17 (t, J = 6.0 Hz, 2H), 2.91 (t, J = 7.2 Hz, 2H), 2.31 (m, 2H); ^{13}C NMR (126 MHz, DMSO-d6): δ 169.6, 162.0, 139.7, 136.2, 135.1, 132.4, 123.1, 120.2, 118.5, 115.6, 66.8, 29.9, 24.3; HRMS (ESI): $[M+H]^+$ calcd. for $C_{18}H_{15}Br_3O_5$, 561.8500; found, 561.8495.

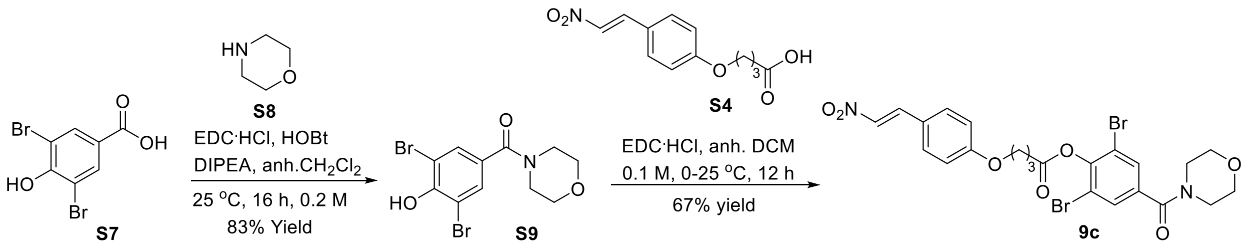

Supplementary Fig. 4. Synthesis of 2,6-dibromo-4-(morpholine-4-carbonyl)phenyl (E)-4-(4-(2-nitrovinyl) phenoxy)butanoate **9c**.

Synthesis of (3,5-dibromo-4-hydroxyphenyl)(morpholino)methanone (S9)

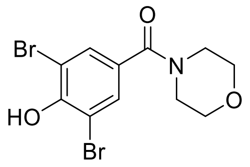

In a 50 ml round bottom flask, 3,5-dibromo-4-hydroxybenzoic acid **S7** (888 mg, 3 mmol), 3-(((ethylimino)methylene)amino)-N,N-dimethylpropan-1-amine (EDC·HCl, 863 mg, 4.5 mmol), and HOBt (405 mg, 3 mmol) were mixed in anh. CH_2Cl_2 (15 ml) at 25 °C. To this solution, DIPEA (1306 µl, 7.5 mmol) and morpholine **S8** (775 µl, 9 mmol) were added and stirred for 6 h. The reaction mixture was concentrated in vacuo. The purification of crude reaction mixture was performed by silica gel flash column chromatography (ethyl acetate:n-hexane, 15:85) to isolate (3,5-dibromo-4-hydroxyphenyl)(morpholino)methanone **S9** (yield 83%, white solid). TLC (ethyl acetate:*n*-hexane, 80:20 v/v): R_f = 0.60; ^{1}H NMR (400 MHz, $CDCl_3$): δ 7.46 (s, 2H), 3.90-3.25 (m, 8H); ^{13}C NMR (101 MHz, $CDCl_3$) δ 167.5, 151.2, 131.4, 129.7, 110.4, 66.9 (2C); HRMS (ESI): $[M+H]^+$ calcd. for $C_{11}H_{12}Br_2NO_3$, 363.9184; found, 363.9173.

Synthesis of 2,6-dibromo-4-(morpholine-4-carbonyl)phenyl (E)-4-(4-(2-nitrovinyl)phenoxy) butanoate (9c)

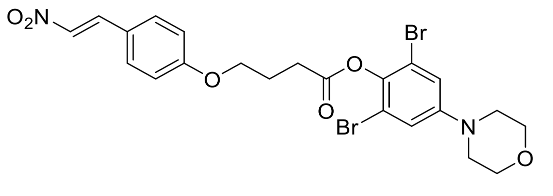

In a 50 ml round bottom flask, (E)-4-(4-(2-nitrovinyl)phenoxy)butanoic acid **S4** (502 mg, 2 mmol) and 1-(3-dimethylaminopropyl)-3-ethylcarbodiimide hydrochloride (764 mg, 4 mmol) were kept on high vacuum for 30 min. Next, the reagents were dissolved in anh. DCM (20 ml) under inert atmosphere and ice-cold conditions. Subsequently, the reaction mixture is allowed to achieve the ambient temperature. To this solution, (3,5-dibromo-4-hydroxyphenyl)(morpholino)methanone (1451 mg, 4 mmol) was added and stirred at 25 °C, under an inert atmosphere. The progress of the reaction was followed by TLC. After 12 h, the product was extracted using DCM (50 ml × 2). The collected organic fractions were dried over anh. sodium sulfate and concentrated in vacuo. The purification of crude reaction mixture was performed by silica gel flash column chromatography (ethyl acetate:n-hexane, 30:70) to isolate

2,6-dibromo-4-morpholinophenyl (E)-4-(4-(2-nitrovinyl)phenoxy)butanoate **9c** (760 mg, 67% yield, crystalline yellow solid). TLC (ethyl acetate:*n*-hexane, 50:50 v/v): R_f = 0.58; ^{1}H NMR (500 MHz, $CDCl_3$): δ 7.97 (d, J = 13.6 Hz, 1H), 7.60 (s, 2H), 7.55 – 7.49 (m, 3H), 6.97 (d, J = 8.8 Hz, 2H), 4.18 (t, J = 6.0 Hz, 2H), 3.87 – 3.40 (m, 8H), 2.94 (t, J = 7.2 Hz, 2H), 2.32 (m, 2H); ^{13}C NMR (126 MHz, DMSO-d_6): δ 169.3, 166.2, 165.4, 161.7, 146.1, 139.4, 136.5, 135.8, 132.1, 131.2, 131.0, 122.8, 117.3, 115.3, 111.6, 66.5, 65.9, 29.7, 24.0; HRMS (ESI): $[M+H]^+$ calcd. for $C_{23}H_{23}Br_2N_2O_7$, 596.9872; found, 596.9867.

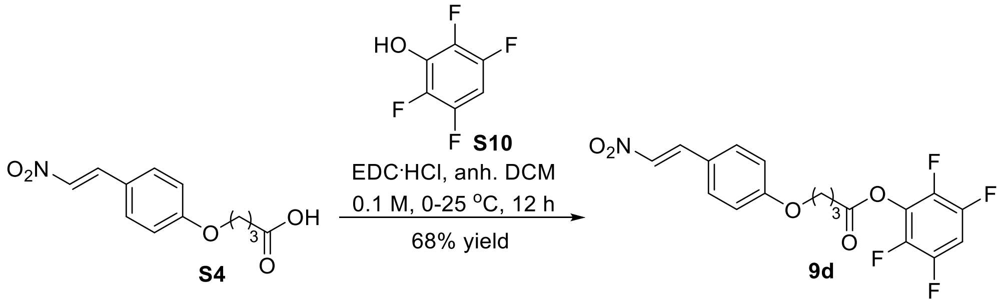

Supplementary Fig. 5. Synthesis of 2,3,5,6-tetrafluorophenyl (E)-4-(4-(2-nitrovinyl)phenoxy)butanoate **9d**.

Synthesis of 2,3,5,6-tetrafluorophenyl (E)-4-(4-(2-nitrovinyl)phenoxy)butanoate (9d)

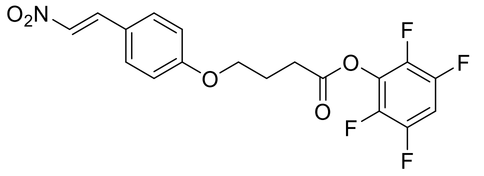

In a 50 ml round bottom flask, (E)-4-(4-(2-nitrovinyl)phenoxy)butanoic acid **S4** (502 mg, 2 mmol) and 1-(3-dimethylaminopropyl)-3-ethylcarbodiimide hydrochloride (764 mg, 4 mmol) were dried on high vacuum for 30 min. Next, the reagents were dissolved in anh. DCM (20 ml) under the presence of an inert atmosphere and ice-cold condition. Subsequently, the reaction mixture is allowed to achieve the ambient temperature. To this solution, 2,3,5,6-tetrafluorophenol (474 µl, 4 mmol) was added and stirred at 25 °C, under an inert atmosphere for 12 h. The progress of the reaction was followed by TLC. The purification of crude reaction mixture was performed by silica gel flash column chromatography (chloroform) to isolate 2,3,5,6-tetrafluorophenyl (E)-4-(4-(2-nitrovinyl)phenoxy)butanoate **9d** (542 mg, 68% yield, yellow solid). TLC ($CHCl_3$): R_f = 0.63; ^{1}H NMR (500 MHz, $CDCl_3$): δ 7.97 (d, J = 13.6 Hz, 1H), 7.56 – 7.47 (m, 3H), 7.01 (m, 1H), 6.97 (d, J = 8.7 Hz, 2 H), 4.15 (t, J = 5.9 Hz, 2H), 2.93 (t, J = 7.2 Hz, 2H), 2.30 (m, 2H); ^{13}C NMR (126 MHz, $CDCl_3$): δ 169.1, 162.1, 146.3 (dtd, J = 248.7, 11.8, 4.1 Hz, 1C), 140.9 (m, 1C), 139.0, 135.3, 131.3, 129.9 (tt, J = 14.4, 4.1 Hz, 1C), 123.0, 115.5, 103.4 (t, J = 22.8 Hz, 1C), 66.6, 30.1, 24.4; HRMS (ESI): $[M+H]^+$ calcd. for $C_{18}H_{14}F_4NO_5$, 400.0808; found, 400.0803.

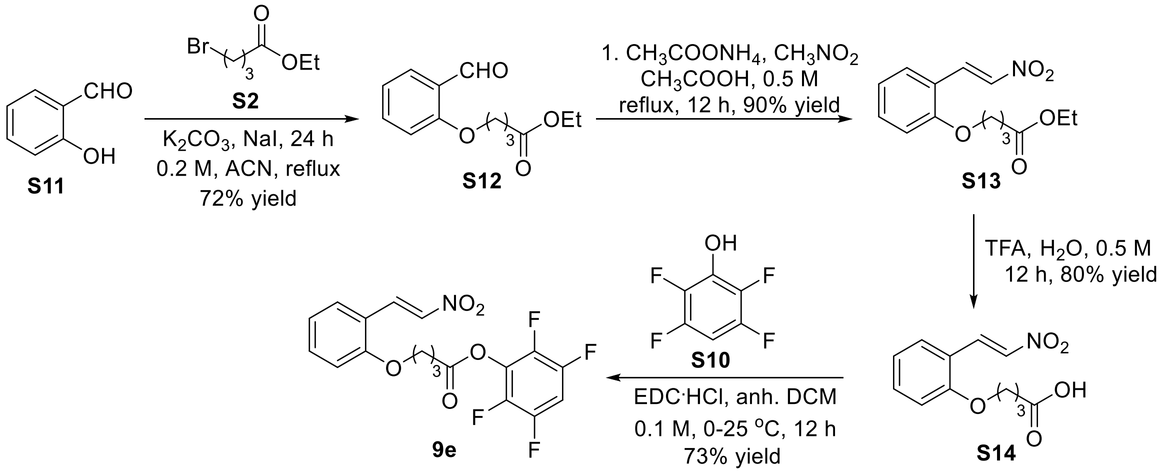

Supplementary Fig. 6. Synthesis of 2,3,5,6-tetrafluorophenyl (E)-4-(2-(2-nitrovinyl)phenoxy)butanoate **9e**.

Synthesis of ethyl 4-(2-formylphenoxy)butanoate (S12)

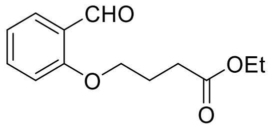

In a 50 ml round bottom flask, *2*-hydroxybenzaldehyde **S11** (611 mg, 5 mmol), ethyl 4-bromobutyrate **S2** (859 µl, 6 mmol), K_2CO_3 (1.037 g, 7.5 mmol) and NaI (374 mg, 2.5 mmol) were dissolved in ACN (25 ml). The reaction mixture was stirred at reflux condition for 24 h followed by filtration to remove potassium carbonate. The filtrate was concentrated to remove ACN and the residue was purified using silica gel flash column chromatography ethyl acetate:n-hexane (3:97). to give **S12** (851 mg, 72% yield, colorless liquid). TLC (ethyl acetate:n-hexane, 10:90 v/v): R_f = 0.60; ^{1}H NMR (500 MHz, $CDCl_3$): δ 10.46 (s, 1H), 7.79 (dd, J = 7.7, 1.8 Hz, 1H), 7.53 – 7.46 (m, 1H), 7.01 – 6.93 (m, 2H), 4.16 – 4.08 (m, 4H), 2.51 (t, J = 7.2 Hz, 2H), 2.20 – 2.11 (m, 2H), 1.22 (t, J = 7.2 Hz, 3H); ^{13}C NMR (126 MHz, $CDCl_3$): δ 189.7, 173.0, 161.2, 136.0, 128.4, 125.0, 120.8, 112.5, 67.4, 60.6, 30.7, 24.5, 14.2; HRMS (ESI): $[M+Na]^+$ calcd. for $C_{13}H_{16}NaO_4$, 259.0946; found, 259.0934.

Synthesis of ethyl (E)-4-(2-(2-nitrovinyl)phenoxy)butanoate (S13)

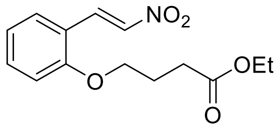

In a 100 ml round bottom flask, ethyl 4-(2-formylphenoxy)butanoate **S12** (2.36 g, 10 mmol), ammonium acetate (2.31 g, 30 mmol), nitromethane (2.7 ml, 50 mmol) and acetic acid (25 ml) were refluxed for 12 h. The reaction mixture turns into a red-brown solution while heating. The progress of the reaction was followed by TLC and 2,4-DNP was utilized to check the consumption of starting material i.e., aldehyde. After the completion of reaction, reaction mixture was allowed to cool and poured onto the ice to get a yellow colour solid, which was filtered using Büchner funnel. The crude reaction mixture was purified by silica gel flash column chromatography using ethyl acetate:n-hexane (2:98) to afford ethyl (E)-4-(2-(2-

nitrovinyl)phenoxy)butanoate **S13** (2.51 g, 90% yield, yellow solid). TLC (ethyl acetate:n-hexane, 10:90 v/v): R_f = 0.91; ^{1}H NMR (700 MHz, $CDCl_3$): δ 8.16 (d, J = 13.6 Hz, 1H), 7.82 (d, J = 13.6 Hz, 1H), 7.47 – 7.41 (m, 2H), 7.01 (m, 1H), 6.97 (d, J = 8.3 Hz, 1H), 4.16 (t, J = 6.3 Hz, 2H), 4.14 (q, J = 7.1 Hz, 2H), 2.54 (t, J = 7.1 Hz, 2H), 2.22 (m, 2H), 1.26 (t, J = 7.2 Hz, 3H); ^{13}C NMR (126 MHz, $CDCl_3$): δ 172.9, 158.7, 138.2, 135.5, 133.6, 132.2, 121.3, 119.3, 112.3, 67.7, 60.8, 30.7, 24.4, 14.3; HRMS (ESI): $[M+Na]^+$ calcd. for $C_{14}H_{17}NNaO_5$, 302.1004; found, 302.0999.

Synthesis of (E)-4-(2-(2-nitrovinyl)phenoxy)butanoic acid (S14)

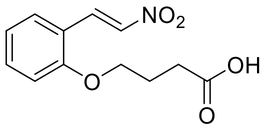

In a 10 ml round bottom flask, ethyl (E)-4-(2-(2-nitrovinyl)phenoxy)butanoate **S13** (279 mg, 1 mmol) and trifluoroacetic acid (307 µl, 4 mmol) were dissolved in water (5 ml).). The reaction mixture was stirred at 100 °C for 12 h. The reaction mixture is allowed to cool and results into green yellow solid, which was filter using Büchner funnel. The progress of the reaction was followed by TLC. The purification of crude reaction mixture was performed by silica gel flash column chromatography using ethyl acetate:n-hexane (30:70) to isolate gave **(**E)-4-(2-(2-nitrovinyl)phenoxy)butanoic acid **S14** (202 mg, 80% yield, yellow solid). TLC (ethyl acetate:n-hexane, 30:70 v/v): R_f = 0.32; ^{1}H NMR (500 MHz, $CDCl_3$): δ 8.14 (d, J = 13.6 Hz, 1H), 7.80 (d, J = 13.6 Hz, 1H), 7.47 – 7.37 (m, 2H), 7.01 – 6.92 (m, 2H), 4.13 (t, J = 6.3 Hz, 2H), 2.54 (d, J = 7.0 Hz, 2H), 2.20 (m, 2H); ^{13}C NMR (126 MHz, $CDCl_3$): δ 158.7, 138.1, 135.6, 133.6, 132.1, 121.3, 119.2, 112.3, 67.7, 30.5, 24.4; HRMS (ESI): $[M+H]^+$ calcd. for $C_{12}H_{14}NO_5$, 252.0872; found, 252.0866.

Synthesis of 2,3,5,6-tetrafluorophenyl (E)-4-(2-(2-nitrovinyl)phenoxy)butanoate (9e)

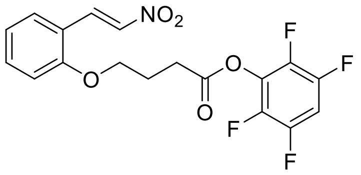

In a 50 ml round bottom flask, (E)-4-(2-(2-nitrovinyl)phenoxy)butanoic acid **S14** (100 mg, 0.25 mmol) and 1-(3-dimethylaminopropyl)-3-ethylcarbodiimide hydrochloride (96 mg, 0.5 mmol) were dried on high vacuum for 30 min. Next, the reagents were dissolved in anh. DCM (2.5 ml) under the presence of an inert atmosphere and ice-cold condition. Subsequently, the reaction mixture is allowed to achieve the ambient temperature. To this solution, 2,3,5,6-tetrafluorophenol **S10** (59 µl, 0.5 mmol) was added and stirred at 25 °C, under an inert atmosphere for 12 h. The progress of the reaction was followed by TLC. The purification of crude reaction mixture was performed by silica gel flash column chromatography (chloroform) to isolate 2,3,5,6-tetrafluorophenyl (E)-4-(2-(2-nitrovinyl)phenoxy)butanoate **9e** (115 mg, 73% yield, yellow solid). TLC ($CHCl_3$): R_f = 0.64; ^{1}H NMR (500 MHz, $CDCl_3$): δ 8.20 (d, J = 13.7 Hz, 1H), 7.81 (d, J = 13.6 Hz, 1H), 7.51 – 7.43 (m, 2H), 7.07 – 6.95 (m, 2H), 4.24 (t, J = 6.2 Hz, 2H), 2.95 (t, J = 7.0 Hz, 2H), 2.38 (m, 2H); ^{13}C NMR (126 MHz, $CDCl_3$): δ 168.9, 158.5,

146.5 (dtd, J = 248.4, 12.2, 3.7 Hz, 1C), 140.7 (m), 138.2, 135.2, 133.7, 131.9, 129.7 (m, 1C), 121.6, 119.4, 112.4, 103.5 (t, J = 22.8 Hz, 1C), 67.1, 30.0, 24.4; HRMS (ESI): $[M+H]^+$ calcd. for $C_{18}H_{14}F_4NO_5$, 400.0808; found, 400.0803.

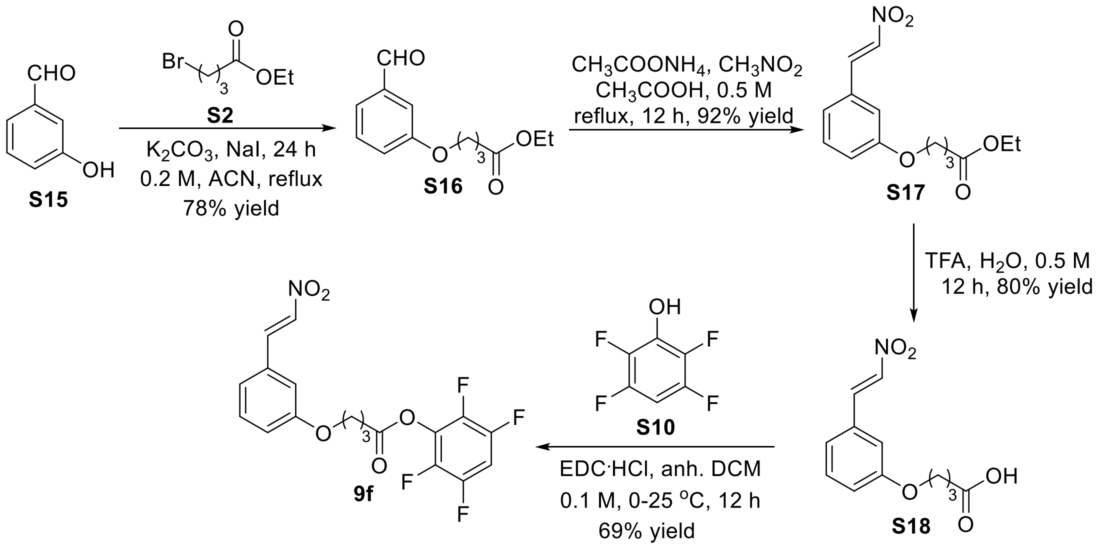

Supplementary Fig. 7. Synthesis of 2,3,5,6-tetrafluorophenyl (E)-4-(3-(2-nitrovinyl)phenoxy)butanoate **9f**.

Synthesis of ethyl 4-(3-formylphenoxy)butanoate (S16)

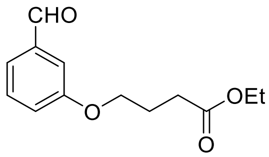

In a 50 ml round bottom flask, 3-hydroxybenzaldehyde **S15** (611 mg, 5 mmol), ethyl 4-bromobutyrate **S2** (859 µl, 6 mmol), K_2CO_3 (1.037 g, 7.5 mmol) and NaI (374 mg, 2.5 mmol) were dissolved in ACN (25 ml). The reaction mixture was stirred at reflux condition for 24 h followed by filtration to remove potassium carbonate. The filtrate was concentrated to remove ACN and the residue was purified using silica gel flash column chromatography ethyl acetate:n-hexane (3:97); to give ethyl 4-(3-formylphenoxy)butanoate **S16** (921 mg, 78% yield, colorless liquid). TLC (ethyl acetate:n-hexane 10:90 v/v): R_f = 0.60; ^{1}H NMR (500 MHz, $CDCl_3$): δ 9.94 (s, 1H), 7.46 – 7.37 (m, 2H), 7.35 (d, J = 1.5 Hz, 1H), 7.14 (dt, J = 7.0, 2.4 Hz, 1H), 4.13 (q, J = 7.1 Hz, 2H), 4.05 (t, J = 6.2 Hz, 2H), 2.50 (t, J = 7.3 Hz, 2H), 2.16 – 2.07 (m, 2H), 1.23 (t, J = 7.2 Hz, 3H). ^{13}C NMR (126 MHz, $CDCl_3$): δ 192.1, 173.1, 159.5, 137.9, 130.1, 123.5, 121.9, 112.9, 67.1, 60.6, 30.8, 24.6, 14.3; HRMS (ESI): $[M+H]^+$ calcd. for $C_{13}H_{17}O_4$, 237.1127; found, 237.1032.

Synthesis of ethyl (E)-4-(3-(2-nitrovinyl)phenoxy)butanoate (S17)

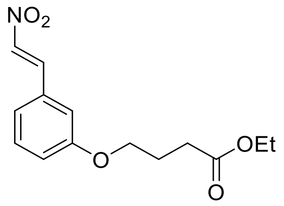

In a 100 ml round bottom flask, ethyl 4-(3-formylphenoxy)butanoate **S16** (2.36 g, 10 mmol), ammonium acetate (2.31 g, 30 mmol), nitromethane (2.7 ml, 50 mmol) and acetic acid (25 ml) were refluxed for 12 h. The reaction mixture turns into a red-brown solution while heating. The progress of the reaction was followed by TLC and 2,4-DNP was utilized to check the consumption of starting material i.e., aldehyde. After the completion of reaction, reaction mixture was allowed to cool and poured onto the ice to get a yellow colour solid, which was filtered using Büchner funnel. The crude reaction mixture was purified by silica gel flash column chromatography using ethyl acetate:n-hexane (2:98) to afford ethyl ethyl (E)-4-(3-(2-nitrovinyl)phenoxy)butanoate **S17** (2.56 g, 92% yield, yellow solid). TLC (ethyl acetate:n-hexane 10:90 v/v): R_f = 0.90; ^{1}H NMR (700 MHz, $CDCl_3$): δ 7.95 (d, J = 13.6 Hz, 1H), 7.57 (d, J = 13.6 Hz, 1H), 7.35 (m, 1H), 7.13 (d, J = 7.5 Hz, 1H), 7.05 (s, 1H), 7.02 (d, J = 8.3 Hz, 1H), 4.15 (q, J = 7.1 Hz, 2H), 4.04 (t, J = 6.1 Hz, 2H), 2.52 (t, J = 7.2 Hz, 2H), 2.13 (m, 2H), 1.26 (t, J = 7.1 Hz, 3H); ^{13}C NMR (176 MHz, $CDCl_3$): δ 173.2, 159.5, 139.2, 137.5, 131.5, 130.6, 122.0, 118.6, 114.6, 67.1, 60.7, 30.8, 24.6, 14.4; HRMS (ESI): $[M+Na]^+$ calcd. for $C_{14}H_{17}NNaO_5$, 302.1004; found, 302.1004.

Synthesis of (E)-4-(3-(2-nitrovinyl)phenoxy)butanoic acid (S18)

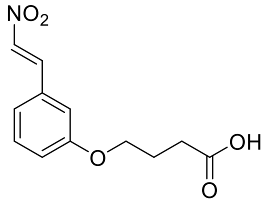

In a 10 ml round bottom flask, ethyl (E)-4-(3-(2-nitrovinyl)phenoxy)butanoate **S17** (279 mg, 1 mmol) and trifluoroacetic acid (307 µl, 4 mmol) were dissolved in water (5 ml).). The reaction mixture was stirred at 100 °C for 12 h. The reaction mixture is allowed to cool results into green yellow solid, which was filter using Büchner funnel. The progress of the reaction was followed by TLC. The purification of crude reaction mixture was performed by silica gel flash column chromatography using ethyl acetate:n-hexane (30:70) to isolate gave (E)-4-(3-(2-nitrovinyl)phenoxy)butanoic acid **S18** (202 mg, 80% yield, yellow solid). TLC (ethyl acetate:n-hexane 30:70 v/v): R_f = 0.34; ^{1}H NMR (700 MHz, CD_3OD): δ 8.00 (d, J = 13.6 Hz, 1H), 7.89 (d, J = 13.6 Hz, 1H), 7.35 (m, 1H), 7.24 (d, J = 7.5 Hz, 1H), 7.25 (s, 1H) 7.06 (d, J = 8.4 Hz, 1H), 4.06 (t, J = 2.0 Hz, 2H), 2.50 (t, J = 7.3 Hz, 2H), 2.07 (m, 2H); ^{13}C NMR (176 MHz, CD_3OD): δ 177.2, 161.2, 140.2, 139.2, 133.4, 131.6, 123.4, 119.8, 115.7, 68.5, 31.6, 26.0; HRMS (ESI): $[M+Na]^+$ calcd. for $C_{12}H_{13}NNaO_5$, 274.0691; found, 274.0686.

Synthesis of 2,3,5,6-tetrafluorophenyl (E)-4-(3-(2-nitrovinyl)phenoxy)butanoate (9f)

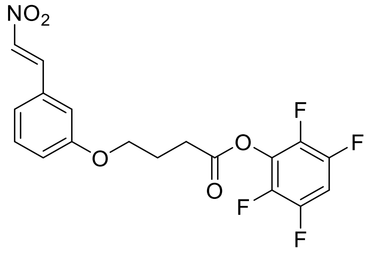

In a 50 ml round bottom flask, (E)-4-(3-(2-nitrovinyl)phenoxy)butanoic acid **S18** (100 mg, 0.25 mmol) and 1-(3-dimethylaminopropyl)-3-ethylcarbodiimide hydrochloride (96 mg, 0.5 mmol) were dried on high vacuum for 30 min. Next, the reagents were dissolved in anh. DCM (2.5 ml) under the presence of an inert atmosphere and ice-cold condition. Subsequently, the reaction mixture is allowed to achieve the ambient temperature. To this solution, 2,3,5,6-tetrafluorophenol **S10** (59 µ*l*, 0.5 mmol) was added and stirred at 25 °C, under an inert atmosphere for 12 h. The progress of the reaction was followed by TLC. The purification of crude reaction mixture was performed by silica gel flash column chromatography (chloroform) to isolate 2,3,5,6-tetrafluorophenyl (E)-4-(3-(2-nitrovinyl)phenoxy)butanoate **9f** (108 mg, 69% yield, yellow solid). TLC ($CHCl_3$): R_f = 0.65; ^{1}H NMR (500 MHz, $CDCl_3$): δ 7.97 (d, J = 13.7 Hz, 1H), 7.57 (d, J = 13.6 Hz, 1H), 7.37 (m, 1H), 7.16 (d, J = 7.6 Hz, 1H), 7.09 – 6.96 (m, 3H), 4.13 (t, J = 6.0 Hz, 2H), 2.93 (t, J = 7.2 Hz, 2H), 2.29 (m, 2H); ^{13}C NMR (126 MHz, $CDCl_3$): δ 169.2, 159.4, 146.2 (dtd, J = 248.6, 12.1, 4.3 Hz, 1C), 141.8 (m, 1C), 139.0, 137.6, 131.6, 130.7, 129.7 (m, 1C), 122.2, 118.5, 114.7, 103.4 (t, J = 22.8 Hz, 1C), 66.6, 30.1, 24.5; HRMS (ESI): $[M+H]^+$ calcd. for $C_{18}H_{14}F_4NO_5$, 400.0808; found, 400.0803.

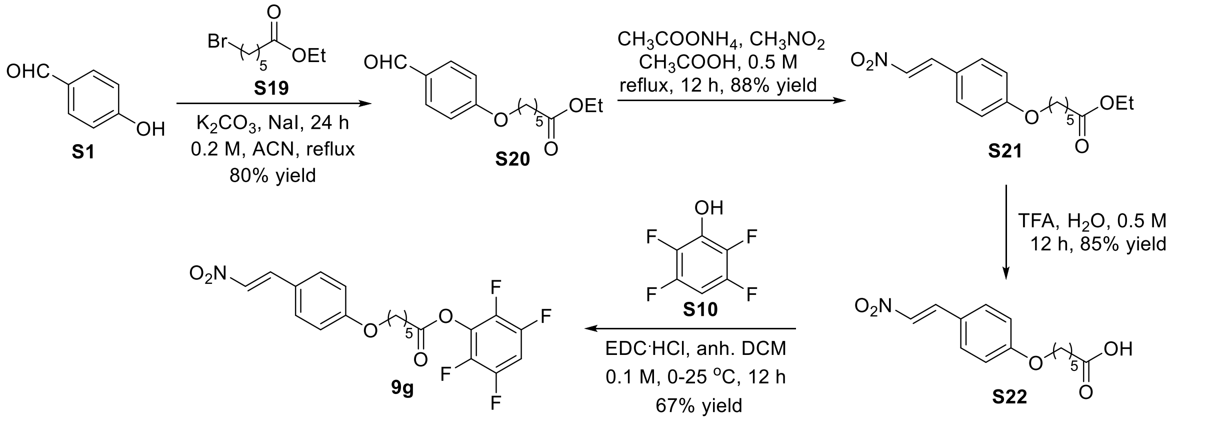

Supplementary Fig. 8. Synthesis of 2,3,5,6-tetrafluorophenyl (E)-6-(4-(2-nitrovinyl)phenoxy)hexanoate **9g**.

Synthesis of ethyl 6-(4-formylphenoxy)hexanoate (S20)

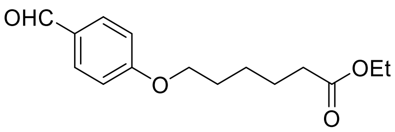

In a 50 ml round bottom flask, 4-hydroxybenzaldehyde **S1** (610 mg, 5 mmol), ethyl 6-bromohexanoate **S19** (1 ml, 6 mmol), K_2CO_3 (1.037 g, 7.5 mmol) and NaI (374 mg, 2.5 mmol) were dissolved in ACN (25 ml). The reaction mixture was stirred at reflux condition for 24 h followed by filtration to remove potassium carbonate. The filtrate was concentrated to remove ACN and the residue was purified using silica gel flash column chromatography (ethyl acetate:n-hexane 3:97) to give ethyl 6-(4-formylphenoxy)hexanoate **S20** (1.05 g, 80% yield, colorless liquid). TLC (ethyl acetate:n-hexane 10:90 v/v): R_f = 0.57; ^{1}H NMR (500 MHz, $CDCl_3$): δ 9.85 (s, 1H), 7.80 (d, J = 8.7 Hz, 2H), 6.96 (d, J = 8.7 Hz, 2H), 4.11 (q, J = 7.1 Hz, 2H), 4.02 (t, J = 6.3 Hz, 2H), 2.32 (t, J = 7.5 Hz, 2H), 1.82 (m, 2H), 1.69 (m, 2H), 1.50 (m, 2H), 1.23 (t, J = 7.2 Hz, 3H); ^{13}C NMR (126 MHz, $CDCl_3$): δ 190.9, 173.6, 164.2, 132.1, 129.9, 114.8, 68.1, 60.4, 34.3, 28.8, 25.6, 24.7, 14.3; HRMS (ESI): $[M+H]^+$ calcd. for $C_{15}H_{21}O_4$, 265.1440; found, 265.1434.

Synthesis of ethyl (E)-6-(4-(2-nitrovinyl)phenoxy)hexanoate (S21)

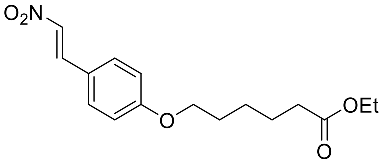

In a 100 ml round bottom flask, ethyl 6-(4-formylphenoxy)hexanoate **S20** (1.32 g, 5 mmol), ammonium acetate (1.15 g, 15 mmol), nitromethane (1.35 ml, 25 mmol) and acetic acid (12.5 ml) were refluxed for 12 h. The reaction mixture turns into a red-brown solution while heating. The progress of the reaction was followed by TLC and 2,4-DNP was utilized to check the consumption of starting material i.e., aldehyde. After the completion of reaction, reaction mixture was allowed to cool and poured onto the ice to get a yellow colour solid, which was filtered using Büchner funnel. The crude reaction mixture was purified by silica gel flash column chromatography using ethyl acetate:n-hexane (2:98) to afford ethyl (E)-6-(4-(2-nitrovinyl)phenoxy)hexanoate **S21** (1.34 g, 88% yield, yellow solid). TLC (ethyl acetate:n-hexane 10:90 v/v): R_f = 0.85; ^{1}H NMR (500 MHz, $CDCl_3$): δ 7.97 (d, J = 13.6 Hz, 1H), 7.51 (d, J = 13.6 Hz, 1H), 7.48 (d, J = 8.7 Hz, 2H), 6.93 (d, J = 8.7 Hz, 2H), 4.13 (q, J = 7.1 Hz, 2H), 4.01 (t, J = 6.4 Hz, 2H), 2.34 (t, J = 7.4 Hz, 2H), 1.82 (m, 2H), 1.71 (m, 2H), 1.52 (m, 2H), 1.25 (t, J = 7.1 Hz, 3H); ^{13}C NMR (126 MHz, $CDCl_3$): δ 173.7, 162.6, 139.2, 135.1, 131.3, 122.6, 115.5, 68.2, 60.4, 34.3, 28.9, 25.7, 24.8, 14.4; HRMS (ESI): $[M+Na]^+$ calcd. for $C_{16}H_{21}NNaO_5$, 330.1317; found, 330.1312.

Synthesis of (E)-6-(4-(2-nitrovinyl)phenoxy)hexanoic acid (S22)

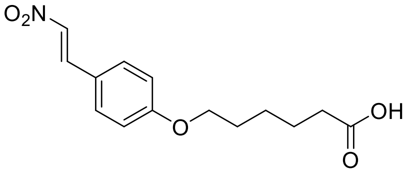

In a 10 ml round bottom flask, ethyl (E)-6-(4-(2-nitrovinyl)phenoxy)hexanoate **S21** (307 mg, 1 mmol) and trifluoroacetic acid (307 µl, 4 mmol) were dissolved in water (5 ml). The reaction mixture was stirred at 100 ˚C for 12 h. The reaction mixture is allowed to cool results into green yellow solid, which was filter using Büchner funnel. The progress of the reaction was followed by TLC. The purification of crude reaction mixture was performed by silica gel flash column chromatography using ethyl acetate:n-hexane (30:70) to isolate gave (E)-6-(4-(2-nitrovinyl)phenoxy)hexanoic acid **S22** (237 mg, 85% yield, crystalline yellow solid). TLC (ethyl acetate:n-hexane 30:70 v/v): R_f = 0.28; ^{1}H NMR (500 MHz, $CDCl_3$): δ 7.90 (d, J = 13.6, 1H), 7.53 – 7.39 (m, 3H), 6.86 (m, 2H), 3.95 (m, 2H), 2.28 (m, 2H), 1.76 (m, 2H), 1.62 (m, 2H), 1.45 (m, 2H); ^{13}C NMR (126 MHz, $CDCl_3$): δ 162.5, 139.3, 134.9, 131.3, 122.4, 115.4, 68.1, 33.9, 25.5, 24.6; HRMS (ESI): $[M+H]^+$ calcd. for $C_{14}H_{18}NO_5$, 280.1185; found, 280.1179.

Synthesis of 2,3,5,6-tetrafluorophenyl (E)-6-(4-(2-nitrovinyl)phenoxy)hexanoate (9g)

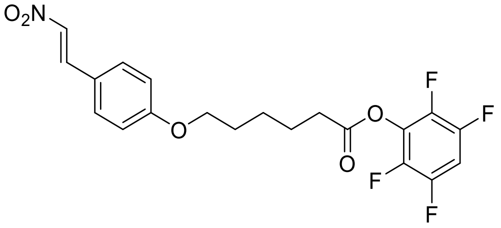

In a 50 ml round bottom flask, (E)-6-(4-(2-nitrovinyl)phenoxy)hexanoic acid **S22** (100 mg, 0.35 mmol) and 1-(3-dimethylaminopropyl)-3-ethylcarbodiimide hydrochloride (134 mg, 0.7 mmol) were dried on high vacuum for 30 min. Next, the reagents were dissolved in anh. DCM (3.5 ml) under the presence of an inert atmosphere and ice-cold condition. Subsequently, the reaction mixture is allowed to achieve the ambient temperature. To this solution, 2,3,5,6-tetrafluorophenol **S10** (83 µ*l*, 0.5 mmol) was added and stirred at 25 ˚C, under an inert atmosphere for 12 h. The progress of the reaction was followed by TLC. The purification of crude reaction mixture was performed by silica gel flash column chromatography (Chloroform) to isolate 2,3,5,6-tetrafluorophenyl (E)-6-(4-(2-nitrovinyl)phenoxy)hexanoate **9g** (101 mg, 67% yield, yellow solid). TLC ($CHCl_3$): R_f = 0.60; ^{1}H NMR (500 MHz, $CDCl_3$): δ 7.97 (d, J = 13.6 Hz, 1H), 7.56 – 7.46 (m, 3H), 7.00 (m, 1H), 6.94 (d, J = 8.6 Hz, 2H) 4.05 (t, J = 6.3 Hz, 2H), 2.73 (t, J = 7.3 Hz, 2H), 1.93 – 1.82 (m, 4H), 1.6 (m, 2H); ^{13}C NMR (126 MHz, $CDCl_3$): δ 169.4, 162.5, 146.2 (dtd, J = 248.6, 12.1, 4.1 Hz, 1C), 141.7 (m, 1C), 139.2, 135.2, 131.3, 129.8 (m, 1C), 122.7, 115.5, 103.3 (t, J = 22.8 Hz), 68.0, 33.4, 28.8, 25.5, 24.6; HRMS (ESI): $[M+H]^+$ calcd. for $C_{20}H_{18}F_4NO_5$, 428.1121; found, 428.1116.

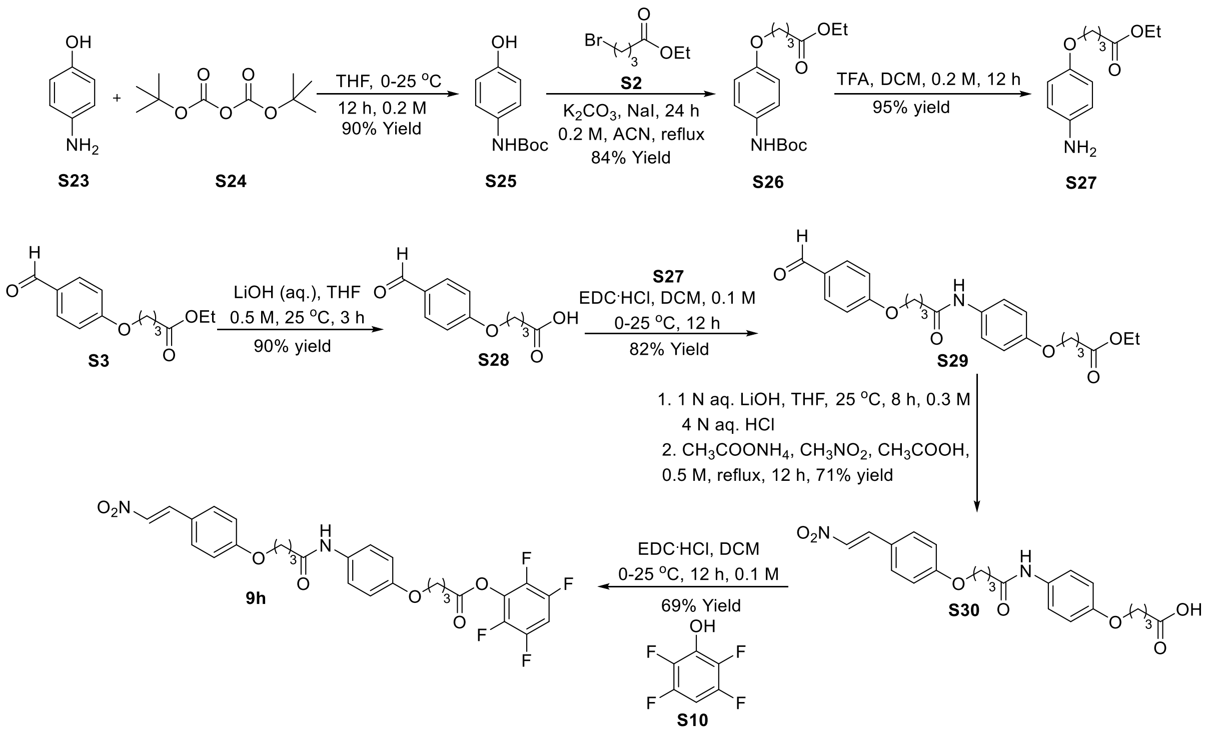

Supplementary Fig. 9. Synthesis of 2,3,5,6-tetrafluorophenyl (E)-4-(4-(4-(4-(2-nitrovinyl)phenoxy) butanamido)phenoxy)butanoate **9h**.

Synthesis of tert-butyl (4-hydroxyphenyl)carbamate (S25)

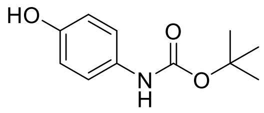

In a dry 100 ml round bottom flask, 4-aminophenol **S23** (2.18 g, 20 mmol) was dissolved in THF (50 ml). To this solution, di-tert-butyl dicarbonate **S24** (4.59 ml, 24 mmol) in THF (50 ml) was added slowly at 0 °C by a dropping funnel. After 2 h, the reaction mixture was brought to 25 °C and stirred for 10 h. The reaction mixture was concentrated in vacuo. Subsequently, washing of the crude solid by hexane rendered the analytically pure product tert-butyl (4-hydroxyphenyl)carbamate **S25** (3.75 g, 90% yield, white solid). TLC (ethyl acetate:n-hexane, 20:80 v/v): R_f = 0.40; ^{1}H NMR (500 MHz, $CDCl_3$): δ 7.20-7.10 (m, 2H), 6.73 (d, J = 8.7 Hz, 2H), 6.34 (s, 1H), 5.36 (s, 1H), 1.51 (s, 9H); ^{13}C NMR (126 MHz, $CDCl_3$): δ 153.6, 152.1, 131.0, 121.4, 115.7, 80.4, 28.4; HRMS (ESI): $[M+Na]^+$ calcd. for $C_{11}H_{15}NNaO_3$, 232.0950; found, 232.0938.

Synthesis of ethyl 4-(4-((tert-butoxycarbonyl)amino)phenoxy)butanoate (S26)

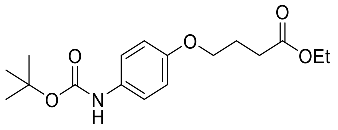

In a 100 ml round bottom flask, tert-butyl (4-hydroxyphenyl)carbamate **S25** (2.092 g, 10 mmol), ethyl 4-bromobutanoate **S2** (1.717 ml, 12 mmol), K_2CO_3 (2.764 g, 20 mmol) and NaI (749 mg, 5 mmol) were dissolved in ACN (50 ml) and refluxed for 24 h. The reaction mixture

was concentrated in vacuo and subjected to partial purification by solvent-solvent extraction using ethyl acetate and water. The organic layer was separated, dried over anh. sodium sulfate and filtered. The filtrate was concentrated under reduced pressure. The crude compound was purified by silica gel flash column chromatography using ethyl acetate:n-hexane (20:80) to afford ethyl 4-(4-((tert-butoxycarbonyl)amino)phenoxy)butanoate **S26** (2.70 g, 84% yield, white solid). TLC (ethyl acetate:n-hexane, 20:80 v/v): R_f = 0.21; ^{1}H NMR (500 MHz, $CDCl_3$): δ 7.26-7.19 (m, 2H), 6.81 (d, J = 9.0 Hz, 2H), 6.36 (s, 1H), 4.14 (q, J = 7.1 Hz, 2H), 3.96 (t, J = 6.0 Hz, 2H), 2.50 (t, J = 7.3 Hz, 2H), 2.14-2.04 (m, 2H), 1.50 (s, 9H), 1.25 (t, J = 7.1 Hz, 3H); ^{13}C NMR (126 MHz, $CDCl_3$): δ 173.4, 155.1, 153.3, 131.6, 120.7, 115.0, 80.4, 67.2, 60.6, 31.0, 28.5, 24.8, 14.4; HRMS (ESI): $[M+Na]^+$ calcd. for $C_{17}H_{25}NNaO_5$, 346.1630; found, 346.1649.

Synthesis of ethyl 4-(4-aminophenoxy)butanoate (S27)

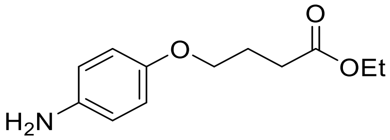

In a 100 ml round bottom flask, ethyl 4-(4-((tert-butoxycarbonyl)amino)phenoxy)butanoate **S26** (1.62 g, 5 mmol) was dissolved in CH_2Cl_2 (25 ml). To this solution, trifluoroacetic acid (1.53 ml, 20 mmol) was added slowly over 15 minutes and stirred at 25 °C for 12 h. The reaction mixture was quenched with sodium bicarbonate (slow addition), followed by addition of CH_2Cl_2. The organic layer was separated, dried with anh. sodium sulfate, filtered, and concentrated in vacuo to give ethyl 4-(4-aminophenoxy)butanoate **S27** (1.05 g, 95% yield, pale yellow liquid). TLC (ethyl acetate:n-hexane, 30:70 v/v): R_f = 0.25; ^{1}H NMR (500 MHz, $CDCl_3$): δ 6.76-6.70 (m, 2H), 6.66-6.61 (m, 2H), 4.14 (q, J = 7.1 Hz, 2H), 3.92 (t, J = 6.1 Hz, 2H), 3.41 (s, 2H), 2.49 (t, J = 7.3 Hz, 2H), 2.12-2.01 (m, 2H), 1.25 (t, J = 7.1 Hz, 3H); ^{13}C NMR (126 MHz, $CDCl_3$): δ 173.5, 152.1, 140.2, 116.5, 115.8, 67.6, 60.5, 31.0, 24.9, 14.4; HRMS (ESI): $[M+H]^+$ calcd. for $C_{12}H_{17}NO_3$, 224.1287; found, 224.1265.

Synthesis of 4-(4-formylphenoxy)butanoic acid (S28)

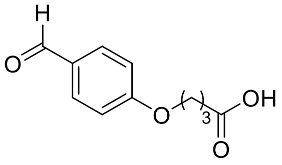

In a 25 ml round bottom flask, ethyl 4-(4-formylphenoxy)butanoate **S3** (236 mg, 1 mmol) and 1 N LiOH (aq., 3 ml) were dissolved in THF (0.3 ml) and stirred at room temperature. The progress of the reaction was followed by TLC. After 3 h, the reaction mixture was concentrated to remove THF and rest part was quenched with 4 N HCl (aq.) and compounds were extracted with ethyl acetate. The collected organic fractions were dried over anh. sodium sulfate and concentrated in vacuo. The purification of crude reaction mixture was performed by silica gel flash column chromatography using ethyl acetate:hexane (70:30) to isolate 4-(4-formylphenoxy)butanoic acid **S28** (187 mg, 90% yield, crystalline solid). TLC (ethyl acetate:n-

hexane, 50:50 v/v): R_f = 0.52; ^{1}H NMR (500 MHz, $CDCl_3$): δ 9.89 (s, 1H), 7.83 (d, J = 8.7 Hz, 2H), 6.99 (d, J = 8.7 Hz, 2H), 4.12 (t, J = 6.1 Hz, 2H), 2.61 (t, J = 7.2 Hz, 2H), 2.17 (m, 2H); ^{13}C NMR (176 MHz, $CDCl_3$): δ 191.0, 175.4, 164.0, 132.2, 130.2, 114.9, 67.1, 29.8, 24.4; HRMS (ESI): $[M+H]^+$ calcd. for $C_{11}H_{13}O_4$, 209.0814; found, 209.0808.

Synthesis of ethyl 4-(4-(4-(4-formylphenoxy)butanamido)phenoxy)butanoate (S29)

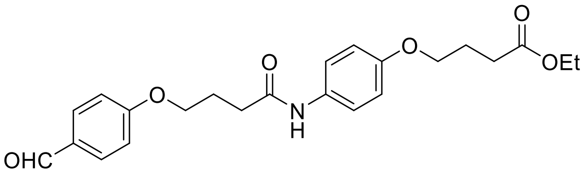

In a 50 ml round bottom flask 4-(4-formylphenoxy)butanoic acid **S28** (416 mg, 2 mmol) and 1-(3-dimethylaminopropyl)-3-ethylcarbodiimide hydrochloride (764 mg, 4 mmol) were dried on high vacuum for 30 min. Next, the reagents were dissolved in anh. DCM (20 ml) under the presence of an inert atmosphere and ice-cold condition. Subsequently, the reaction mixture is allowed to achieve the ambient temperature. To this solution ethyl 4-(4-aminophenoxy)butanoate (892 mg, 4 mmol) was added and stirred at 25 °C, under an inert atmosphere. The progress of the reaction was followed by thin-layer chromatography. After 12 h, the product was extracted using DCM (50 ml × 2). The collected organic fractions were dried over anh. sodium sulfate and concentrated in vacuo. The purification of crude reaction mixture was performed by silica gel flash column chromatography (ethyl acetate:n-hexane, 30:70) to ethyl 4-(4-(4-(4-formylphenoxy)butanamido)phenoxy)butanoate **S29** (677 mg, 82% yield, crystalline yellow solid). TLC (ethyl acetate:n-hexane, 50:50 v/v): R_f = 0.68; ^{1}H NMR (500 MHz, $CDCl_3$): δ 9.87 (s, 1H), 7.81 (d, J = 7.6 Hz, 2H), 7.37 (d, J = 8.8 Hz, 2H), 7.28 (s, 1H), 6.98 (d, J = 8.4 Hz, 2H), 6.82 (d, J = 8.8 Hz, 2H), 4.19 – 4.10 (m, 4H), 3.97 (t, J = 6.1 Hz, 2H), 2.56 (t, J = 7.1 Hz, 2H), 2.49 (t, J = 7.3 Hz, 2H), 2.28 – 2.18 (m, 2H), 2.13 – 2.04 (m, 2H), 1.25 (t, J = 7.1 Hz, 4H); ^{13}C NMR (126 MHz, $CDCl_3$): δ 191.0, 173.4, 170.2, 164.0, 155.8, 132.2, 131.0, 130.1, 121.8, 114.9, 67.4, 67.2, 60.6, 33.6, 30.9, 25.0, 24.8, 14.4; HRMS (ESI): $[M+H]^+$ calcd. for $C_{23}H_{28}NO_6$, 414.1917; found, 414.1965.

Synthesis of (E)-4-(4-(4-(4-(2-nitrovinyl)phenoxy)butanamido)phenoxy)butanoic acid (S30)

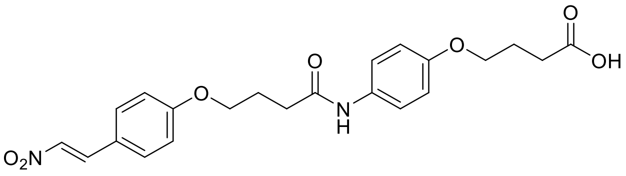

In a 100 ml round bottom flask, ethyl 4-(4-(4-(4-formylphenoxy)butanamido)phenoxy)butanoate **S29** (770.24 mg, 2 mmol), 1 (N) aq. LiOH were added into THF solvent and kept the whole reaction mixture at room temperature with constant stirring. Reaction was monitored by thin-layer chromatography. After 8 h THF was completely concentrated in vacuo and neutralized the whole reaction mixture with 4 (N) aq. HCl solution, the product was extracted using ethyl acetate (50 ml × 2). The collected organic fractions were dried over anh. sodium sulfate and concentrated in vacuo to obtain the product.

Followed by obtained product was treated with ammonium acetate (462 mg, 6 mmol), nitromethane (535 µl, 10 mmol) and acetic acid (5 ml) were refluxed for 12 h. The reaction mixture turns into a red-brown solution while heating. The progress of the reaction was followed by TLC and 2,4-DNP was utilized to check the consumption of starting material i.e., aldehyde. After the completion of reaction, reaction mixture was allowed to cool and poured onto the ice to get a yellow colour solid, which was filtered using Büchner funnel. The crude reaction mixture was purified by silica gel flash column chromatography using ethyl acetate:n-hexane (2:98) to (E)-4-(4-(4-(4-(2-nitrovinyl)phenoxy)butanamido)phenoxy)butanoic acid **S30** (770 mg, 71% yield, crystalline yellow solid). TLC (ethyl acetate:n-hexane, 10:90 v/v): R_f = 0.95; ^{1}H NMR (500 MHz, $CDCl_3$): δ 7.86 (d, J = 13.5 Hz, 1H), 7.44 (d, J = 13.5 Hz, 1H), 7.39 (d, J = 8.2 Hz, 1H), 7.12 (s, 1H), 7.06 (t, J = 8.0 Hz, 1H), 6.93 (d, J = 8.3 Hz, 1H), 6.84 (d, J = 8.4 Hz, 2H), 6.51 (d, J = 8.4 Hz, 1H), 4.00 (t, 2H), 3.88 (t, 2H), 2.53 – 2.42 (m, 2H), 2.41 – 2.34 (m, 1H), 2.13 – 2.04 (m, 2H), 2.00 – 1.91 (m, 2H), 1.16 (t, 4H); ^{13}C NMR (126 MHz, $CDCl_3$): δ 175.8., 162.3, 159.19, 139.3, 134.9, 131.3, 129.5, 122.5, 115.3, 112.2, 110.2, 106.3, 67.3, 66.7, 33.2, 30.5, 24.8, 24.5; HRMS (ESI): $[M+H]^+$ calcd. for $C_{22}H_{25}N_2O_7$, 429.1662; found, 429.1656.

Synthesis of 2,3,5,6-tetrafluorophenyl (E)-4-(4-(4-(4-(2-nitrovinyl)phenoxy)butanamido) phenoxy)butanoate (9h)

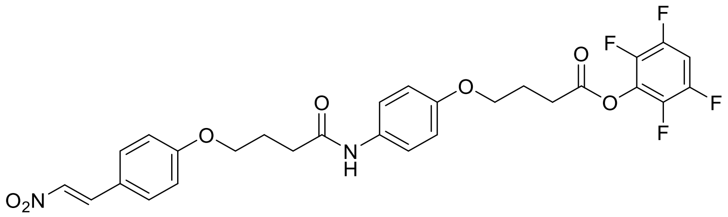

In a 50 ml round bottom flask, (E)-4-(4-(4-(4-(2-nitrovinyl)phenoxy)butanamido) phenoxy)butanoic acid **S30** (856 mg, 2 mmol) and 1-(3-dimethylaminopropyl)-3-ethylcarbodiimide hydrochloride (764 mg, 4 mmol) were dried on high vacuum for 30 min. Next, the reagents were dissolved in anh. DCM (20 ml) under the presence of an inert atmosphere and ice-cold condition. Subsequently, the reaction mixture is allowed to achieve the ambient temperature. 2,3,5,6-tetrafluorophenol **S10** (474 µl, 4 mmol) was added and stirred at 25 ^{o}C, under an inert atmosphere for 12 h. The purification of crude reaction mixture was performed by silica gel flash column chromatography (ethyl acetate:n-hexane, 30:70) to isolate 2,3,5,6-tetrafluorophenyl (E)-4-(4-(4-(4-(2-nitrovinyl)phenoxy)butanamido) phenoxy)butanoate **9h** (793 mg, 69% yield, crystalline yellow solid). TLC (ethyl acetate:n-hexane, 50:50 v/v): R_f = 0.67; ^{1}H NMR (500 MHz, $CDCl_3$): δ 7.96 (d, J = 13.6 Hz, 1H), 7.51 (d, J = 13.6 Hz, 1H), 7.49 (d, J = 8.7 Hz, 2H), 7.33 (s, 1H), 7.24 – 7.17 (m, 2H), 7.04 – 6.97 (m, 1H), 6.97 – 6.89 (m, 3H), 6.67 (d, J = 8.2 Hz, 1H), 4.13 (t, J = 5.9 Hz, 2H), 4.07 (t, J = 5.9 Hz, 2H), 2.90 (t, J = 7.3 Hz, 2H), 2.59 (t, J = 7.0 Hz, 2H), 2.29 – 2.18 (m, 4H), 0.88 (t, J = 7.1 Hz, 2H); ^{13}C NMR (126 MHz, $CDCl_3$): δ 170.4, 169.3, 162.3, 159.4, 139.1, 135.3, 131.4, 129.9, 115.5, 112.1, 110.8, 106.3, 103.37 (t, J = 22.7 Hz, 1C), 67.3, 66.3, 33.8, 30.2, 24.8, 24.6; HRMS (ESI): $[M+H]^+$ calcd. for $C_{28}H_{24}F_4N_2O_7$, 577.1520; found, 577.1592.

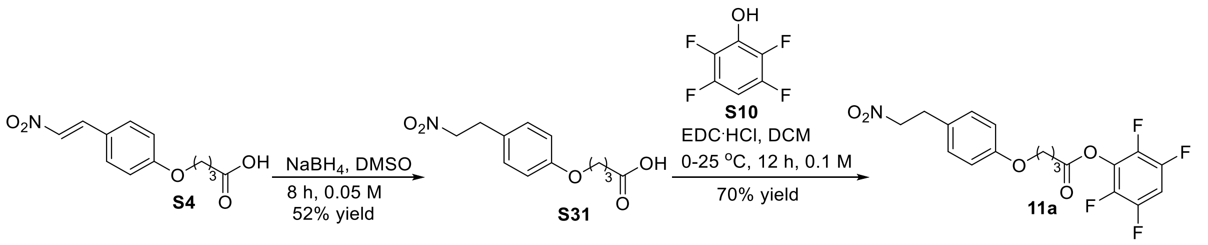

Supplementary Fig. 10. Synthesis of 2,3,5,6-tetrafluorophenyl 4-(4-(2-nitroethyl)phenoxy)butanoate **11a**.

Synthesis of 4-(4-(2-nitroethyl)phenoxy)butanoic acid (S31)

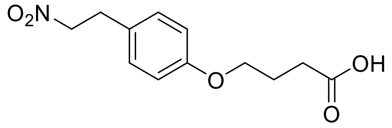

In a 50 ml round bottom flask, (E)-4-(4-(2-nitrovinyl)phenoxy)butanoic acid **S4** (300 mg, 1.19 mmol) was dissolved in DMSO (23.8 ml). To this solution, slowly sodium borohydride (90 mg, 2.38 mmol) was added under ice cold condition and stirred at 25 °C for 8 h. the progress of the reaction was monitored by TLC. After 8 h, the reaction mixture was concentrated in vacuum. This was followed by solvent-solvent extraction using 40% hexane:ethyl acetate and water. The organic fractions were combined, dried on anh. sodium sulfate, filtered, and concentrated on rotary evaporator. The purification of crude reaction mixture was performed by silica gel flash column chromatography (ethyl acetate:hexane, 60:40) to isolate 4-(4-(2-nitroethyl)phenoxy)butanoic acid **S31** (160 mg, 52% yield, crystalline yellow solid). TLC (ethyl acetate:n-hexane, 50:50 v/v): R_f = 0.52; ^{1}H NMR (500 MHz, $CDCl_3$): δ 7.11 (d, J = 8.6 Hz, 2H), 6.84 (d, J = 8.6 Hz, 2H), 4.56 (t, J = 7.4 Hz, 2H), 4.00 (t, J = 6.1 Hz, 2H), 3.25 (t, J = 7.4 Hz, 2H), 2.58 (t, J = 7.2 Hz, 2H), 2.11 (m, 2H); ^{13}C NMR (126 MHz, $CDCl_3$): δ 179.3, 158.2, 129.8, 127.9, 115.1, 76.7, 66.7, 32.8, 30.6, 24.5; HRMS (ESI): $[M+H]^+$ calcd. for $C_{12}H_{16}NO_5$, 254.1028; found, 254.1023.

Synthesis of 2,3,5,6-tetrafluorophenyl 4-(4-(2-nitroethyl)phenoxy)butanoate (11a).

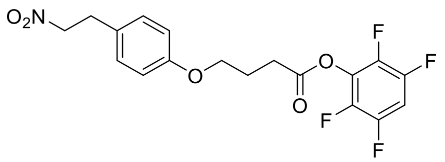

In a 10 ml round bottom flask, 4-(4-(2-nitroethyl)phenoxy)butanoic acid **S31** (100 mg, 0.39 mmol) and 1-(3-dimethylaminopropyl)-3-ethylcarbodiimide hydrochloride (149 mg, 0.78 mmol) were dried on high vacuum for 30 min. Next, the reagents were dissolved in anh. DCM (3.9 ml) under the presence of an inert atmosphere and ice-cold condition. Subsequently, the reaction mixture is allowed to achieve the ambient temperature. To this solution, 2,3,5,6-tetrafluorophenol (82.5 µl, 0.78 mmol) was added and stirred at 25 °C, under an inert atmosphere for 12 h. The progress of the reaction was followed by TLC. The purification of crude reaction mixture was performed by silica gel flash column chromatography (ethyl acetate:hexane, 25:65) to isolate 2,3,5,6-tetrafluorophenyl 4-(4-(2-nitroethyl)phenoxy)

butanoate **11a** (110 mg, 70% yield, crystalline yellow solid). TLC (ethyl acetate:n-hexane, 50:50 v/v): R_f = 0.70; ^{1}H NMR (500 MHz, $CDCl_3$): δ 7.13 (d, J = 8.6 Hz, 2H), 7.00 (m, 1H), 6.87 (d, J = 8.6 Hz, 2H), 4.57 (t, J = 7.4 Hz, 2H), 4.06 (t, J = 5.9 Hz, 2H), 3.26 (t, J = 7.4 Hz, 2H), 2.91 (t, J = 7.3 Hz, 2H), 2.25 (m, 2H); ^{13}C NMR (126 MHz, $CDCl_3$): δ 169.3, 158.2, 129.8, 128.1, 115.1, 103.4, 76.7, 66.4, 32.9, 30.2, 24.7; HRMS (ESI): $[M+Na]^+$ calcd. for $C_{18}H_{15}F_4NNaO_5$, 424.0784; found, 424.0779.

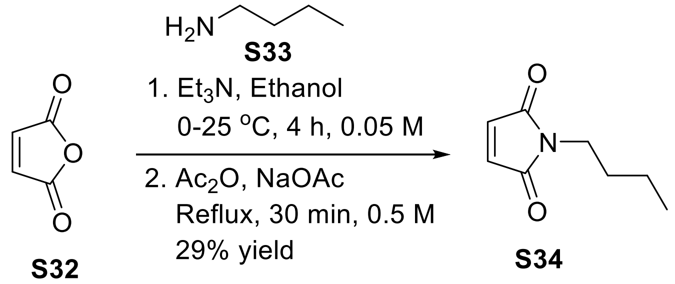

Supplementary Fig. 11. Synthesis of 1-butyl-1H-pyrrole-2,5-dione **S34**.

Synthesis of 1-butyl-1H-pyrrole-2,5-dione (S34)

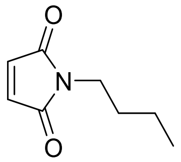

To a 250 ml round bottom flask containing maleic anhydride **S32** (784 mg, 8 mmol) in ethanol (80 ml), dropwise addition of n-Butylamine (948 µl, 9.6 mmol) and triethyl amine (2.24 ml, 16 mmol) in ethanol (80 ml) was done at ice cold condition followed by stirring for 4 h. The progress of the reaction was followed by TLC. The reaction mixture was concentrated to remove solvent and content were redissolve in acetic anhydride (6 ml) with sodium acetate (327 mg, 3.99 mmol). The reaction mixture was heated over 70 °C for 30 mins and allow to cool down followed by addition of water (10 ml). Subsequently extraction was carried out with ethyl acetate (30 ml × 3). The organic fractions were combined, dried on anh. sodium sulfate, filtered, and concentrated on rotary evaporator. The purification of crude reaction mixture was performed by silica gel flash column chromatography (ethyl acetate:hexane, 30:70) to isolate 1-butyl-1H-pyrrole-2,5-dione **S34** (120 mg, 29% yield, pale yellow solid). TLC (ethyl acetate:n-hexane, 50:50 v/v): R_f = 0.48; ^{1}H NMR (500 MHz, $CDCl_3$): δ 6.66 (s, 2H), 3.49 (t, J = 7.3 Hz, 2H), 1.54 (m, 2H), 1.27 (m, 2H), 0.90 (t, J = 7.4 Hz, 3H); ^{13}C NMR (126 MHz, $CDCl_3$): δ 171.0, 134.1, 37.7, 30.7, 20.0, 13.7; HRMS (ESI): $[M+H]^+$ calcd. for $C_8H_{12}NO_2$, 154.0868; found, 154.0863.

3.2 Derivatives of O-hydroxyl amine

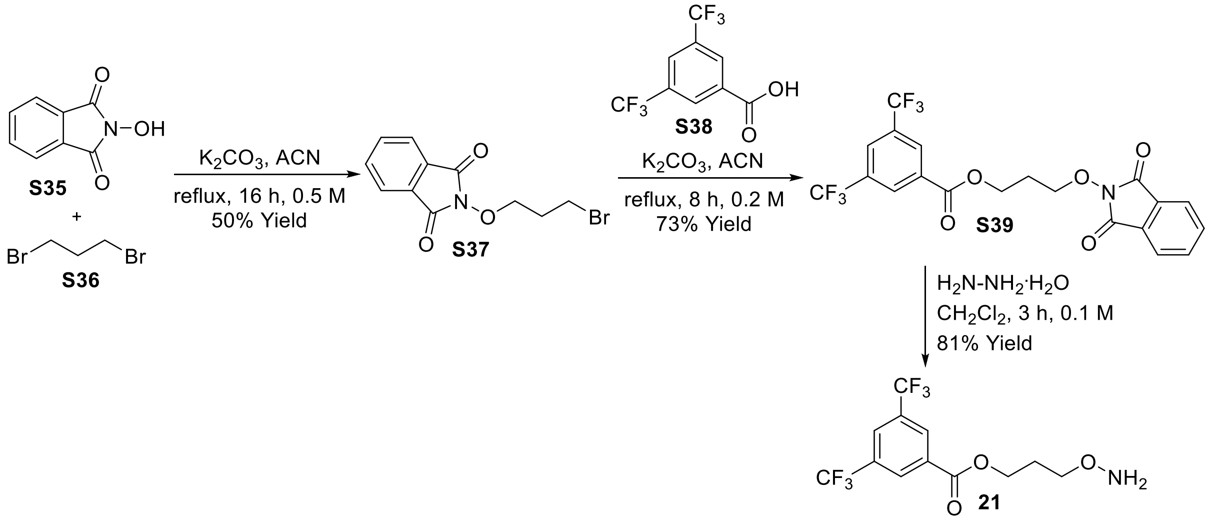

Supplementary Fig. 12. Synthesis of 1-butyl-1H-pyrrole-2,5-dione **9b**.

Synthesis of 2-(3-bromopropoxy)isoindoline-1,3-dione (S37)

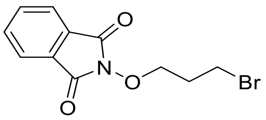

In a 250 ml round bottom flask charged with magnetic stir bar, N-hydroxyphthalimide **S35** (4.89 g, 30 mmol) and K_2CO_3 (8.29 g, 60 mmol) were dissolved in ACN (60 ml). Next, 1,3-dibromopropane **S36** (8.34 ml, 60 mmol) was added in the reaction mixture was reflux for 16 h. The reaction mixture was concentrated in vacuo followed by addition of 1 N NaOH solution and ethyl acetate for extraction. The organic layer was separated, dried over anh. sodium sulfate, filtered, and concentrated in vacuo. The purification of crude mixture was performed by flash column chromatography using ethyl acetate:hexane (3:97) to render **S37** (50% yield, white solid). TLC (ethyl acetate:n-hexane, 30:70 v/v): $R_f = 0.57$; ^{1}H NMR (400 MHz, $CDCl_3$): δ 7.85-7.82 (m, 2H), 7.77-7.74 (m, 2H), 4.36 (t, J = 5.8 Hz, 2H), 3.70 (t, J = 6.5 Hz, 2H), 2.30-2.28 (m, 2H); ^{13}C NMR (101 MHz, $CDCl_3$): δ 163.7, 134.7, 129.0, 123.7, 76.2, 31.6, 29.4; HRMS (ESI): $[M+H]^+$ calcd. for $C_{11}H_{11}{}^{81}BrNO_3$, 286.0; found, 285.9.

Synthesis of 3-((1,3-dioxoisoindolin-2-yl)oxy)propyl 3,5-bis(trifluoromethyl)benzoate S39

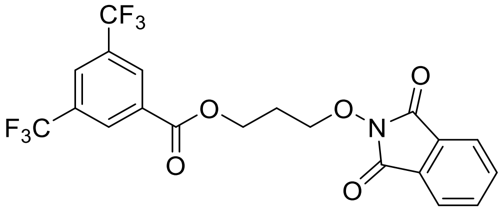

In a 25 ml round bottom flask, 3,5-bis(trifluoromethyl)benzoic acid S40 (258 mg, 1 mmol), 2-(3- bromopropoxy)isoindoline-1,3-dione **S38** (312 mg, 1.1 mmol) and K_2CO_3 (414 mg, 3 mmol) were dissolved in acetonitrile (5 ml). The reaction mixture was refluxed and the progress of the reaction was followed by TLC. After 8 h, the reaction mixture was concentrated and purification by silica gel flash column chromatography (ethyl acetate:n-hexane, 2:98) led to the

isolation of 3-((1,3-dioxoisoindolin-2-yl)oxy)propyl 3,5-bis(trifluoromethyl)benzoate **S39** (335 mg, 73% yield, white solid). TLC (ethyl acetate:n-hexane, 30:70 v/v): R_f = 0.67; ^{1}H NMR (400 MHz, $CDCl_3$): δ 8.51 (s, 2H), 8.05 (s, 1H), 7.91-7.81 (m, 2H), 7.80-7.72 (m, 2H), 4.72 (t, J = 6.3 Hz, 2H), 4.41 (t, J = 6.0 Hz, 2H), 2.39-2.21 (m, 2H); 13C NMR (101 MHz, $CDCl_3$): δ 164.0, 163.7, 134.7, 132.5, 132.3 (q, J = 34.1 Hz, 2C), 130.1-129.8 (m, 2C), 129.0, 126.6-126.6 (m, 1C), 123.7, 123.0 (q, J = 272.8 Hz, 2C), 74.9, 62.7, 27.8; ^{19}F NMR (376 MHz, $CDCl_3$) δ -62.94 (TFA was used as an internal standard, -75.70 ppm); HRMS (ESI): $[M+H]^+$ calcd. for $C_{20}H_{14}F_6NO_5$, 462.0775; found, 462.0775.

Synthesis of 3-(aminooxy)propyl 3,5-bis(trifluoromethyl)benzoate (21)

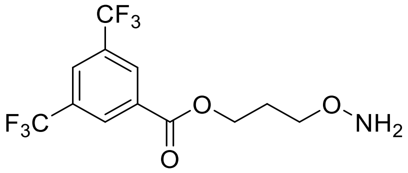

In a 5 ml round bottom flask, 3-((1,3-dioxoisoindolin-2-yl)oxy)propyl 3,5-bis(trifluoromethyl)benzoate **S39** (138 mg, 0.3 mmol) in DCM (3 ml) and hydrazine monohydrate (80%, 37 µl, 0.75 mmol) were stirred at room temperature. The progress of the reaction was followed by TLC. After 3 h, the reaction mixture was filtered and concentration of the filtrate in vacuo led to the isolation of 3-(aminooxy)propyl 3,5-bis(trifluoromethyl)benzoate **21** (80 mg, 81% yield, white solid). TLC (ethyl acetate:n-hexane, 30:70 v/v): R_f = 0.33; ^{1}H NMR (400 MHz, $CDCl_3$): δ 8.48 (s, 2H), 8.07 (s, 1H), 5.43 (bs, 2H), 4.50 (t, J = 6.5 Hz, 2H), 3.83 (t, J = 6.1 Hz, 2H), 2.19-2.04 (m, 2H); ^{13}C NMR (101 MHz, $CDCl_3$): δ 164.1, 132.6, 132.4 (q, J = 33.9 Hz, 2C), 130.0-129.7 (m, 2C), 126.6-126.3 (m, 1C), 123.02 (q, J = 273.0 Hz, 2C), 72.2, 63.6, 27.9; ^{19}F NMR (376 MHz, $CDCl_3$) δ -62.54 (TFA was used as an internal standard, -75.70 ppm); HRMS (ESI): $[M+H]^+$ calcd. for $C_{12}H_{12}F_6NO_3$, 332.0721; found, 332.0699.

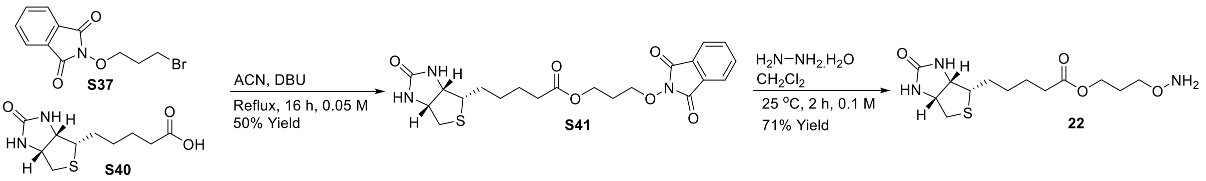

Supplementary Fig. 13. Synthesis of 7-((3-(aminooxy)propyl)thio)-4-methyl-2H-chromen-2-one **22**.

Synthesis of 3-((1,3-dioxoisoindolin-2-yl)oxy)propyl 5-(2-oxohexahydro-1H-thieno[3,4-d]imidazol-4-yl)pentanoate (S41)

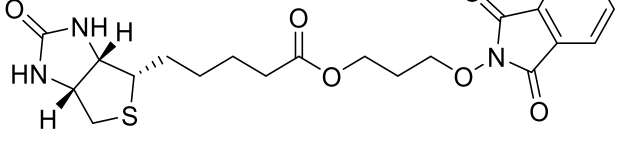

In a 5 ml round bottom flask, biotin **S40** (244 mg, 1 mmol), 2-(3-bromopropoxy)isoindoline-1,3- dione **S37** (568 mg, 2 mmol), and DBU (304 µl, 2 mmol) were dissolved in acetonitrile (20 ml). The reaction mixture was refluxed and the progress of the reaction was monitored by

TLC. After 16 h, the reaction mixture was concentrated in vacuum. This was followed by solvent-solvent extraction using ethyl acetate and water. The organic fractions were combined, dried on anh. sodium sulfate, filtered, and concentrated on rotary evaporator. The purification of crude reaction mixture was performed by silica gel flash column chromatography (MeOH:DCM, 0.5-5%) to isolate 3-((1,3-dioxoisoindolin-2-yl)oxy)propyl 5-(2-oxohexahydro-1H-thieno[3,4-d]imidazol-4- yl)pentanoate **S41** (224 mg, 50% yield, white solid). TLC (MeOH:DCM 5:95 v/v): R_f = 0.33; ^{1}H NMR (500 MHz, $CDCl_3$): δ 7.89-7.82 (m, 2H), 7.80-7.73 (m, 2H), 5.96 (s, 1H), 5.48 (s, 1H), 4.56-4.47 (m, 1H), 4.38-4.26 (m, 5H), 3.23-3.13 (m, 1H), 2.91 (dd, J = 12.8, 5.0 Hz, 1H), 2.74 (d, J = 12.8 Hz, 1H), 2.36 (t, J = 7.4 Hz, 2H), 2.16-2.09 (m, 2H), 1.80-1.60 (m, 4H), 1.54-1.39 (m, 2H); ^{13}C NMR (126 MHz, $CDCl_3$): δ 173.7, 163.8, 163.7, 134.7, 129.0, 123.7, 75.1, 62.0, 60.7, 60.2, 55.5, 40.7, 34.0, 28.4, 28.3, 27.8, 24.9; HRMS (ESI): $[M+H]^+$ calcd. for $C_{21}H_{26}N_3O_6S$, 448.1542; found, 448.1548.

Synthesis of 3-(aminooxy)propyl 5-(2-oxohexahydro-1H-thieno[3,4-d]imidazol-4-yl)pentanoate (22)

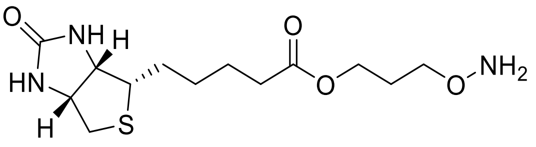

This compound was synthesized according to the procedure for synthesis of **21**. (yield 71%, white solid). TLC (MeOH:DCM 5:95 v/v): R_f = 0.21; ^{1}H NMR (500 MHz, D_2O): δ 4.63 (dd, J = 7.9, 4.9 Hz, 1H), 4.45 (dd, J = 7.9, 4.5 Hz, 1H), 4.21 (t, J = 6.3 Hz, 2H), 3.90 (t, J = 6.2 Hz, 2H), S40 3.45-3.27 (m, 1H), 3.02 (dd, J = 13.1, 5.0 Hz, 1H), 2.80 (d, J = 13.0 Hz, 1H), 2.44 (t, J = 7.3 Hz, 2H), 2.08-1.94 (m, 2H), 1.84-1.55 (m, 4H), 1.53-1.37 (m, 2H); ^{13}C NMR (126 MHz, D_2O): δ 176.9, 165.3, 72.4, 62.1, 62.0, 60.3, 55.3, 39.7, 33.6, 27.9, 27.6, 26.7, 24.1; HRMS (ESI): $[M+H]^+$ calcd. for $C_{13}H_{24}N_3O_4S$, 318.1488; found, 318.1467.

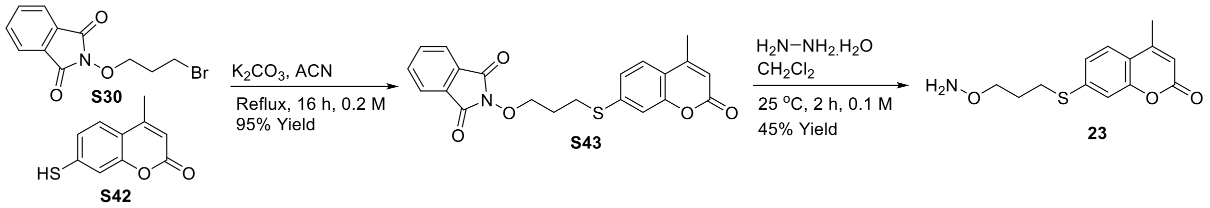

Supplementary Fig. 14. Synthesis of 7-((3-(aminooxy)propyl)thio)-4-methyl-2H-chromen-2-one **23**.

Synthesis of 2-(3-((4-methyl-2-oxo-2H-chromen-7-yl)thio)propoxy)isoindoline-1,3-dione (S43)

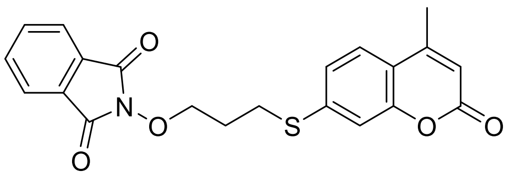

In a 25 ml round bottom flask, 7-mercapto-4-methylcoumarin **S42** (192 mg, 1 mmol), K_2CO_3 (276 mg, 2 mmol), and 2-(3-bromopropoxy)isoindoline-1,3-dione **S30** (568 mg, 2 mmol) were dissolved in degassed acetonitrile (5 ml) and refluxed for 16 h. The reaction mixture was concentrated in vacuo and purified by silica gel flash column chromatography using ethyl

acetate:hexane (7:3) to give **S43** (375 mg, 95% yield, white solid). TLC (ethyl acetate:n-hexane 50:50 v/v): R_f = 0.37; ^{1}H NMR (400 MHz, $CDCl_3$): δ 7.90-7.81 (m, 2H), 7.80-7.73 (m, 2H), 7.48 (d, J = 8.2 Hz, 1H), 7.26-7.20 (m, 2H), 6.22 (d, J = 0.8 Hz, 1H), 4.36 (t, J = 5.8 Hz, 2H), 3.35 (t, J = 7.1 Hz, 2H), 2.41 (d, J = 0.9 Hz, 3H), 2.23-2.08 (m, 2H); ^{13}C NMR (101 MHz, $CDCl_3$): δ 163.8, 160.7, 154.0, 152.3, 142.6, 134.7, 129.0, 124.9, 123.8, 123.4, 117.5, 114.8, 114.1, 76.6, 28.7, 27.8, 18.7; HRMS (ESI): $[M+H]^+$ calcd. for $C_{21}H_{18}NO_5S$, 396.0906; found, 396.0925.

Synthesis of 7-((3-(aminooxy)propyl)thio)-4-methyl-2H-chromen-2-one (23)

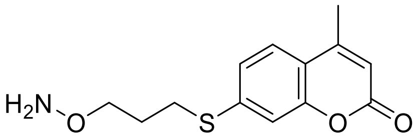

2-(3-((4-methyl-2-oxo-2H-chromen-7-yl)thio)propoxy)isoindoline-1,3-dione **S43** (237 mg, 0.6 mmol) was dissolved in CH_2Cl_2 (12 ml) in a 50 ml round bottom flask. To this solution, hydrazine monohydrate (80%, 29 µl, 0.6 mmol) was added and stirred at 25 °C for 3 h. The reaction mixture was filtered and the filtrate was concentrated. The purification of crude reaction mixture was performed by reverse phase preparative HPLC to isolate **23** (76 mg, 45% yield, pale green viscous liquid). TLC (ethyl acetate:n-hexane 50:50 v/v): R_f = 0.60; ^{1}H NMR (400 MHz, $CDCl_3$): δ 7.46 (d, J = 8.3 Hz, 1H), 7.23-7.13 (m, 2H), 6.18 (d, J = 0.9 Hz, 1H), 3.79 (t, J = 5.9 Hz, 2H), 3.07 (t, S41 J = 7.3 Hz, 2H), 2.40 (d, J = 0.8 Hz, 3H), 2.02-1.90 (m, 2H); ^{13}C NMR (101 MHz, $CDCl_3$): δ 160.7, 154.0, 152.3, 143.3, 124.7, 123.1, 117.2, 114.1, 113.9, 73.9, 29.0, 27.8, 18.6; HRMS (ESI): $[M+H]^+$ calcd. for $C_{13}H_{16}NO_3S$, 266.0851; found, 266.0841.

The compounds **35a** was synthesized according to the reported procedure.[17]

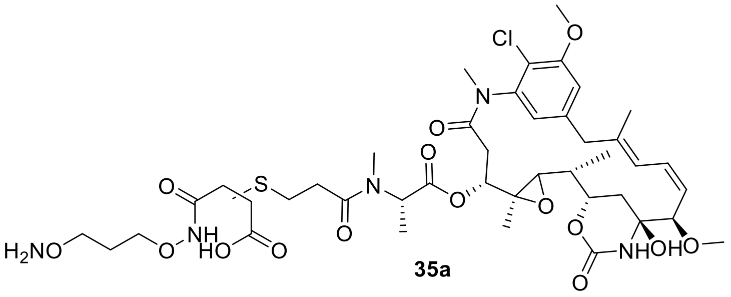

35a

4. Reference table: characterization data for protein labeling

Figure	Related data in supplementary information	Page(s)
Fig. 2a	Sec. 5.1, Supplementary Figs. 15-26, Supplementary Table 1	S41-S47
Fig. 2b	Sec. 5.1, Supplementary Figs. 27-28, Supplementary Table 2	S48-S50
Fig. 2c	Sec. 5.1, Supplementary Figs. 29-37, Supplementary Table 3	S51-S55
Fig. 3a<br>Fig. 3b	Sec. 5.2, Supplementary Figs. 38-43<br>Sec. 3.1, Supplementary Figs. 1-10	S56-S57<br>S18-S34
Fig. 4	Sec. 5.3, Supplementary Figs. 44-50, Supplementary Table 4<br>Sec. 8.8a, Supplementary Fig. 99-101<br>Sec. 8.8b, Supplementary Fig. 102-106	S58-S67<br>S118-S121<br>S122-S124
Fig. 5a	Sec. 6.4 and 6.5, Supplementary Fig. 55 and 56	S72-S73
Fig. 5b	Sec. 6.8, Supplementary Figs. 66-70	S76-S85
Fig. 5c	Sec. 6.7, Supplementary Fig. S65, Supplementary Table 7	S75-S76
Fig. 5d-5f	Sec. 8.1, Supplementary Fig. 91 and 92	S105-S106
Fig. 6a-6b	Sec. 6.9 and 6.10, Supplementary Figs. 71 –77	S86-S92
Fig. 7a	Sec. 6.12, Supplementary Fig. 85 and 86	S98-S100
Fig. 7b	Sec. 6.13, Supplementary Fig. 87	S101
Fig. 8	Sec. 7, Supplementary Fig. 88-90	S102-S105
Supplementary Figs. 12-14	Sec. 3.2	S36-S38
Supplementary Fig. 51<br>Supplementary Table 5	Sec. 6.1	S68
Supplementary Fig. 52	Sec. 6.1.1	S69-S70
Supplementary Fig. 53	Sec. 6.2	S70-S71
Supplementary Fig. 54	Sec. 6.3	S71
Supplementary Figs. 57-64<br>Supplementary Table 6	Sec. 6.6	S73-S75
Supplementary Figs. 78-84	Sec. 6.11	S92-S97
Supplementary Fig. 96	Sec. 8.5	S116
Supplementary Fig. 97	Sec. 8.6	S116
Supplementary Fig. 98	Sec. 8.7	S117
Supplementary Figs. 114-188	Sec. 10	S137-S174
Supplementary Fig. 93<br>Supplementary Table 8	Sec. 8.2	S106-S111
Supplementary Fig. 111-113	Sec. 8.11	S131-S134
Supplementary Fig. 94<br>Supplementary Table 9	Sec. 8.3	S112-S113
Supplementary Fig. 108-109	Sec. 8.9a & Sec 8.9b	S128-S129
Supplementary Fig. 95, Supplementary Table 10	Sec. 8.4	S114-S115

5. Supplementary Notes 2: small molecules and peptides

5.1 Small molecule characterization

Supplementary Table 1. Screening of Cys-selective electrophiles (F_C) for C-S bond formation

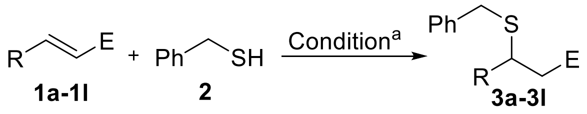

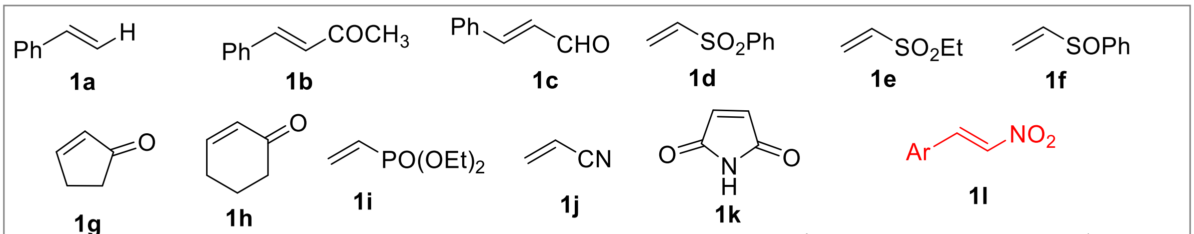

[a] DMSO-d_6:D_2O (7:3), 10 mM, 10 min.[b]% Conversion based on ^{1}H-NMR

Entry	Electrophile (E)	Product (3a-3l)	% Conversion[b]
1	**1a**	**3a**	0
2	**1b**	**3b**	0
3	**1c**	**3c**	0
4	**1d**	**3d**	>99
5	**1e**	**3e**	60
6	**1f**	**3f**	0
7	**1g**	**3g**	0
8	**1h**	**3h**	0
9	**1i**	**3i**	0
10	**1j**	**3j**	83
11	**1k**	**3k**	>99
**12**	**1l**	**3l**	**>99**

[b]% Conversions by ^{1}H NMR.

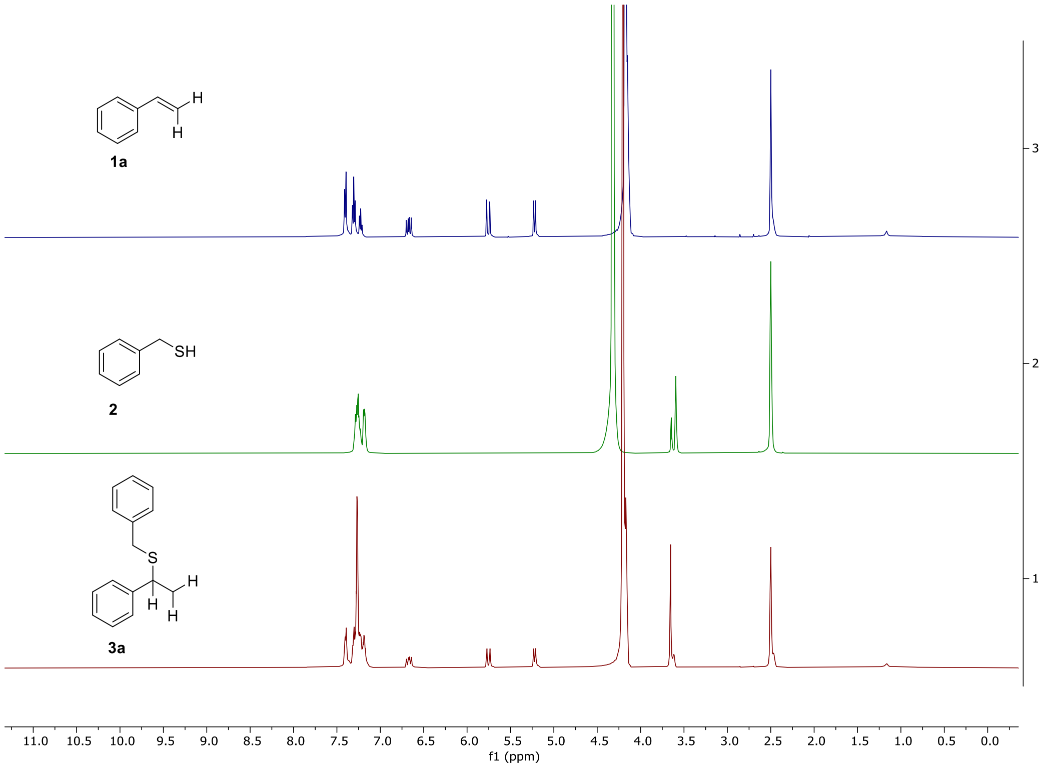

Supplementary Fig. 15. Screening of electrophile (**1a**) for identification of F_C. ^{1}H-NMR (DMSO-d_6:D_2O, 7:3) of electrophile (**1a**), benzyl mercaptan (**2**), and (**1a**+**2**) to form the adduct **3a** (0%, 10 min).

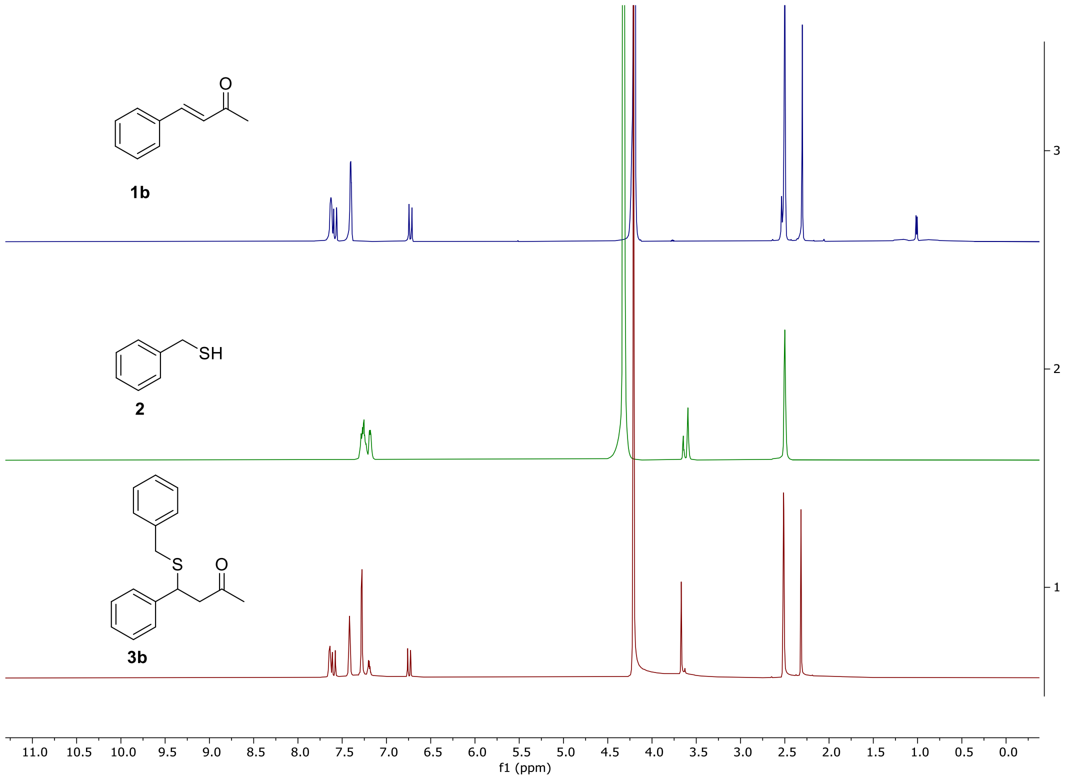

Supplementary Fig. 16. Screening of electrophile (**1b**) for identification of F_C. ^{1}H-NMR (DMSO-d_6:D_2O, 7:3) of electrophile (**1b**), benzyl mercaptan (**2**), and (**1b**+**2**) to form the adduct **3b** (0%, 10 min).

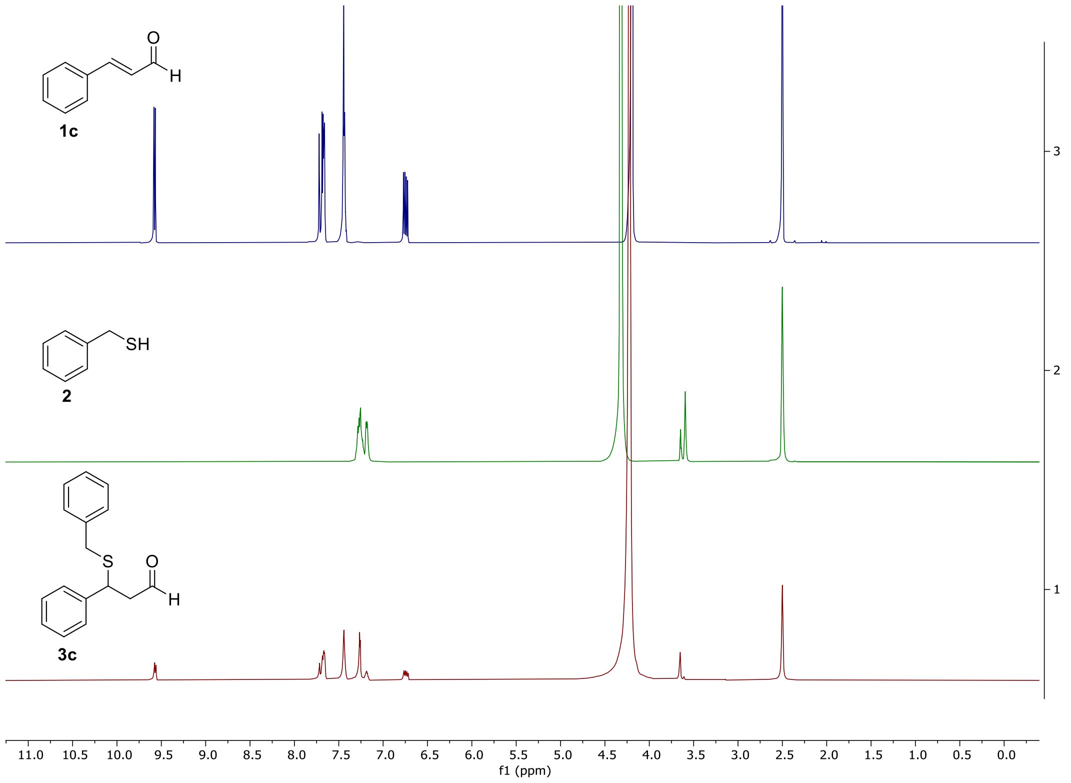

Supplementary Fig. 17. Screening of electrophile (**1c**) for identification of F_C. ^{1}H-NMR (DMSO-d_6:D_2O, 7:3) of electrophile (**1c**), benzyl mercaptan (**2**), and (**1c**+**2**) to form the adduct **3c** (0%, 10 min).

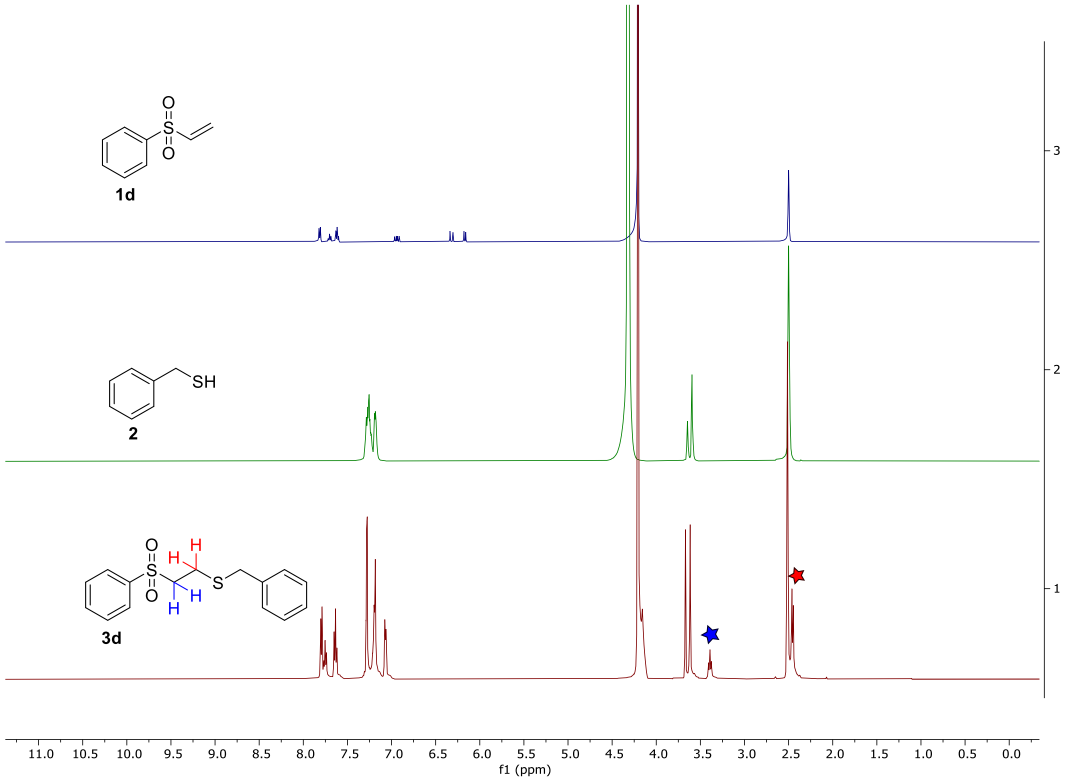

Supplementary Fig. 18. Screening of electrophile (**1d**) for identification of F_C. ^{1}H-NMR (DMSO-d_6:D_2O, 7:3) of electrophile (**1d**), benzyl mercaptan (**2**), and (**1d**+**2**) to form the adduct **3d** (>99%, 10 min).

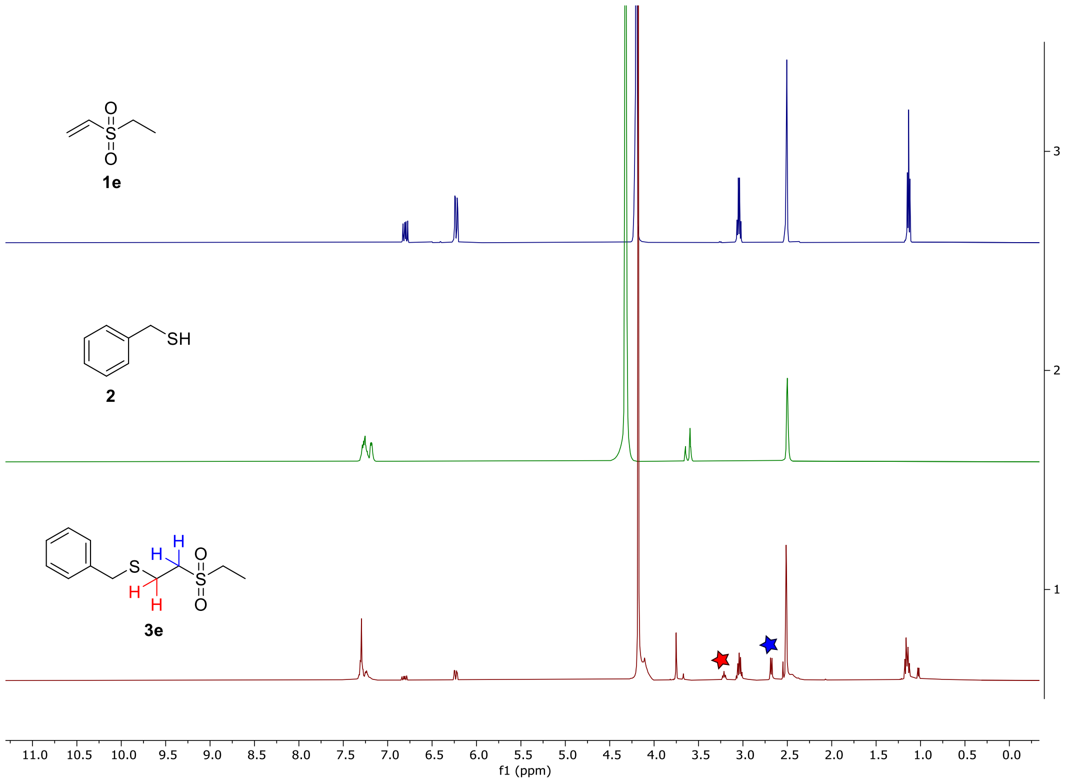

Supplementary Fig. 19. Screening of electrophile (**1e**) for identification of F_C. ^{1}H-NMR (DMSO-d_6:D_2O, 7:3) of electrophile (**1e**), benzyl mercaptan (**2**), and (**1e**+**2**) to form the adduct **3e** (60%, 10 min).

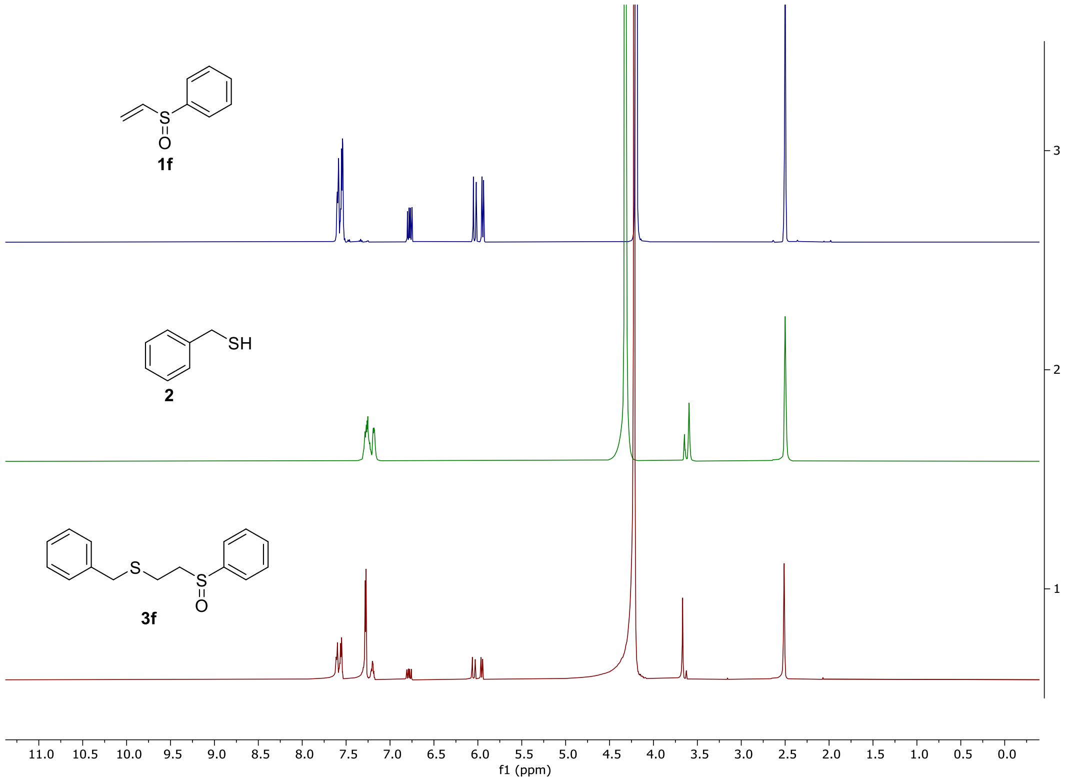

Supplementary Fig. 20. Screening of electrophile (**1f**) for identification of F_C. ^{1}H-NMR (DMSO-d_6:D_2O, 7:3) of electrophile (**1f**), benzyl mercaptan (**2**), and (**1f**+**2**) to form the adduct **3f** (0%, 10 min).

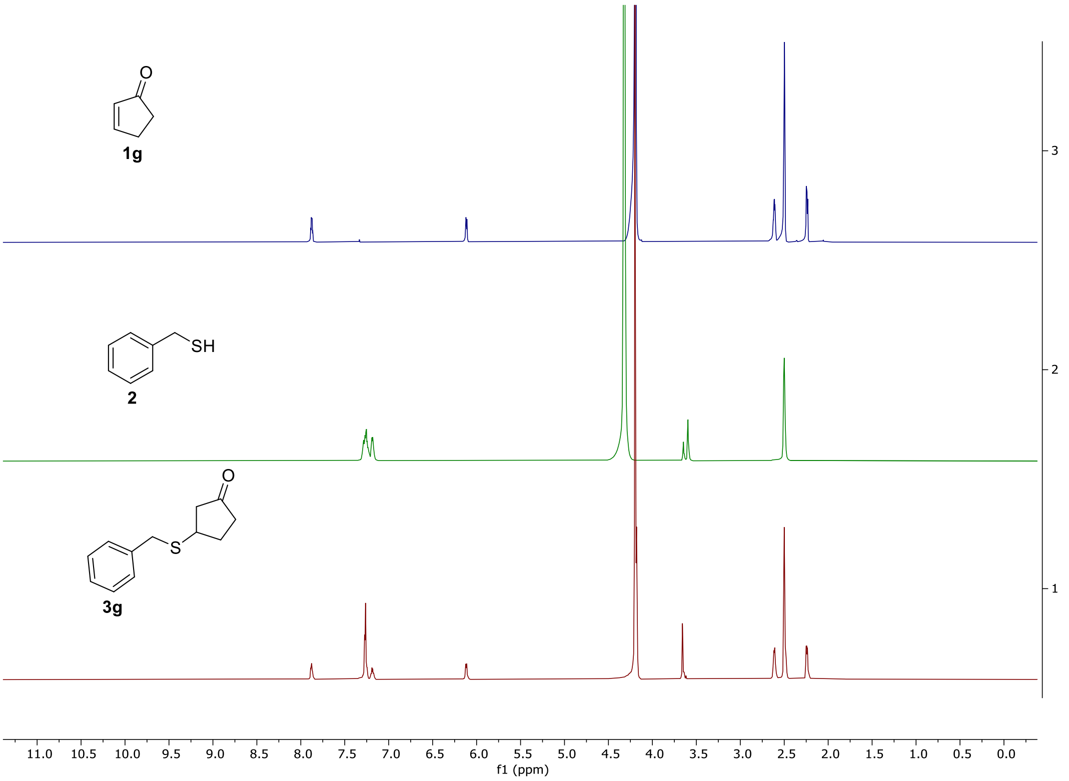

Supplementary Fig. 21. Screening of electrophile (**1g**) for identification of F_C. ^{1}H-NMR (DMSO-d_6:D_2O, 7:3) of electrophile (**1g**), benzyl mercaptan (**2**), and (**1g**+**2**) to form the adduct **3g** (0%, 10 min).

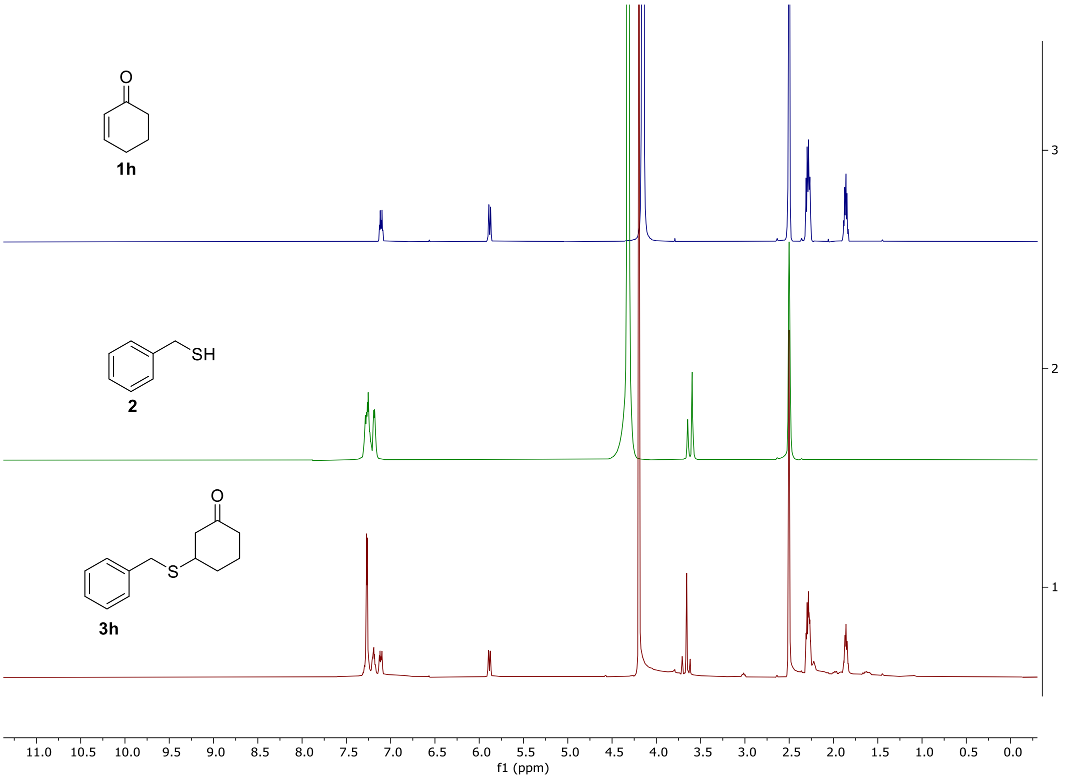

Supplementary Fig. 22. Screening of electrophile (**1h**) for identification of F_C. ^{1}H-NMR (DMSO-d_6:D_2O, 7:3) of electrophile (**1h**), benzyl mercaptan (**2**), and (**1h**+**2**) to form the adduct **3h** (0%, 10 min).

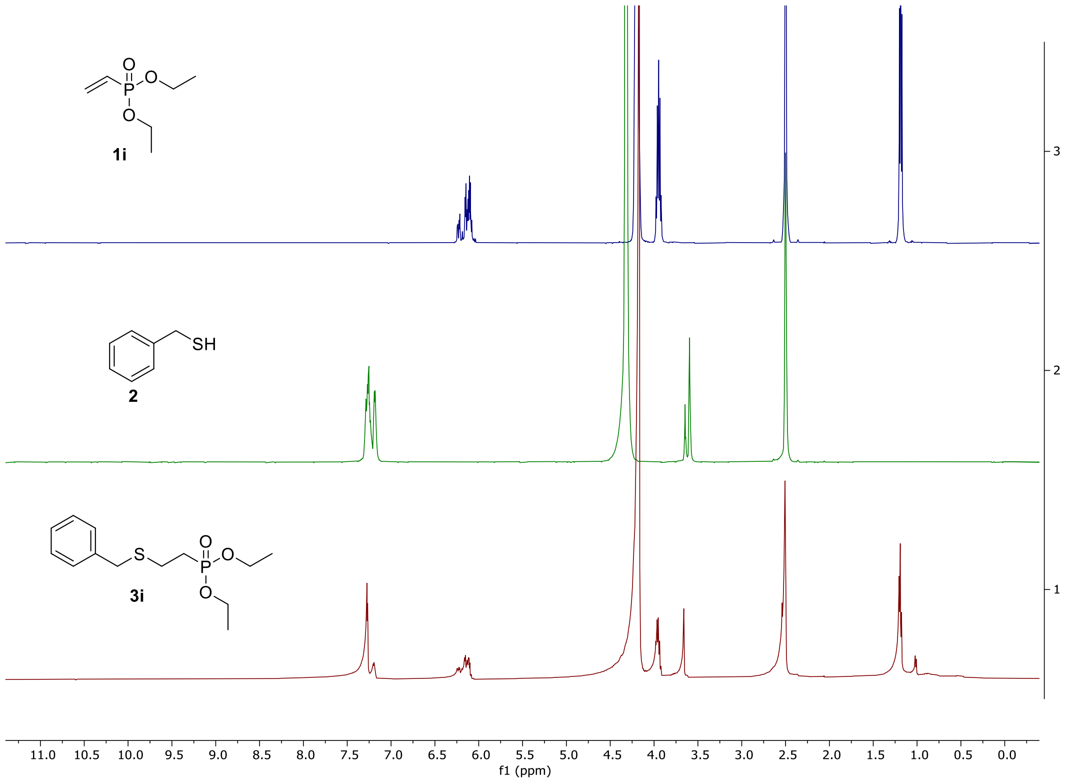

Supplementary Fig. 23. Screening of electrophile (**1i**) for identification of $F_{C.}$ ^{1}H-NMR (DMSO-d_6:D_2O, 7:3) of electrophile (**1i**), benzyl mercaptan (**2**), and (**1i**+**2**) to form the adduct **3i** (0%, 10 min).

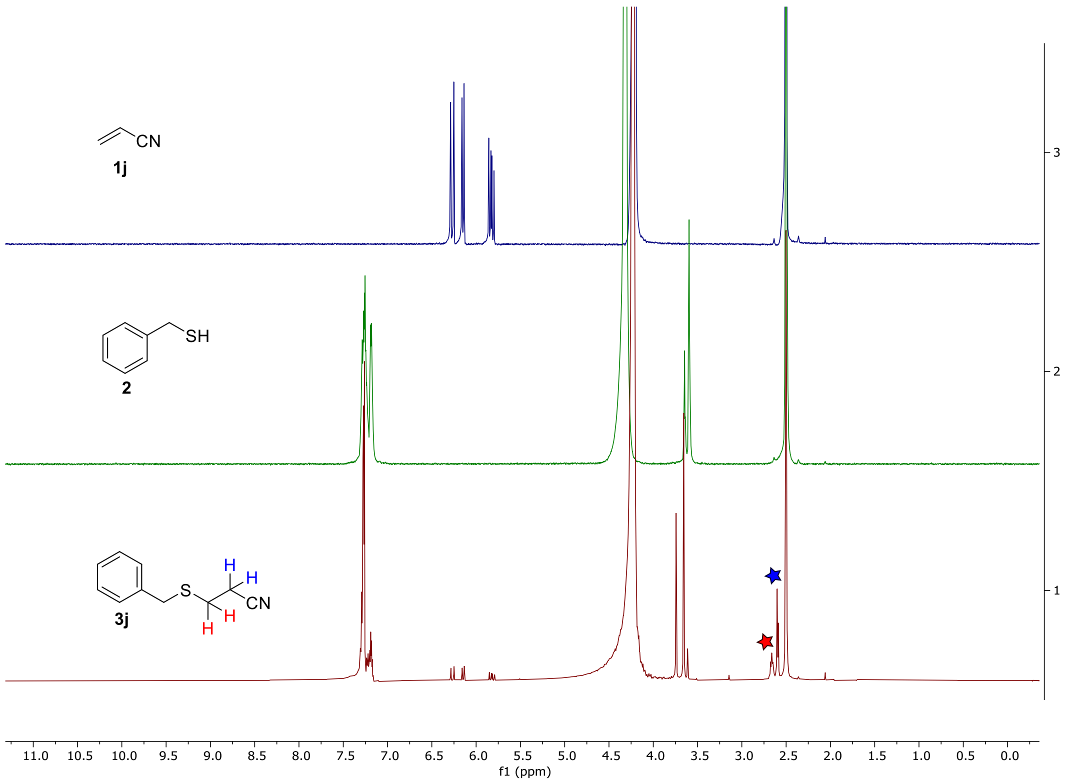

Supplementary Fig. 24. Screening of electrophile (**1j**) for identification of $F_{C.}$ ^{1}H-NMR (DMSO-d_6:D_2O, 7:3) of electrophile (**1j**), benzyl mercaptan (**2**), and (**1j**+**2**) to form the adduct **3j** (83%, 10 min).

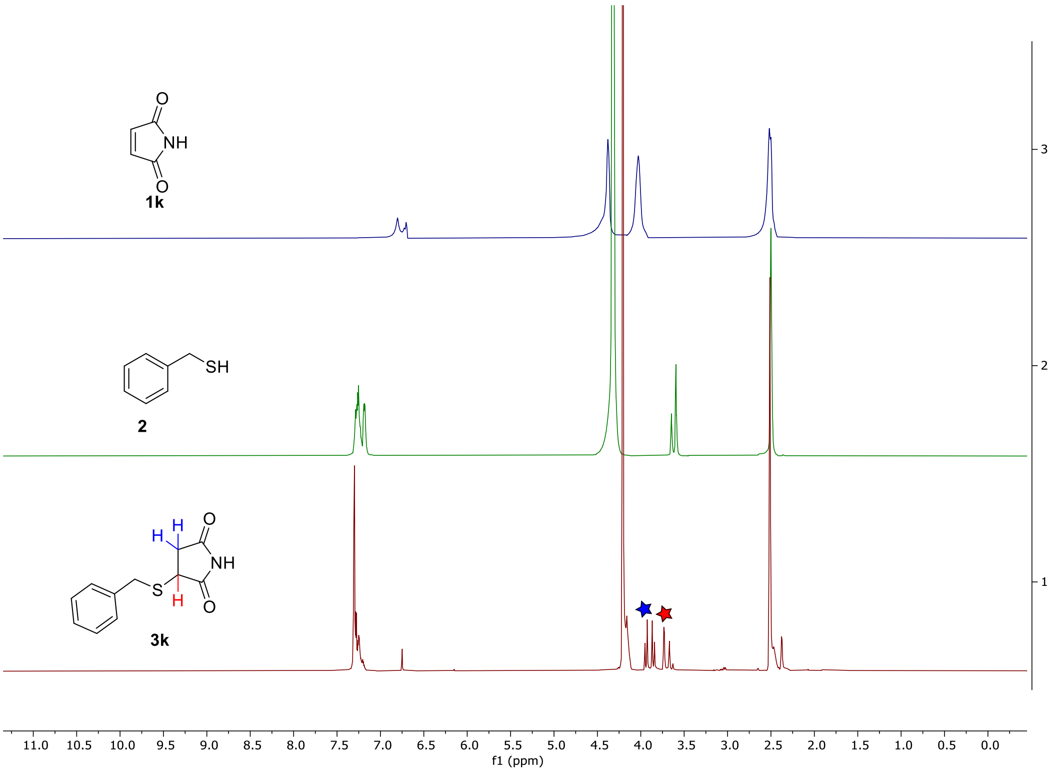

Supplementary Fig. 25. Screening of electrophile (**1k**) for identification of F_C. ^{1}H-NMR (DMSO-d_6:D_2O, 7:3) of electrophile (**1k**), benzyl mercaptan (**2**), and (**1k**+**2**) to form the adduct **3k** (>99%, 10 min).

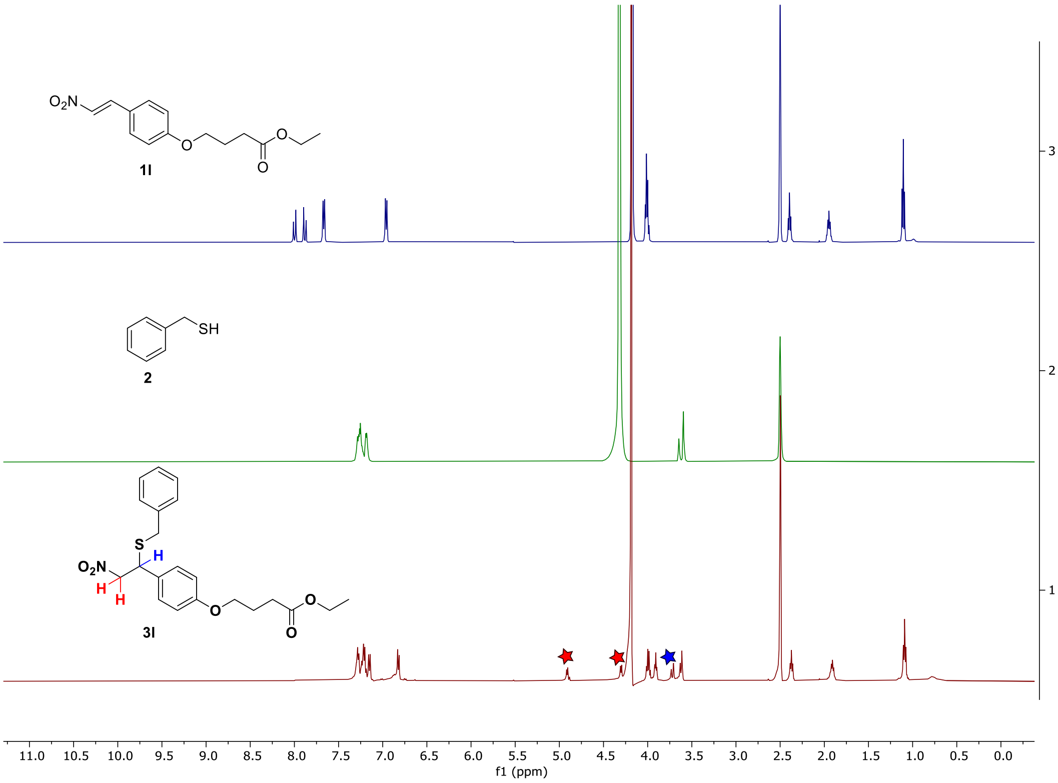

Supplementary Fig. 26. Screening of electrophile (**1l**) for identification of F_C. ^{1}H-NMR (DMSO-d_6:D_2O, 7:3) of electrophile (**1l**), benzyl mercaptan (**2**), and (**1l**+**2**) to form the adduct **3l** (>99%, 10 min).

Supplementary Table 2. Chemoselectivity of nitroolefin (Fc) with cysteine.

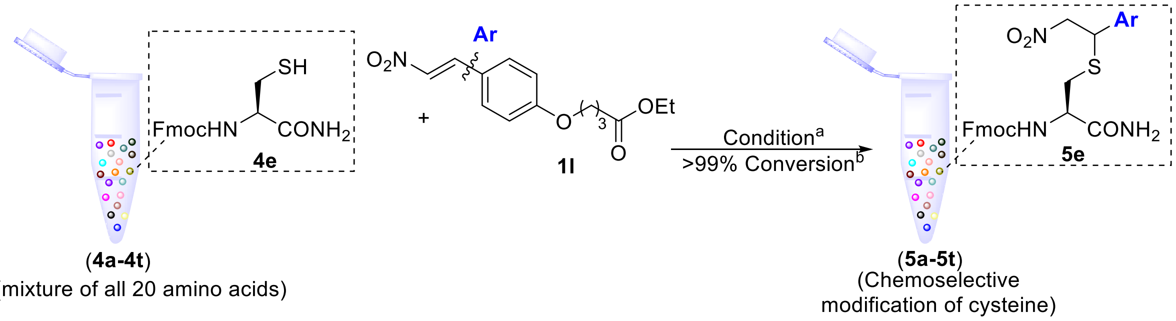

Entry	Fmoc-AA-NH2	Product (5a-5t)	%Conversion[b]
1	Fmoc-Ala-NH2 (**4a**)	**5a**	0
2	Fmoc-Arg-NH2 (**4b**)	**5b**	0
3	Fmoc-Asn-NH2 (**4c**)	**5c**	0
4	Fmoc-Asp-NH2 (**4d**)	**5d**	0
**5**	**Fmoc-Cys-NH2 (4e)**	**5e**	**>99**
6	Fmoc-Glu-NH2 (**4f**)	**5f**	0
7	Fmoc-Gln-NH2 (**4g**)	**5g**	0
8	Fmoc-Gly-NH2 (**4h**)	**5h**	0
9	Fmoc-His-NH2 (**4i**)	**5i**	0
10	Fmoc-Ile-NH2 (**4j**)	**5j**	0
11	Fmoc-Leu-NH2 (**4k**)	**5k**	0
12	Fmoc-Lys-NH2 (**4l**)	**5l**	0
13	Fmoc-Met-NH2 (**4m**)	**5m**	0
14	Fmoc-Phe-NH2 (**4n**)	**5n**	0
15	Fmoc-Pro-NH2 (**4o**)	**5o**	0
16	Fmoc-Ser-NH2 (**4p**)	**5p**	0
17	Fmoc-Thr-NH2 (**4q**)	**5q**	0
18	Fmoc-Trp-NH2 (**4r**)	**5r**	0
19	Fmoc-Tyr-NH2 (**4s**)	**5s**	0
20	Fmoc-Val-NH2 (**4t**)	**5t**	0

[a] 30% ACN, NaP (pH 7.0, 50 mM), 500 µM, 25 ˚C, 30 min. [b] % Conversions by LC-ESI-MS.

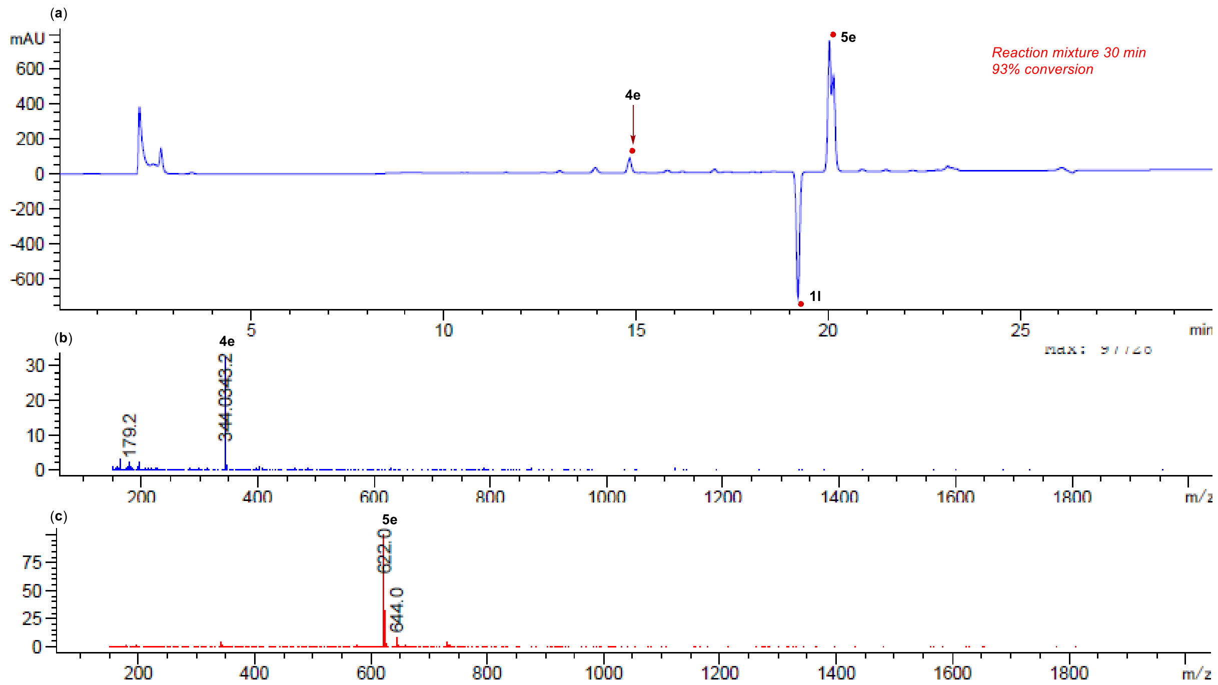

Supplementary Fig. 27. Chemoselective labeling of Fmoc-Cys-NH_2 (**4e**) with nitroolefin (**1l**) monitored by LC-ESI-MS. (a) LC profile for Chemoselective labeling of Fmoc-Cys-NH_2 (**4e**, 1 equiv.) enabled by reagent (**1l**, 1 equiv.) resulted into adduct (**5e**, 93%**)** after 30 min. (b) ESI-MS data for Fmoc-Cys-NH_2 (**4e**). (c) ESI-MS data of Cys adduct with **1l** (**5e**).

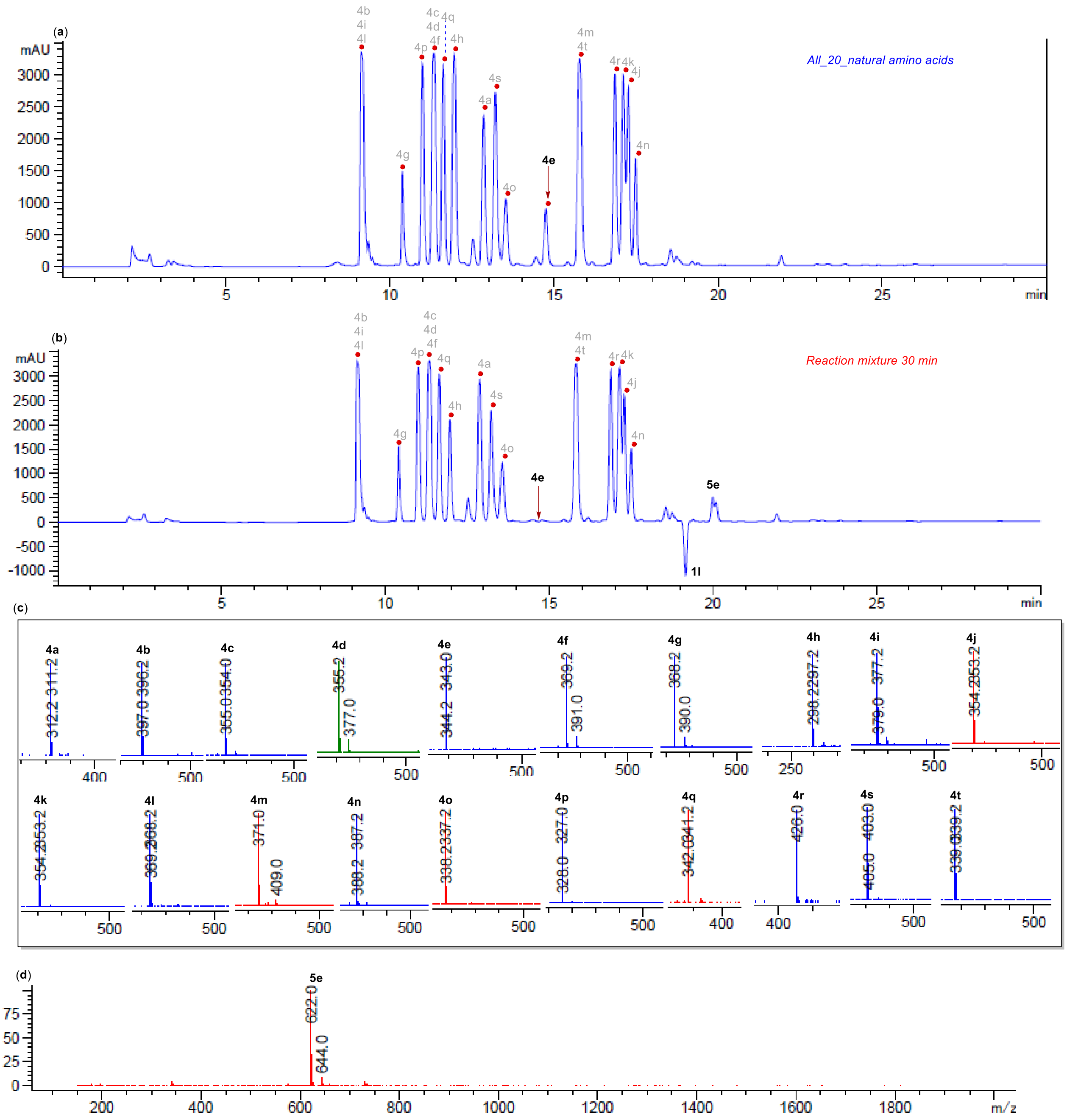

Supplementary Fig. 28. Chemoselective labeling of Fmoc-Cys-NH_2 (**4e**) in presence of all twenty natural amino acid with nitroolefin (**1l**) monitored by LC-ESI-MS. (a) LC profile for all the twenty natural amino acids (**4a-4t**). (b) LC profile for Chemoselective labeling of Fmoc-Cys-NH_2 (**4e**, 1 equiv.) enabled by reagent (**1l**, 1 equiv.) in presence of all twenty natural amino acid resulted into adduct (**5e**, >99%) after 30 min. (c) ESI-MS data for all the twenty natural amino acids (**4a-4t**). (d) ESI-MS data of Cys adduct with **1l** (**5e**).

Supplementary Table 3. C-S bond dissociation of benzylmercaptan-nitrolefin adduct (3l).

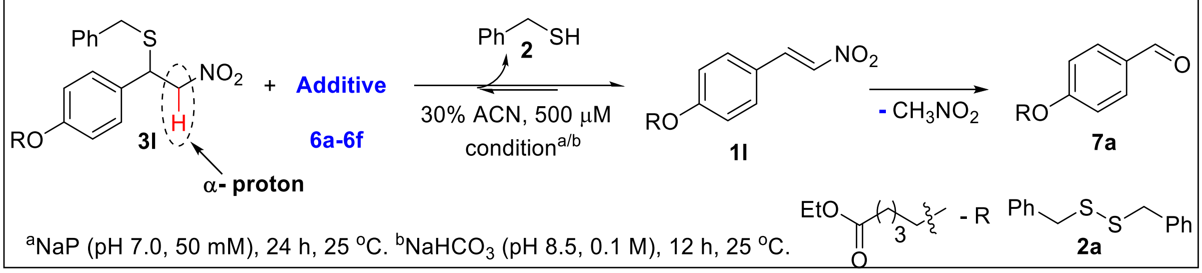

[a]NaP (pH 7.0, 50 mM), 24 h, 25 °C. [b]NaHCO3 (pH 8.5, 0.1 M), 12 h, 25 °C.

Entry	Additive	% Conversion (1l)[b]	% Conversion (7a)[b]
1	None	27	0
2	DABCO (**6a**)	33	0
3	DBU (**6b**)	40	14
4	DIPEA (**6c**)	42	22
5	Tetramethylguanidine (**6d**)	33	0
6	Triethylamine (**6e**)	40	18
7	Urea (**6f**)	31	0
8[c]	0.1 M $NaHCO_3$	44	55

[c] 30% ACN, $NaHCO_3$ (pH 8.5, 0.1 M), 25 °C, 500 µm, 12 h. [d] % Conversions by LC-ESI-MS. The formation of disulfide adduct **2a** in the control experiments with small molecules can be ignored as it remains negligible upon translation of reaction to large biomolecules such as proteins.

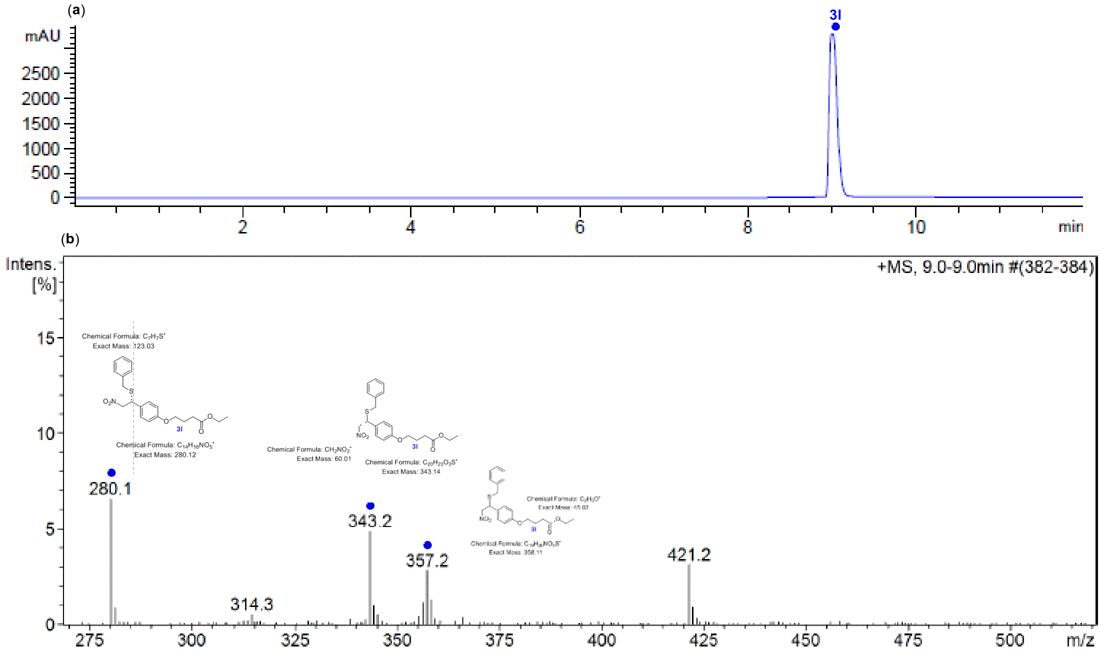

Supplementary Fig. 29. C-S bond dissociation of benzylmercaptan-nitrolefin adduct (**3l**) monitored by LC-ESI-MS. (a) LC profile for benzylmercaptan-nitrolefin adduct (**3l**). (b) ESI-MS data for benzylmercaptan-nitrolefin adduct (**3l**) with various fragment pattern of intact mass.

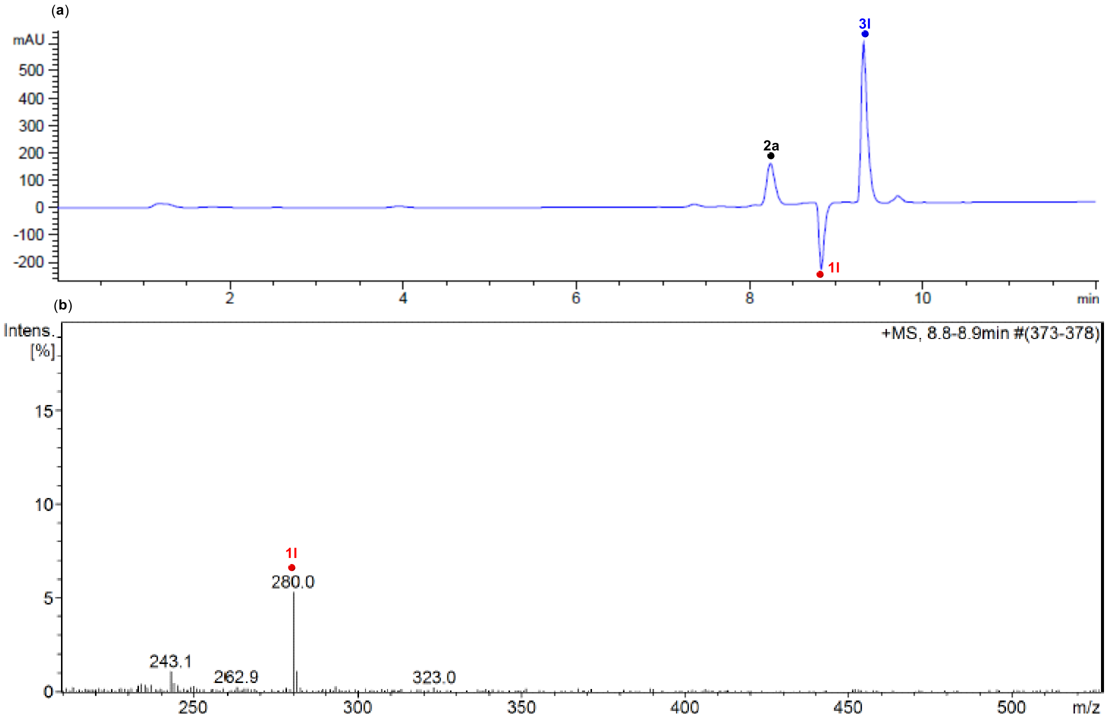

Supplementary Fig. 30. C-S bond dissociation of benzylmercaptan-nitrolefin adduct (**3l**) monitored by LC-ESI-MS. (a) LC profile for reaction of benzylmercaptan-nitrolefin adduct (**3l**) in NaP (pH 7.0, 50 mM) resulted into products (**1l**, 27% and **7a**, 0%) after 24 h. (b) ESI MS data for product **1l**.

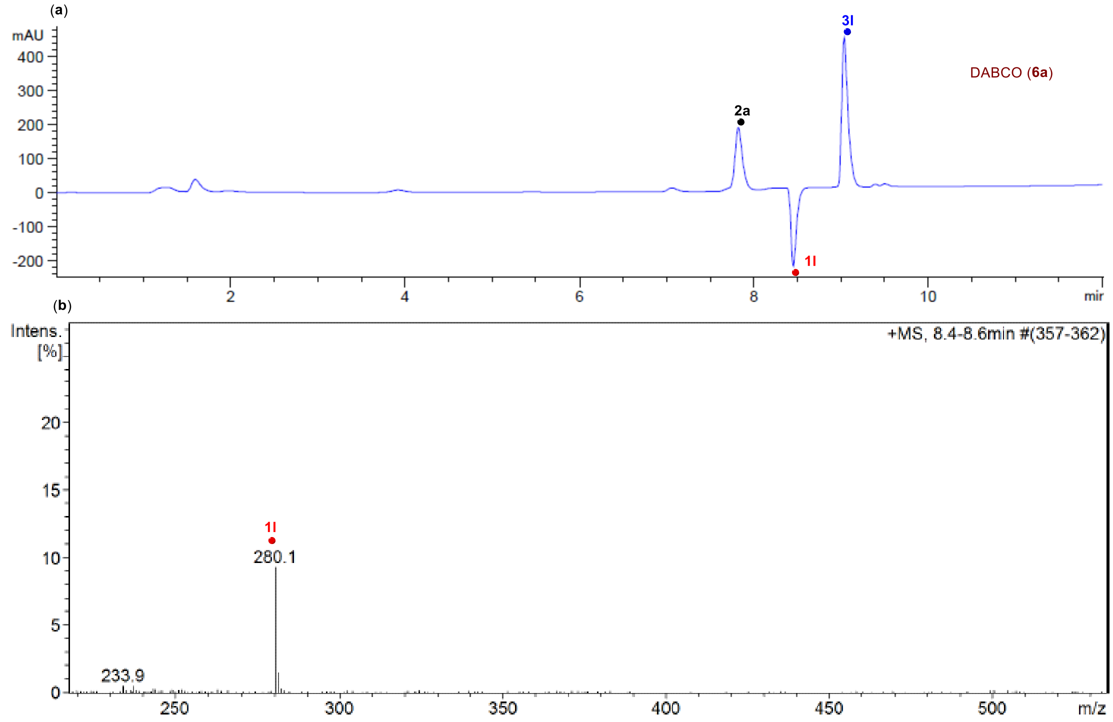

Supplementary Fig. 31. C-S bond dissociation of benzylmercaptan-nitrolefin adduct (**3l**) monitored by LC-ESI-MS. (a) LC profile for reaction of benzylmercaptan-nitrolefin adduct (**3l**) with of DABCO (**6a**, 1 equiv.) in NaP (pH 7.0, 50 mM) resulted into products (**1l**, 33% and **7a**, 0%) after 24 h. (b) ESI-MS data for product **1l**.

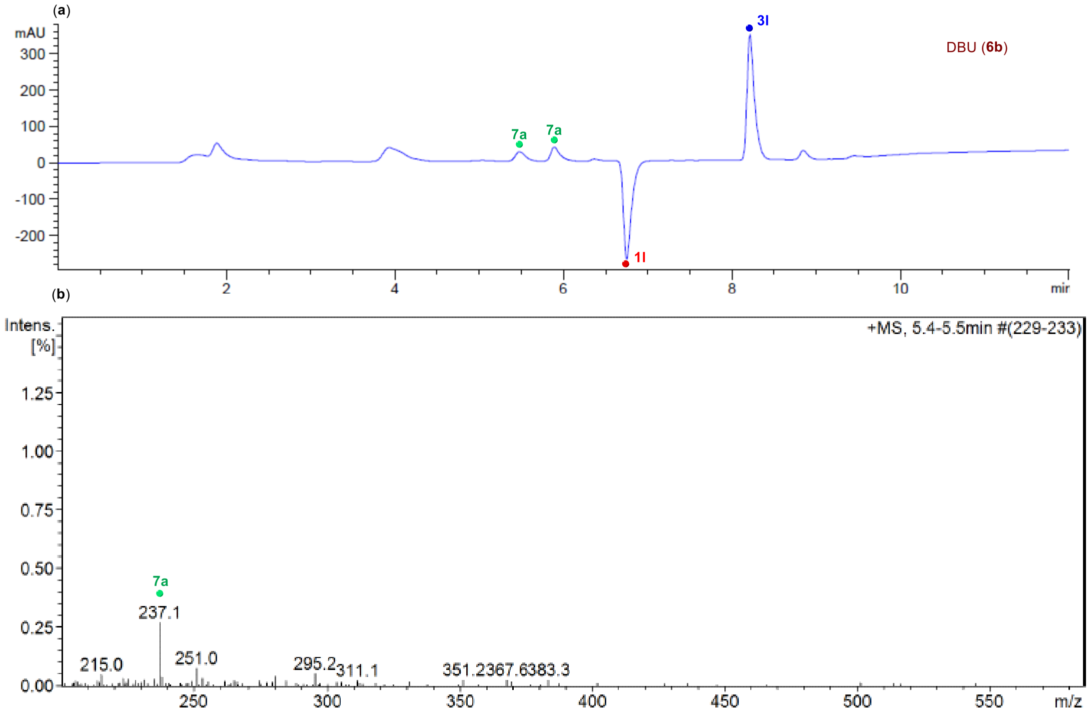

Supplementary Fig. 32. C-S bond dissociation of benzylmercaptan-nitrolefin adduct (**3l**) monitored by LC-ESI-MS. (a) LC profile for reaction of benzylmercaptan-nitrolefin adduct (**3l**) with of DBU (**6b**, 1 equiv.) in NaP (pH 7.0, 50 mM) resulted into products (**1l**, 40% and **7a**, 14%) after 24 h. (b) ESI-MS data for product **7a**.

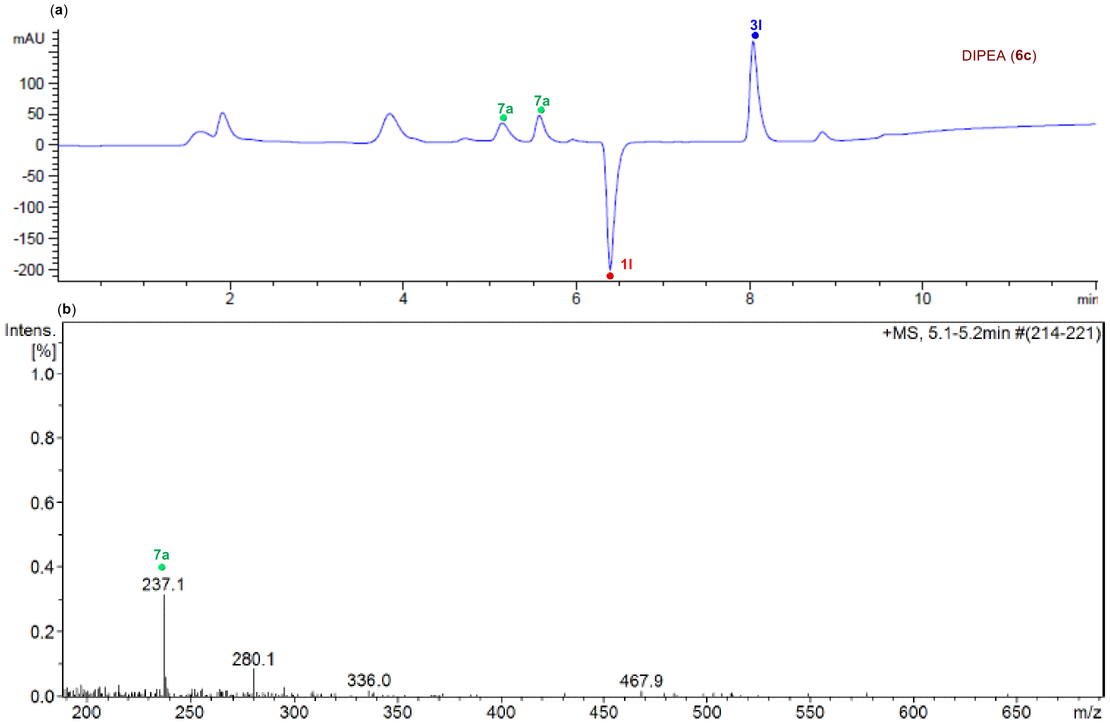

Supplementary Fig. 33. C-S bond dissociation of benzylmercaptan-nitrolefin adduct (**3l**) monitored by LC-ESI-MS. (a) LC profile for reaction of benzylmercaptan-nitrolefin adduct (**3l**) with of DIPEA (**6c**, 1 equiv.) in NaP (pH 7.0, 50 mM) resulted into products (**1l**, 42% and **7a**, 22%) after 24 h. (b) ESI-MS data for product **7a**.

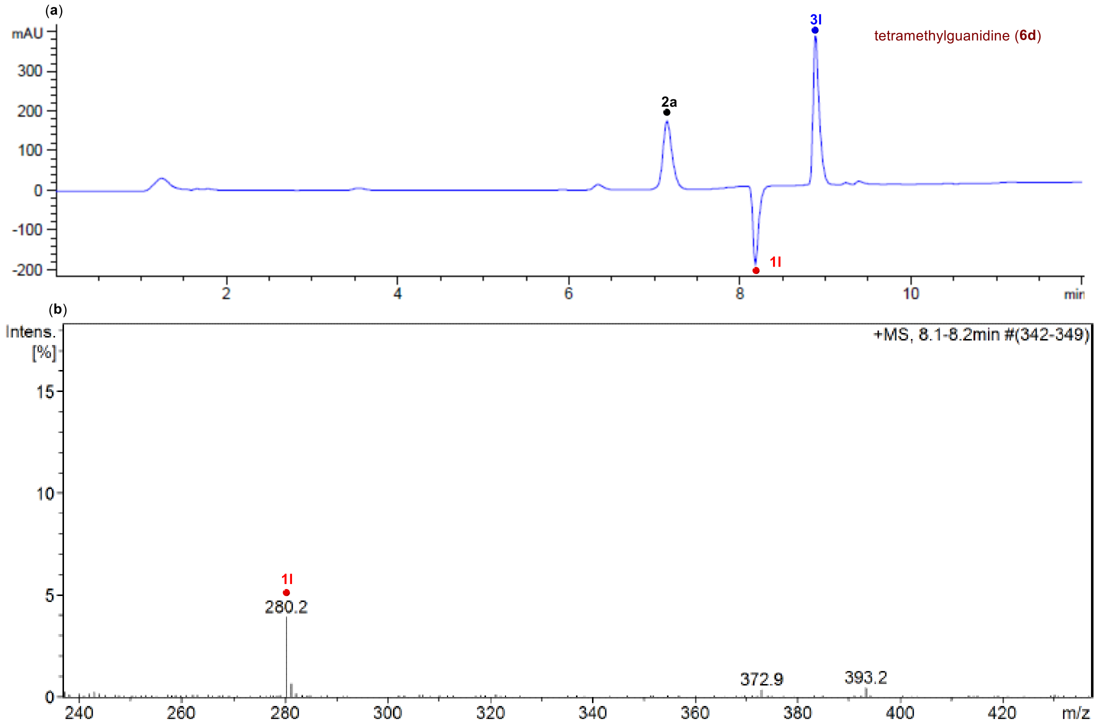

Supplementary Fig. 34. C-S bond dissociation of benzylmercaptan-nitrolefin adduct (**3l**) monitored by LC-ESI-MS. (a) LC profile for reaction of benzylmercaptan-nitrolefin adduct (**3l**) with of tetramethylguanidine (**6d**, 1 equiv.) in NaP (pH 7.0, 50 mM) resulted into products (**1l**, 33% and **7a**, 0%) after 24 h. (b) ESI-MS data for product **1l**.

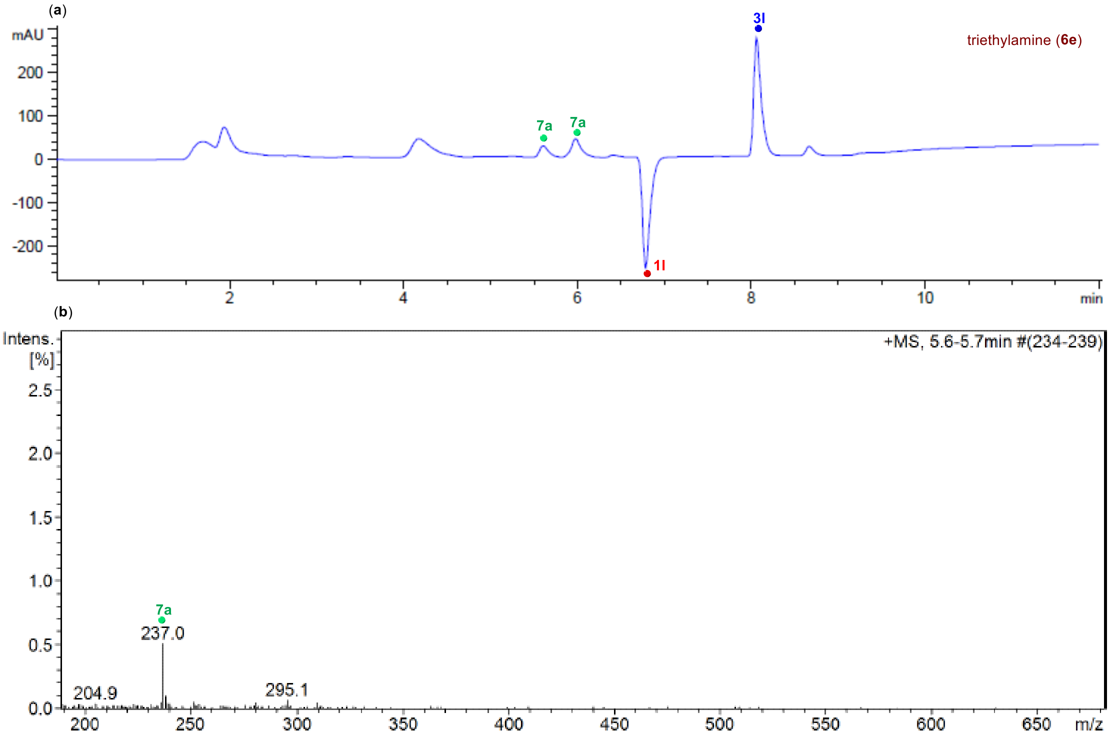

Supplementary Fig. 35. C-S bond dissociation of benzylmercaptan-nitrolefin adduct (**3l**) monitored by LC-ESI-MS. (a) LC profile for reaction of benzylmercaptan-nitrolefin adduct (**3l**) with of triethylamine (**6e**, 1 equiv.) in NaP (pH 7.0, 50 mM) resulted into products (**1l**, 40% and **7a**, 18%) after 24 h. (b) ESI-MS data for product **7a**.

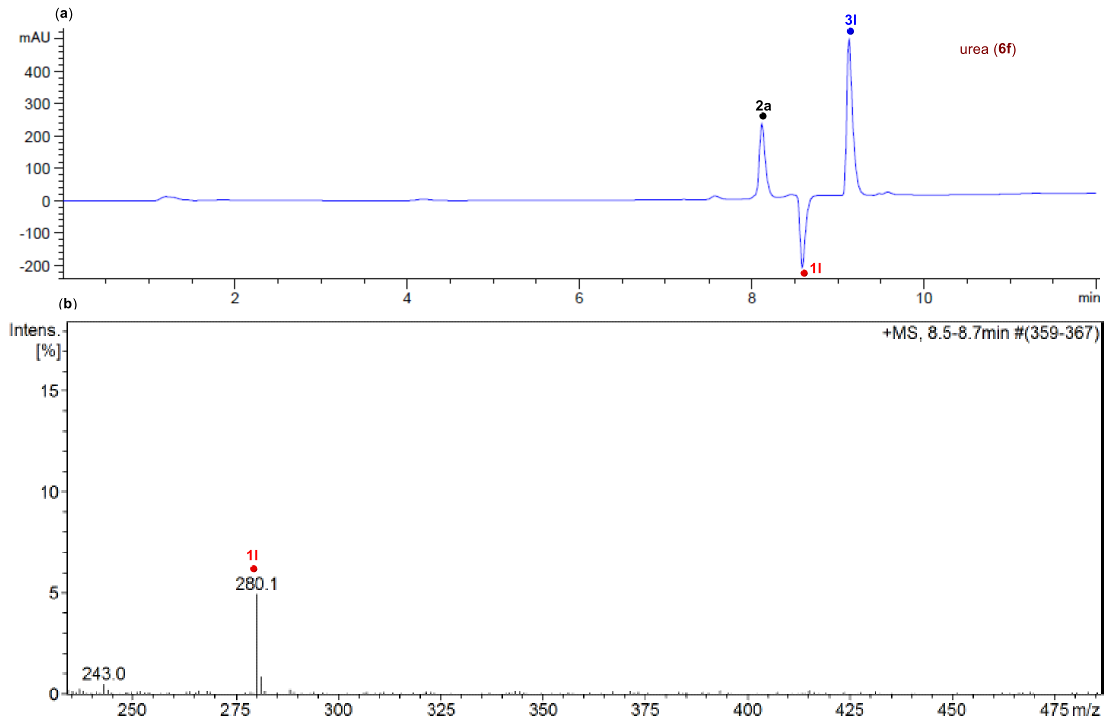

Supplementary Fig. 36. C-S bond dissociation of benzylmercaptan-F_C adduct (**3l**) monitored by LC-ESI-MS. (a) LC profile for reaction of benzylmercaptan-nitrolefin adduct (**3l**) with of urea (**6f**, 1 equiv.) in NaP (pH 7.0, 50 mM) resulted into products (**1l**, 31% and **7a**, 0%) after 24 h. (b) ESI-MS data for product **1l**.

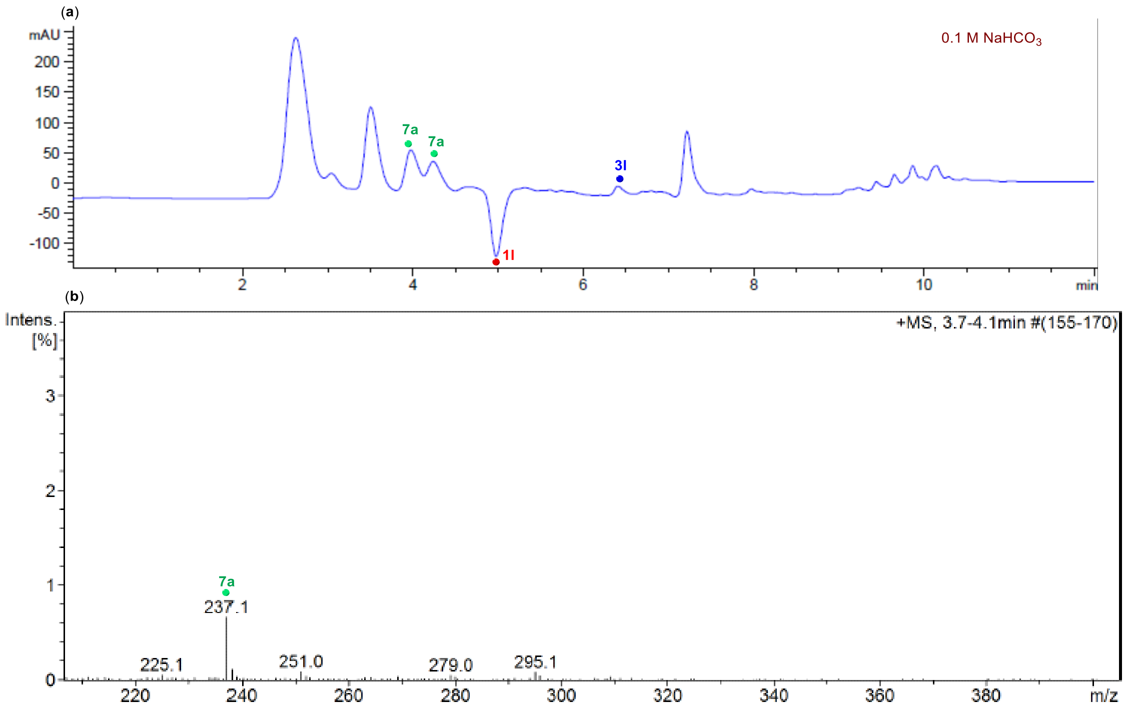

Supplementary Fig. 37. C-S bond dissociation of benzylmercaptan-nitrolefin adduct (**3l**) monitored by LC-ESI-MS. (a) LC profile for reaction of benzylmercaptan-nitrolefin adduct (**3l**) in $NaHCO_3$ (pH 8.5, 0.1 M) resulted into products (**1l**, 44% and **7a**, 55%) after 12 h. (b) ESI-MS data for product **7a**.

.

5.2 Selection of acyl-donor (F_K)

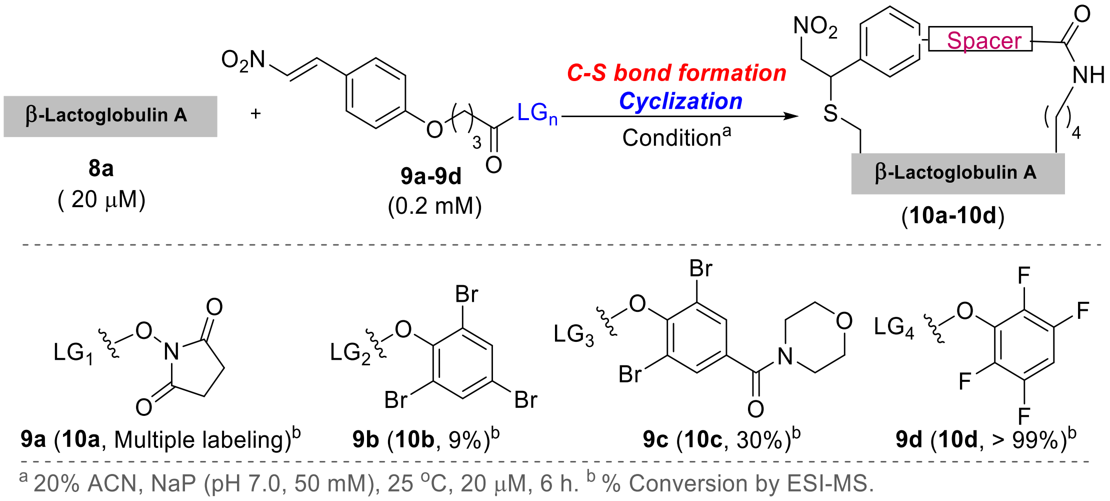

Supplementary Fig. 38. Screening of various electrophiles for selection of acyl-donor (F_K).

Native BLG A (β-lactoglobulin variant A)

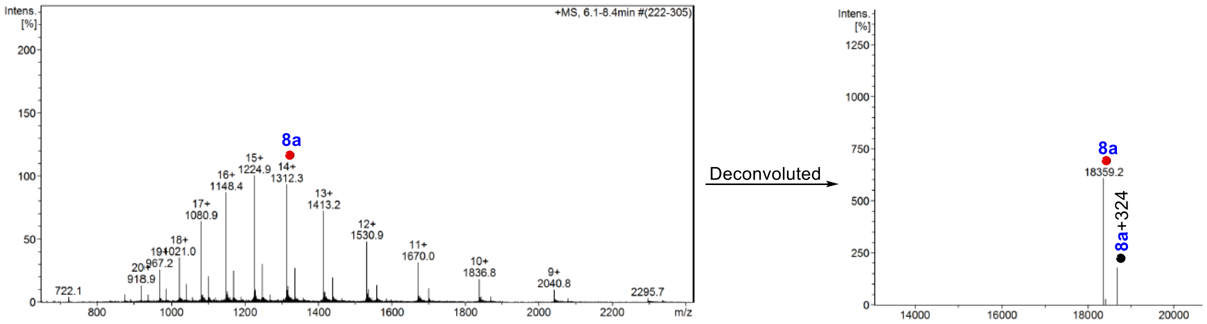

Supplementary Fig. 39. ESI-MS spectrum for β-lactoglobulin variant A [**8a**, (**8a**+324)].

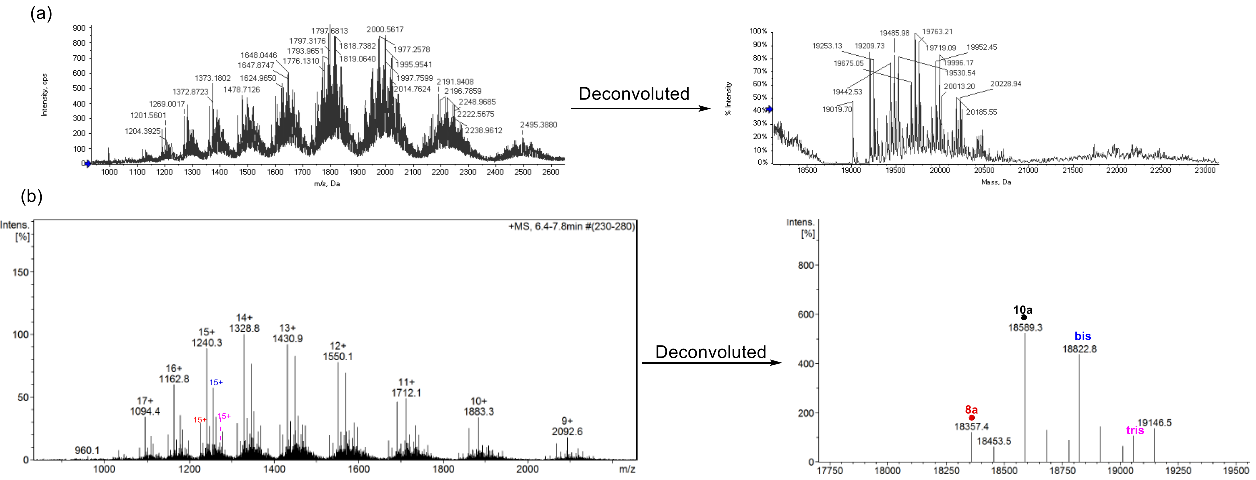

Supplementary Fig. 40. (a) ESI-MS spectrum for highly heterogenous-labeled β-lactoglobulin A from the reaction of β-lactoglobulin A **8a** (1 equiv.) and $LDM_{C\text{-}K}$ reagent **9a** (10 equiv.) after 6 h. (b) ESI-MS spectrum for highly multi-labeled β-lactoglobulin A **10a** from the reaction of β-lactoglobulin A **8a** (1 equiv.) and $LDM_{C\text{-}K}$ reagent **9a** (10 equiv.) after 30 sec.

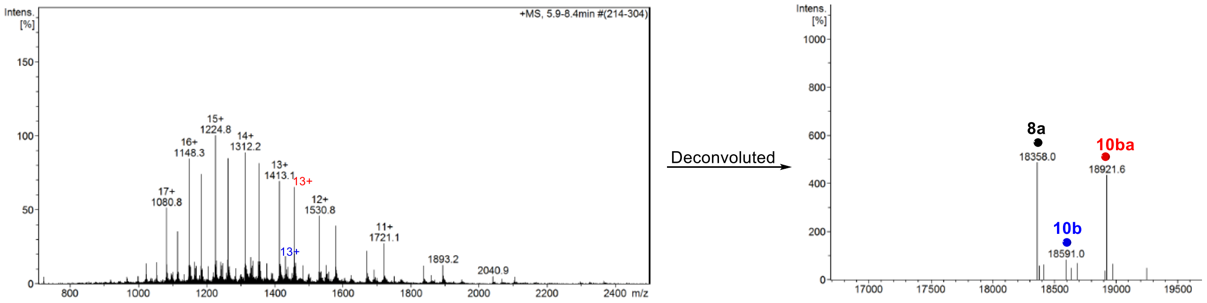

Supplementary Fig. 41. ESI-MS spectrum for mono-labeled β-lactoglobulin A **10b** from the reaction of β-lactoglobulin A **8a** (1 equiv.) with LDM$_{C\text{-}K}$ reagent **9b** (10 equiv.). **10ba** indicates the Michael adduct of β-lactoglobulin A with reagent **9b.**

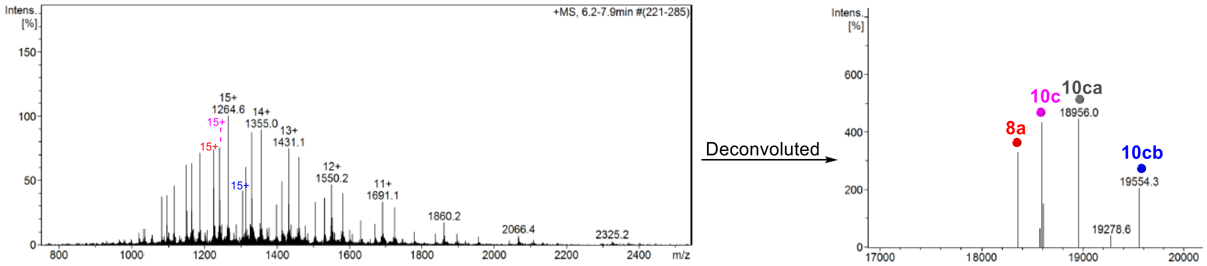

Supplementary Fig. 42. ESI-MS spectrum for mono-labeled β-lactoglobulin A **10c** from the reaction of β-lactoglobulin A **8a** (1 equiv.) and LDM$_{C\text{-}K}$ reagent **9c** (10 equiv.). **10ca** and **10cb** indicates mono and bis-labeled Michael adduct of β-lactoglobulin A with reagent **9c**.

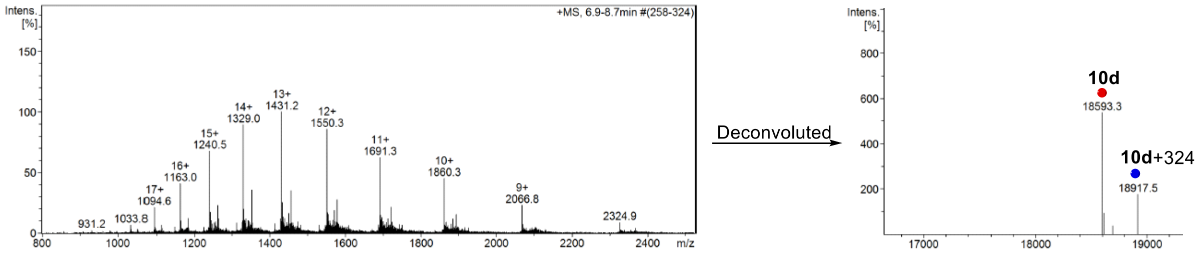

Supplementary Fig. 43. ESI-MS spectrum for mono-labeled β-lactoglobulin A **10d** from the reaction of β-lactoglobulin A **8a** (1 equiv.) with LDM$_{C\text{-}K}$ reagent **9d** (10 equiv.).

5.3 Peptide modification

Supplementary Table 4. Peptide screening

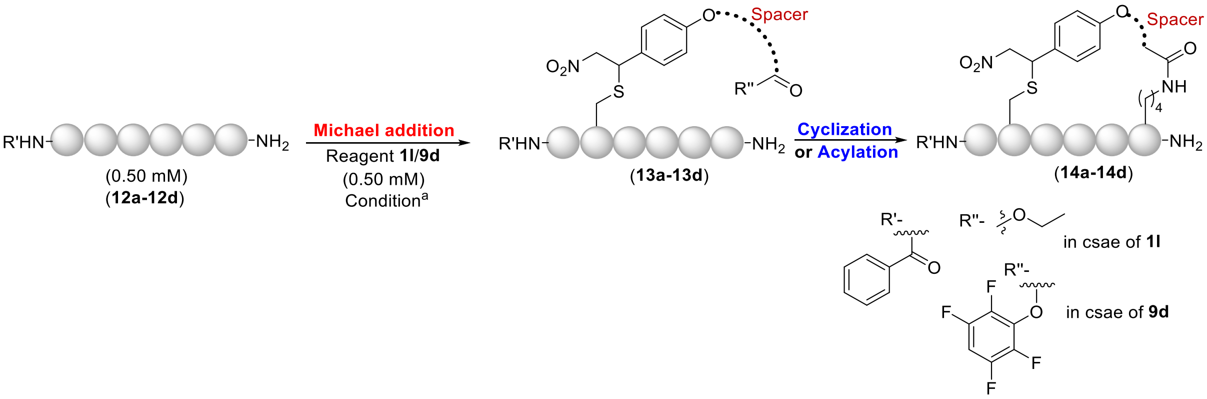

Entry	Peptides	% Conversion[b]	
		13a-13d	14a-14d
1[c]	Bz-LCGLLG-NH2 (**12a**)	77 (**13a**)	0
2[d]	Bz-LCKGLL-NH2 (**12S1**)	0 (**13S1**)	65 (**14S1**)
3[d]	Bz-LCGKLL-NH2 (**12S2**)	0 (**13S2**)	33 (**14S2**)
4[d]	Bz-LCGLKL-NH2 (**12S3**)	0 (**13S3**)	75 (**14S3**)
5[d]	Bz-LCGLLK-NH2 (**12b**)	3 (**13b**)	75 (**14b**)
6[e]	Bz-C(^{t}Bu)GGGK-NH2 (**12c**)	0 (**13c**)	0 (**14c**)
7[f]	Bz-LCGLLKGLLK-NH2 (**12d**)	5 (**13d**)	33 (**14d**) (K6)<br>0 (K10)

[a] 30% ACN, NaP (pH 7.0, 50 mM), 500 µM, 25 °C, 10-60 min. [b] % Conversions by LC-ESI-MS.[c] Peptide containing only Cys **12a** resulted in F_C adduct **13a** with **1l** reagent. [d] Peptide containing Cys (ith) and Lys (i+1 to i+4) **12s1-12b** position were treated with $LDM_{C\text{-}K}$ reagent **9d** resulted into F_C adduct and cyclization. The high conformational flexibility of a small peptide counters the effect from relative Cys/Lys position. However, this effect becomes more pronounced in the case of proteins. [e] Peptide having Cys protected with ^{t}Bu and free Lys **12c** was reacted with $LDM_{C\text{-}K}$ reagent **9d** to check the rate of acylation.[f] Peptide containing Cys (ith) and Lys at two distinct position (i+4 and i+8) **12d** were treated with $LDM_{C\text{-}K}$ reagent **9d** for site-selectivity. All the peptides used for the study here, were N-terminus protected with benzoyl group to get better LC profile and the C-terminus carboxylate were converted to amide using rink amide resin via solid phase peptide synthesizer.

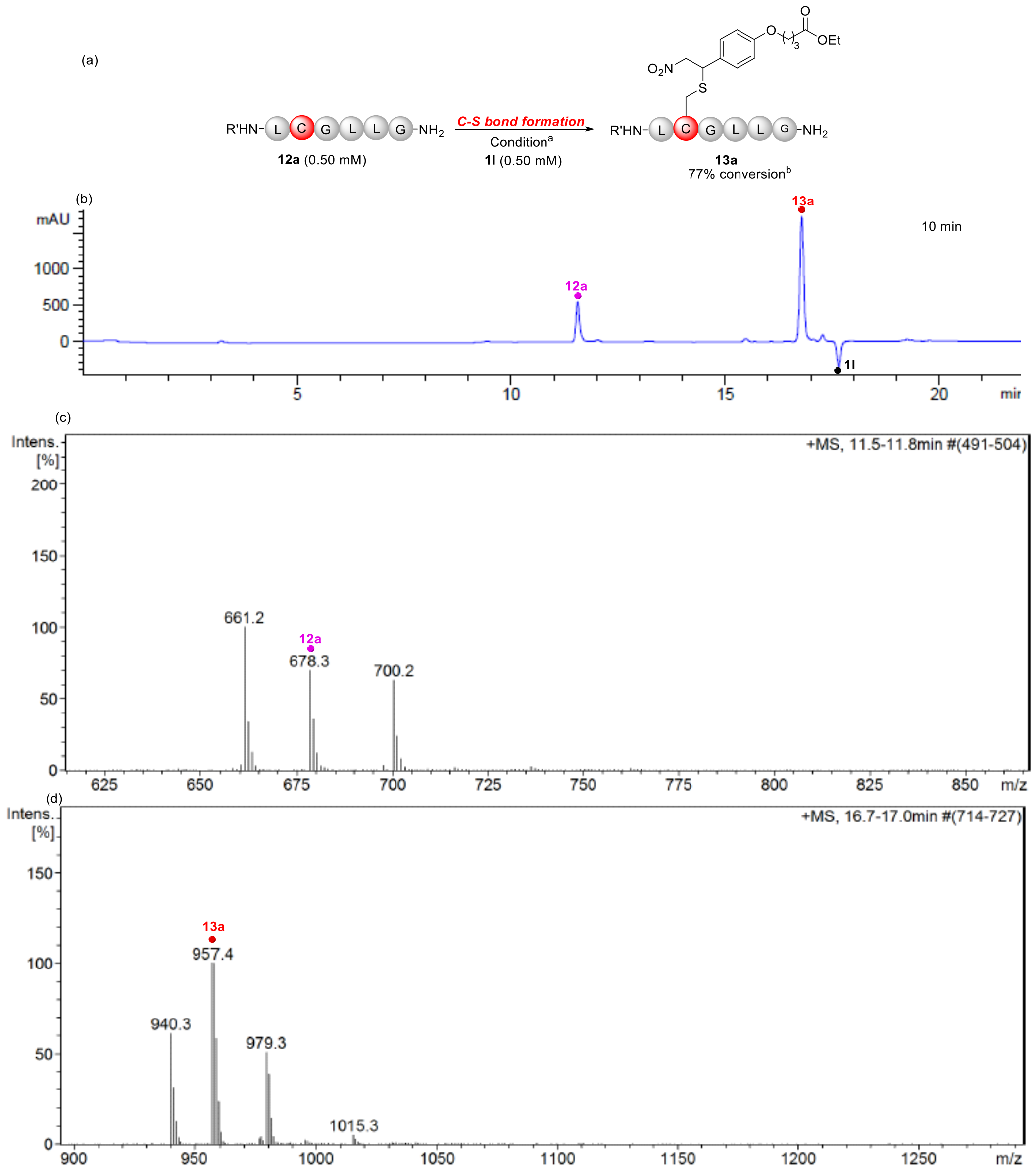

[a] 30% ACN, NaP (pH 7.0, 50 mM), 500 μM, 25 °C, 10 min. [b] % Conversions by LC-ESI-MS.

Supplementary Fig. 44. (a) C-S bond formation for Cys containing peptide **12a** with reagent **1l**. (b) LC data for F_C adduct **13a** of Cys containing peptide **12a** enabled by reagent 1l after 10 min. (c) ESI-MS data for **12a**. (d) ESI-MS data for F_C adduct **13a**.

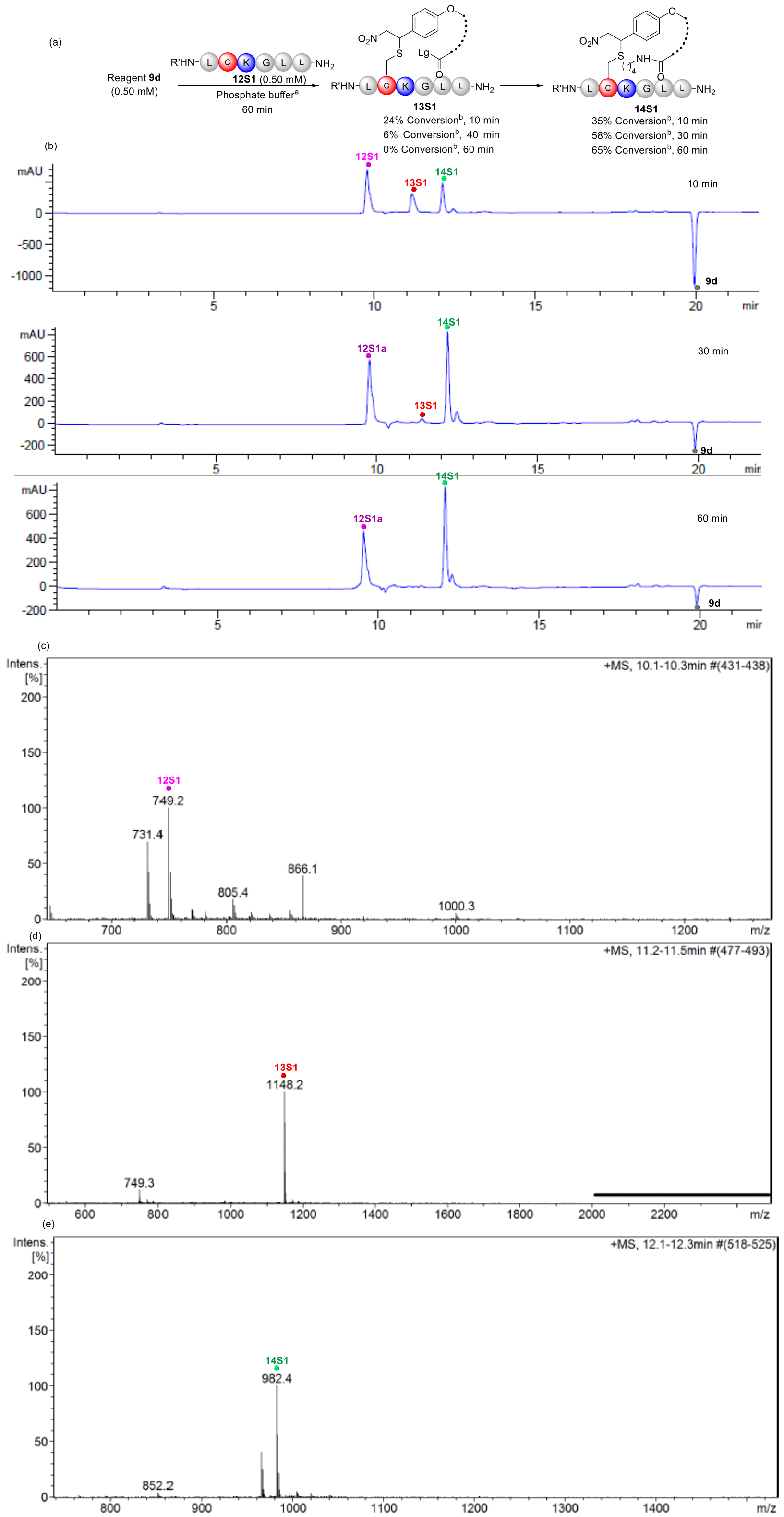

[a]NaP (pH 7.0, 50 mM), 30% ACN, 25 °C, 500 μM, 60 min
[b]% Conversion of **13S1** and **14S1** were esimated on the basis of **12S1** using LC-ESI-MS.
Under the physiological reaction condition oxidation of **12S1** results into **12S1a** (disulfide), which limit the conversion of **13S1** and **14S1**.

Supplementary Fig. 45. (a) C-S bond formation and cyclization for peptide containing Cys (i^h) and lysine (i+1) **12S1** enabled by LDM$_{C\text{-}K}$ reagent **9d**. (b) LC data showing C-S bond formation and cyclization for peptide containing Cys (i^{th}) and lysine (i+1) enabled by reagent **9d** for different interval time (10-60 min). (c) ESI-MS data for **12S1**. (d) ESI-MS data for Michael adduct **13S1**. (e) ESI-MS data for cyclized peptide **14S1**.

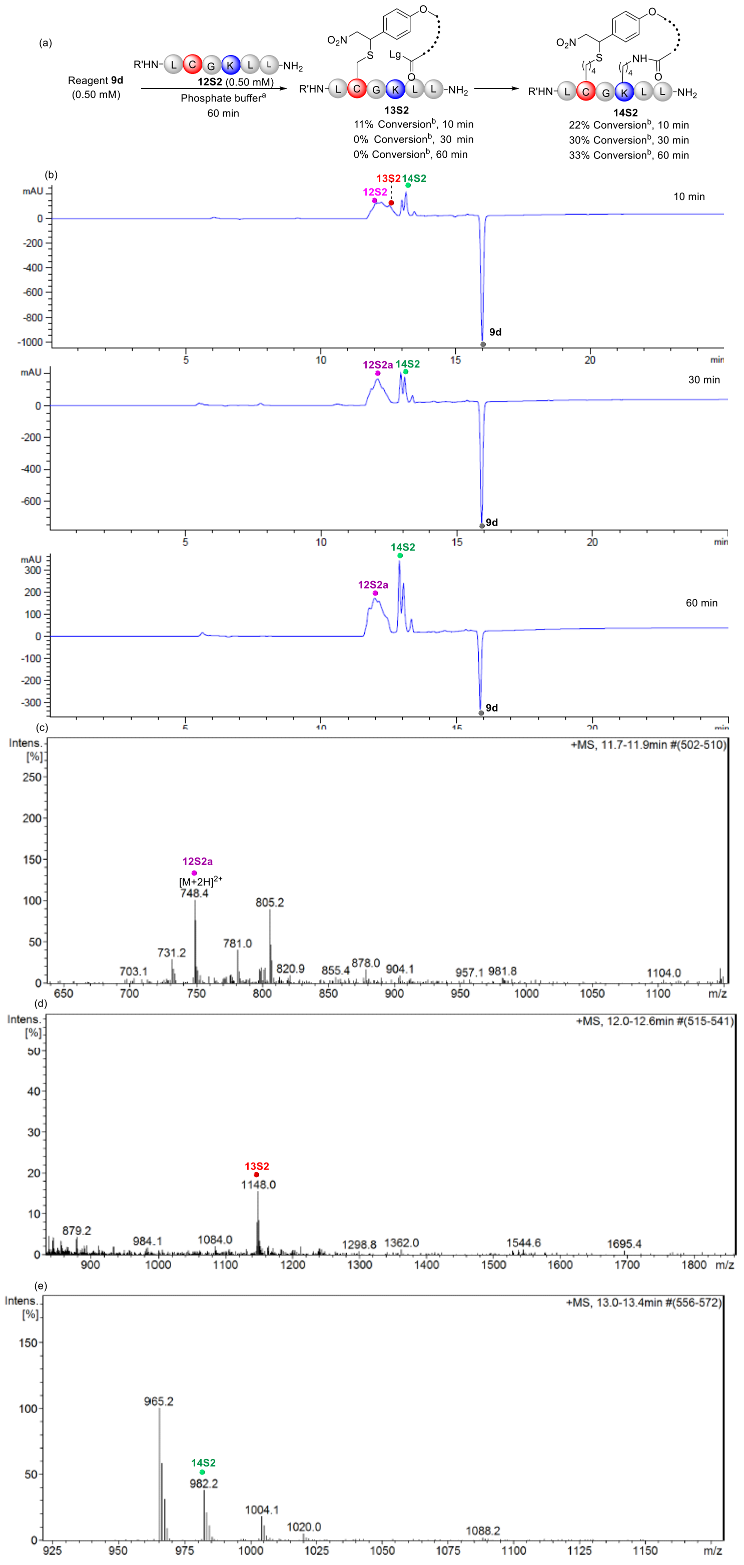

[a]NaP (pH 7.0, 50 mM), 30% ACN, 25 °C, 500 μM, 60 min

[b]% Conversion of **13S2** and **14S2** were esimated on the basis of **12S2**using LC-ESI-MS.

Under the physiological reaction condition oxidation of **12S2** results into **12S2a** (disulfide), which limit the conversion of **13S2** and **14S2.**

Supplementary Fig. 46. (a) C-S bond formation and cyclization for peptide containing Cys (i[th]) and lysine (i+2) **12S2** enabled by LDM$_{C\text{-}K}$ reagent **9d**. (b) LC data showing C-S bond formation and cyclization for peptide containing Cys (i[th]) and lysine (i+2) enabled by reagent **9d** for different interval time (10-60 min). (c) ESI-MS data for **12S2a**. (d) ESI-MS data for Michael adduct **13S2**. (e) ESI-MS data for cyclized peptide **14S2**.

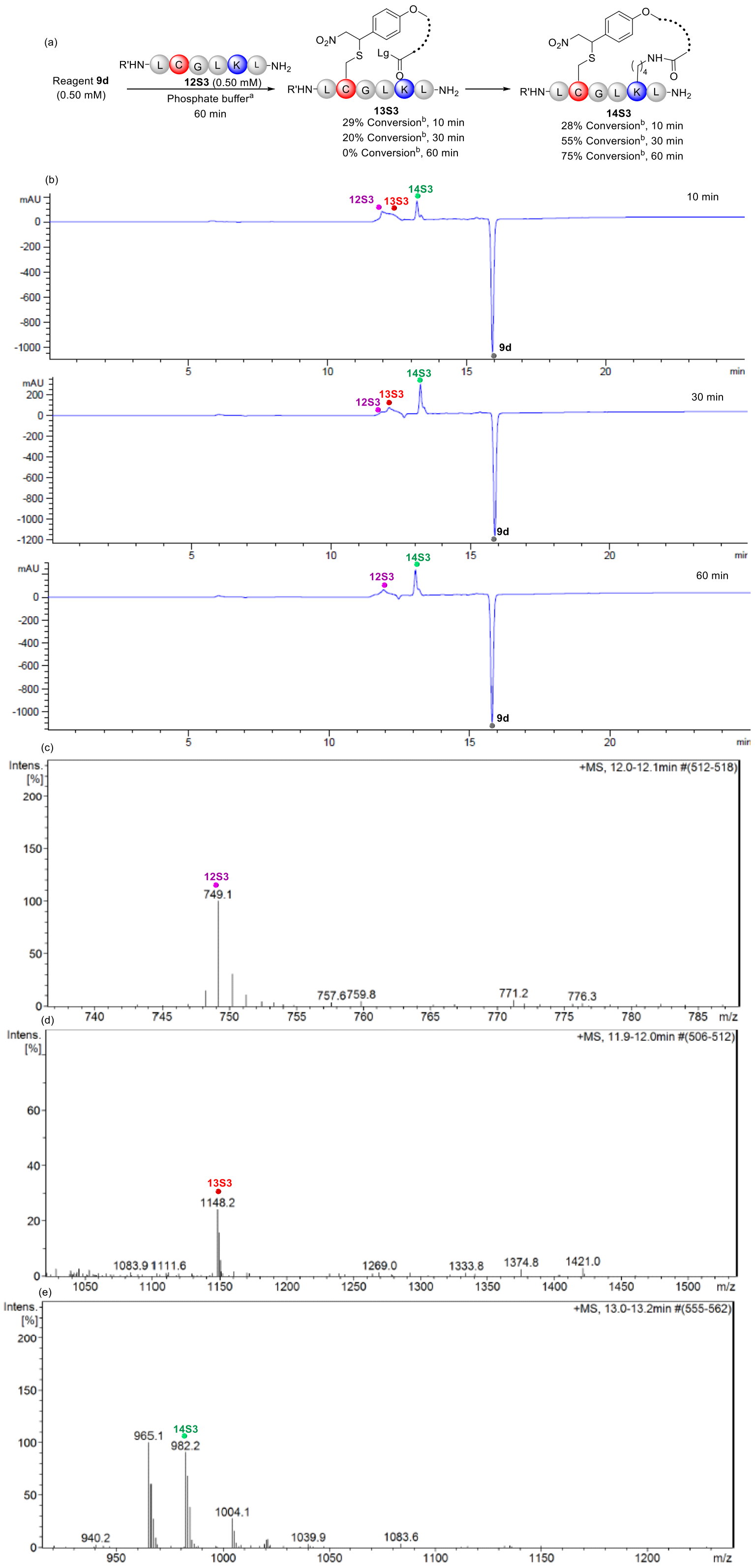

[a]NaP (pH 7.0, 50 mM), 30% ACN, 25 °C, 500 μM, 60 min

[b]% Conversion of **13S3** and **14S3** were esimated on the basis of **12S3** using LC-ESI-MS.

Supplementary Fig. 47 (a) C-S bond formation and cyclization for peptide containing Cys (ith) and lysine (i+3) **12S3** enabled by LDM$_{C\text{-}K}$ reagent **9d**. (b) LC data showing C-S bond formation and cyclization for peptide containing Cys (ith) and lysine (i+3th) enabled by reagent **9d** for different interval time (10-60 min). (c) ESI-MS data for **12S3**. (d) ESI-MS data for Michael adduct **13S3**. (e) ESI-MS data for cyclized peptide **14S3**.

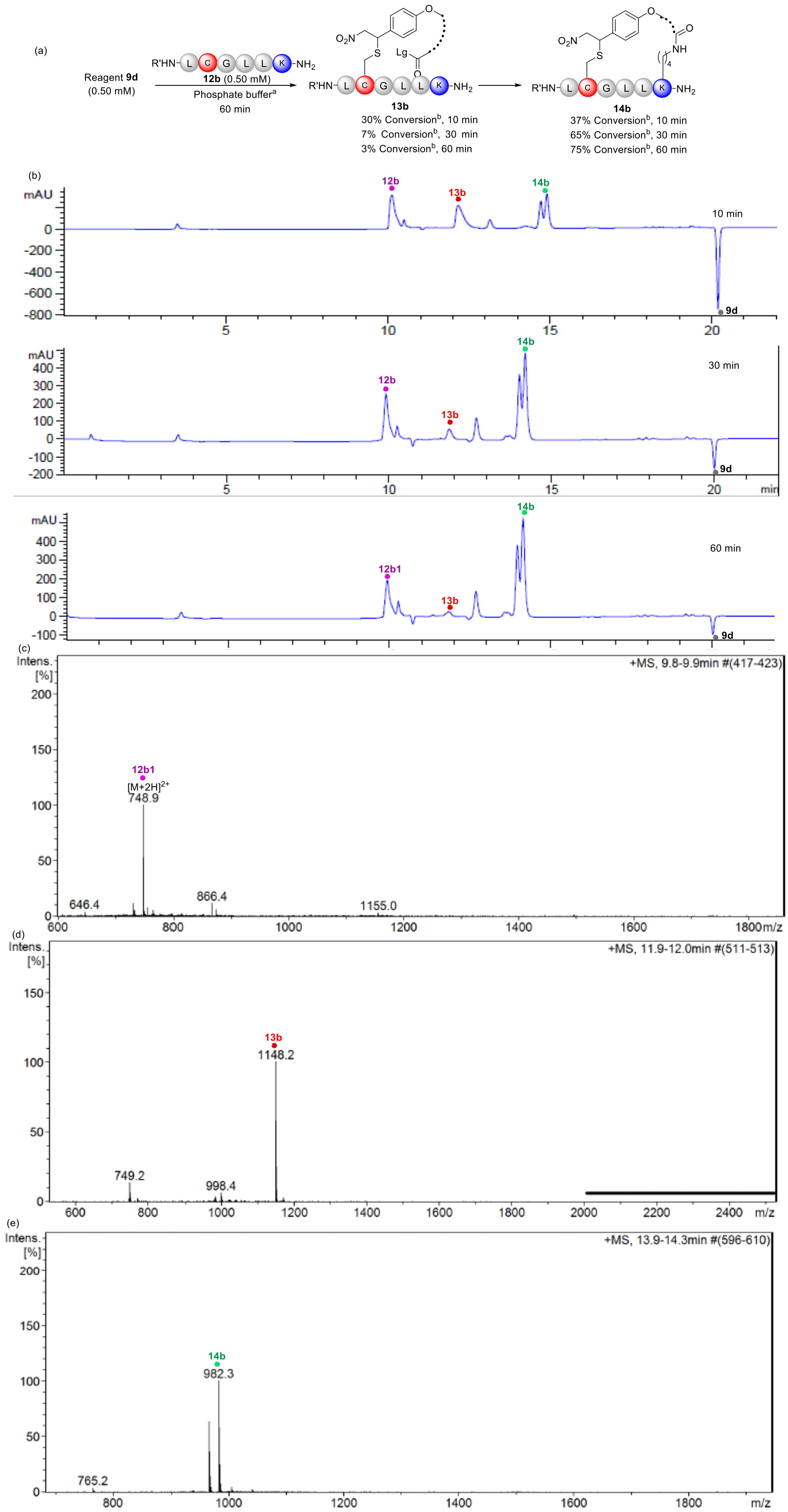

[a] NaP (pH 7.0, 50 mM), 30% ACN, 25 °C, 0.50 mM, 60 min

[b] % Conversion of **13b**and **14b** were esimated on the basis of **12b** using LC-ESI-MS.

Under the physiological reaction condition oxidation of **12b** results into **12b1** (disulfide), which limit the conversion of **13b** and **14b.**

(f)

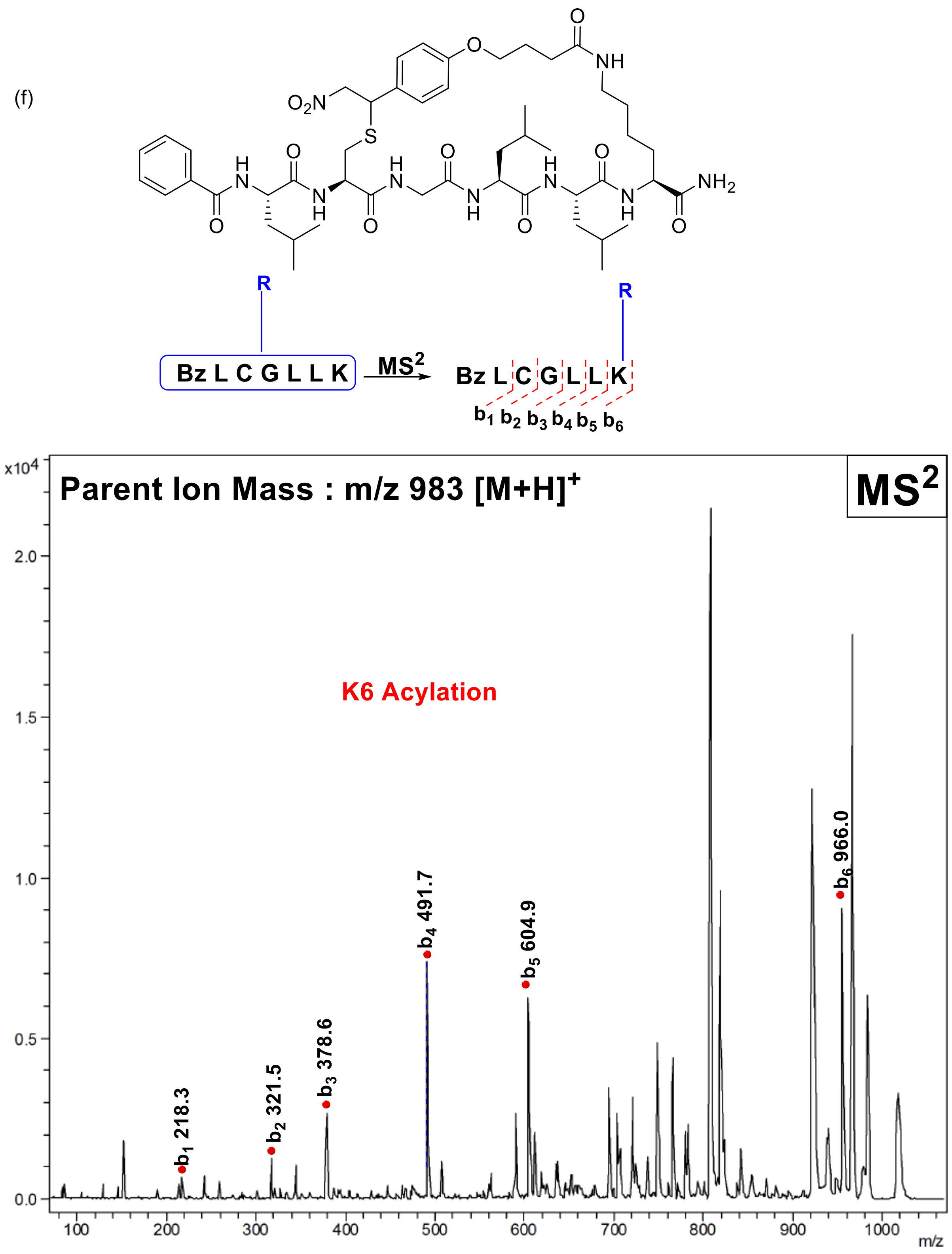

Supplementary Fig. 48. (a) C-S bond formation and cyclization for peptide containing Cys (ith) and lysine (i+4th) **12b** enabled by LDM$_{C\text{-}K}$ reagent **9d**. (b) LC data showing C-S bond formation and cyclization for peptide containing Cys (ith) and lysine (i+4th) enabled by reagent **9d** with different interval time (10-60 min). (c) ESI-MS data for **12b1**. (d) ESI-MS data for Michael adduct **13b**. (e) ESI-MS data for cyclized peptide **14b**. (f) MS-MS data for **K6** modification in **14b**.

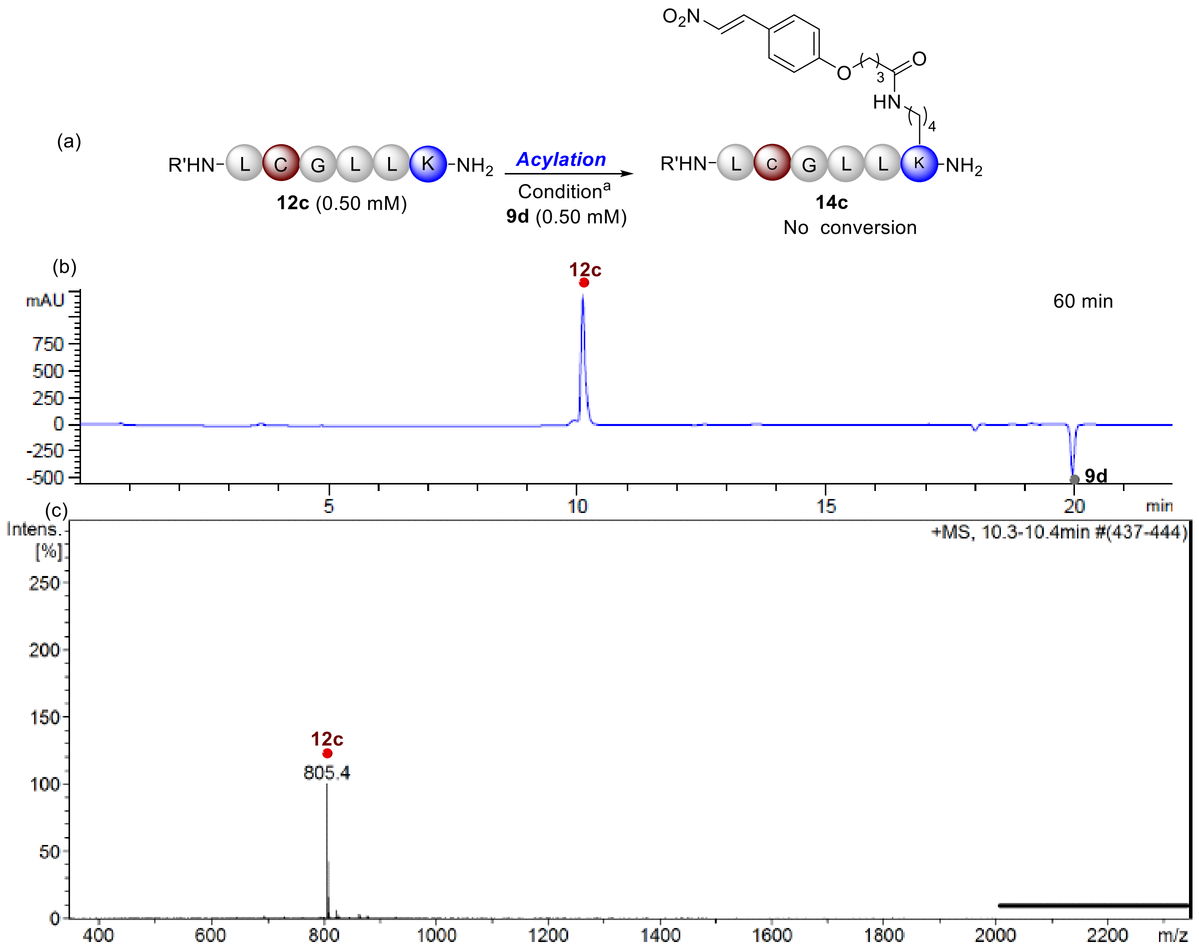

[a] 30% ACN, NaP (pH 7.0, 50 mM), 500 μM, 25 °C, 60 min. [b] % Conversions by LC-ESI-MS.

Supplementary Fig. 49. (a) Acylation of Lys containing peptide (in which Cys protected with ^{t}Bu group) **12c** enabled by LDM$_{C\text{-}K}$ reagent **9d**. (b) LC data for acylation of Lys containing peptide **12c** enabled by reagent **9d**. (c) ESI-MS data for **12c**.

Peptide having Lys at two distinct positions

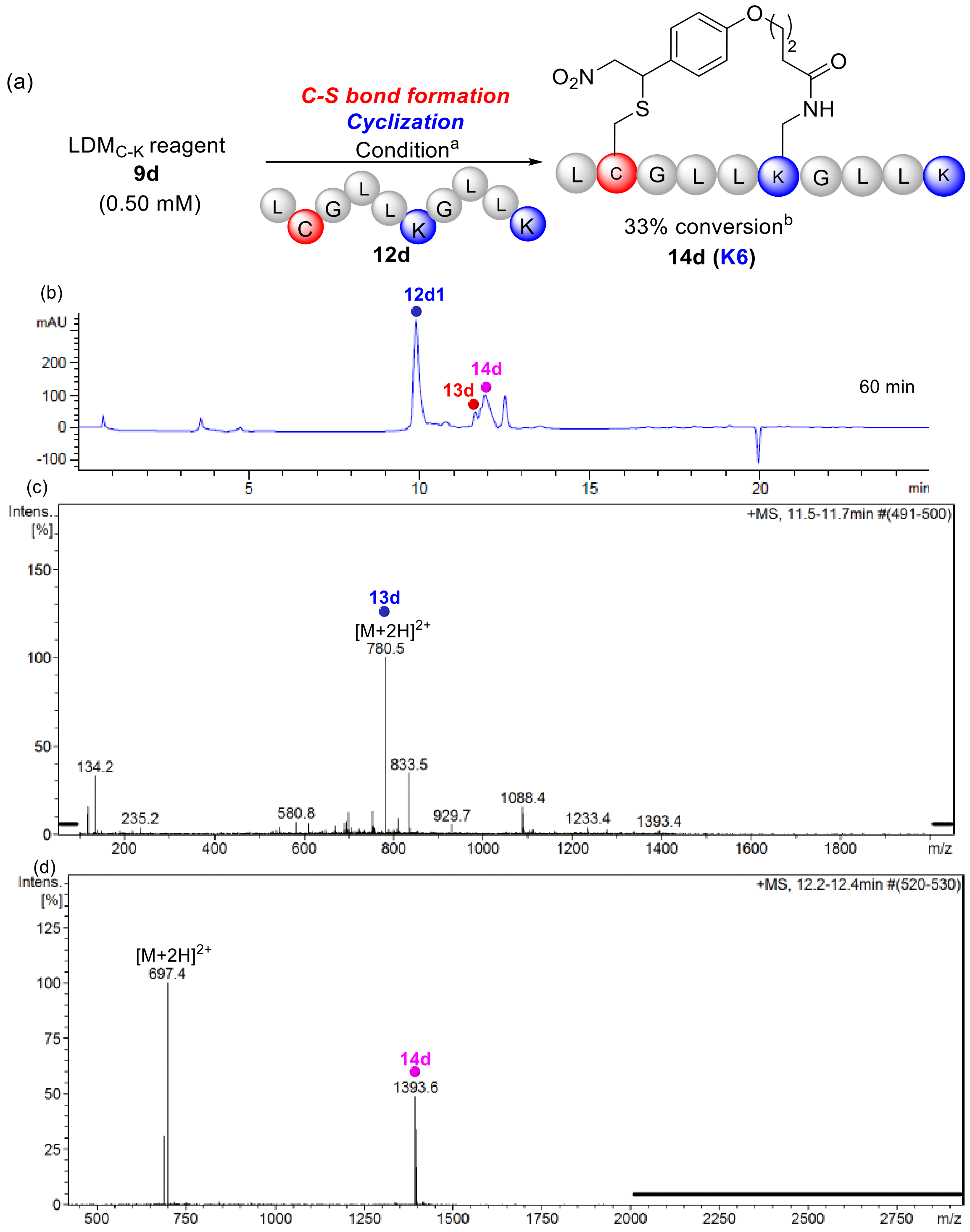

[a] 30% ACN, NaP (pH 7.0, 50 mM), 500 µM, 25 °C, 60 min. [b] % Conversions by LC-ESI-MS.
12d1 indicates oxidised and other mases observed in the reaction mixture after 60 min but not the mass of **12d**

(e)

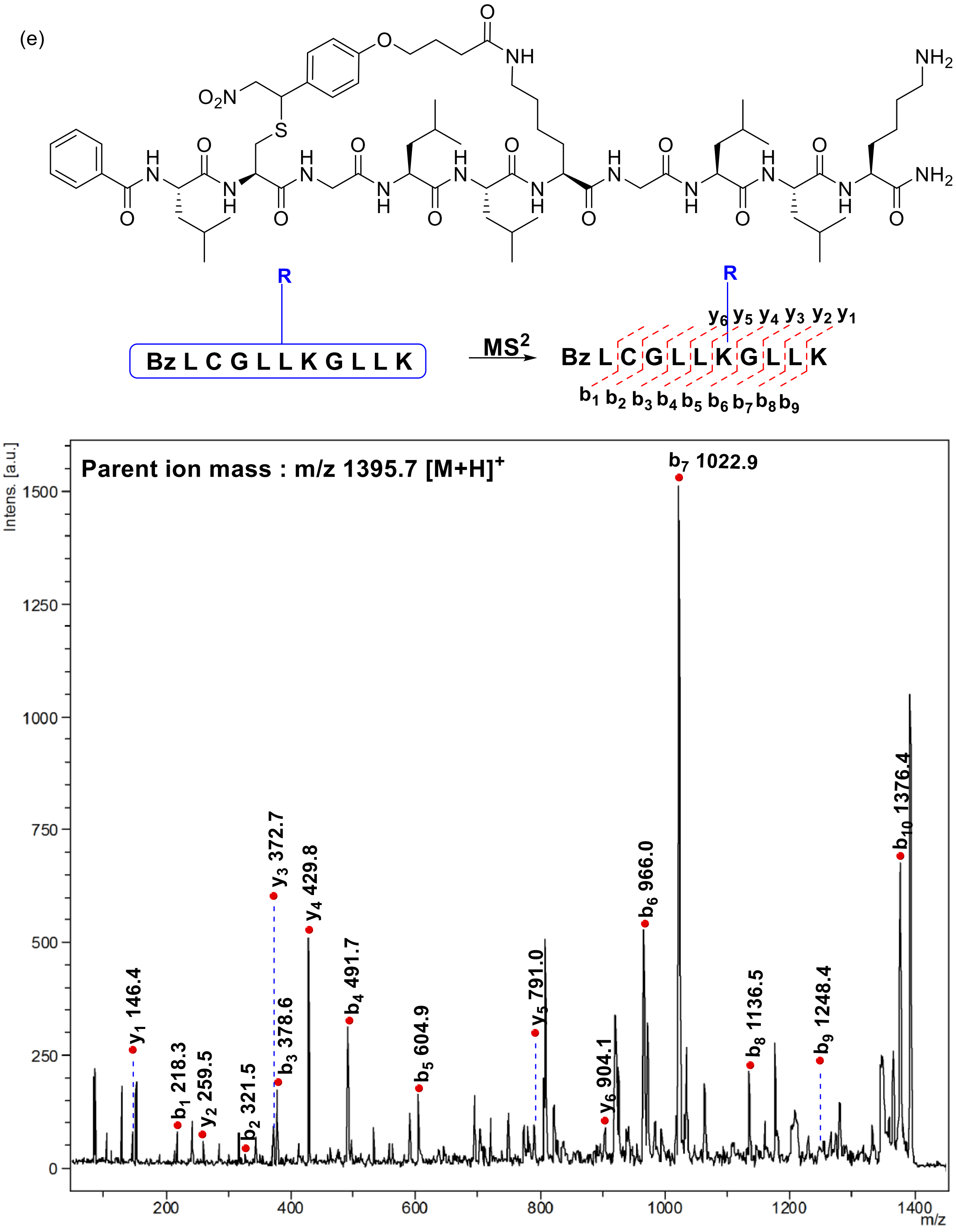

Supplementary Fig. 50. (a) Macrocyclization of Cys and two Lys containing peptide (i+4 and i+8) **12d** enabled by LDM_{C-K} reagent **9d**. (b) LC data for macrocyclization of cysteine and two Lys containing peptide (i+4 and i+8) **12d** enabled by LDM_{C-K} reagent **9d**. (c) ESI-MS data for **12d**. (d) ESI-MS data for cyclized peptide **14d**. (e) MS-MS of cyclic product **14d** further confirmed the site-selective modification i.e., K6.

6. Supplementary Notes 3: protein labelling

6.1 Screening of proteins with nitroolefin

Supplementary Table 5. Screening of proteins with nitroolefin

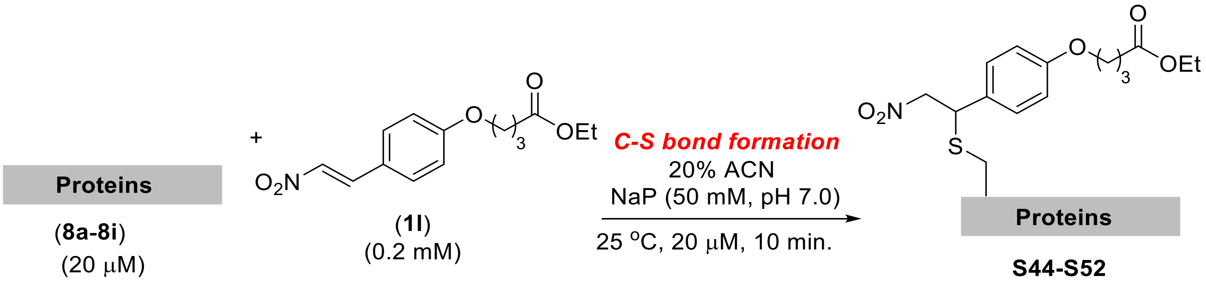

Entry	Proteins	Product (S44-S51)	% Conversion[a]
**1[b]**	**β-Lactaglobulin A(8a)**	**S44**	**88 (m)**
2	Lysozyme C (**8b**)	**S45**	0
3	Myoglobin (**8c**)	**S46**	0
4	Cytochrome C (**8d**)	**S47**	0
5	α-lactalbumin (**8e**)	**S48**	0
6	Insulin (**8f**)	**S49**	0
7	Ubiquitin (**8g**)	**S50**	0
8	RNase A (**8h**)	**S51**	0
9	Aprotinin (**8i**)	**S52**	0

[a] % Conversion based on MALDI-MS.[b] m-mono-labeled β-lactoglobulin A (**S44**) with reagent **1l** within 10 min.

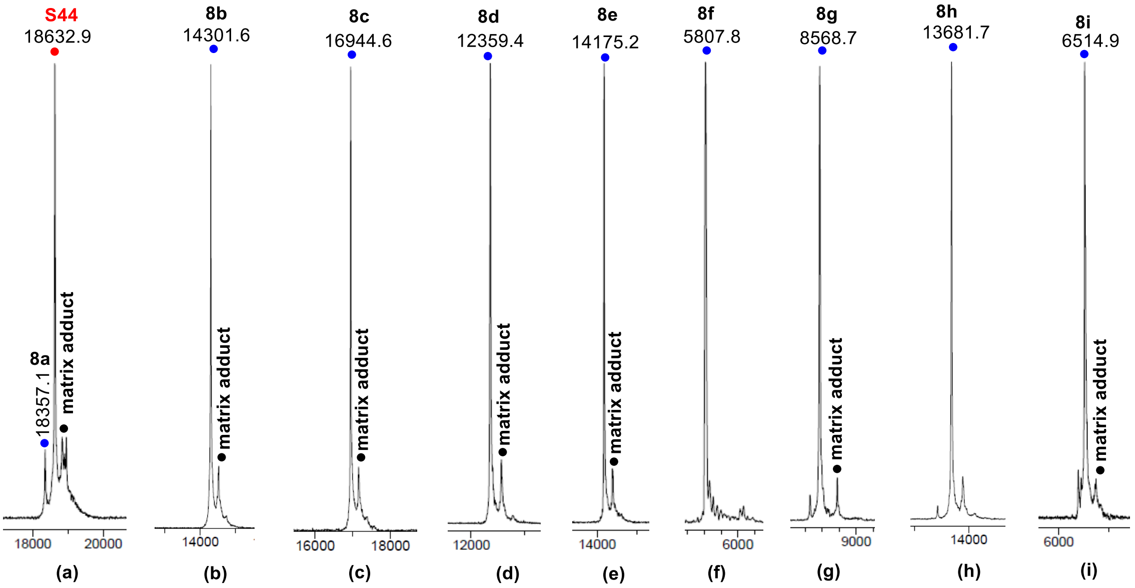

Supplementary Fig. 51. MALDI-MS spectrum for various proteins (**8a-8i**) including mono-labeled β-lactoglobulin A (**S44**, 88% conversion) after the reaction with reagent **1l**.

6.1.1 C-S bond formation of nitroolefin with β-lactoglobulin

(a)

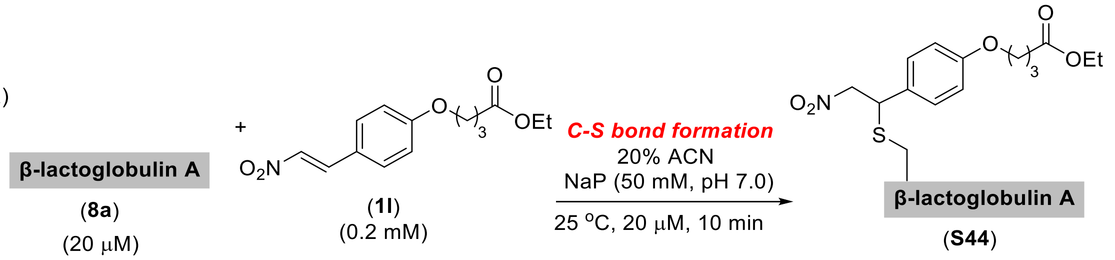

(b)

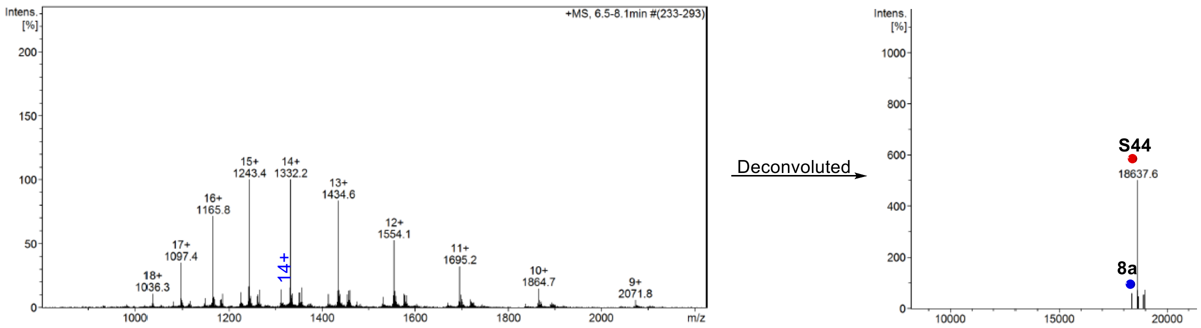

(c)

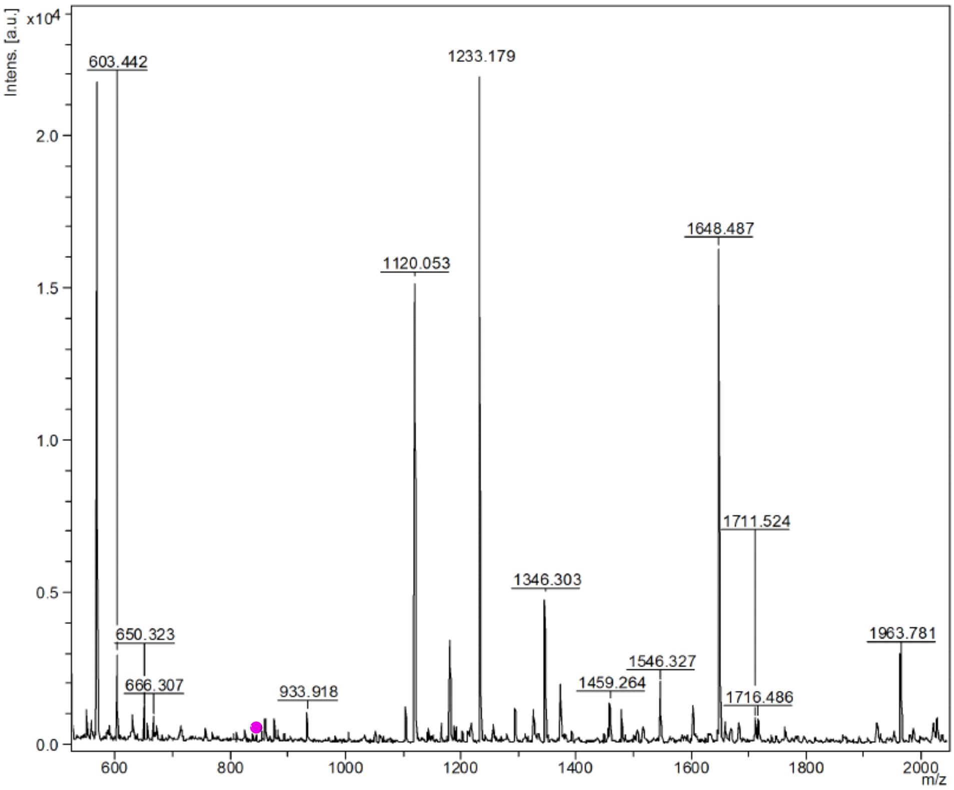

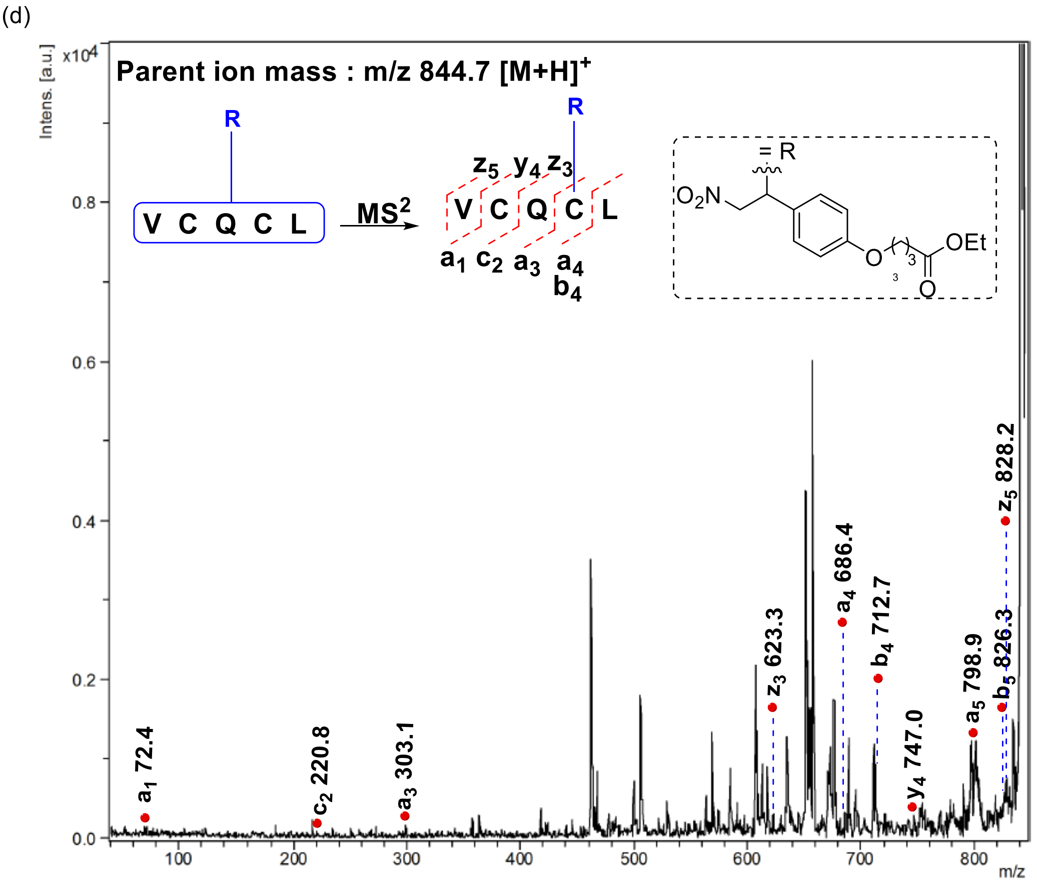

Supplementary Fig. 52. (a) Michael addition of β-lactoglobulin A **8a** (1 equiv.) enabled by reagent **1l** (10 equiv.). (b) ESI-MS spectrum for mono-labeled β-lactoglobulin A **S44**. (b) Peptide mapping of **S44** after the digestion with α-chymotrypsin. (d) MS-MS spectrum of labeled VCQCL (V118-L122, *m/z* 844.7 $[M+H]^+$). The site of modification in mono-labeled β-lactoglobulin A **S44** is C121.

6.2 Acylation of lysine in BLG-A (Cys-Protected)

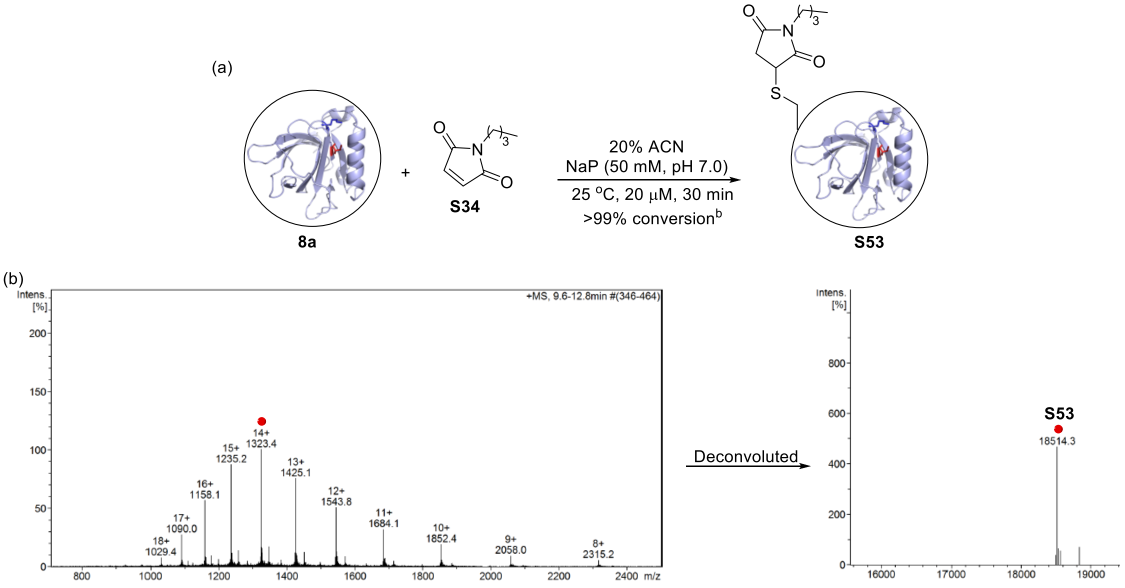

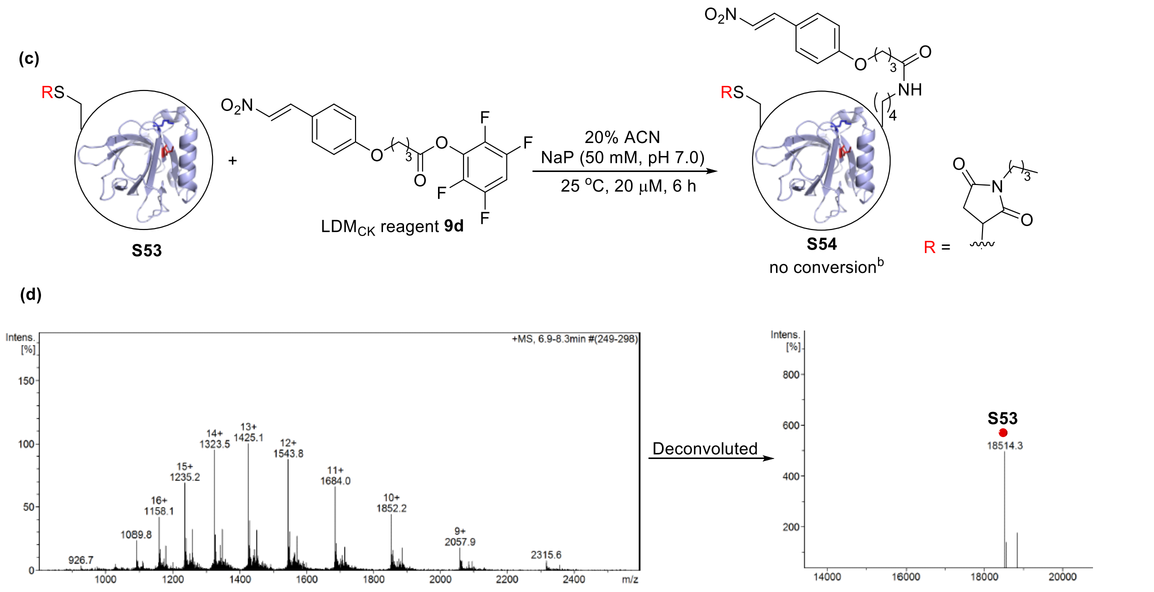

Supplementary Fig. 53. (a) Formation of C-S bond (Cys protection) of β-lactoglobulin A **8a** (1 equiv.) enabled by reagent **S34** (10 equiv.). (b) ESI-MS spectrum mono-labeled β-lactoglobulin A **S53** after C-S bond formation (Cys protection). (c) Acylation of Cys-protected β-lactoglobulin A **S53** with LDM$_{C-K}$ reagent **9d**. (d) ESI-MS spectrum of mono-labeled β-lactoglobulin A **S53** after no acylation with LDM$_{C-K}$ reagent **9d**.

6.3 Acylation of Cysteine in BLG-A

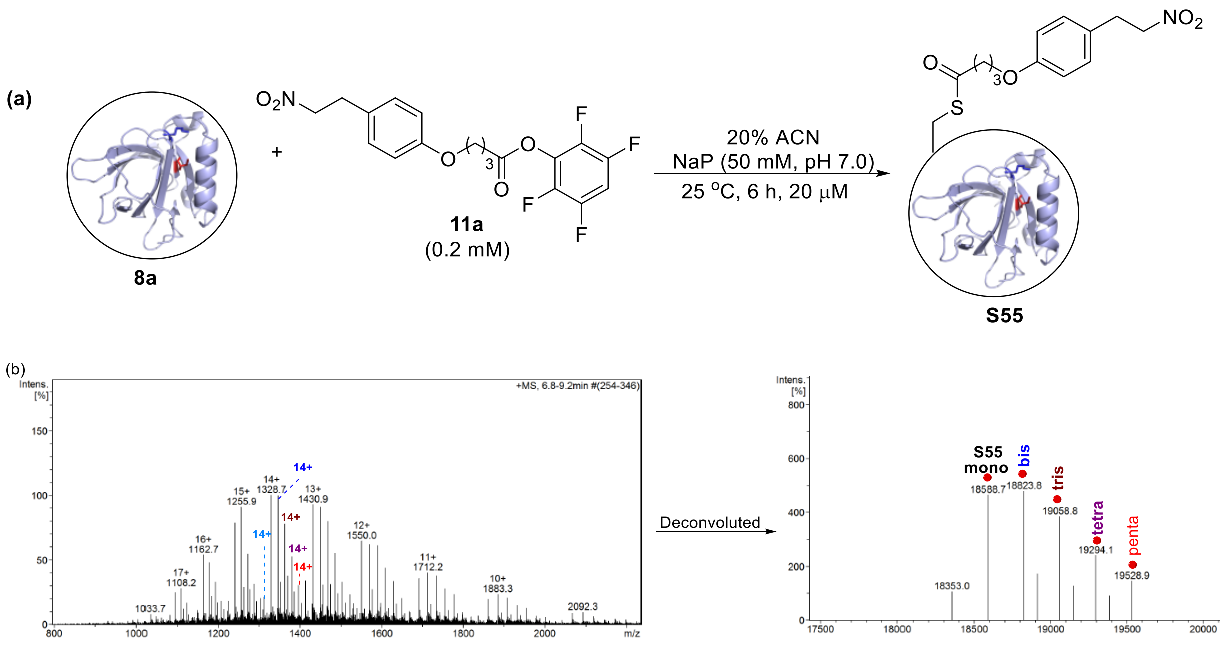

Supplementary Fig. 54. (a) Reaction of β-lactoglobulin A **8a** with control reagent **11a**. (b) ESI-MS spectrum penta-labeled β-lactoglobulin A **S55** from **8a** (1 equiv.) and **11a** (10 equiv.).

6.4 Retro-Michael addition with protein

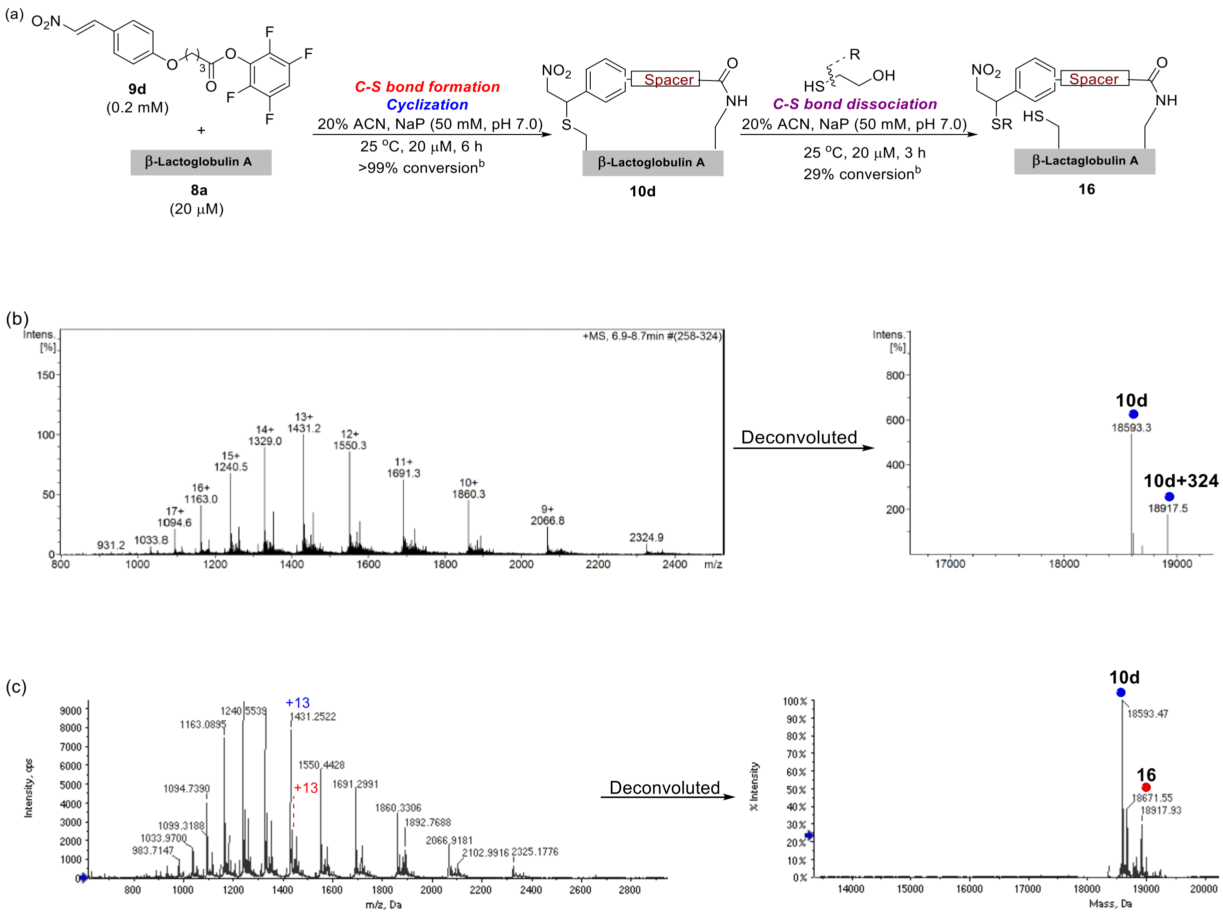

Supplementary Fig. 55. (a) Reaction of LDM$_{C\text{-}K}$ reagent **9d** with β-lactoglobulin A **8a** followed by C-S bond dissociation via retro- Michael reaction. (b) ESI-MS spectrum mono-labeled β-lactoglobulin A **10d** after C-S bond formation and cyclization with a mass impurity of 324 (**10d**+**324**). (c) ESI-MS spectrum of mono-labeled β-lactoglobulin A (**16**, 29% conversion) after C-S bond dissociation via retro-Michael reaction.

6.5 Retro-Henry reaction with protein

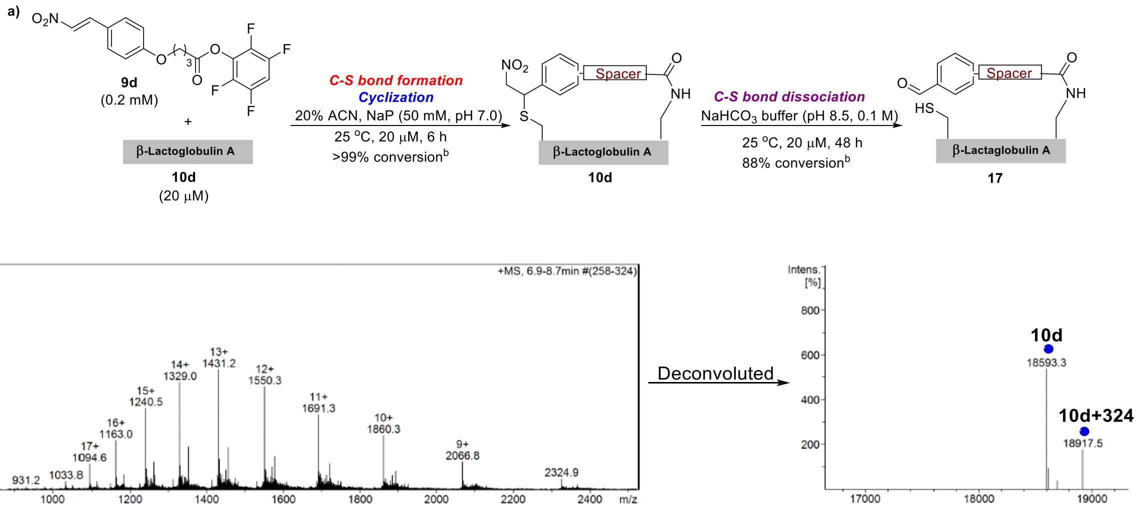

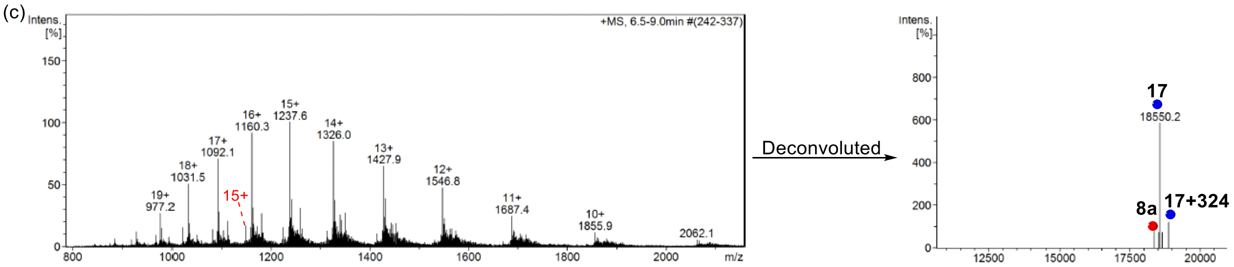

Supplementary Fig. 56. (a) Reaction of LDM$_{C-K}$ reagent **9d** with β-lactoglobulin A **8a** followed by C-S bond dissociation via retro- Henry reaction. (b) ESI-MS spectrum mono-labeled β-lactoglobulin A **10d** with mass impurity of 324 (**10d**+**324**) after C-S bond formation and cyclization. (c) ESI-MS spectrum of mono-labeled β-lactoglobulin A (**17,** 88% conversion) after C-S bond dissociation via retro-Henry reaction.

6.6 Screening of proteins with LDM$_{C-K}$ reagent (9d)

Supplementary Table 6. Screening of proteins with LDM$_{C-K}$ reagent 9d.

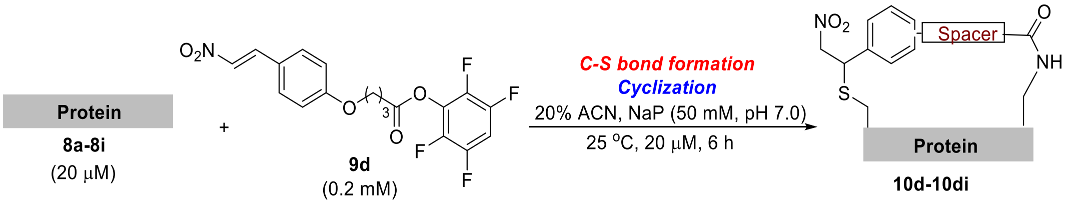

Entry	Proteins	Product (10d-10di)	% Conversion[a]
**1[b]**	**β-Lactaglobulin A (8a)**	**10d**	**>99 (m)**
2	Lysozyme C (**8b**)	**10db**	0
3	Myoglobin (**8c**)	**10dc**	0
4	Cytochrome C (**8d**)	**10dd**	0
5	Insulin (**8f**)	**10df**	0
6	Ubiquitin (**8g**)	**10dg**	0
7	RNase A (**8h**)	**10dh**	0
8	Aprotinin (**8i**)	**10di**	0

[a] % Conversion based on ESI-MS. [b] m-mono-labeled β-lactoglobulin A **10d** with reagent **9d**.

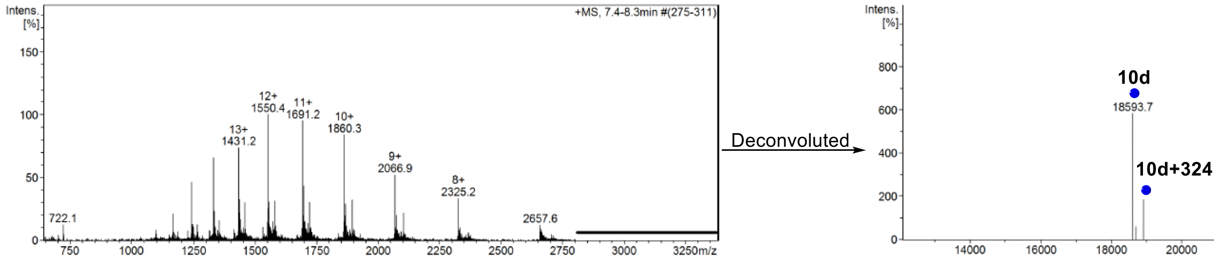

Supplementary Fig. 57. ESI-MS spectrum of mono-labeled β-lactoglobulin A **10d** after the reaction of β-lactoglobulin A **8a** with LDM$_{C-K}$ reagent **9d**.

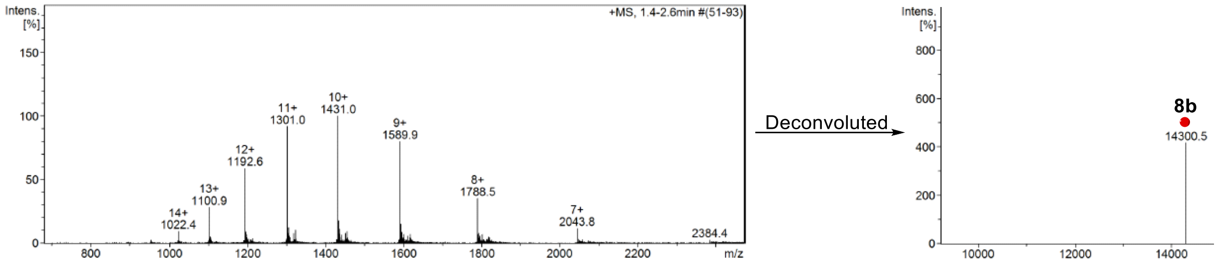

Supplementary Fig. 58. ESI-MS spectrum of Lysozyme C **8b** after no reaction with LDM$_{C\text{-}K}$ reagent **9d**.

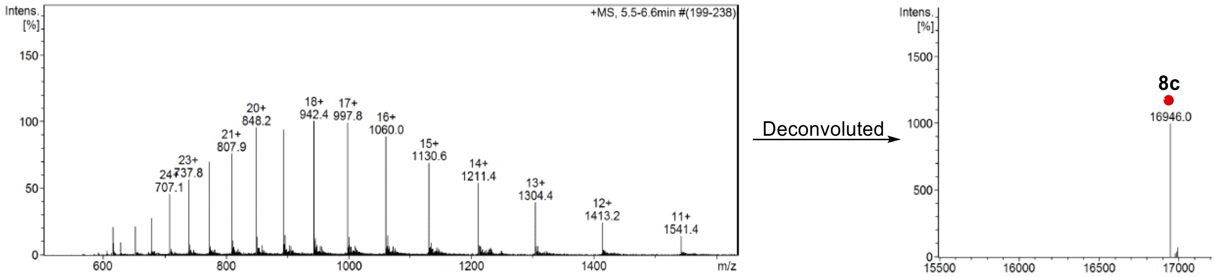

Supplementary Fig. 59. ESI-MS spectrum of Myoglobin **8c** after no reaction with LDM$_{C\text{-}K}$ reagent **9d**.

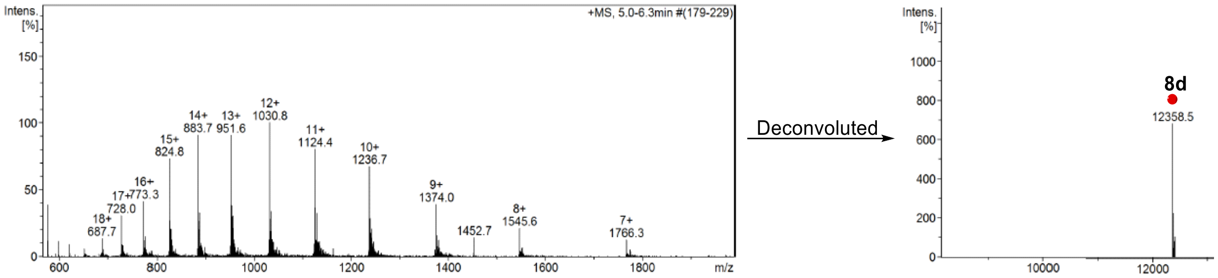

Supplementary Fig. 60. ESI-MS spectrum of Cytochrome C **8d** after no reaction with LDM$_{C\text{-}K}$ reagent **9d**.

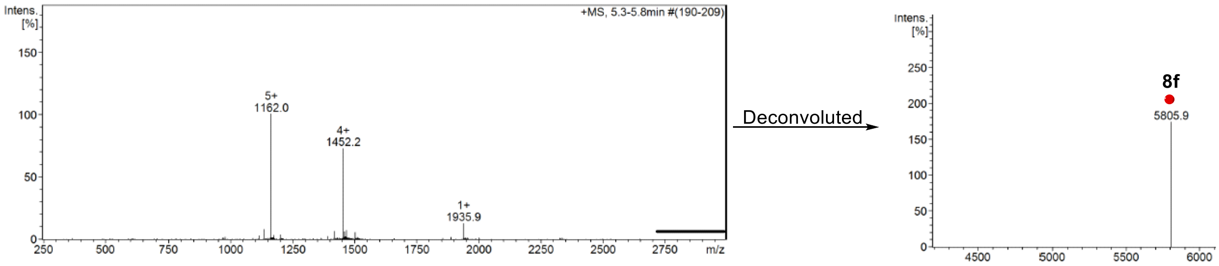

Supplementary Fig. 61. ESI-MS spectrum of Insulin **8f** after no reaction with LDM$_{C\text{-}K}$ reagent **9d**.

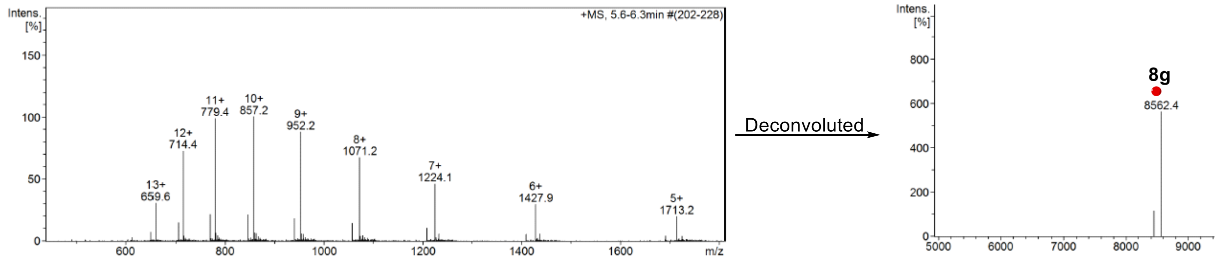

Supplementary Fig. 62. ESI-MS spectrum of Ubiquitin **8g** after no reaction with LDM$_{C\text{-}K}$ reagent **9d**.

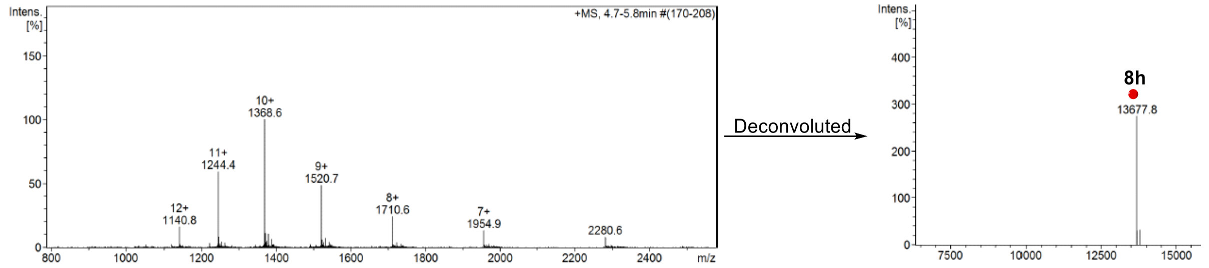

Supplementary Fig. 63. ESI-MS spectrum of RNase A **8h** after no reaction with LDM_{C-K} reagent **9d**.

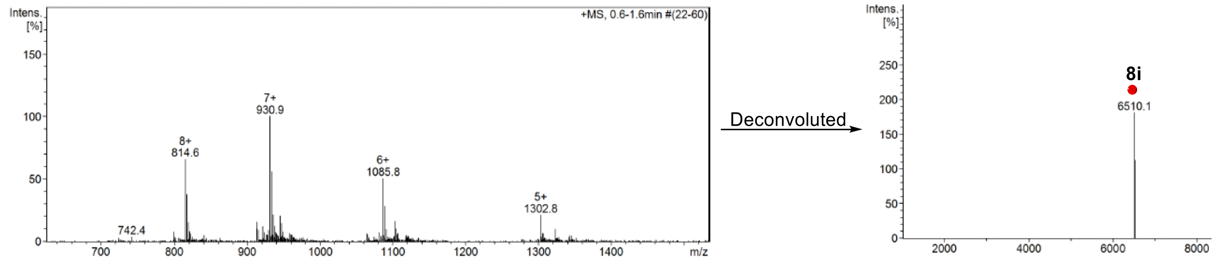

Supplementary Fig. 64. ESI-MS spectrum of Aprotinin **8i** after no reaction with LDM_{C-K} reagent **9d**.

6.7 Kinetics of C-S bond formation and cyclization

Supplementary Table 7. Time-wise monitoring of C-S bond formation and cyclization with reagent 9d.

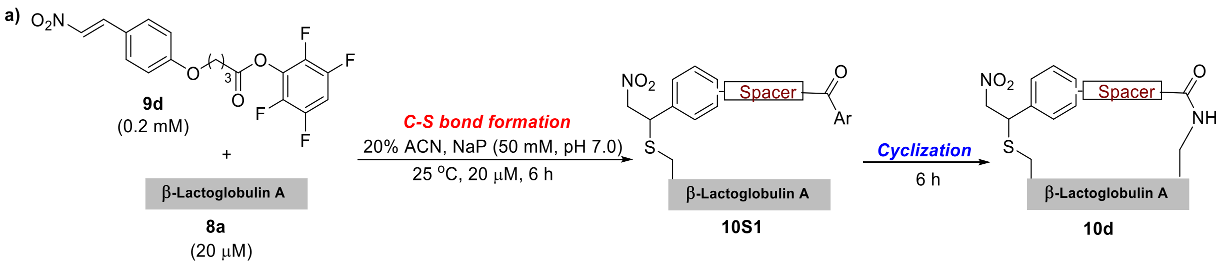

Entry	Time	% Conversion[a, b] Michael adduct (10S1)			% Conversion[a, b] Cyclization (10d)		
		I	II	III	I	II	III
1	10 sec	0	6	10	0	0	0
2	30 sec	26	19	20	4	0	0
3	1 min	18	19	19	5	0	0
4	2 min	40	44	39	18	18	20
5	5 min	26	21	24	29	25	24
6	10 min	27	16	21	49	31	41
7	30 min	12	11	15	33	42	58
8	60 min	7	6	4	68	71	57
9	3 h	0	6	6	80	79	78
10	6 h	0	0	0	92	>99	>99

[a] % Conversion based on ESI-MS. [b] This experiment was done in triplicate (I-III).

(b)

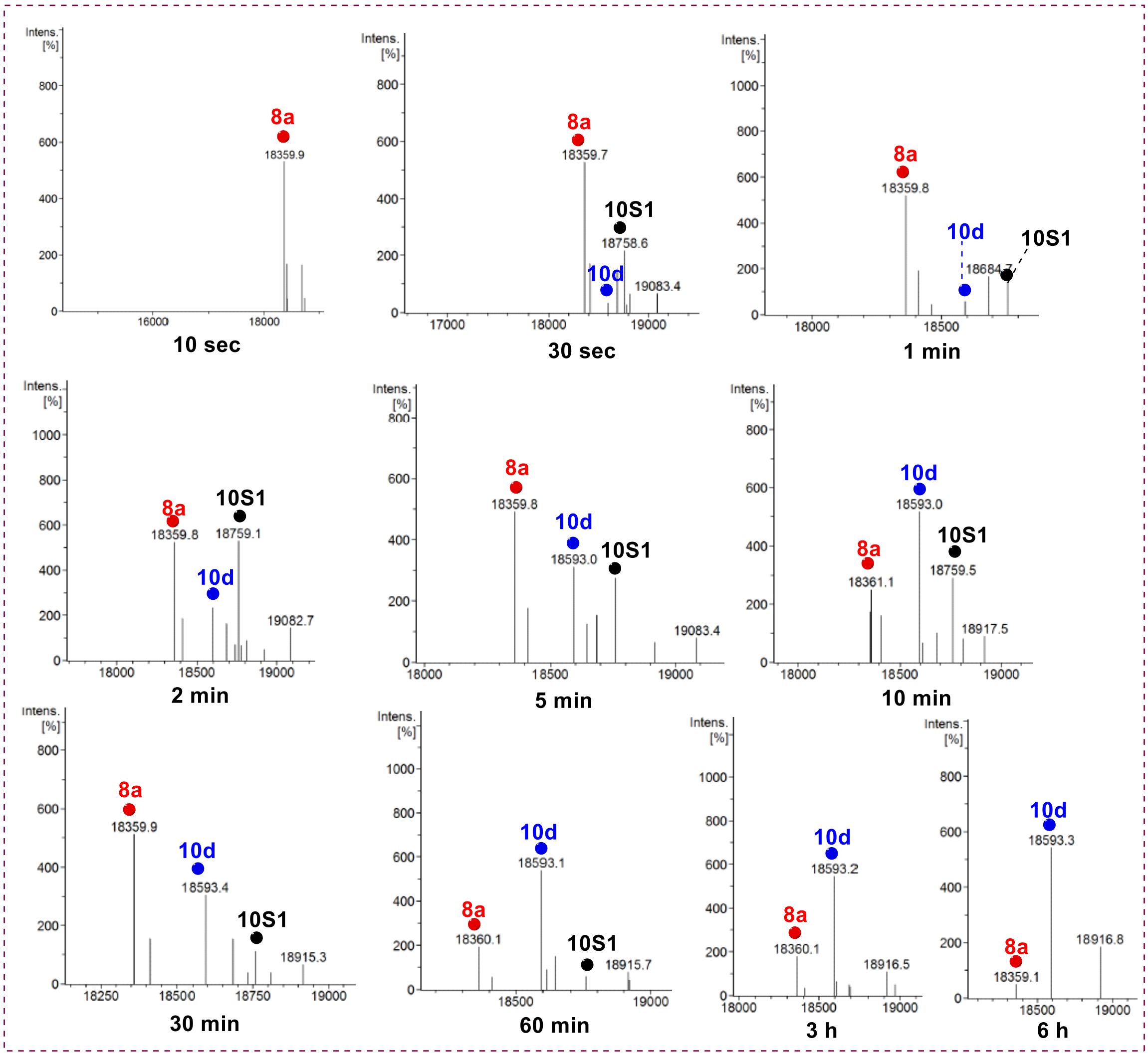

Supplementary Fig. 65. (a) Time-wise monitoring of C-S bond formation and cyclization with LDM$_{CK}$ reagent **9d**. (b) ESI-MS deconvoluted spectrum for various time interval of C-S bond formation and cyclization for β-lactoglobulin A **8a** with LDM$_{C-K}$ reagent **9d** (for trial I).

6.8 Single-site labeling of native proteins

(a)

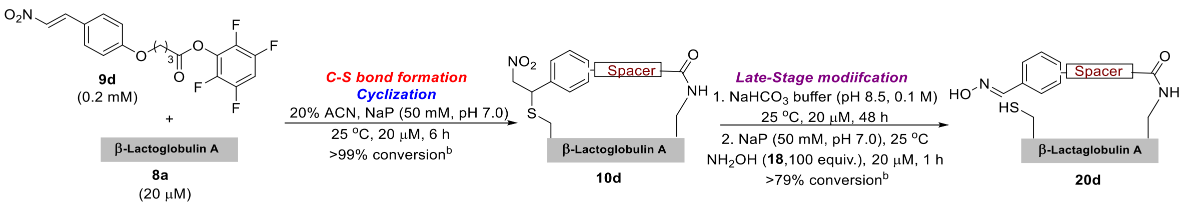

(b)

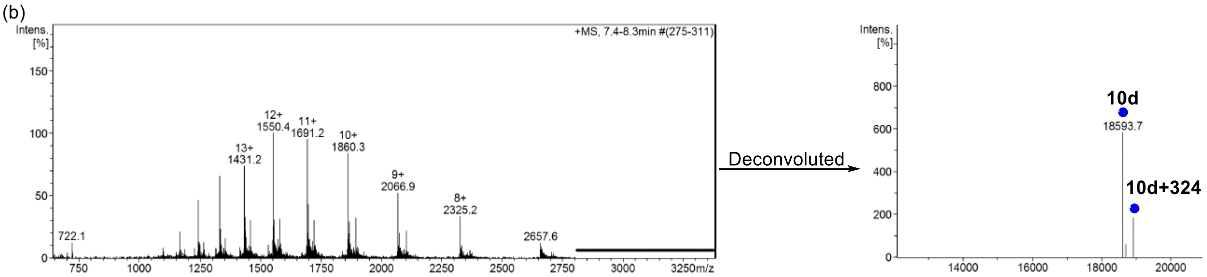

(c)

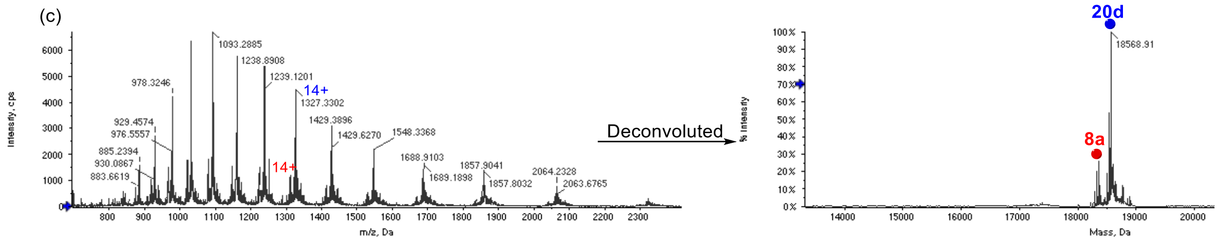
Intensity, cps
1093.2885
1238.8908
1239.1201
978.3246
14+
1327.3302
929.4574
976.5557
885.2394
930.0867
883.6619
1429.3896
1429.6270
1548.3368
1688.9103
1689.1898
1857.9041
1857.8032
2064.2328
2063.6765
14+
m/z, Da
Deconvoluted
% Intensity
20d
18568.91
8a
Mass, Da

(d)

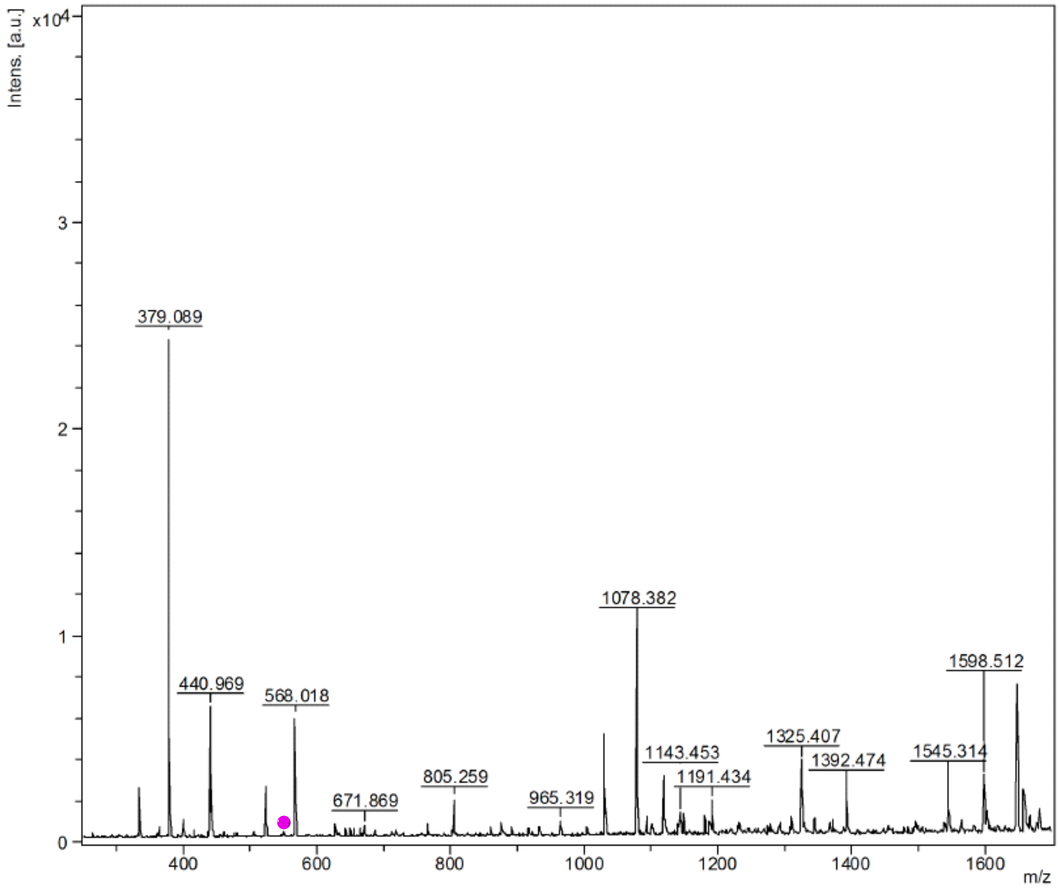
Intens. [a.u.]
x10^4
379.089
440.969
568.018
671.869
805.259
965.319
1078.382
1143.453
1191.434
1325.407
1392.474
1545.314
1598.512
m/z

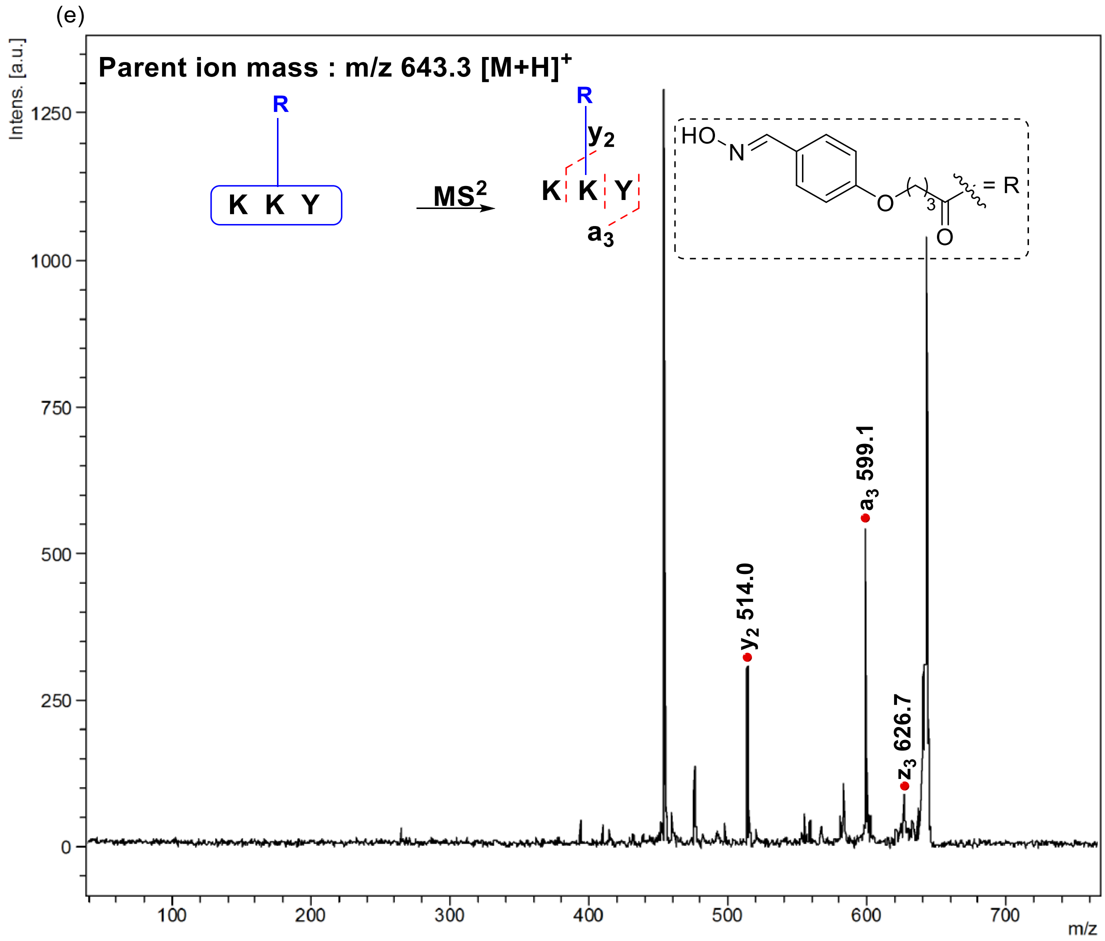

Supplementary Fig. 66. (a) Site-selective labeling of β-lactoglobulin A **8a** (1 equiv.) enabled by LDM_{CK} reagent **9d** (10 equiv.). (b) ESI-MS spectrum for mono-labeled β-lactoglobulin A **10d** after cyclization. (c) ESI-MS spectrum for mono-labeled β-lactoglobulin A **20d** after oxime formation. (d) Peptide mapping of **20d** after the digestion with α-chymotrypsin. (e) MS-MS spectrum of labeled KKY (K100-Y102, *m/z* 643.3 $[M+H]^+$). The site of modification in mono-labeled β-lactoglobulin A **20d** is K101.

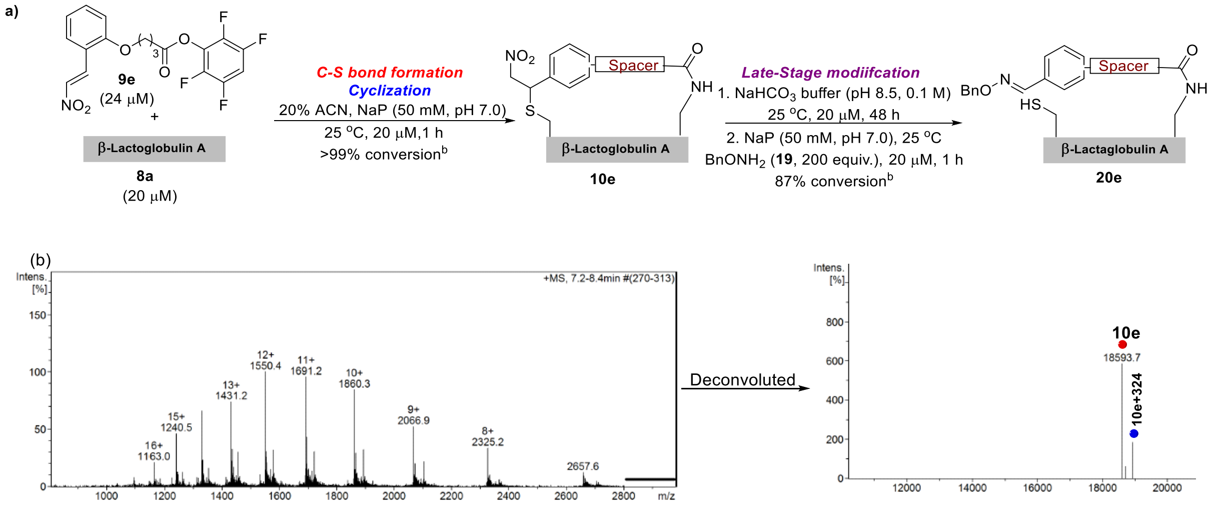

(c)

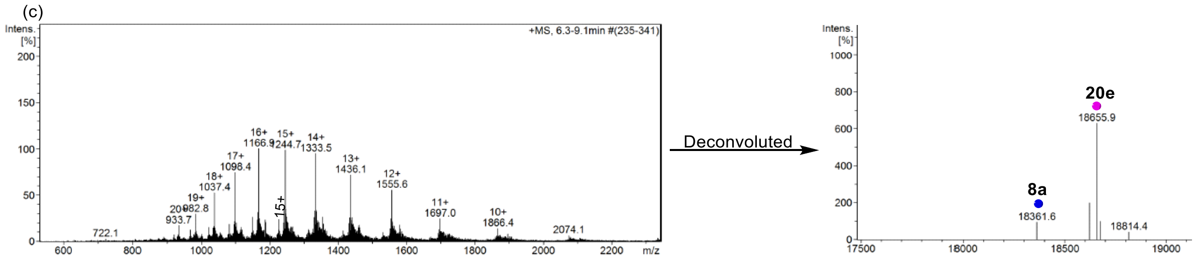
Intens. [%]
+MS, 6.3-9.1min #(235-341)
20+ 933.7
19+ 982.8
18+ 1037.4
17+ 1098.4
16+ 1166.9
15+ 1244.7
14+ 1333.5
13+ 1436.1
12+ 1555.6
11+ 1697.0
10+ 1866.4
15+
722.1
2074.1
m/z
Deconvoluted
Intens. [%]
20e
18655.9
8a
18361.6
18814.4

(d)

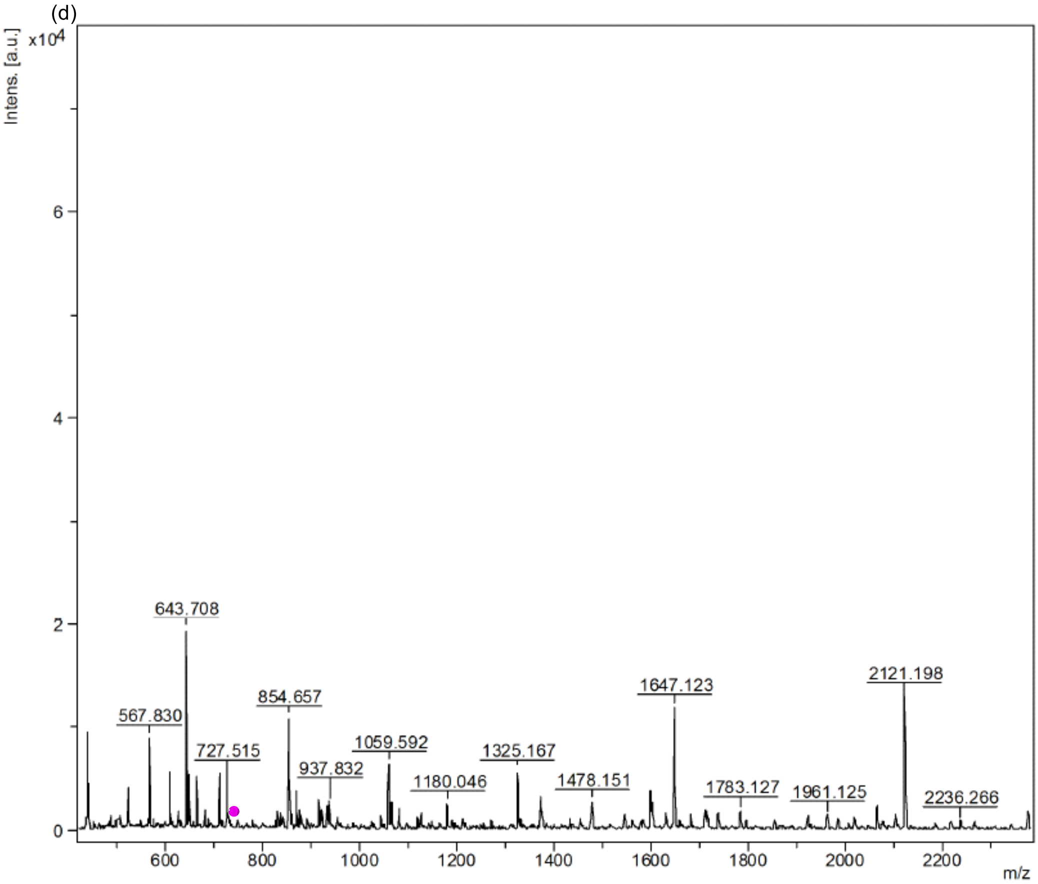
Intens. [a.u.]
x10^4
643.708
567.830
727.515
854.657
937.832
1059.592
1180.046
1325.167
1478.151
1647.123
1783.127
1961.125
2121.198
2236.266
m/z

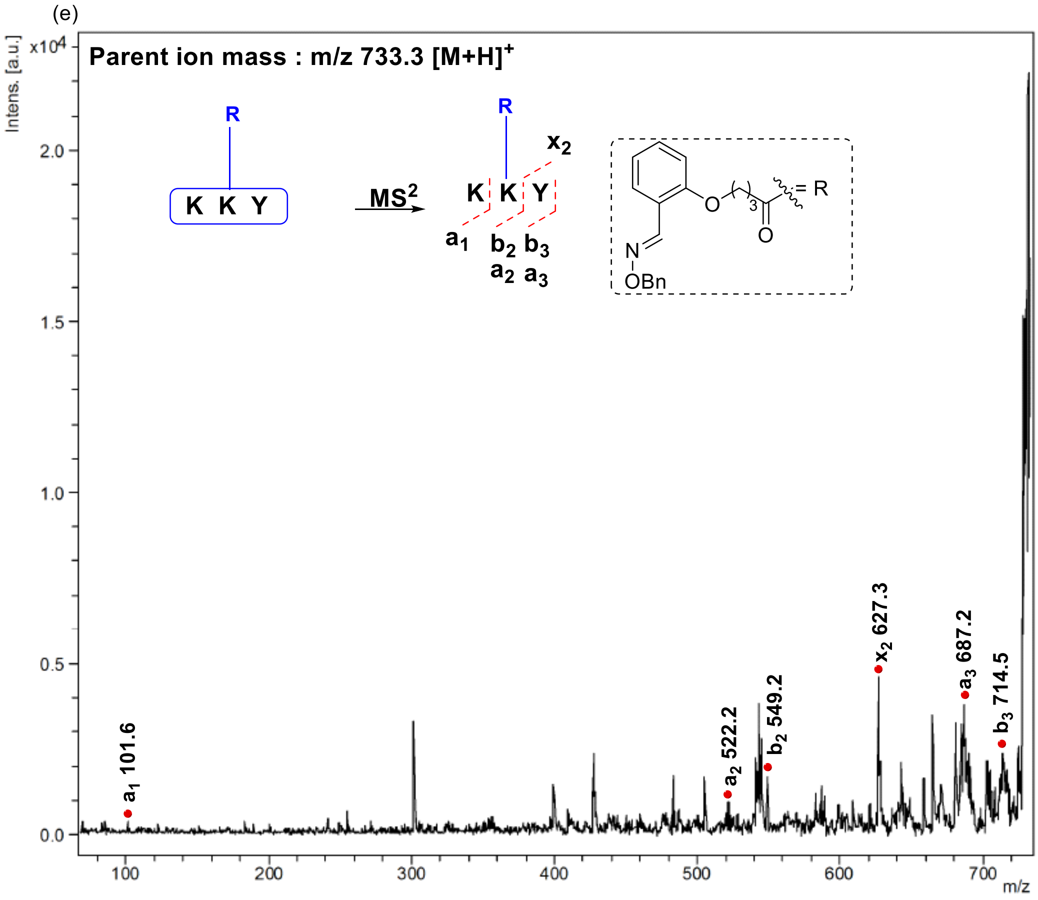

Supplementary Fig. 67. (a) Site-selective labeling of β-lactoglobulin A **8a** (1 equiv.) enabled by LDM_{CK} reagent **9e** (1.2 equiv.). (b) ESI-MS spectrum for mono-labeled β-lactoglobulin A **10e** after cyclization with mass impurity of 324 (**10e**+**324**). (c) ESI-MS spectrum for mono-labeled β-lactoglobulin A **20e** after oxime formation. (d) Peptide mapping of **20e** after the digestion with α-chymotrypsin. (e) MS-MS spectrum of labeled KKY (K100-Y102, *m/z* 733.3 $[M+H]^+$). The site of modification in mono-labeled β-lactoglobulin A **20e** is K101.

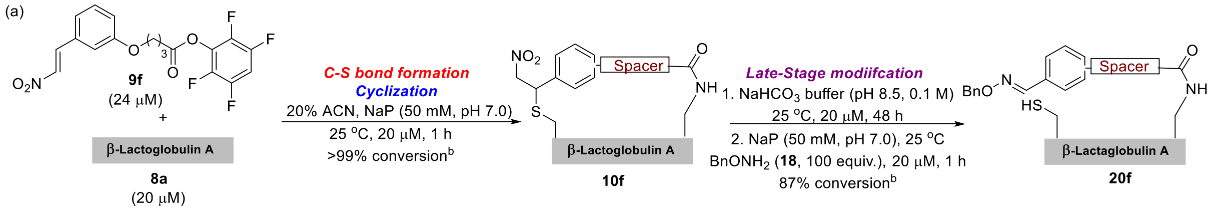

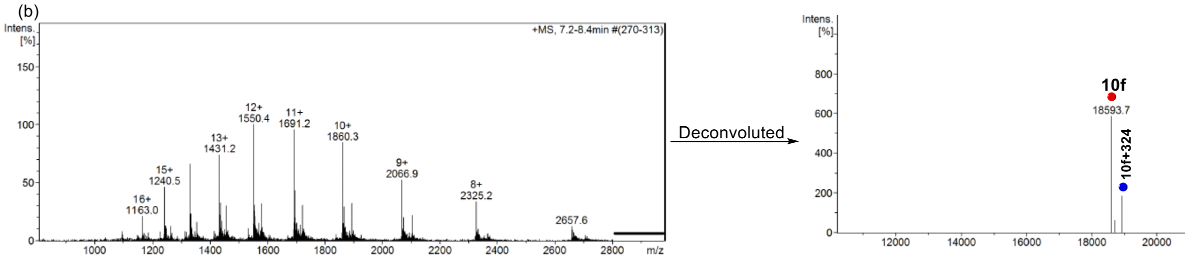

(c)

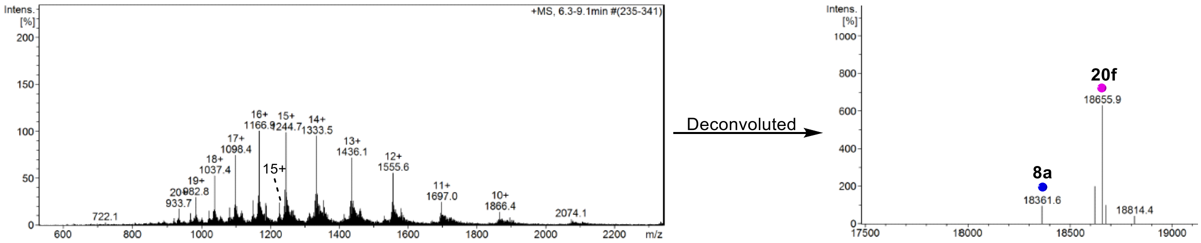
Intens. [%]
+MS, 6.3-9.1min #(235-341)
16+ 1166.9
15+ 1244.7
14+ 1333.5
17+ 1098.4
13+ 1436.1
18+ 1037.4
12+ 1555.6
15+
19+ 982.8
20+ 933.7
11+ 1697.0
10+ 1866.4
722.1
2074.1
m/z
Deconvoluted
20f
18655.9
8a
18361.6
18814.4

(d)

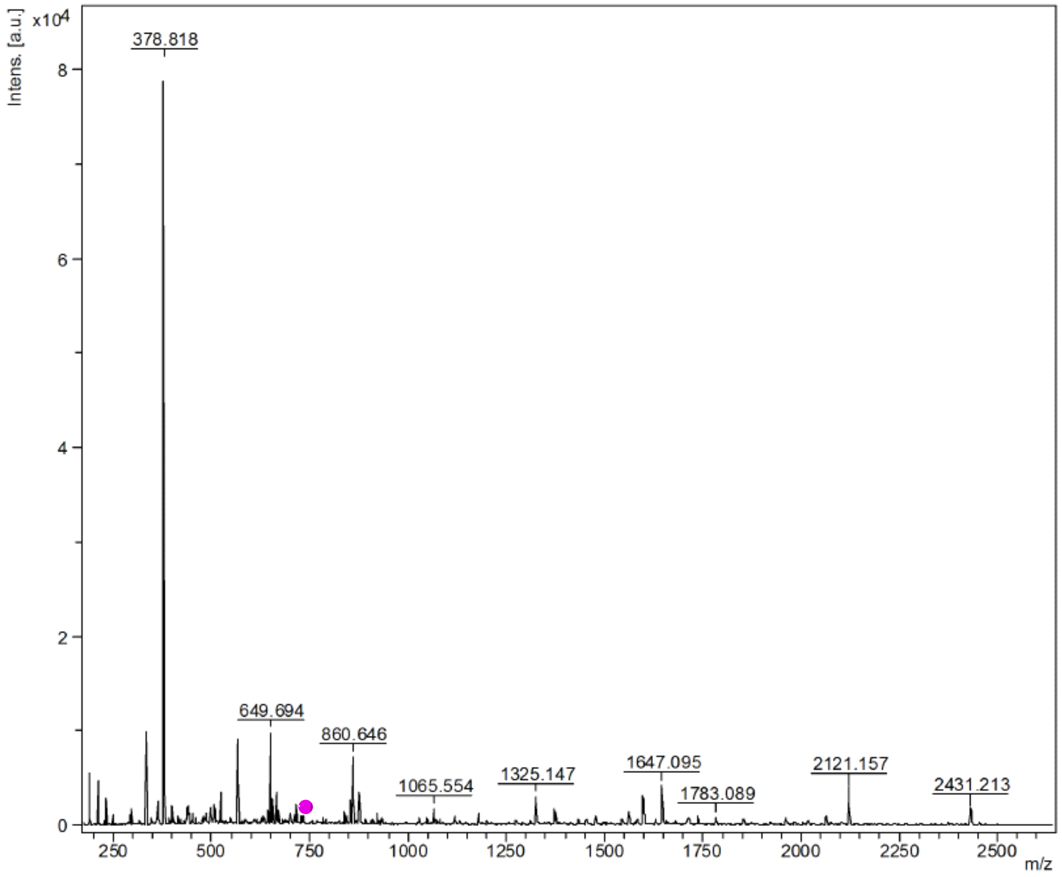
Intens. [a.u.]
x10^4
378.818
649.694
860.646
1065.554
1325.147
1647.095
1783.089
2121.157
2431.213
m/z

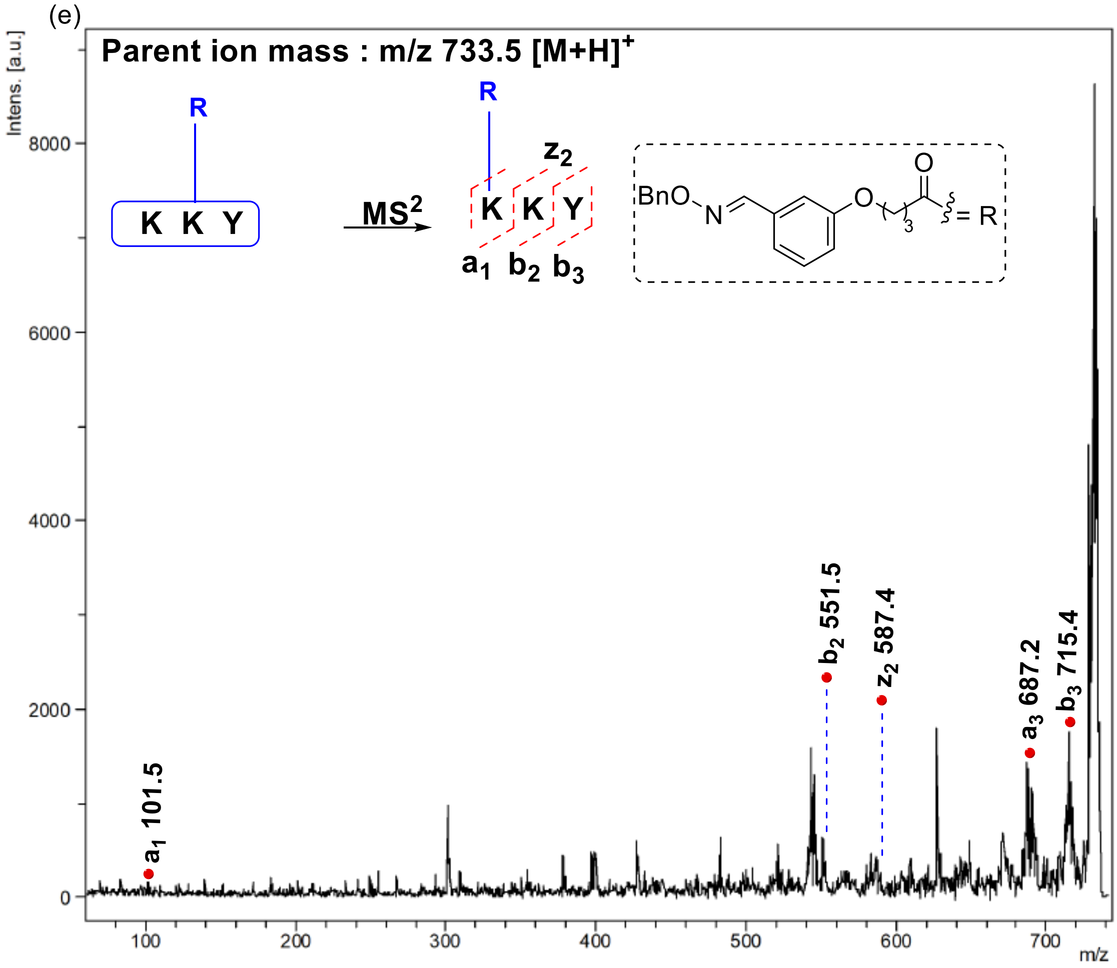

Supplementary Fig. 68. (a) Site-selective labeling of β-lactoglobulin A **8a** (1 equiv.) enabled by LDM_{CK} reagent **9f** (1.2 equiv.). (b) ESI-MS spectrum for mono-labeled β-lactoglobulin A **10f** after cyclization with mass impurity of 324 (**10f**+**324**). (c) ESI-MS spectrum for mono-labeled β-lactoglobulin A **20f** after oxime formation. (d) Peptide mapping of **20f** after the digestion with α-chymotrypsin. (e) MS-MS spectrum of labeled KKY (K100-Y102, *m/z* 733.3 $[M+H]^+$). The site of modification in mono-labeled β-lactoglobulin A **20f** is K101.

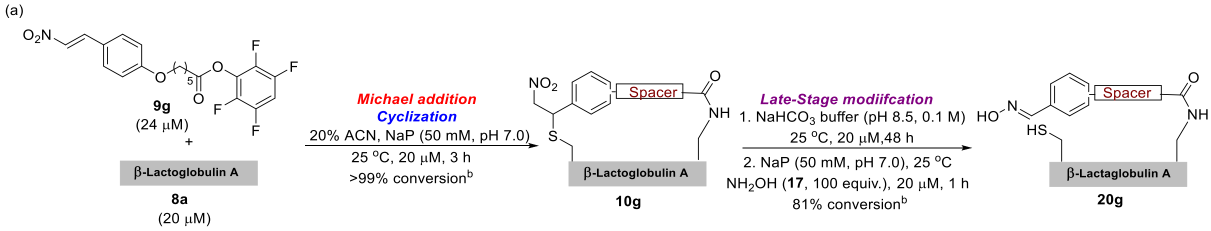

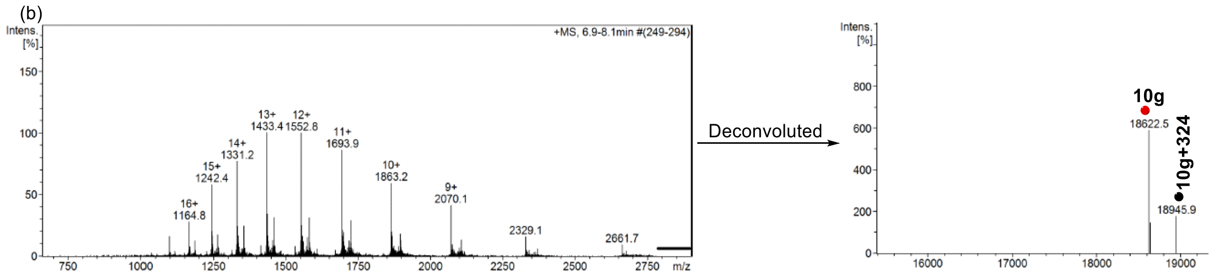

(c)

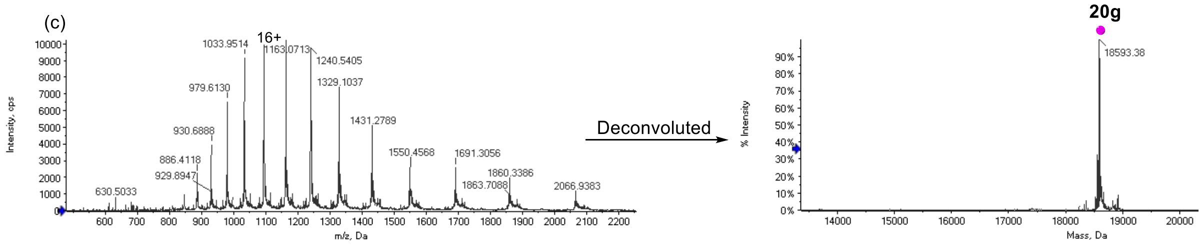

16+
1033.9514
1163.0713
1240.5405
979.6130
1329.1037
930.6888
1431.2789
886.4118
929.8947
1550.4568
1691.3056
1860.3386
1863.7088
630.5033
2066.9383
Intensity, cps
m/z, Da
Deconvoluted
20g
18593.38
% Intensity
Mass, Da

(d)

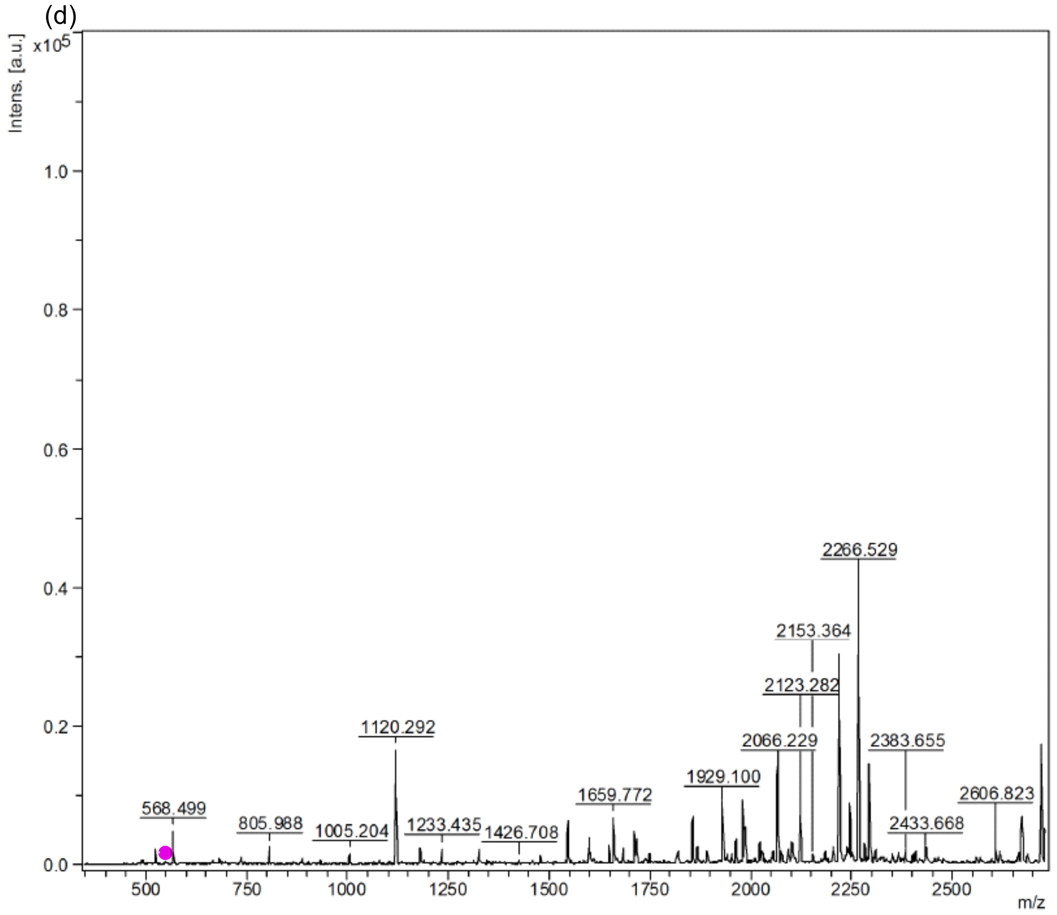

Intens. [a.u.]
x10^5
2266.529
2153.364
2123.282
1120.292
2066.229
2383.655
1929.100
2606.823
1659.772
568.499
805.988
1233.435
2433.668
1005.204
1426.708
m/z

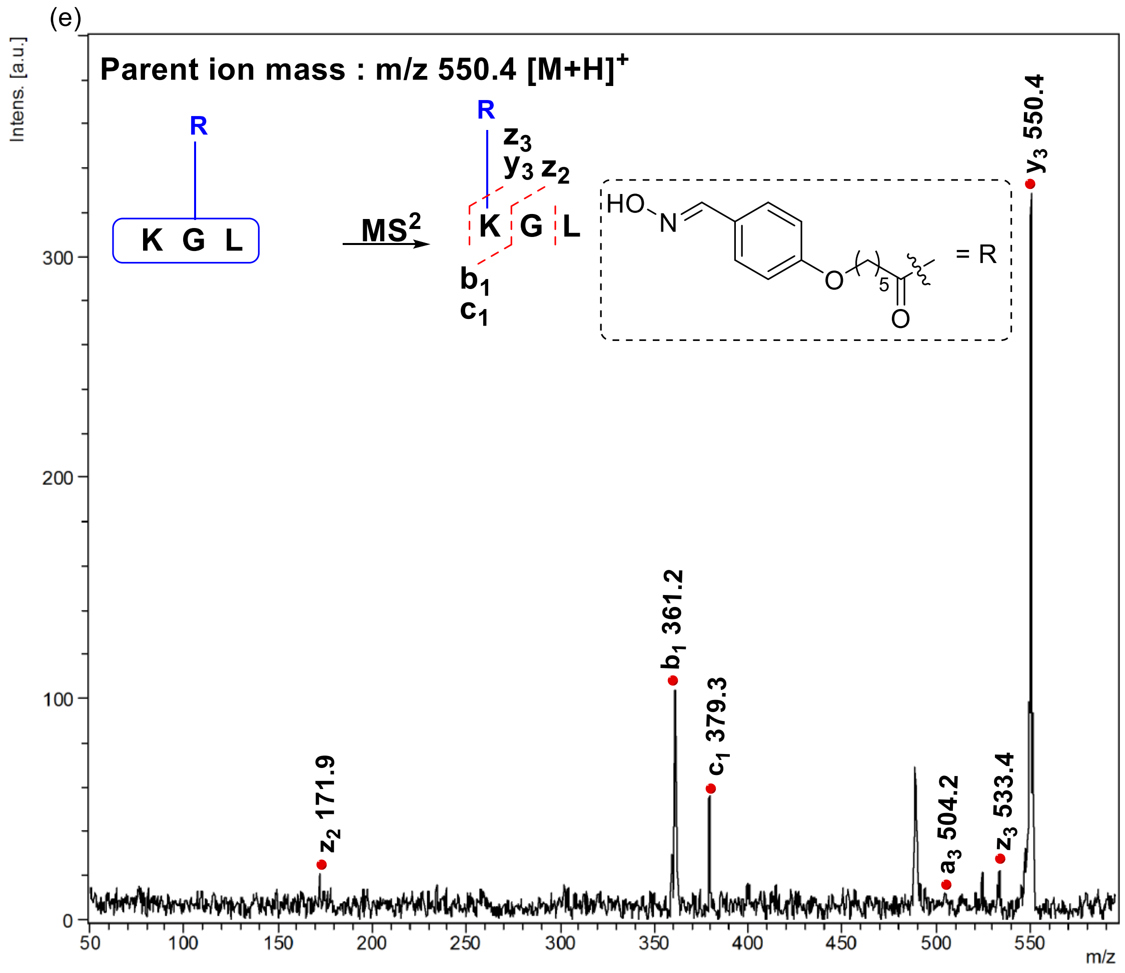

Supplementary Fig. 69. (a) Site-selective labeling of β-lactoglobulin A **8a** (1 equiv.) enabled by LDM$_{CK}$ reagent **9g** (1.2 equiv.). (b) ESI-MS spectrum for mono-labeled β-lactoglobulin A **10g** after cyclization with mass impurity of 324 (**10g**+**324**). (c) ESI-MS spectrum for mono-labeled β-lactoglobulin A **20g** after oxime formation. (d) Peptide mapping of **20g** after the digestion with α-chymotrypsin. (e) MS-MS spectrum of labeled KGL (K8-L10, *m/z* 550.4 [M+H]$^+$). The site of modification in mono-labeled β-lactoglobulin A **20g** is K8.

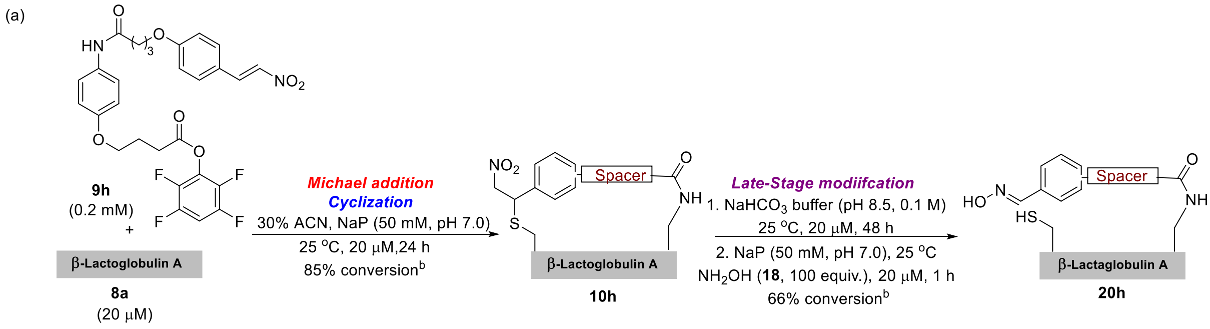

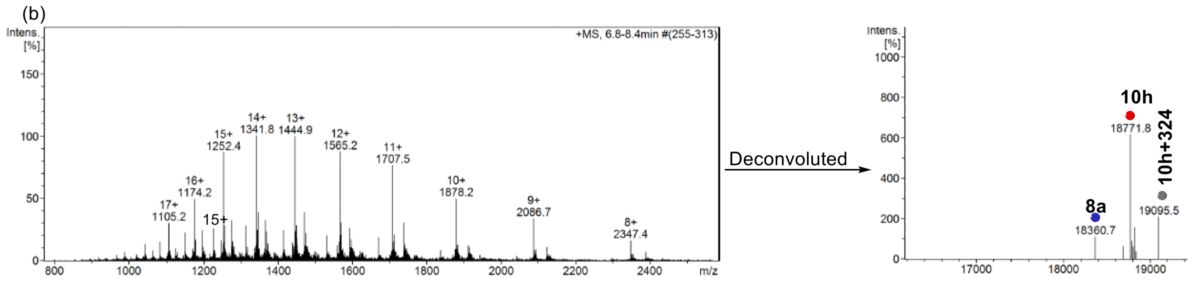

(c)

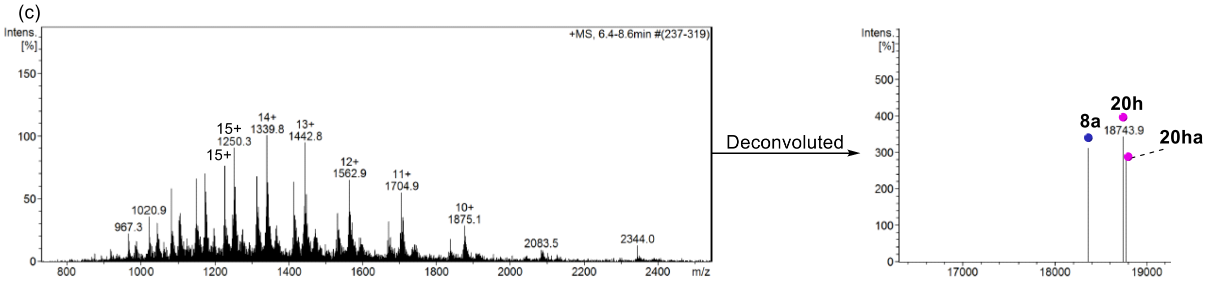

(d)

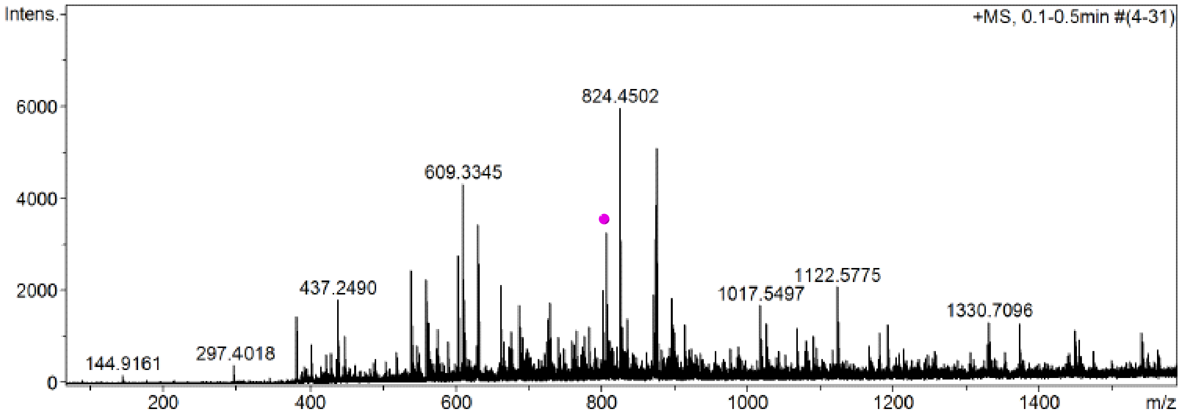

(e)

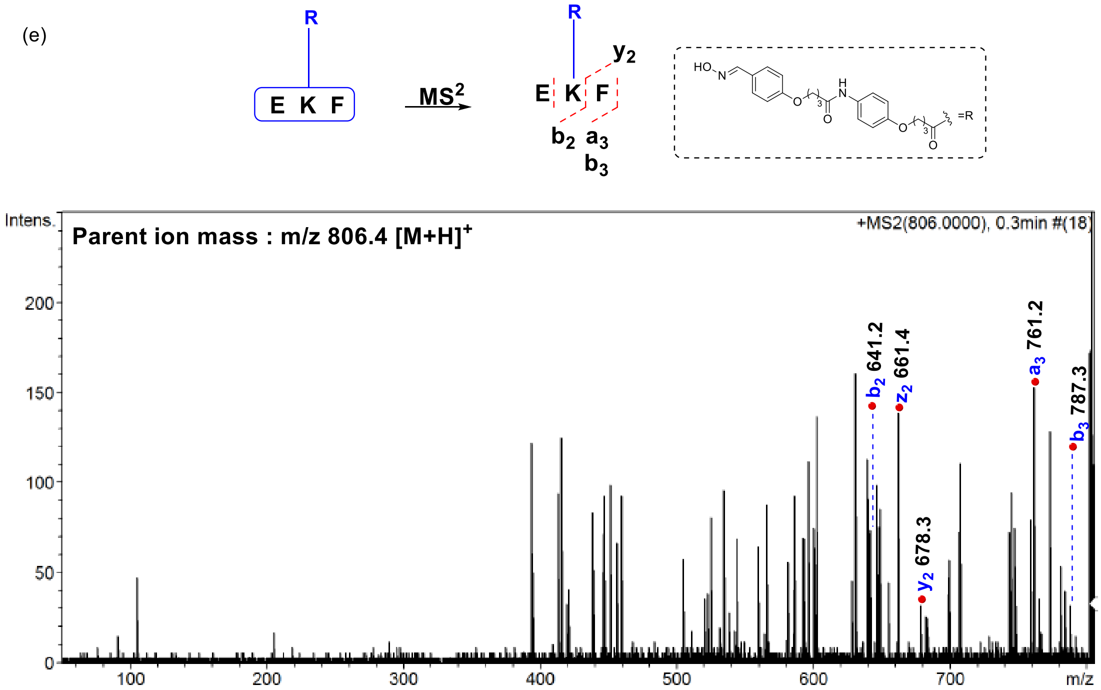

Supplementary Fig. 70. (a) Site-selective labeling of β-lactoglobulin A **8a** (1 equiv.) enabled by LDM_{CK} reagent **9h** (10 equiv.). (b) ESI-MS spectrum for mono-labeled β-lactoglobulin A **10h** after cyclization with mass impurity of 324 (**10h**+**324**). (c) ESI-MS spectrum for mono-labeled β-lactoglobulin A **20h (**with oxidised product **20ha)** after oxime formation. (d) Peptide mapping of **20h** after the digestion with α-chymotrypsin. (e) MS-MS spectrum of labeled EKF (E134-F136, *m/z* 806.4 $[M+H]^+$). The site of modification in mono-labeled β-lactoglobulin A **20h** is K135.

6.9 Late-stage modification

(a)

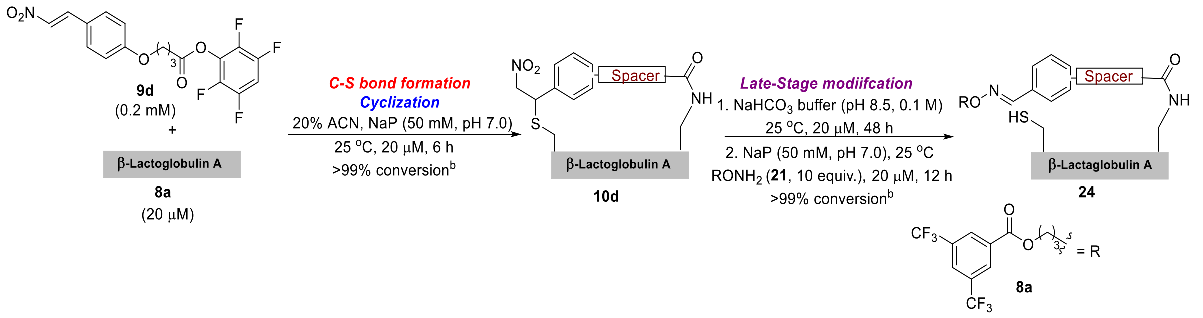

(b)

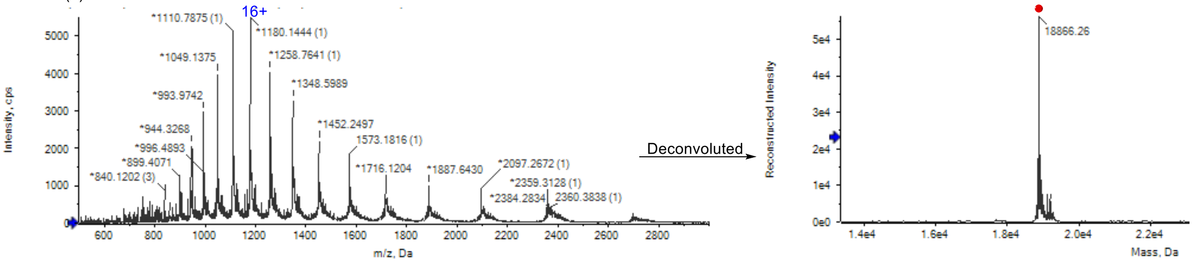

(c)

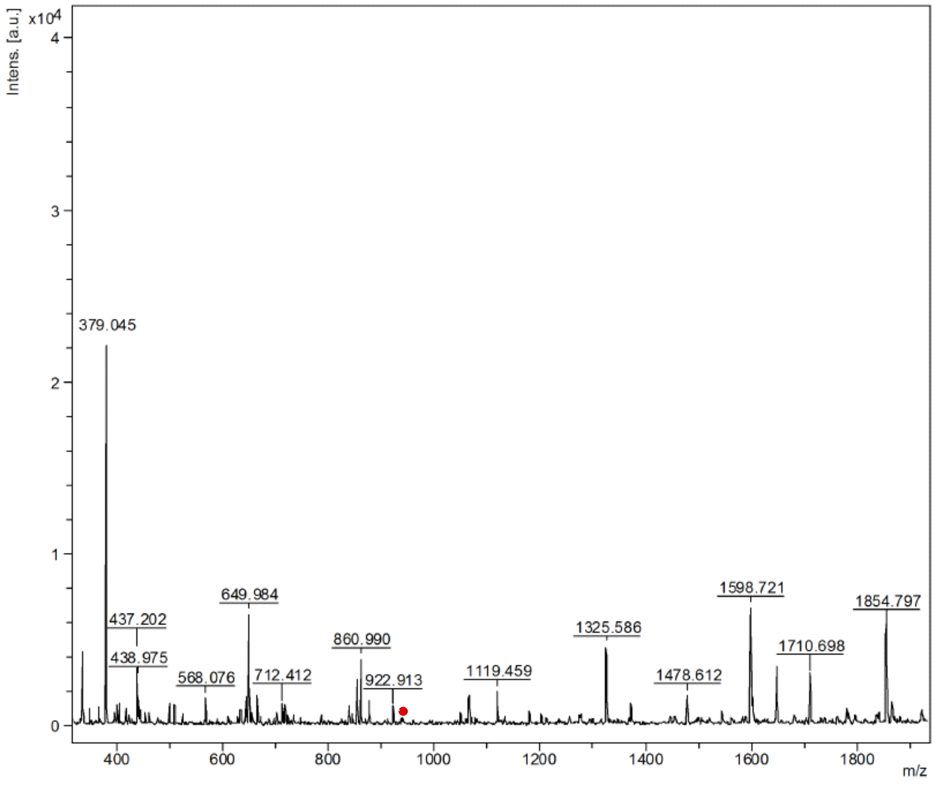

(d)

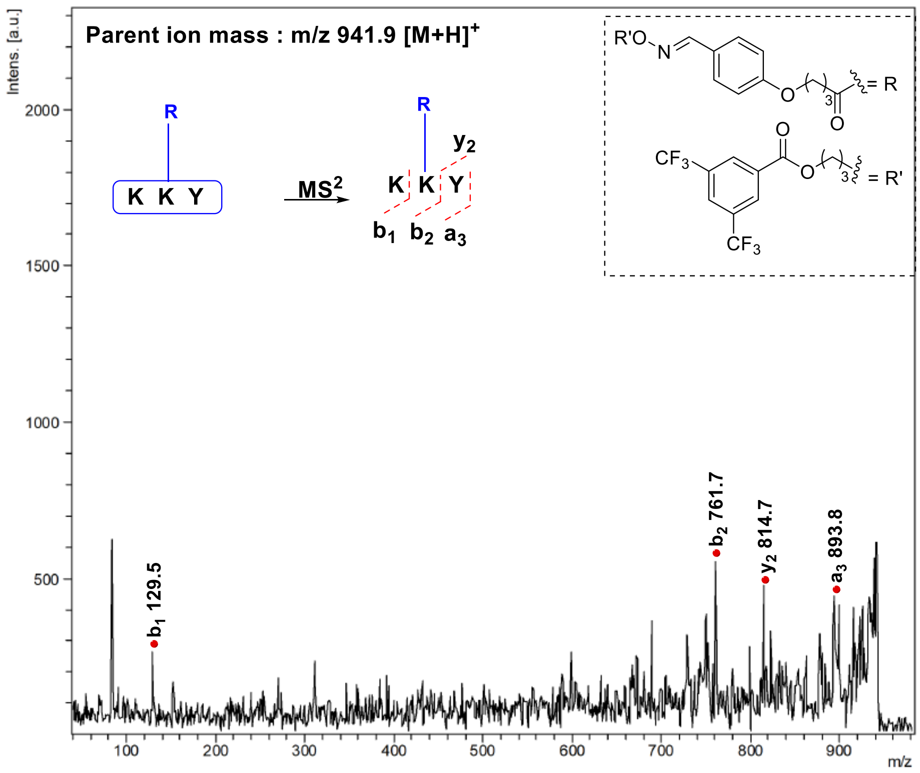

Supplementary Fig. 71. (a) Site-selective labeling of β-lactoglobulin A **8a** (1 equiv.) enabled by LDM$_{CK}$ reagent **9d** (10 equiv.) followed by oxime formation with reagent **21**. (b) ESI-MS spectrum for mono-labeled β-lactoglobulin A (**24**, >99% conversion**)**. (c) Peptide mapping of **24** after the digestion with α-chymotrypsin. (d) MS-MS spectrum of labeled KKY (K100-K102, *m/z* 941.9 [M+H]$^+$). The site of modification in mono-labeled β-lactoglobulin A **24** is K101.

(a)

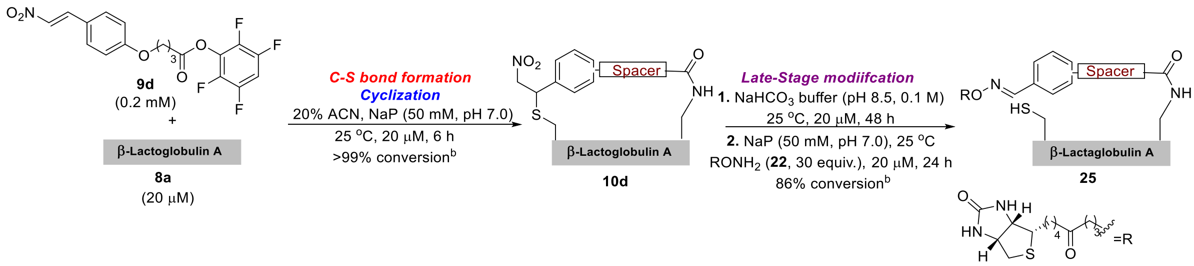

(b)

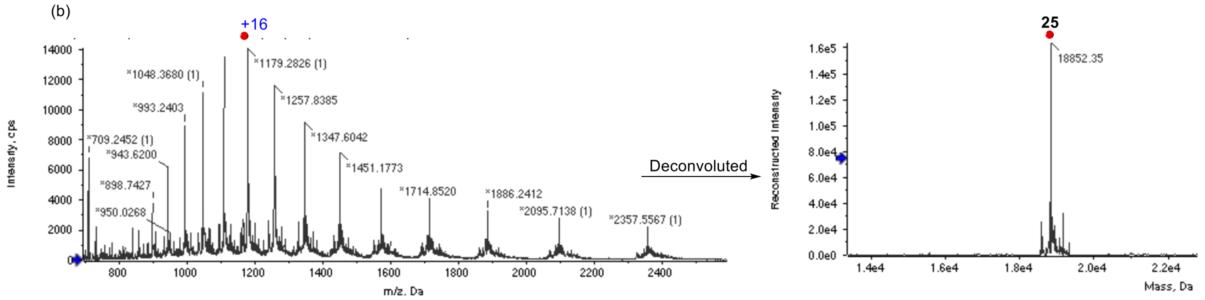

(c)

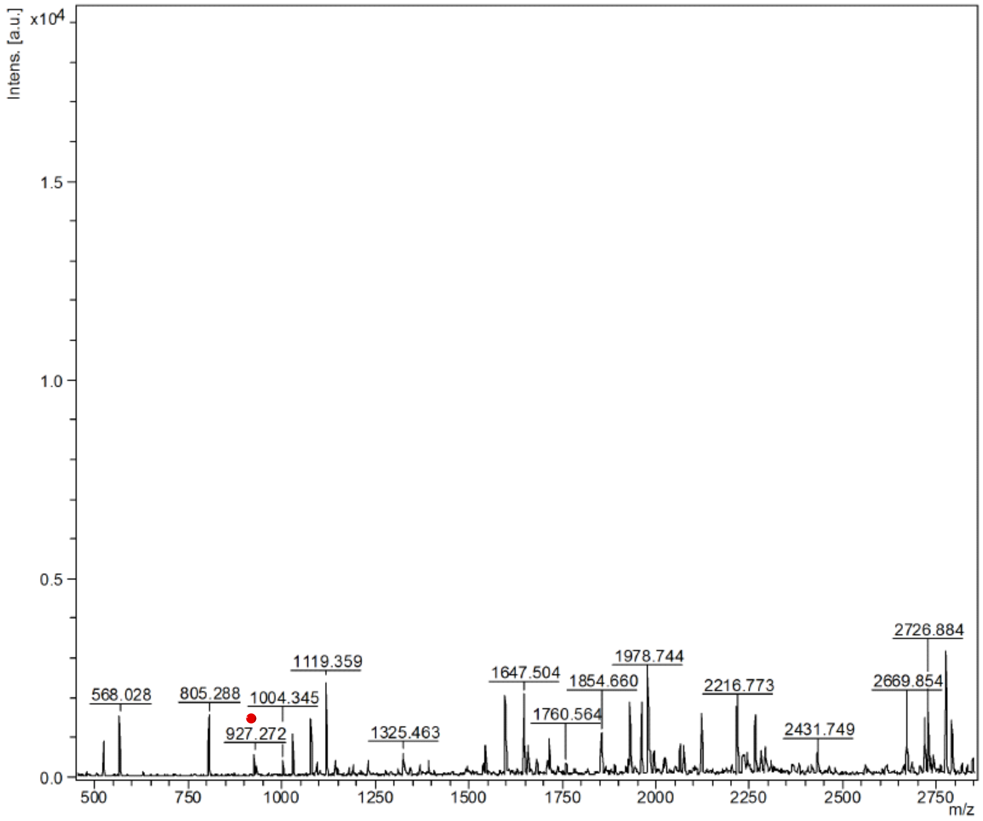

(d)

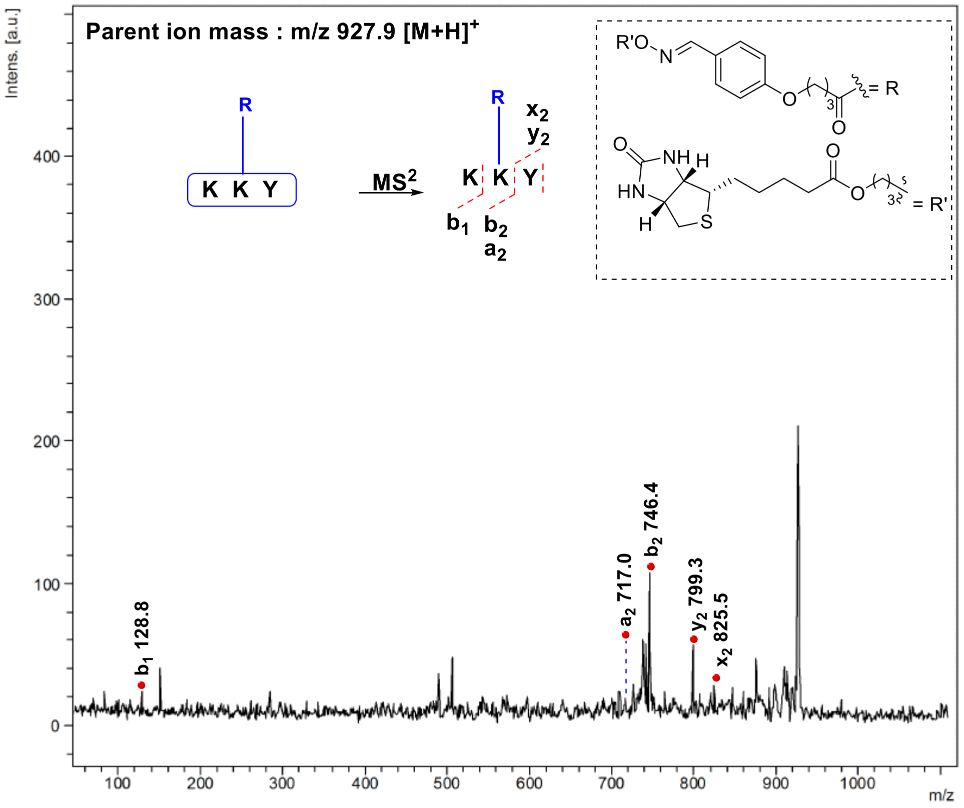

Supplementary Fig. 72. (a) Site-selective labeling of β-lactoglobulin A **8a** (1 equiv.) enabled by LDM$_{CK}$ reagent **9d** (10 equiv.) followed by oxime formation with reagent **22**. (b) ESI-MS spectrum for mono-labeled β-lactoglobulin A (**25**, 86% conversion**)**. (c) Peptide mapping of **25** after the digestion

with α-chymotrypsin. (c) MS-MS spectrum of labeled KKY (K100-K102, *m/z* 927.9 $[M+H]^+$). The site of modification in mono-labeled β-lactoglobulin A **25** is K101.

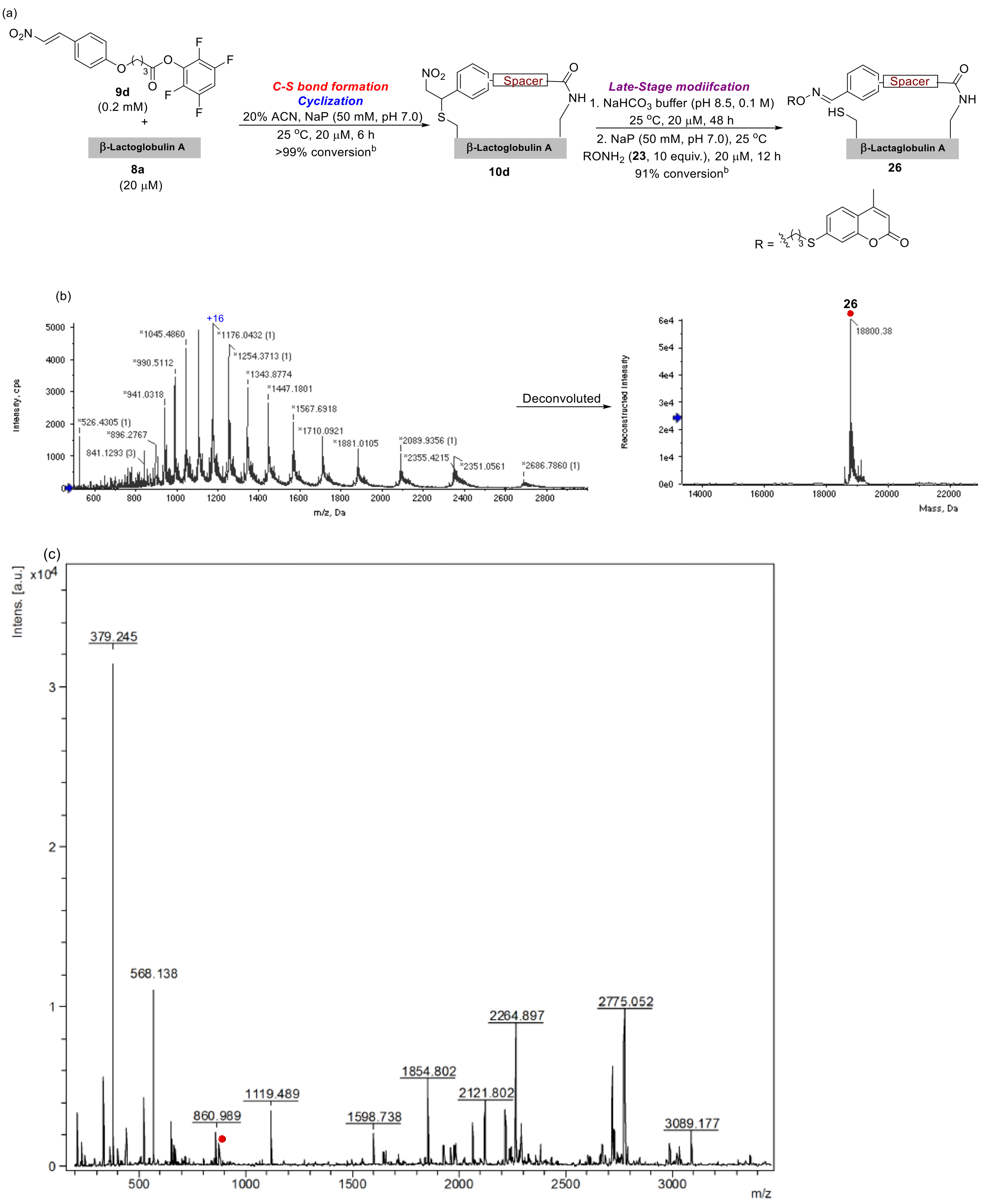

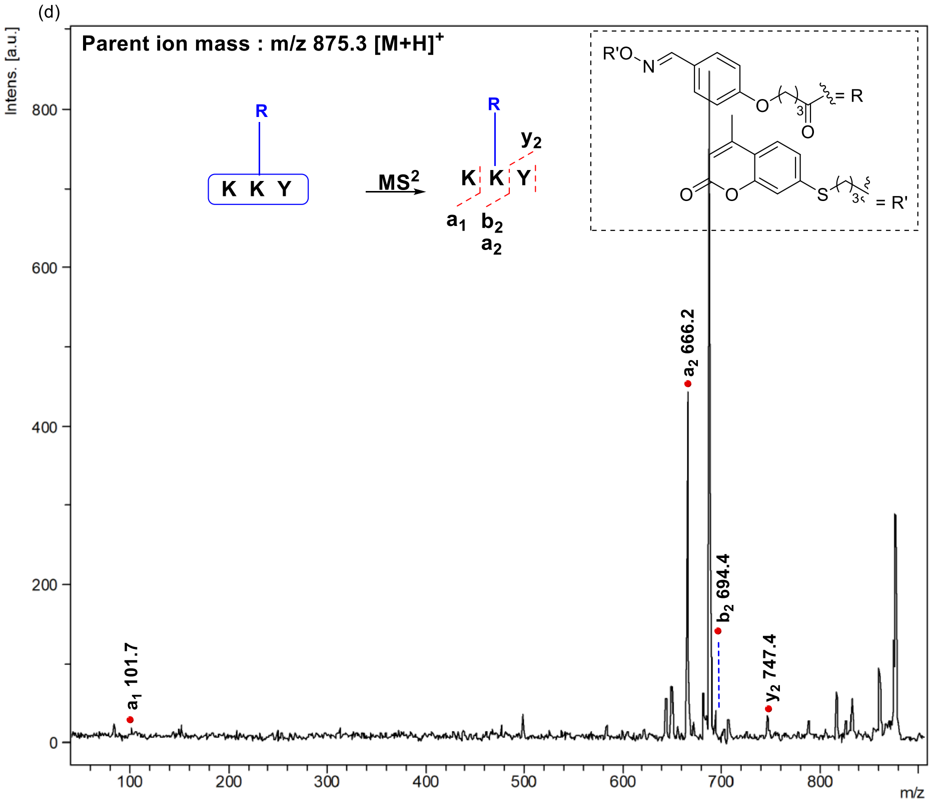

Supplementary Fig. 73. (a) Site-selective labeling of β-lactoglobulin A **8a** (1 equiv.) enabled by LDM_{CK} reagent **9d** (10 equiv.) followed by oxime formation with reagent **23**. (b) ESI-MS spectrum for mono-labeled β-lactoglobulin A (**26**, 91% conversion**)**. (c) Peptide mapping of **26** after the digestion with α-chymotrypsin. (c) MS-MS spectrum of labeled KKY (K100-K102, *m/z* 875.3 $[M+H]^+$). The site of modification in mono-labeled β-lactoglobulin A **26** is K101.

6.10 Dual probe installation

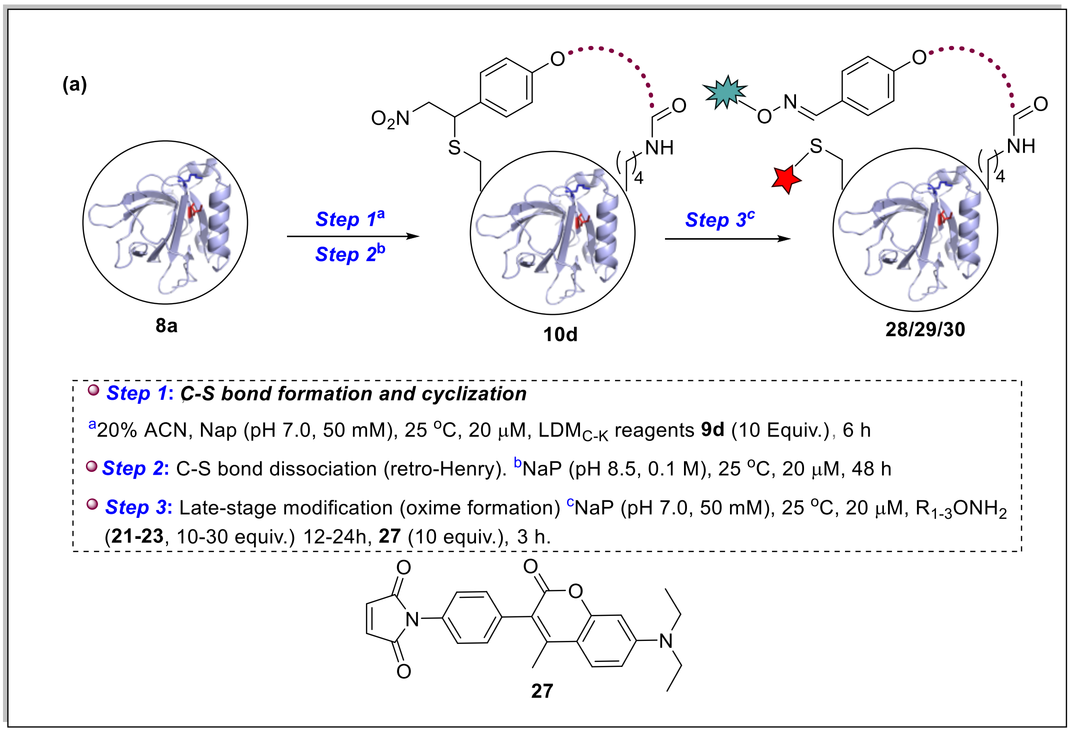

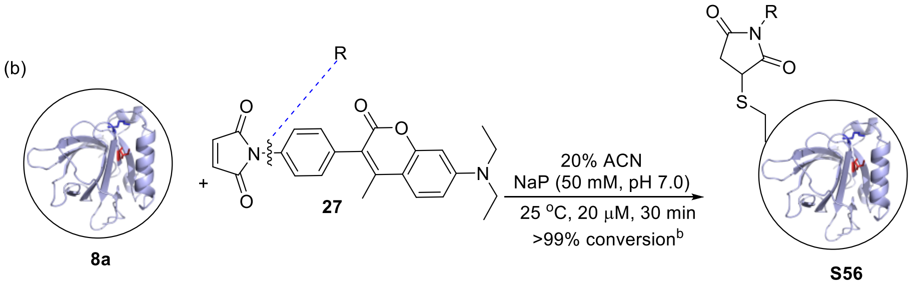

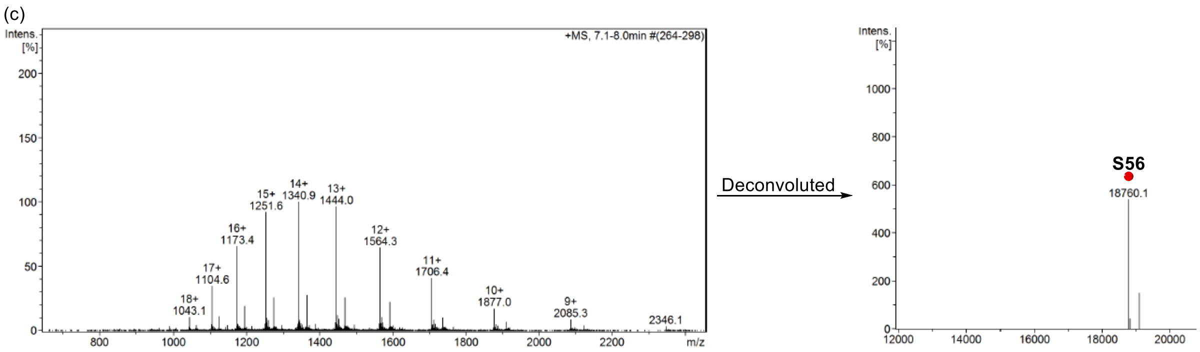

Supplementary Fig. 74. (a) Dual probe installation on β-lactoglobulin A **8a** enabled by LDM$_{CK}$ reagent **9d** (10 equiv.) followed by oxime formation with various hydroxyl amine derivative (**21-23,** 10-30 equiv.) and Michael addition by CPM dye (**27**, 10equiv.). (b) Reaction of CPM dye **27** with β-lactoglobulin A **8a**. (c) ESI-MS spectrum for mono-labeled β-lactoglobulin A (**S56**, >99% conversion**)**.

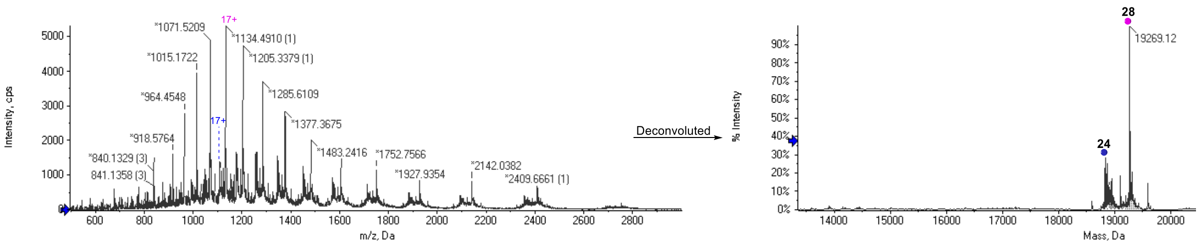

Supplementary Fig. 75. ESI-MS spectrum for mono-labeled β-lactoglobulin A (**28**, 78% conversion**)** after dual probe installation with hydroxyl amine derivative **21**, and CPM dye **27**.

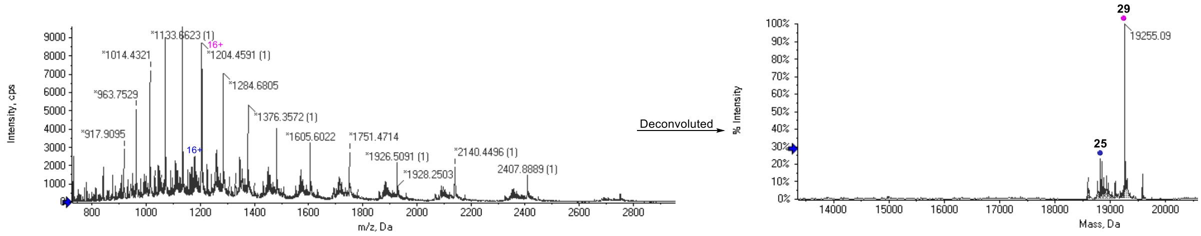

Supplementary Fig. 76. ESI-MS spectrum for mono-labeled β-lactoglobulin A (**29**, 73% conversion**)** after dual probe installation with hydroxyl amine derivative **22**, and CPM dye **27**.

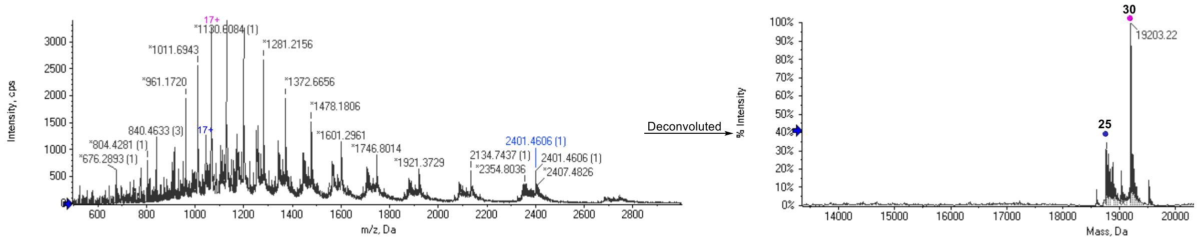

Supplementary Fig. 77. ESI-MS spectrum for mono-labeled β-lactoglobulin A (**30**, 71% conversion**)** after dual probe installation with hydroxyl amine derivative **23**, and CPM dye **27**.

6.11 Single-site modification of serum albumins (BSA and HSA)

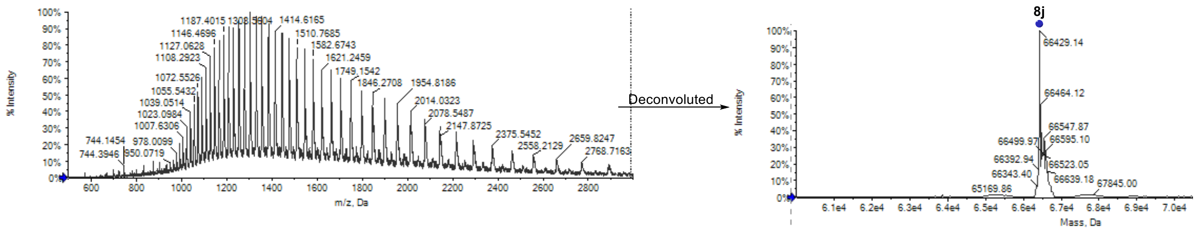

Supplementary Fig. 78. ESI-MS spectrum of native BSA **8j**.

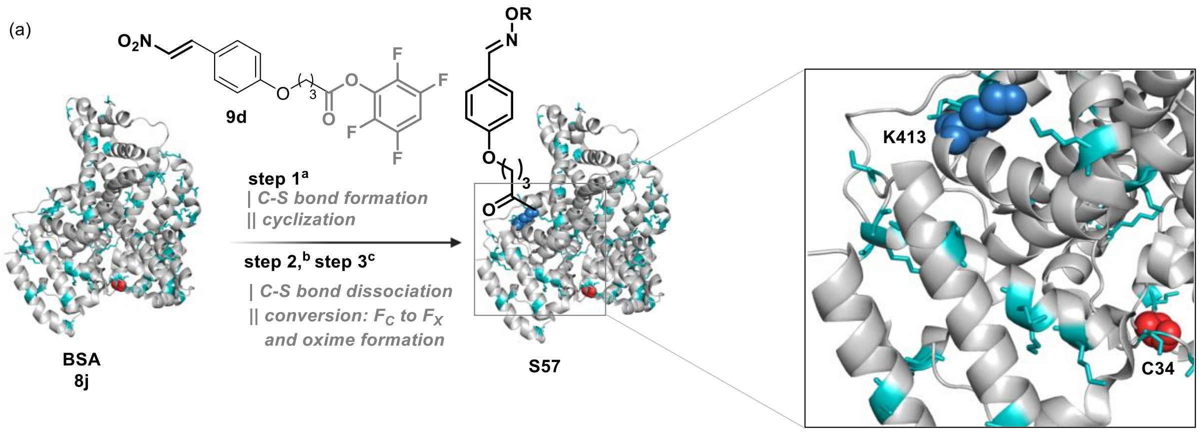

- **Step 1: C-S bond formation and cyclization**
  [a]20% ACN, Nap (pH 7.0, 50 mM), 25 °C, 20 μM, LDM_{C-K} reagents **9d** (10 Equiv.), 6 h
- **Step 2:** C-S bond dissociation (retro-Henry). [b]NaP (pH 8.5, 0.1 M), 25 °C, 20 μM, 48 h
- **Step 3:** Late-stage modification (oxime formation) [c]NaP (pH 7.0, 50 mM), 25 °C, 20 μM, $RONH_2$ (**19**, 200 equiv.) 3 h.

O O

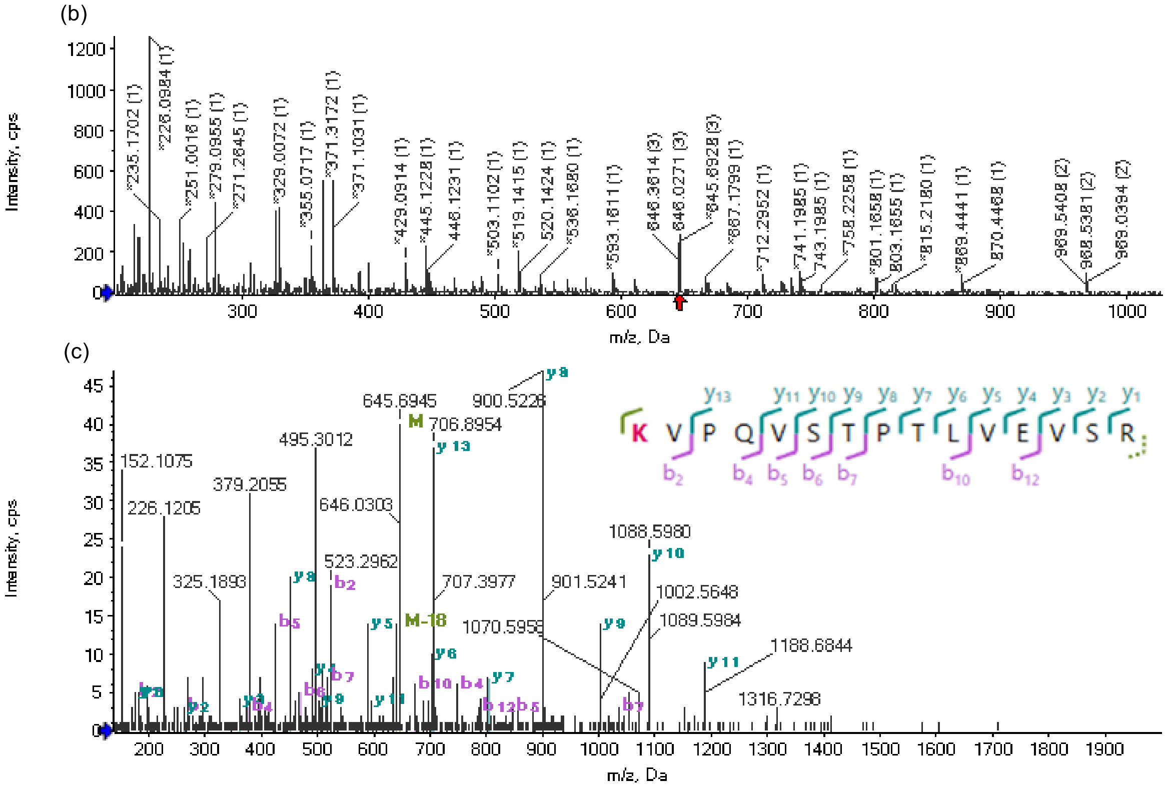

Supplementary Fig. 79. (a) Site-selective labeling of BSA **8j** (1 equiv.) enabled by LDM_{CK} reagent **9d** (10 equiv.). (b) Peptide mapping of **S57** after the digestion with trypsin. (c) MS-MS spectrum of labeled KVPQVSTPTLVEVSR (K413-R427, *m/z* 645.6 $[M+3H]^{3+}$). The site of modification in mono-labeled BSA **S57** is K413.

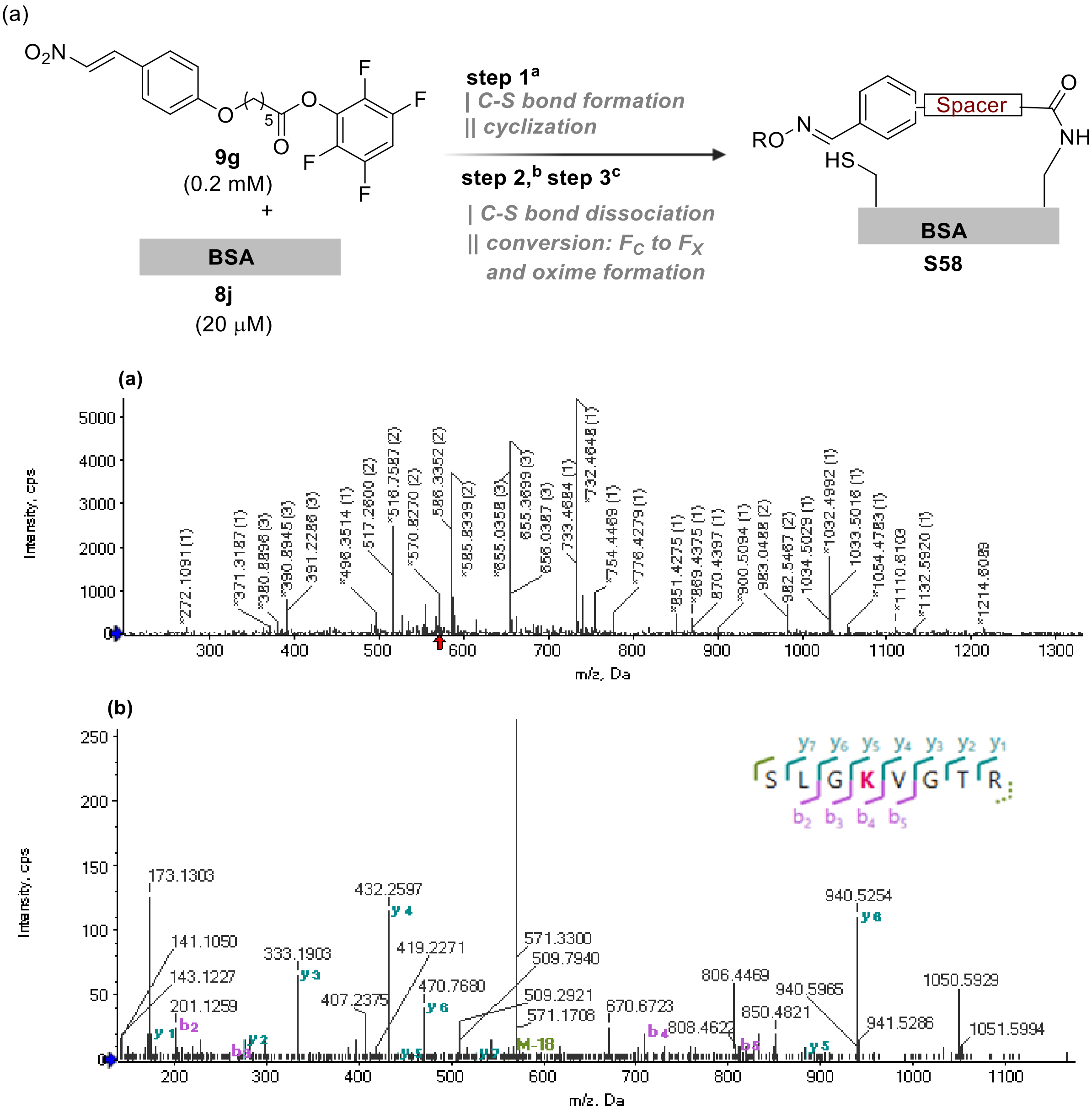

Supplementary Fig. 80. (a) Site-selective labeling of BSA **8j** (1 equiv.) enabled by LDM$_{CK}$ reagent **9g** (10 equiv.). (b) Peptide mapping of **S58** after the digestion with trypsin. (c) MS-MS spectrum of labeled SLGKVGTR (S428-R435, *m/z* 570.8 $[M+2H]^{2+}$). The site of modification in mono-labeled BSA **S58** is K431.

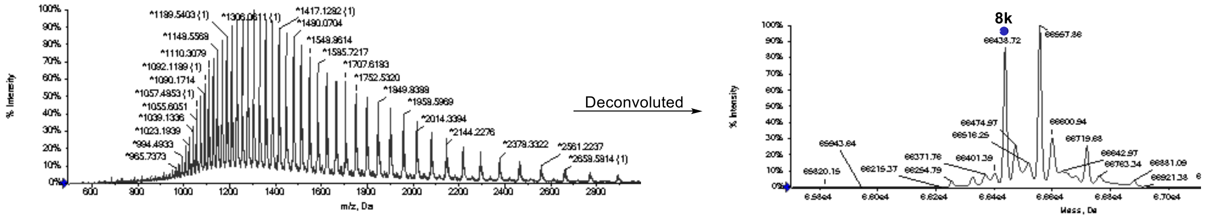

Supplementary Fig. 81. ESI-MS spectrum of native HSA **8k**.

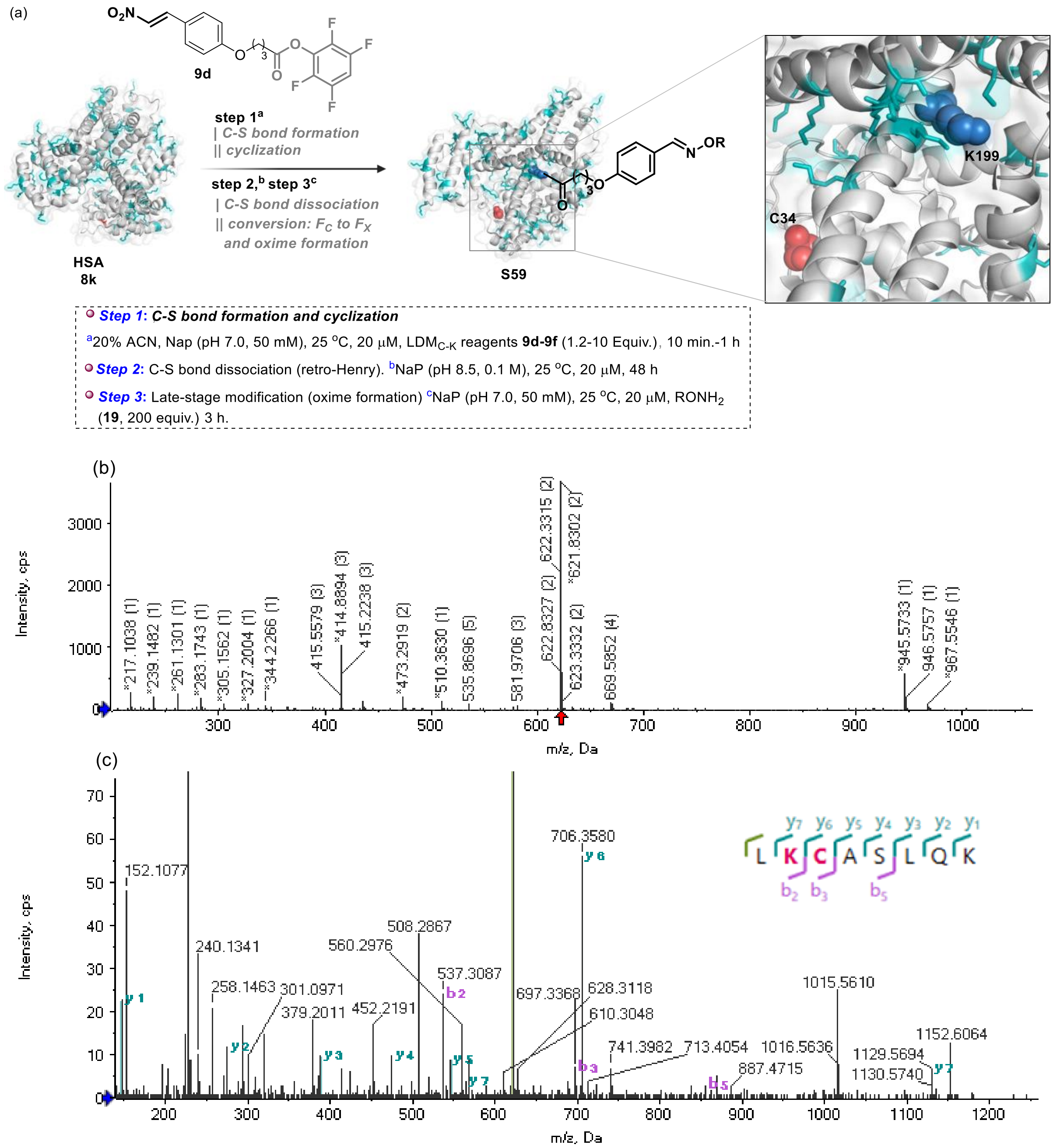

Supplementary Fig. 82. (a) Site-selective labeling of HSA **8k** (1 equiv.) enabled by LDM_{CK} reagent **9d** (10 equiv.). (b) Peptide mapping of **S59** after the digestion with trypsin. (c) MS-MS spectrum of labeled LKCASLQK (L198-K205, *m/z* 621.8 $[M+2H]^{2+}$). The site of modification in mono-labeled HSA **S59** is K199.

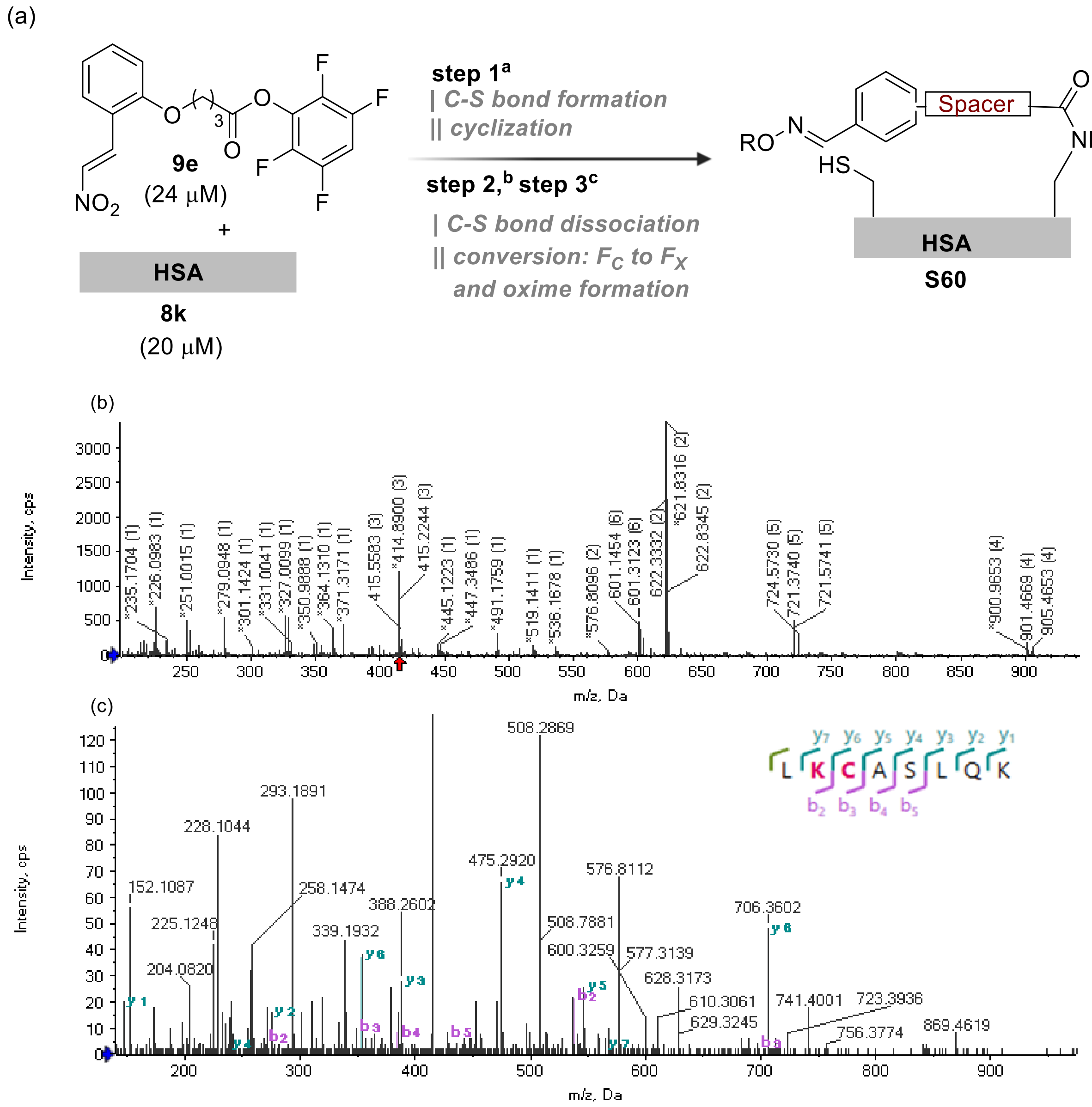

Supplementary Fig. 83. (a) Site-selective labeling of HSA **8k** (1 equiv.) enabled by LDM_{CK} reagent **9e** (1.2 equiv.). (b) Peptide mapping of **S60** after the digestion with trypsin. (c) MS-MS spectrum of labeled LKCASLQK (L198-K205, *m/z* 414.8 $[M+3H]^{3+}$). The site of modification in mono-labeled HSA **S60** is K199.

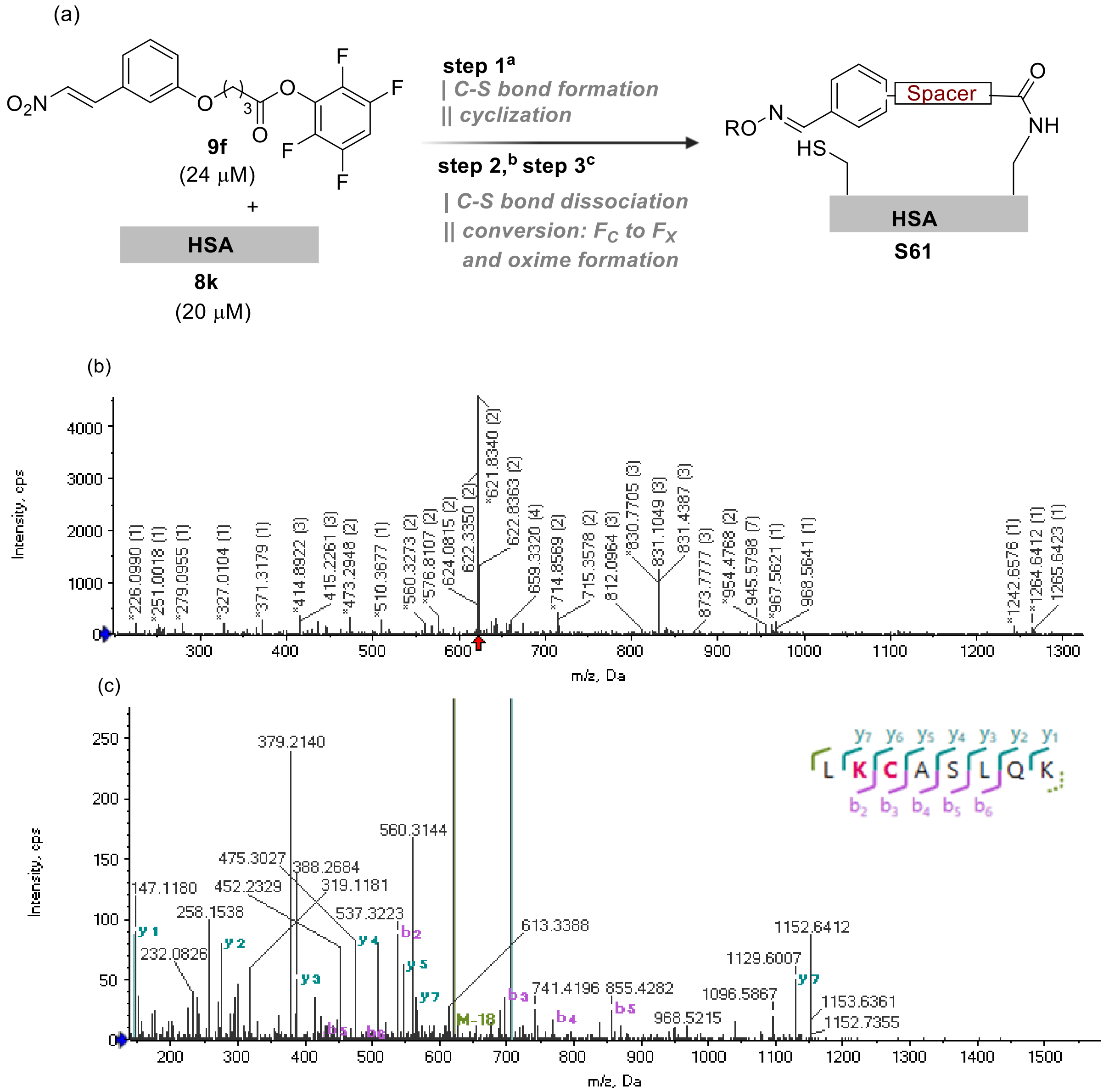

Supplementary Fig. 84. (a) Site-selective labeling of HSA **8k** (1 equiv.) enabled by LDM_{CK} reagent **9f** (1.2 equiv.). (b) Peptide mapping of **S61** after the digestion with trypsin. (c) MS-MS spectrum of labeled LKCASLQK (L198-K205, *m/z* 621.8 $[M+2H]^{2+}$). The site of modification in mono-labeled HSA **S61** is K199.

6.12 Modification and enrichment of HSA from mixture of proteins

(a)

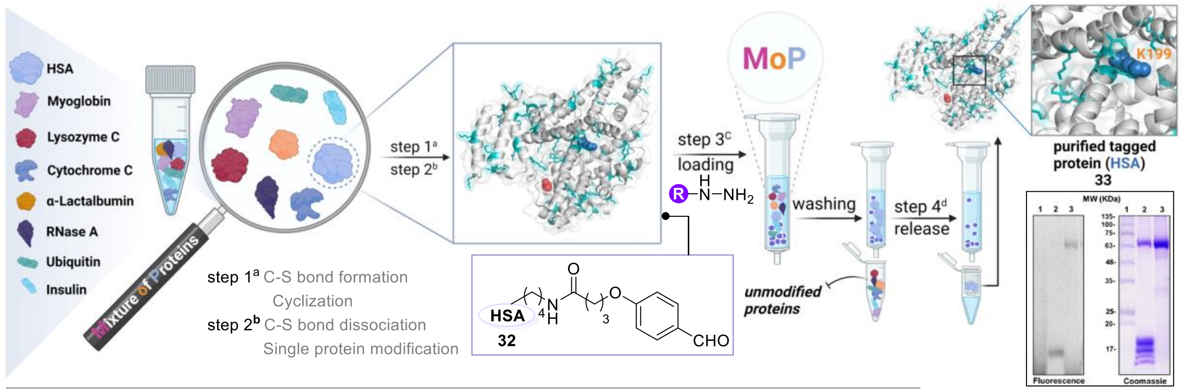

[a]NaP buffer (pH 7.0, 50 mM), 20% ACN, LDM_{C-K} reagent (**9d**); [b]$NaHCO_3$ buffer (pH 8.5, 0.1 M), 25 °C, 48 h.
[c]NaP buffer (pH 7.0, 50 mM), 25 °C, 24 h; [d]NaP buffer (pH 6.5, 50 mM), 25 °C, $RONH_2$ (**23**, 100 equiv.), 10% ACN, 24 h.

(b)

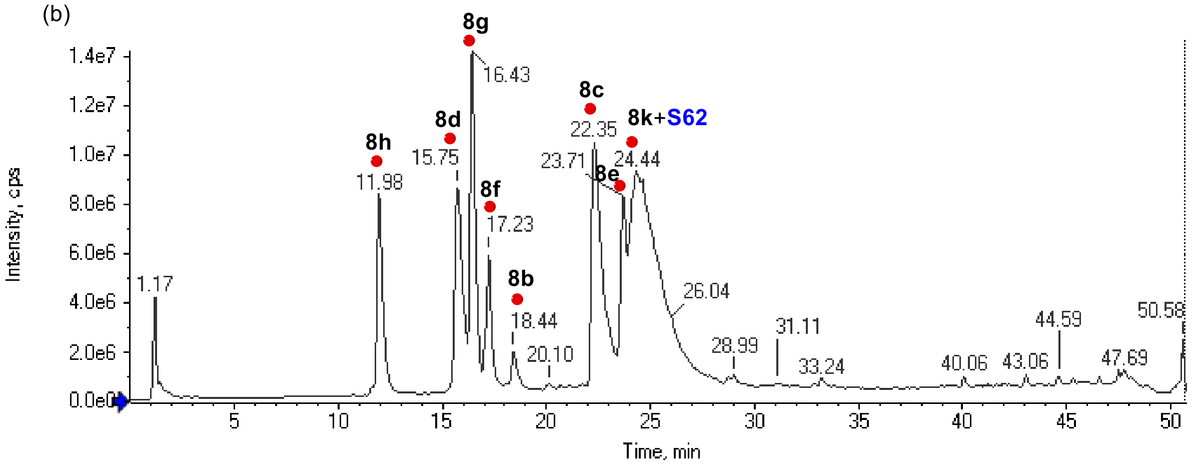

(c)

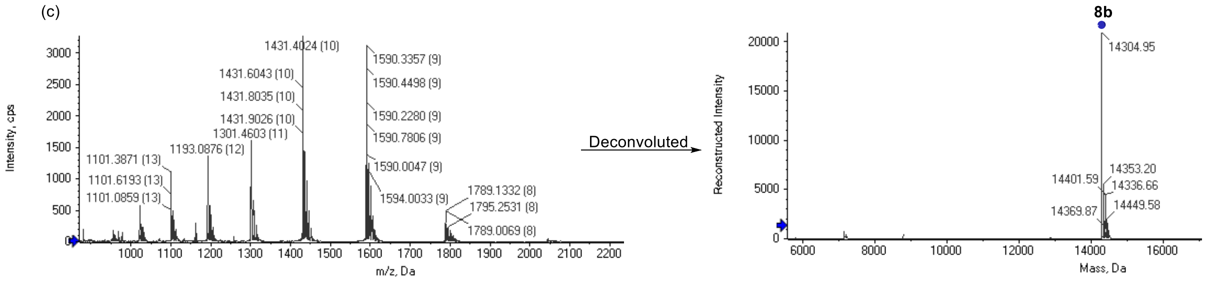

(d)

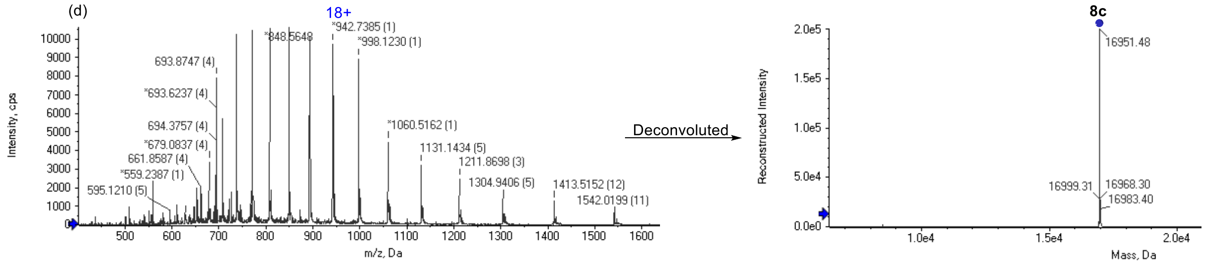

(e)

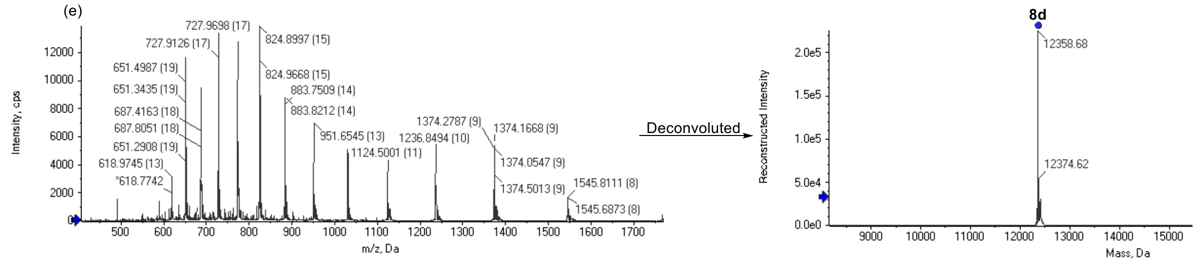
727.9698 (17)
727.9126 (17)
824.8997 (15)
651.4987 (19)
651.3435 (19)
824.9668 (15)
883.7509 (14)
883.8212 (14)
687.4163 (18)
687.8051 (18)
651.2908 (19)
951.6545 (13)
1124.5001 (11)
1236.8494 (10)
1374.2787 (9)
1374.1668 (9)
1374.0547 (9)
1374.5013 (9)
1545.8111 (8)
1545.6873 (8)
618.9745 (13)
*618.7742
Intensity, cps
m/z, Da
Deconvoluted
8d
12358.68
12374.62
Reconstructed Intensity
Mass, Da

(f)

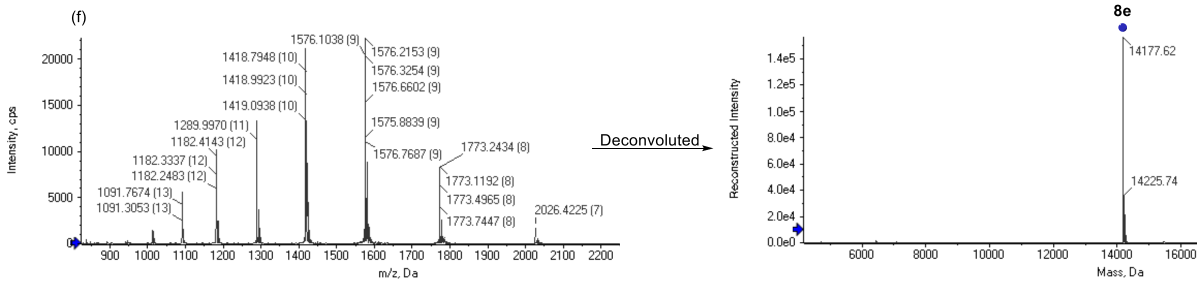
1576.1038 (9)
1576.2153 (9)
1576.3254 (9)
1576.6602 (9)
1575.8839 (9)
1576.7687 (9)
1418.7948 (10)
1418.9923 (10)
1419.0938 (10)
1289.9970 (11)
1182.4143 (12)
1182.3337 (12)
1182.2483 (12)
1091.7674 (13)
1091.3053 (13)
1773.2434 (8)
1773.1192 (8)
1773.4965 (8)
1773.7447 (8)
2026.4225 (7)
Intensity, cps
m/z, Da
Deconvoluted
8e
14177.62
14225.74
Reconstructed Intensity
Mass, Da

(g)

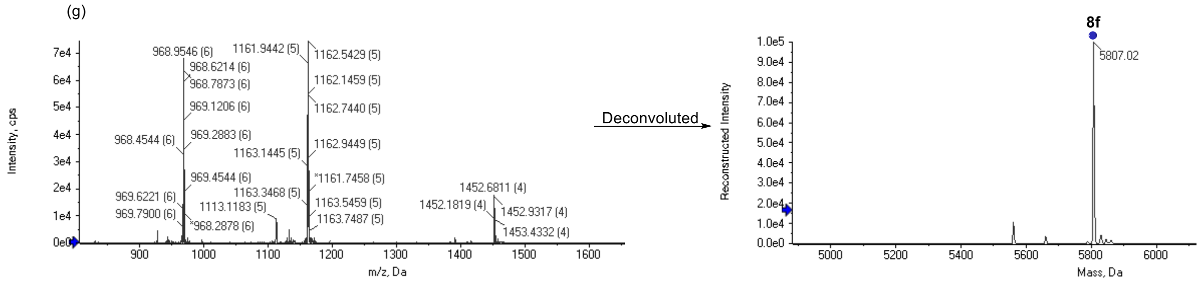
968.9546 (6)
968.6214 (6)
968.7873 (6)
969.1206 (6)
969.2883 (6)
968.4544 (6)
969.4544 (6)
969.6221 (6)
969.7900 (6)
*968.2878 (6)
1161.9442 (5)
1162.5429 (5)
1162.1459 (5)
1162.7440 (5)
1162.9449 (5)
1163.1445 (5)
*1161.7458 (5)
1163.3468 (5)
1163.5459 (5)
1163.7487 (5)
1113.1183 (5)
1452.6811 (4)
1452.1819 (4)
1452.9317 (4)
1453.4332 (4)
Intensity, cps
m/z, Da
Deconvoluted
8f
5807.02
Reconstructed Intensity
Mass, Da

(h)

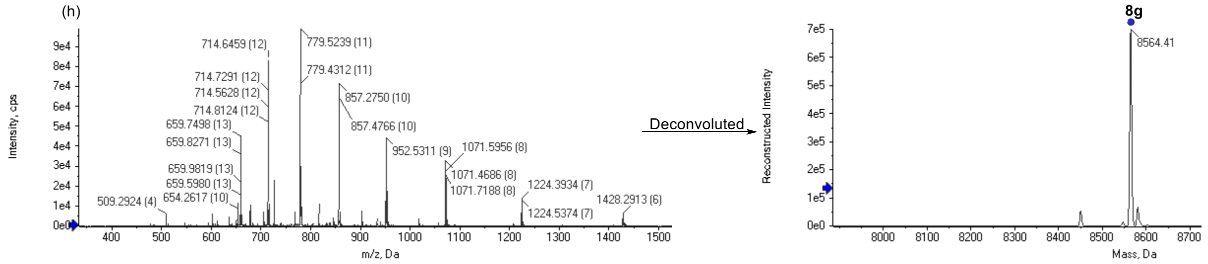
714.6459 (12)
779.5239 (11)
779.4312 (11)
714.7291 (12)
714.5628 (12)
714.8124 (12)
857.2750 (10)
857.4766 (10)
659.7498 (13)
659.8271 (13)
659.9819 (13)
659.5980 (13)
654.2617 (10)
509.2924 (4)
952.5311 (9)
1071.5956 (8)
1071.4686 (8)
1071.7188 (8)
1224.3934 (7)
1224.5374 (7)
1428.2913 (6)
Intensity, cps
m/z, Da
Deconvoluted
8g
8564.41
Reconstructed Intensity
Mass, Da

(i)

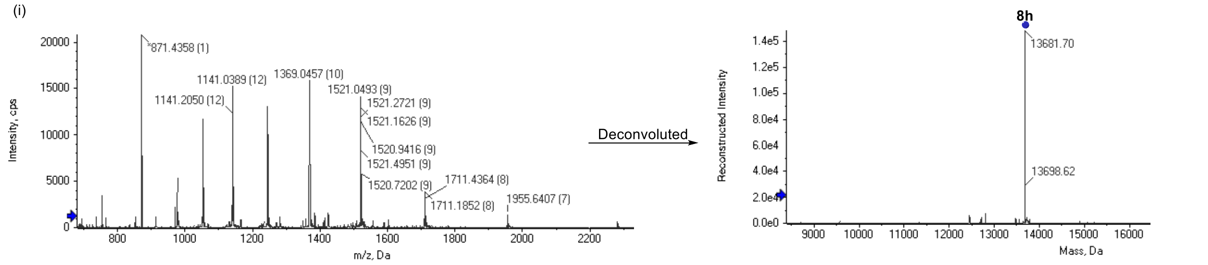
*871.4358 (1)
1141.0389 (12)
1141.2050 (12)
1369.0457 (10)
1521.0493 (9)
1521.2721 (9)
1521.1626 (9)
1520.9416 (9)
1521.4951 (9)
1520.7202 (9)
1711.4364 (8)
1711.1852 (8)
1955.6407 (7)
Intensity, cps
m/z, Da
Deconvoluted
8h
13681.70
13698.62
Reconstructed Intensity
Mass, Da

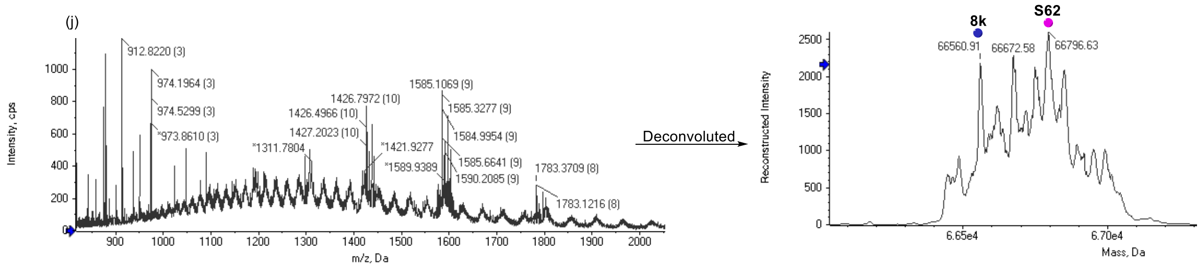

Supplementary Fig. 85. (a) Modification and enrichment of HSA **8k** in mixture of proteins enabled by LDM$_{CK}$ reagent **9d** (10 equiv.). (b) TIC showing mixture of proteins (**8b-8h**) also modified HSA (**S62**) eluted at different time interval after the treatment with LDM$_{CK}$ reagent **9d**. (c-i) ESI-MS spectra of various f proteins (**8b-8h**) including modified HSA (**S62**) suggesting the selective modification of HSA (**S62**) after the reaction with LDM$_{CK}$ reagent **9d**.

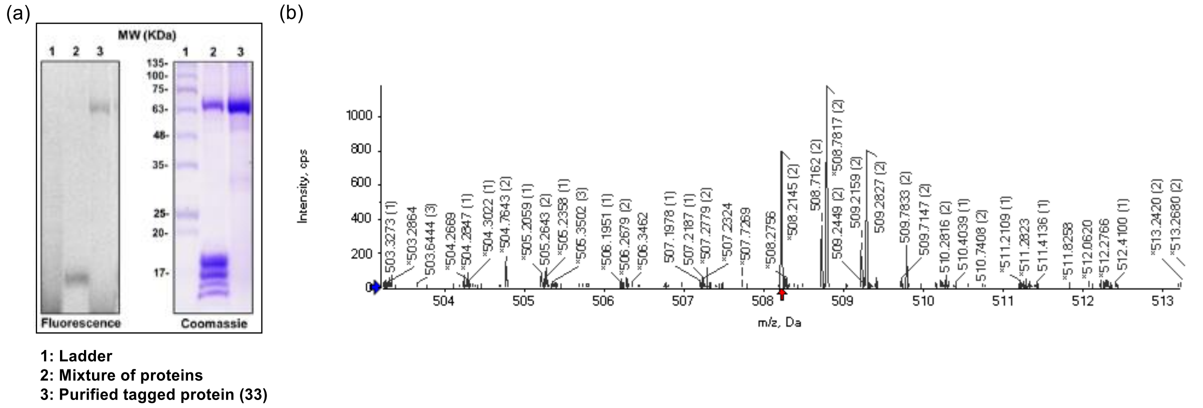

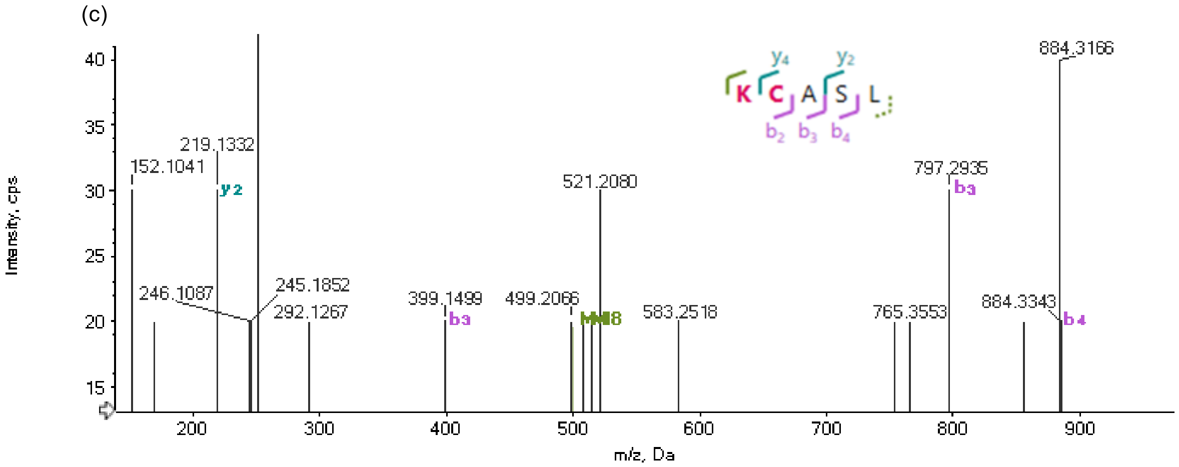

Supplementary Fig. 86. (a) Gradient 10-20% SDS-PAGE showing fluorescence imaging and Coomassie staining. MW - Molecular Weight, SDS-PAGE: lane 1 - ladder, lane 2 – mixture of proteins containing HSA **8k**, lane 3 - coumarin-tagged purified HSA **33**. (b) Peptide mapping of coumarin-tagged purified HSA **33** after the digestion with α-chymotrypsin. (c) MS-MS spectrum of labeled KCASL (K199-L203, *m/z* 508.2 $[M+2H]^{2+}$). The site of modification in coumarin-tagged purified HSA **33** is K199.

6.13 Modification and enrichment of HSA from cell lysate

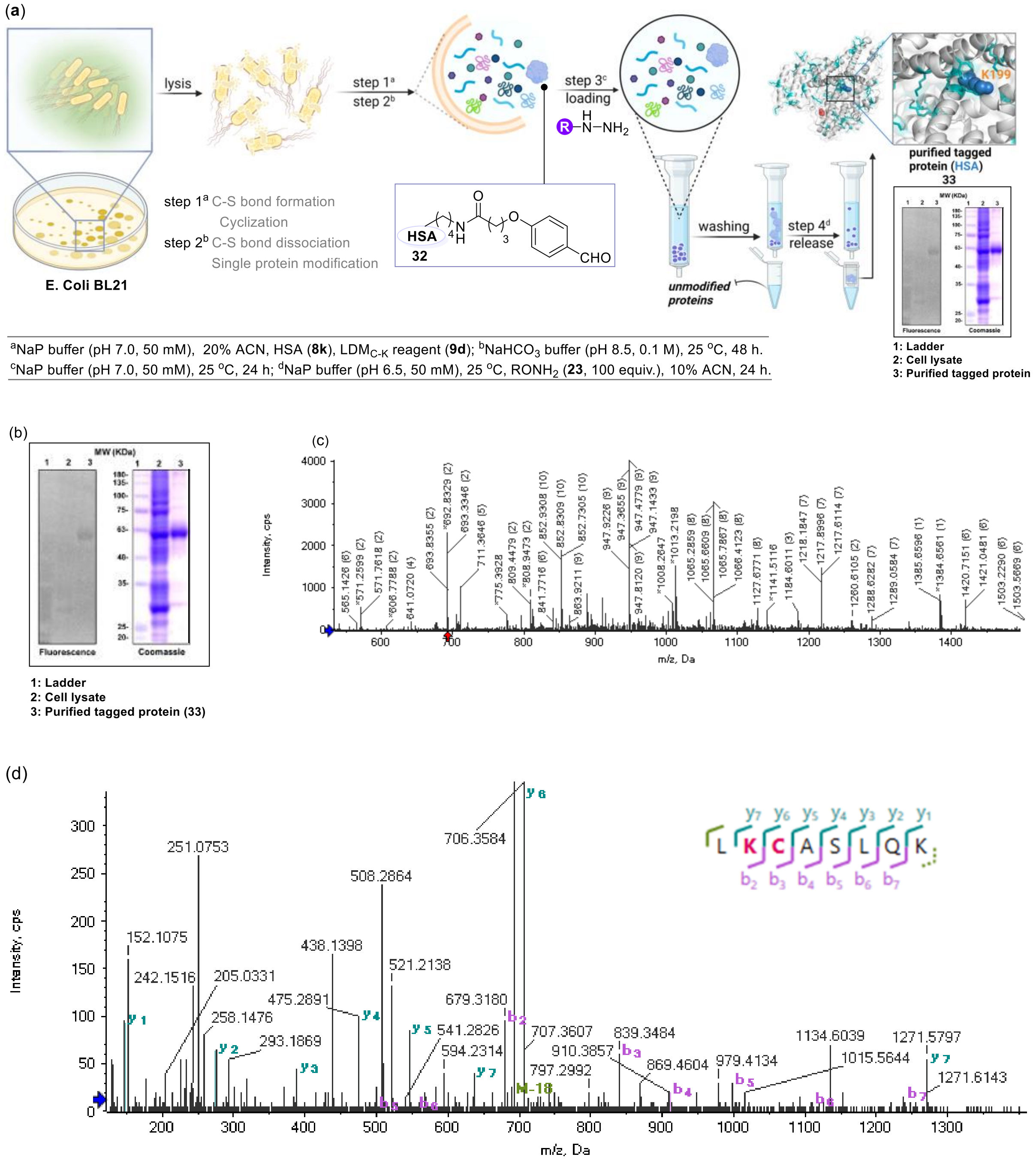

Supplementary Fig. 87. (a) Modification and enrichment of HSA **8k** in cell lysate derived from BL21 cells, E. coli enabled by LDM$_{CK}$ reagent **9d** (10 equiv.). (b) 10% SDS-PAGE showing fluorescence imaging and Coomassie staining. MW - Molecular Weight, SDS-PAGE: lane 1 - ladder, lane 2 - cell lysate containing HSA **8k**, lane 3 - coumarin-tagged purified HSA **33**. (c) Peptide mapping of coumarin-tagged purified HSA **33** after the digestion with trypsin. (d) MS-MS spectrum of labeled LKCASLQK (L198-K205, *m/z* 692.8 $[M+2H]^{2+}$). The site of modification in coumarin-tagged purified HSA **33** is K199.

7. Trastuzumab modification

(**a**)

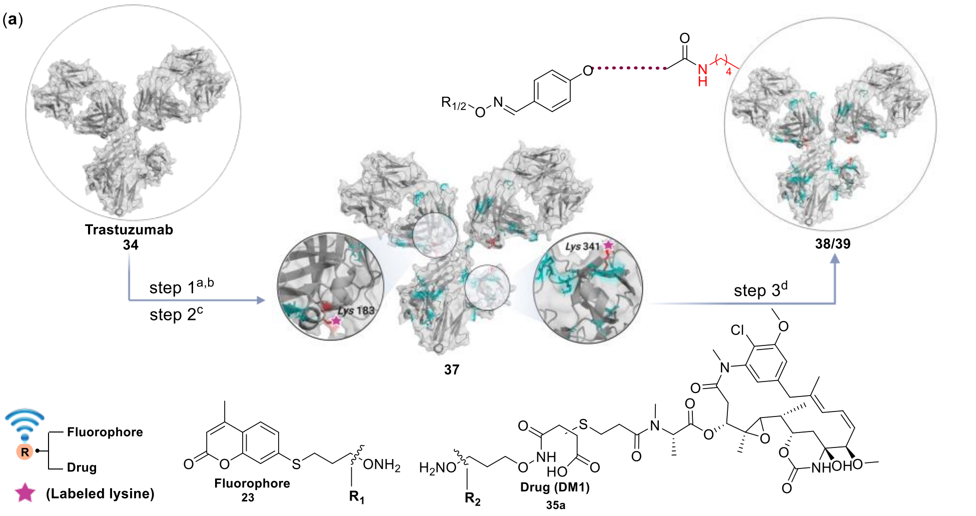

step 1: C-S bond formation and cyclization. [a]TCEP (1 equiv.), 1 h, NaP (pH 7.0, 50 mM), 25 °C, 20 μM; [b]20% ACN, $LDM_{C\text{-}K}$ reagent **9d** (15 equiv.), 8 h.
step 2: C-S and C-C bond dissociation (retro-Henry). [c]$NaHCO_3$ buffer (pH 8.5, 0.1 M), 25 °C, 20 μM, 24 h.
step 3: Late-stage modification (oxime formation). [d]NaP (pH 7.0, 50 mM), 25 °C, 20 μM, $R_{1\text{-}2}ONH_2$ (50-100 equiv.), 3 -12 h.

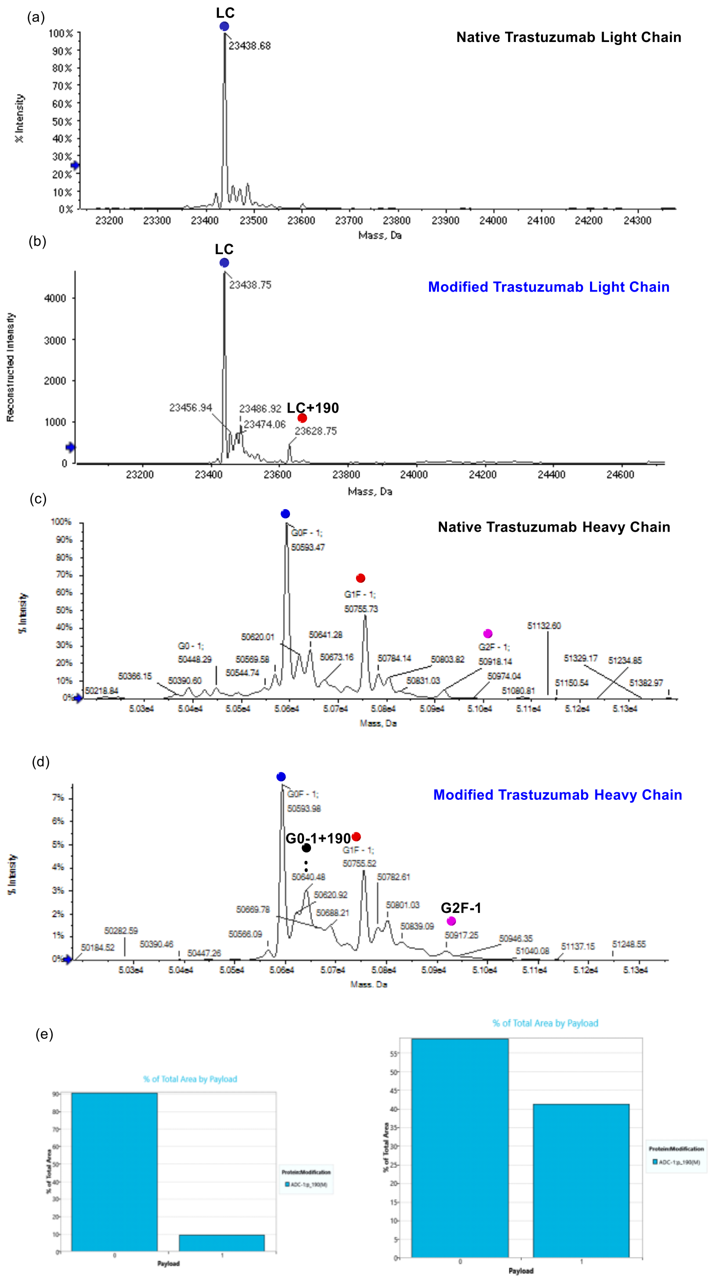

Supplementary Fig. 88. (a) Labeling of trastuzumab **34** (1 equiv.) enabled by TCEP (1 equiv.) followed by LDM$_{C\text{-}K}$ reagent **9d** (15 equiv.) followed by oxime formation with (**23/35a)**. (b) ESI-MS data for light chain in native trastuzumab. (d) ESI-MS data for light chain in modified trastuzumab. (c) ESI-MS data for heavy chain in native trastuzumab, showing all the post-translational modification. (d) ESI-MS data for heavy chain in modified trastuzumab, showing all the post-translational modification. (e) The bar graph suggesting the percentage modification of light chain (10%) and heavy chain (41%), which sum up the aldehyde antibody ratio (AAR) 1.0.

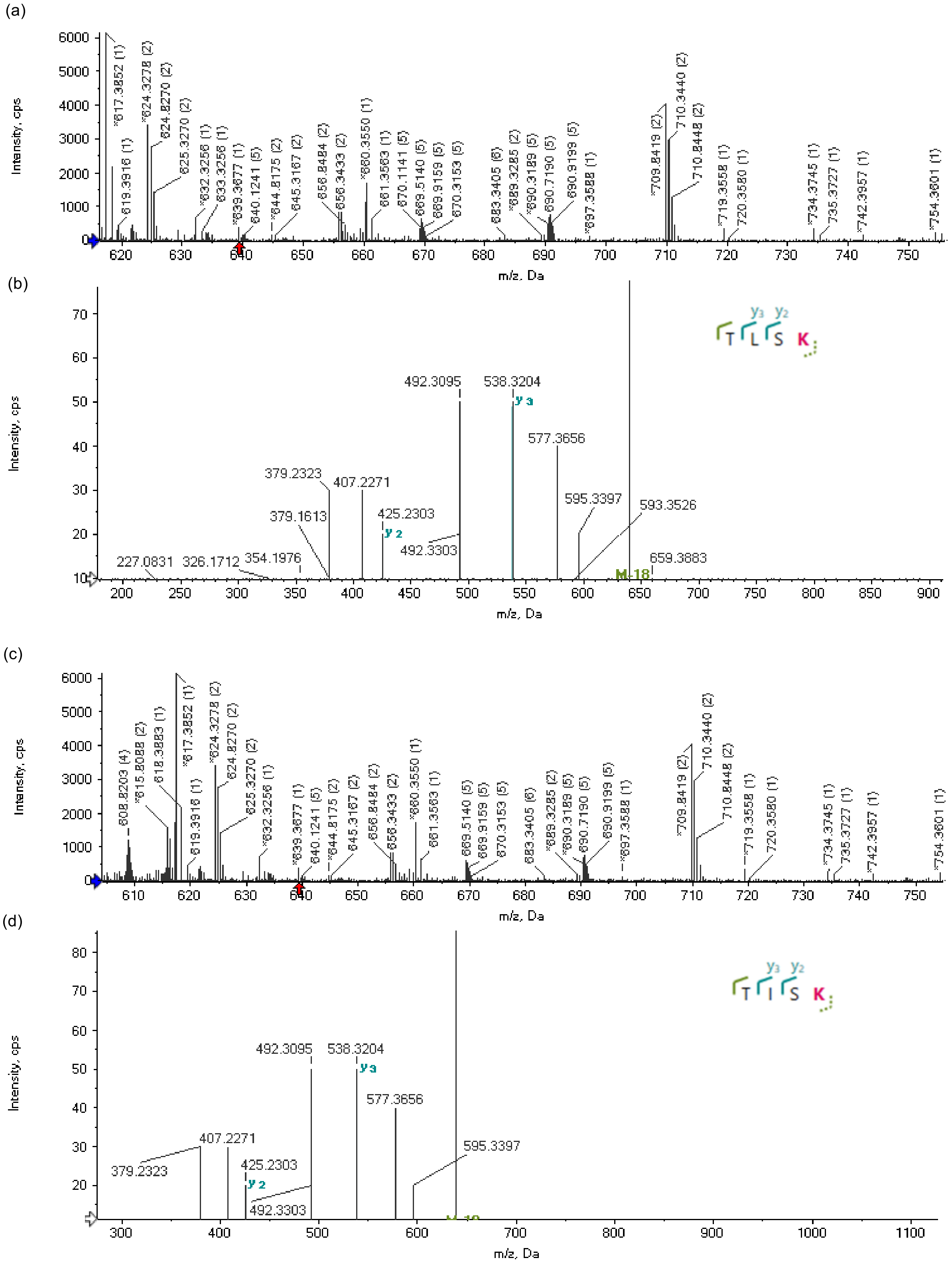

Supplementary Fig. 89. (a) Peptide mapping of light chain of aldehyde-antibody conjugate **37** after the digestion with α-chymotrypsin and trypsin both. (b) MS-MS spectrum of labeled TLSK (T180-K183, *m/z* 639.3 $[M+H]^+$). The site of modification in light chain of modified trastuzumab **37** is K183. (c) Peptide mapping of heavy chain of **37** after the digestion with α-chymotrypsin and trypsin both. (d) MS-MS spectrum of labeled TISK (T338-K341, *m/z* 639.3 $[M+H]^+$). The site of modification in heavy chain of modified trastuzumab **37** is K341.

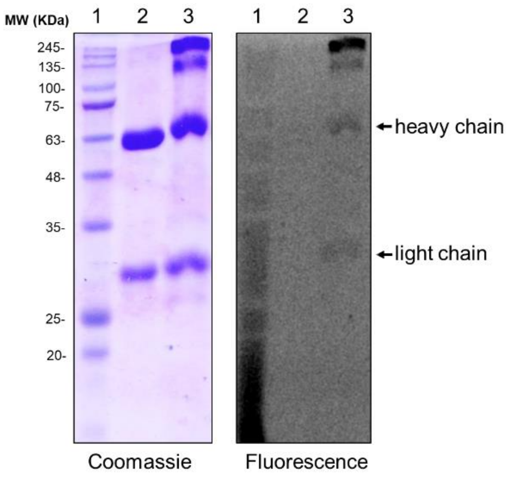

Supplementary Fig. 90. 12% SDS-PAGE of trastuzumab **34** and coumarin-tagged trastuzumab **38** after DTT (10 mM, 15 min, 37 ºC) reduction followed by Coomassie staining and fluorescence imaging. MW - Molecular Weight, SDS-PAGE: lane 1 - ladder, lane 2 - trastuzumab (**34**), lane 3 - coumarin-tagged trastuzumab (AFC, **38**). The band at ~25 kDa and ~50 kDa correspond to the reduced light chain and heavy chain, respectively. The presence of band ~25 kDa and ~50 kDa shown by fluorescence imaging confirms the labeling of light and heavy chain.

8. Supplementary Notes 4

8.1 Molecular dynamics simulations

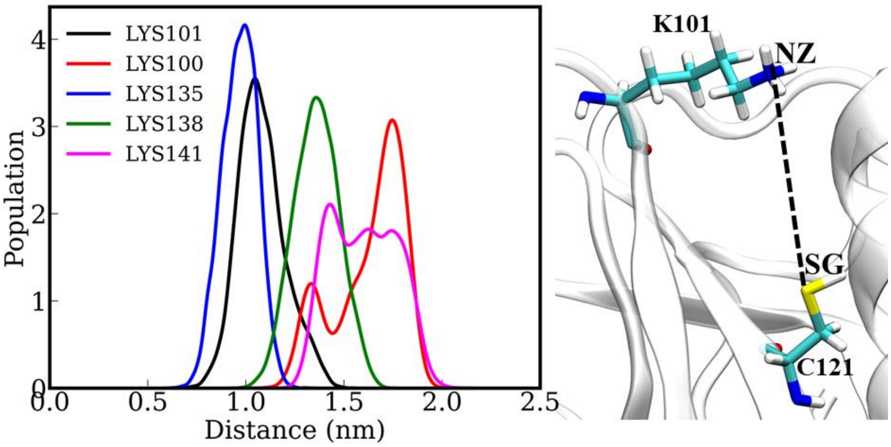

Supplementary Fig. 91. Probability distribution of the distance between the Cys121-SG atom and the NZ atom of nearby lysine residues in the protein without LDM_{C-K} reagent in water. As an example, the distance for Lys101 is shown in the inset.

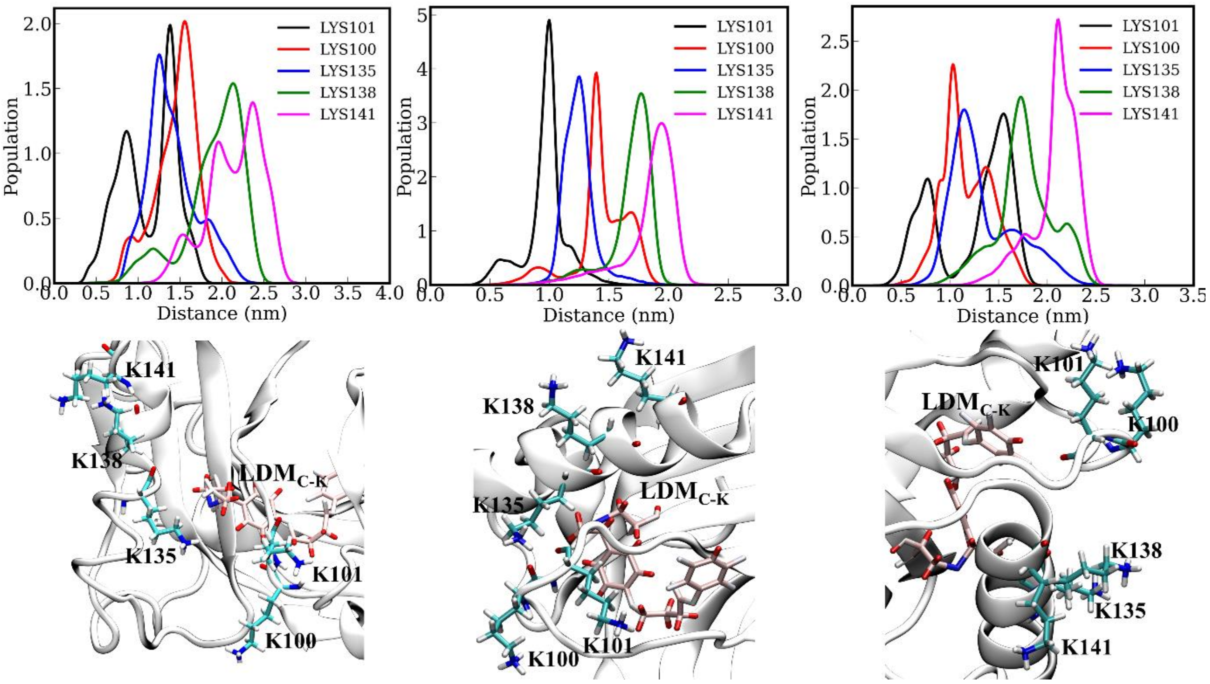

Supplementary Fig. 92. Probability distribution of the distance between the C11 atom of the LDM_{C-K} reagent and the NZ atom of nearby lysine residues in the protein in all the three sets of independent runs. The structural snapshots show the interaction of those lysine residues with the LDM_{C-K} reagent.

8.2 Stability of the LDM_{C-K} reagent 9d

Supplementary Table 8: Stability of LDM_{C-K} reagent 9d.

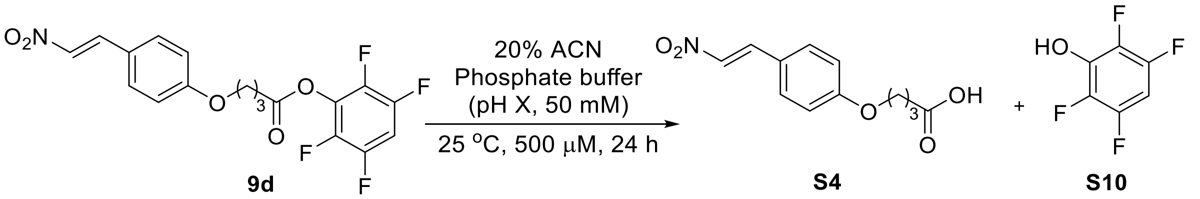

Entry	pH (X)	% Conversion[a] (S4) 0 h	6 h	12 h	24 h
1	6.0	0	0	0	0
2	7.0	0	0	0	0
3	7.4	0	0	0	0
4	8.0	0	0	0	0
5	9.0	0	13	20	46

[a] % Conversion based on LC-ESI-MS. For data, see Supplementary Fig. 93 below.

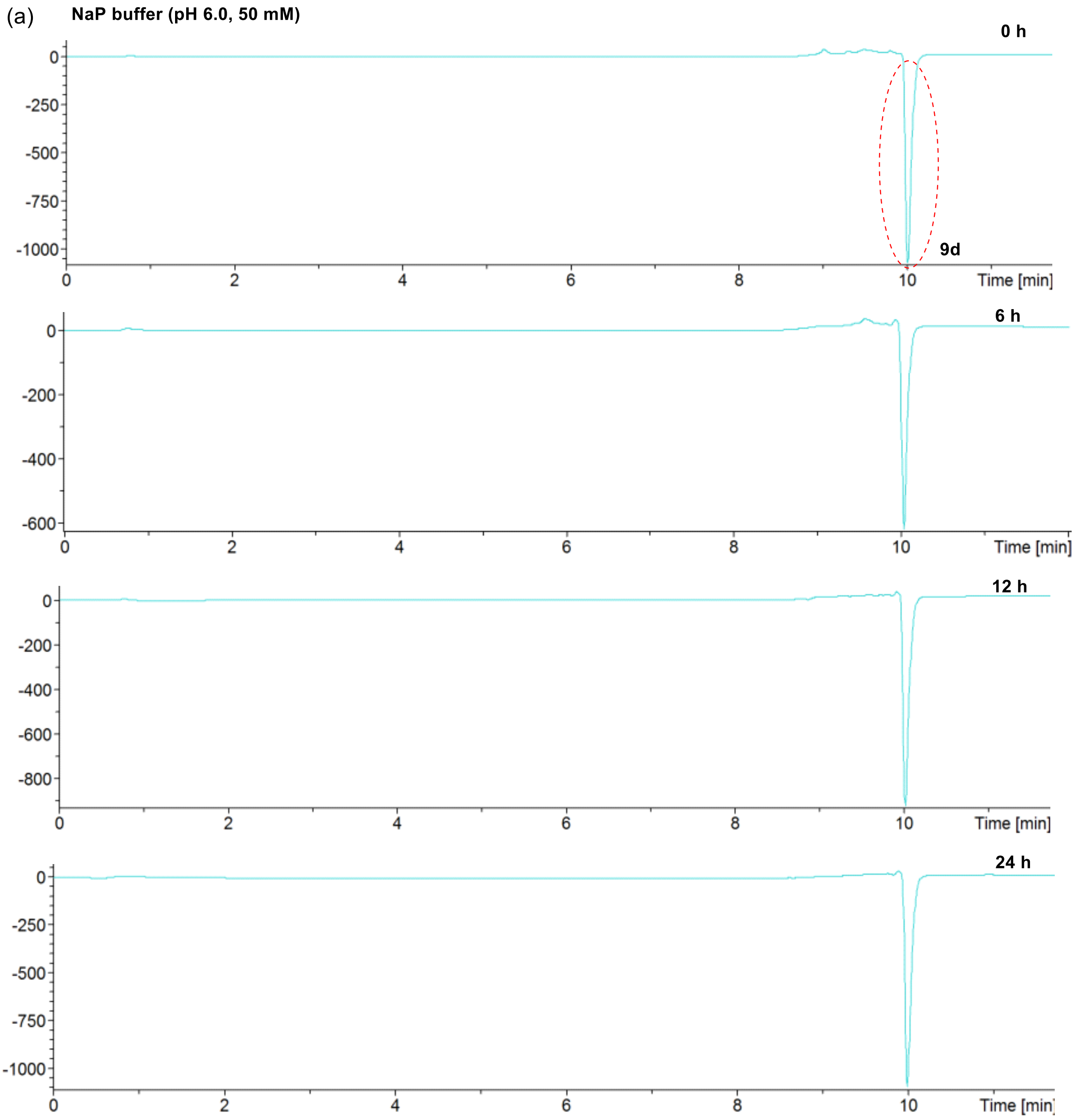

(a)
NaP buffer (pH 6.0, 50 mM)
0 h
9d
6 h
12 h
24 h
Time [min]

(b) **NaP buffer (pH 7.0, 50 mM)**

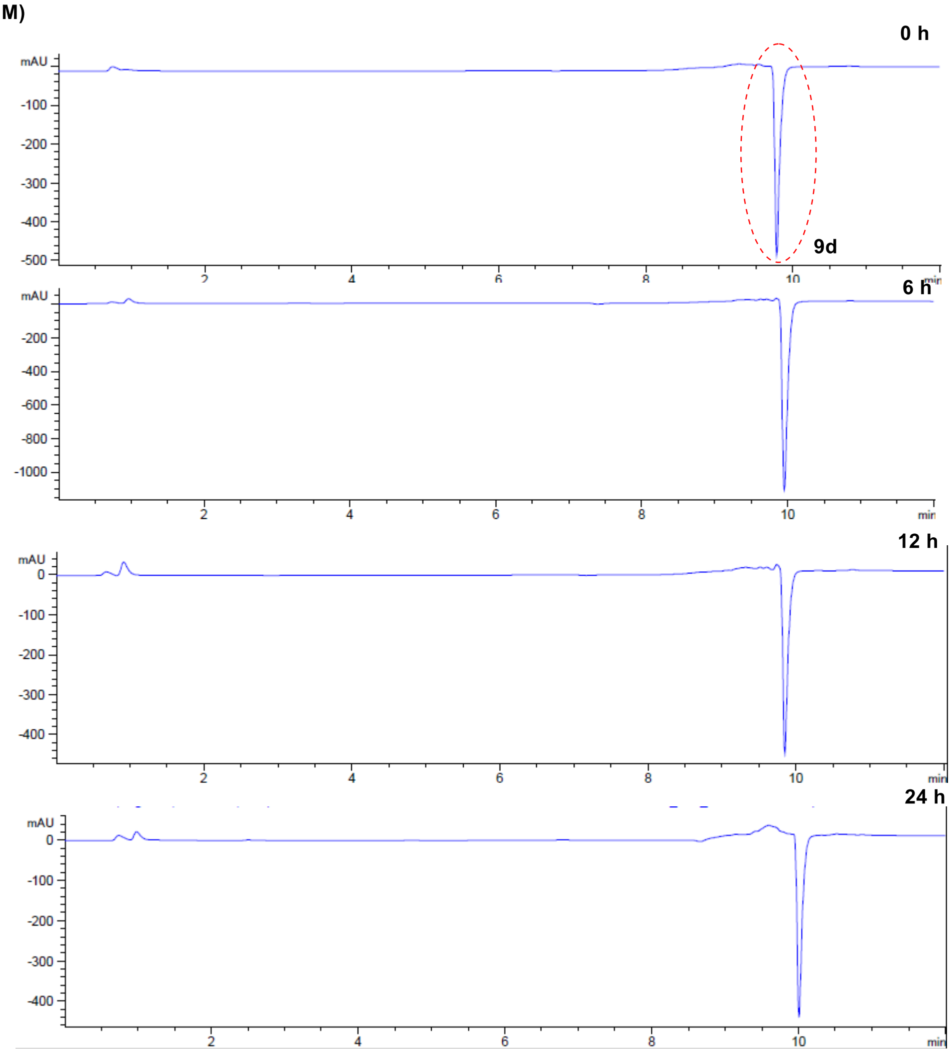

(c) **NaP buffer (pH 7.4, 50 mM)**

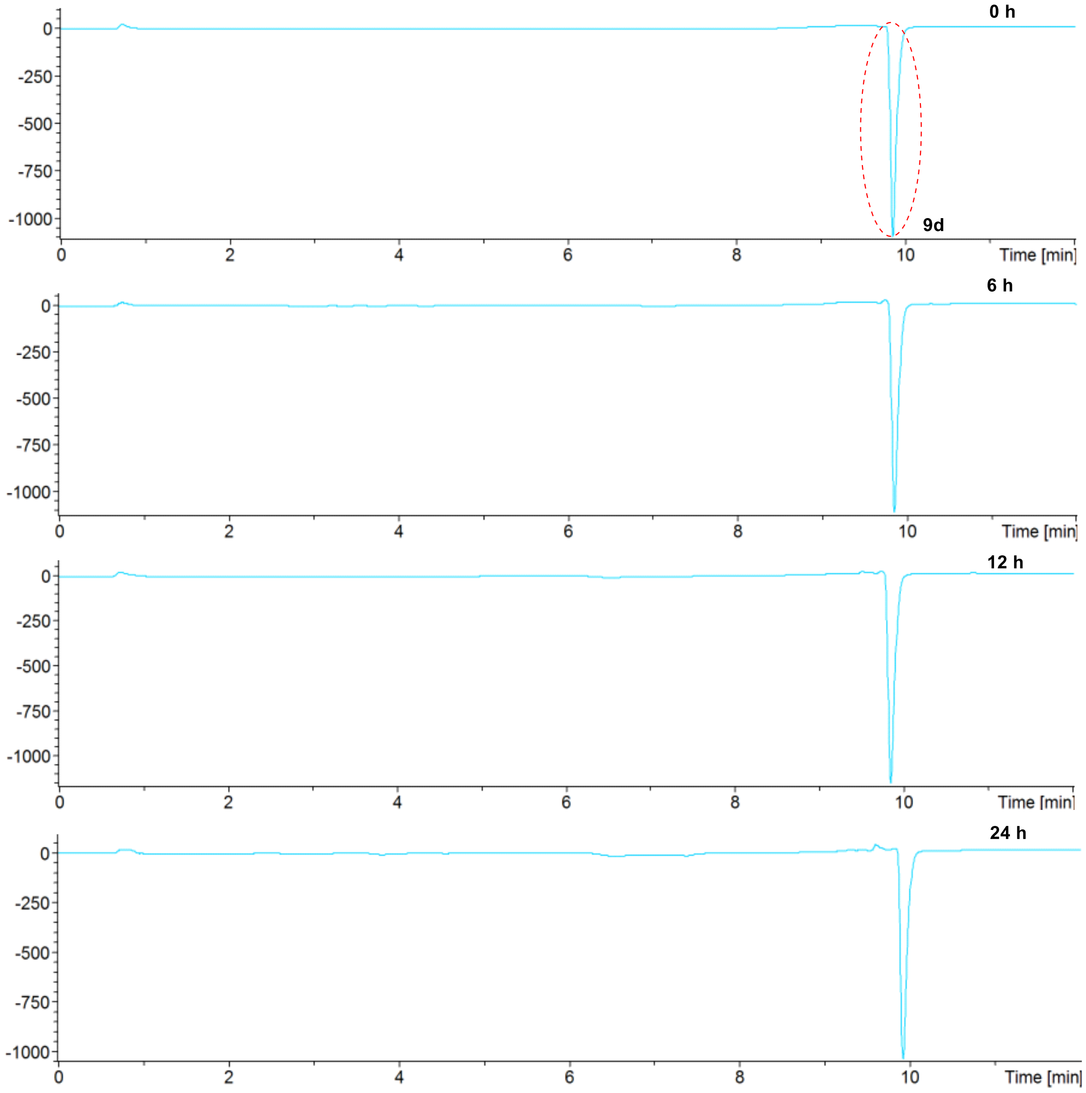

(d) **NaP buffer (pH 8.0, 50 mM)**

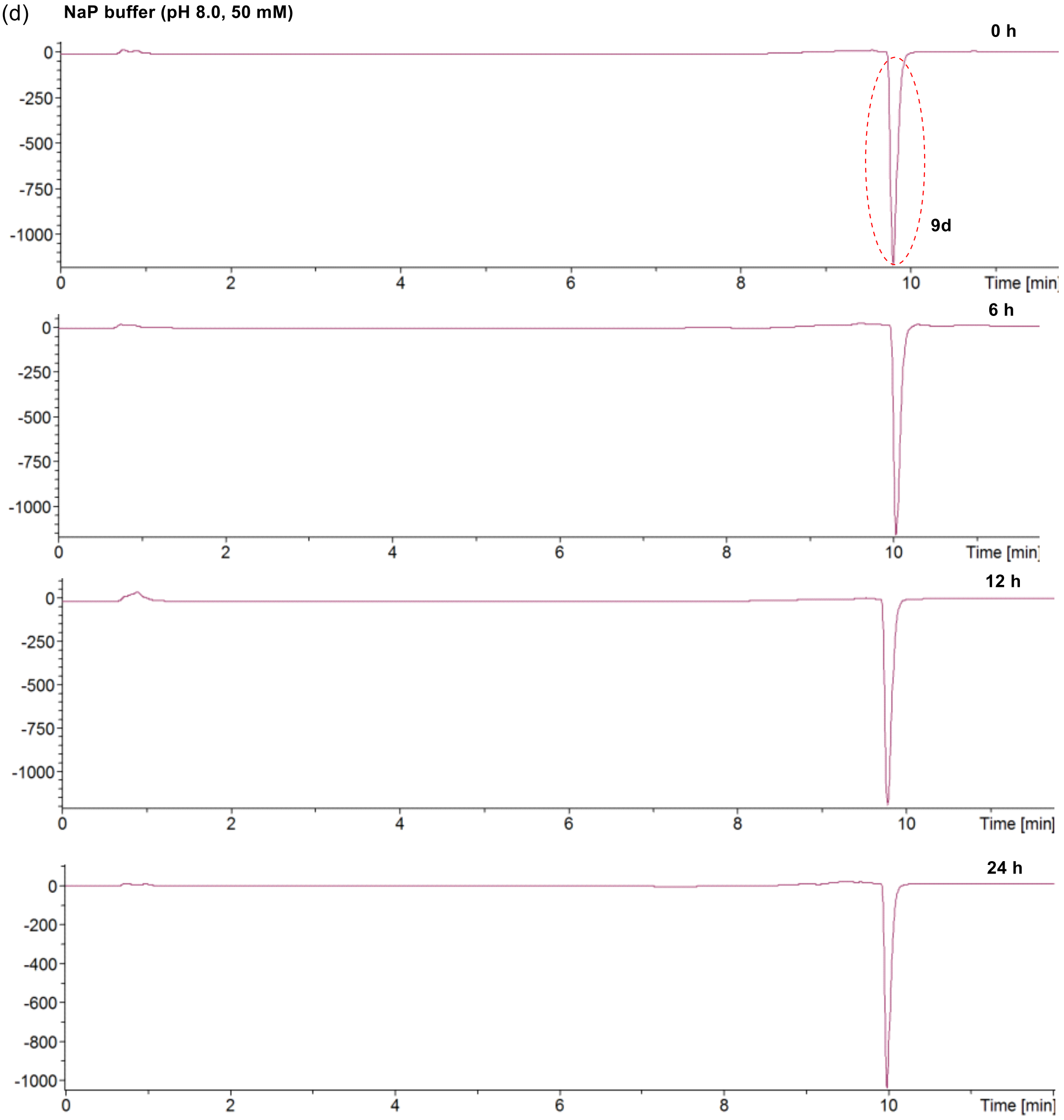

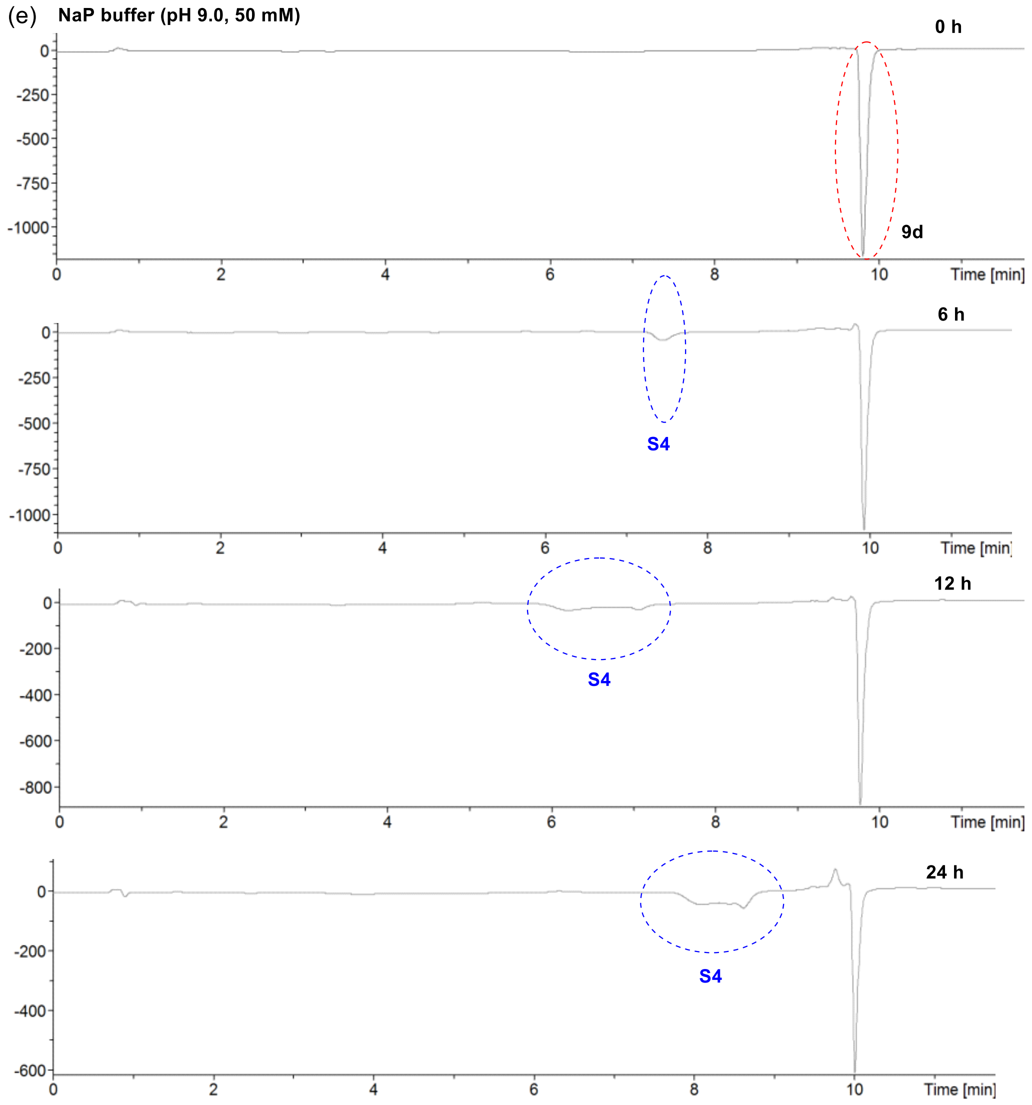

Supplementary Fig. 93. (a) LC data of LDM$_{C-K}$ reagent **9d** at pH 6.0 after 0, 6, 12, and 24 h. (b) LC data of LDM$_{C-K}$ reagent **9d** at pH 7.0 after 0, 6, 12, and 24 h. (c) LC data of LDM$_{C-K}$ reagent **9d** at pH 7.4 after 0, 6, 12, and 24 h. (d) LC data of LDM$_{C-K}$ reagent **9d** at pH 8.0 after 0, 6, 12, and 24 h. (e) LC data of LDM$_{C-K}$ reagent **9d** at pH 9.0 after 0, 6, 12, and 24 h.

8.3 pH screening

Supplementary Table 9. pH screening with β-lactoglobulin A and LDM$_{C\text{-}K}$ reagent 9d.

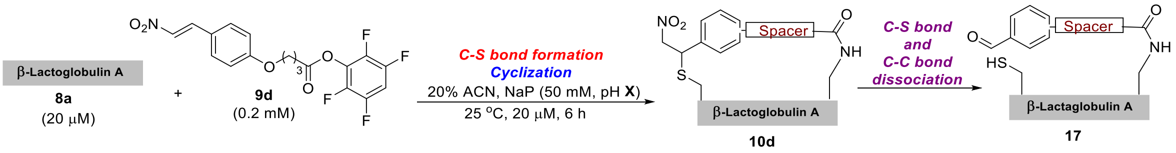

Entry	pH (X)	% Conversion[a] (10d)	% Conversion[a] (17)
1	6.0	28	0
**2**	**7.0**	**>99**	**0**
3	7.4	82 (mono), 18 (bis)	0
4[b]	8.0	66 (mono), 16 (bis)	5 (mono), 12 (bis)
5[b]	9.0	51 (mono), 17 (bis)	25 (bis)

[a] % Conversion based on ESI-MS. [b] In this case the retro-Henry product (C-S and C-C bond dissociation) **17** (mono) and **17b** (bis) was also observed.

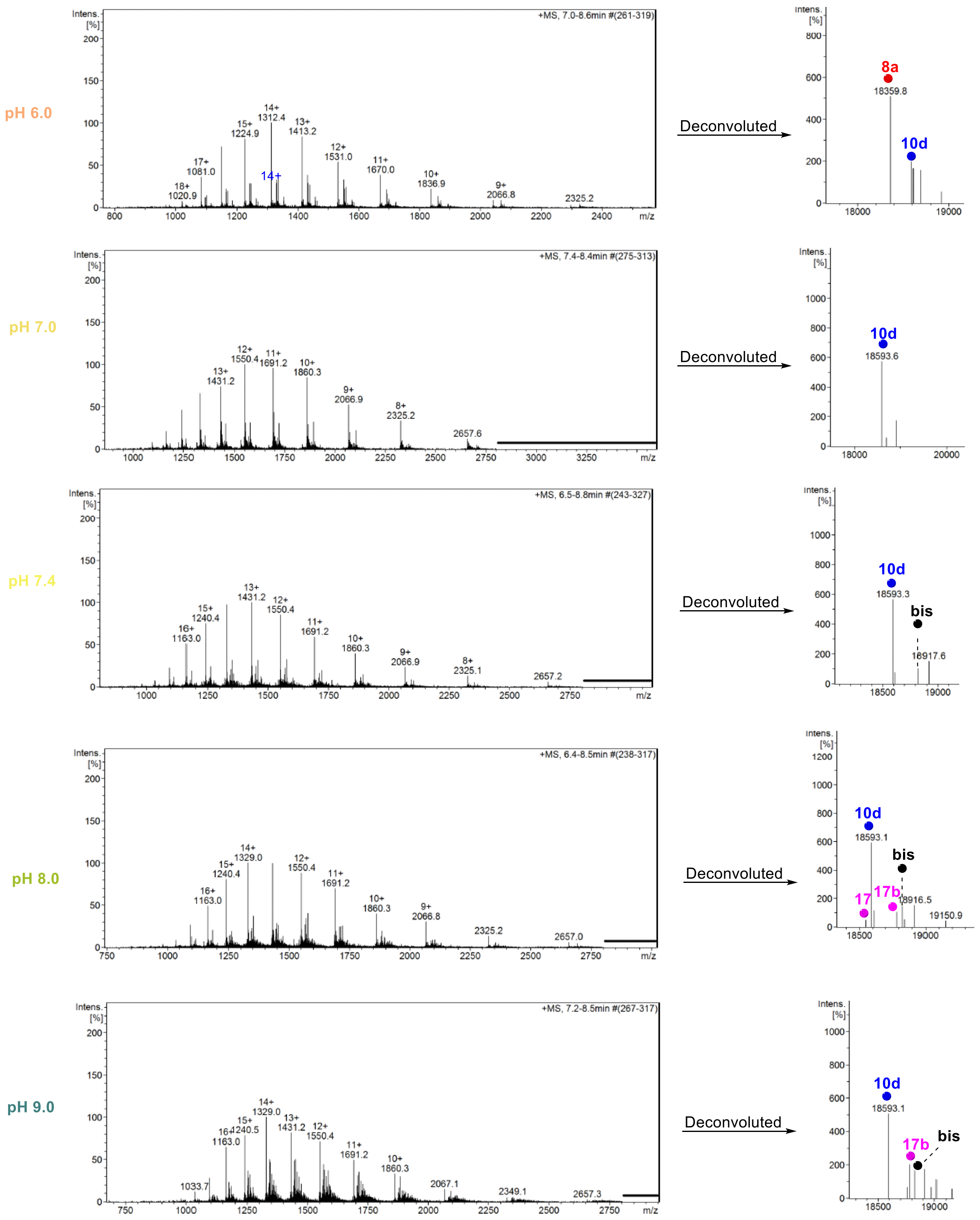

Supplementary Fig. 94. ESI-MS spectrum of labeled β-lactoglobulin enabled by LDM$_{C\text{-}K}$ reagent **9d** (10 equiv.) at various pH (pH 6.0-9.0).

8.4 Concentration screening

Supplementary Table 10. Concentration screening with β-lactoglobulin A and LDM$_{C\text{-}K}$ reagent 9d.

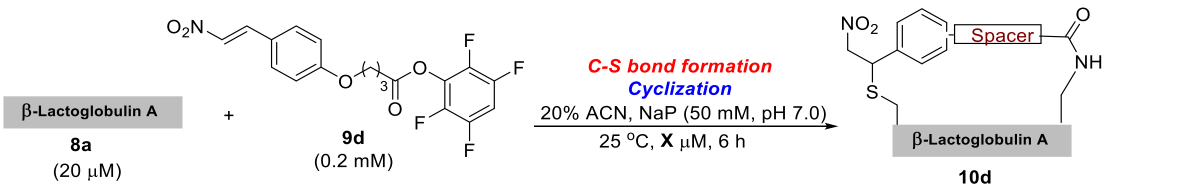

Entry	Concentration (μM)	% Conversion[a]
1	5.0	85
2	10.0	87
**3**	**20.0**	**>99**
4	50.0	81 (mono), 8 (bis)
5	73.0	89 (mono), 10 (bis)

[a] % Conversion based on ESI-MS.

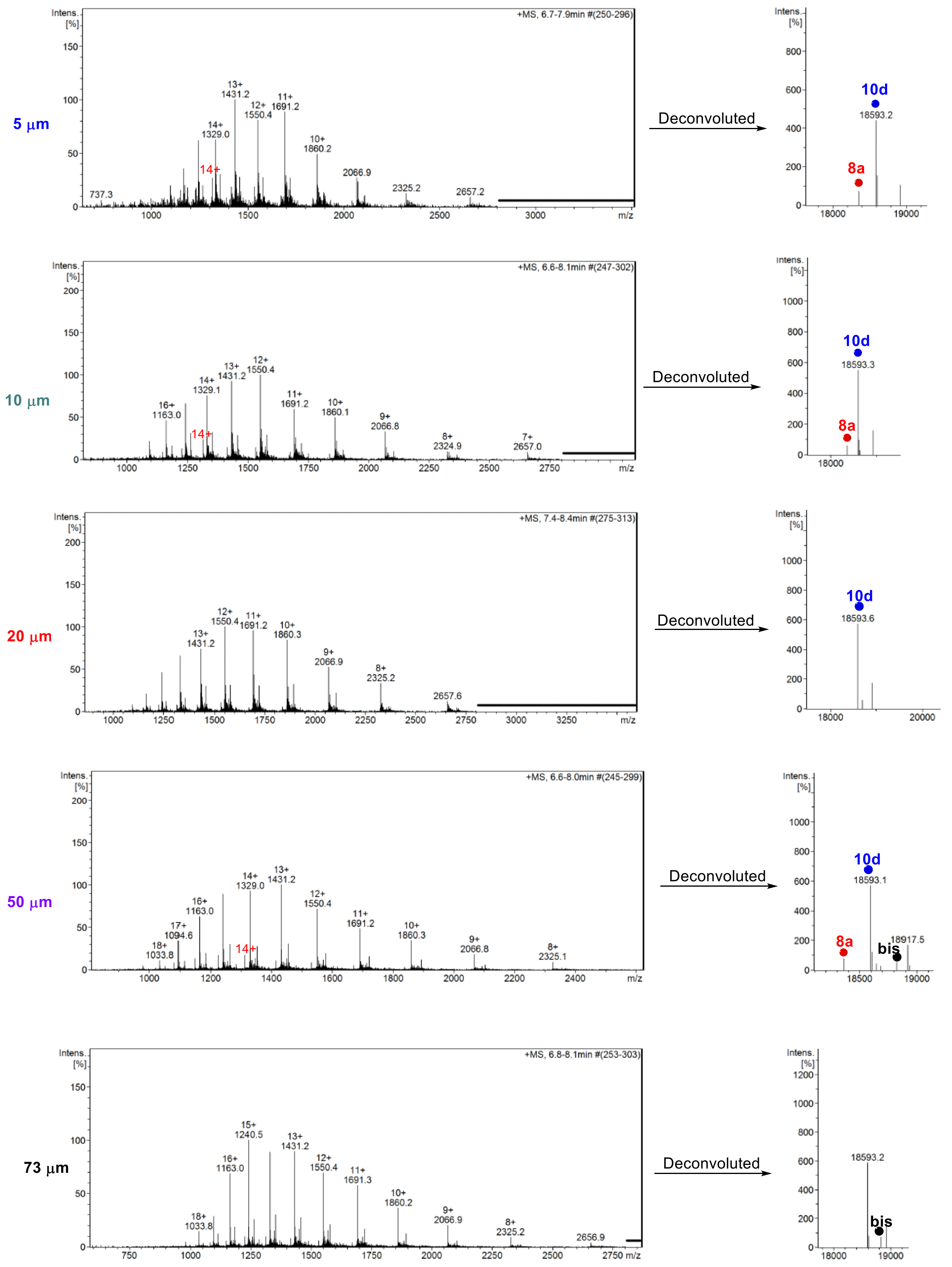

Supplementary Fig. 95. ESI-MS spectrum of labeled β-lactoglobulin A enabled by $LDM_{C\text{-}K}$ reagent **9d** (10 equiv.) at various concentrations (5 µM- 73 µM).

8.5 ^{19}F NMR data of labeled β-lactoglobulin A

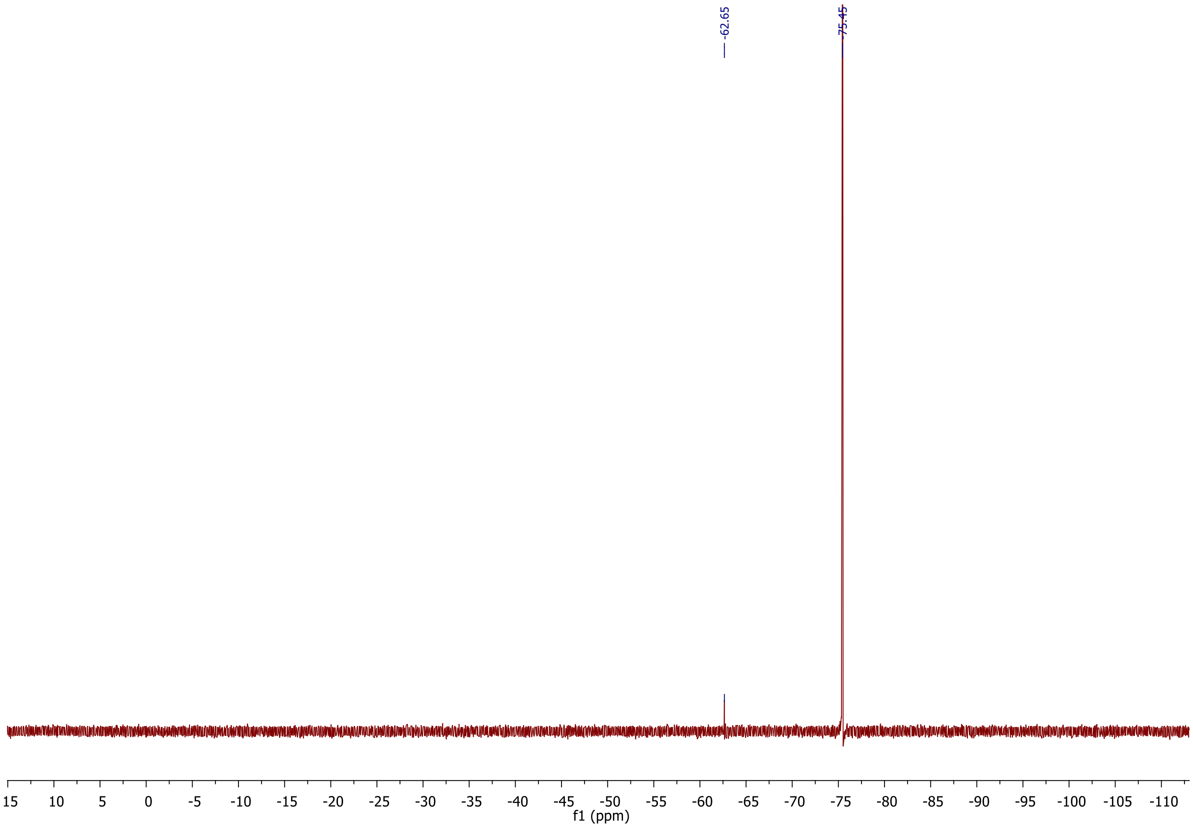

Supplementary Fig. 96. ^{19}F-NMR spectrum of labeled BLGA **24 (**a sharp signal at -62.65 ppm, internal standard: trifluoroacetic acid (0.2 mM) signal at -75.45 ppm). Sample was recorded in phosphate buffer (0.1 M, pH 7.0):D_2O (9:1).

8.6 Fluorescence data of labeled β-lactoglobulin A

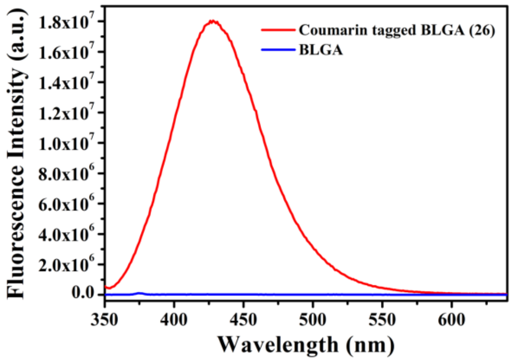

Supplementary Fig. 97. Steady-state fluorescence spectra of coumarin tagged BLGA (**26**) in phosphate buffer (0.1 M, pH 7.0). **26** exhibits emission band peaked at 428 nm (excitation at 333 nm). For MS data, see Supplementary Fig. **73**.

8.7 Circular dichroism spectra of proteins

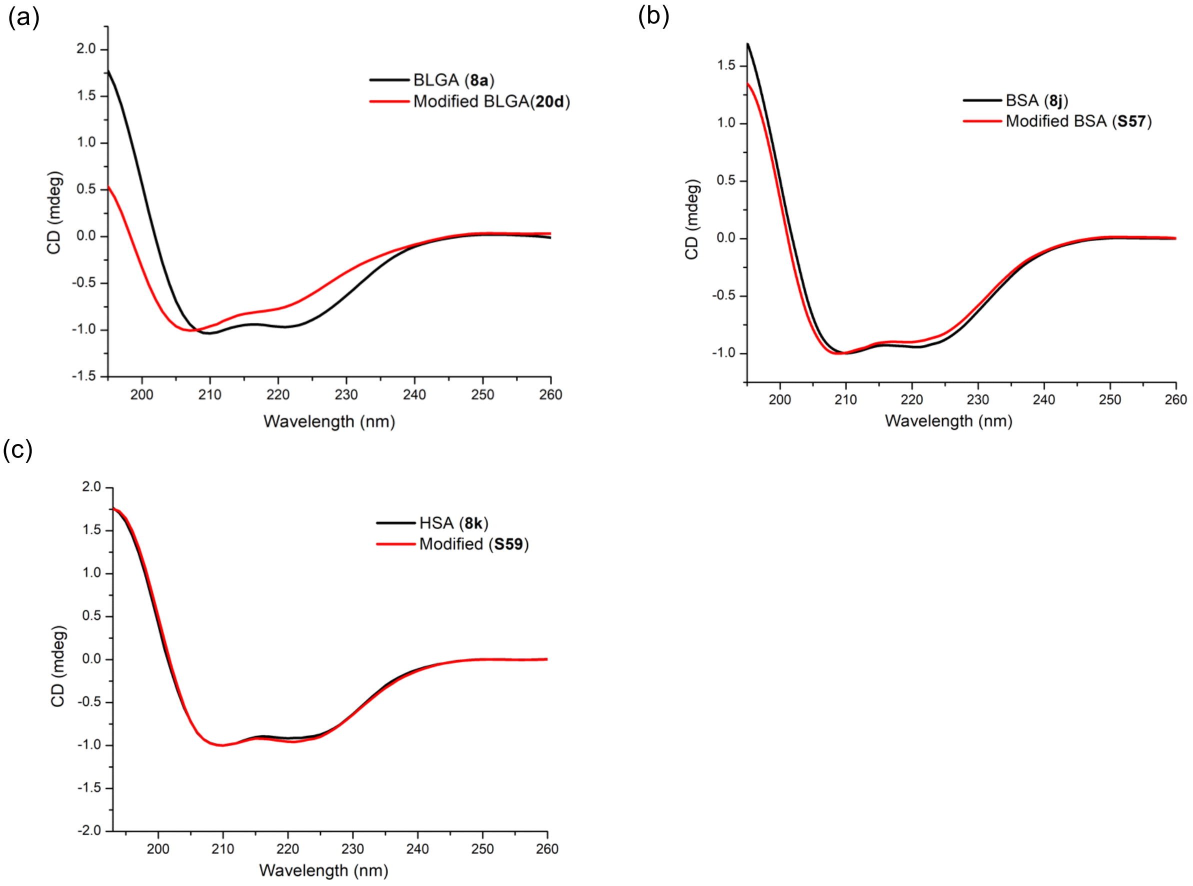

Supplementary Fig. 98. Effect of the labeling on structure of proteins. (a) Circular Dichroism (CD) spectra of BLGA **8a** (black line), modified BLGA **20d** (red line) at concentration 0.1 mg/ml. (b) Circular Dichroism (CD) of BSA **8j** (black line), modified BSA **S57** (red line) at concentration 0.1 mg/ml. (c) Circular Dichroism (CD) of HSA **8k** (black line), modified HSA **S59** (red line) at concentration 0.1 mg/ml.

8.8a Reaction of peptides (competing nucleophile) with LDM$_{C\text{-}K}$ reagent 9d

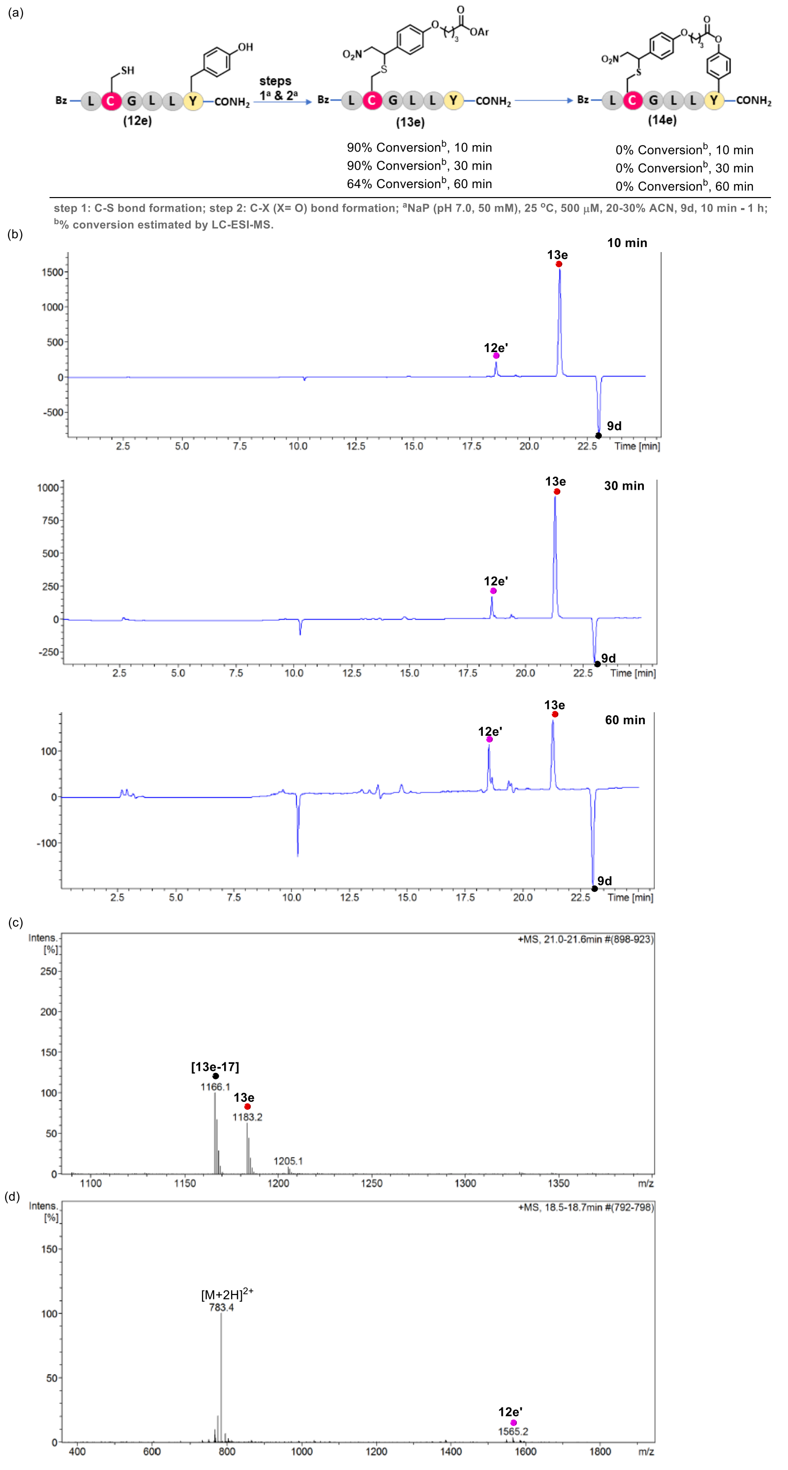

[a]NaP (pH 7.0, 50 mM), 30% ACN, 25 °C, 0.50 mM, 60 min

[b]% Conversion of **13e** and **14e** were esimated on the basis of **12e** using LC-ESI-MS.

Under the physiological reaction condition oxidation of **12e** results into **12e'** (disulfide), which limit the conversion of **13e** and **14e.**

Supplementary Fig. 99. (a) C-S bond formation and cyclization for peptide containing Cys (i[th]) and Tyr (i+4) **12e** enabled by LDM$_{C\text{-}K}$ reagent **9d**. (b) LC data showing C-S bond formation and cyclization for peptide containing Cys (i[th]) and Tyr (i+4th) enabled by reagent **9d** for different interval time (10-60 min). (c) ESI-MS data for Michael adduct **13e**. (d) ESI-MS data for **12e**'.

(a)

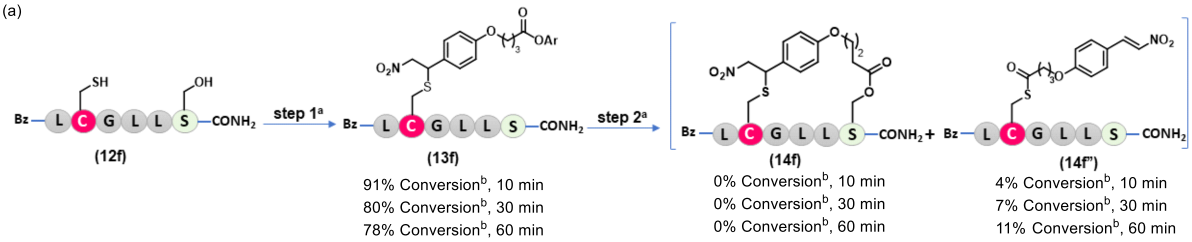

step 1: C-S bond formation; step 2: C-X (X= O) bond formation; [a]NaP (pH 7.0, 50 mM), 25 °C, 500 μM, 20-30% ACN, 9d, 10 min - 1 h; [b]% conversion estimated by LC-ESI-MS.

(b)

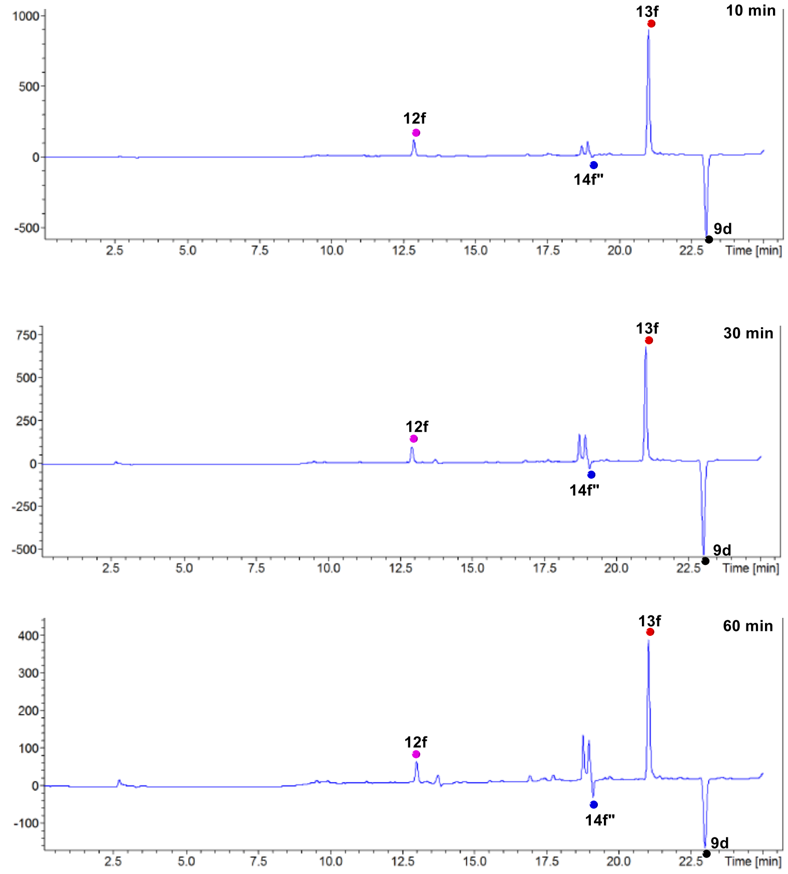

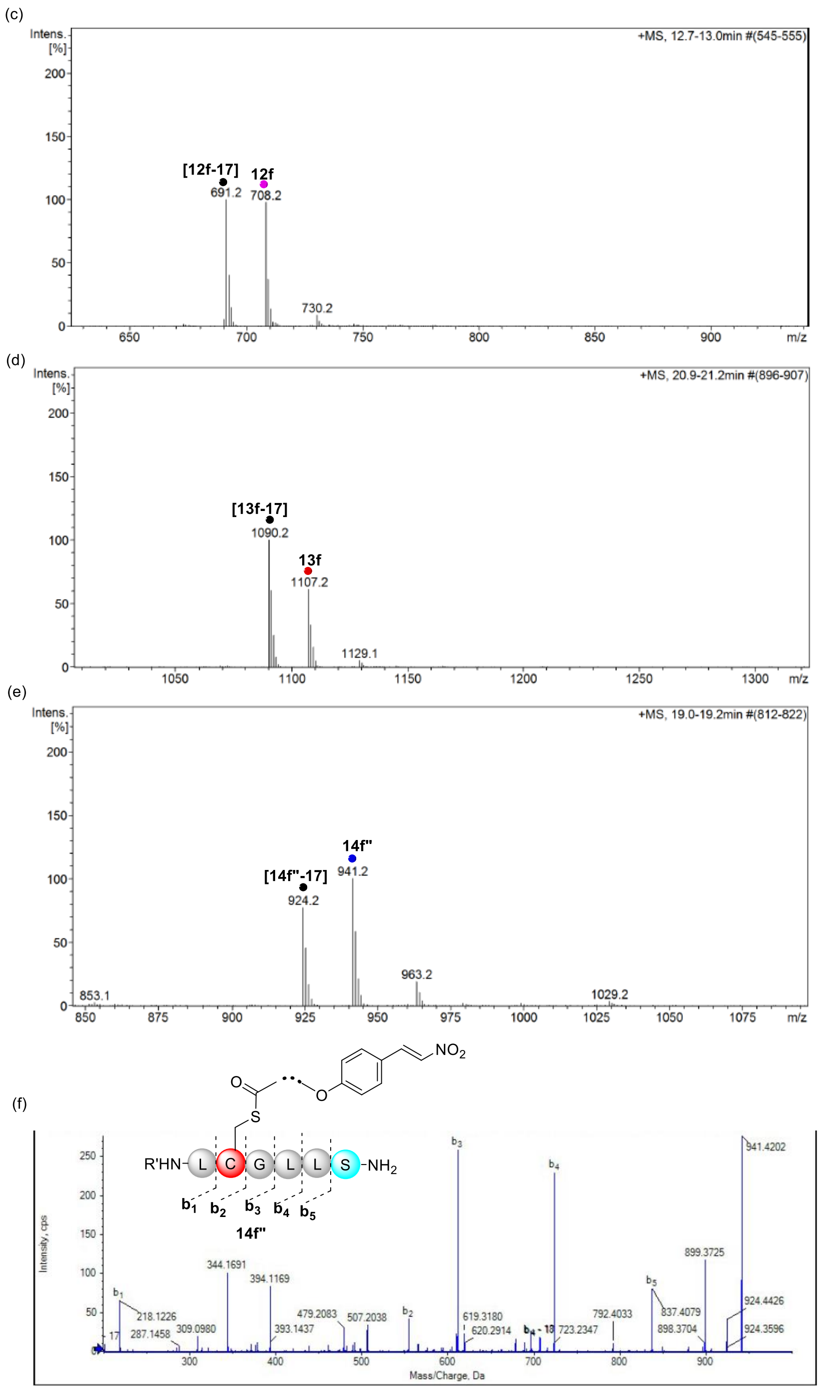

Supplementary Fig. 100. (a) C-S bond formation and cyclization for peptide containing Cys (ith) and Ser (i+4) **12f** enabled by LDM$_{C-K}$ reagent **9d**. (b) LC data showing C-S bond formation and cyclization for peptide containing Cys (ith) and Ser (i+4th) enabled by reagent **9d** for different interval time (10-60 min). (c) ESI-MS data for **12f**. (d) ESI-MS data for Michael adduct **13f**. (e) ESI-MS data for S-acylation **14f"**. (f) MS-MS data for S-acylation **14f"**.

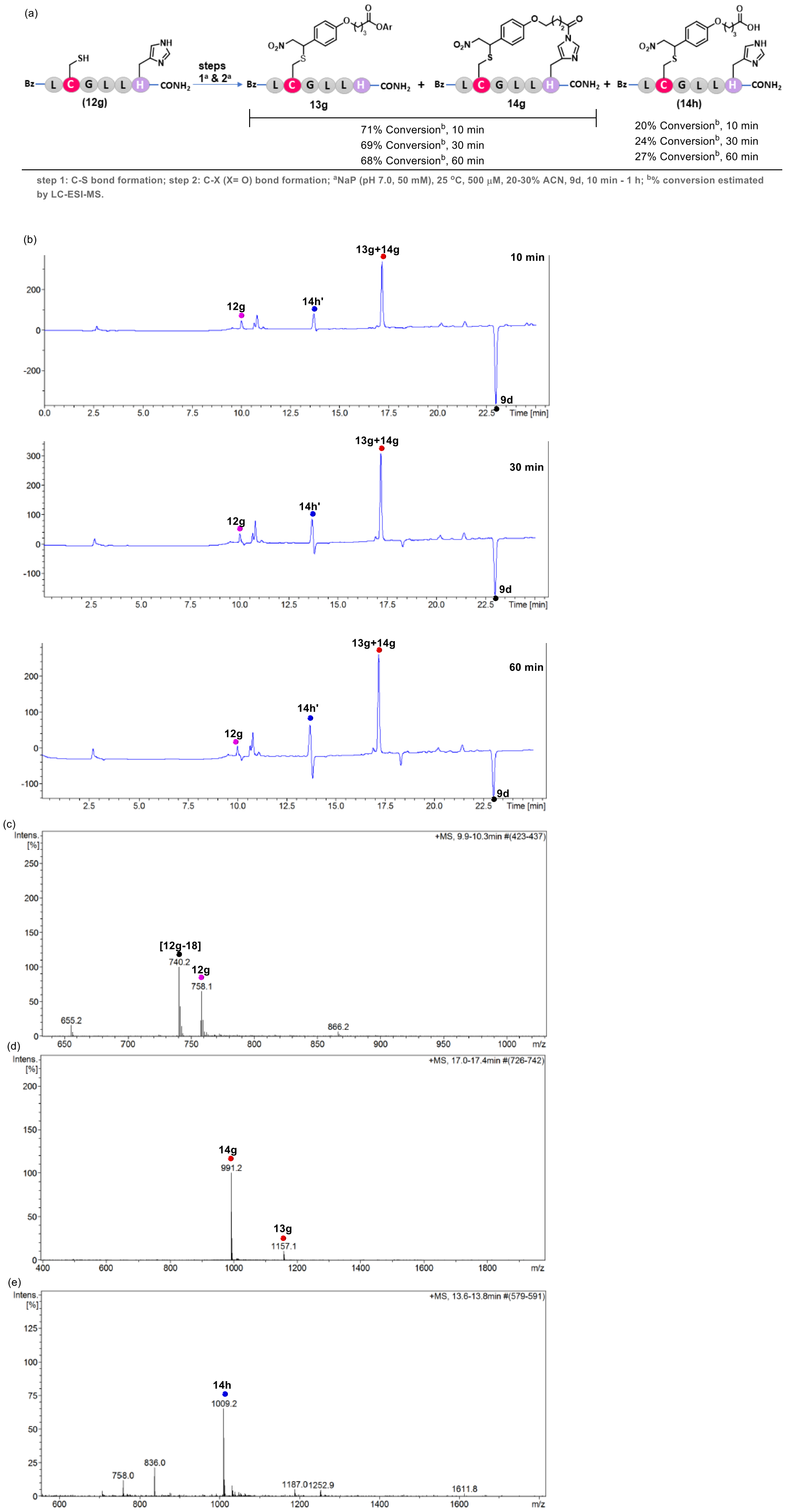

Supplementary Fig. 101. (a) C-S bond formation and cyclization for peptide containing Cys (ith) and His (i+4) **12g** enabled by LDM$_{C\text{-}K}$ reagent **9d**. (b) LC data showing C-S bond formation and cyclization for peptide containing Cys (ith) and His (i+4th) enabled by reagent **9d** for different interval time (10-60 min). (c) ESI-MS data for **12g.** (d) ESI-MS data for Michael adduct **13g** and cyclization **14g.** (e) ESI-MS data for hydrolysed Michael adduct **14h**.

8.8b C-S and C-C bond cleavage in peptides

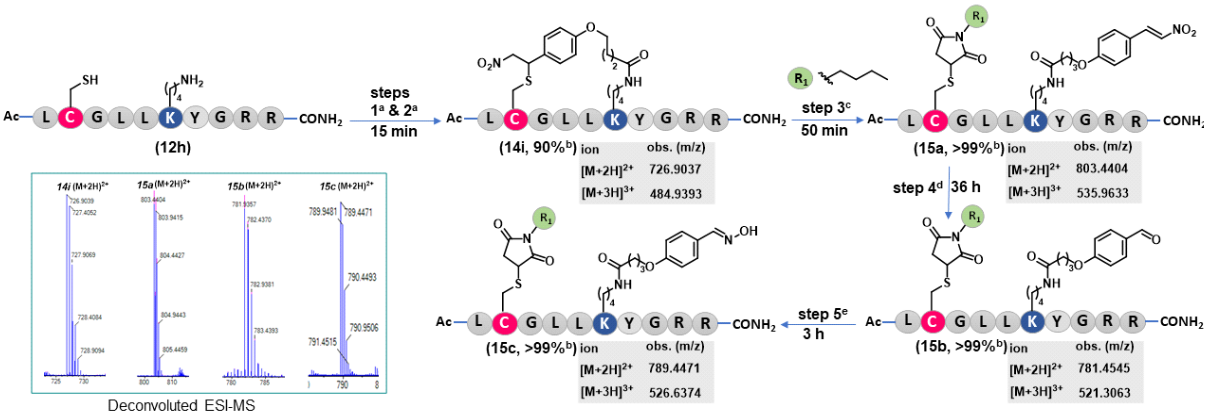

step 1: C-S bond formation; step 2: C-X (X= N, O) bond formation; step 3: C-S bond dissociation and reformation; step 4: nitroolefin to aldehyde formation (retro-Henry); step 5: oxime formation; [a]NaP (pH 7.0, 50 mM), 25 °C, 500 μM, 20-30% ACN, **9d**, 15 min; [b]% conversion estimated by LC-ESI-MS; [c]n-butyl maleimide (50 equiv.),10% ACN, 25 °C, 50 min; [d]re-adjustment of pH (~9.0), 25 °C, 36 h; [e]re-adjustment of pH (~6.5), $NH_2OH.HCl$ (100 equiv.), 25 °C, 3 h.

Supplementary Fig. 102. Linchpin-directed modification of Lys and downstream synthetic manipulations in a peptide. The selected MS data for **14i**, **15a**, **15b**, and **15c** are given in the inset.

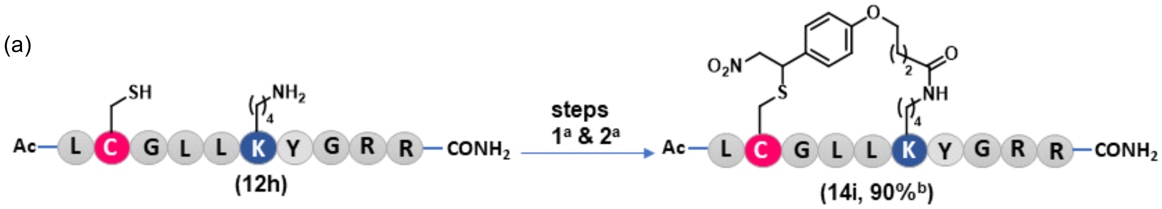

step 1: C-S bond formation; step 2: C-N bond formation; [a]NaP (pH 7.0, 50 mM), 25 °C, 500 μM, 20-30% ACN, 15 min; [b]% conversion estimated by LC-ESI-MS;

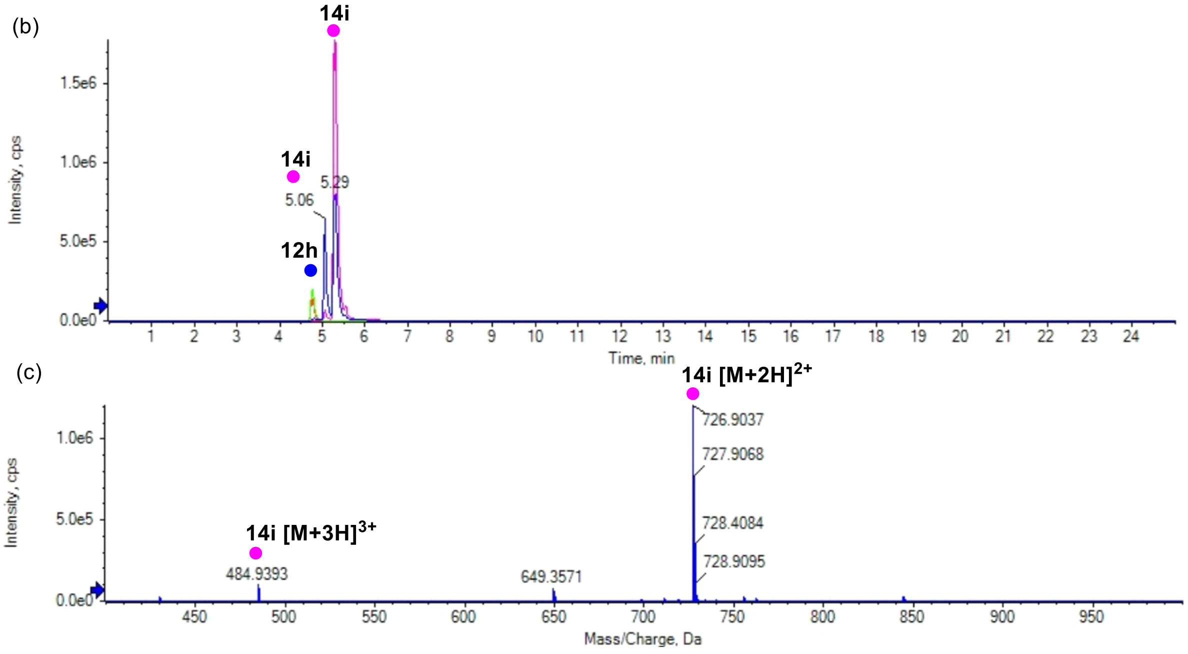

Supplementary Fig. 103. (a) C-S bond formation and cyclization with peptide **12h** enabled by $LDM_{C\text{-}K}$ reagent **9d** resulted into **14i**. (b) XIC data for **14i**. (c) ESI-MS data for cyclized product **14i**.

(a)

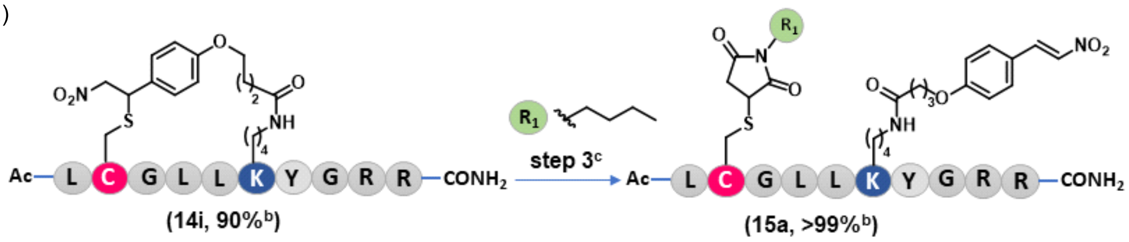

step 3: C-S bond dissociation and reformation; [b]% conversion estimated by LC-ESI-MS; [c]n-butyl maleimide (50 equiv.),10% ACN, 25 °C, 50 min;

(b)

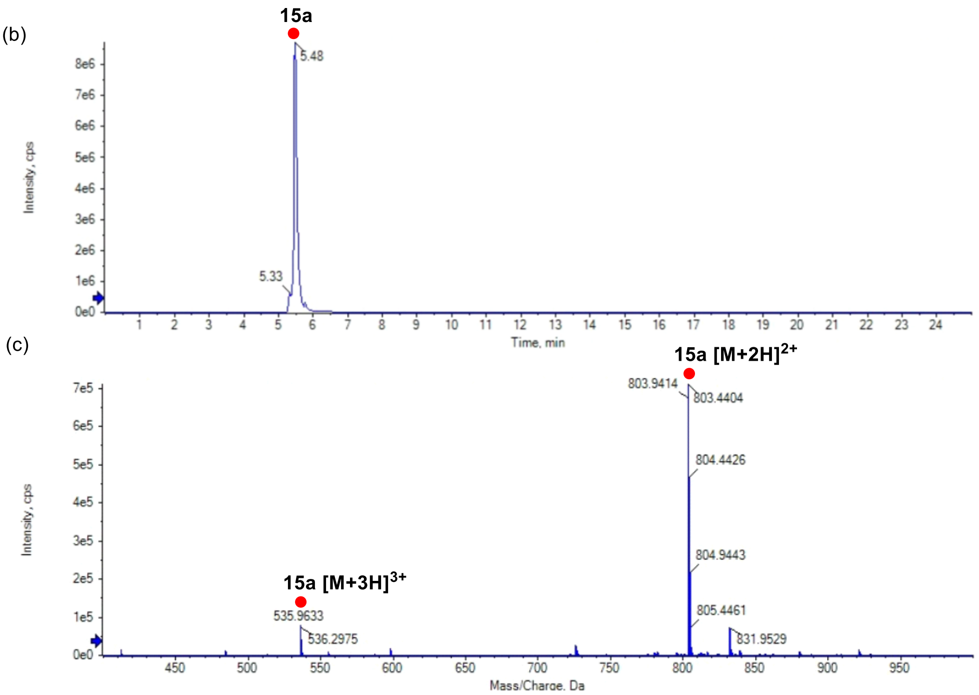

Supplementary Fig. 104. (a) C-S bond dissociation and formation enabled by maleimide derivative from **14i** to yield **15a**. (b) XIC data for **15a**. (c) ESI-MS data **15a**.

(a)

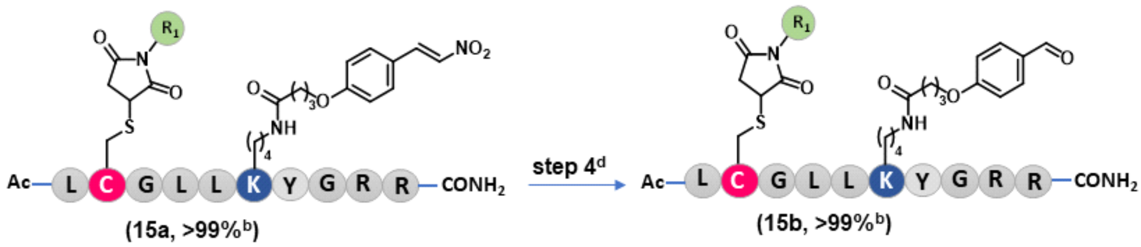

step 4: nitroolefin to aldehyde formation (retro-Henry); [b]% conversion estimated by LC-ESI-MS; [d]re-adjustment of pH (~9.0), 25 °C, 36 h.

(b)

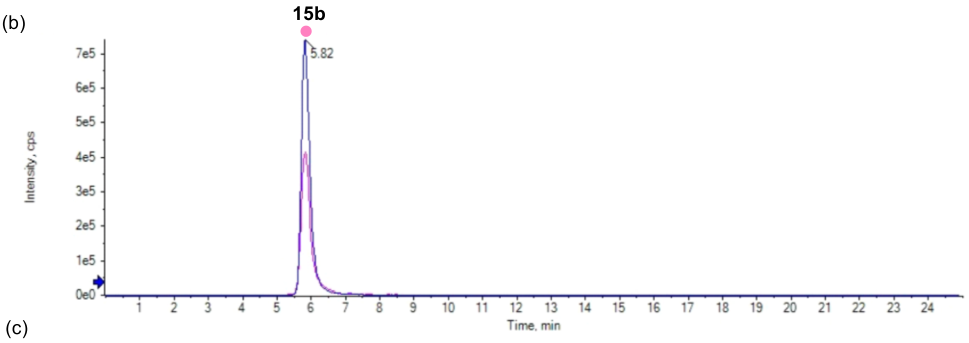

(c)

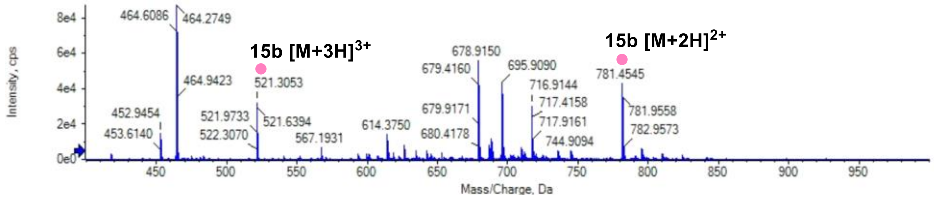

Supplementary Fig. 105. Generation of Aldehyde (**15b**) from nitro-olefin containing peptide **15a** by readjustment of pH. (a) XIC data for aldehyde formation **15b**. (b) ESI-MS data for.

(a)

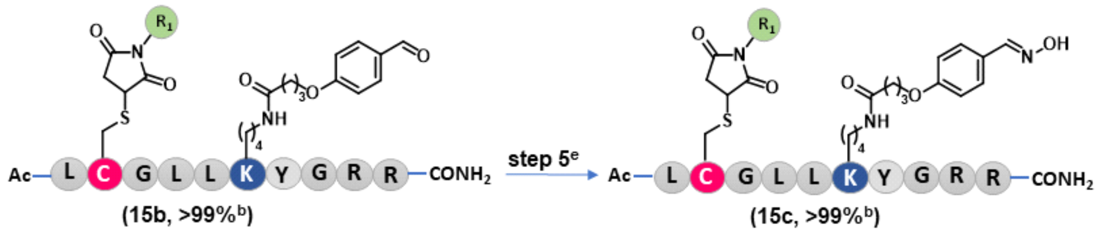

step 5: oxime formation; b% conversion estimated by LC-ESI-MS; ere-adjustment of pH (~6.5), NH_2OH.HCl (200 equiv.), 25 °C, 3 h.

(b)

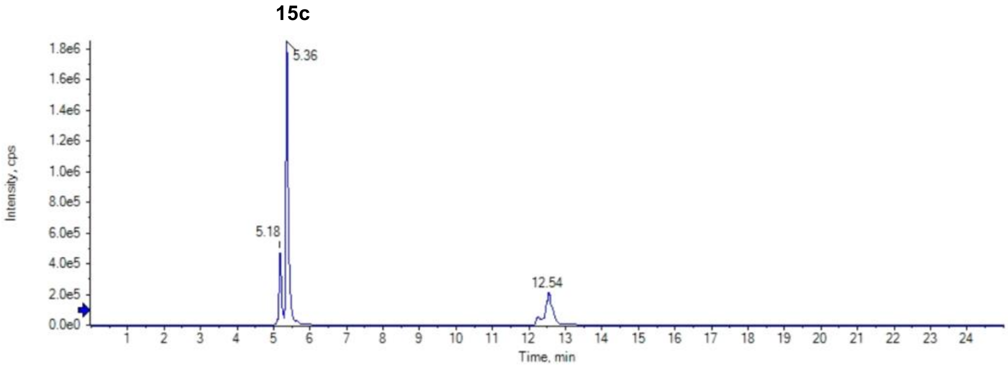

(c)

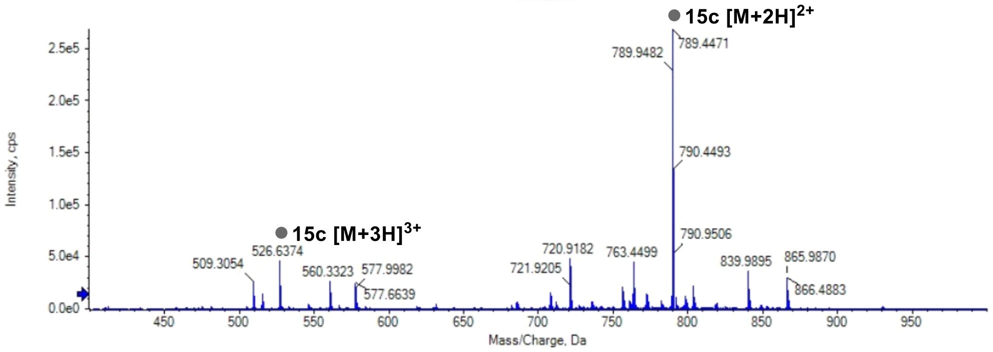

Supplementary Fig. 106. Oxime formation **15c**. resulted from **15b** and NH_2OH (a) XIC data for **15c**. (b) ESI-MS data for **15c**.

8.8c Linchpin dissociation in cyclic protein (BLGA)

Supplementary Table 11: Stability of cyclic BLGA at different pH as a function of time.

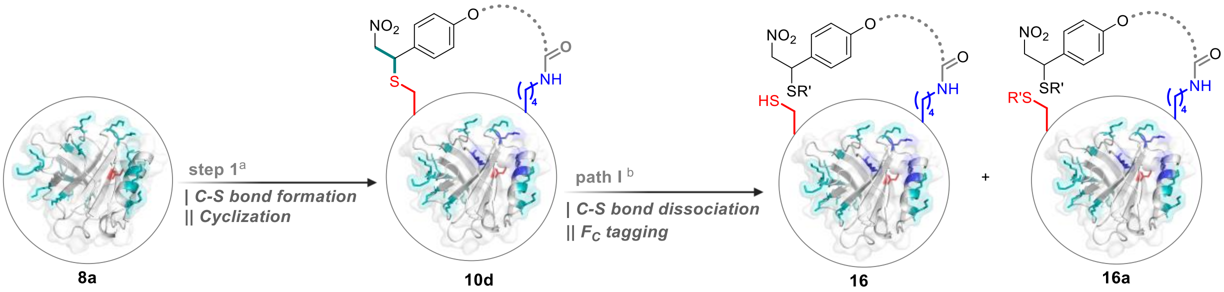

[a]NaP buffer (pH 7.0, 50 mM), 20 μM, 20% ACN, LDM_{C-K} reagent (**9d**), 6 h, >99% conv. [b]NaP buffer (pH X, 50 mM), 20 μM, β-mercaptoethanol (**15**,100 equiv.), 3 h [R′ = $(CH_2)_2OH$)]

Entry	pH (X)	% Conversion[a] (with time in min)					
		30		60		180	
		16	16a	16	16a	16	16a
1	7.0	15	0	22	0	30	22
2	8.0	48	0	44	8	43	10
3	9.0	42	6	43	11	46	14

[a] % Conversion based on ESI-MS.

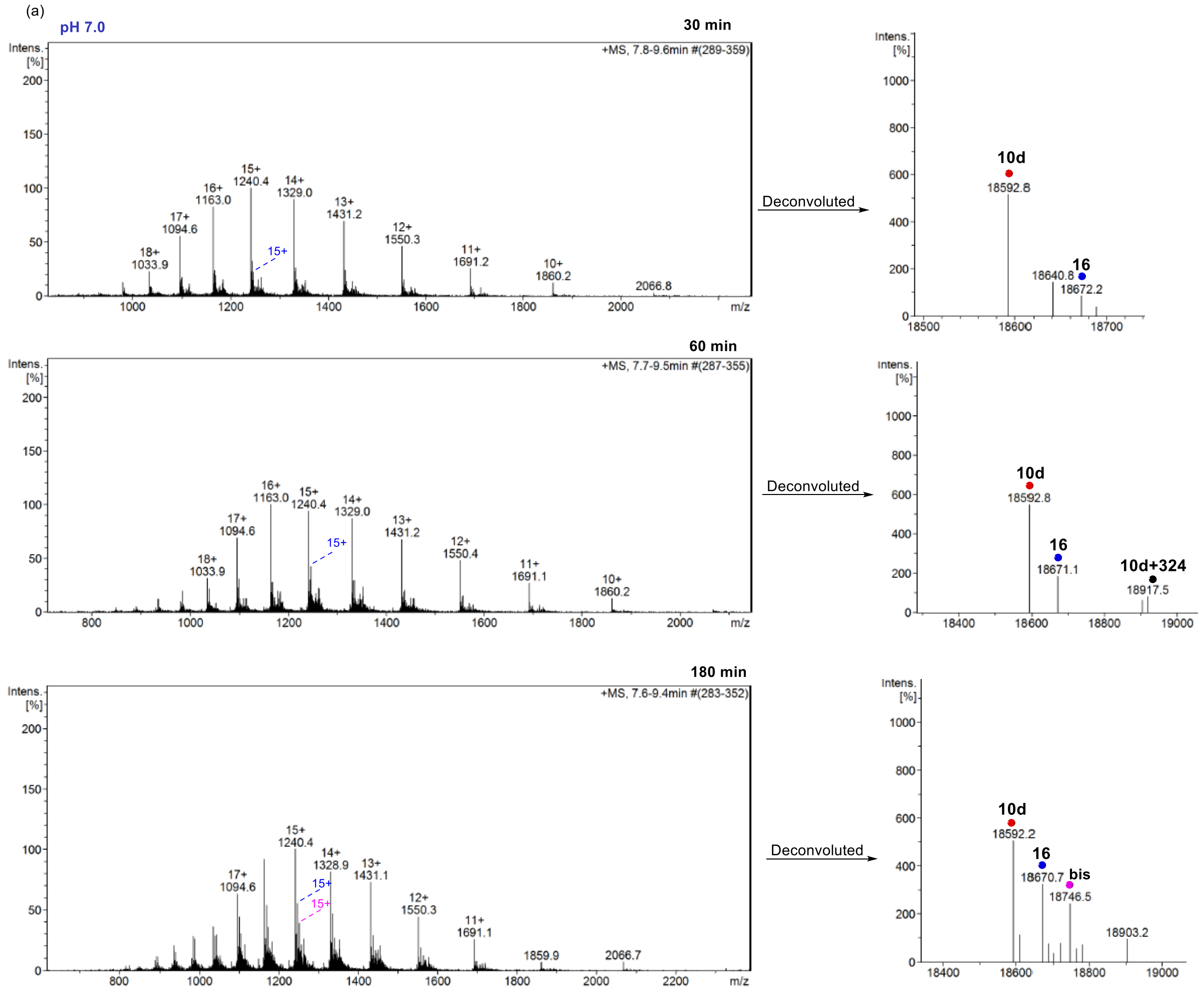

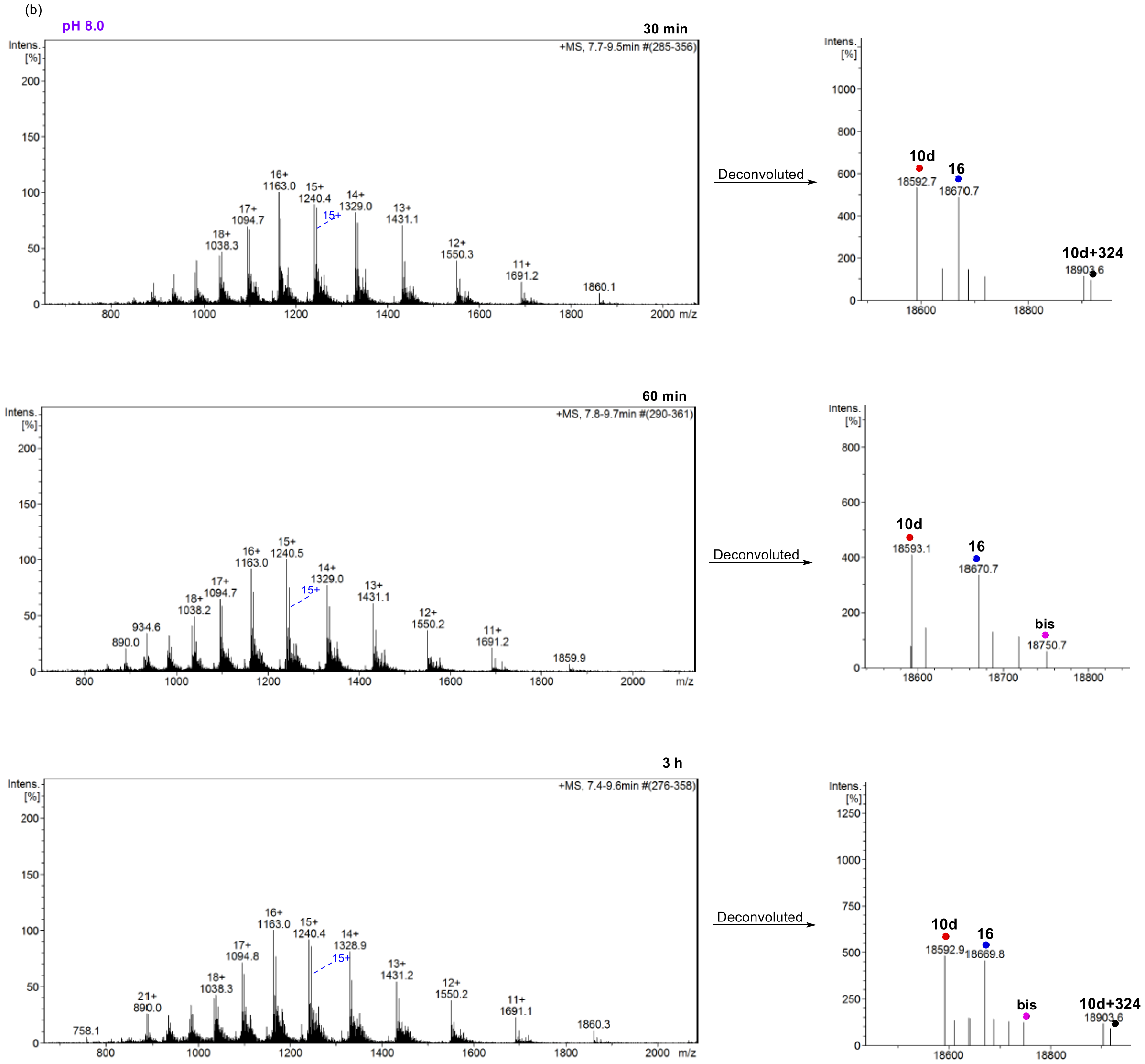
(b)
pH 8.0
30 min
+MS, 7.7-9.5min #(285-356)
16+ 1163.0
15+ 1240.4
14+ 1329.0
17+ 1094.7
18+ 1038.3
13+ 1431.1
12+ 1550.3
11+ 1691.2
1860.1
Deconvoluted
10d 18592.7
16 18670.7
10d+324 18903.6
60 min
+MS, 7.8-9.7min #(290-361)
15+ 1240.5
16+ 1163.0
14+ 1329.0
17+ 1094.7
18+ 1038.2
13+ 1431.1
934.6
890.0
12+ 1550.2
11+ 1691.2
1859.9
Deconvoluted
10d 18593.1
16 18670.7
bis 18750.7
3 h
+MS, 7.4-9.6min #(276-358)
16+ 1163.0
15+ 1240.4
14+ 1328.9
17+ 1094.8
18+ 1038.3
21+ 890.0
13+ 1431.2
12+ 1550.2
11+ 1691.1
758.1
1860.3
Deconvoluted
10d 18592.9
16 18669.8
bis
10d+324 18903.6

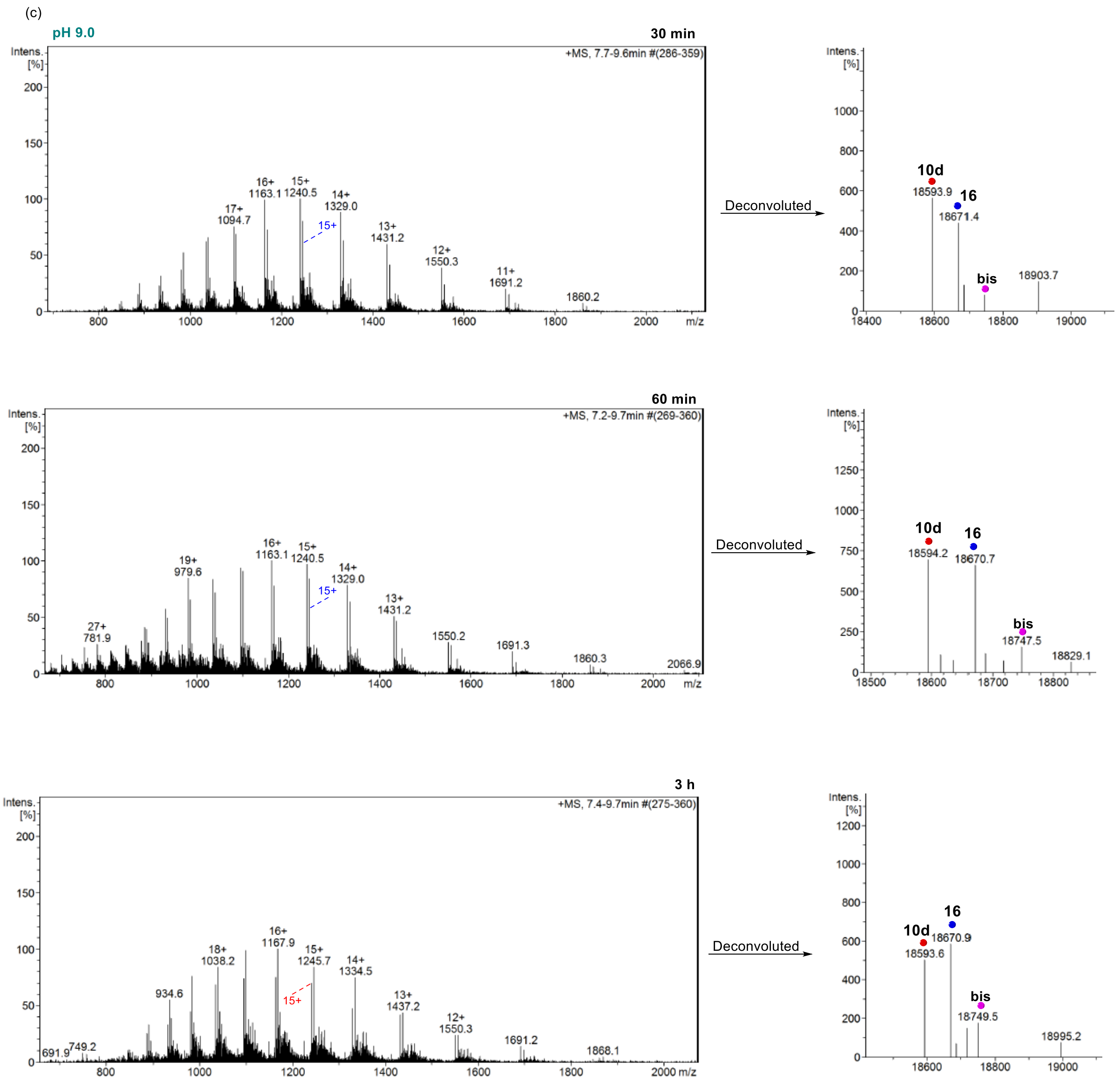

Supplementary Fig. 107. ESI-MS data for retro-Michael reaction of stapled construct at (a) pH 7.0 from 30 min to 3 h, (b) pH 8.0 from 30 min to 3 h, (c) pH 7.0 from 30 min to 3 h.

8.9a Enrichment of protein from E. Coli BL21 cell-lysate (without HSA) using coumarin tag

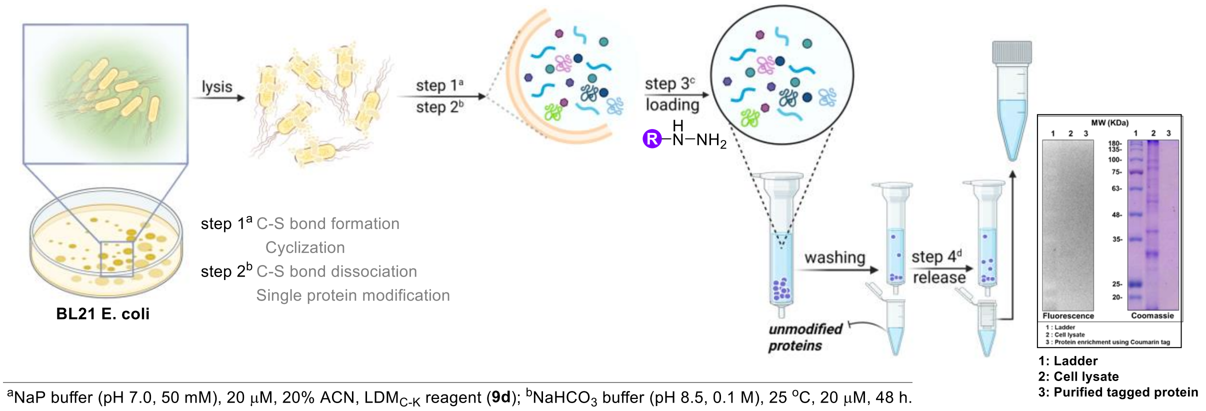

[a]NaP buffer (pH 7.0, 50 mM), 20 μM, 20% ACN, $LDM_{C\text{-}K}$ reagent (**9d**); [b]$NaHCO_3$ buffer (pH 8.5, 0.1 M), 25 °C, 20 μM, 48 h. [c]NaP buffer (pH 7.0, 50 mM), 25 °C, 24 h; [d]NaP buffer (pH 6.5, 50 mM), 25 °C, $RONH_2$ (**23**, 100 equiv.), 10% ACN, 24 h.

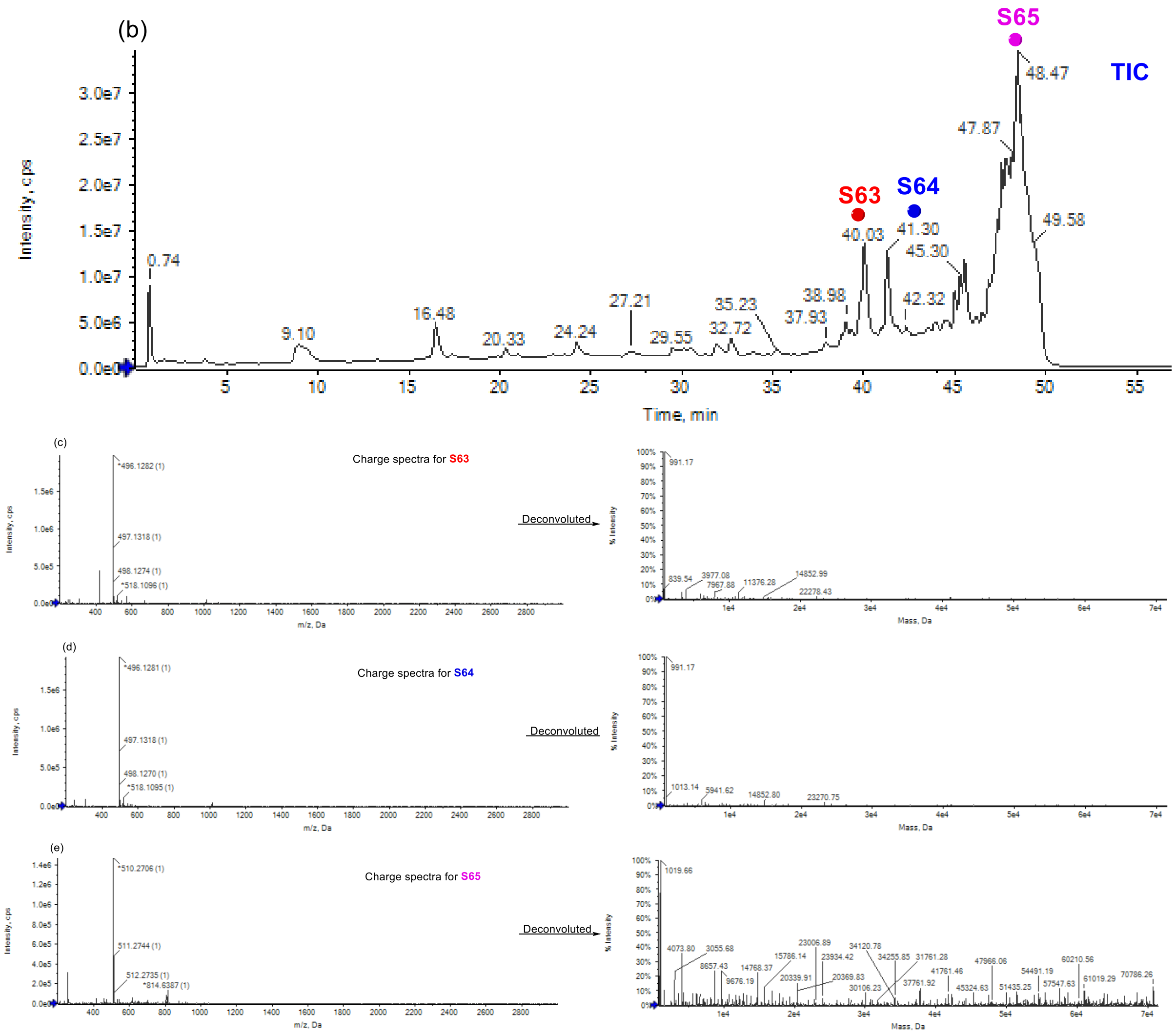

(f)

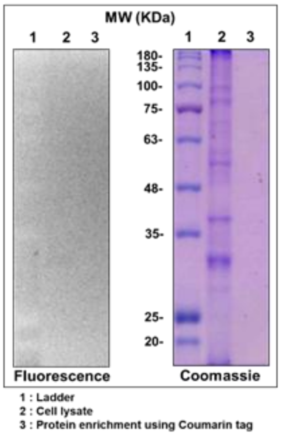

Supplementary Fig. 108. (a) Modification and enrichment of proteins in cell lysate derived from BL21 cells, E. coli enabled by LDM_{C-K} reagent **9d** (10 equiv.). (b) TIC showing a few peaks eluted at different time interval after the treatment with LDM_{C-K} reagent **9d**. The ESI-MS data confirms the absence of protein within the MS detection limits. (c) ESI-MS data for **S63**. (d) ESI-MS data for **S64**. (e) ESI-MS data for **S65**. (f) 10% SDS-PAGE showing fluorescence imaging and Coomassie staining. MW - Molecular Weight, SDS-PAGE: lane 1 - ladder, lane 2 - cell lysate (without HSA), lane 3 - coumarin-tagged purified protein.

8.9b Enzymatic assay of HSA (Human serum albumin)

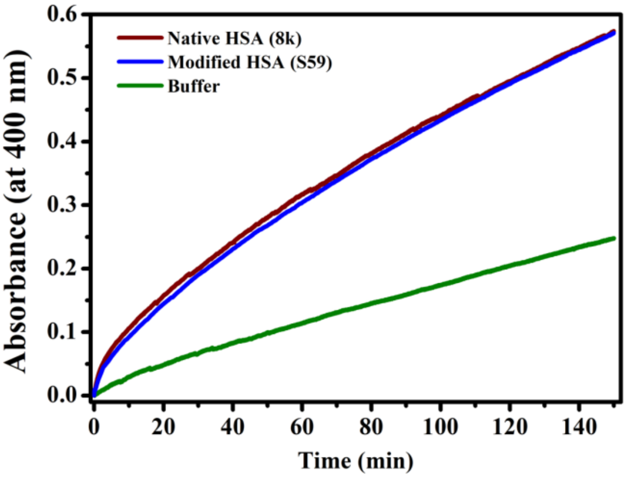

Supplementary Fig. 109. Effect of K199-bioconjugation on the esterase-like activity of HSA. Time dependent UV-Vis spectra was recorded to compare the enzymatic activity of native HSA (**8k**) and labeled HSA (**S59**). Esterase-like activity of HSA before and after the labeling are examined by monitoring the catalytic reactivity of HSA using p-nitrophenyl acetate (*p*NPA) as the substrate. The enzymatic activity of HSA remains unperturbed after the chemical modification.

8.10 Comparative analysis of light chain (LC) and heavy chain (HC) in native and modified trastuzumab

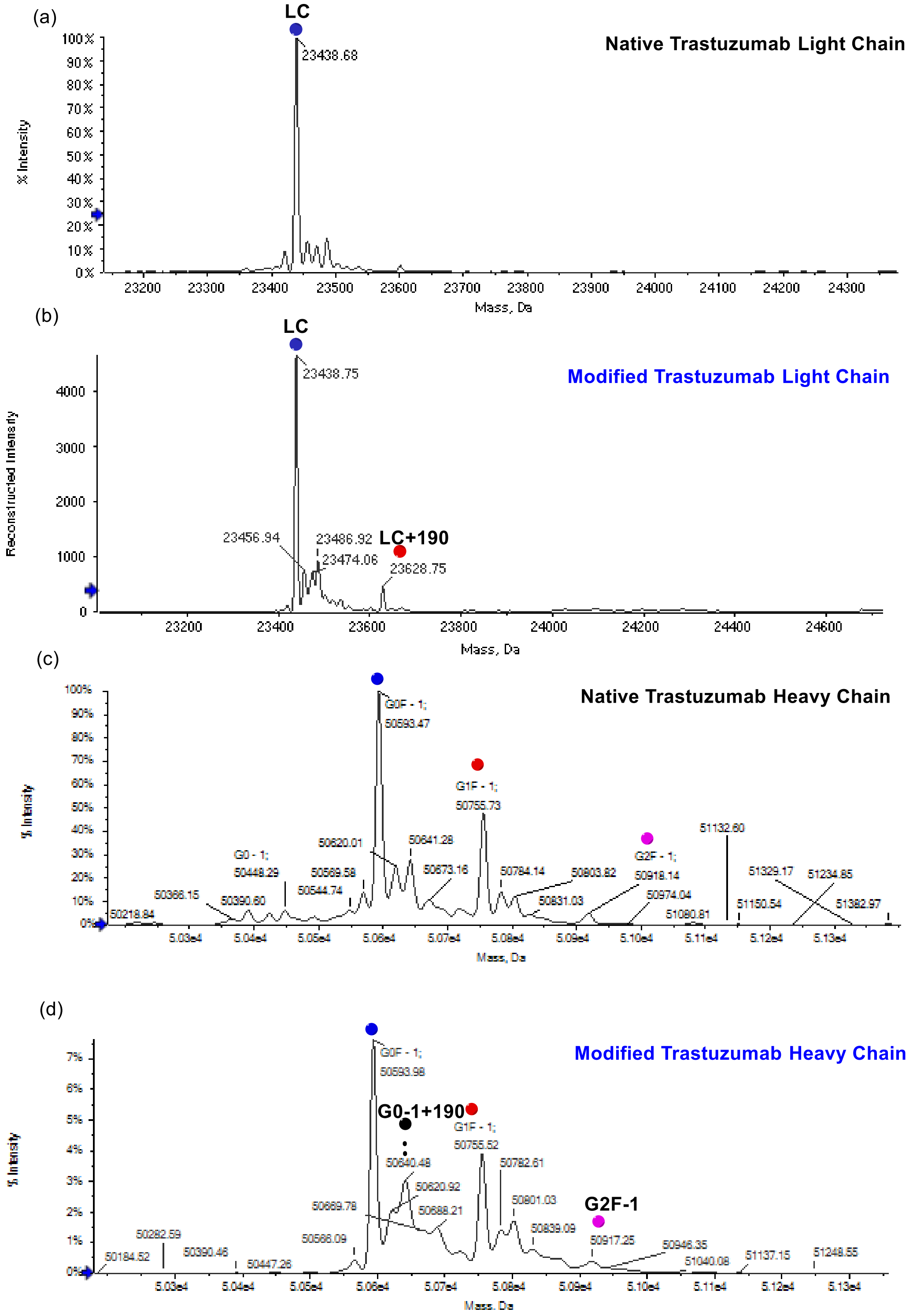

Supplementary Fig. 110. Comparative analysis of light and heavy chain in native and modified trastuzumab by using ESI-MS. (c) ESI-MS data for light chain in native trastuzumab. (d) ESI-MS data for light chain in modified trastuzumab. (c) ESI-MS data for heavy chain in native trastuzumab, showing all the post-translational modification. (d) ESI-MS data for heavy chain in modified trastuzumab, showing all the post-translational modification.

8.11 Experiments with CASLQK tagged Hs-Ubc9

Construction of plasmid for tagged protein production: The pET28a(+)-Hs-Ubc9 plasmid was used to generate a construct capable of adding -CASLQK- hexameric tag to the N-terminus of the protein. Two partially complementary oligonucleotides (5'-CTAGCTGTGCTTCACTCCAGAAAG-3' and 5'-GATCCTTTCTGGAGTGAAGCAGAG-3') providing a NheI and BamHI overhangs respectively at 5' and 3' ends were synthesized and annealed in a thermocycler. The pET28-a(+)-Hs-Ubc9 plasmid was digested with NheI and BamHI restriction enzymes, gel purified, and used in a ligation reaction for 2 h at 25 °C with oligos prepared earlier. The resultant construct (pET28-a(+)-Hs-Ubc9_tag) was transformed into *E. coli* DH5α cells. Isolated plasmids were sent for Sanger sequencing for the correctness of generated clones and confirmation of oligonucleotide addition.

Recombinant protein expression and purification: To express -CASLQK- tagged Hs-Ubc9 protein, pET28-a(+)-Hs-Ubc9_tag construct was transformed into *Escherichia coli* BL21 (DE3) cells. A single transformed colony was inoculated in 5 ml primary culture and kept for overnight growth at 37°C at 192 x g. The secondary culture was initiated by 1% of the primary culture in antibiotic containing media and was allowed to grow at 37°C. The protein production was induced by 100 µM of IPTG was added in secondary culture for induction of recombinant protein for 4 hours at 30°C. Subsequently, the induced culture was centrifuged at 7680 x g for 10 minutes at 4°C. Bacterial pellets were lysed in lysis buffer (50 mM NaH_2PO_4 pH 8.0, 300 mM NaCl) containing 0.2% Triton X-100, 1 mg/mL of lysozyme, 1X PIC (Protease inhibitor cocktail) and 1 mM PMSF (phenyl methane sulfonyl fluoride). The resuspended pellet was incubated on ice for 20 minutes and sonicated (45% amplitude, 10 s on pulse, 10 s off pulse) for 5 minutes on ice. After sonication 10 mM imidazole was added into cell lysate and centrifuged at 17280 x g for 30 minutes at 4°C to pellet down cell debris. The resultant supernatant was incubated with Ni-NTA beads for 1 h at 4°C for binding of His-tagged recombinant protein carrying hexameric -CASLQK- tag. Beads were washed with a wash buffer (50 mM NaH_2PO_4 pH 8.0, 300 mM NaCl, and 30 mM imidazole) to remove non-specifically bound proteins. The recombinant protein was eluted with an elution buffer (50 mM NaH_2PO_4 pH 8.0, 300 mM NaCl, and 250 mM imidazole). Purified and dialysed proteins (Fig. S109) were used for further enzymatic experiments.

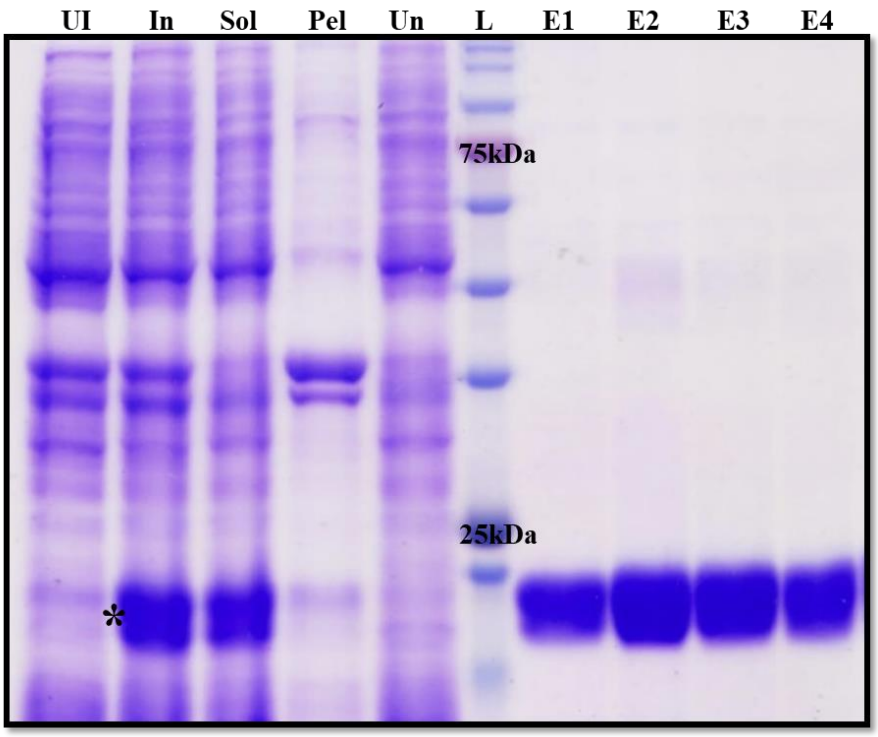

Supplementary Fig. 111. Purification of recombinant Hs-UBC9 carrying an N-terminal hexa-histidine and CASLQK peptide sequence. The asterisk on the left denotes induced protein present in different fractions during purification. Lanes after ladder (L) indicate elutions of recombinant protein from the Ni-NTA column.

$LDM_{C\text{-}K}$ modification and *in vitro* SUMOylation: Subsequently, we treated the His-tagged recombinant Hs-Ubc9 carrying CASLQK tag with the $LDM_{C\text{-}K}$ reagent **9d** to render the mono-labeled CASLQK tagged Hs-Ubc9 (also see ESI Supplementary Fig. 111). Subsequently, we performed in vitro SUMOylation assay and established that the activity remains unperturbed post-bioconjugation. The purified CASLQK-hexameric-tag bearing Hs-Ubc9 and its mono-labeled bioconjugate were used in an *in vitro* SUMOylation reaction. 0.25 µg of GST tagged SAE2/SAE1 (E1 enzyme), 1.0 µg of Hs-Ubc9 (E2 enzyme), 4 µg GST-SUMO1, and 4 µg of GST-tagged standard peptide substrate were taken in a reaction buffer (50 mM Tris pH 7.5, 5 mM $MgCl_2$, 5 mM ATP and 5 mM dithiothreitol). *In vitro* SUMOylation reactions proceeded for 4 h at 37 °C and were terminated by adding 6X Laemmli buffer. Before analyzing the reaction contents on an SDS-PAGE gel to visualize the activity (Fig. S110), samples were heated for 10 minutes at 100°C.

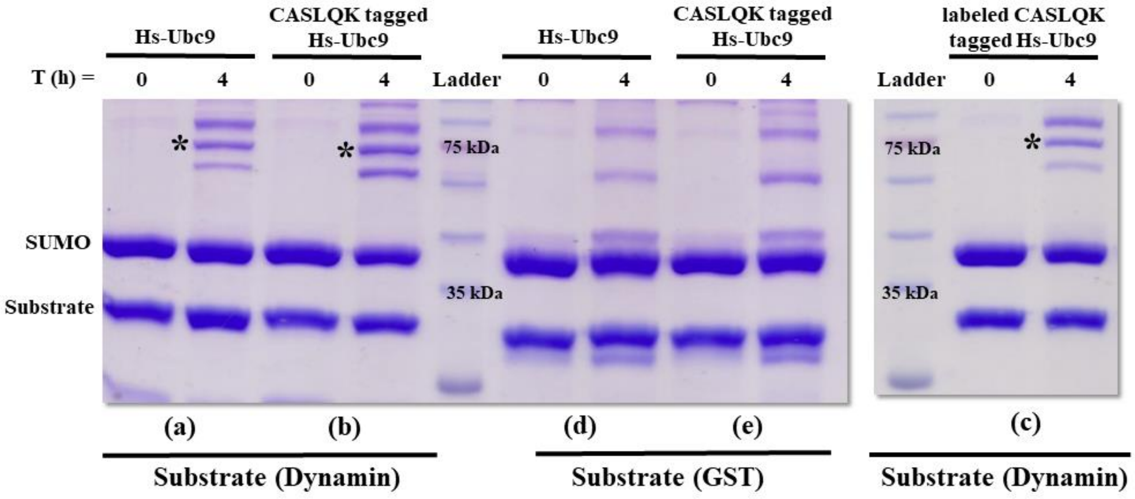

Supplementary Fig. 112. *In vitro* SUMOylation with recombinant Hs-Ubc9 and its bioconjugate. Sumoylation of GST-dynamin peptide substrate in the presence of all components of a SUMOylation reaction with (a) native Hs-Ubc9, (b) CASLQK tagged Hs-Ubc9**,** and (c) labeled CASLQK tagged Hs-Ubc9. Reactions in **(d)** and **(e)** are carried out in the presence of GST alone as substrate and serve as a negative control corresponding to **(a)** and **(b),** respectively. The asterisk denotes SUMO-modified substrate.

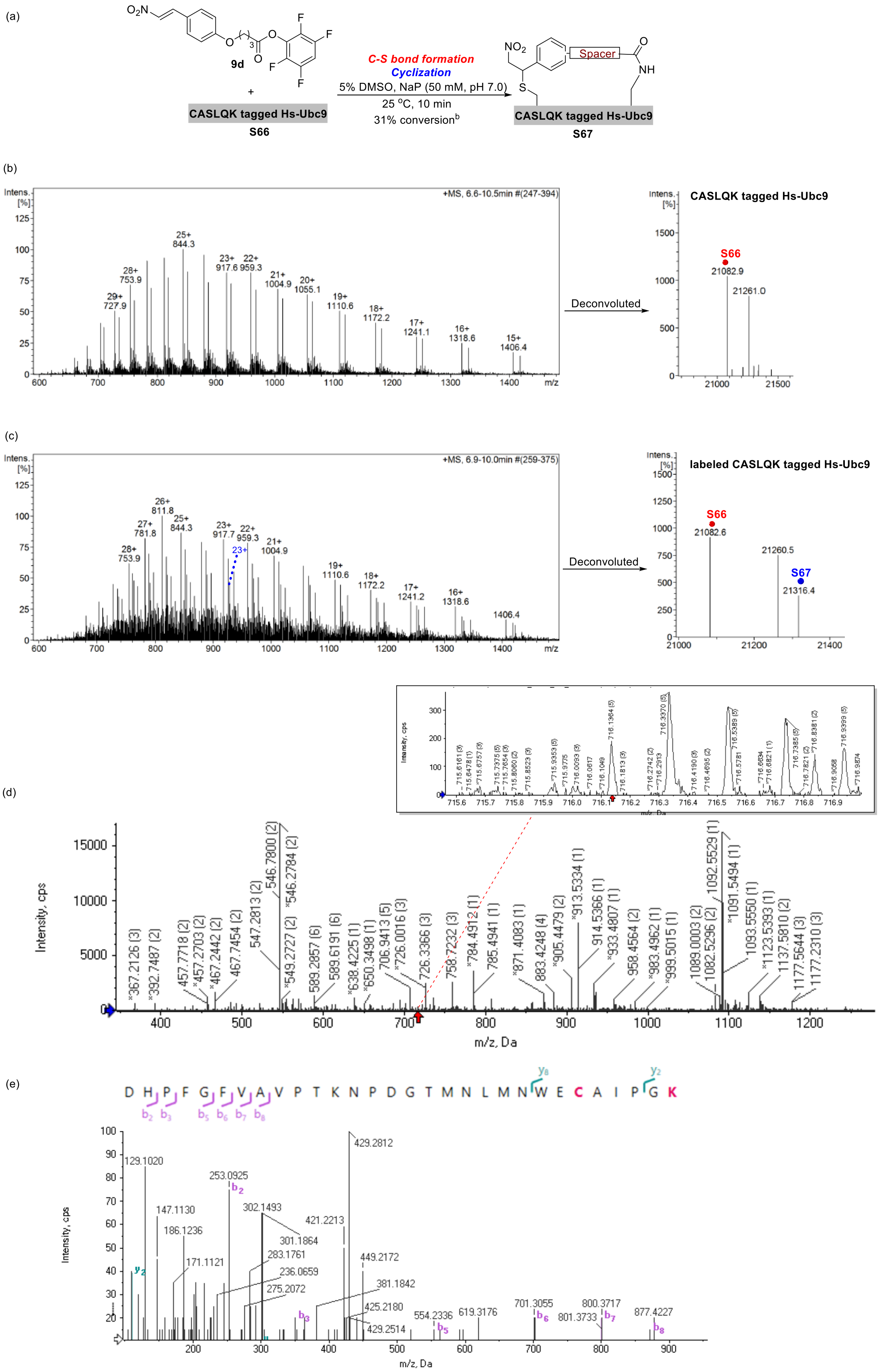

Supplementary Fig. 113. (a) Site-selective labeling of CASLQK tagged Hs-Ubc9 enabled by LDM_{CK} reagent **9d**. (b) ESI-MS data of CASLQK tagged Hs-Ubc9 **S66**. (c) ESI-MS data mono-labeled CASLQK tagged Hs-Ubc9 **S67**. (d) Peptide mapping of **S67** after the digestion with trypsin. (c) MS-MS spectrum of labelled DHPFGFVAVPTKNPDGTMNLMNWECAIPGK (D50-K79, *m/z* 716.3 $[M+5H]^{5+}$). The site of modification in mono-labeled CASLQK tagged Hs-Ubc9 **S67** is K79.

9. Protein sequence

PDB ID: 3BLG

[https://www.rcsb.org/structure/3BLG; PDB DOI: 10.2210/pdb3BLG/pdb]

LIVTQTMKGLDIQKVAGTWYSLAMAASDISLLDAQSAPLRVYVEELKPTPEGDLEIL
LQKWENDECAQKKIIAEKTKIPAVFKIDALNENKVLVLDTDYKKYLLFCMENSAEPE
QSLVCQCLVRTPEVDDEALEKFDKALKALPMHIRLSFNPTQLEQCHI

PDB ID: 1AO6 Human Serum Albumin

[https://www.rcsb.org/structure/1AO6; PDB DOI: 10.2210/pdb1AO6/pdb]

DAHKSEVAHRFKDLGEENFKALVLIAFAQYLQQCPFEDHVKLVNEVTEFAKTCVAD
ESAENCDKSLHTLFGDKLCTVATLRETYGEMADCCAKQEPERNECFLQHKDDNPNL
PRLVRPEVDVMCTAFHDNEETFLKKYLYEIARRHPYFYAPELLFFAKRYKAAFTECC
QAADKAACLLPKLDELRDEGKASSAKQRLKCASLQKFGERAFKAWAVARLSQRFP
KAEFAEVSKLVTDLTKVHTECCHGDLLECADDRADLAKYICENQDSISSKLKECCEK
PLLEKSHCIAEVENDEMPADLPSLAADFVESKDVCKNYAEAKDVFLGMFLYEYARR
HPDYSVVLLLRLAKTYETTLEKCCAAADPHECYAKVFDEFKPLVEEPQNLIKQNCEL
FEQLGEYKFQNALLVRYTKKVPQVSTPTLVEVSRNLGKVGSKCCKHPEAKRMPCAE
DYLSVVLNQLCVLHEKTPVSDRVTKCCTESLVNRRPCFSALEVDETYVPKEFNAETF
TFHADICTLSEKERQIKKQTALVELVKHKPKATKEQLKAVMDDFAAFVEKCCKADD
KETCFAEEGKKLVAASQAALGL

PDB ID: 3V03 Bovine Serum Albumin

[https://www.rcsb.org/structure/3V03; PDB DOI: 10.2210/pdb3V03/pdb]

DTHKSEIAHRFKDLGEEHFKGLVLIAFSQYLQQCPFDEHVKLVNELTEFAKTCVADE
SHAGCEKSLHTLFGDELCKVASLRETYGDMADCCEKQEPERNECFLSHKDDSPDLP
KLKPDPNTLCDEFKADEKKFWGKYLYEIARRHPYFYAPELLYYANKYNGVFQECCQ
AEDKGACLLPKIETMREKVLTSSARQRLRCASIQKFGERALKAWSVARLSQKFPKAE
FVEVTKLVTDLTKVHKECCHGDLLECADDRADLAKYICDNQDTISSKLKECCDKPLL
EKSHCIAEVEKDAIPENLPPLTADFAEDKDVCKNYQEAKDAFLGSFLYEYSRRHPEY
AVSVLLRLAKEYEATLEECCAKDDPHACYSTVFDKLKHLVDEPQNLIKQNCDQFEK
LGEYGFQNALIVRYTRKVPQVSTPTLVEVSRSLGKVGTRCCTKPESERMPCTEDYLS
LILNRLCVLHEKTPVSEKVTKCCTESLVNRRPCFSALTPDETYVPKAFDEKLFTFHAD
ICTLPDTEKQIKKQTALVELLKHKPKATEEQLKTVMENFVAFVDKCCAADDKEACF
AVEGPKLVVSTQTALA

native Hs-Ubc9

MGSSHHHHHHSSGLVPRGSHMASMTGGQQMGRGSMSGIALSRLAQERKAWRKDH
PFGFVAVPTKNPDGTMNLMNWECAIPGKKGTPWEGGLFKLRMLFKDDYPSSPPKCK
FEPPLFHPNVYPSGTVCLSILEEDKDWRPAITIKQILLGIQELLNEPNIQDPAQAEAYTI
YCQNRVEYEKRVRAQAKKFAPS

CASLQK tagged Hs-Ubc9

MGSSHHHHHHSSGLVPRGSHMASCASLQKGSMSGIALSRLAQERKAWRKDHPFGF
VAVPTKNPDGTMNLMNWECAIPGKKGTPWEGGLFKLRMLFKDDYPSSPPKCKFEPP

LFHPNVYPSGTVCLSILEEDKDWRPAITIKQILLGIQELLNEPNIQDPAQAEAYTIYCQ
NRVEYEKRVRAQAKKFAPS

2. Trastuzumab (recombinant humanized monoclonal antibody)
Light chain amino acid sequence (1-214)
DIQMTQSPSSLSASVGDRVTITCRASQDVNTAVAWYQQKPGKAPKLLIYSASFLYSGVP
SRFSGSRSGTDFTLTISSLQPEDFATYYCQQHYTTPPTFGQGTKVEIKRTVAAPSVFIFPPS
DEQLKSGTASVVCLLNNFYPREAKVQWKVDNALQSGNSQESVTEQDSKDSTYSLSSTL
TLSKADYEKHKVYACEVTHQGLSSPVTKSFNRGEC

Heavy chain amino acid sequence (1-449)
EVQLVESGGGLVQPGGSLRLSCAASGFNIKDTYIHWVRQAPGKGLEWVARIYPTNGYT
RYADSVKGRFTISADTSKNTAYLQMNSLRAEDTAVYYCSRWGGDGFYAMDYWGQGT
LVTVSSASTKGPSVFPLAPSSKSTSGGTAALGCLVKDYFPEPVTVSWNSGALTSGVHTFP
AVLQSSGLYSLSSVVTVPSSSLGTQTYICNVNHKPSNTKVDKKVEPKSCDKTHTCPPCPA
PELLGGPSVFLFPPKPKDTLMISRTPEVTCVVVDVSHEDPEVKFNWYVDGVEVHNAKTK
PREEQYNSTYRVVSVLTVLHQDWLNGKEYKCKVSNKALPAPIEKTISKAKGQPREPQV
YTLPPSREEMTKNQVSLTCLVKGFYPSDIAVEWESNGQPENNYKTTPPVLDSDGSFFLY
SKLTVDKSRWQQGNVFSCSVMHEALHNHYTQKSLSLSPG

10. Spectral data

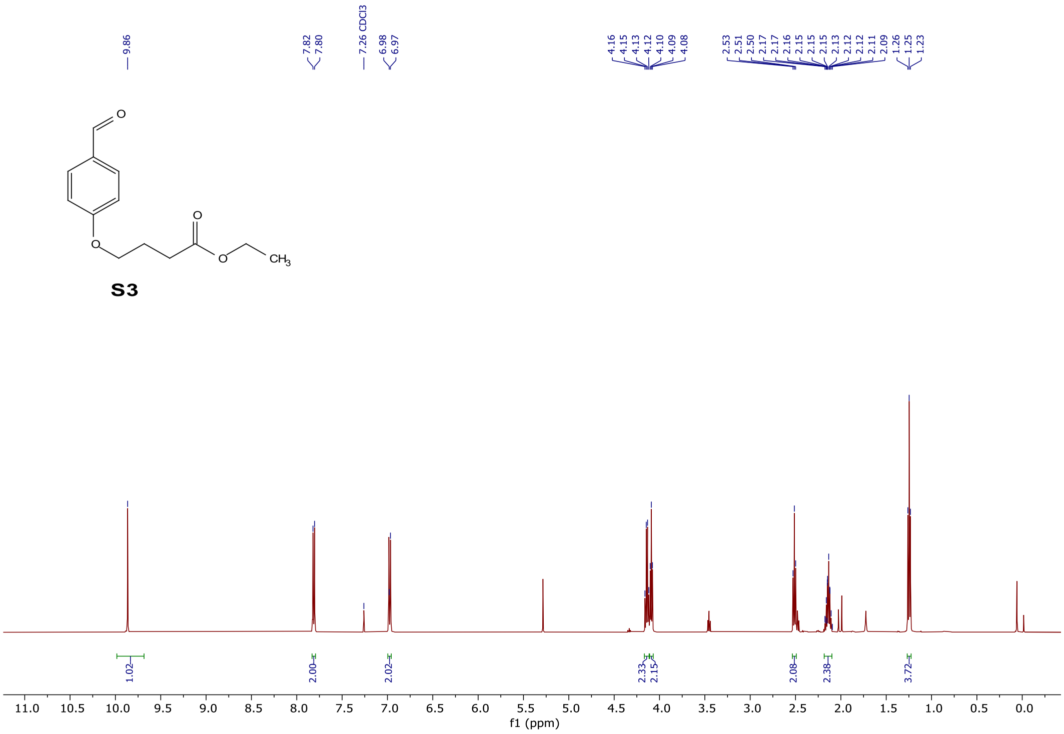

Supplementary Fig. 114. ^{1}H NMR spectrum in $CDCl_3$ of compound **S3**.

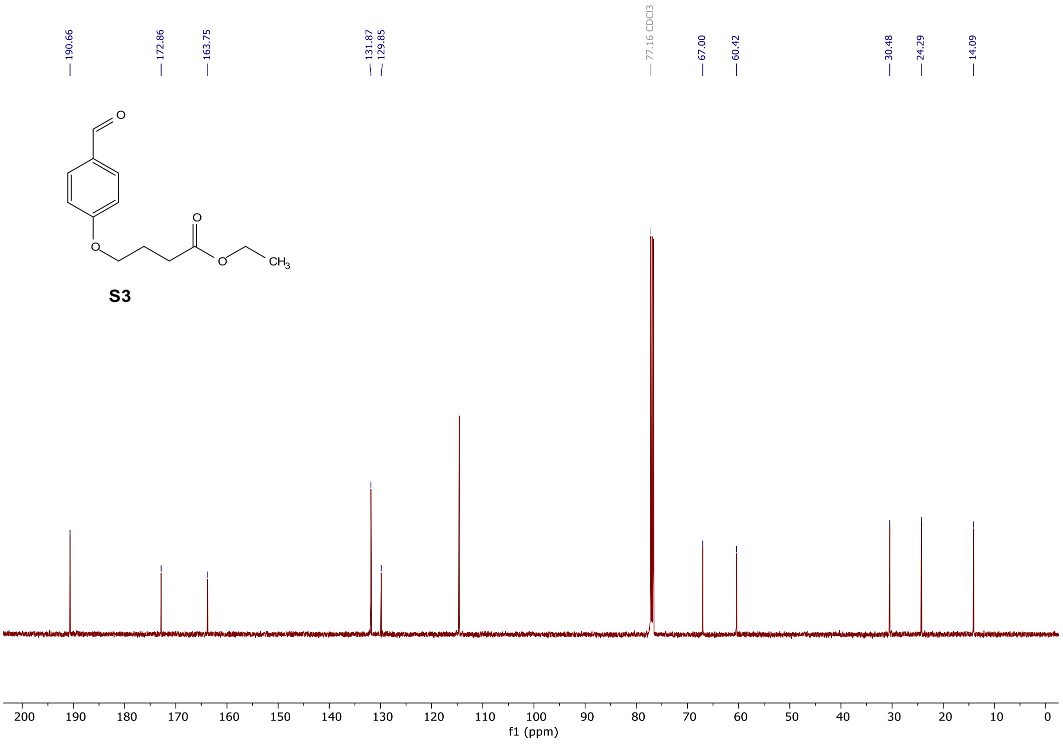

Supplementary Fig. 115. ^{13}C NMR spectrum in $CDCl_3$ of compound **S3**.

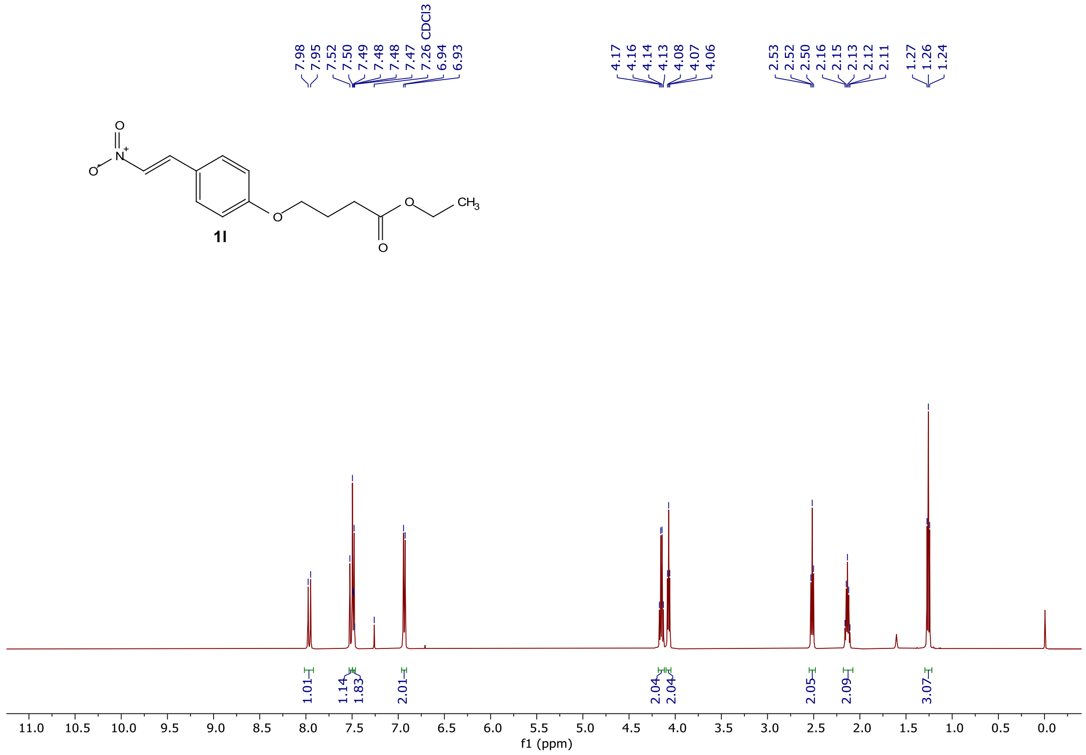

Supplementary Fig. 116. ^{1}H NMR spectrum in $CDCl_3$ of compound **1l**.

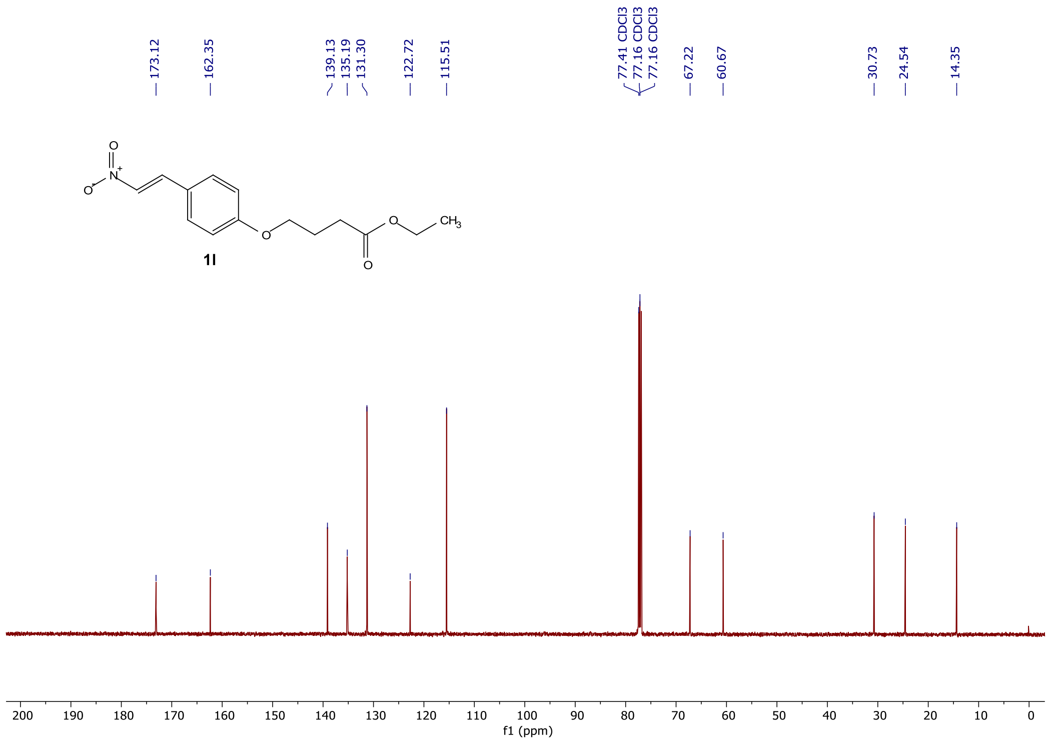

Supplementary Fig. 117. ^{13}C NMR spectrum in $CDCl_3$ of compound **1l**.

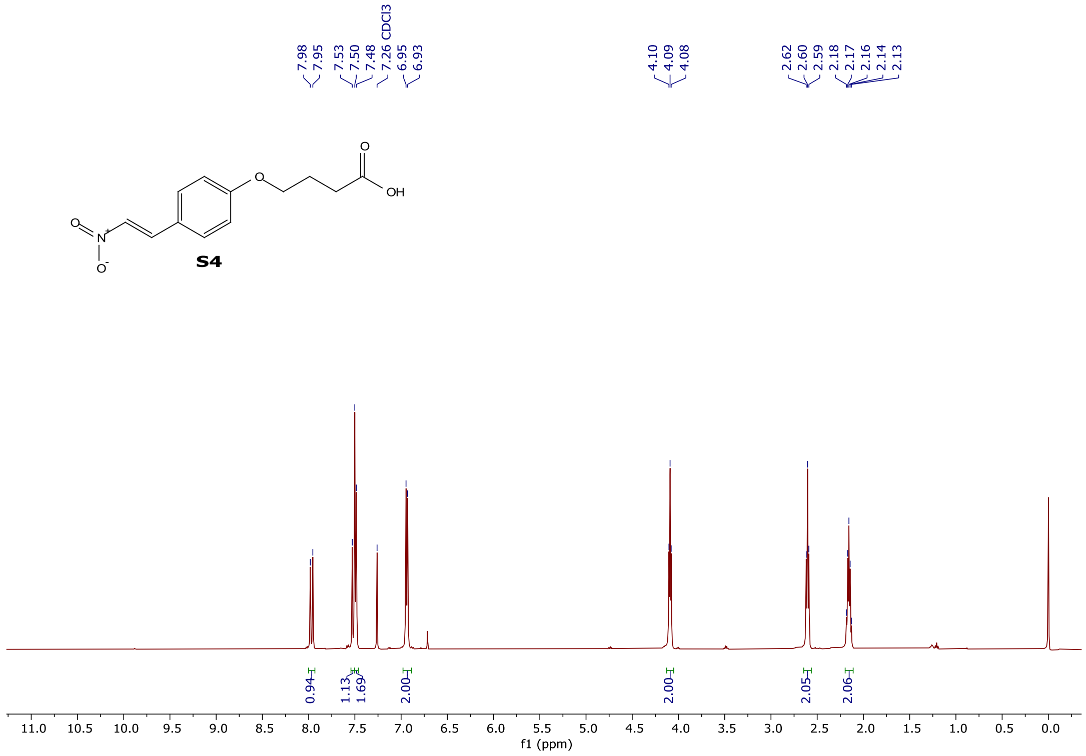

Supplementary Fig. 118. ^{1}H NMR spectrum in $CDCl_3$ of compound **S4**.

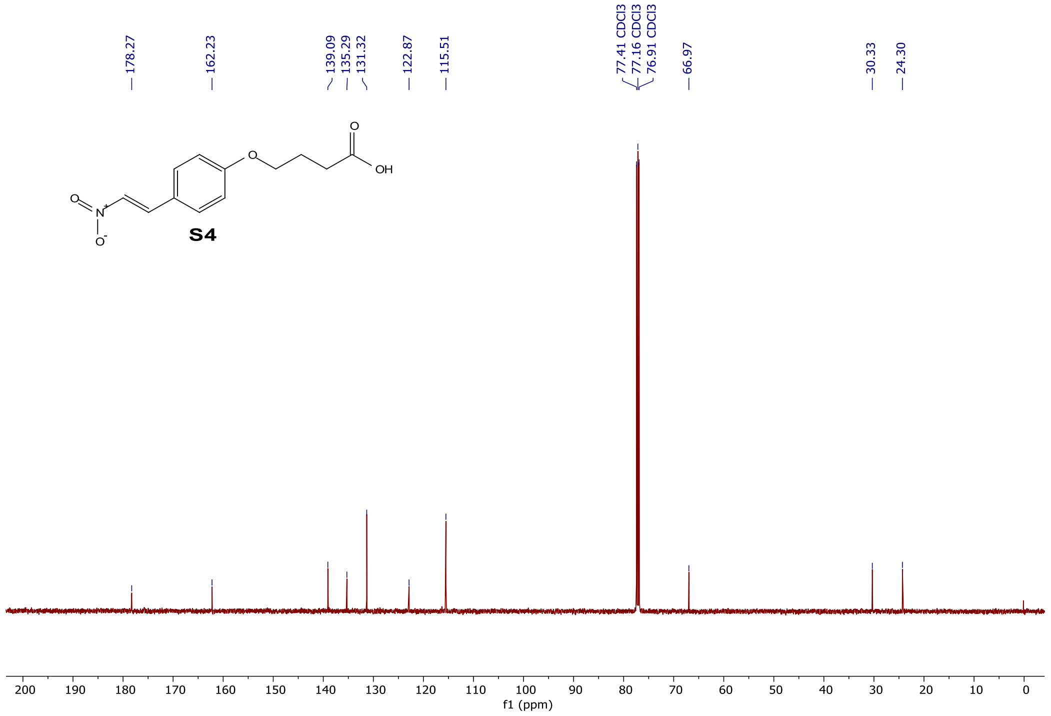

Supplementary Fig. 119. ^{13}C NMR spectrum in $CDCl_3$ of compound **S4**.

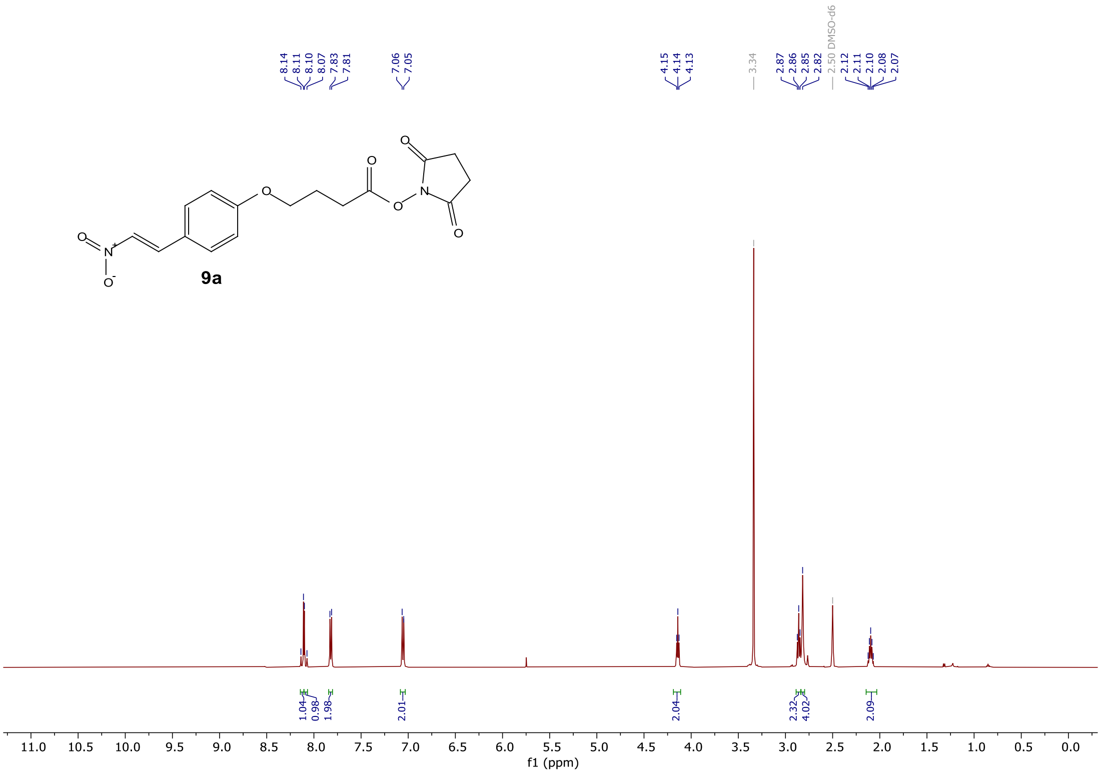

Supplementary Fig. 120. ^{1}H NMR spectrum in DMSO-d_6 of compound **9a**.

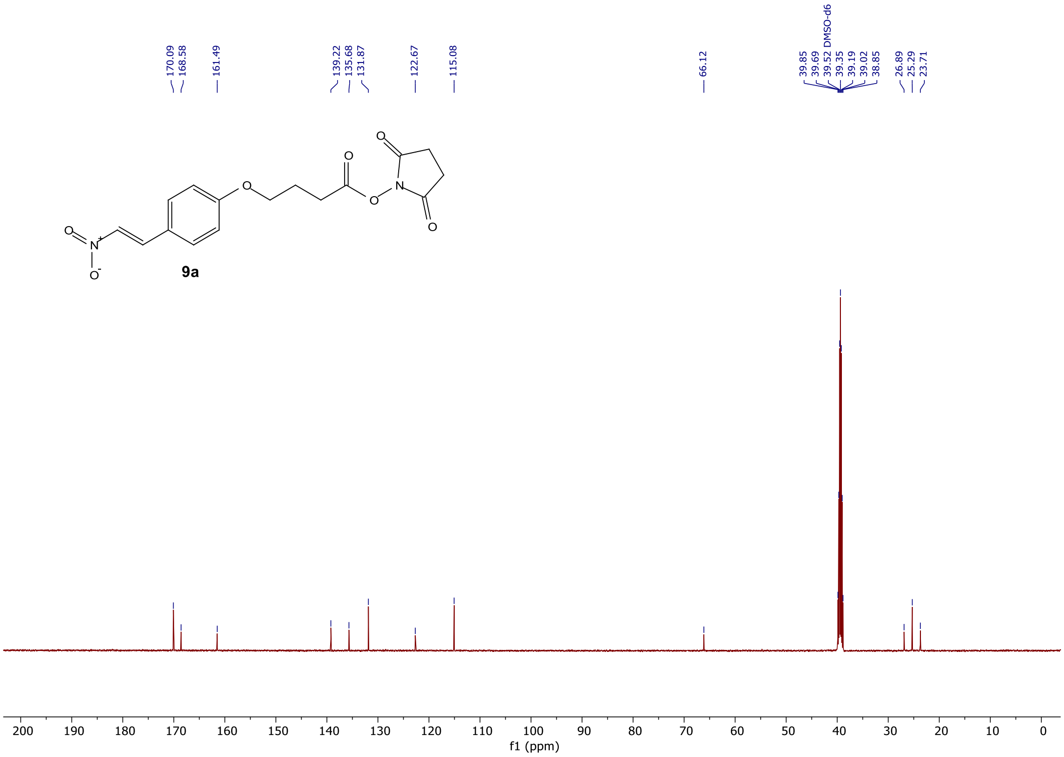

Supplementary Fig. 121. ^{13}C NMR spectrum in DMSO-d_6 of compound **9a**.

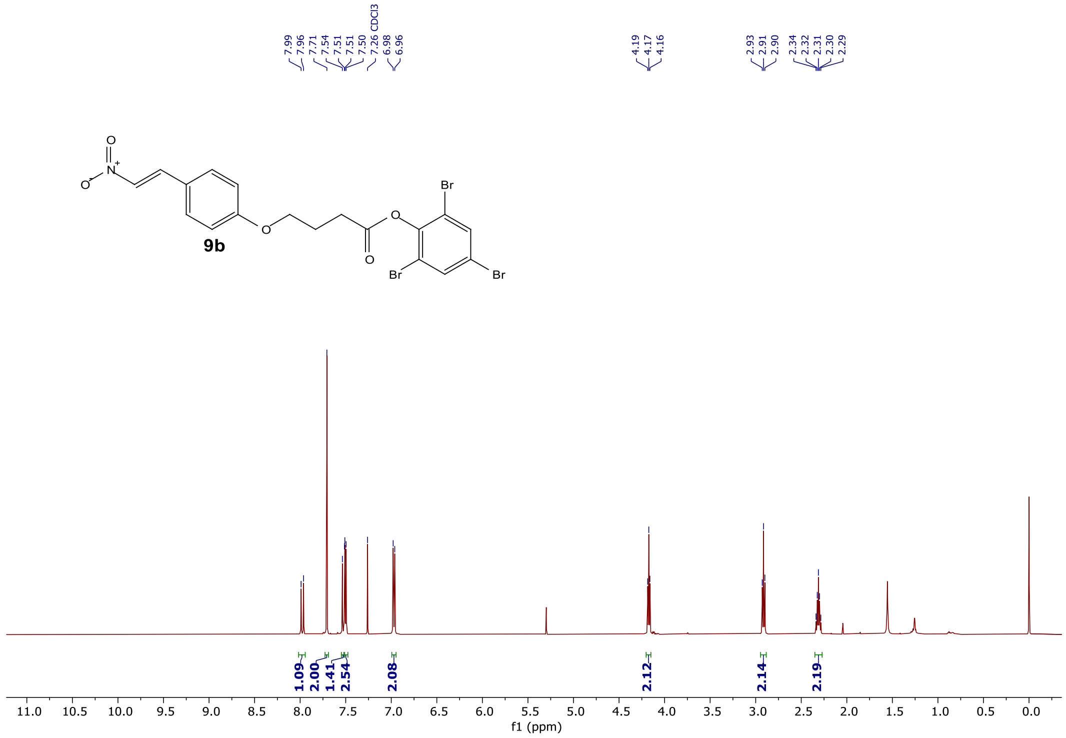

Supplementary Fig. 122. ^{1}H NMR spectrum in $CDCl_3$ of compound **9b**.

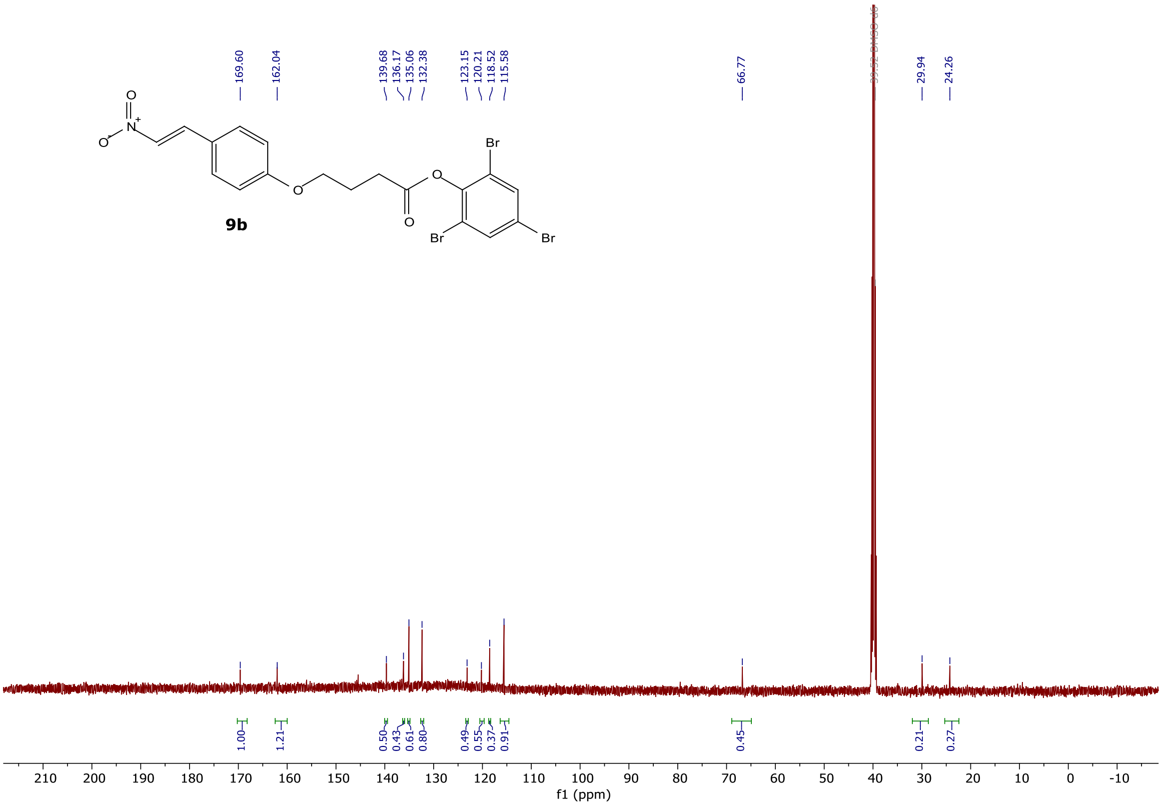

Supplementary Fig. 123. ^{13}C NMR spectrum in DMSO-d_6 of compound **9b**.

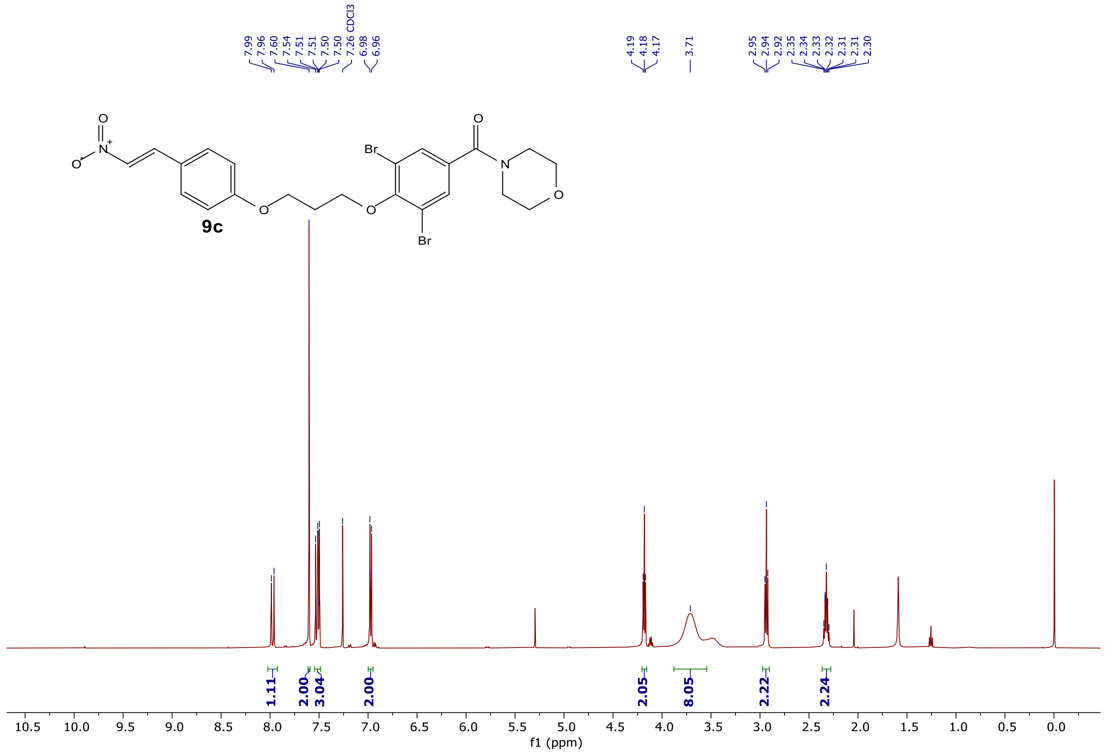

Supplementary Fig. 124. ^{1}H NMR spectrum in $CDCl_3$ of compound **9c**.

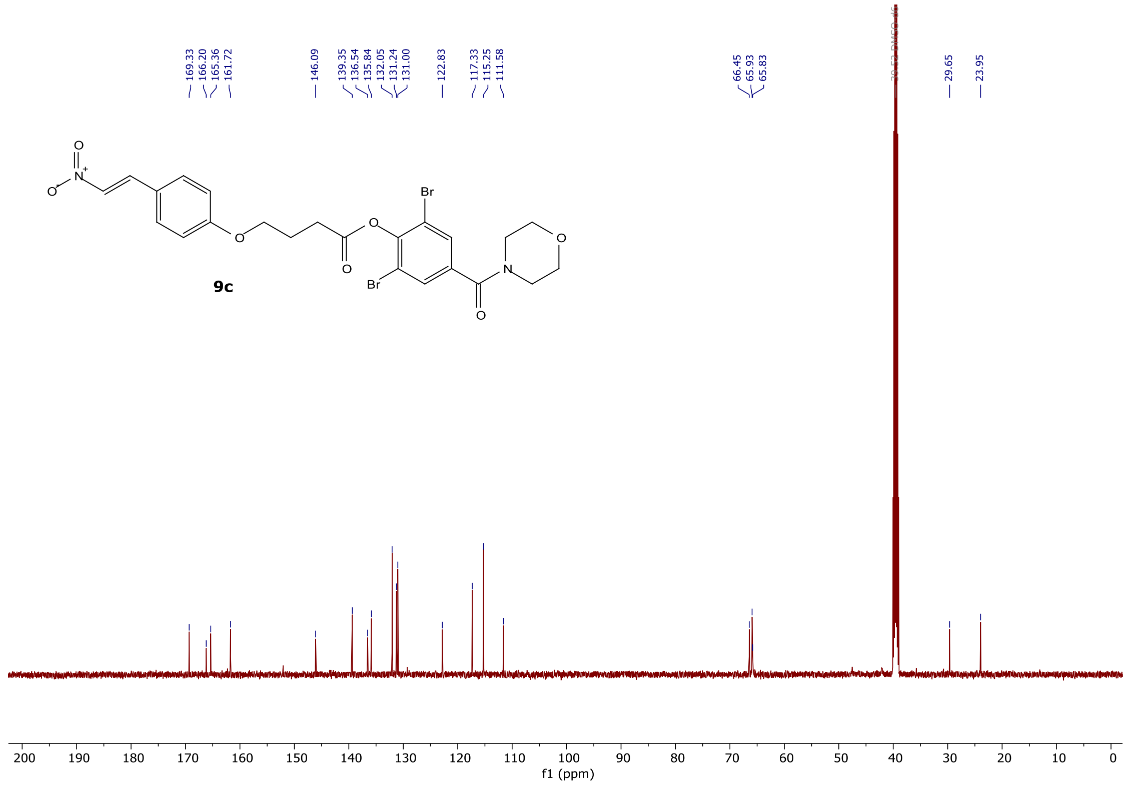

Supplementary Fig. 125. ^{13}C NMR spectrum in DMSO-d_6 of compound **9c**.

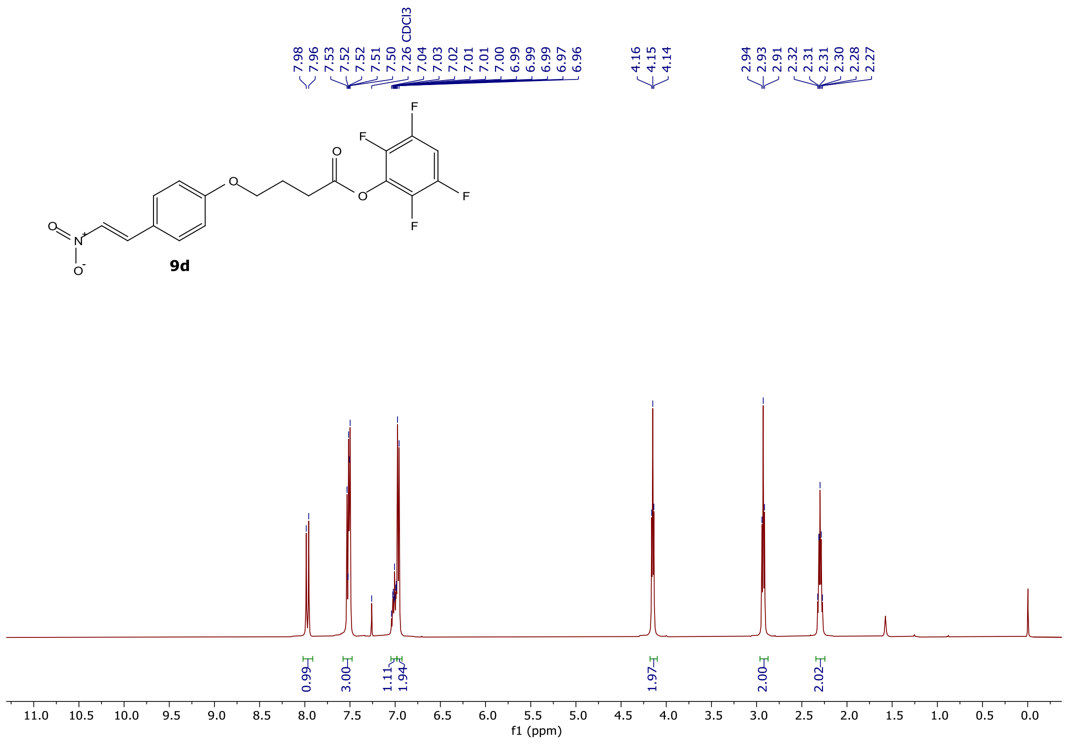

Supplementary Fig. 126. ^{1}H NMR spectrum in $CDCl_3$ of compound **9d**.

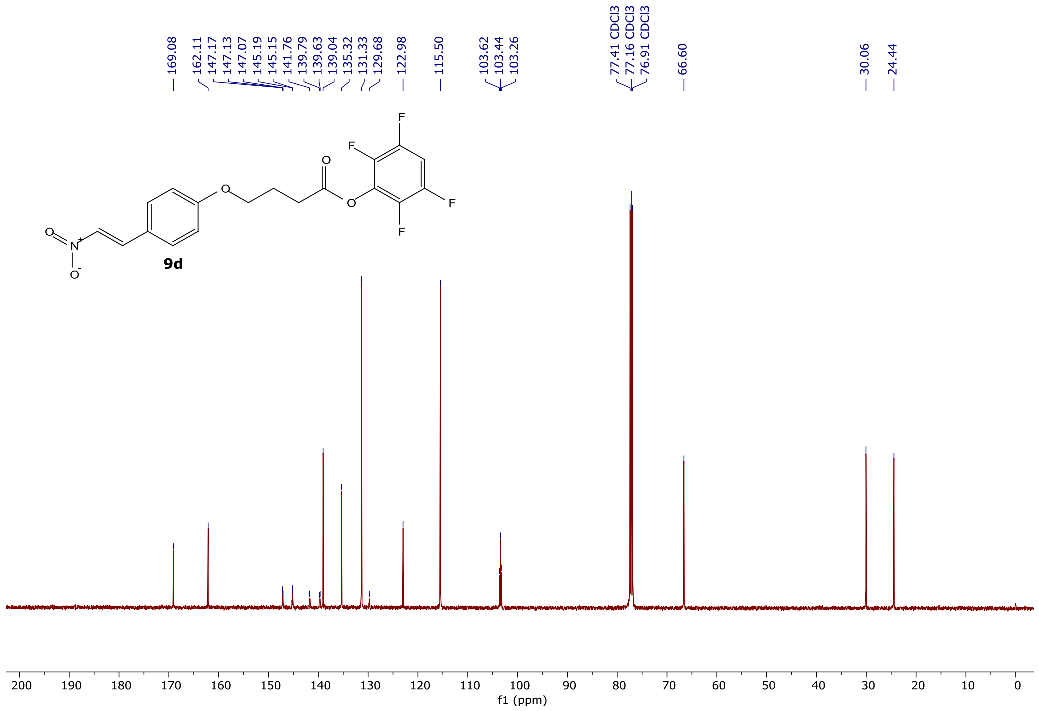

Supplementary Fig. 127. ^{13}C NMR spectrum in $CDCl_3$ of compound **9d**.

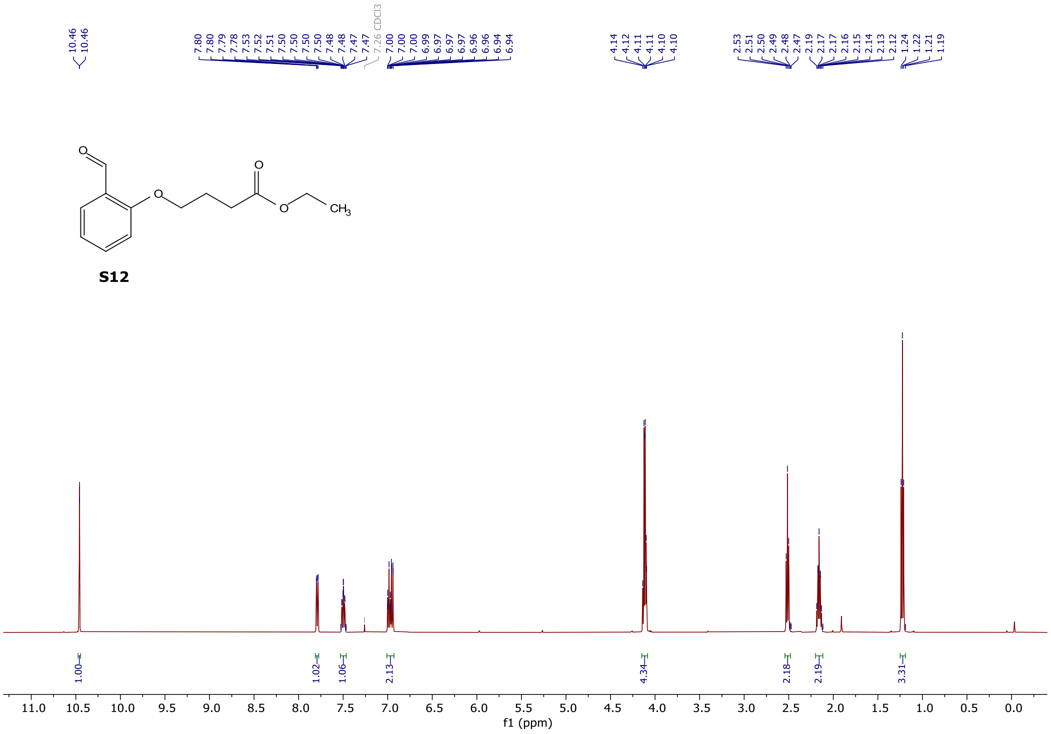

Supplementary Fig. 128. ^{1}H NMR spectrum in $CDCl_3$ of compound **S12**.

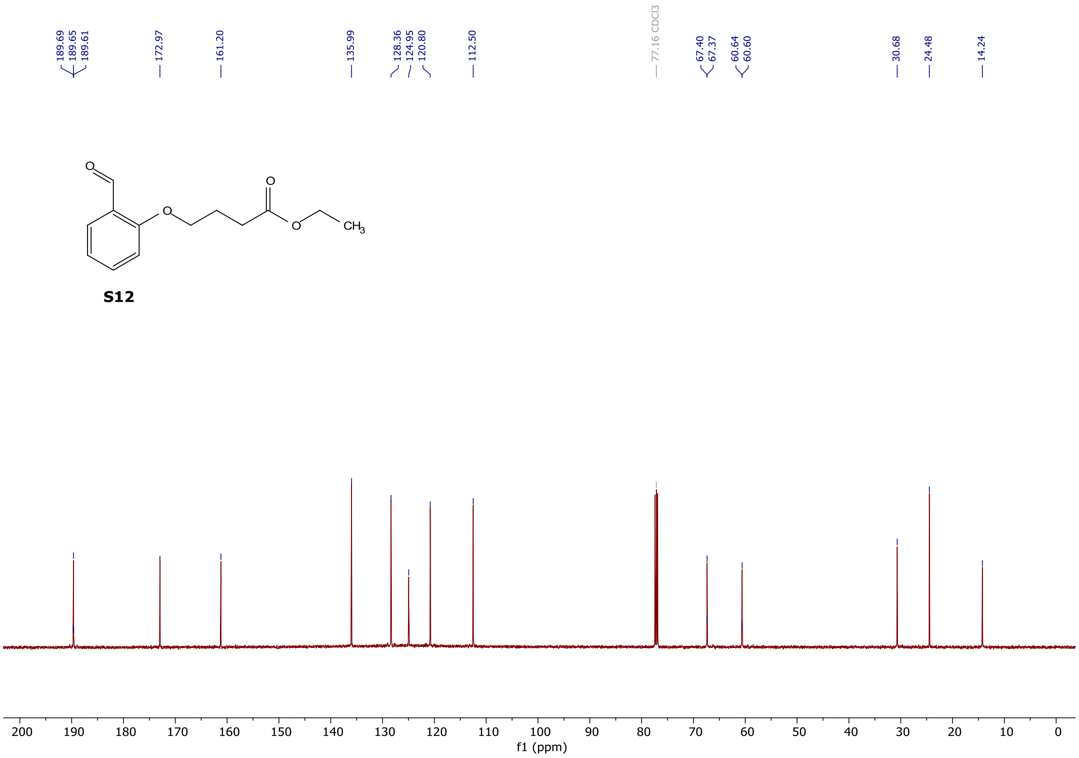

Supplementary Fig. 129. ^{13}C NMR spectrum in $CDCl_3$ of compound **S12**.

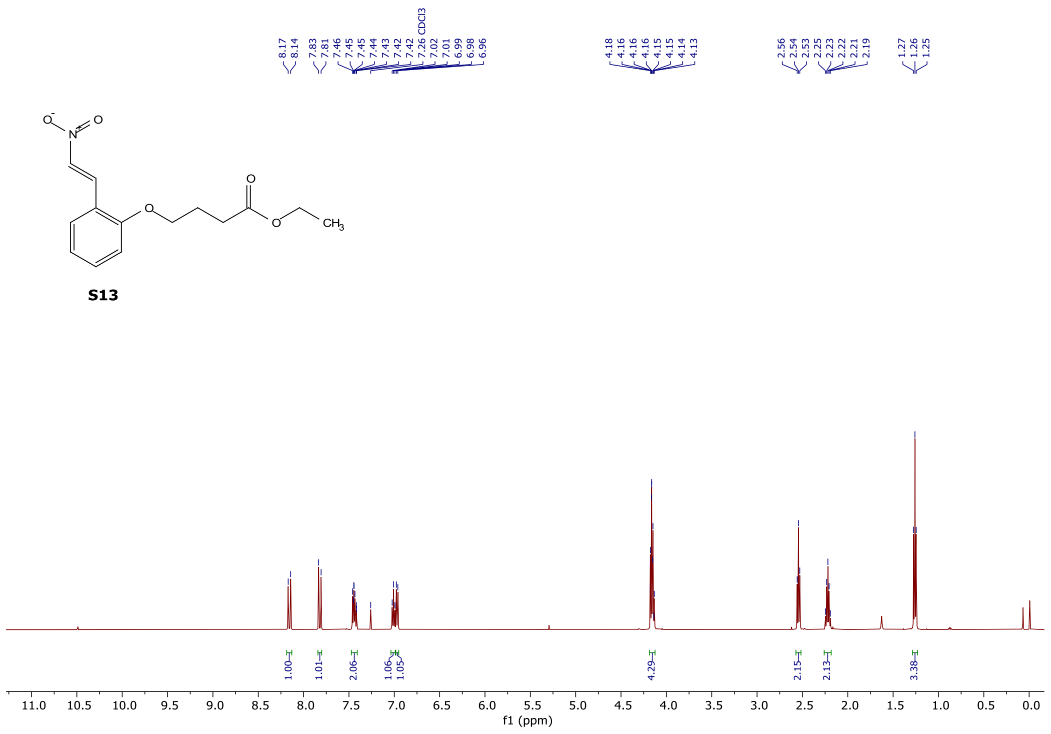

Supplementary Fig. 130. ^{1}H NMR spectrum in $CDCl_3$ of compound **S13**.

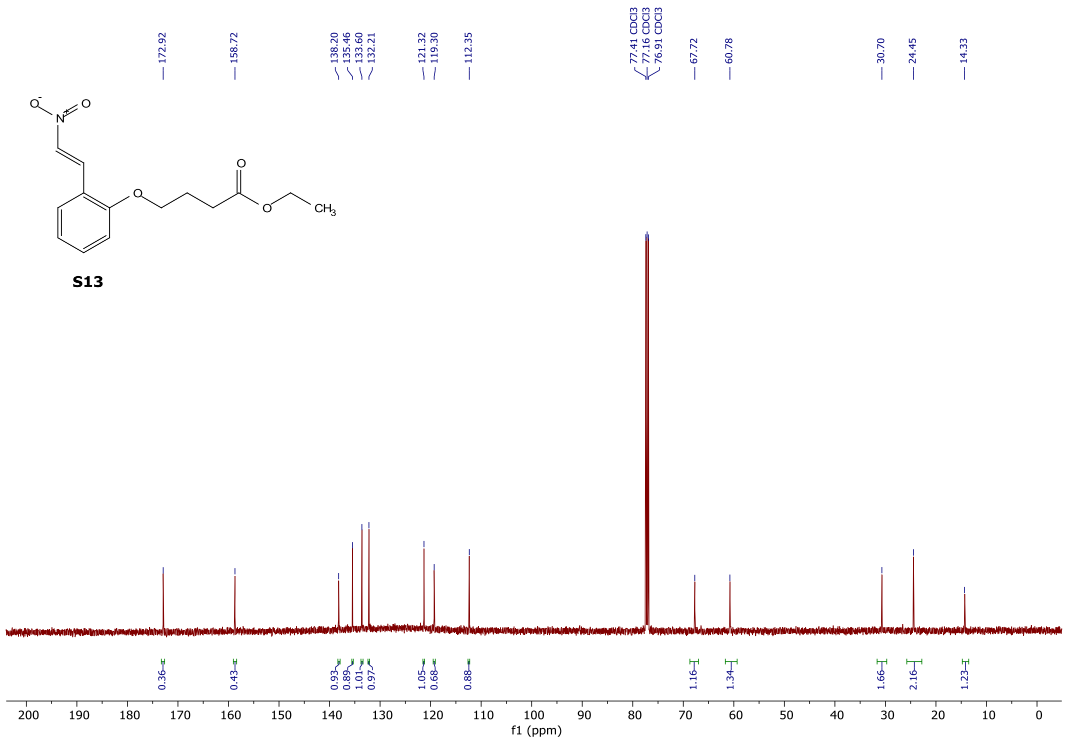

Supplementary Fig. 131. ^{13}C NMR spectrum in $CDCl_3$ of compound **S13**.

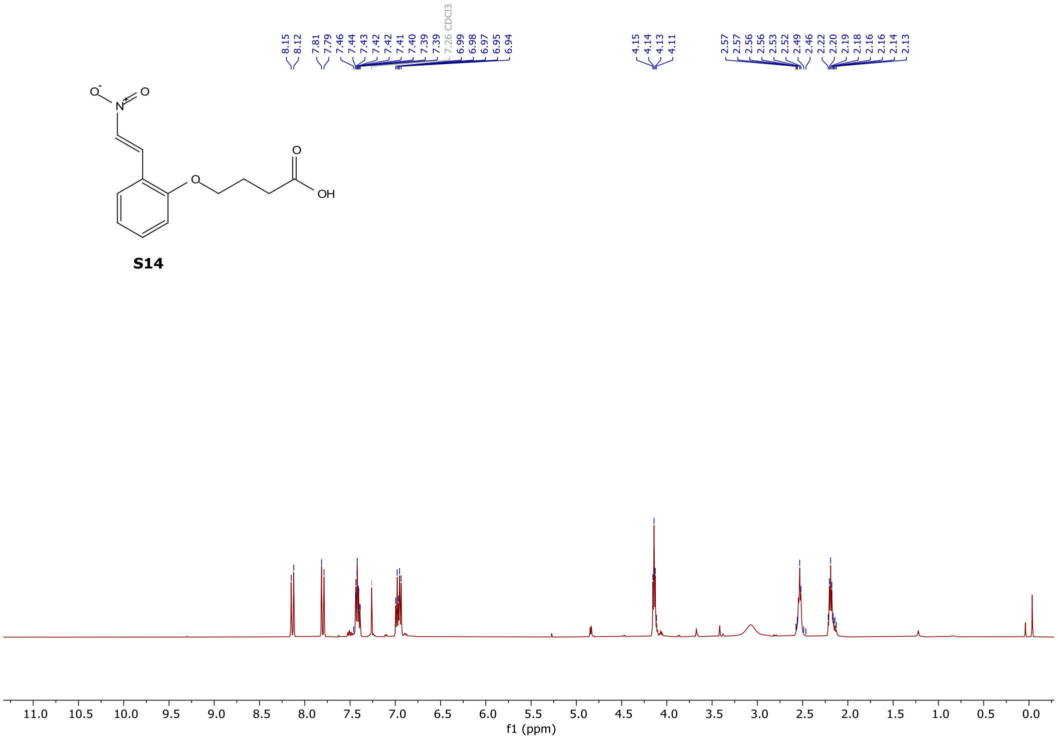

Supplementary Fig. 132. ^{1}H NMR spectrum in $CDCl_3$ of compound **S14**.

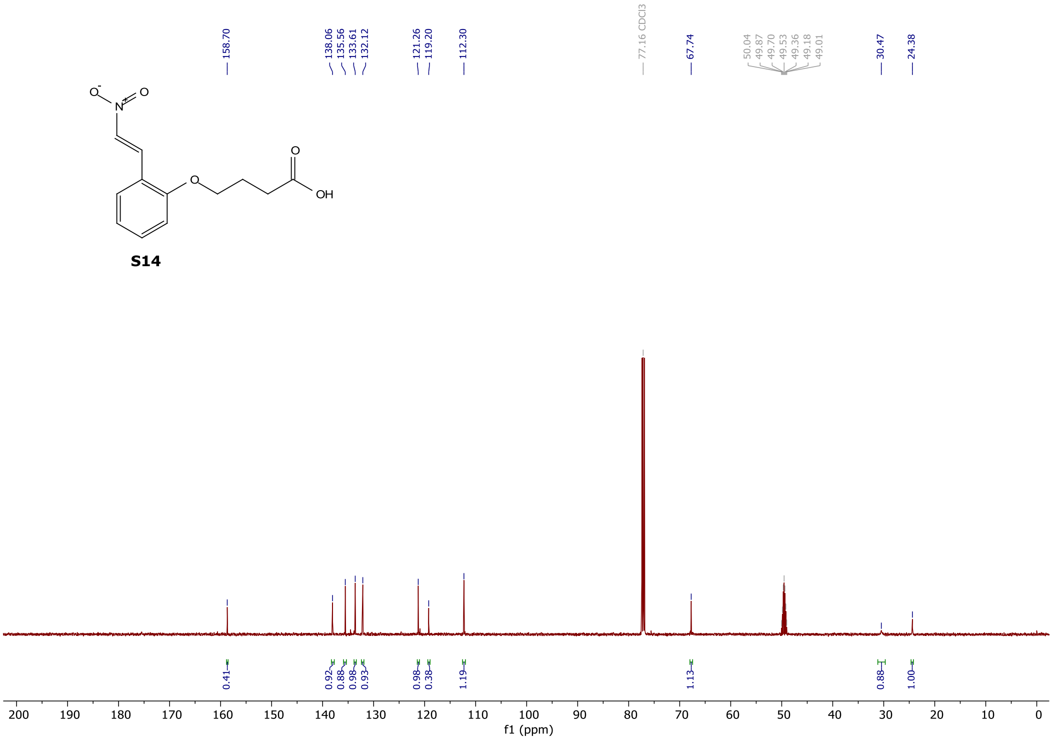

Supplementary Fig. 133. ^{13}C NMR spectrum in $CDCl_3$ of compound **S14**.

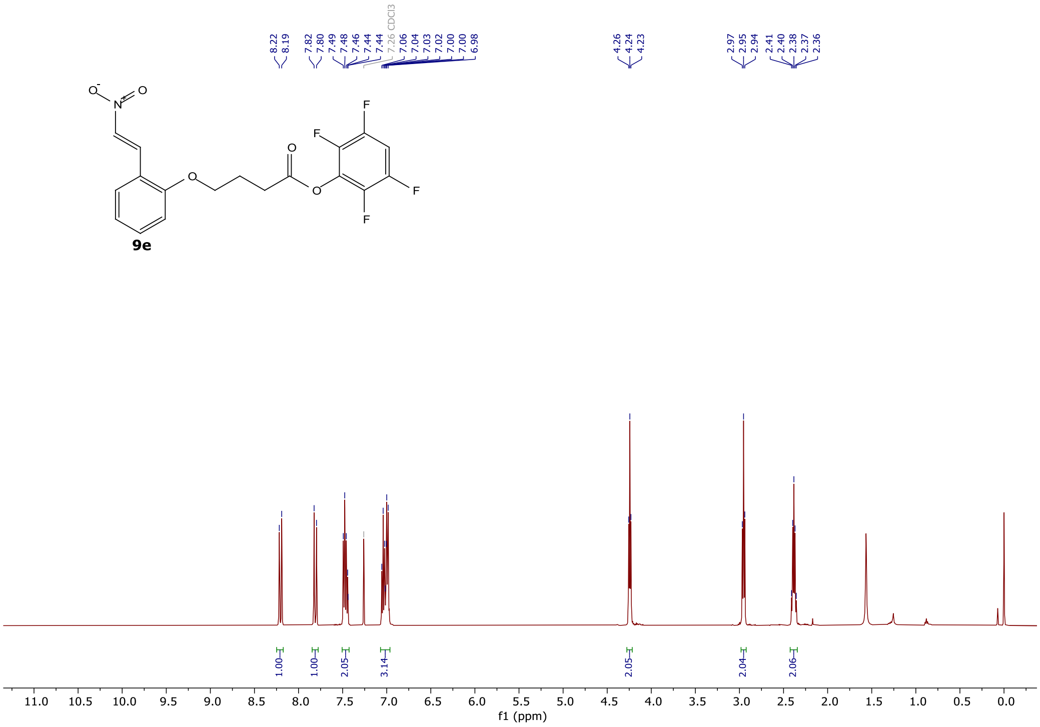

Supplementary Fig. 134. ^{1}H NMR spectrum in $CDCl_3$ of compound **9e**.

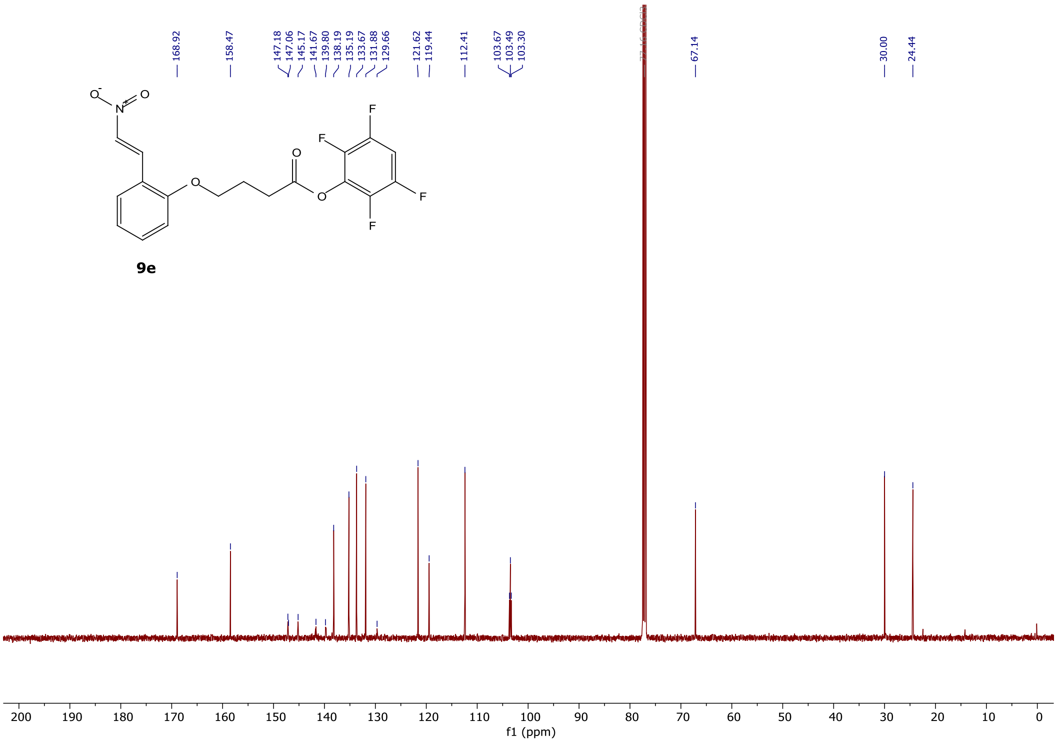

Supplementary Fig. 135. ^{13}C NMR spectrum in $CDCl_3$ of compound **9e**.

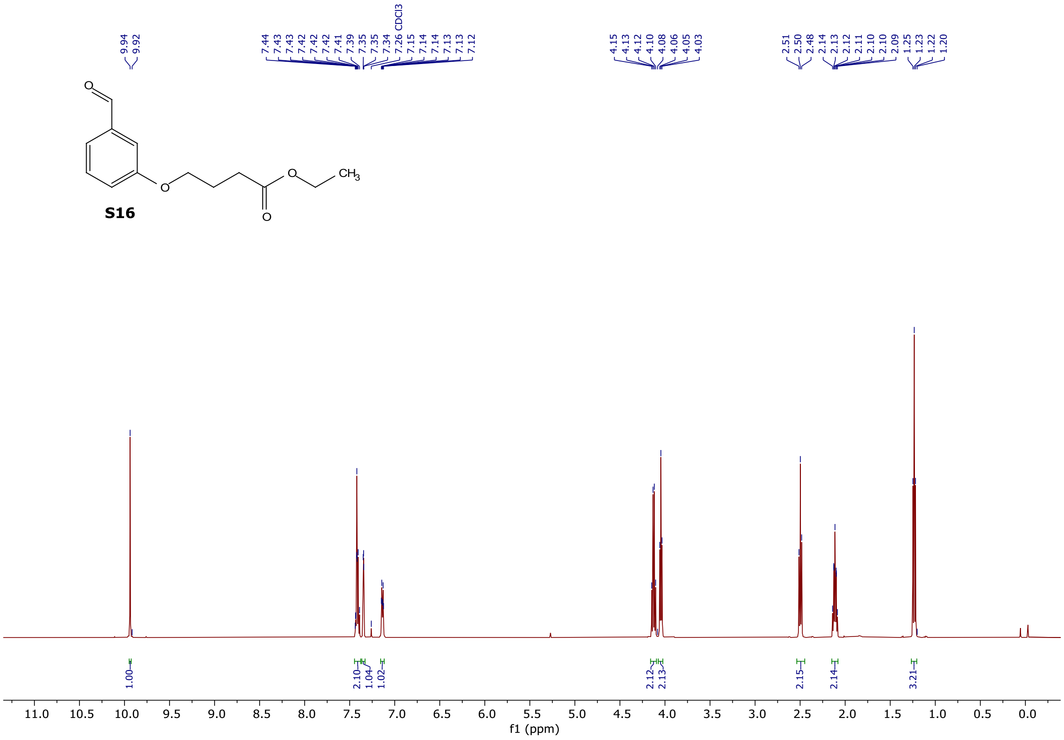

Supplementary Fig. 136. ^{1}H NMR spectrum in $CDCl_3$ of compound **S16**.

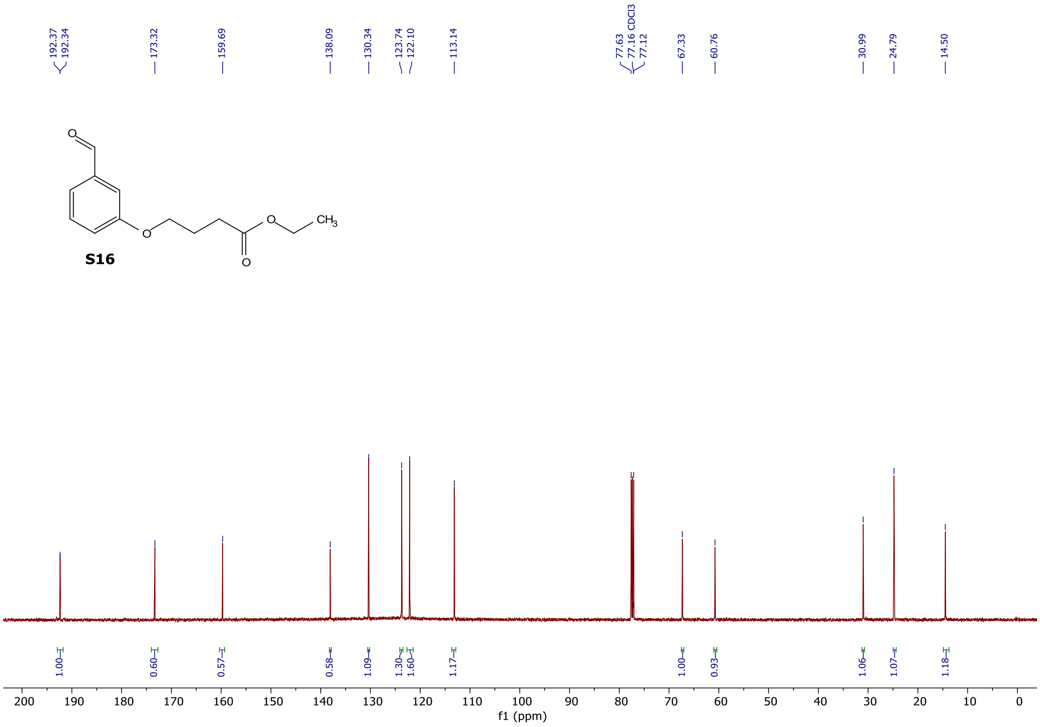

Supplementary Fig. 137. ^{13}C NMR spectrum in $CDCl_3$ of compound **S16**.

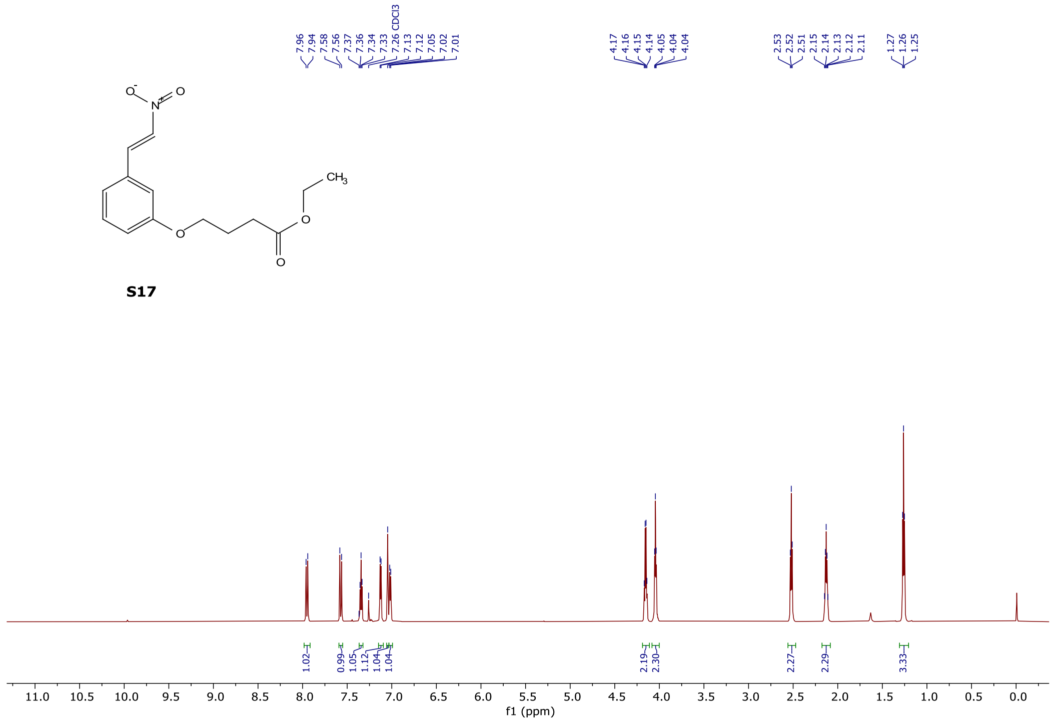

Supplementary Fig. 138. ^{1}H NMR spectrum in $CDCl_3$ of compound **S17**.

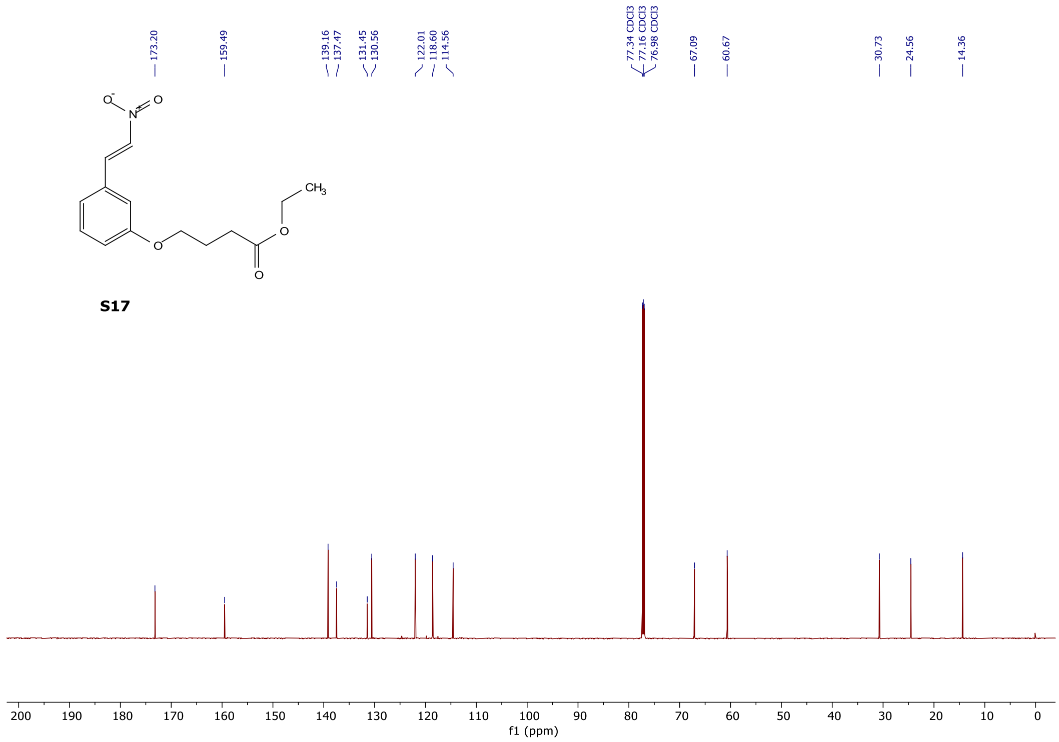

Supplementary Fig. 139. ^{13}C NMR spectrum in $CDCl_3$ of compound **S17**.

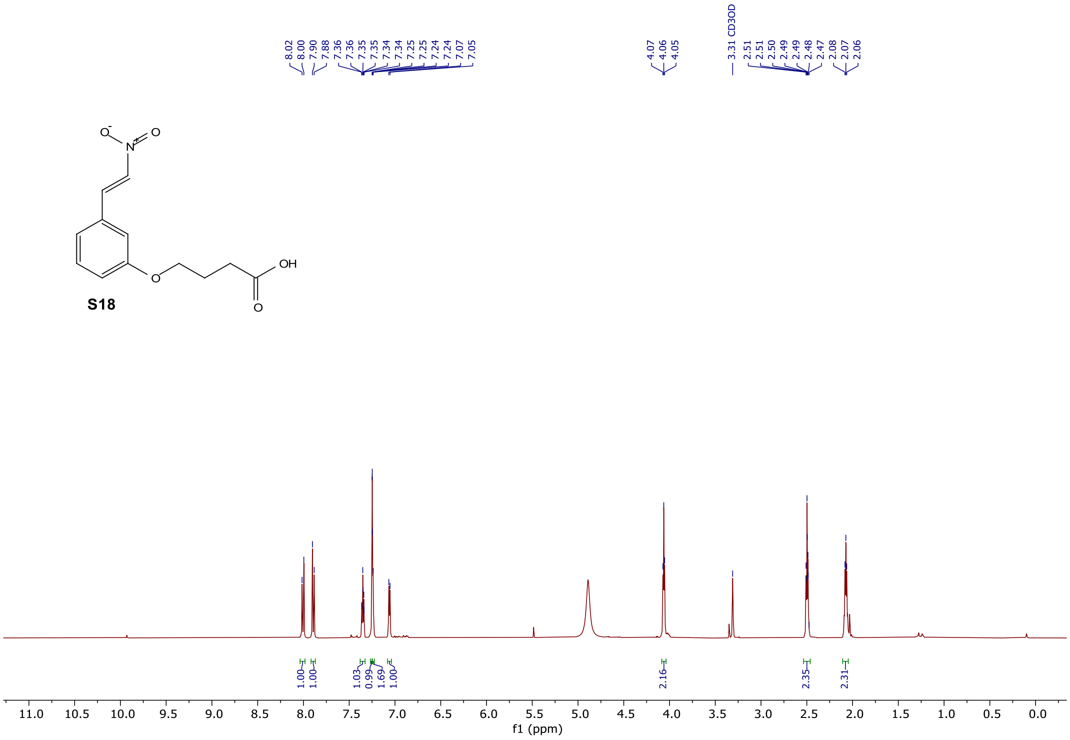

Supplementary Fig. 140. ^{1}H NMR spectrum in $CDCl_3$ of compound **S18**.

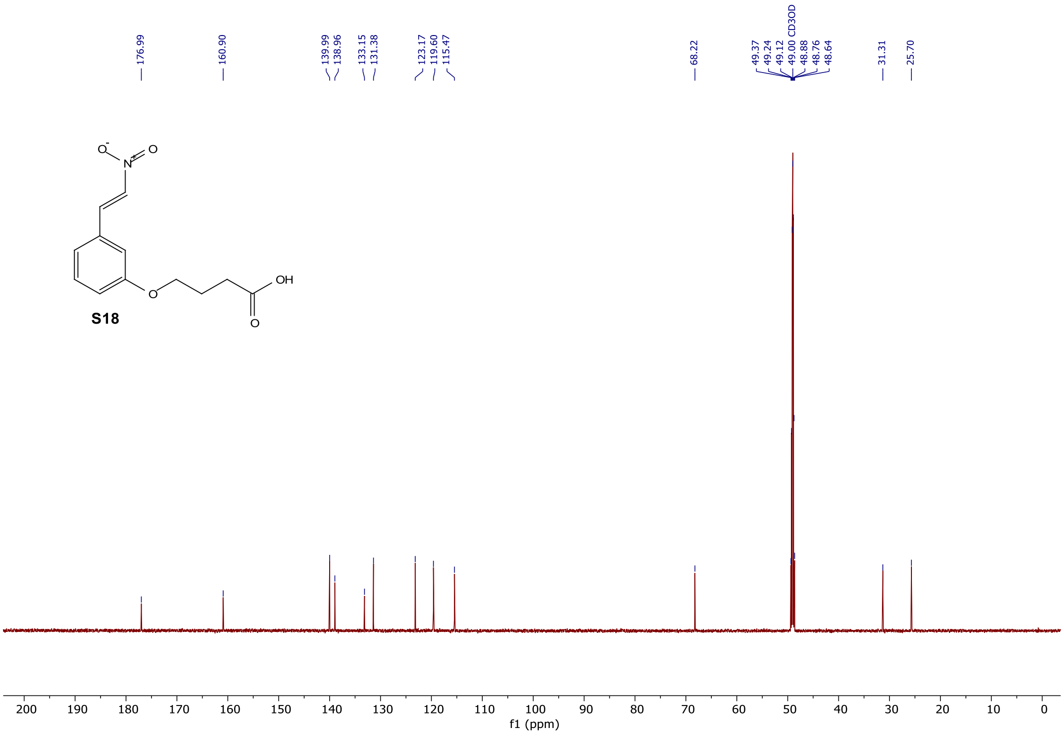

Supplementary Fig. 141. ^{13}C NMR spectrum in $CDCl_3$ of compound **S18**.

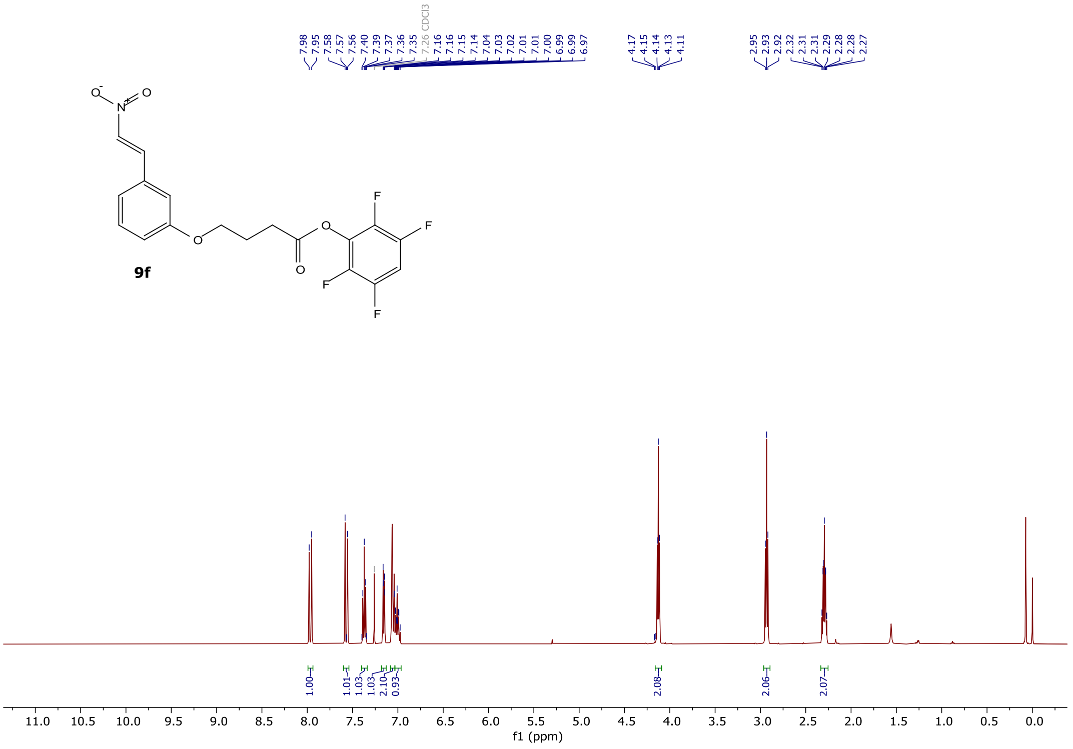

Supplementary Fig. 142. ^{1}H NMR spectrum in $CDCl_3$ of compound **9f**.

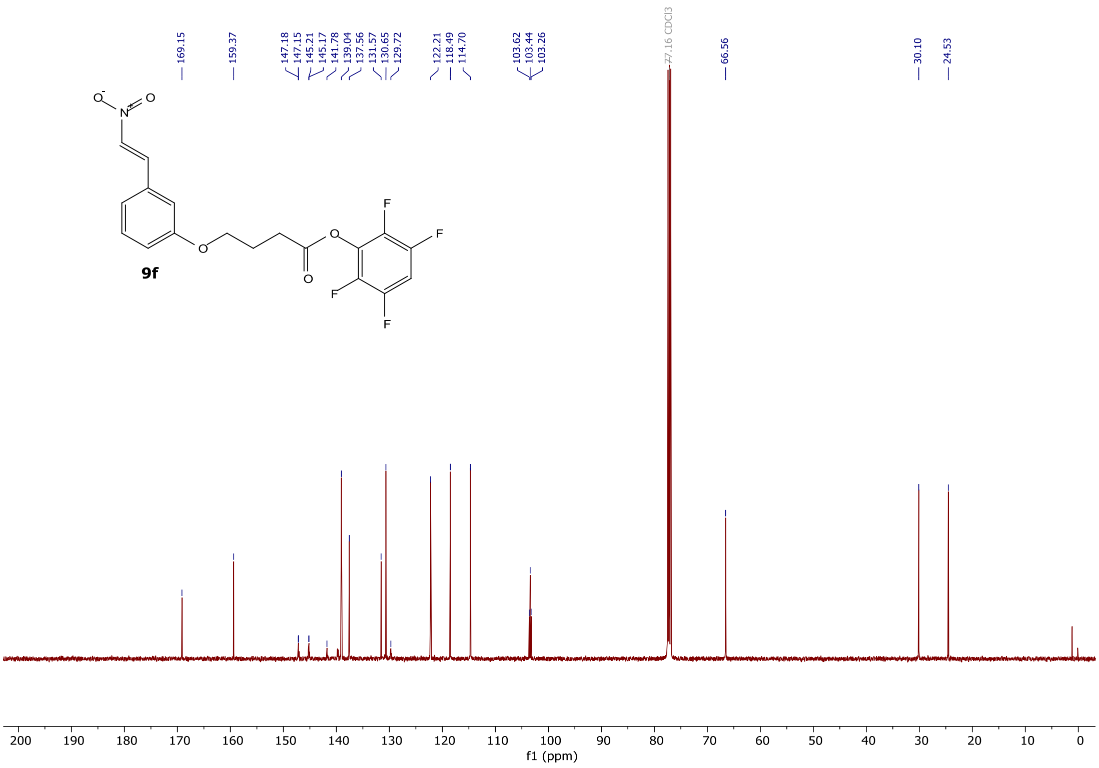

Supplementary Fig. 143. ^{13}C NMR spectrum in $CDCl_3$ of compound **9f**.

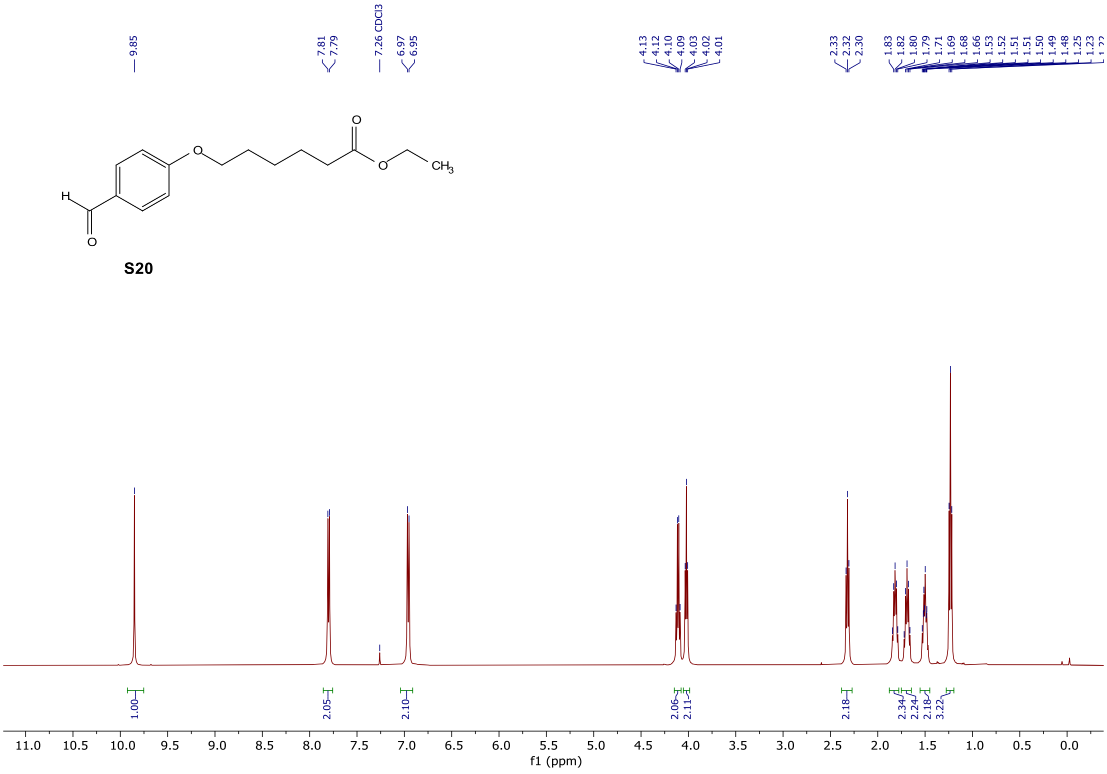

Supplementary Fig. 144. ^{1}H NMR spectrum in $CDCl_3$ of compound **S20**.

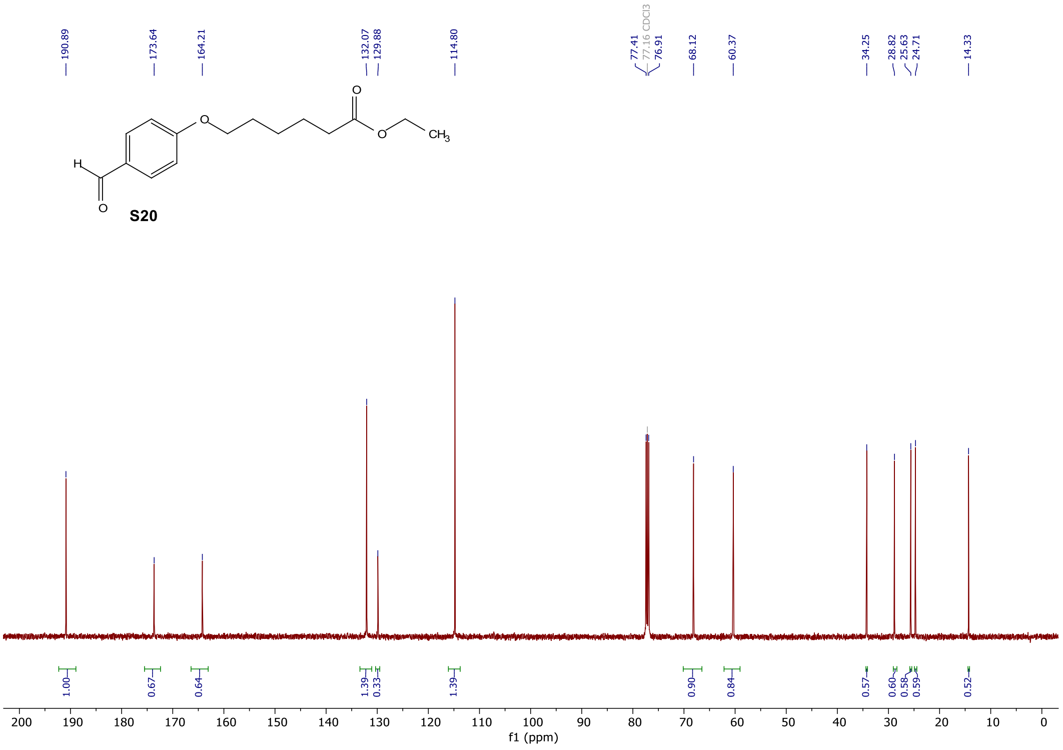

Supplementary Fig. 145. ^{13}C NMR spectrum in $CDCl_3$ of compound **S20**.

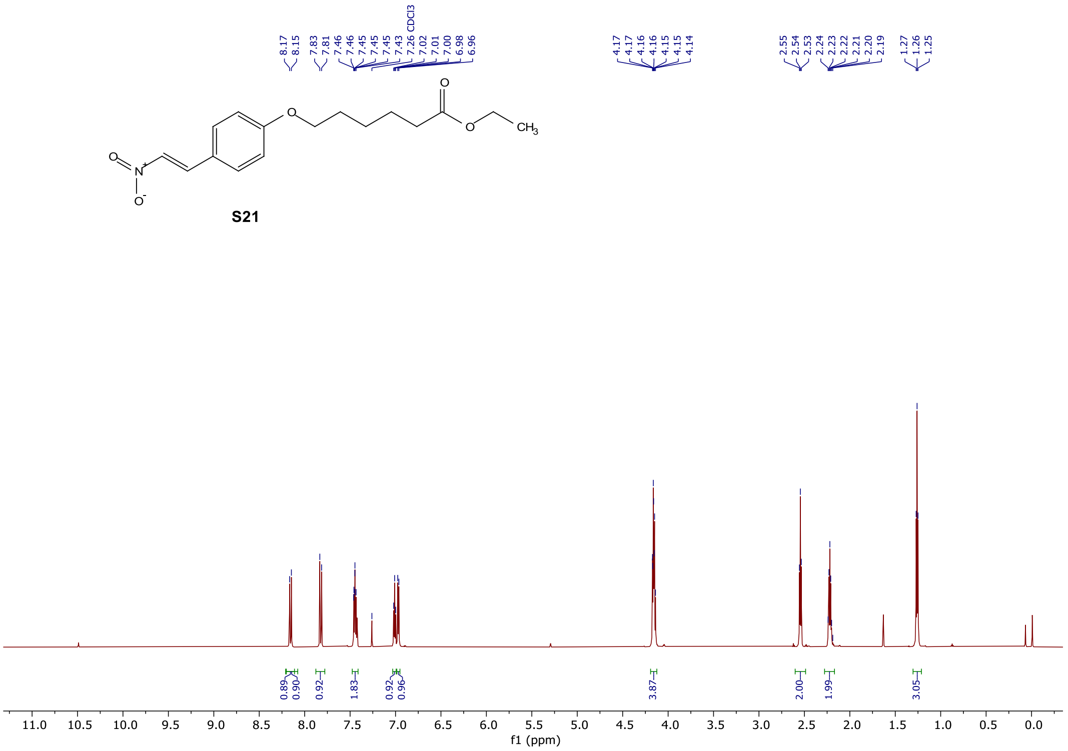

Supplementary Fig. 146. ^{1}H NMR spectrum in $CDCl_3$ of compound **S21**.

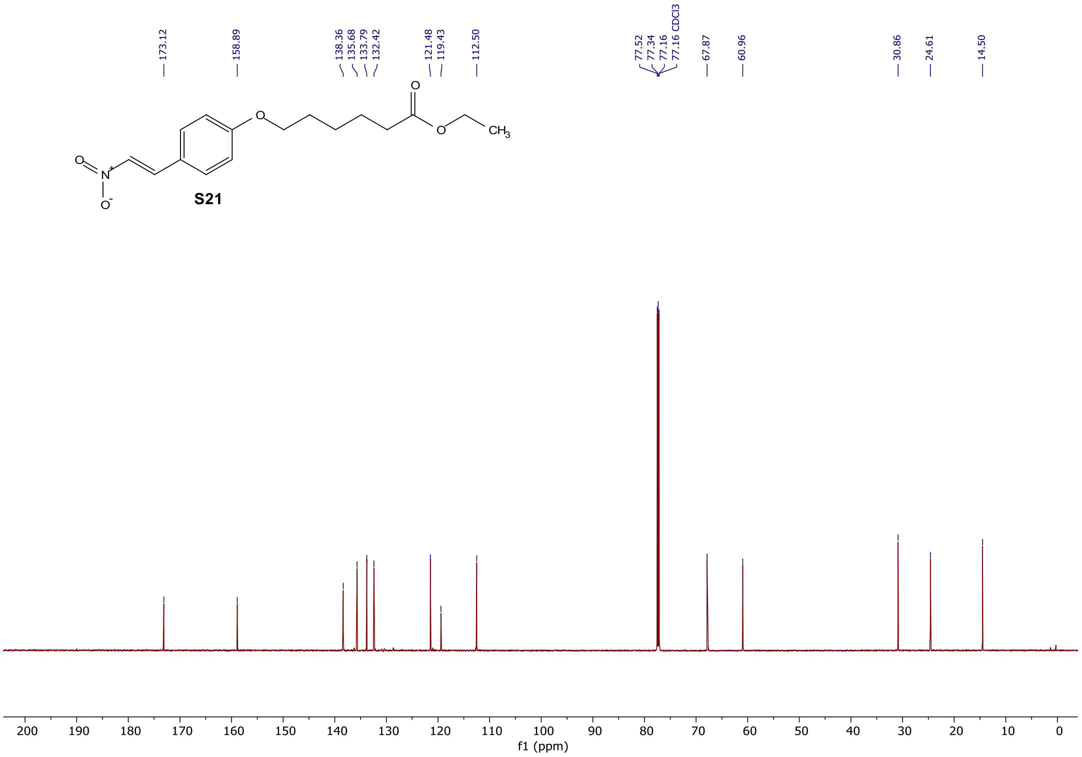

Supplementary Fig. 147. ^{13}C NMR spectrum in $CDCl_3$ of compound **S21**.

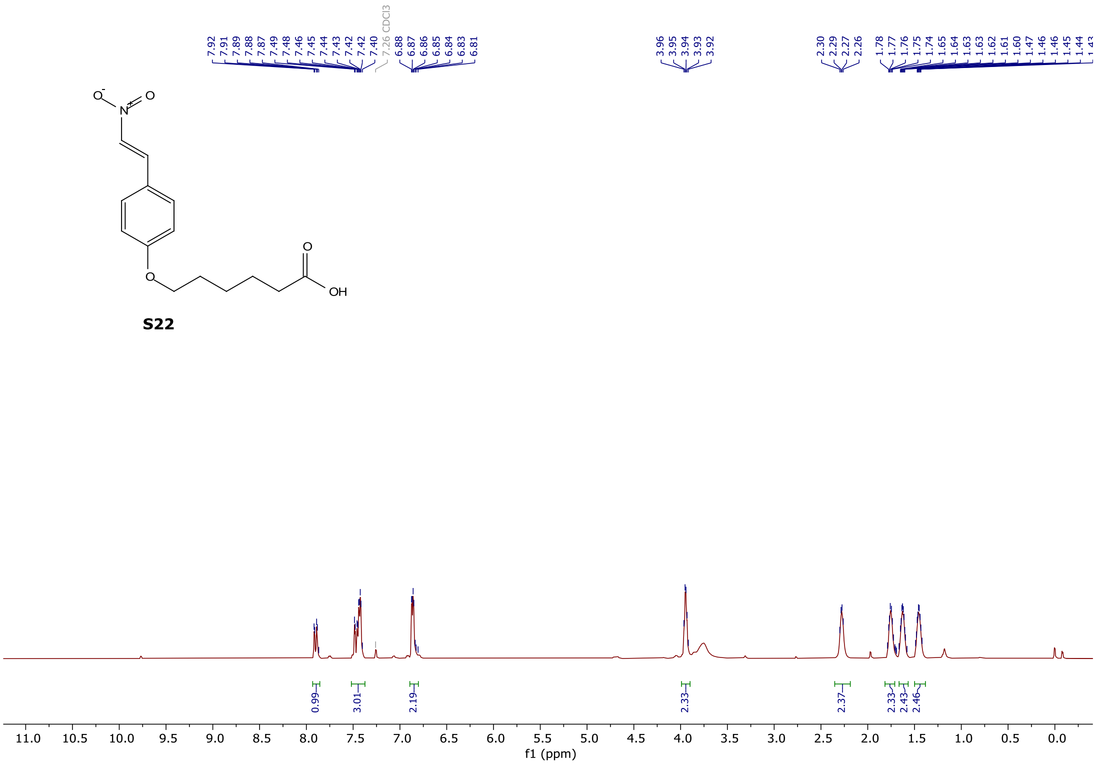

Supplementary Fig. 148. ^{1}H NMR spectrum in $CDCl_3$ of compound **S22**.

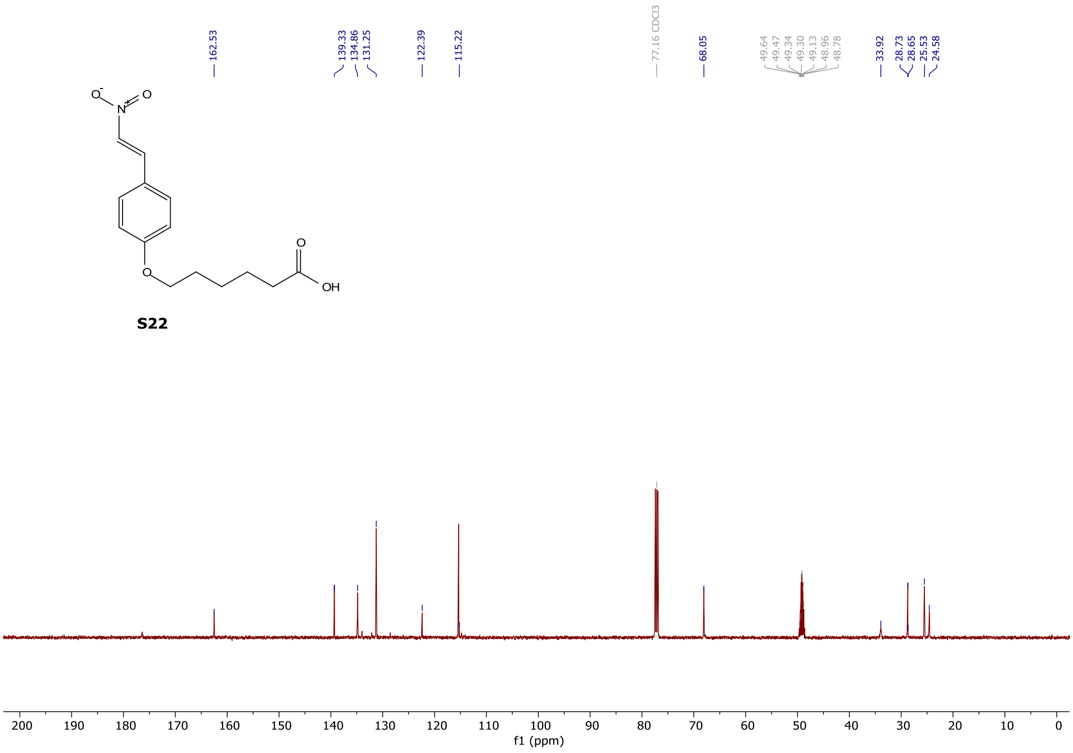

Supplementary Fig. 149. ^{13}C NMR spectrum in $CDCl_3$ of compound **S22**.

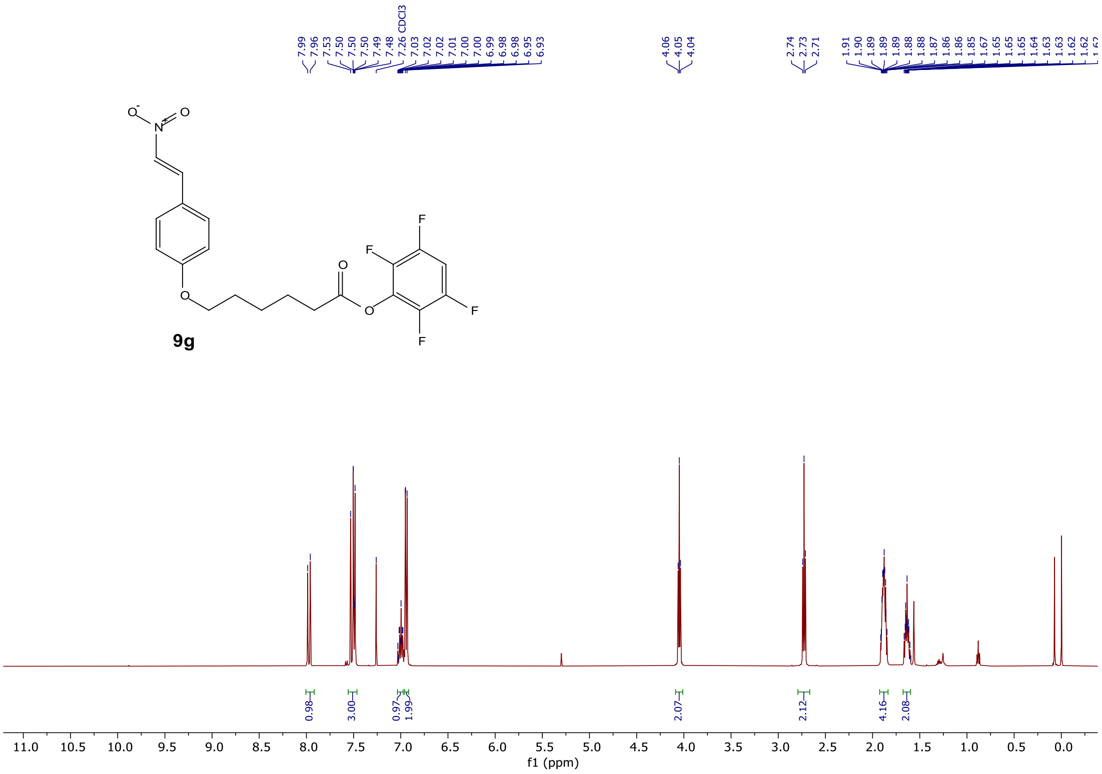

Supplementary Fig. 150. ^{1}H NMR spectrum in $CDCl_3$ of compound **9g**.

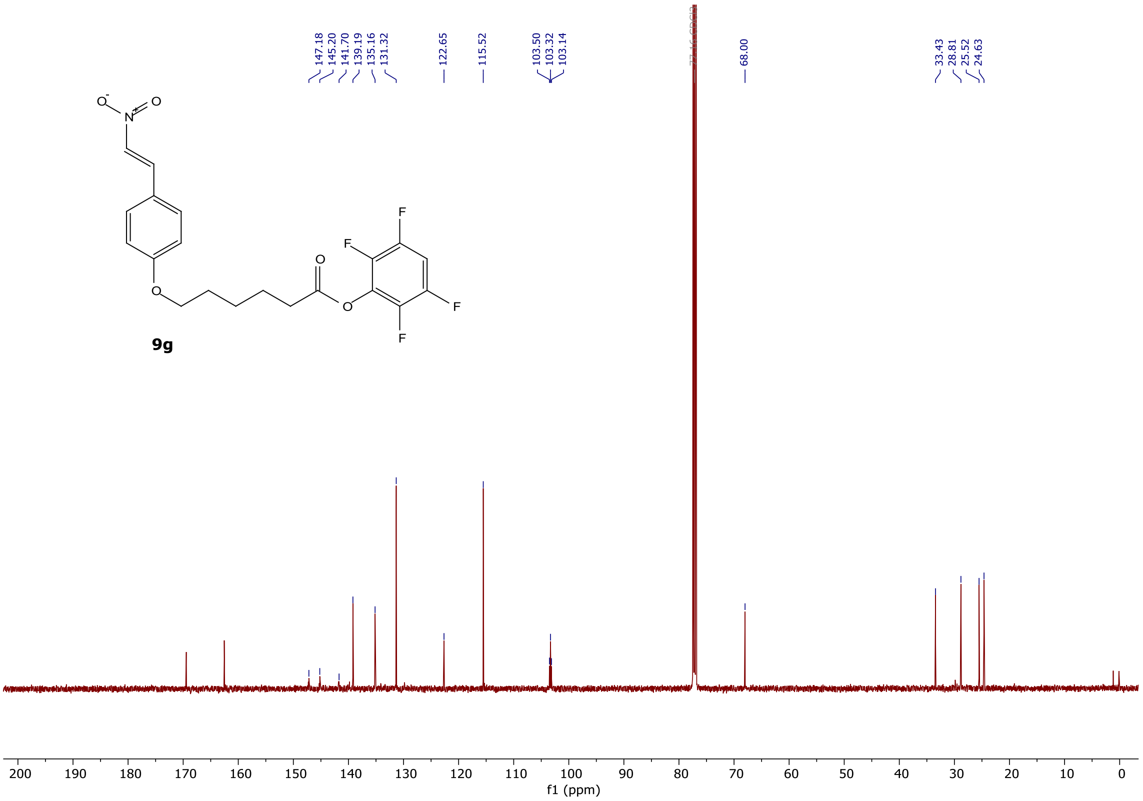

Supplementary Fig. 151. ^{13}C NMR spectrum in $CDCl_3$ of compound **9g**.

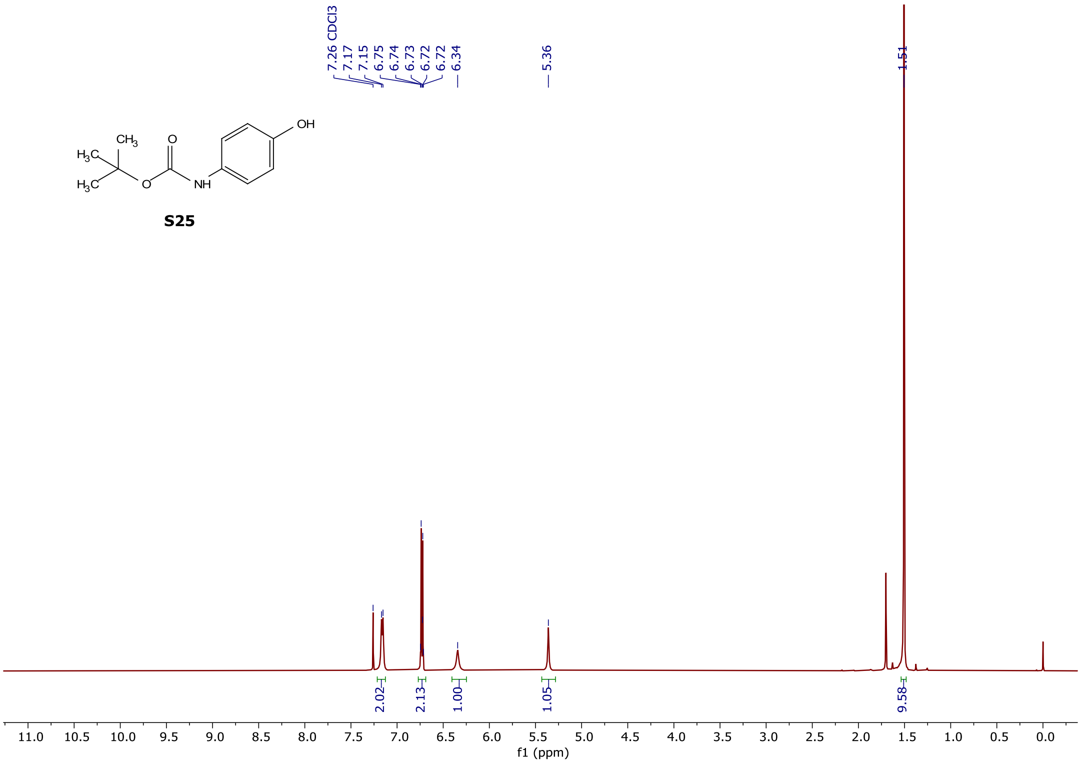

Supplementary Fig. 152. ^{1}H NMR spectrum in $CDCl_3$ of compound **S25**.

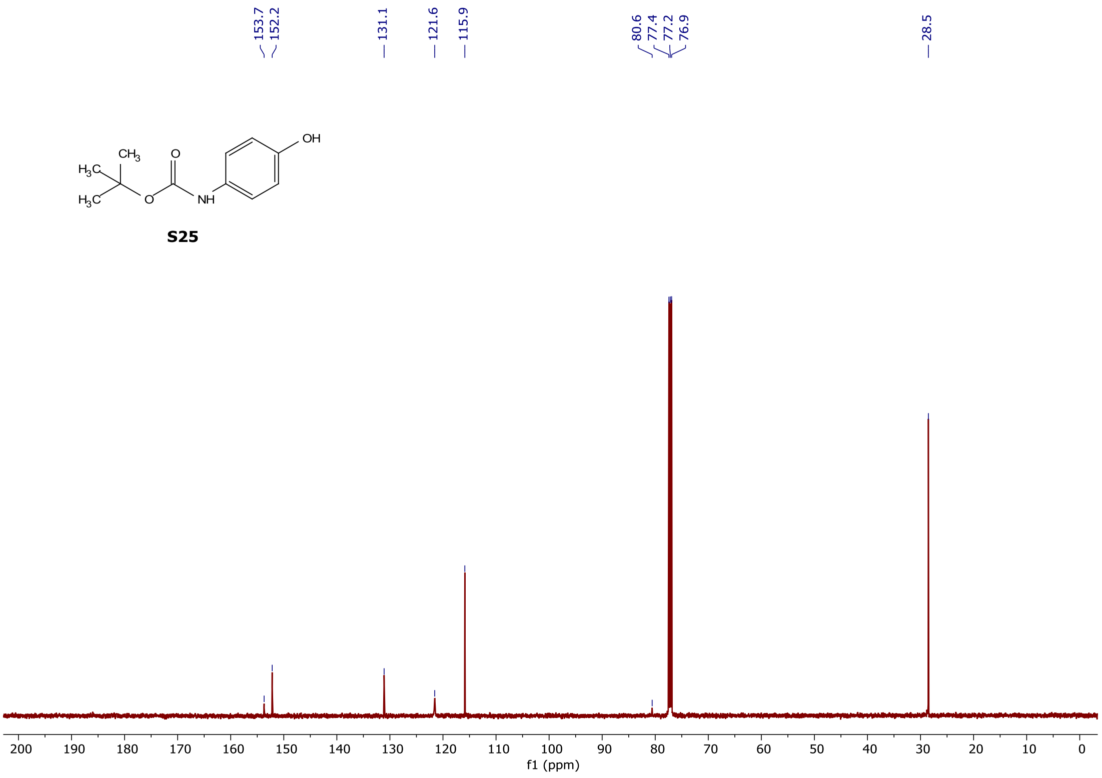

Supplementary Fig. 153. ^{13}C NMR spectrum in $CDCl_3$ of compound **S25**.

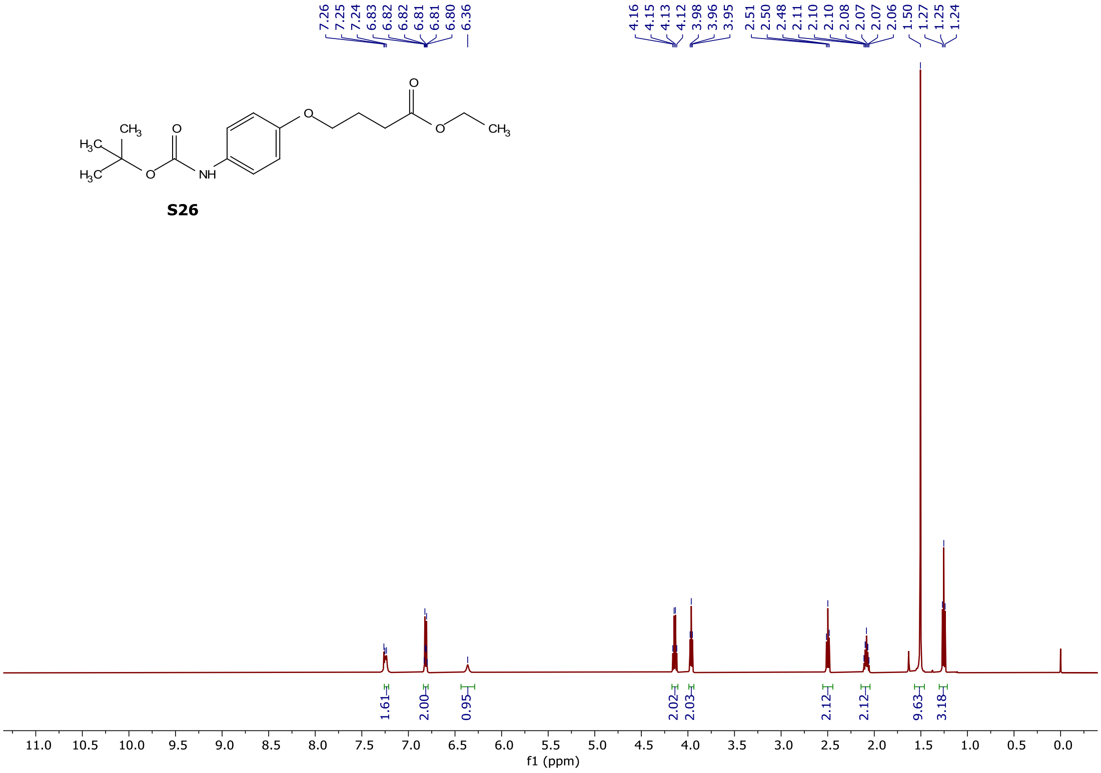

Supplementary Fig. 154. ^{1}H NMR spectrum in $CDCl_3$ of compound **S26**.

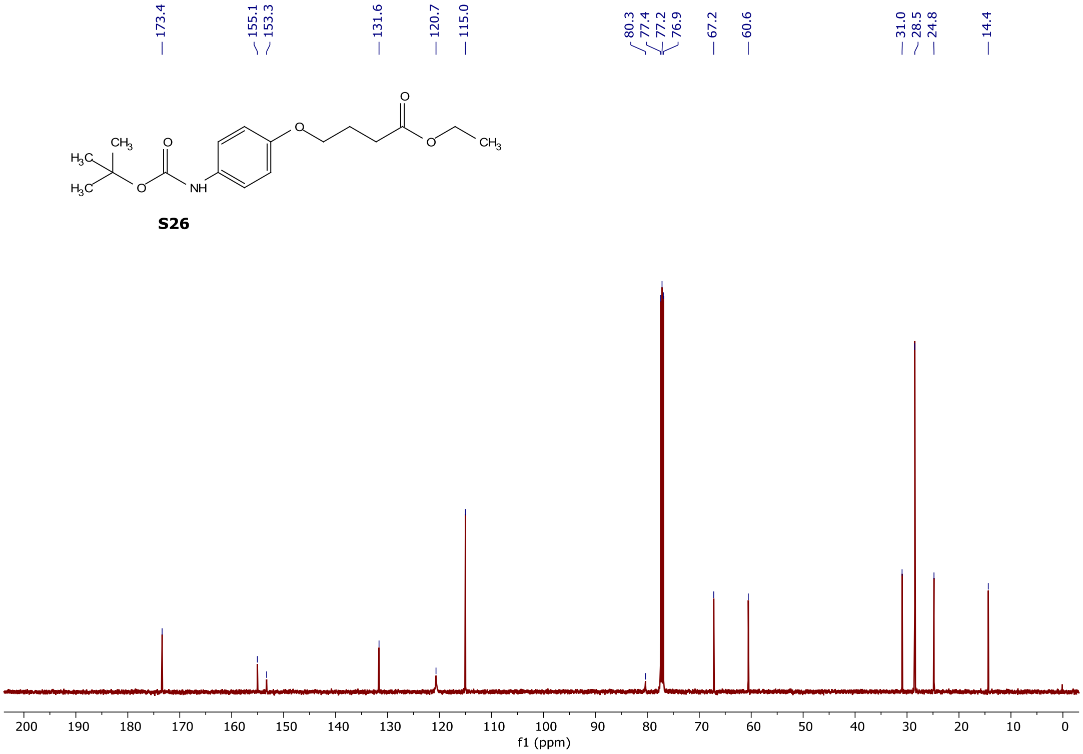

Supplementary Fig. 155. ^{13}C NMR spectrum in $CDCl_3$ of compound **S26**.

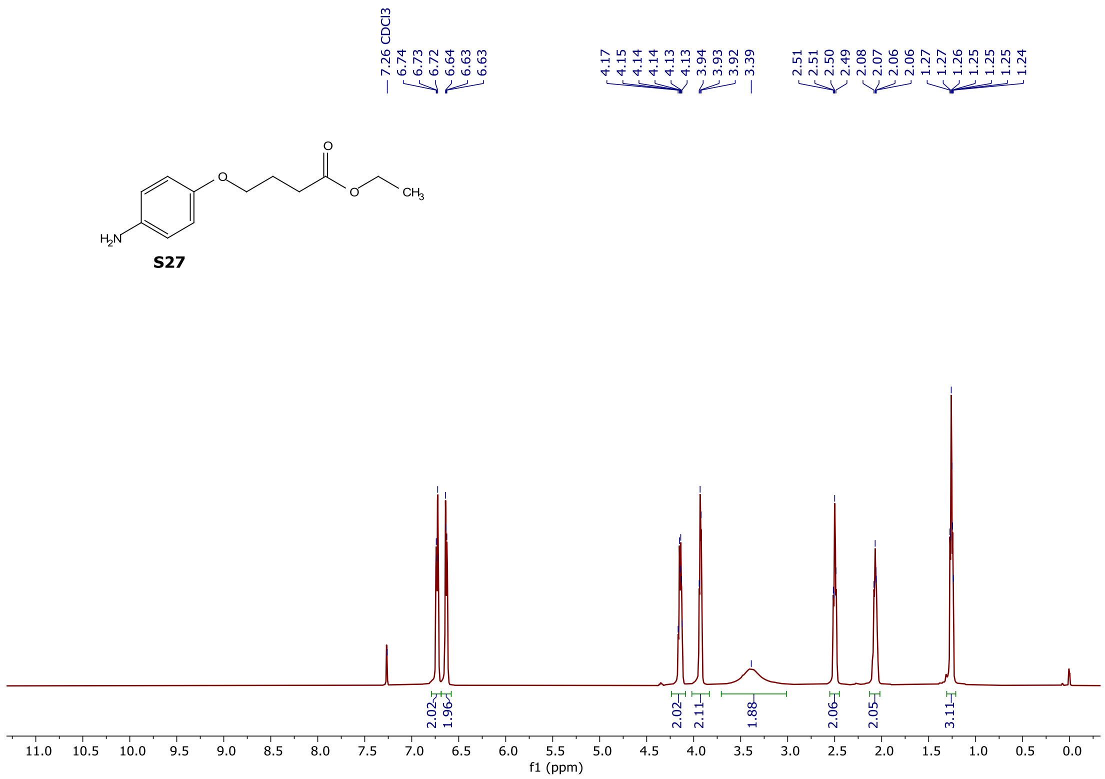

Supplementary Fig. 156. ^{1}H NMR spectrum in $CDCl_3$ of compound **S27**.

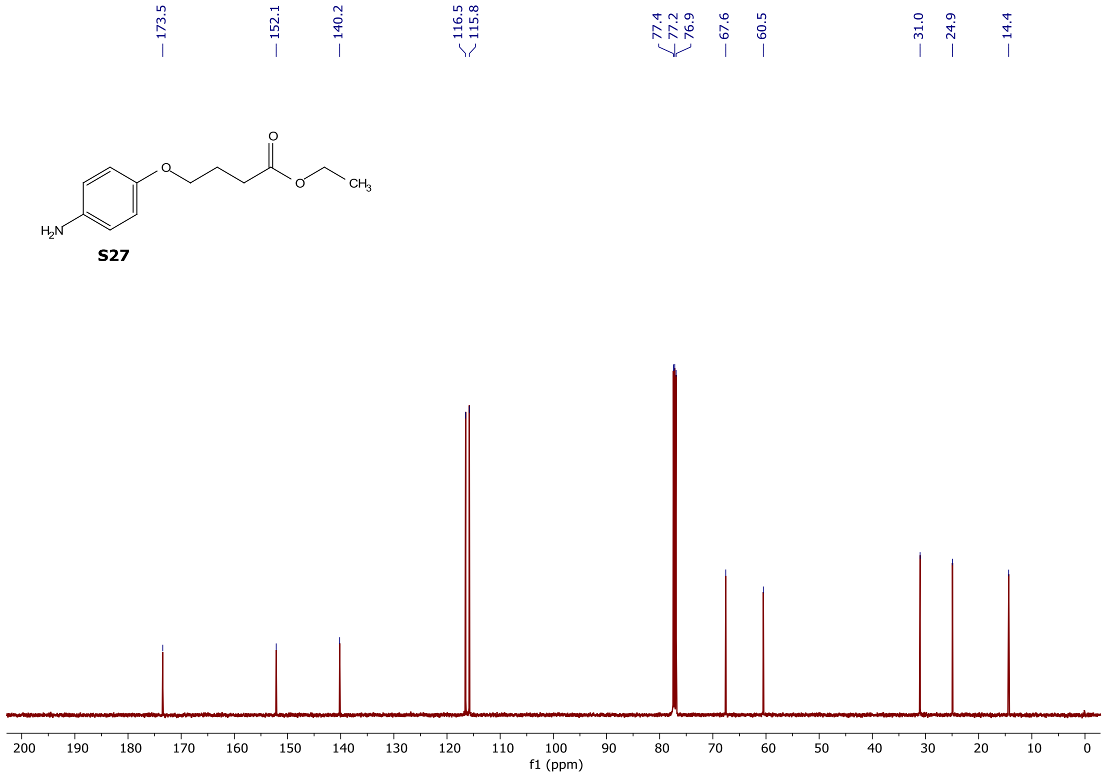

Supplementary Fig. 157. ^{13}C NMR spectrum in $CDCl_3$ of compound **S27**.

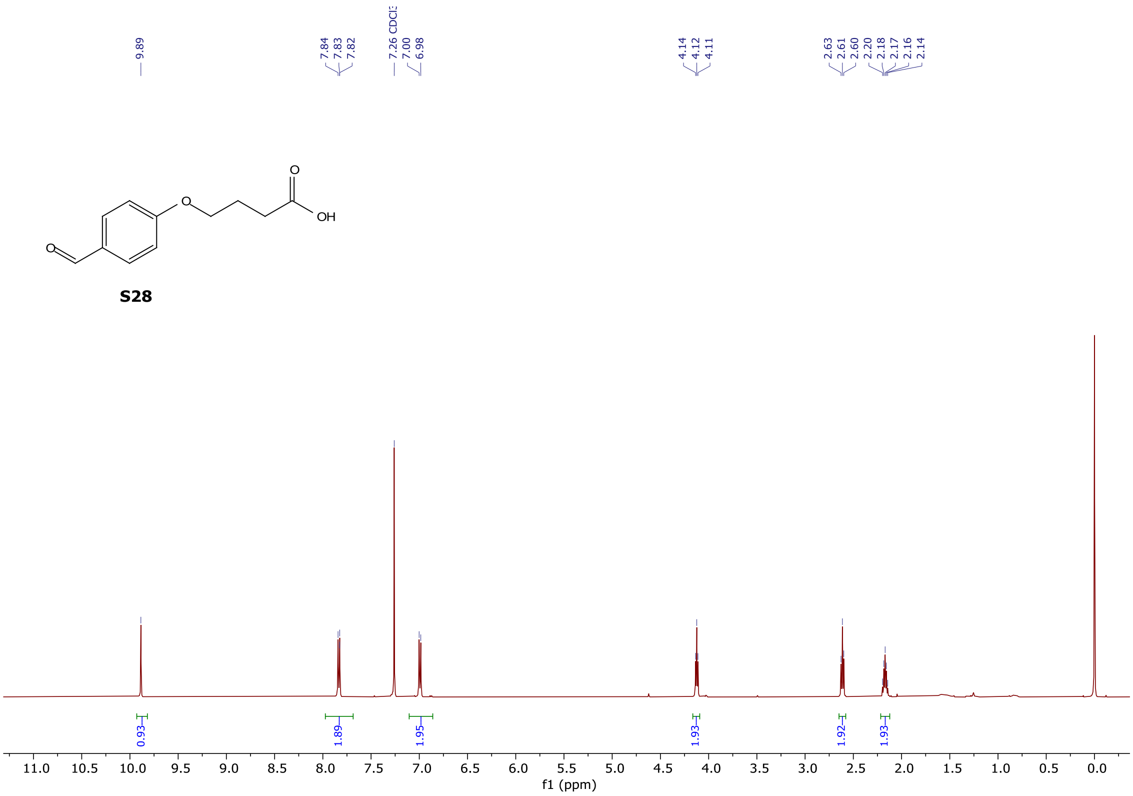

Supplementary Fig. 158. ^{1}H NMR spectrum in $CDCl_3$ of compound **S28**.

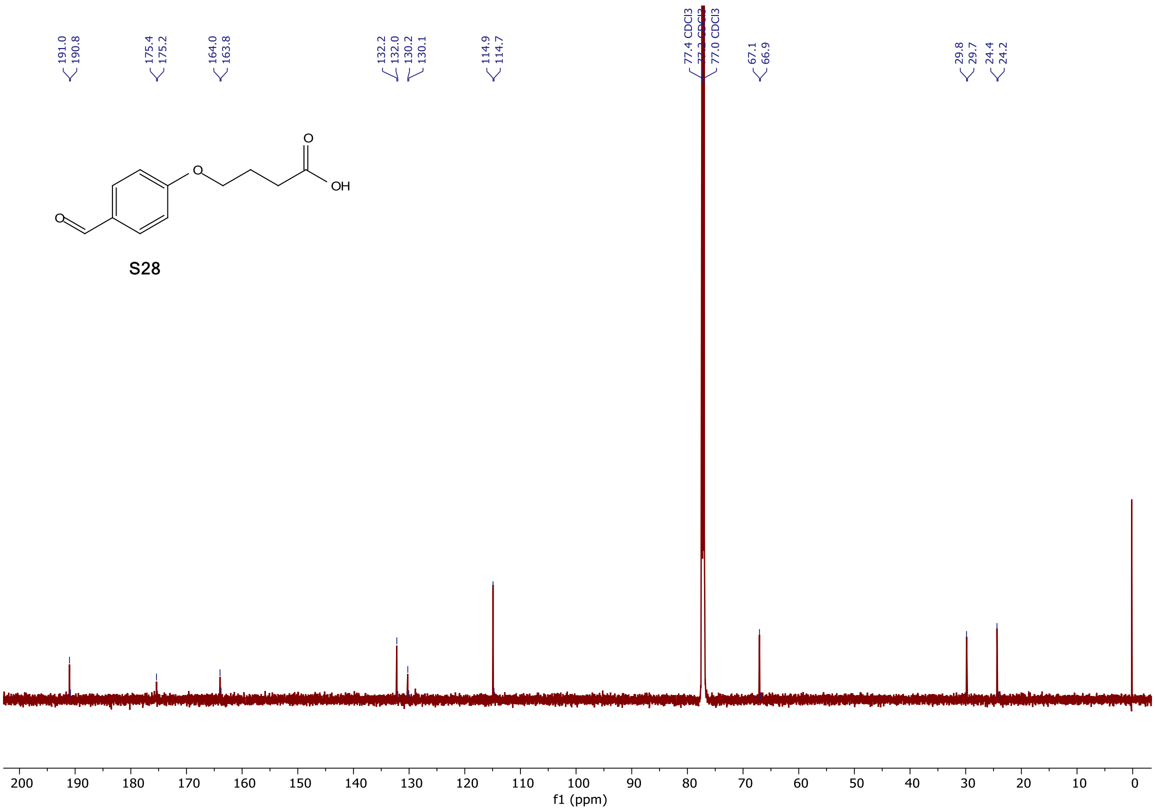

Supplementary Fig. 159. ^{13}C NMR spectrum in $CDCl_3$ of compound **S28**.

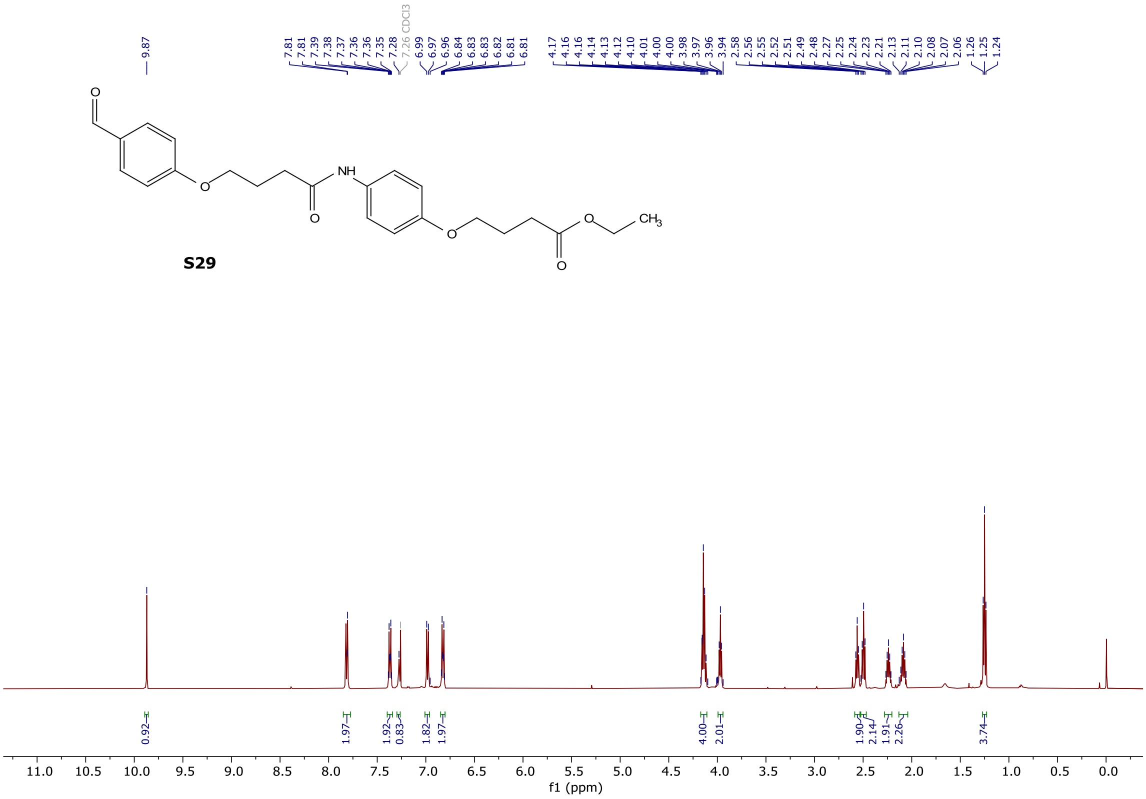

Supplementary Fig. 160. ^{1}H NMR spectrum in $CDCl_3$ of compound **S29**.

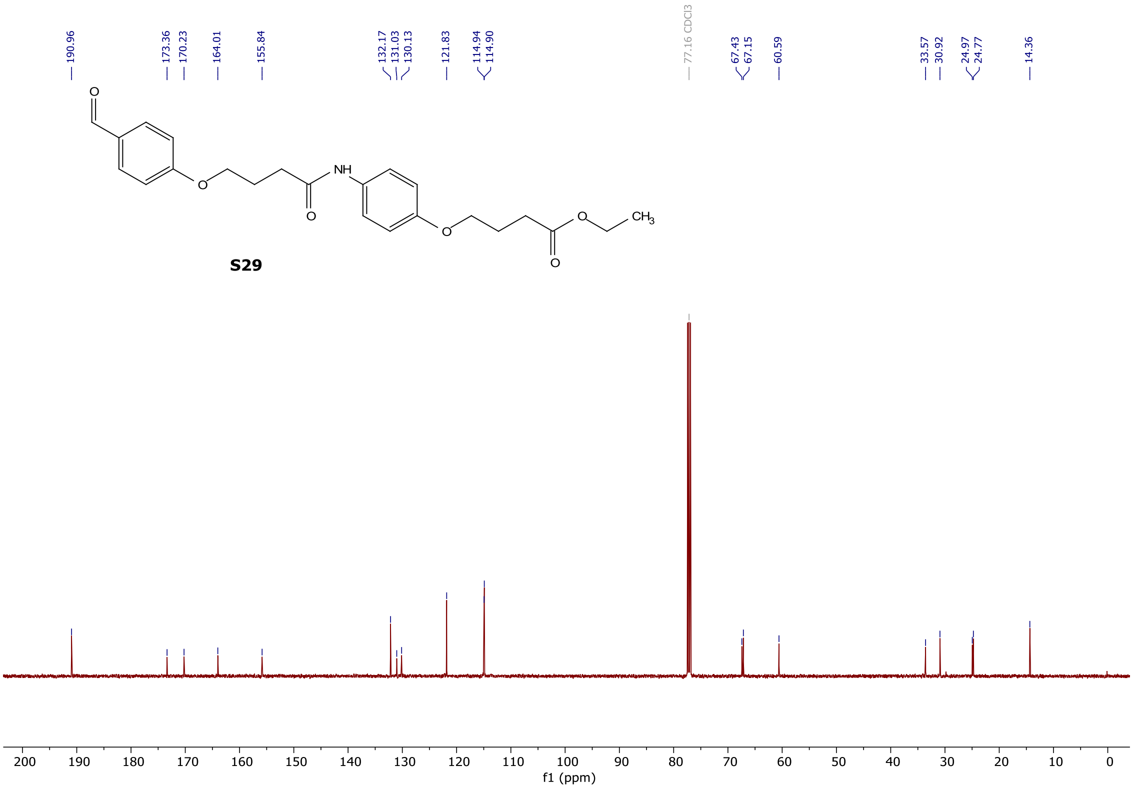

Supplementary Fig. 161. ^{13}C NMR spectrum in $CDCl_3$ of compound **S29**.

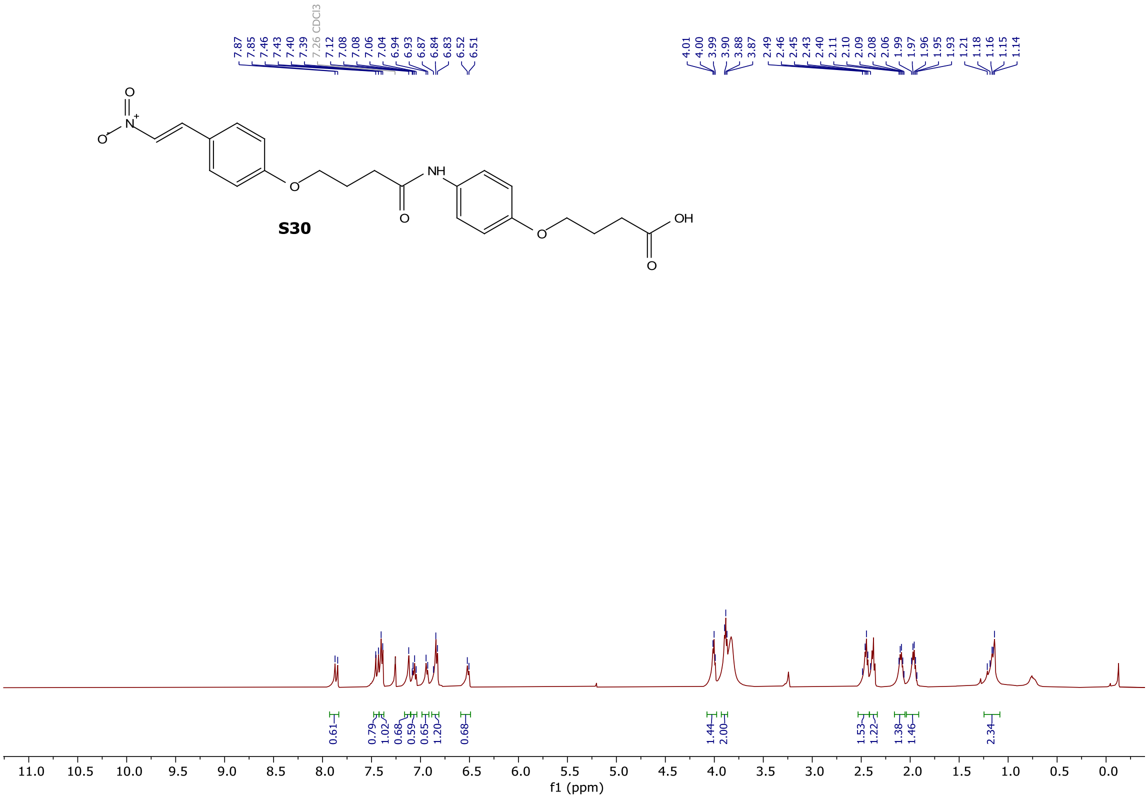

Supplementary Fig. 162. ^{1}H NMR spectrum in $CDCl_3$ of compound **S30**.

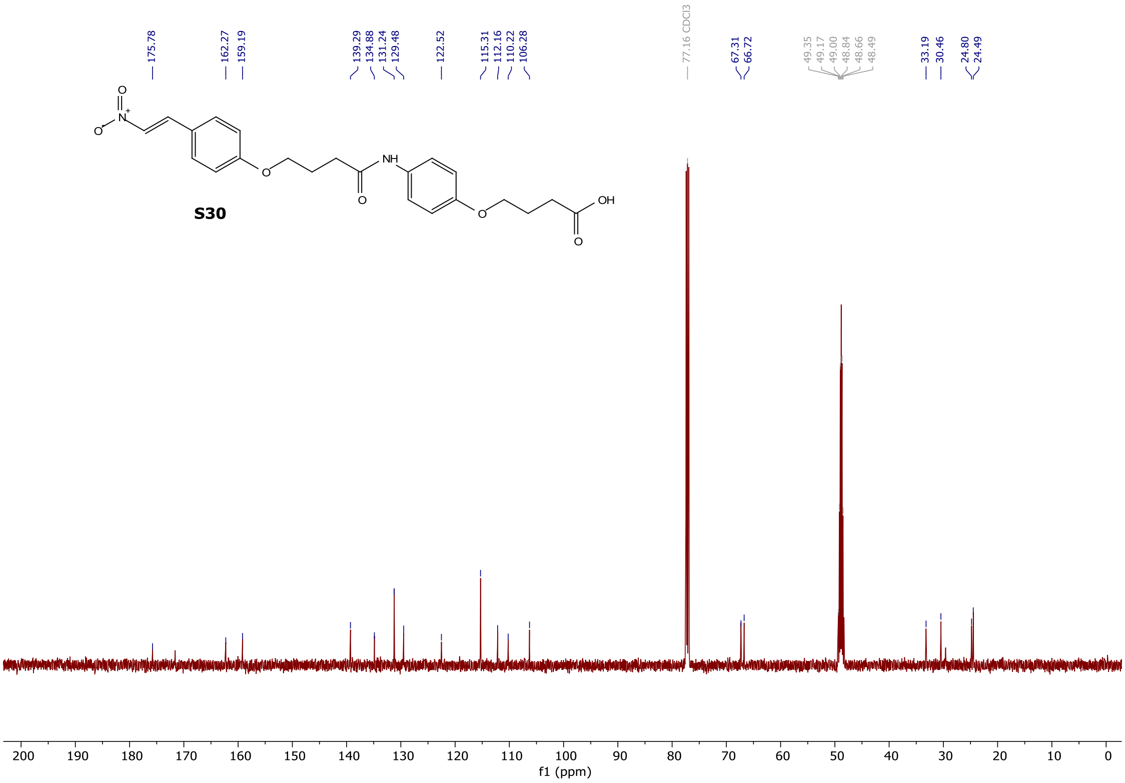

Supplementary Fig. 163. ^{13}C NMR spectrum in $CDCl_3$ of compound **S30**.

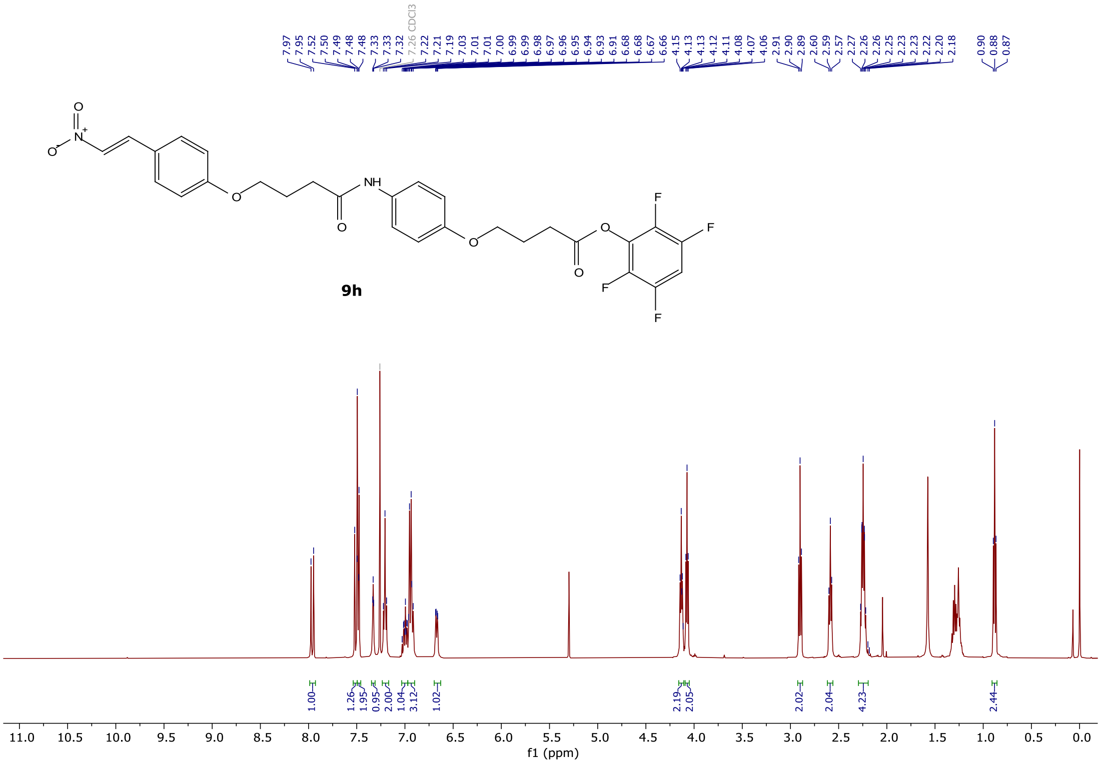

Supplementary Fig. 164. ^{1}H NMR spectrum in $CDCl_3$ of compound **9h**.

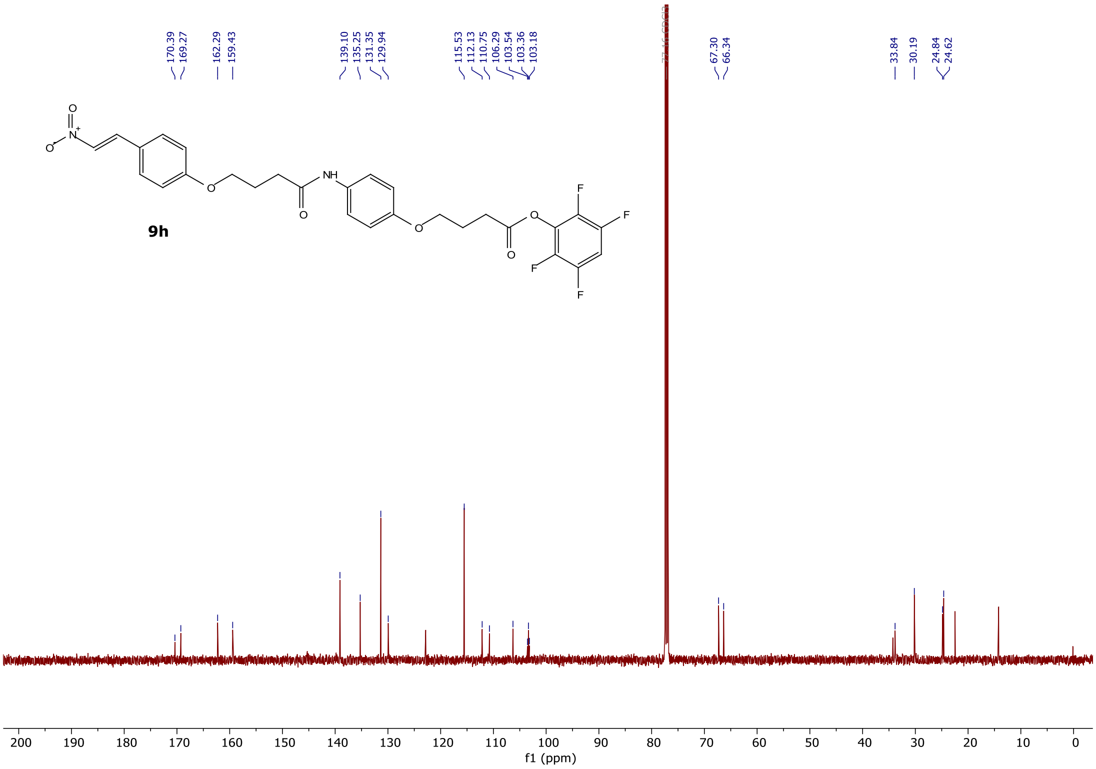

Supplementary Fig. 165. ^{13}C NMR spectrum in $CDCl_3$ of compound **9h**.

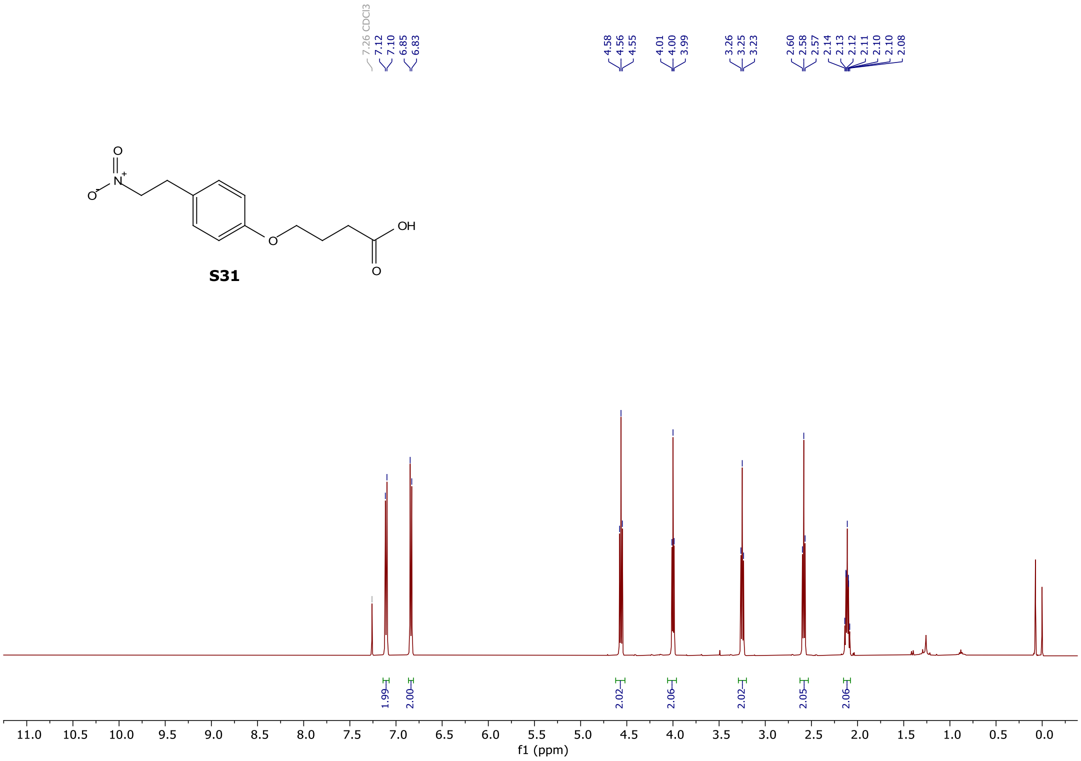

Supplementary Fig. 166. ^{1}H NMR spectrum in $CDCl_3$ of compound **S31**.

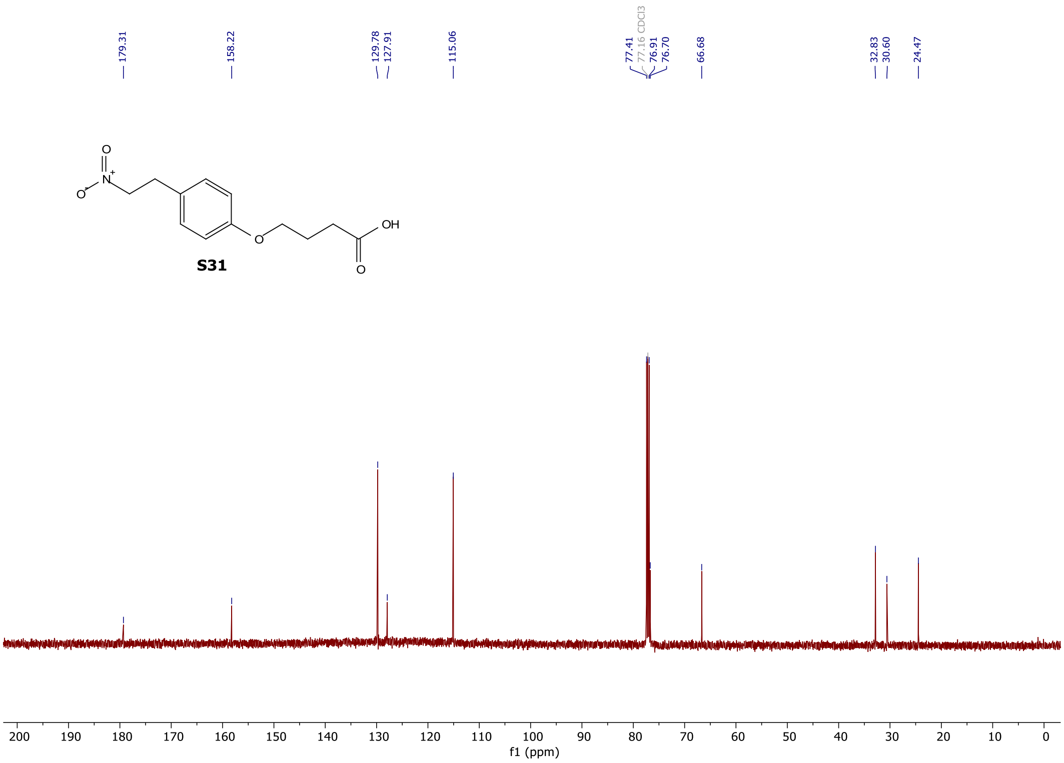

Supplementary Fig. 167. ^{13}C NMR spectrum in $CDCl_3$ of compound **S31**.

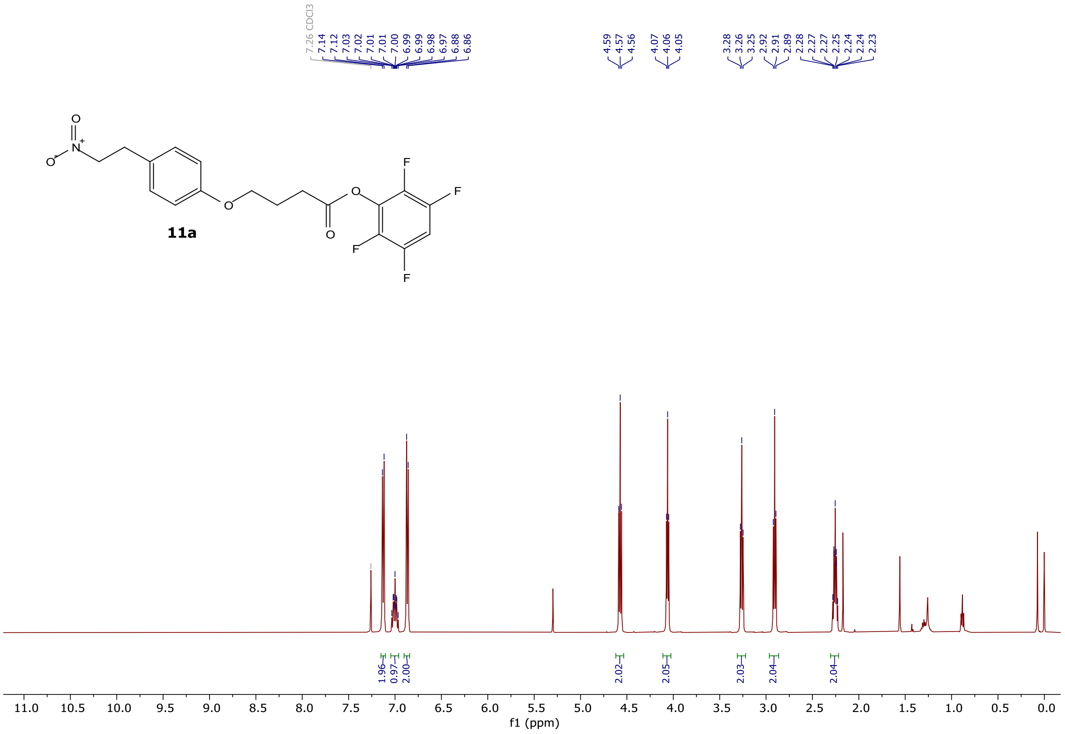

Supplementary Fig. 168. ^{1}H NMR spectrum in $CDCl_3$ of compound **11a**.

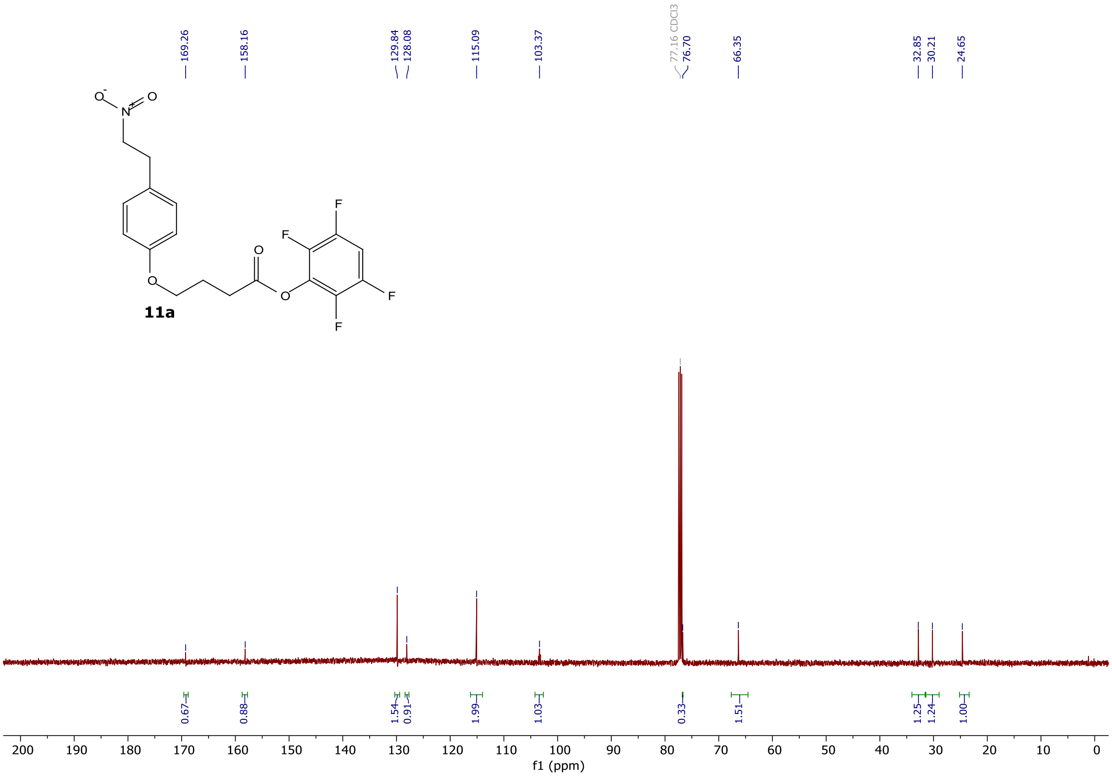

Supplementary Fig. 169. ^{13}C NMR spectrum in $CDCl_3$ of compound **11a**.

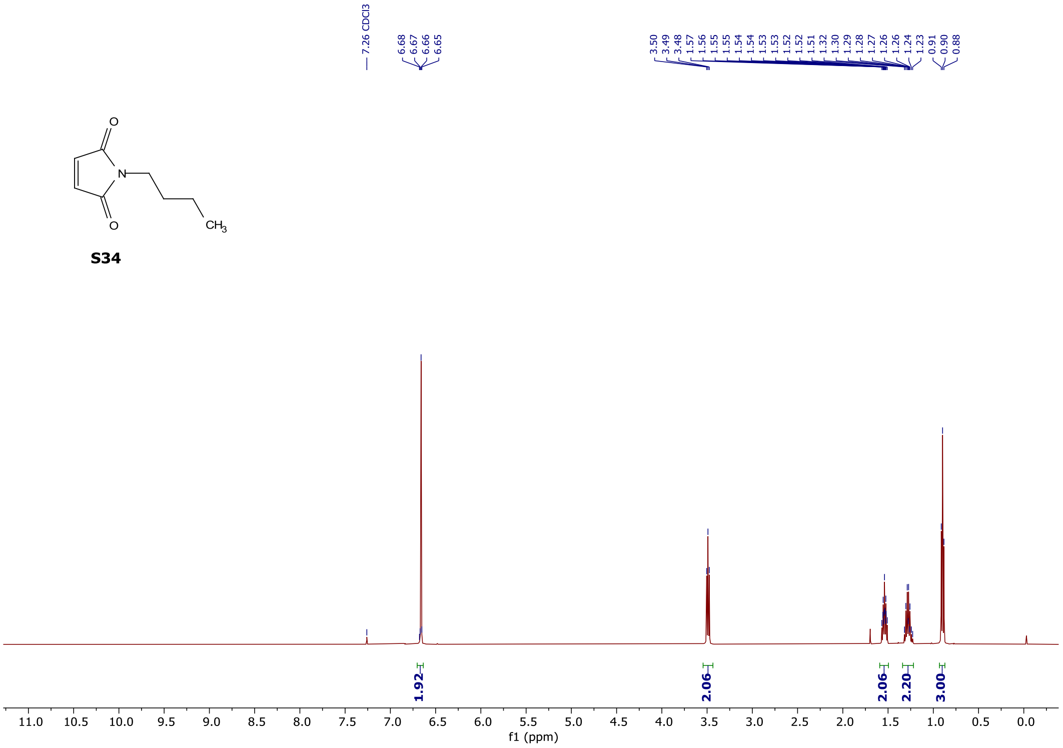

Supplementary Fig. 170. ^{1}H NMR spectrum in $CDCl_3$ of compound **S34**.

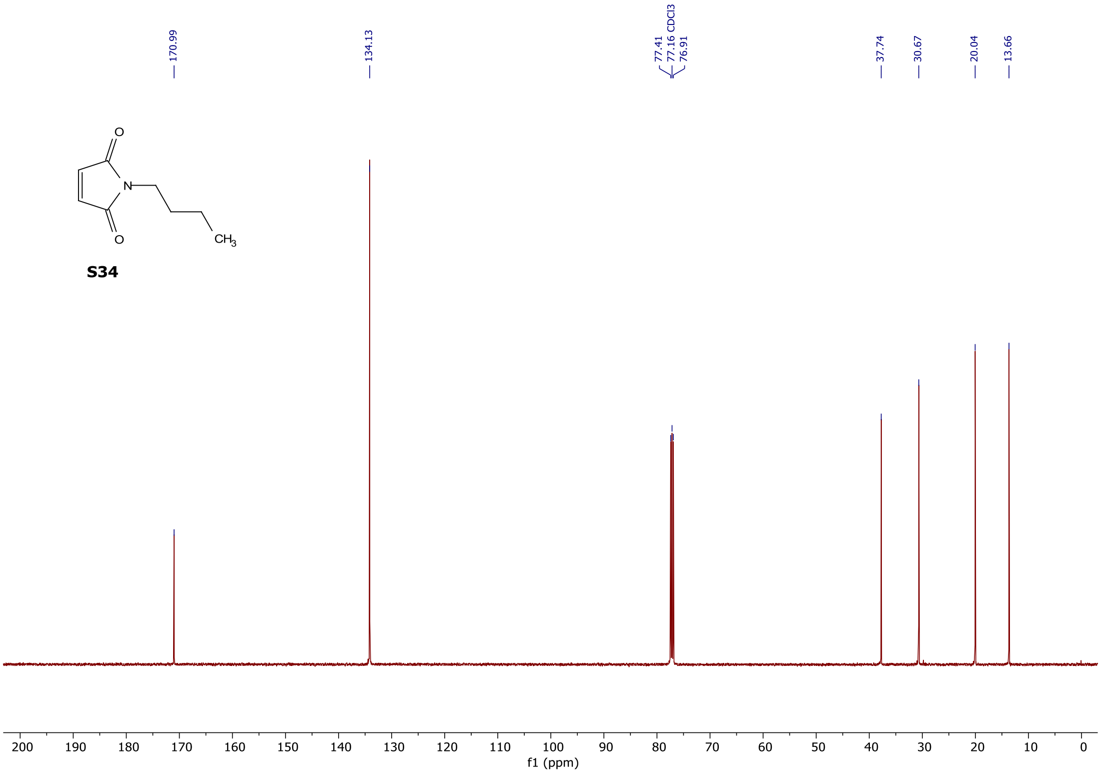

Supplementary Fig. 171. ^{13}C NMR spectrum in $CDCl_3$ of compound **S34**.

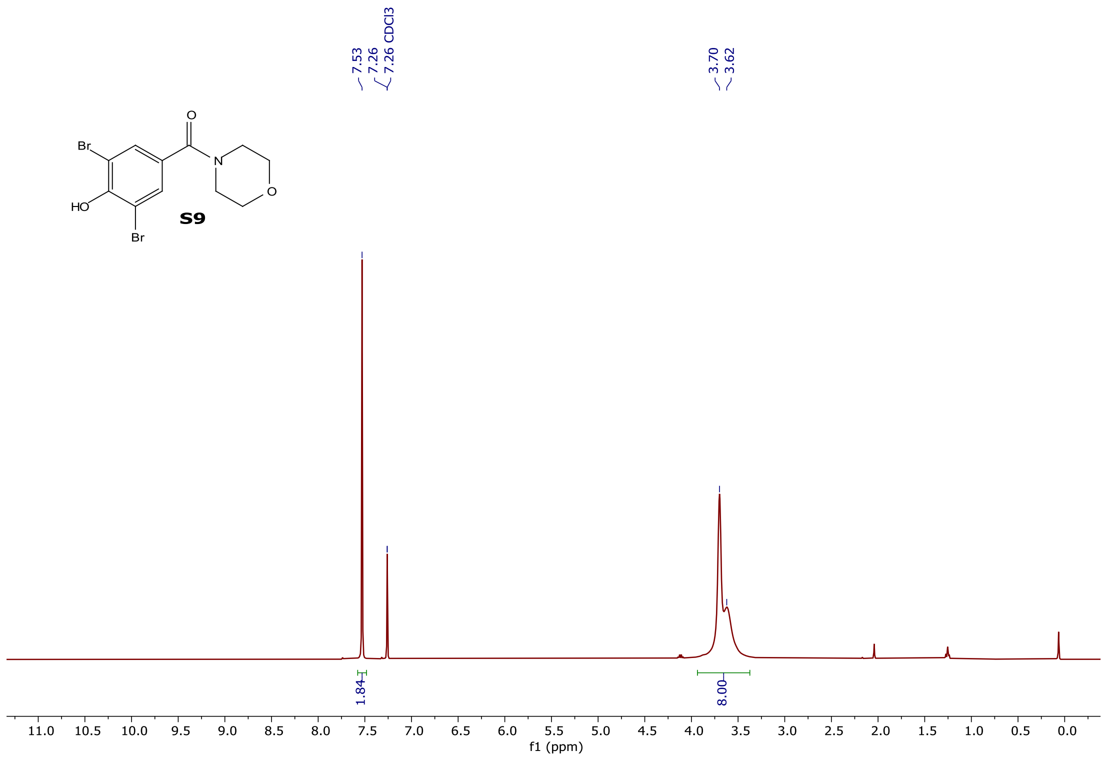

Supplementary Fig. 172. ^{1}H NMR spectrum in $CDCl_3$ of compound **S9**.

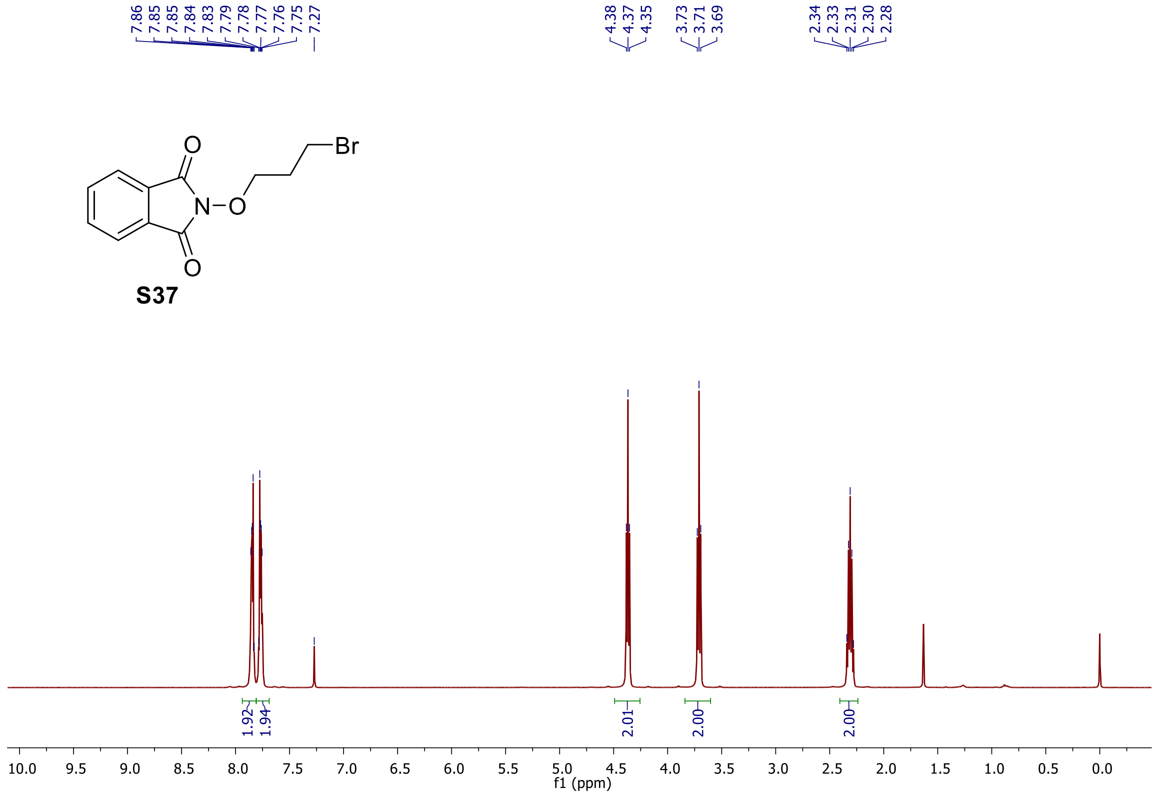

Supplementary Fig. 173. ^{1}H NMR spectrum in $CDCl_3$ of compound **S37**.

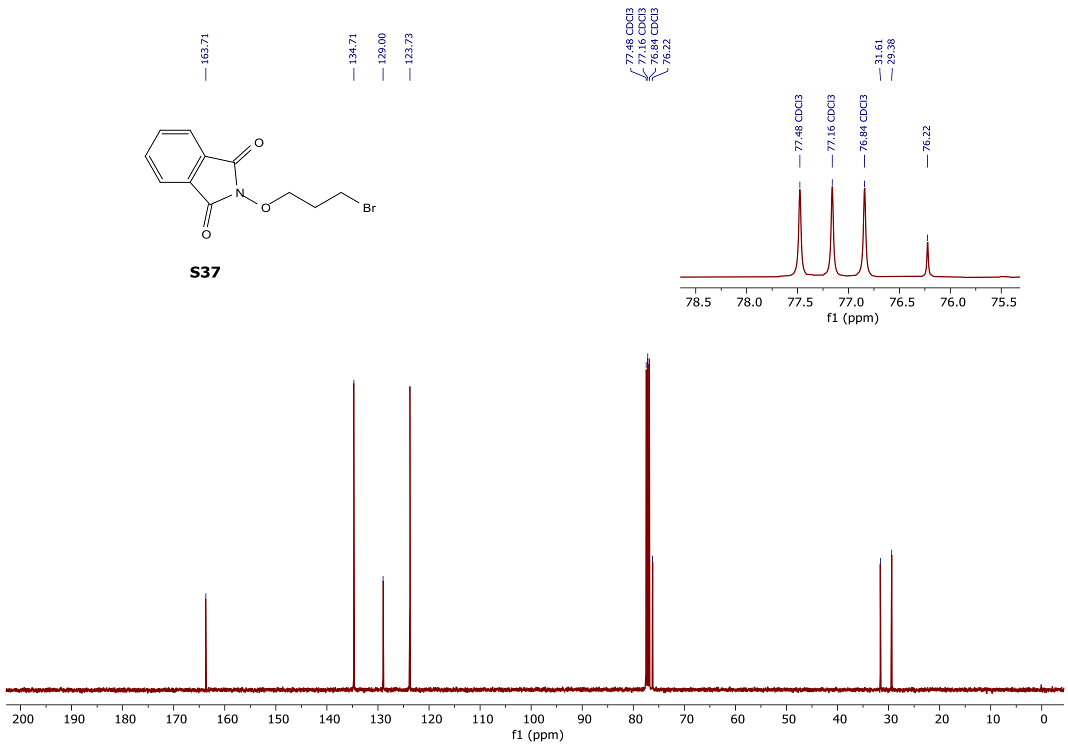

Supplementary Fig. 174. ^{13}C NMR spectrum in $CDCl_3$ of compound **S37**.

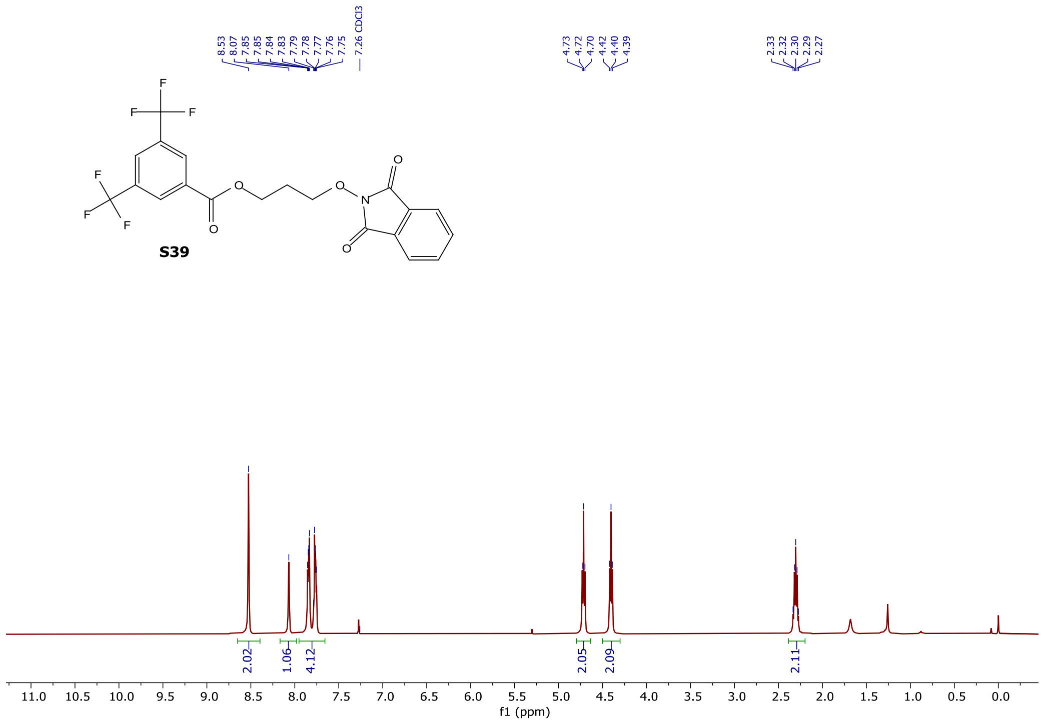

Supplementary Fig. 175. ^{1}H NMR spectrum in $CDCl_3$ of compound **S39**.

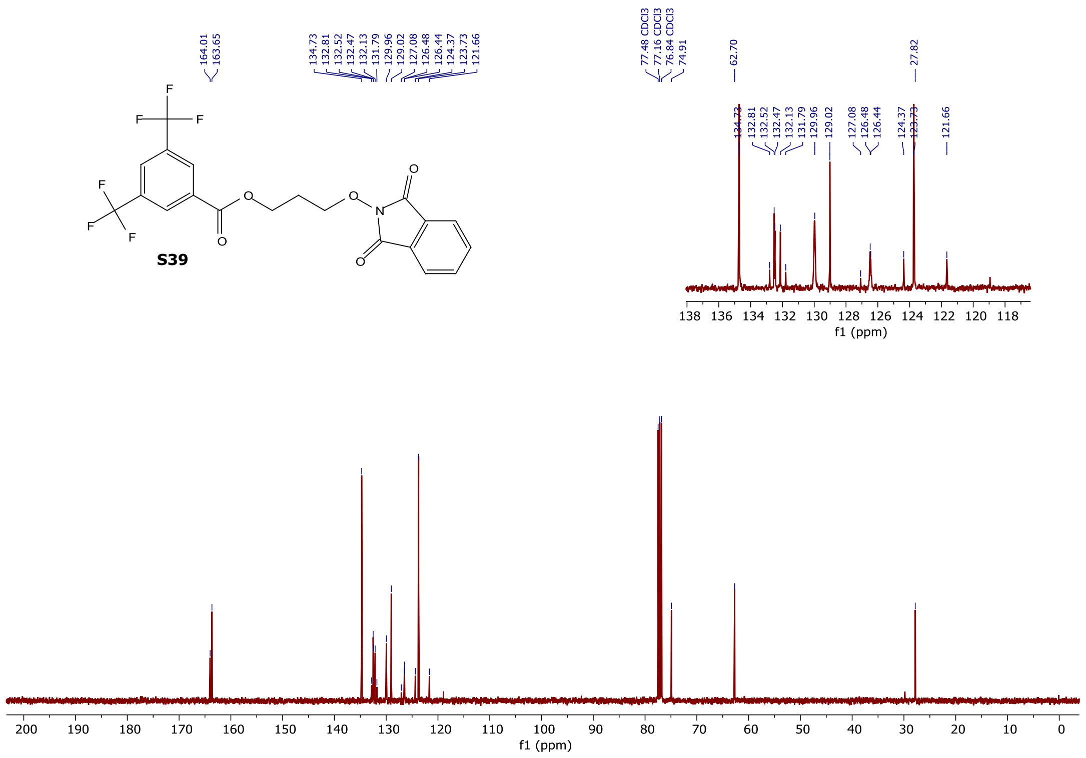

Supplementary Fig. 176. ^{13}C NMR spectrum in $CDCl_3$ of compound **S39**.

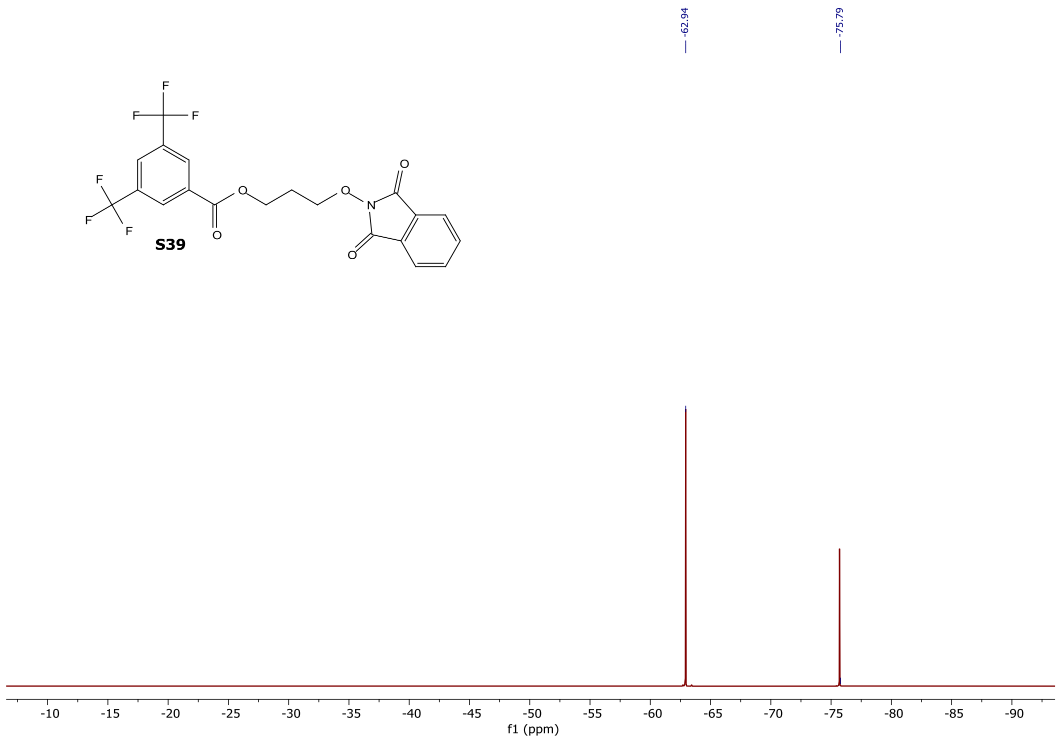

Supplementary Fig. 177. ^{1}H NMR spectrum in $CDCl_3$ of compound **S39**.

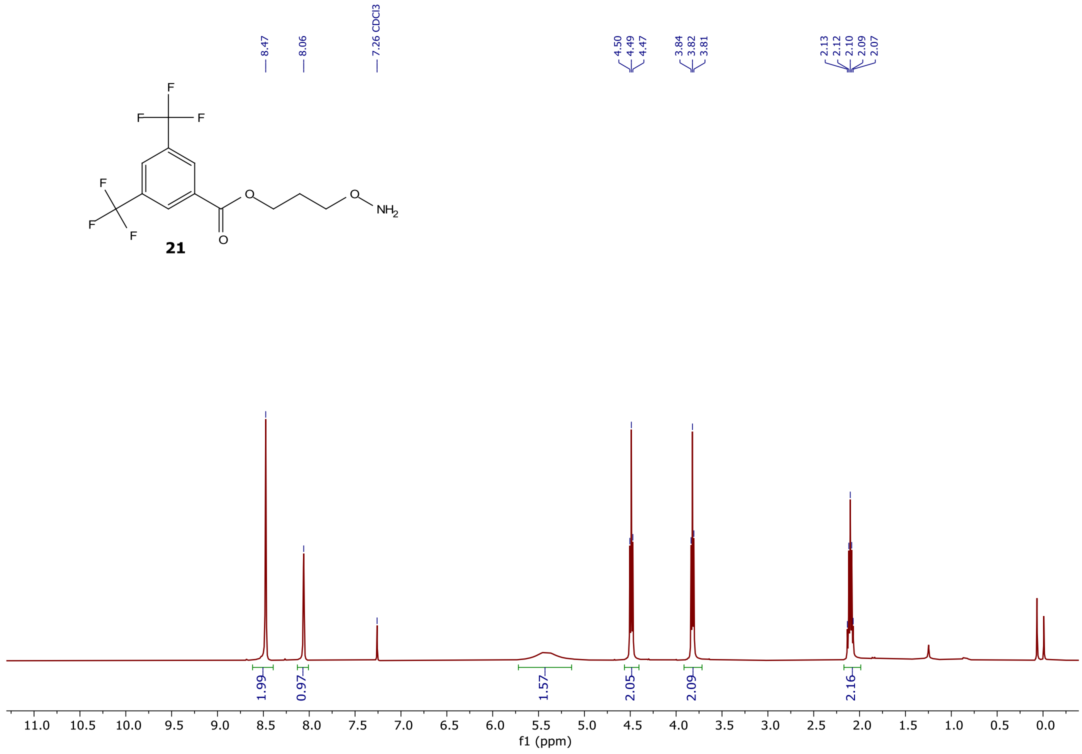

Supplementary Fig. 178. ^{1}H NMR spectrum in $CDCl_3$ of compound **21**.

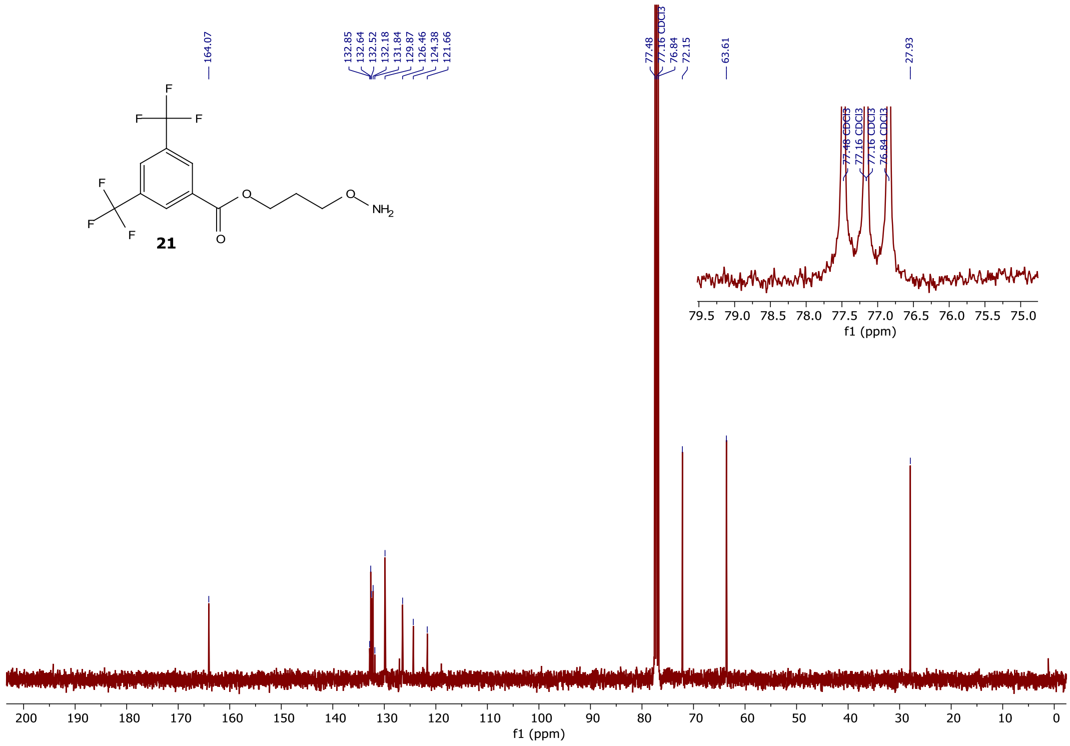

Supplementary Fig. 179. ^{13}C NMR spectrum in $CDCl_3$ of compound **21**.

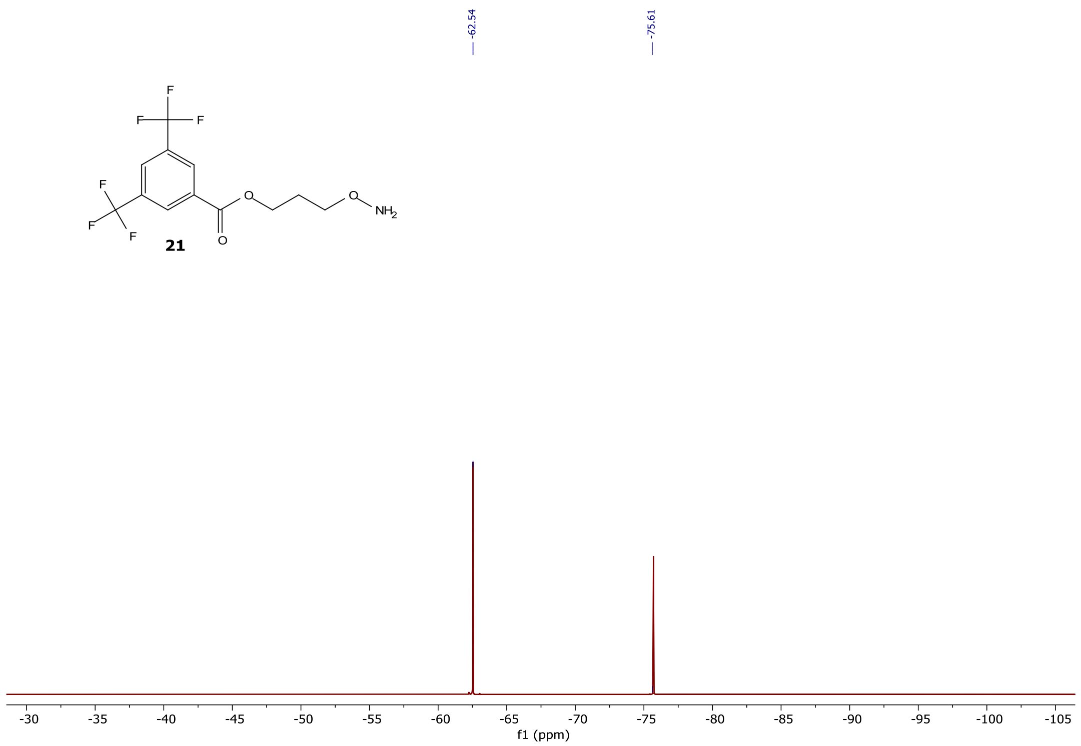

Supplementary Fig. 180. ^{19}F NMR spectrum in $CDCl_3$ of compound **21**.

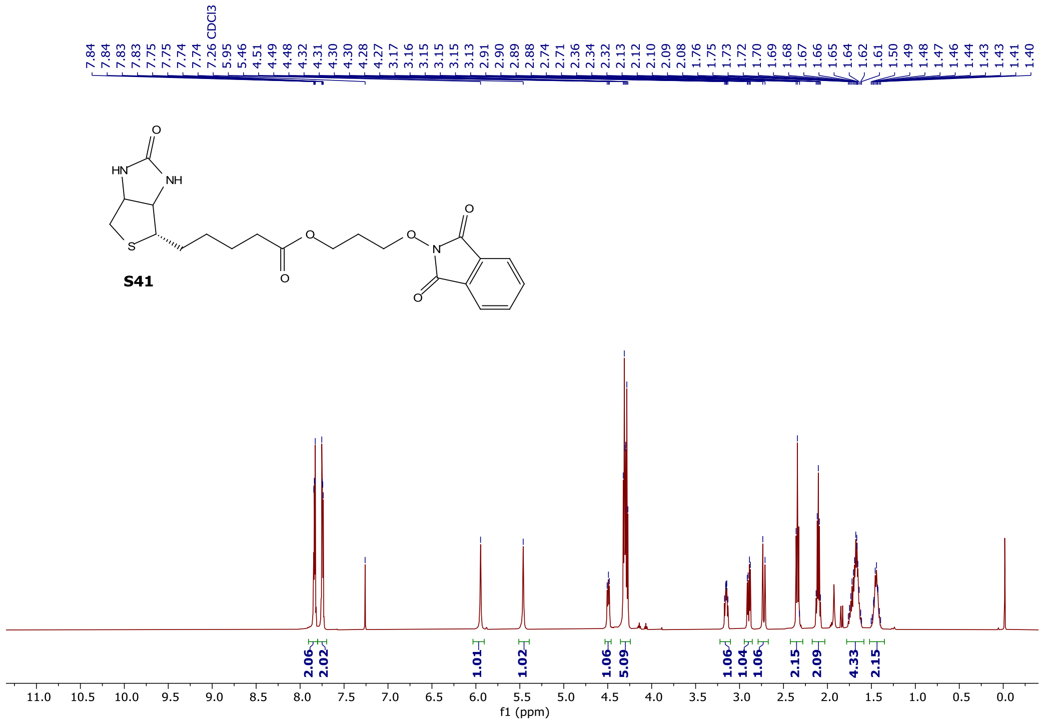

Supplementary Fig. 181. ^{1}H NMR spectrum in $CDCl_3$ of compound **S41**.

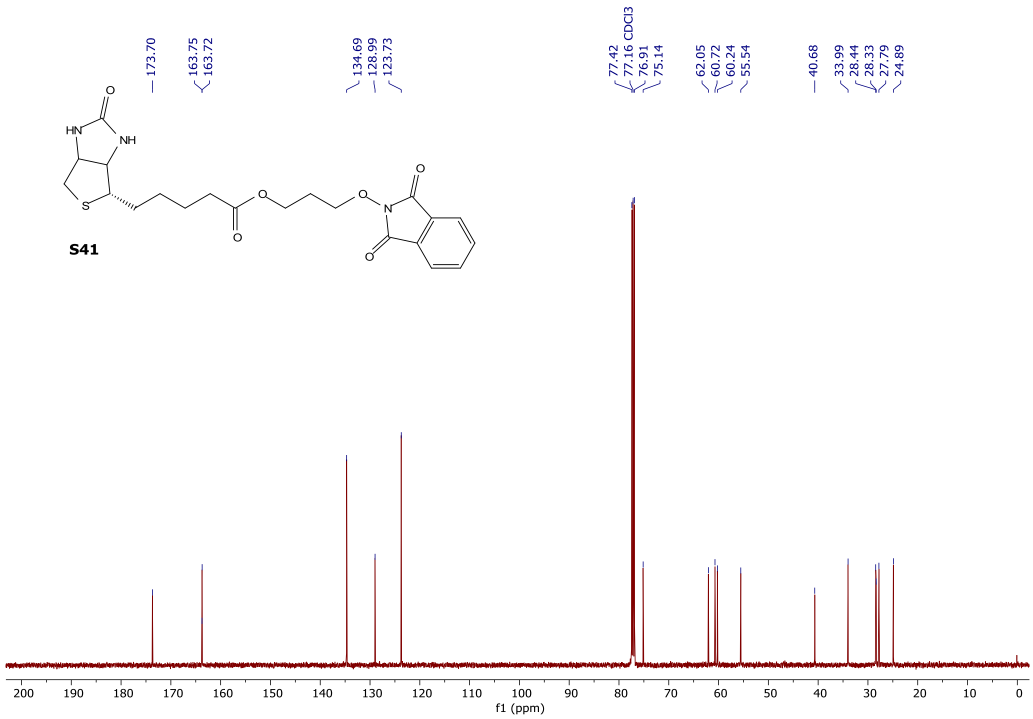

Supplementary Fig. 182. ^{13}C NMR spectrum in $CDCl_3$ of compound **S41**.

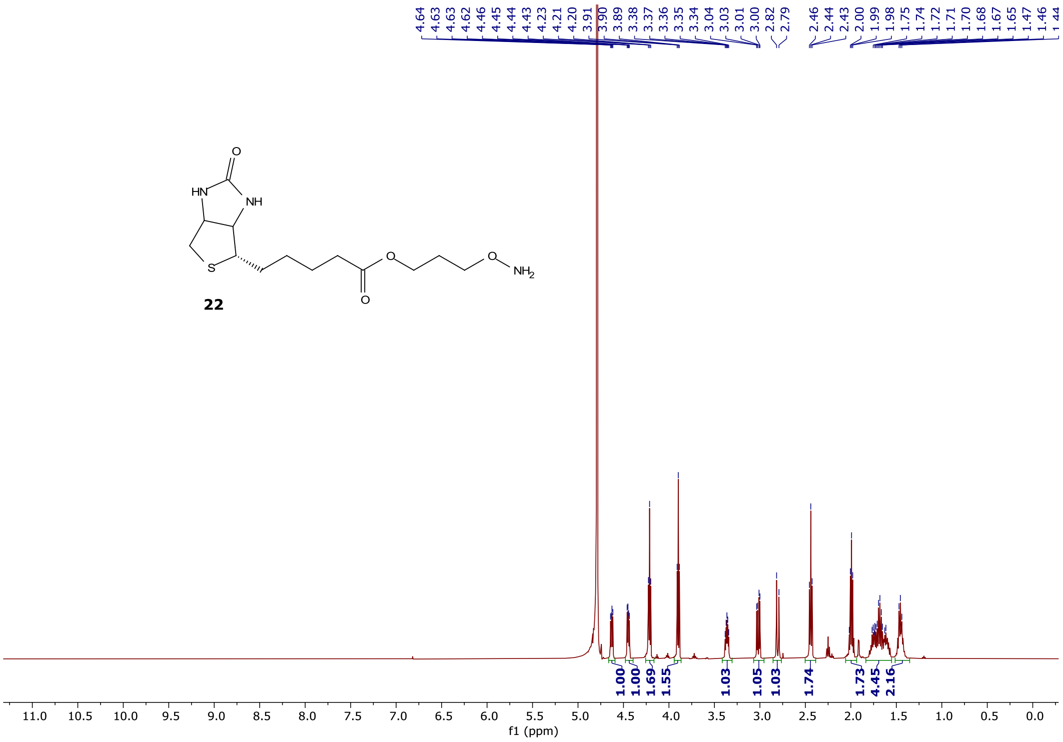

Supplementary Fig. 183. ^{1}H NMR spectrum in D_2O of compound **22**.

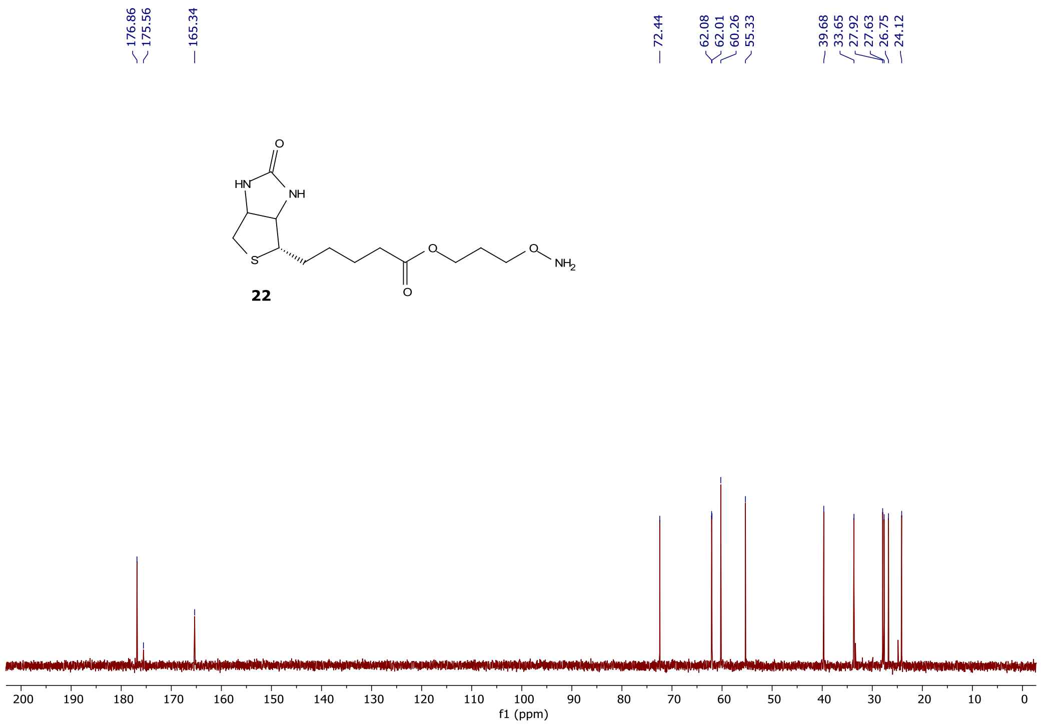

Supplementary Fig. 184. ^{13}C NMR spectrum in D_2O of compound **22**.

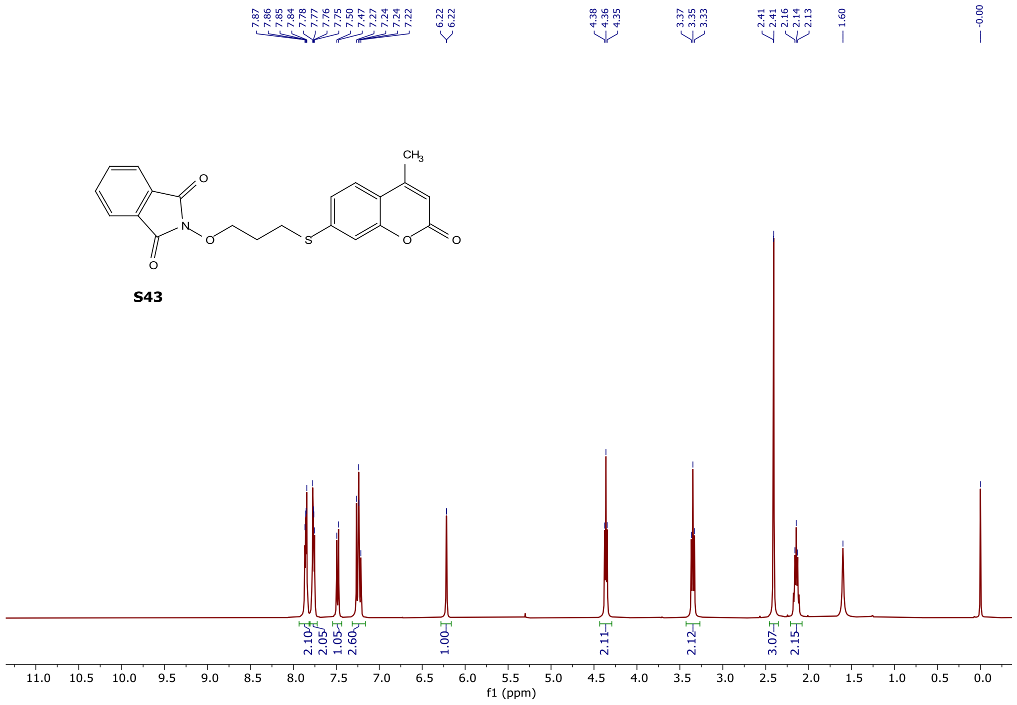

Supplementary Fig. 185. ^{1}H NMR spectrum in $CDCl_3$ of compound **S43**.

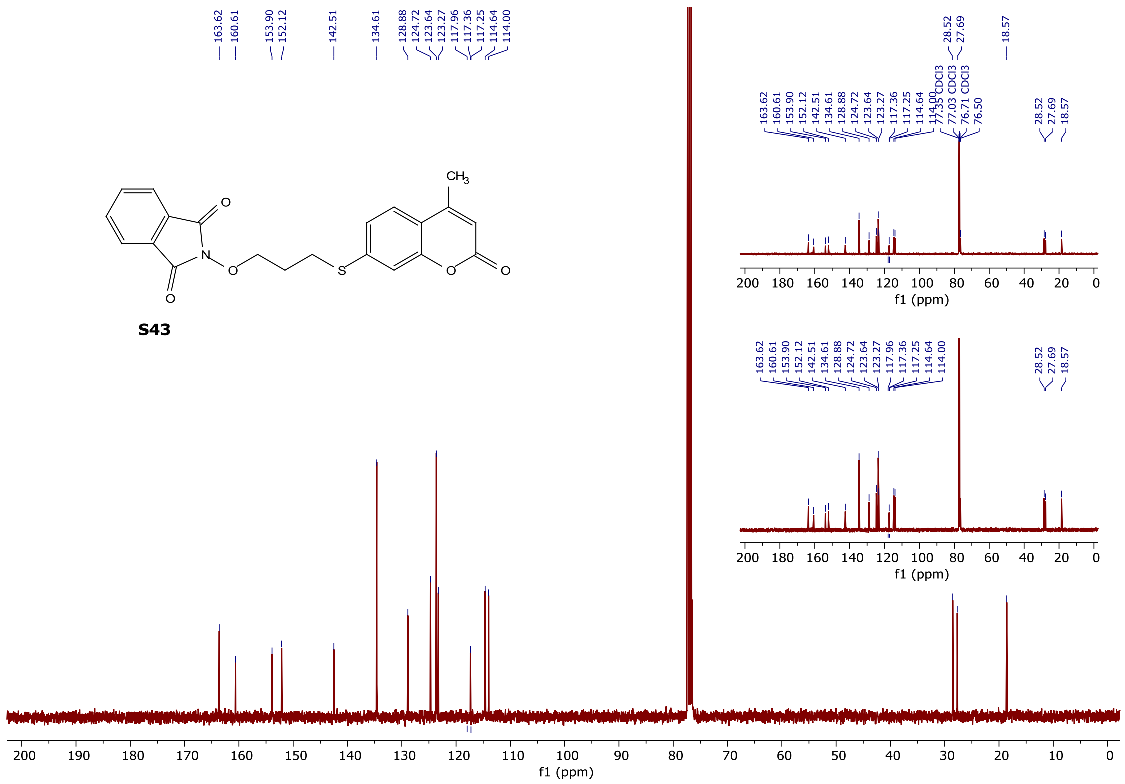

Supplementary Fig. 186. ^{13}C NMR spectrum in $CDCl_3$ of compound **S43**.

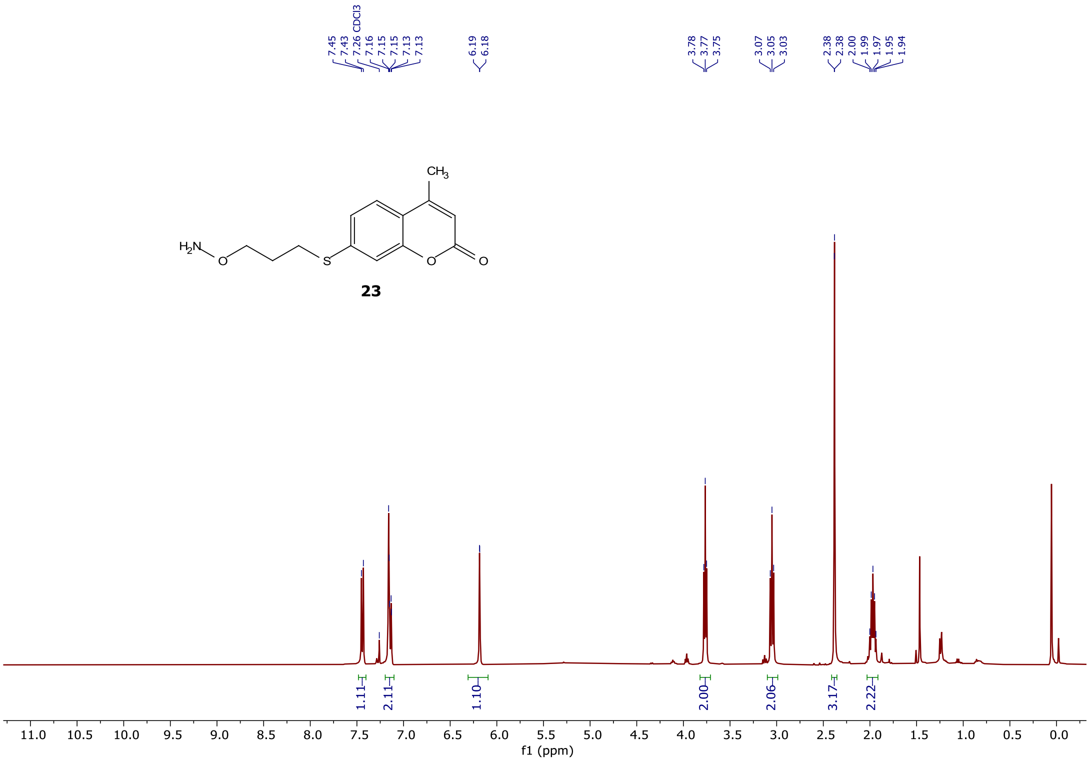

Supplementary Fig. 187. ^{1}H NMR spectrum in $CDCl_3$ of compound **23**.

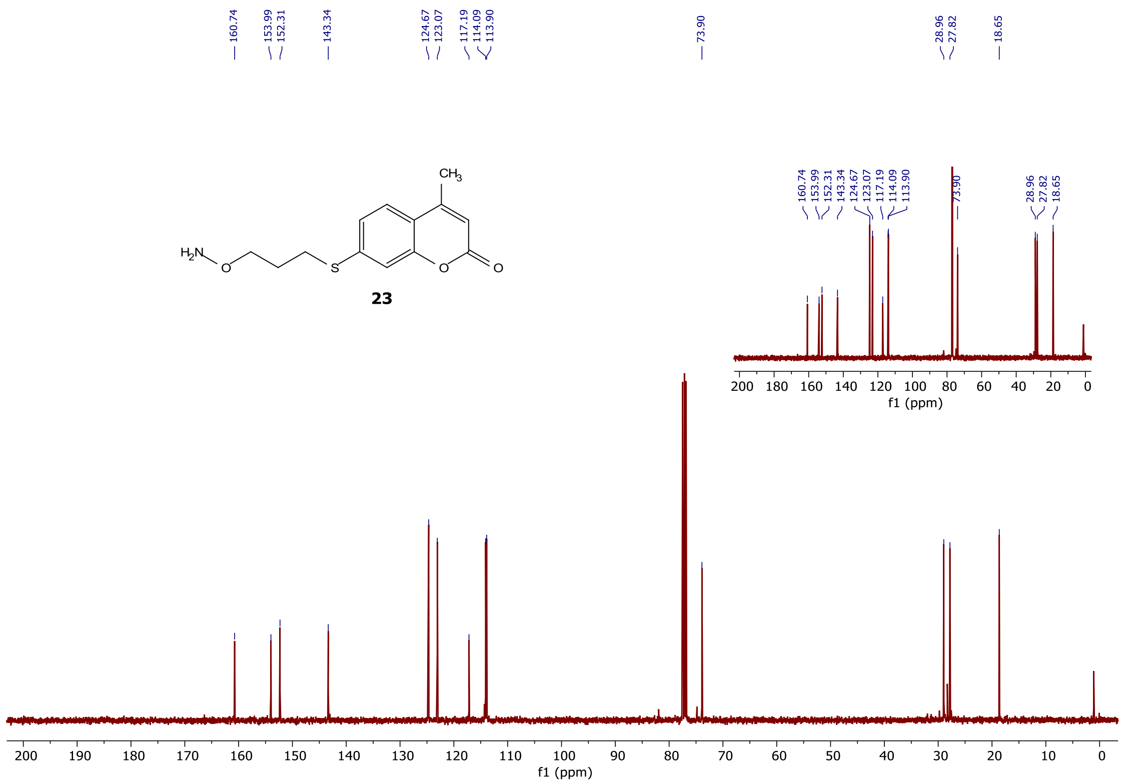

Supplementary Fig. 188. ^{13}C NMR spectrum in $CDCl_3$ of compound **23**.

Supplementary references

1. The PyMOL Molecular Graphics System, Version 2.0 Schrödinger, LLC.
2. Hanwell, M. D. et al. Avogadro: An advanced semantic chemical editor, visualization, and analysis platform. *Journal of Cheminformatics* **2012**, 4:17.
3. Wang, J., Wang, W., Kollman, P. A. & Case, D. A. Automatic atom type and bond type perception in molecular mechanical calculations. *Journal of Molecular Graphics and Modelling* **25**, 247260 (2006).
4. Wang, J., Wolf, R. M.; Caldwell, J. W. & Kollman, P. A. & Case, D. A. Development and testing of a general AMBER force field. *Journal of Computational Chemistry* **25**, 1157–1174 (2004).
5. Jorgensen, W. L.; Chandrasekhar, J.; Madura, J. D.; Impey, R. W. & Klein, M. L. Comparison of simple potential functions for simulating liquid water. *J. Chem. Phys.* **79**, 926–935 (1983).
6. Case, D. A. et al. Amber, University of California, San Francisco (2021).
7. Case, D. A. et al. Amber, University of California, San Francisco (2015).
8. Joung, I. S. & Cheatham, T. E. III Determination of alkali and halide monovalent ion parameters for use in explicitly solvated biomolecular simulations. *J. Phys. Chem. B* **112**, 9020-9041 (2008).
9. Case, D. A. et al. The amber biomolecular simulation programs. *J. Comput. Chem*. **26**, 1668–1688 (2005).
10. Van Gunsteren, W. F. & Berendsen, H. J. C. A leap-frog algorithm for stochastic dynamics. *Mol. Simul*. **1**, 173–185 (1988).
11. Berendsen, H. J. C.; Postma, J. P. M.; van Gunsteren, W. F.; Dinola, A. & Haak, J. R. Molecular-dynamics with coupling to an external bath. *J. Chem. Phys.* **81**, 3684–3690 (1984).
12. Darden, T.; York, D. & Pedersen, L. Particle mesh Ewald: an N.log (*N*) method for Ewald sums in large systems. *J. Chem. Phys.* **98**, 10089–10092 (1993).
13. Ryckaert, J.P.; Ciccotti, G. & Berendsen, H. J. C. Numerical integration of cartesian equations of motion of a system with constraints π molecular-dynamics of N-alkanes. *J. Comput. Phys.* **23**, 327–341 (1977).
14. Humphrey, W.; Dalke, A. & Schulten, K. VMD: Visual molecular dynamics. *J. Mol. Graphics Modell.* **14**, 33–38 (1996).
15. Kinter, M. & Sherman, N. E. *Wiley Interscience* (2000).
16. Nandy, A., Chakraborty, S., Nandy, S., Bhattacharyya, K. & Mukherjee, S. Structure activity, and dynamics of human serum albumin in a crowded pluronic F127 hydrogel. *J. Phys. Chem. B* **123**, 3397−3408 (2019).
17. Lelle, M.; Frick, S. U.; Steinbrinkb, K. & Peneva, K. Novel cleavable cell-penetrating peptide drug conjugates: synthesis and characterization. *J. Pept. Sci.* **20**, 323–333 (2014).

General: The figures were made using ChemOffice, PyMol, and Biorender.
