# Supplementary material for: Traceless cysteine-linchpin enables precision engineering of lysine in native proteins
Source: Nat Commun. 2022 Oct 13;13:6038. doi: 10.1038/s41467-022-33772-1 (PMC9561114; doi:10.1038/s41467-022-33772-1)
Supplement: Supplementary file 2 — Reporting Summary [file 41467_2022_33772_MOESM2_ESM.pdf]

nature portfolio

Corresponding author(s): Vishal Rai

Last updated by author(s): Sep 26, 2022

Reporting Summary

Nature Portfolio wishes to improve the reproducibility of the work that we publish. This form provides structure for consistency and transparency in reporting. For further information on Nature Portfolio policies, see our Editorial Policies and the Editorial Policy Checklist.

Statistics

For all statistical analyses, confirm that the following items are present in the figure legend, table legend, main text, or Methods section.

n/a	Confirmed	
☐	☒	The exact sample size (*n*) for each experimental group/condition, given as a discrete number and unit of measurement
☐	☒	A statement on whether measurements were taken from distinct samples or whether the same sample was measured repeatedly
☒	☐	The statistical test(s) used AND whether they are one- or two-sided<br>*Only common tests should be described solely by name; describe more complex techniques in the Methods section.*
☒	☐	A description of all covariates tested
☒	☐	A description of any assumptions or corrections, such as tests of normality and adjustment for multiple comparisons
☐	☒	A full description of the statistical parameters including central tendency (e.g. means) or other basic estimates (e.g. regression coefficient) AND variation (e.g. standard deviation) or associated estimates of uncertainty (e.g. confidence intervals)
☒	☐	For null hypothesis testing, the test statistic (e.g. *F*, *t*, *r*) with confidence intervals, effect sizes, degrees of freedom and *P* value noted<br>*Give P values as exact values whenever suitable.*
☒	☐	For Bayesian analysis, information on the choice of priors and Markov chain Monte Carlo settings
☒	☐	For hierarchical and complex designs, identification of the appropriate level for tests and full reporting of outcomes
☒	☐	Estimates of effect sizes (e.g. Cohen's *d*, Pearson's *r*), indicating how they were calculated

Our web collection on statistics for biologists contains articles on many of the points above.

Software and code

Policy information about availability of computer code

Data collection	Amber 20, SCIEX OS 1.7, Open LAB CDS A.01
Data analysis	CPPTRAJ module of Amber 20, VMD 1.9.3, Pymol 2.4.1, SCIEX OS 1.7, BioPharmaView Flex 2.1, Bruker Daltonics UltrafleXtreme Software-Flex control version 3.4, Bruker Daltonics ESI Compass 1.3

For manuscripts utilizing custom algorithms or software that are central to the research but not yet described in published literature, software must be made available to editors and reviewers. We strongly encourage code deposition in a community repository (e.g. GitHub). See the Nature Portfolio guidelines for submitting code & software for further information.

Data

Policy information about availability of data

All manuscripts must include a data availability statement. This statement should provide the following information, where applicable:

- Accession codes, unique identifiers, or web links for publicly available datasets
- A description of any restrictions on data availability
- For clinical datasets or third party data, please ensure that the statement adheres to our policy

The datasets generated during and/or analysed during the current study are available in the supplementary information. The raw data has been uploaded on Figshare public repository [DOI: 10.6084/m9.figshare.21219896] and the details have been included in the “Data Availability” statement. Appropriate references have been given in the supplementary information for the publicly available dataset used in the studies.

Human research participants

Policy information about studies involving human research participants and Sex and Gender in Research.

Reporting on sex and gender	Not applicable
Population characteristics	Not applicable
Recruitment	Not applicable
Ethics oversight	Not applicable

Note that full information on the approval of the study protocol must also be provided in the manuscript.

Field-specific reporting

Please select the one below that is the best fit for your research. If you are not sure, read the appropriate sections before making your selection.

- [x] Life sciences
- [ ] Behavioural & social sciences
- [ ] Ecological, evolutionary & environmental sciences

For a reference copy of the document with all sections, see nature.com/documents/nr-reporting-summary-flat.pdf

Life sciences study design

All studies must disclose on these points even when the disclosure is negative.

Sample size	All the experiments were performed in at least three biological replicates. The exact sample size (n) is provided in the figure legends. Statistical tests were not performed to determine the sample size.
Data exclusions	No data were excluded from the analysis.
Replication	All the experiments were performed in at least three biological replicates.
Randomization	The treatment and control groups were attributed randomly between wells/plates for the cell culture experiments.
Blinding	Investigators were blinded for the chemical entities during anti-proliferative assays.

Reporting for specific materials, systems and methods

We require information from authors about some types of materials, experimental systems and methods used in many studies. Here, indicate whether each material, system or method listed is relevant to your study. If you are not sure if a list item applies to your research, read the appropriate section before selecting a response.

Materials & experimental systems

n/a	Involved in the study	
[ ]	[x]	Antibodies
[ ]	[x]	Eukaryotic cell lines
[x]	[ ]	Palaeontology and archaeology
[x]	[ ]	Animals and other organisms
[x]	[ ]	Clinical data
[x]	[ ]	Dual use research of concern

Methods

n/a	Involved in the study	
[x]	[ ]	ChIP-seq
[x]	[ ]	Flow cytometry
[x]	[ ]	MRI-based neuroimaging

Antibodies

Antibodies used	Trastuzumab (Emcure, N7123B01), Kadcyla (Roche, N1037B18)
Validation	Validated by mass spectrometry

Eukaryotic cell lines

Policy information about cell lines and Sex and Gender in Research

Cell line source(s)	SKBR-3 from NCCS Pune, MDAMB-231 from ATCC
Authentication	MDAMB-231 cell line was authenticated by STR profiling in Nov 2021. The SKBR-3 cell line was procured from NCCS Pune at the time of experiments and was not authenticated.
Mycoplasma contamination	All cell lines tested were mycoplasma negative
Commonly misidentified lines (See ICLAC register)	No commonly misidentified cell lines were used in the study.
